# HIV Glasgow 2018, 28–31 October 2018, Glasgow, UK

**DOI:** 10.1002/jia2.25187

**Published:** 2018-10-26

## KL1

### HIV/AIDS, global health and the Sustainable Development Goals

**K De Cock**

CDC Country Office, US Centers for Disease Control and Prevention, Nairobi, Kenya

Sustainable Goal (SDG) 3 calls for an end to the epidemics of AIDS, tuberculosis, malaria and neglected tropical diseases by 2030, and the concomitant UNAIDS Fast‐Track Strategy aims to reduce new HIV infections to no more than 500,000 annually by 2020 and 200,000 by 2030. Central to the global effort is the UNAIDS 90‐90‐90 initiative which requires 90% of persons with HIV to be diagnosed, 90% of those to receive ART and 90% of the treated to be virally suppressed. There is controversy around how “the end of AIDS” is defined, about whether this ambitious goal is achievable and whether AIDS exceptionalism is still appropriate. UNAIDS has targeted 30 million people to be on ART by 2020, when fiscal requirements are expected to be 26 billion US dollars annually; current expenditure is about 7 billion US dollars less. This presentation will review progress in the AIDS response in the overall context of current global health. It honours Jacqueline Van Tongeren and Joep Lange and their work, and is dedicated to their memory.

## KL2

### Strategies to reduce HIV incidence in Europe

**A Pharris**

European Centre for Disease Prevention and Control (ECDC), Stockholm, Sweden

HIV incidence is increasing in the European region as a whole, although there are large epidemiological differences between Western, Central and Eastern Europe. Whilst overall 80% of people in the European region have been diagnosed with HIV, this varies greatly across sub‐regions with 86%, 83% and 76% of people diagnosed in Western, Central and Eastern Europe respectively. Among those diagnosed, 64% are estimated to be on treatment and this, too, differs across the region with 90%, 73% and 46% of those diagnosed on treatment in Western, Central and Eastern sub‐regions, respectively. Among those on treatment in the European region, 85% are virally suppressed with variations across sub‐regions in Europe (92%, 78% and 74% in Western, Central and Eastern). Within sub‐regions and among key populations within countries there is considerable diversity in diagnosis, proportion on treatment and viral suppression rates. While some countries within the region have been successful in meeting and surpassing the 90‐90‐90 targets, others are facing enormous challenges and are lagging behind. While the tools to prevent HIV – including diversified testing strategies, treatment as prevention, PrEP and harm reduction – have multiplied in recent years, their application across Europe is uneven and, in most settings, far lower than needed to impact incidence. Differences in epidemiology of HIV and health systems across Europe necessitate context‐specific strategies to strengthen and control HIV prevention and care efforts.

## KL3

### PrEP: what's happening in Europe and the world in general

**S McCormack**

MRC Clinical Trials Unit, University College London, London, UK

Within and beyond Europe, PrEP is undoubtedly contributing to the decline in new diagnoses reported in gay and other MSM, but the public health benefit is difficult to assess precisely and the impressive decline seen in some city clinics is not universal. San Francisco, central London and New South Wales have seen the largest gains. In all these settings testing and treatment were already at scale when PrEP was introduced. The contribution of PrEP to the toolkit is most accurately captured in New South Wales where they observed a 35% reduction in state‐wide new HIV diagnoses in MSM following rapid scale‐up of PrEP in the EPIC trial, two seroconversions amongst 3927 years of follow‐up amongst trial participants [1]. TDF/FTC PrEP is extremely effective biologically, but it is costly and needs to be delivered as part of a comprehensive package of interventions to reduce the risk of sexually transmitted infections including HIV – a package that is not available to everyone in Europe or globally in spite of the current burden of sexually transmitted infections. Introducing PrEP is therefore an opportunity to strengthen prevention services, and one of the most cost‐efficient methods is to employ key populations to deliver services when and where convenient to eligible peers (AIDS 2018). Adherence remains the Achilles heel for PrEP, and the products in the pipeline may go some way to addressing this: vaginal rings, long‐acting injectables and implants. However, first and foremost is the need to empower key populations with the information they need to understand their risk of HIV/STIs and how to reduce this during the various phases of their sexual lifetime.

Abstract KL3 – Figure 1. Status of formal PrEP implement in Europe.

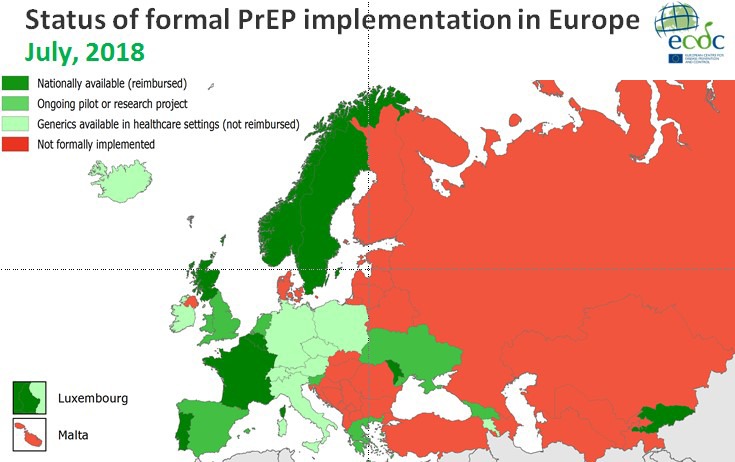

**Reference**

[1] Grulich et al. Rapid reduction in HIV diagnoses after targeted PrEP implementation in NSW, Australia. CROI 2018; Abs 88.

## Oral Abstracts O11 – Living Well with HIV: Ongoing Challenges

## O111

### Retention and re‐engagement in care: a combination approach again required

**F Burns**

Centre for Sexual Health and HIV Research, and Royal Free Hospital, London, UK

Effective ART remains the cornerstone of successful HIV management, with life expectancy in those successfully treated similar to that of the general population. ART is also an effective means of reducing population HIV transmission with the goal of zero new infections. However, suboptimal engagement in HIV care threatens to derail this success and is associated with serious consequences for both individual and public health. Engagement in care for any individual is dynamic and disengagement may happen at any time. Indeed, in the UK as many as one in four HIV clinic appointments are missed. While ‘living well with HIV’ is the current mantra, it is still denied to many. The population groups most at risk of disengagement are invariably those most marginalised and with the least advocacy. They include people who struggle with HIV‐related stigma, those with insecure residency and/or employment and people living with mental health, alcohol and drug dependency issues – problems that may increase in the current political and economic environment. Sustainable engagement will require a combination of biomedical, behavioural and structural strategies that recognise and address individual level factors and create a more enabling environment for health over the life course. Community participation and partnership in this process will be vital, with peer and social support services playing a key role. To tailor and target interventions appropriately, mechanisms are needed to predict those at risk of subsequent disengagement as well to respond once this occurs. Effective mechanisms for predicting and monitoring engagement are either limited or lacking, and the pool of evidence‐based interventions to improve engagement small. Future investment in research and services to tackle engagement is required to ensure the health inequalities we see across our cohorts reduced.

## O112

### HIV and aging: challenges and goals

**J Falutz**

Department of Medicine, McGill University, Montreal, Canada

Currently, overall long‐term survival of treated PLWHIV world‐wide approaches that of the general population. An increasing minority will live as long as their seronegative peers. As a result, the average age of PLWHIV, currently in the mid‐50s in resource‐rich countries, has increased. The proportion of older PLWHIV who are long‐term survivors compared to those who seroconvert at an older age varies according to local factors. The salutary impact on survival has nevertheless been challenged by several developments. The increasing proportion of PLWHIV approaching a typical geriatric age range will significantly impact health care delivery; their clinical features are similar to that of the general population about 5 to 10 years older. In addition to the earlier occurrence of common age‐related conditions, with increased multimorbidity compared to controls, several common geriatric syndromes have also impacted this younger population. These often difficult‐to‐evaluate and ‐manage conditions may include: sarcopenia, impaired mobility and falls, sensory complaints (neuropathy, visual and auditory deficits), cognitive decline and, significantly, frailty. This latter condition, a state of increased vulnerability to biologic and environmental stressors, with reduced ability to maintain homeostasis, remains challenging to evaluate and operationalize. In the general population, a simple and reliable metric to diagnose frailty in the usual clinical setting remains elusive. This is compounded by the poorly understood biologic basis for frailty, distinct from its increased risk of concurrent disabilities and comorbidities. Research into common determinants of frailty between the geriatric population and PLWHIV related to immune‐senescence, chronic inflammation, epigenetics and mitochondriopathy provide clues to potential avenues for prevention and management. Frailty may be key to understanding the discordance between chronologic and biologic age. Concurrently, investigation of predictors of successful aging in PLWHIV is progressing. Insights into the concepts of both psychological and physical resilience in seronegatives may be an important bridge contributing not only to increased lifespan but also to improved health‐span for PLWHIV.

## O113

### 48‐week changes in biomarkers in subjects with high cardiovascular risk switching from ritonavir‐boosted protease inhibitors to dolutegravir: the NEAT022 study

**E Martinez^1^, L Assoumou^2^, G Moyle^3^, L Waters^4^, M Johnson^5^, P Domingo^6^, J Fox^7^, H Stellbrink^8^, G Guaraldi^9^, M Masia^10^, M Gompels^11^, S de Wit^12^, E Florence^13^, S Esser^14^, F Raffi^15^, A Pozniak^3^, J Gatell^16^**

^1^Infectious Diseases, Hospital Clínic & University of Barcelona, Barcelona, Spain. ^2^Institut Pierre Louis d’Épidémiologie et de Santé, INSERM, Sorbonne Universités, Paris, France. ^3^St Stephen's AIDS Trust, Chelsea and Westminster Hospital, London, UK. ^4^Mortimer Market Centre, Central and North West London NHS Foundation Trust, London, UK. ^5^Infectious Diseases, Royal Free Hospital, London, UK. ^6^Infectious Diseases Unit, Hospital de la Santa Creu i Sant Pau, Barcelona, Spain. ^7^Infectious Diseases, Guy's and St Thomas’ Hospital, London, UK. ^8^Infectious Diseases, Infektionsmedizinisches Centrum, Hamburg, Germany. ^9^Infectious Diseases, University of Modena and Reggio Emilia, Modena, Italy. ^10^Infectious Diseases Unit, Hospital de Elche, Elche, Spain. ^11^Infectious Diseases, Southmead Hospital, Bristol, UK. ^12^Infectious Diseases, Saint Pierre Hospital, Universite Libre de Bruxelles, Brussels, Belgium. ^13^Infectious Diseases, Institute of Tropical Medicine, Antwerp, Belgium. ^14^Infectious Diseases, Universitatsklinikum, Essen, Germany. ^15^Infectious Diseases, University Hospital of Nantes, Nantes, France. ^16^Medicine, University of Barcelona, Barcelona, Spain

**Background: **Switching from ritonavir‐boosted protease inhibitors (PI/r) to dolutegravir (DTG) in subjects with a high cardiovascular risk resulted in a better lipid profile at 48 weeks than continuing PI/r. Whether this strategy may have an impact on biomarkers involved in the pathogenesis of cardiovascular disease in HIV‐infected subjects is unknown.

**Materials and methods: **Within a pre‐planned sub‐study, we assessed 48‐week changes in several biomarkers including serum high‐sensitivity C reactive protein (hsCRP), interleukin‐6 (IL‐6), intercellular adhesion molecule‐1 (ICAM‐1), vascular adhesion molecule‐1 (VCAM‐1), selectin E and P, adiponectin, insulin, oxidised LDL, malondialdehyde, soluble CD14 (sCD14) and CD163 (sCD163), and cystatin C, and urine beta‐2 microglobulin. The median percent changes from baseline were compared with Mann‐Whitney test, and the association between the percent changes in biomarkers and lipid fractions or other variables of interest with Spearman correlation test. All *p* values were two‐sided with a significance level of 0.01 to account for the multiplicity of tests.

**Results: **Of 415 randomised patients, 313 (147 DTG, 166 PI/r) remained on their allocated therapy for 48 weeks and had samples available. We observed significant decreases in sCD14 (−11%, *p* < 0.001) and adiponectin (−11%, *p* < 0.001), and a trend to decrease in hsCRP (−13%, *p* = 0.069) and oxidised LDL (−13%, *p* = 0.084) in the DTG group relative to PI/r group. Percent change in sCD14 was inversely correlated with percent change in CD4 count (coefficient ‐0.113, *p* = 0.049). Median (IQR) CD4 cell (/mm^3^) change was +32 (‐66 to 109) in DTG arm and ‐6 (‐87 to 73) in PI/r arm (*p* = 0.049). Percent change in adiponectin was inversely correlated with percent change in body mass index (BMI) (coefficient ‐0.227, *p* < 0.001). Median (IQR) baseline BMI (kg/m^2^) was 25.7 (23.4 to 28.0) in DTG arm and 26.1 (23.5 to 28.2) in PI/r arm (*p* = 0.907). Median (IQR) BMI (kg/m^2^) change was +0.3 (‐0.4 to 1.1) in DTG arm and +0.2 (‐0.7 to 0.8) in PI/r arm (*p* = 0.121).

**Conclusions: **Switching from a PI/r‐containing to a DTG‐containing regimen in virologically suppressed HIV‐infected adults with a high cardiovascular risk decreased sCD14 but also adiponectin at 48 weeks. sCD14 and adiponectin reductions may have opposite decreasing [1] and increasing [2] cardiovascular effects in HIV‐infected subjects. Although the overall cardiovascular impact of the NEAT022 study switching strategy was positive [3], the decrease in adiponectin was associated with BMI gain and this sub‐study highlights the importance of further assessing the potential impact of DTG therapy on the mechanisms involved in body weight.

**References**

[1] Longenecker CT, Jiang Y, Orringer CE, Gilkeson RC, Debanne S, Funderburg NT, et al. Soluble CD14 is independently associated with coronary calcification and extent of subclinicalvascular disease in treated HIV infection. AIDS 2014;28:969‐77.

[2] Ketlogetswe KS, Post WS, Li X, Palella FJ Jr, Jacobson LP, Margolick JB, et al. Lower adiponectin is associated with subclinical cardiovascular disease among HIV‐infected men. AIDS 2014;28:901‐9.

[3] Gatell JM, Assoumou L, Moyle G, Waters L, Johnson M, Domingo P, et al. Switching from a ritonavir‐boosted protease inhibitor to a dolutegravir‐based regimen for maintenance of HIV viral suppression in patients with high cardiovascular risk. AIDS 2017;31:2503‐14.

## O114

### Risk of hospitalisation according to gender, sexuality and ethnicity among people with HIV in the modern ART era

**S Rein^1^, F Lampe^1^, M Johnson^2^, C Chaloner^1^, F Burns^1^, S Madge^2^, A Phillips^1^, C Smith^1^**

^1^Institute for Global Health, University College London, London, UK. ^2^HIV Medicine, Royal Free Hampstead NHS Trust, London, UK

**Background: **There has been little research on the impact of gender and sexual orientation on hospitalisations in HIV‐positive people in the UK in the modern ART era.

**Materials and methods: **All HIV‐diagnosed individuals attending the Royal Free Hospital, London, from 2007 onwards were followed until 2016. Rates of all‐cause hospitalisation in the first year after diagnosis (analysis A) and from Year 1 onwards (analysis B) were calculated according to gender/sexuality/ethnicity and adjusted for demographic and clinical factors using Cox and Poisson regression respectively. Repeated hospitalisations were permitted in analysis B.

**Results: **For analysis A, 166 hospitalisations occurred in 1307 newly‐diagnosed individuals. Forty‐four percent, 55% and 46% of hospitalisations in MSM, men who have sex with women (MSW) and women were AIDS‐related. The higher hospitalisation rate in MSW and women compared to MSM was only partially explained by CD4 count and other factors (Table 1). Lower CD4, older age and earlier diagnosis date were independently associated with higher hospitalisation rate. For analysis B, 4211 individuals diagnosed for >1 year contributed 773 hospitalisations from 553 individuals. Seven percent, 18% and 10% of hospitalisations in MSM, MSW and women were AIDS‐related. Non‐Black MSW and women remained at higher risk of hospitalisation, but the association was weaker than that seen in the first year after diagnosis (Table 1). Lack of viral suppression, lower CD4, older age and earlier diagnosis date were also independently associated with hospitalisations.

**Table:** **Abstract O114 –** Table 1. Association between gender / sexual orientation & ethnicity and all‐cause hospitalisation rate in HIV‐positive individuals in the first year after diagnosis (analysis A) and >1 year after diagnosis (analysis B)

	A: Hospitalisation in first year after diagnosis				B: Hospitalisations from one year after diagnosis			
	N (PY)	Rate^a^	Unadjusted HR (95% CI)	Adjusted HR (95% CI)^b^	N (PY)	Rate^a^	Unadjusted RR (95% CI)	Adjusted RR (95% CI)^c^
MSM	655 (592)	6.1	1.0	1.0	2310 (15,013)	2.2	1.0	1.0
Black MSW	138 (103)	27.2	4.2 (2.5 to 6.8)	2.4 (1.5 to 4.0)	391 (2514)	3.7	1.7 (1.4 to 2.2)	1.1 (0.9 to 1.4)
Other ethnicity MSW	169 (127)	30.6	4.7 (3.0 to 7.5)	3.2 (2.0 to 5.1)	431 (2314)	4.8	2.2 (1.8 to 2.8)	1.8 (1.4 to 2.2)
Black women	245 (185)	25.4	3.8 (2.5 to 5.9)	2.4 (1.6 to 3.8)	752 (4621)	3.7	1.7 (1.4 to 2.0)	1.4 (1.1 to 1.7)
Other ethnicity women	100 (81)	19.8	3.1 (1.7 to 5.6)	2.3 (1.3 to 4.2)	327 (2090)	3.3	1.5 (1.2 to 2.0)	1.4 (1.1 to 1.9)

All *p* values <0.0001. ^a^per 100 person‐years; adjusted for: ^b^age, diagnosis year, 1st visit CD4; ^c^age, current CD4, current CD4 nadir, current viral non‐suppression, time since diagnosis, previous AIDS. HR = hazard ratio; RR = rate ratio.

**Conclusions: **MSW and women have increased rate of hospitalisation in the modern ART era partially independent of clinical factors. Reasons for these variations in clinical outcomes should be investigated further to establish whether targeted interventions are needed.

## O115

### Multimorbidity and risk of death differs by gender in people living with HIV in the Netherlands: the ATHENA cohort study

**F Wit^1^, M van der Valk^2^, J Gisolf^3^, W Bierman^4^, P Reiss^2^**

^1^Stichting HIV Monitoring, Academic Medical Center, Amsterdam, Netherlands. ^2^Department of Internal Medicine, Academic Medical Center, Amsterdam, Netherlands. ^3^Department of Internal Medicine, Rijnstate Ziekenhuis, Arnhem, Netherlands. ^4^Department of Internal Medicine – Infectious Diseases, University Medical Center Groningen, Groningen, Netherlands

**Background: **PLWHIV on cART are living longer and because of ageing are experiencing more non‐AIDS comorbidities, which have become the most common cause of death in PLWHIV on cART. We investigated if multimorbidity predicts mortality in PLWHIV on cART and whether this differs by gender.

**Materials and methods: **We used data from PLWHIV from the ATHENA cohort collected from 2000 to 2016. Comorbidities identified were: cardiovascular disease; stroke; non‐AIDS malignancies, excluding non‐melanoma skin cancers and pre‐malignant cervical/anal lesions; moderate‐severe chronic kidney disease (eGFR <30 mL/min ≥6 months, Grade ≥G3b); diabetes mellitus; hypertension (use of antihypertensives or blood pressure ≥160/100 mmHg); obesity (BMI >30). Poisson regression compared mortality between genders adjusting for demographics, traditional risk factors and HIV‐related parameters.

**Results: **Data from 24,383 PLWHIV (19.2% females) were included (see Table 1). At cART initiation the mean number of non‐AIDS comorbidities in males (0.26) and females (0.25) were similar (*p* = 0.34). At last available follow‐up in 2016 the mean number of comorbidities had increased in both males (0.59) and females (0.59), *p* = 0.18. Mortality risk increased with number of comorbidities, from 6.83 deaths per 1000 person‐years in PLWHIV with zero comorbidities, to 13.8, 28.2, 65.6 and 139 per 1000 person‐years with 1, 2, 3, ≥4 comorbidities, respectively. Poisson regression confirmed the relationship between multimorbidity and mortality: risk ratio (RR) 2.66 (2.54 to 2.79) per additional comorbidity. Overall mortality risk, adjusted for the number of comorbidities, was significantly lower in women than men (RR 0.78 [0.67 to 0.91], *p* = 0.002). However, there was a significant interaction between gender, number of comorbidities and mortality (*p* < 0.0001) with the RR for women compared to men, ranging from 0.57 (0.45 to 0.74), to 0.75 (0.59 to 0.95), to 1.00 (0.74 to 1.38), to 1.52 (1.00 to 2.32), and 1.77 (0.83 to 3.78) for those with 0, 1, 2, 3, ≥4 comorbidities. Every individual comorbidity, except non‐AIDS malignancies, carried excess mortality risk for women. Excess mortality in women with more extensive multimorbidity was driven partly by exposure to mono‐ and dual nucleoside analogues before the cART era, as the increased risk attenuated and lost statistical significance after excluding PLWHIV pre‐treated with nucleoside analogues before start of cART: RR for women compared to men at three and four comorbidities were 1.39 (0.87 to 2.23) and 1.17 (0.47 to 2.91), respectively.

**Table:** **Abstract O115 –** Table 1. All results are presented as percentages or as median (IQR)

	Females	Males	*p* value
Characteristics at entry into the cohort			
Number of participants	4687 (19.2%)	19,696 (80.8%)	‐
Age	33.5 (27.7 to 40.9)	39.3 (32.5 to 47.1)	<0.0001
Dutch nationality	27.1%	63.1%	<0.0001
Transmission category	<0.0001
MSM	‐	73.4%
Heterosexual	87.8%	17.0%
Injecting drug use	4.2%	2.7%
Other	8.0%	6.9%
Smoking status	<0.0001
Never	42.0%	27.5%
Current	21.0%	36.6%
Past	8.8%	12.8%
Missing	28.3%	23.1%
Chronic HBV infection	3.9%	5.1%	0.0003
Chronic HCV infection	7.1%	5.2%	<0.0001
CD4 count (cells/mm^3^)	310 (171 to 490)	323 (200 to 482)	0.0002
Viral load (log10 copies/mL)	3.6 (2.4 to 4.7)	4.2 (2.6 to 5.0)	<0.0001
Characteristics at start cART			
Pre‐treated with nucleosides before start cART	7.6%	8.9%	0.0002
Years known HIV‐positive	0.5 (0.2 to 3.2)	0.8 (0.3 to 3.5)	<0.0001
CD4 count (cells/mm^3^)	270 (150 to 410)	290 (160 to 430)	<0.0001
Duration of prior CD4 count <200 (years)	0.0 (0.0 to 0.23)	0.0 (0.0 to 0.21)	<0.0001
Viral load (log10 copies/mL)	4.1 (2.9 to 4.9)	4.6 (3.5 to 5.1)	<0.0001
Prior AIDS diagnosis	14.3%	14.5%	0.76

**Conclusions: **Multimorbidity was a strong independent predictor of mortality in adult PLWHIV. Although women in general and especially women with less than three comorbidities had lower mortality than men, their risk was reversed and increased compared to men when experiencing three or more comorbidities.

## O116

### The economic burden of comorbidities among people living with HIV in Germany: a cohort analysis using health insurance claims data

**E Wolf^1^, S Christensen^2^, H Diaz‐Cuervo^3^**

^1^MUC Research, Munich, Germany. ^2^Infectious Diseases, Center for Interdisciplinary Medicine, Münster, Germany. ^3^HEOR, Gilead Sciences, London, UK

**Background: **Current treatment options for HIV increased life expectancy of PLWHIV. Therefore, management of non‐HIV related comorbidities became an essential part of HIV care. Although the clinical burden of comorbidities is well described in PLWHIV, data on the economic burden of comorbidities are limited. This analysis estimated the cost of acute and chronic non‐HIV related comorbidities by using a large health insurance claims database in Germany.

**Materials and methods: **The German InGef health insurance claims database was used to identify a cohort of adult patients with HIV diagnosis record within every calendar between 1 January 2011 and 31 December 2014. HIV infection and comorbidities were detected using ICD‐10‐GM codes (see Figure 1 for evaluation periods). Total costs (Euro, €) including outpatient, inpatient, medication costs were evaluated during the last available 1‐year period before study end (31 December 2015), date of death or loss to follow‐up. A multivariable GLM regression model using log‐link function and gamma distribution was used to estimate the contribution of comorbidity to total healthcare costs excluding ART costs. The model included patient demographics, the clinical conditions used in Charlson comorbidity index (CCI), other chronic comorbidities not overlapping with CCI and acute comorbidities (i.e. acute/chronic cardiovascular disease excluding congestive heart failure, acute/chronic hepatitis B or C, alcohol abuse, bone fractures due to osteoporosis, dyslipidaemia and hypertension). The results of statistically significant estimators (using backward selection) are presented here.

**Results: **Two thousand one hundred and five patients met eligibility criteria (82.6% male, median age 47.8 years, 41.6% >50 years). Average number of acute and chronic comorbidities was 0.3 (range 0 to 3) and 1 (range 0 to 6), respectively. Mean annual total healthcare costs including ART were 22,817€, excluding ART 7609€; mean inpatient costs were 1467€, mean outpatient costs were 1589€, mean medication costs excluding ART were 4196€, and mean ART costs were 14,232€. Estimated incremental annual costs were 4791€ for acute cardiovascular disease, 14,525€ for acute hepatitis C, 7366€ for acute renal disease, 3511€ for bone fractures due to osteoporosis, 16,023€ for chronic hepatitis C, 2581€ for diabetes mellitus type 2, ‐867€ for dyslipidaemia and ‐1678€ for being female (Table 1, Figure 2).

**Abstract O116 – Figure 1**. Evaluation periods. CCI = Charlson comorbidity index.

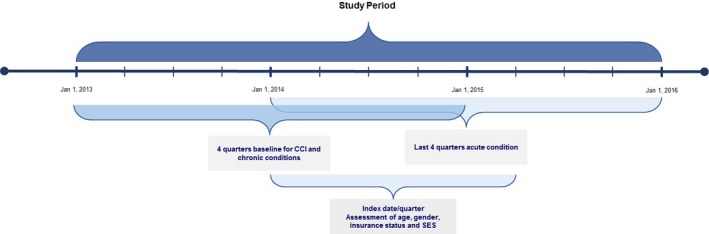

**Abstract O116 – Figure 2**. Incremental cost estimates of acute and chronic non‐HIV related comorbidities.

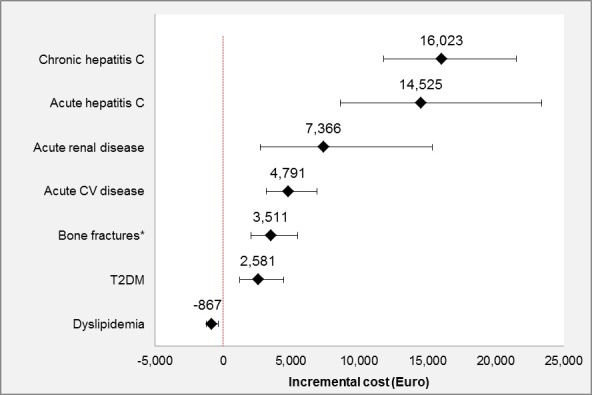

**Table:** **Abstract O116 –** Table 1. Prevalence and incremental cost estimates for acute and chronic non‐HIV related comorbidities included in the final model

Comorbidities	Prevalence	Incremental cost (€)	95% CI of cost (€) (lower)	95% CI of cost (€) (upper)	*p* value
Intercept	4766	4460	5093
Gender, being female	17.4%	‐1,678	‐1929	‐1327	<0.001
Acute conditions					
Acute cardiovascular disease	12.7%	4791	3165	6884	<0.001
Acute hepatitis C	2.4%	14,525	8618	23,360	<0.001
Acute renal disease	1.3%	7366	2742	15,340	<0.001
Bone fractures due to osteoporosis	9.0%	3511	2020	5481	<0.001
Chronic conditions					
Chronic hepatitis C	8.5%	16,023	11,771	21,535	<0.001
Diabetes mellitus type 2	8.3%	2581	1214	4421	<0.001
Dyslipidaemia	23.5%	‐867	‐1250	‐356	0.002
Moderate or severe liver disease	0.6%	0.438

ART = antiretroviral treatment. Evaluation of the model appropriateness; AIC = 40,587, Box Cox λ = 0, Park test estimate = 2.24, scaled deviance/df =1.2.

**Conclusions: **This multivariable GLM regression model using claims data in Germany revealed the high economic impact of some comorbidities with estimated incremental annual costs ranging from 2581€ to 16,023€. These results support the importance of management of comorbidity in PLWHIV to decrease medical and economic burden.

## O13 – Lock Lecture

## O131

### STIs among MSM: new challenges in prevention, diagnosis and treatment

**J Molina**

Department of Infectious Diseases and University of Paris 7, St Louis Hospital, Paris, France

The incidence of sexually transmitted infections (STIs), both bacterial (due to *Chlamydia trachomatis, Neisseria gonorrheae, Mycoplasma genitalium* and *Treponema pallidum*) and viral (due to viral hepatitis A and C and human papillomaviruses), is increasing worldwide, especially in MSM, and represents a major public health concern. Indeed, the advances in the treatment and prevention of HIV infection over the last couple of years have led to an increase in risk compensation with less consistent use of condoms in this population. Although these high rates of STIs among MSM predated the implementation of oral PrEP and do not undermine its efficacy against HIV, they represent a new challenge in prevention, diagnosis and treatment. This review will first address the different approaches that could be proposed today to reduce the rate of STIs in a context of low condom use, and will include prospects for future strategies including antibiotic prophylaxis and vaccines for STIs. Also, most guidelines have updated their recommendations to encourage systematic testing for STIs every three months among MSM with high‐risk behaviour, and implementation of this recommendation combined with early treatment and partner notification should lead to a decrease in STI rates. The different diagnostic tools available and in development will be presented with a focus on point‐of‐care tests and tests that allow detection of antimicrobial resistance to guide treatment. Indeed, one of the main challenges with bacterial STIs is the increasing rate of antibiotic resistance, especially for gonorrhoea and *Mycoplasma genitalium*. National networks for surveillance of antimicrobial resistance need to be implemented and supported. Also, new antibiotics need to be developed to overcome the limitations of current treatment strategies. Altogether, there is a need to foster research in STIs to address all these challenges and meet the World Health Organization global targets for STIs in 2030, such as a 90% reduction of the global incidence of gonorrhoea and syphilis.

## O14 – Drug Interactions, ARV Toxicity and Switching

## O141

### The top 10 DDIs in day‐to‐day clinical management of HIV

**C Marzolini**

Division of Infectious Diseases and Hospital Epidemiology, University Hospital Basel, Basel, Switzerland

Large surveys suggest that up to a quarter of HIV‐infected patients may be at risk of a clinically significant drug‐drug interaction (DDI) even with the latest antiretroviral treatments. Antiretroviral drugs are recognised to be amongst the therapeutic agents with the highest potential for DDIs as these drugs can be both a perpetrator and a victim of DDIs. Common mechanisms of DDIs with antiretroviral agents involve inhibition or induction of drug metabolism enzymes or drug transporters as well as chelation with divalent cations or pH‐dependent changes in drug absorption. DDIs can lead to drug toxicity or treatment failure of the antiretroviral drug and/or the co‐administered drug. DDIs are practically unavoidable in HIV care given the life‐long antiretroviral treatments and the growing prevalence of non‐HIV polypharmacy particularly in the context of an ageing HIV population. Thus, the identification, prevention and management of DDIs should remain a key priority in HIV care. The potential for DDIs needs to be considered systematically when selecting an antiretroviral regimen or when adding any new comedication to an existing HIV treatment with particular attention to adjust dosage or perform clinical monitoring when needed. In this regard, searchable online drug interactions databases constitute valuable tools to recognise and manage unwanted DDIs in clinical practice. In addition, educational programmes should be encouraged to improve awareness on the issue of DDIs and thereby prevent deleterious drug effects. This presentation will review the clinical management including mechanistic aspects of 10 selected clinically relevant DDIs between antiretroviral agents and non‐HIV drugs.

## O142

### Adverse effects of current ARVs: myths and realities

**P Mallon**

School of Medicine, University College Dublin, Dublin, Ireland

Advances in antiretroviral drug development have helped the majority of people living with HIV‐1 infection with access to current antiretroviral therapy (ART) to realise long‐term treatment goals of near‐normal life span with limited drug‐related toxicity. Often, discrimination between which ART to use, in both treatment initiation and switch, is based on knowledge of subclinical toxicities that may not necessarily have clinically meaningful impact for the individual. Examples of these toxicities include laboratory or imaging changes such as proteinuria and loss of bone density. Switch for potential subclinical toxicity carries with it a risk of unintentional introduction of new clinical problems, which for a particular individual may have important clinical consequences. Additionally, some new compounds may have toxicities that remain ill‐defined or are still emerging as they become more widely used in populations underrepresented in registration clinical trials. Understanding these nuances is central to the appropriate use of antiretrovirals in treatment of chronic HIV‐1 infection. This presentation will discuss common toxicities, provide evidence behind associations between toxicities and specific antiretrovirals and place these within a relevant clinical context to provide a framework for healthcare providers to consider the best regimen for an individual person living with HIV.

## O143

### Meta‐analysis of the risk of Grade 3/4 or serious clinical adverse events in 12 randomised trials of PrEP (n = 15,678)

**V Pilkington^1^, A Hill^2^, S Hughes^3^, N Nkwolo^4^, A Pozniak^4^**

^1^Faculty of Medicine, Imperial College London, London, UK. ^2^Department of Translational Medicine, University of Liverpool, Liverpool, UK. ^3^Research, Metavirology Ltd, London, UK. ^4^Chelsea and Westminster Hospital, London, UK

**Background: **TDF/FTC used as pre‐exposure prophylaxis (PrEP) has proven benefits in preventing HIV infection. Generic formulations of TDF/FTC are available for <$60 per person‐year in low‐income countries. Widespread use of TDF/FTC can only be justified if the preventative benefits outweigh potential risks of adverse events. A previous meta‐analysis of TDF/FTC compared to alternative TAF/FTC for treatment found no significant difference in safety endpoints [1], but more evidence around the safety of TDF/FTC is needed to address concerns and inform widespread use.

**Methods: **A systematic review identified 13 randomised trials of PrEP, using either TDF/FTC or TDF, versus placebo or no treatment: VOICES, PROUD, IPERGAY, FEM‐PrEP, TDF‐2, iPREX, IAVI Kenya, IAVI Uganda, PrEPare, PARTNERS, US Safety study, Bangkok TDF study, W African TDF study. The number of participants with Grade 3/4 adverse events or serious adverse events (SAEs) was compared between treatment and control in meta‐analysis. Further analyses of specific renal and bone markers were also undertaken, with fractures as a marker of bone effects and creatinine elevations as a surrogate marker for renal impairment. Analyses were stratified by study duration (one year of follow‐up).

**Results: **The 13 randomised trials included 15,678 participants in relevant treatment and control arms. Three studies assessed TDF use only. The number of participants with Grade 3/4 adverse events was 1305/7504 (17.4%) on treatment versus 1259/7502 (16.8%) on control (difference 0%, 95% CI ‐1% to +2%). The number of participants with SAEs was 738/7843 (9.4%) on treatment versus 795/7835 (10.1%) on no treatment (difference 0%, 95% CI ‐1% to +1). Similarly, adverse renal and bone outcomes did not occur significantly more often in participants taking PrEP versus control regimens (difference 0%, 95% CI 0% to 0%). There was no difference in outcome between studies with <1 versus >1 year of randomised treatment.

**Conclusions: **In this meta‐analysis of 13 randomised clinical trials of PrEP in 15,678 participants, there was no significant difference in risk of Grade 3/4 clinical adverse events or SAEs between TDF/FTC (or TDF) and control (Table 1). Furthermore, there was no significant difference in risk of specific renal or bone adverse outcomes. The safety profile of TDF/FTC would support more widespread use of PrEP in populations with a lower risk of HIV infection.

**Table:** Abstract O143 – Table 1. Results of the meta‐analyses of overall risk difference between treatment and control study arms for each outcome of interest

Outcome	Events (PrEP)	Total participants (PrEP)	Events (control)	Total participants (control)	Risk difference (95% CI)	Significance
Grade 3/4 adverse events	1305	7504	1259	7502	0% (‐1% to 2%)	*p* = 0.56
Serious adverse events	738	7843	795	7835	0% (‐1% to 1%)	*p* = 0.74
Renal (creatinine elevations)	11	7620	5	7622	0% (0%‐0%)	*p* = 0.38
Bone (fractures)	202	5588	184	5596	0% (0%‐0%)	*p* = 0.69

**Reference: **[1] Hill A, Hughes SL, Gotham D, Pozniak AL. Tenofovir alafenamide versus tenofovir disoproxil fumarate: is there a true difference in efficacy and safety? J Virus Erad. 2018;4:72‐9.

## O144

### Dual therapy with PI/r+3TC or PI/r+TDF shows non‐inferior HIV RNA suppression and lower rates of discontinuation for adverse events, versus triple therapy. Meta‐analysis of seven randomised trials in 1624 patients

**Z Liew^1^, A Hill^2^, B Simmons^1^**

^1^Faculty of Medicine, Imperial College London, London, UK. ^2^Department of Translational Medicine, University of Liverpool, Liverpool, UK

**Background:** ****Using fewer nucleos(t)ide analogues could improve safety, increase adherence and lower treatment costs. Generic versions of lamivudine (3TC), tenofovir (TDF), atazanavir/r (ATVr), darunavir/r (DRV/r) and lopinavir/r (LPV/r) are becoming available worldwide. Several randomised trials have evaluated two drug combinations of a ritonavir‐boosted protease inhibitor (PI/r) in combination with 3TC or TDF in naive patients or those with HIV RNA suppression at baseline.

**Methods: **A systematic search of PubMed, Embase, conference proceedings and trial registries was conducted to identify all randomised controlled trials comparing PI/r+3TC or PI/r+TDF dual therapy to triple therapy in treatment‐naïve and treatment‐experienced, suppressed patients. Using inverse‐variance weighting, pooled risk differences (RD) were calculated for virological suppression (FDA Snapshot), protocol‐defined virological failure, treatment‐emergent resistance and discontinuation due to adverse events. Virological suppression was assessed for non‐inferiority (FDA non‐inferiority margin delta=‐4%).

**Results:** ****Seven studies were identified of three different ritonavir‐boosted PIs in 1624 patients, three treatment naïve (ANDES n = 145, GARDEL n = 306, Kalead n = 152) and four treatment experienced (ATLAS n = 236, DUAL n = 249, OLE n = 239, SALT n = 267). The pooled risk difference for viral suppression at 48 weeks of dual therapy compared to triple therapy was +2% (95% CI ‐2% to +6%) which met the FDA criteria for non‐inferiority. Results were consistent in treatment‐naïve and switching studies (*p* = 0.94). There were 5/822 patients on dual therapy with treatment‐emergent primary IAS NRTI drug resistance mutations, versus 5/802 on triple therapy (*p* = 0.98) (Table 1). Treatment discontinuation for adverse events was significantly lower for dual therapy at Week 48 (RD ‐2.6%, 95% CI ‐4.2 to ‐0.9%, *p* = 0.002).

**Conclusions:** ****In this meta‐analysis of seven randomised trials in 1624 patients, rates of HIV RNA suppression <50 copies/mL at Week 48 on PI/r+3TC or PI/r+TDF dual therapy were non‐inferior to triple therapy by US FDA criteria, with significantly fewer discontinuations for adverse events. Consistent results were seen in treatment‐naive and suppressed patients. There was no increased risk of treatment‐emergent drug resistance for dual therapy. Combination treatment with DRV/r+3TC costs <$500 per person‐year in low‐ and middle‐income countries. Generic combinations of DRV/r+3TC could save significant costs relative to branded TDF/FTC or TAF/FTC, with potential improvements in safety.

**Table:** **Abstract O144 –** Table 1. Meta‐analysis of dual therapy trials

Endpoint	Dual therapy	Triple therapy	Difference (95% CI)
HIV RNA <50 copies/mL FDA Snapshot	686/822 (83.5%)	644/802 (80.3%)	+2% (‐2% to +4%)
Protocol‐defined virological failure	41/822 (5.0%)	36/802 (4.5%)	0% (‐2% to +2%)
Treatment‐emergent drug resistance	5/822 (0.6%)	5/802 (0.6%)	0% (‐1% to +1%)

Country of research: United Kingdom. Key population: People living with HIV.

## O145

### No significant changes to residual viremia after switch to dolutegravir and lamivudine in a randomized trial

**J Li^1^, P Sax^1^, V Marconi^2^, J Fajnzylber^1^, B Berzins^3^, A Nyaku^4^, C Fichtenbaum^5^, T Wilkin^6^, C Benson^7^, S Koletar^8^, R Lorenzo‐Redondo^3^, B Taiwo^3^**

^1^Infectious Diseases, Brigham and Women's Hospital, Boston, MA, USA. ^2^Infectious Diseases, Emory University, Atlanta, GA, USA. ^3^Infectious Diseases, Northwestern University, Chicago, IL, USA. ^4^Infectious Diseases, Rutgers University, Newark, NJ, USA. ^5^Internal Medicine, University of Cincinnati, Cincinnati, OH, USA. ^6^Infectious Diseases, Weill Cornell Medicine, New York, NY, USA. ^7^Infectious Diseases and Global Public Health, University of California, San Diego, CA, USA. ^8^Infectious Diseases, The Ohio State University, Columbus, OH, USA

**Background: **The Antiretroviral Strategy to Promote Improvement and Reduce Exposure (ASPIRE) study was a randomized, 48‐week, controlled trial for participants who were virologically suppressed on a standard three‐drug antiretroviral regimen (ART) and maintained viral suppression by commercial viral load testing after switching to dolutegravir and lamivudine (DTG+3TC) [1]. We assessed levels of residual viremia by an ultrasensitive viral load assay to determine whether DTG+3TC resulted in increased low‐level viral replication.

**Materials and methods: **The integrase single‐copy assay (iSCA, limit of detection 0.5 HIV‐1 RNA copies/mL) was performed on plasma from study entry, Week 24 and Week 48 after randomization to DTG+3TC versus continued three‐drug ART. Differences in residual viremia between the treatment arms were analyzed by fitting a linear model accounting for possible within‐patient correlation using a generalized least square fit. We included the study time point and treatment arm in the model. Participants who discontinued ART during the study were excluded from this analysis.

**Results: **Of the 89 participants randomized in the ASPIRE study, seven discontinued their randomized ART due to virologic rebound (N = 2), adverse events (N = 1) or noncompliance (N = 4). A total of 82 participants were included in the current analysis (41 from each arm). The 82 participants were 88% male, 61% white and had a median age of 48 years with a median CD4 count of 677 cells/mm^3^.  Prior ART exposure (median 5.8 years) consisted of integrase inhibitors (40%), non‐nucleoside reverse transcriptase inhibitors (30%) and protease inhibitor‐based regimens (29%) at the time of study entry. At baseline, mean residual viremia in the DTG+3TC versus three‐drug ART arms (4.9 vs. 5.3 copies/mL HIV‐1 RNA copies/mL, Figure 1) did not differ significantly (difference = ‐0.5 copies/mL, 95% CI ‐3.8 to 2.8, *p* = 0.78). After randomization, the differences in residual viremia, between the DTG+3TC versus three‐drug ART arms, adjusting for the baseline values, were not statistically significant (at Week 24: 1.3 copies/mL, 95% CI ‐2.1 to 4.7, *p* = 0.45; and at Week 48: 0.5 copies/mL, 95% CI ‐2.9 to 3.9, *p* = 0.77).

**Abstract O145 – Figure 1**. Levels of HIV viral load (copies/mL) by the ultrasensitive integrase single‐copy assay by treatment arm at baseline, 24 and 48 weeks after ART switch. ART specifies participants who maintained their three‐drug ART regimen. Tukey's box‐and‐whisker plots, box limits: interquartile range (IQR); middle line: median; diamond: mean; vertical lines: adjacent values (1st quartile −1.5 IQR; 3rd quartile +1.5 IQR); dots: outliers.

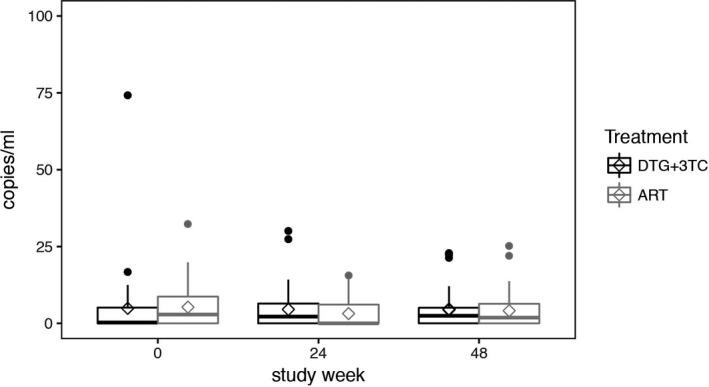

**Conclusions: **In this randomized trial, we found no evidence for increased viral replication after a switch to DTG+3TC as reflected by stable levels of residual viremia. These results support further investigation of DTG+3TC dual therapy.

**Reference: **[1] Taiwo BO, Marconi VC, Berzins B, Moser CB, Nyaku AN, Fichtenbaum CJ, et al. Dolutegravir plus lamivudine maintain HIV‐1 suppression through week 48 in a pilot randomized trial. Clin Infect Dis. 2018;66:1794‐7.

## O21 – Approaches to Treatment and Cure

## O211

### Phase III randomized, controlled clinical trial of bictegravir coformulated with FTC/TAF in a fixed‐dose combination (B/F/TAF) versus dolutegravir (DTG) + F/TAF in treatment‐naïve HIV‐1 positive adults: Week 96

**H Stellbrink^1^, J Arribas^2^, J Stephens^3^, H Albrecht^4^, P Sax^5^, F Maggiolo^6^, C Creticos^7^, C Martorell^8^, X Wei^9^, K White^10^, S Collins^11^, A Cheng^11^, H Martin^11^**

^1^ICH Study Center, Hamburg, Germany. ^2^Infectious Diseases Unit, Hospital La Paz, Madrid, Spain. ^3^Department of Internal Medicine, Mercer University School of Medicine, Macon, GA, USA. ^4^Palmetto Health, University of South Carolina, Columbia, SC, USA. ^5^Division of Infectious Diseases, Brigham and Women's Hospital, Boston, MA, USA. ^6^Infectious Diseases, Azienda Ospedaliera Papa Giovanni XXIII, Bergamo, Italy. ^7^Infectious Diseases, Howard Brown Health Center, Chicago, IL, USA. ^8^Infectious Diseases, The Research Institute, Springfield, MA, USA. ^9^Biometrics, Gilead Sciences, Foster City, CA, USA. ^10^Clinical Virology, Gilead Sciences, Foster City, CA, USA. ^11^HIV Clinical Research, Gilead Sciences, Foster City, CA, USA

**Background: **Bictegravir (B), a novel, potent integrase strand transfer inhibitor with a high barrier to resistance, is coformulated with emtricitabine (F) and tenofovir alafenamide (TAF) as the European Medicine Agency‐approved single‐tablet regimen, B/F/TAF. We report Week (W) 96 secondary endpoint results from an ongoing, double‐blind, Phase III study directly comparing B with dolutegravir (DTG), each given with F/TAF in treatment‐naïve, HIV‐infected adults. Both treatments demonstrated high efficacy with no viral resistance and were well tolerated through W48.

**Methods: **Six hundred and forty‐five treatment‐naive adults living with HIV‐1 and estimated glomerular filtration rate (eGFR) ≥30 mL/min were randomized 1:1 to receive blinded treatment with B/F/TAF (50/200/25 mg) or DTG (50 mg) + F/TAF (200/25 mg) with matching placebos once daily. Chronic hepatitis B and/or C infection was allowed. Primary endpoint was proportion of participants with HIV‐1 RNA <50 copies/mL (c/mL) at W48 (FDA Snapshot); same measure of efficacy was evaluated as a secondary endpoint at W96. Noninferiority for the secondary endpoint was assessed through 95% CIs using a 12% margin. Secondary endpoints were safety measures (adverse events [AEs], laboratory results).

**Results: **At W96, 84.1% (269 of 320) on B/F/TAF and 86.5% (281 of 325) on DTG+F/TAF had HIV‐1 RNA <50 c/mL (difference ‐2.3%; 95% CI ‐7.9% to 3.2%, *p* = 0.41). Number of participants with HIV‐1 RNA ≥50 c/mL at W96 was 0 for B/F/TAF and 5 (1.5%) for DTG+F/TAF. In the per‐protocol analysis, 100% of participants on B/F/TAF had HIV‐1 RNA <50 c/mL versus 98.2% on DTG+F/TAF (*p* = 0.03). Through W96, no participant had emergent resistance to study drugs. AEs led to discontinuation in six (2%) B/F/TAF versus five (2%) DTG+F/TAF (one [B/F/TAF] and four [DTG+F/TAF] after W48). Most common AEs overall were diarrhea (18% B/F/TAF, 16% DTG+F/TAF) and headache (16% B/F/TAF, 15% DTG+F/TAF). Treatment‐related AEs were reported for 20% B/F/TAF versus 28% DTG+F/TAF (*p* = 0.02). Lipid changes were not significantly different between groups. No renal discontinuations and no cases of proximal renal tubulopathy were reported.

**Conclusions: **After 96 weeks, B/F/TAF achieved virologic suppression in 84.1% of treatment‐naïve adults with no treatment‐emergent resistance, and 100% had HIV‐1 RNA <50 c/mL in the per‐protocol analysis. B/F/TAF was safe and well tolerated with fewer treatment‐related AEs compared to DTG+F/TAF.

## O212

### Efficacy and safety of the once‐daily, darunavir/cobicistat/emtricitabine/tenofovir alafenamide (D/C/F/TAF) single‐tablet regimen (STR) in ART‐naïve, HIV‐1‐infected adults: AMBER Week 96 results

**C Orkin^1^, J Eron^2^, J Rockstroh^3^, D Podzamczer^4^, S Esser^5^, L Vandekerckhove^6^, E Van Landuyt^7^, E Lathouwers^7^, V Hufkens^7^, J Jezorwski^7^, M Opsomer^7^**

^1^Barts Health NHS Trust and Queen Mary University of London, London, UK. ^2^School of Medicine, The University of North Carolina, Chapel Hill, NC, USA. ^3^HIV Outpatient Clinic, Universitätsklinikum Bonn, Bonn, Germany. ^4^L'Hospitalet, IDIBELL‐Hospital Universitari de Bellvitge, Barcelona, Spain. ^5^Dermatology und Venerology, University Hospital Essen, Essen, Germany. ^6^Ghent University and Ghent University Hospital, Ghent, Belgium. ^7^Janssen, Pharmaceutica NV, Beerse, Belgium

**Background: **The once‐daily STR D/C/F/TAF 800/150/200/10 mg is approved in the EU and under regulatory review in the US. In AMBER (NCT02431247), D/C/F/TAF was non‐inferior versus D/C+F/TDF (control) (Week 48 VL <50 copies/mL: 91% vs. 88%, respectively; FDA Snapshot), with improved bone and renal biomarker safety, in ART‐naïve, HIV‐1‐infected adults. Week 96 analysis of efficacy, safety and resistance results are presented.

**Methods: **AMBER is a Phase III, randomised, active‐controlled, double‐blind, international, multicentre, non‐inferiority trial. ART‐naïve, HIV‐1‐infected adults were randomised (1:1) to D/C/F/TAF or D/C+F/TDF over at least 48 weeks. After unblinding, patients randomised to D/C/F/TAF continued on open‐label D/C/F/TAF and patients randomised to control were switched to D/C/F/TAF in the extension phase until Week 96.

**Results: **Seven hundred and twenty‐five patients were randomised and treated (at baseline 18% VL ≥100,000 copies/mL; median CD4+ count 453 cells/mm^3^). At Week 96, exposure to D/C/F/TAF was 626 patient‐years in the D/C/F/TAF arm, and consecutively 512 to D/C+F/TDF and 109 to D/C/F/TAF in the control arm. A high proportion of patients in the D/C/F/TAF arm (85%, 308/362) had virological suppression at Week 96 (VL <50 copies/mL; FDA Snapshot). A high Week 96 response rate (from baseline) was also observed in the control arm (84%, 304/363). VL ≥50 copies/mL (VF category by FDA Snapshot) at Week 96 occurred in 20/362 (6%) patients in the D/C/F/TAF arm and 16/363 (4%) from baseline in the control arm. Increases from baseline to Week 96 in CD4+ count (LS means, NC=F) were 229 cells/mm^3^ (D/C/F/TAF) and 227 cells/mm^3^ (control). No darunavir, primary PI or tenofovir RAMs were seen post‐baseline. In one patient in each arm an M184I and/or V RAM was detected (D/C/F/TAF arm Week 36; control arm Week 84). Few serious adverse events (SAEs) and AE‐related discontinuations and no deaths occurred (Table 1). Improvements in renal and bone parameters were maintained in the D/C/F/TAF arm and seen in the control arm after switch, with a small change in TC/HDL‐C ratio (Table 1).

**Table:** **Abstract O212 –** Table 1. Treatment‐emergent AEs and changes in renal, lipid and bone parameters at Week 96

	D/C/F/TAF arm				Control arm		
Treatment‐emergent AEs, n (%)	D/C/F/TAF (baseline — Week 48) N = 362	D/C/F/TAF (Week 48 — Week 96) N = 335	D/C/F/TAF (baseline — Week 96) N = 362	*p*‐value^a^^,^^>b^	D/C+F/TDF (baseline — switch) N = 363	D/C/F/TAF (switch— Week 96)^c^ N = 295	*p*‐value^a,b^
Patient‐years exposure	323	303	626	512	109
AEs, any grade	312 (86)	246 (73)	334 (92)	ND	326 (90)	125 (42)	ND
Grade 3–4 AEs	20 (6)	29 (9)	45 (12)	ND	33 (9)	15 (5)	ND
Serious AEs	17 (5)	24 (7)	39 (11)	ND	36 (10)	8 (3)	ND
AE‐related discontinuations	8 (2)	2 (1)	10 (3)	ND	17 (5)	1 (0.3)	ND
Median change in eGFR							
eGFRcyst, mL/min/1.73 m^2^	+4.0	ND	+4.4	0.007	+1.6	0.0	0.130
eGFRcr, mL/min/1.73 m^2^	‐5.5	ND	‐5.6	0.001	‐8.0	+2.3	0.001
Median changes in renal biomarkers							
UPCR (mg/g)	‐15.7	ND	‐15.5	0.001	‐10.5	‐1.4	0.112
UACR (mg/g)	‐0.6	ND	‐0.7	0.001	‐0.2	‐0.5	0.001
RBP:Cr (µg/g)	+6.9	ND	+13.7	0.001	+35.1	‐35.5	0.001
B2M:Cr (µg/g)	‐30.4	ND	‐27.0	0.001	+18.4	‐40.5	0.001
Median change in fasting lipids							
TC (mg/dL)	+28.6	ND	+34.0	0.001	+10.4	+21.8	0.001
HDL‐C (mg/dL)	+4.4	ND	+5.0	0.001	+1.5	+1.9	0.001
LDL‐C (mg/dL)	+17.4	ND	+21.7	0.001	+5.0	+15.1	0.001
Triglycerides (mg/dL	+24.4	ND	+29.2	0.001	+14.2	+14.2	0.001
TC/HDL‐C ratio	+0.20	ND	+0.25	0.001	+0.08	+0.24	0.001
**Change in BMD**	**N = 113**	**N = 113**	**N = 99**	**N = 83**
Lumbar spine
Mean % change	‐0.7	ND	‐0.9	0.039	‐2.6	+0.5	0.223
Increase by ≥3%	12%	ND	16%	ND	5%	18%	ND
Decrease by ≥3%	27%	ND	34%	ND	43%	10%	ND
Total hip
Mean % change	+0.1	ND	‐0.3	0.473	‐2.8	+0.5	0.160
Increase by ≥3%	12%	ND	17%	ND	2%	18%	ND
Decrease by ≥3%	13%	ND	23%	ND	48%	11%	ND
Femoral neck
Mean % change	‐0.3	ND	‐1.3	0.005	‐3.1	+0.2	0.660
Increase by ≥3%	14%	ND	11%	ND	5%	21%	ND
Decrease by ≥3%	23%	ND	30%	ND	58%	17%	ND

^a^within treatment arm comparisons for change at Week 96 from reference assessed by: Wilcoxon signed‐rank test (eGFR, renal biomarkers and fasting lipids) and paired t‐test (BMD). ^b^reference for the D/C/F/TAF arm is study baseline and for the control arm is the last value before the switch. ^c^respectively 2.5%, 41.3%, 36.4% of patients randomised to the control arm switched to D/C/F/TAF at Week 60, Week 72 and Week 84. eGFRcyst = eGFR based on serum cystatin C (CKD‐EPI formula); eGFRcr = eGFR based on serum creatinine (CKD‐EPI formula); UPCR = urine protein: creatinine ratio; UACR = urine albumin: creatinine ratio; RBP:Cr = urine retinol binding protein: creatinine ratio; B2M:Cr = urine beta‐2‐microglobulin: creatinine ratio; TC = total cholesterol; HDL‐C = high density lipoprotein‐cholesterol; LDL‐C = low density lipoprotein‐cholesterol; BMD = bone mineral density; ND = not determined.

**Conclusions: **High virological response and low failure rates were seen at Week 96 in both arms, with no development of resistance to darunavir or TAF. FoR D/C/F/TAF, safety findings at Week 96 were consistent with those at Week 48. Bone, renal and lipid safety were consistent with known TAF and cobicistat profiles. In the control arm, safety findings were consistent with those in the D/C/F/TAF arm. D/C/F/TAF combines the efficacy and high genetic barrier to resistance of darunavir with the safety benefits of TAF for ART‐naïve, HIV‐1‐infected patients.

## O213

### Comparable viral decay with dolutegravir plus lamivudine versus dolutegravir‐based triple therapy

**J Gillman^1^, P Janulis^2^, R Gulick^3^, C Wallis^4^, B Berzins^2^, R Bedimo^5^, K Smith^6^, M Aboud^6^, B Taiwo^2^**

^1^Prism Health North Texas, Dallas, TX, USA. ^2^Division of Infectious Diseases, Northwestern University, Chicago, IL, USA. ^3^Division of Infectious Diseases, Weill Cornell Medicine, New York, NY, USA. ^4^BARC South Africa and Lancet Laboratories, Johannesburg, South Africa. ^5^Infectious Diseases Section, VA North Texas Health Care System, Dallas, TX, USA. ^6^ViiV Healthcare, Research Triangle Park, NC, USA

**Background: **The GEMINI studies demonstrated non‐inferiority of dolutegravir (DTG) plus lamivudine (3TC) compared to DTG plus tenofovir disoproxil fumarate/emtricitabine (TDF/FTC) in treatment‐naïve HIV‐1 infected individuals with pre‐treatment plasma HIV‐1 RNA (viral load, VL) 1000 to 500,000 copies/mL. However, rapidity of viral suppression with DTG plus 3TC, which may influence the risk of viral transmission or selection of resistant variants, has not been studied adequately, particularly at higher pre‐treatment VL. A substudy of the PADDLE trial showed comparable viral decay between DTG plus 3TC and DTG‐based three‐drug therapy in participants with pre‐treatment VL <100,000 copies/mL.  Viral decay in A5353, a pilot study of DTG plus 3TC where 30% of participants had pre‐treatment VL of 100,000 to 500,000 copies/mL, was determined in comparison to the decay in the DTG (50 mg) plus two nucleos(t)ide reverse transcriptase inhibitors arms of the SPRING‐1 and SINGLE studies.

**Materials and methods: **Change in VL from baseline (pre‐treatment) was calculated for time points shared by A5353 (N = 120), SPRING‐1 (N = 51) and SINGLE (N = 417), i.e. study entry (Week 0), and Weeks 2, 4, 8, 12, 16 and 24. Ninety‐five percent confidence intervals of change from baseline for each observed week, using the log10 transformed VL, were examined and compared across the two‐drug and three‐drug therapy groups using the Wilcoxon Rank Sum test for non‐inferiority (δ = 0.5). For the viral decay analysis, we examined a bi‐exponential non‐linear mixed effect model. Three variables were added as covariates of the initial and secondary decay parameters: two‐drug versus three‐drug therapy, baseline VL (≤ vs. > 100,000 copies/mL) and an interaction term of the drug therapy and baseline VL stratum.

**Results: **The VL change from baseline with two‐drug therapy was non‐inferior to three‐drug therapy (*p* < 0.001). In the decay model (Figure 1), two‐drug therapy was associated with a faster initial decay rate compared to three‐drug therapy. Baseline VL greater than 100,000 copies/mL was associated with a slower initial decay rate. The faster initial decay rate with two‐drug therapy is partially offset when baseline VL is greater than 100,000 copies/mL as indicated by a significant interaction between baseline VL and drug therapy, resulting in simple slope decay rates as shown below. The later decay rate was non‐significantly different from zero, with no significant associations.

**Abstract O213 – Figure 1.** Simple slope decay rates.

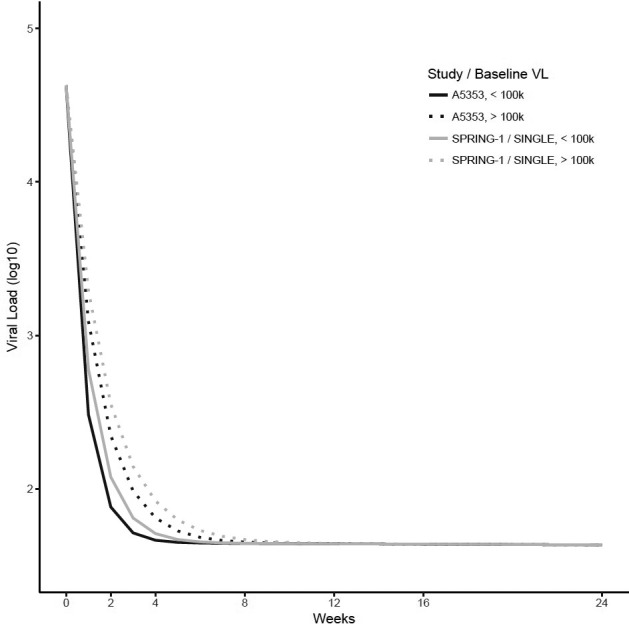

Two‐drug – baseline VL <100,000 copies/mL = 1.272; two‐drug – baseline VL >100,000 copies/mL = 0.725; three‐drug – baseline VL <100,000 copies/mL = 0.969; three‐drug – baseline VL >100,000 copies/mL = 0.596.

**Conclusions: **Viral decay with the two‐drug regimen of DTG plus 3TC is comparable to the viral decay with DTG‐based triple therapy, even in individuals with higher pre‐treatment VL up to 500,000 copies/mL.

## O214

### The impact of M184V/I mutation on the efficacy of abacavir/lamivudine/dolutegravir regimens prescribed in treatment‐experienced patients

**F Olearo^1^, H Nguyen^2^, F Bonnet^3^, G Wandeler^4^, M Stoeckle^5^, V Bättig^5^, M Cavassini^6^, A Scherrer^7^, P Schmid^8^, H Bucher^9^, H Günthard^7^, J Böni^10^, S Yerly^11^, A D'Armino Monforte^12^, M Zazzi^13^, P Bellerive^14^, B Rijnders^15^, P Reiss^16^, F Wit^16^, R Kouyos^17^, A Calmy^18^**

^1^Infectious Diseases Department, University Hospitals of Geneva, Geneva, Switzerland. ^2^Epidemiology, University of Zurich, Zurich, Switzerland. ^3^Infectious Diseases Department, University of Bordeaux, Bordeaux, France. ^4^Infectious Diseases Department, University Hospital of Bern, Bern, Switzerland. ^5^Infectious Diseases Department, University Hospital of Basel, Basel, Switzerland. ^6^Infectious Diseases Department, University Hospital of Lausanne, Lausanne, Switzerland. ^7^Infectious Diseases Department, University Hospital of Zurich, Zurich, Switzerland. ^8^Infectious Diseases Department, University of Bern, Bern, Switzerland. ^9^Epidemiology, Basel University Hospital of Basel, Switzerland. ^10^Virology, Infectious Diseases Department, Zurich, Switzerland. ^11^Virology, University Hospital of Geneva, Geneva, Switzerland. ^12^Infectious Diseases Department, Azienda Ospedaliera‐Polo Universitario San Paolo, Milan, Italy. ^13^Molecular Biology, University of Siena, Siena, Italy. ^14^Virology, University Hospital of Bordeaux, Bordeaux, France. ^15^Infectious Diseases Department, University Hospital Erasmus MC, Rotterdam, Netherlands. ^16^Academic Medical Center, University of Amsterdam, Amsterdam, Netherlands. ^17^Infectious Diseases Department and Epidemiology, University Hospital of Zurich, Zurich, Switzerland. ^18^Infectious Diseases Department, University Hospital of Geneva, Geneva, Switzerland

**Background:** ****The impact of archived resistance mutation M184V/I on virological success remains unclear in treatment‐experienced patients switching to the fixed dose combination abacavir (ABC)/lamivudine (3TC)/dolutegravir (DTG). Considering the possible role of this mutation in impairing the efficacy of both ABC and 3TC, we aimed to determine its impact on the virological failure (VF) rate in patients with suppressed viraemia on cART switching to an ABC/3TC/DTG regimen.

**Materials and methods:** ****This prospective study included treatment‐experienced adults from five European HIV cohorts (ARCA, Aquitaine, ATHENA, ICONA and SHCS) who switched to ABC/3TC/DTG between 2012 and 2016, with ≤50 copies/mL of HIV‐RNA at the time of switch and at least one pre‐existing genotypic resistance test from plasma (when drug‐naïve or during previous treatment failure). The primary outcome was the time to first VF (defined as two consecutive HIV‐RNA measurements >50 copies/mL or one HIV‐RNA measurement >50 copies/mL accompanied by a change in ART). We further considered a composite outcome considering the presence of VF or virological blips, defined as an isolated detectable HIV‐RNA >50 copies/mL followed by a return to virological suppression. A secondary outcome was discontinuation due to adverse events. Multivariate Cox proportional hazard models were used to quantify the effect of the M184V/I on outcomes.

**Results: **One thousand six hundred and twenty‐six patients were included in the analysis (median follow‐up 289 days; IQR 154 to 441). Patients with archived M184V/I (n = 137) were older, more likely to have a history of previous injection drug use and with a longer duration of virological suppression before the switch. The VF rate was 15.1 per 1000 person‐years (95% CI 9.9 to 23.2) and higher in patients harbouring an archived M184V/I mutation, although not statistically significant (29.80 [11.17 to 79.39] vs. 13.56 [8.43 to 21.83]; *p* = 0.093) (Table 1). Figure 1 shows the estimated probability of being free from VF according to the presence of M184V/I mutations. In the multivariable model, M184I/V was not associated with VF (adjustment for VL zenith) or the composite endpoint (HR 3.03, CI 0.84 to 10.82; HR 2.22, CI 0.8 to 5.6, respectively). There were no differences in treatment discontinuation for reasons other than VF between patients with and without documented M184V/I (10.22% vs. 15.58%, respectively; *p* = 0.12).

**Abstract O214 – Figure 1.** Estimated probability of being free from virological failure according to the presence of M184V/I mutations.

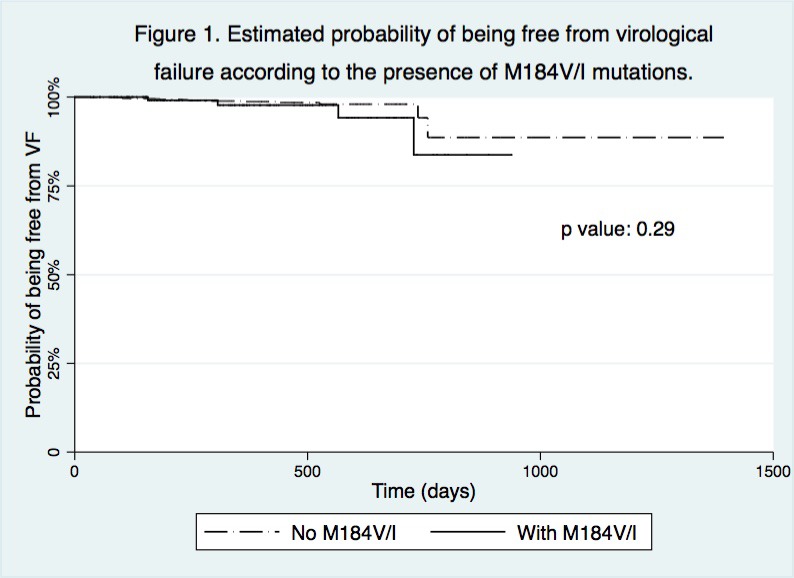

**Table:** **Abstract O214 –** Table 1. Virological primary outcomes

	Without M184V/I, N = 1489 (%)	With M184 V/I, N = 137 (%)
Virological failure (VF)	17 (1.14)	4 (3)
a) 1st definition: 2x HIV‐RNA >50 copies/mL	10 (0.67)	1 (0.73)
b) 2nd definition: 1x HIV‐RNA >50 copies/mL + ABC/3TC/DTG stop	7 (0.47)	3 (2.1)
Treatment (ABC/3TC/DTG) discontinued for reasons other than VF	232 (15.58)	14 (10.22)
Virological blips^a^ (VB) had at least one blip during ABC/3TC/DTG	63 (4.2)	12 (8.8)
VB, median copies/mL (IQR)	79 (62 to 122)	72 (58 to 154)
VF incidence (per 1000 person‐years)	13.56 (8.43 to 21.83)	29.8 (11.17 to 79.39)

^a^virological blips: any viral load measurement >50 copies/mL, with viral load subsequently undetectable.

**Conclusions:** ****The VF rate was very low among treatment‐experienced patients with or without an M184V/I archived mutation who switched to ABC/3TC/DTG and starting this regimen in these patients is safe and well tolerated. However, data over a longer period are needed to confirm the total absence of any impact of M184V/I on the risk of VF.

## O215

### Approaches towards a cure for HIV

**S Fidler**

Department of GUM and HIV Medicine, Imperial College and Imperial College NHS Trust, London, UK

Whilst ART has dramatically improved survival for PLWHIV, maintaining lifelong health through viral suppression requires sustainable global access to ART, lifelong daily adherence to medication, which is untenable for both the individual and implementers. An HIV cure or remission is therefore highly desirable. The accepted definition of an HIV cure is the goal of a significant period of maintained viral suppression off ART (post‐treatment viral control), maintaining zero risk of onward viral transmission as well as individual health. The key barrier to curing HIV is the persistence of virus within a pool of latently infected cells, the so‐called HIV reservoir. These cells appear to persist for the lifetime of the individual and despite years of suppressed viraemia, viral replication re‐emerges, quite rapidly on stopping ART for the vast majority of individuals. The research field of HIV cure has moved rapidly towards new innovations which are currently under trial in addition to ART to explore how to eliminate or significantly reduce the size of the measured HIV reservoir, with a view towards HIV remission. In this presentation I will explore the results of recent studies that are investigating the different approaches to HIV cure. I will present data from some of the UK studies that have recently completed and discuss the next planned studies and approaches with a discussion of what we have learnt along the way.

## O216

### HIV cure and cancer immunotherapy: cross‐disciplinary research at its best

**S Deeks**

Department of Medicine, University of California, San Francisco, CA, USA

Given the challenge of delivering complex, expensive and potentially harmful ART on a global level, there is intense interest in the development of short‐term, well‐tolerated regimens that will either fully eradicate all HIV (a “cure”) or durably prevent HIV replication in the absence of any therapy (a “remission”). Most experts agree that a remission will be easier to achieve than a complete cure. Enthusiasm for this approach is driven in part by recent advances in using novel immunotherapies to reduce and control cancer cells. Cancer and HIV persistence share a number of similarities. In each case, a rare population of cells with the capacity to cause harm becomes established in difficult‐to‐reach tissues. The local environment in cancer and perhaps HIV reshaped to prevent immune mechanisms from clearing the diseased cell. Specifically, a chronic inflammatory environment is often present, resulting in upregulation of a number of pathways which prevent effective immune responses. Therapies that target these immune pathways have either been very successful (in cancer) or now entering the clinic (in HIV disease). Recent observations in HIV‐infected adults with cancer suggest that these approaches are unlikely to cure HIV alone and might have an unacceptable safety profile. Novel approaches to enhance their efficacy and reduce risk are being developed and will be discussed.

## O22 – #Adolescent Lives Matter

## O221

### Living with it: complications of long‐term HIV

**R Ferrand**

London School of Hygiene and Tropical Medicine, London, UK, and Harare, Zimbabwe

The global scale‐up of ART has dramatically increased survival of people with HIV and turned the infection from an invariably fatal disease into a chronic condition. Hence, increasing numbers of children who would previously have died in childhood are now reaching adolescence and adulthood. However, despite ART, HIV‐infected children commonly experience chronic multisystem complications that result in considerable morbidity and increased mortality risk. These include cardiorespiratory, musculoskeletal, neurocognitive and skin disease. These complications are likely a consequence of HIV infection itself, a sequelae of HIV‐associated infections and/or HIV treatment. As coverage of ART increases, it is increasingly apparent that ART alone is insufficient to maintain health and quality of life of children living with HIV. Research to understand the pathogenesis of these complications and to develop therapeutic strategies is needed. Looking ahead, HIV programmes will need to focus not only on delivery of ART but on management of these complications to ensure that children reaching adulthood achieve optimum health outcomes.

## O222

### Taking it: PrEP experiences among adolescent MSM

**S Hosek**

John Stroger Hospital of Cook County, Chicago, IL, USA

While oral HIV pre‐exposure prophylaxis (PrEP) has demonstrated safety and efficacy across populations, uptake and persistent use have been suboptimal among young adult MSM. Furthermore, disparities in race and health care access are emerging that may inhibit the true potential of PrEP as an HIV prevention strategy for young MSM. Prescription rates among adolescent MSM under the age of majority fall even further behind adults, due in part to ethical and regulatory interpretations of consent laws as well as limited knowledge about PrEP and subsequent discomfort of prescribers. This presentation will review the current regulatory landscape for adolescent PrEP access as well as provide data from several recent studies of PrEP knowledge, uptake, adherence and persistence among adolescent and young adult MSM.

## O223

### Staying with it: novel ways to increase adolescent adherence

**C Foster**

Imperial College NHS Trust, London, UK

Is lifelong adherence to ART, or to any medication, potentially for 80+ years, really possible? Why is adherence poorer in adolescents when compared to younger children or older adults? What is the impact of adolescent cognitive development and of mental health on adherence? Are these issues specific to adolescents living with HIV and what can we learn from other chronic diseases? With these questions in mind, how do healthcare professionals, families, peers and the wider community best support adherence for adolescents living with HIV? The existing evidence of the impact of technology, peer mentors, disclosure, education, ART simplification, economic strengthening and cash transfers will be explored, highlighting examples of best practice, yet considerable data gaps persist. The needs of the research community to move from small pilot interventions to well‐powered randomised controlled trials for adolescents struggling with adherence to ART, frequently not a favoured population for research funding, is critical.

## O224

### Dealing with it: mental health and stigma. Report from a study of YPLHIV in the Ukraine

**M Durteste^1^, G Kyselyova^2^, A Volokha^2^, A Judd^3^, C Thorne^1^, R Malyuta^4^, V Martsynovska^5,6^, N Nizova^6^, H Bailey^1^, the Study of Young People Living with HIV in Ukraine**

^1^UCL Great Ormond Street Institute of Child Health, University College London, London, UK. ^2^Shupyk National Medical Academy of Postgraduate Education, Kiev, Ukraine. ^3^MRC Clinical Trials Unit at UCL, Institute of Clinical Trials & Methodology, University College London, London, UK. ^4^Perinatal Prevention of AIDS Initiative, Odessa, Ukraine. ^5^Institute of Epidemiology and Infectious Diseases, The Public Health Center of the Ministry of Health of Ukraine, Kiev, Ukraine. ^6^The Public Health Center of the Ministry of Health of Ukraine, Ukraine.

**Background: **Ukraine has the second largest European HIV epidemic. This study aimed to describe stigma, demographic and social factors and their association with anxiety among young people with perinatally‐acquired HIV (PHIV) or behaviourally‐acquired HIV (BHIV) in Kiev and Odessa.

**Methods: **One hundred and four young people with PHIV and 100 with BHIV aged 13 to 25 years confidentially completed a tablet‐based survey. Tools included the Hospital Anxiety and Depression Scale (HADS) (score of 8 to 10 on anxiety sub‐scale indicating mild and ≥11 indicating moderate/severe symptoms in last seven days), Rosenberg Self‐Esteem Scale (RSES) and HIV Stigma Scale (HSS, short version). Unadjusted Poisson regression models were fitted to explore factors associated with moderate/severe anxiety symptoms.

**Results: **PHIV and BHIV young people had median age 15.5 [IQR 13.9 to 17.1] years and 23.0 [21.0 to 24.3] years respectively and had registered for HIV care a median 12.3 [10.3 to 14.4] years and 0.9 [0.2 to 2.4] years previously; 97% (97/100) and 66% (65/99) respectively were on ART. Overall 30% reported mild and 13% (25/188) moderate/severe anxiety symptoms, with no difference by mode of HIV acquisition (*p* = 0.405) or sex (*p* = 0.700). Forty‐two percent (75/180) reported history of an emotional health problem for which they had not been referred/attended for care, or were unsure regarding care. Higher risk of moderate/severe anxiety symptoms was found with higher HIV‐related stigma (prevalence ratio [PR] 1.24, 95% CI 1.14 to 1.34 per HSS unit increase), lower self‐esteem (PR 0.83, 95% CI 0.78 to 0.90 per RSES point increase), less stable living situation (PR 3.13, 95% CI 1.31 to 7.47 for ≥2 vs. no home moves in last three years), CD4 ≤350 cells/mm^3^ (PR 2.29, 95% CI 1.06 to 4.97), having no‐one at home who knew the respondent's HIV status (PR 9.15, 95% CI 3.40 to 24.66 vs. all know) and, among BHIV, history of drug use (PR 4.64, 95% CI 1.83 to 11.85).

**Conclusions: **Results indicated unmet need for support. Future work is needed to explore strategies for mental health support, particularly around disclosure, self‐esteem and stigma.

## O23 – Mental Health and HIV: What We All Need to Know

## O23

### Mental health and HIV: what we all need to know

**C Orkin^1^, F Lampe^2^, C Izambert^3^, L Waters^4^**

^1^Barts Health NHS Trust and Queen Mary University of London, London, UK; Chair, British HIV Association (BHIVA). ^2^Institute for Global Health, University College London, London, UK. ^3^AIDES, France. ^4^Mortimer Market Centre, University College London Hospitals, London, UK.

People living with HIV experience a higher prevalence of mental health conditions than the general population. Mental health, like HIV, attracts unacceptable levels of stigma; mental ill health negatively impacts quality of life, morbidity & mortality and adherence to medication; neuropsychiatric symptoms, including insomnia, are common and can be associated with HIV medication; medications to treat anxiety, depression and other psychiatric conditions are associated with numerous drug‐drug interactions, requiring prescribers, and patients, to understand how to manage, and mitigate, the risk of related complications. This BHIVA‐led symposium will, with BHIVA and European speakers, describe the epidemiological, stigma and drug interaction challenges related to HIV and mental health and will summarise the management of people reporting insomnia with a view to equipping the community and clinicians to improve mental health care.

## O31 – HIV and Migration: a Renewed Challenge

## O311

### HIV and migration: a renewed challenge

**J del Amo Valero**

National Center for Epidemiology, Institute of Health Carlos III, Madrid, Spain

Migrants are heterogeneous and dynamic populations originating from a variety of countries. In Europe, migrants are exposed to multiple risk‐contexts for HIV infection, which are also related to migration drivers such as poverty and homophobia. This heterogeneity hampers the concept of “migrant” as a single category for analyses. The contribution of migrants to national epidemics varies globally but is the highest in Europe. In the European Union and Economic Area, over half of HIV reports in persons born in a different country to that of residence originate from Sub‐Saharan Africa (SSA), but HIV‐positive persons from Latin America and the Caribbean and from Western, Central and Eastern Europe account for large numbers. These different geographical origins are associated with different epidemiological characteristics, and thus require distinct interventions. The epidemiological patterns largely resemble those of the countries of origin; with a fundamentally heterosexually acquired epidemic in migrants from SSA, a very high proportion of MSM among cases from Latin America and the highest proportion of persons who inject drugs among HIV‐positive European migrants. Time trends are also different; for migrants from SSA sustained declines in new HIV reports have been observed from 2003 onwards whereas steady increases in HIV diagnoses in MSM from Latin America and the Caribbean have been reported. There is solid evidence that HIV acquisition among migrant MSM takes place largely after migration into European cities and accounts for a larger than previously thought proportion among heterosexual migrants from SSA. For most migrant groups, but particularly for undocumented migrants, difficulties to access HIV testing and health care are issues in many European countries and in some, undocumented migrants are not entitled to universal antiretroviral treatment. This talk will address how the dynamism and heterogeneity of migrant populations in Europe demands renewed answers for renewed challenges.

## O32 – Coinfections: TB and Viral Hepatitis

## O321

### HIV‐associated tuberculosis: diagnosis, management and prevention

**G Meintjes**

University of Cape Town, Cape Town, South Africa

Tuberculosis (TB) is the leading cause of death (40%), hospitalisation (18%) and in‐hospital death (25%) among HIV‐infected people globally. The World Health Organization estimates that in 2016, HIV‐associated tuberculosis caused 374,000 deaths. The diagnosis of tuberculosis in patients with advanced HIV can be challenging due to several factors: patients not producing sputum or sputum with low bacillary quantities, and extrapulmonary presentations. Recent advances that have improved diagnostic yield in HIV‐infected people include the Xpert MTB/RIF, Xpert MTB/RIF Ultra and the urine lipo‐arabinomannan (LAM) assay. New versions of the LAM assay promise higher sensitivity, but further field evaluation is required. Co‐treatment of HIV and tuberculosis is complicated by pill burden, drug interactions, shared drug toxicities and the tuberculosis‐associated immune reconstitution inflammatory syndrome (TB‐IRIS). A series of clinical trials published over the last decade have clarified the optimal time to initiate ART in patients being treated for tuberculosis; priority should be given to patients with CD4 <50 cells/mm^3^ in whom starting ART two weeks after TB treatment reduces mortality by 29% compared with waiting to after eight weeks. Pharmacokinetic studies have clarified the impact of TB‐ART drug‐drug interactions and appropriate dose adjustments to deal with these. Efavirenz 600 mg daily can be used with standard tuberculosis treatment. The INSPIRING trial demonstrated that increasing the dolutegravir dose to 50 mg twice daily overcomes the effect of rifampicin induction in patients being treated for tuberculosis. In the Phase I RIFT study, despite the induction of tenofovir alafenamide metabolism by rifampicin, adequate intracellular concentrations of tenofovir diphosphate were maintained with standard dosing. Prednisone is effective at reducing hospital admission and symptom duration when used to treat TB‐IRIS. The PredART trial demonstrated that in high‐risk patients, moderate‐dose prednisone is also effective at preventing TB‐IRIS (30% reduction in incidence) and safe. Tuberculous meningitis is the most severe form of tuberculosis with ˜50% mortality in HIV‐infected people; further investigation of novel treatment strategies is required. The introduction of novel and repurposed drugs (e.g. bedaquiline, linezolid) in the regimens of patients with drug‐resistant tuberculosis has been associated with improved outcomes including in HIV‐infected patients. There are significant drug interactions of protease inhibitors and efavirenz with bedaquiline. Randomised trials have demonstrated that while ART reduces TB incidence in HIV‐infected patients, the addition of isoniazid preventive therapy further reduces TB incidence (37% to 56% reduction) in patients on ART including in patients with higher CD4 counts. Recent trials have demonstrated that shorter preventive regimens of isoniazid plus rifapentine (three or one month) may be equally effective as longer isoniazid regimens with improved tolerability and completion rates. Rifapentine is not currently widely available.

## O322

### Eliminating hepatitis C: a public health perspective

**M Hellard**

Burnet Institute, Melbourne, Australia; and Viral Hepatitis Services, Department of Infectious Diseases, The Alfred Hospital, Melbourne, Australia

Hepatitis C is a leading global health threat affecting hundreds of millions of people annually. More than 71 million people (1.1% of the global population) are chronically infected with hepatitis C, with an estimated 500,000 hepatitis C‐related deaths in 2015. The hepatitis C virus can cause acute and chronic liver infection and is transmitted through blood contact: via unsterile medical and other procedures, sharing of injecting equipment among people who inject drugs and through male‐male sex. People living with hepatitis C suffer worse health and wellbeing, experience considerable social stigma and have lower self‐esteem. Despite the large numbers of people infected with hepatitis C, scientific advances that combine highly effective evidence‐based prevention, and hepatitis C testing and treatment using direct acting antiviral therapy means hepatitis C elimination is possible. The World Health Organization (WHO) has set targets to eliminate hepatitis C as a public health threat by 2030. However, evidence suggests currently only 12 countries are on track to meet the WHO 2030 targets. This paper provides an overview of the global hepatitis C elimination efforts. It will highlight countries that are successfully implementing hepatitis C elimination programmes and on track to meet the WHO targets, and the activities and actions being undertaken contributing to their progress. It will also identify the barriers to our elimination efforts. Hepatitis C elimination is not a public health priority in many countries. Limited data and lack of awareness of the hepatitis C epidemic mean the true national, economic and social impact of hepatitis C is substantially underestimated, including healthcare costs, reduced quality of life, workforce participation and productivity. The paper will discuss ways forward for many countries, including the importance of integrating the hepatitis C response into universal health coverage and the sustainable development goals.

## O323

### Minimum prices of generic hepatitis C direct acting antivirals fall below US$50 per person cured

**A Hill^1^, S Hughes^2^, B Simmons^2^, G Cooke^2^, G Khwairakpam^3^**

^1^Translational Medicine, Liverpool University, Liverpool, UK. ^2^Faculty of Medicine, Imperial College London, London, UK. ^3^HIV/AIDS, Treat Asia, Bangkok, Thailand

**Background: **Worldwide in 2016, there were 1.6 million people cured of HCV using direct acting antivirals (DAAs), but 1.5 million new HCV infections. Elimination of HCV will only be possible by 2030 if at least 5 million people are cured each year. The World Health Organization has pre‐qualified three generic forms of sofosbuvir (SOF) and two formulations of daclatasvir (DCV). Using data on exports of raw materials in 2015 to 2018, we calculated currently feasible generic prices of HCV DAAs, and compared to current national prices.

**Methods: **Data on per‐kilogramme prices of SOF, DCV, ledipasvir (LDV) and velpatasvir (VEL) active pharmaceutical ingredients (API) exported from India were extracted from an online database (www.datamyne.com). All costs were calculated in US dollars ($). To calculate the cost of the amount of API needed for a 12‐week treatment course, we added cost estimates for formulation ($0.01 per tablet), packaging (US$0.35/month) and a profit margin of 26%. National prices were extracted from online price databases.

**Results: **The cost of exported SOF API has fallen from $10,100/kg in January 2015 to $970/kg in January 2018. Costs of API are currently $750/kg for DCV, $2250 for LDV and $9046 for VEL. The predicted minimum production price for SOF/DCV is now $47 per 12‐week treatment course. Five generic formulations of SOF and four generic DCV have shown bioequivalence with originator drugs, by FDA criteria. As shown in Table 1, prices of generic SOF/DCV for public tenders in India are close to predicted minimum costs of production. Prices for sale to individuals in India are higher (Table 1). Prices of SOF/DCV in Egypt, Pakistan and Iran are also under $100 per 12‐week course. By contrast, in high income countries, costs per 12‐week course are approximately $8000 to $20,000, including discounts. Global sales of DAAs worldwide now total over $75 billion since launch in January 2014.

**Table:** **Abstract O323 –** Table 1. Price of HCV DAAs per 12‐week course (US dollars)

HCV DAA	SOF/DCV	SOF/LDV	SOF/VEL
USA (Veteran Affairs)	$67,396	$65,722	$18,023
Norway	$54,505	$65,359	$70,304
UK	$48,045	$51,764	$51,764
Canada	$40,843	$50,365	$45,104
Saudi Arabia	$5973	$66,446	$37,711
Vietnam	$1581	$1800	no data
Indonesia	$1314	no data	no data
Cambodia	$570	$600	no data
Myanmar	$285	$285	no data
India, individuals	$186	$210	$375
Ukraine	$78	$90	no data
India, state tender	$69	$141	$195
Cost price	$47	$61	$127

**Conclusion: **The costs of generic production of HCV DAAs are rapidly decreasing. SOF/DCV combination treatment could be produced for US$47 per patient per 12‐week course. At these low prices, elimination of HCV worldwide could be feasible.

## O324

### Long‐term follow‐up of HIV‐HBV co‐infected patients according to the use of anti‐HBV active drugs

**A Calcagno^1^, C Alessandro^2^, A Saracino^3^, A De Luca^4^, E Colella^5^, A Cingolani^6^, L Sarmati^7^, F Ceccherini‐Silberstein^8^, A Antinori^9^, M Puoti^10^, C Perno^11^, A D'Arminio Monforte^12^, on behalf of Icona Foundation Study Group**

^1^Unit of Infectious Diseases, Department of Medical Sciences, University of Torino, Turin, Italy. ^2^Institute for Global Health, University College London, London, UK. ^3^Unit of Infectious Diseases, University Hospital Policlinico Clinic, Bari, Italy. ^4^University Division of Infectious Diseases, University of Siena, Siena, Italy. ^5^Division of Infectious Diseases, ASST di Monza, Monza, Italy. ^6^Institute of Clinical Infectious Diseases, Policlinic A. Gemelli, Catholic University of the Sacred Heart, Rome, Italy. ^7^Clinical Infectious Diseases, Tor Vergata University, Rome, Italy. ^8^Department of Experimental Medicine and Surgery, Tor Vergata University, Rome, Italy. ^9^HIV/AIDS Unit, INMI L. Spallanzani IRCCS, Rome, Italy. ^10^Division of Infectious Diseases, ASST Grande Ospedale Metropolitano Niguarda, Milan, Italy. ^11^Haematology and Oncohematology Department, University of Milan, Milan, Italy. ^12^ Clinic of Infectious and Tropical Diseases, University of Milan, Milan, Italy.

**Background: **Approximately 10% of HIV‐positive patients are co‐infected with HBV. EACS guidelines suggest life‐long use of tenofovir (TDF/TAF) or entecavir (ENT); caution in NRTI substitutions is advised since drugs with lower genetic barrier (lamivudine/emtricitabine [XTC]) may favour the selection of resistance and the occurrence of hepatic flares. The long‐term outcome of anti‐HBV strategies in HIV‐positive patients is poorly known.

**Methods: **We included HIV‐positive patients from the Icona Foundation Study cohort with 2 positive HBsAg (6 months apart) without end stage liver disease (ESLD). Anti‐HBV strategies were classified as TDF/XTC, TDF monotherapy, XTC monotherapy, ENT or no anti‐HBV treatment. The incidence of ESLD (FIB‐4 score >3.25, liver decompensation, hepato‐carcinoma or liver‐related death) was calculated as number of events per person‐years of follow‐up (PYFU). A Poisson regression framework was used to estimate relative rates (RR [95% CI]) of the described endpoints according to current anti‐HBV treatment. A linear mixed model with random intercept and slope was used to compare ALT changes according to anti‐HBV treatments.

**Results: **We included 624 patients. They were mostly male (501, 80.3%), born in Italy (468, 75%), had acquired HIV through heterosexual contact (248, 38.4%) and showed HCV‐ (122, 18.9%) or HDV‐positivity (98, 22.2%). Median age at ART initiation was 38 years (32 to 45); CD4 and HIV RNA were 373 cells/uL (190 to 591) and 4.58 log10 copies/mL (3.80 to 5.12). XTC‐containing regimens were less used in recent calendar periods (48.5% [2000 to 2003], 9.51% [2012 to 2015]) with the exception of the most recent period (18.64% [2016 to 2018]). Over 2203 PYFU we observed 511 ESLD events in treated patients; they were more frequent in subjects receiving XTC (32.7/100 PYFU), non‐anti‐HBV antiretrovirals (30.4/100 PYFU) and less common in those on TDF/XTC (17.1/100 PYFU) or TDF (10.5/100 PYFU). In the unadjusted model patients on TDF/XTC or TDF had lower risk of ESLD compared to those on XTC (RR 0.54 [0.43 to 0.66] and 0.33 [0.12 to 0.89]); in the fully adjusted model the RR was 0.14 for TDF/XTC [0.07 to 0.28, *p* < 0.001] and 0.13 for TDF [0.03 to 0.63, *p* = 0.011]. ALT change per year was not statistically different in patients on TDF/XTC versus XTC in unadjusted [‐0.36 vs. ‐0.19, *p* = 0.597] and adjusted models [‐0.33 vs. ‐0.15, *p* = 0.902].

**Conclusions: **Anti‐HBV monotherapy with 3TC or FTC is associated with a higher risk of severe hepatic outcomes in HIV/HBV‐positive patients. Additional studies are warranted to verify this observation in randomised comparisons and assess the incidence of HBV‐resistance associated mutations in patients treated with sub‐optimal anti‐HBV regimens.

## O33 – What Women Want

## O331

### PrEP for women in Europe

**N Nwokolo**

Sexual Health and HIV Medicine, Chelsea and Westminster Hospital, London, UK

Women make up over 50% of individuals living with HIV worldwide and up to 60% in Sub‐Saharan Africa. Globally, HIV is the leading cause of morbidity and mortality in women of reproductive age. The use of pre‐exposure prophylaxis (PrEP) has contributed to a significant reduction in new HIV infections in MSM in several large cities, despite the fact that many countries do not have PrEP access programmes. Where PrEP is available, usage in women is low and this poses a significant challenge to HIV prevention efforts in this group. The reasons for the low uptake of PrEP by women are complex, and may include lack of awareness, a focus on “risk assessment” by healthcare workers, which may be stigmatising, lack of belief in the effectiveness of PrEP and concerns about what taking PrEP implies about trust in a partner. Additionally, most PrEP access programmes target women with the same messages as for MSM. However, although factors associated with an increased likelihood of HIV acquisition, such rectal bacterial STIs, syphilis and use of post‐exposure prophylaxis are fairly well defined in MSM, and can be used to identify candidates for PrEP in this group, in women, the situation is less clear. It is possible that a more holistic approach that aims to increase awareness and education and that includes PrEP as one of a number of prevention interventions, rather than being the sole focus, may be more successful in women than the strategies currently in use.

## O332

### Pharmacokinetic studies in women and its impact on clinical practice: case study presentation and lecture

**K Aebi‐Popp**

Department of Infectious Diseases, University of Bern, Bern, Switzerland

Approximately 34 million people worldwide are living with HIV‐1 with one‐half women, mostly of reproductive age. The promotion of effective and safe contraception is critical to avoid unwanted pregnancies and associated adverse outcomes, as well as to avoid HIV vertical transmission worldwide. There are concerns if HIV‐positive women on ART can safely and effectively use all contraceptive options either as short‐acting methods (oral pill and injectables) or long‐acting reversible contraception, such as subdermal implants and copper or hormonal intrauterine devices (IUDs). Oestrogens and progestins are both metabolised by the cytochrome P450 (CYP) enzyme system. Drug‐drug interactions might lead to a reduced effectiveness between hormonal contraception and ART. However, the majority of studies measure hormone levels as a surrogate marker for ovulation instead of unplanned pregnancy as real‐life outcome. Most significant interaction with hormonal contraceptives seems to occur in women using ART containing efavirenz. European AIDS Clinical Society guidelines now state that HIV‐infected mothers who wish to breastfeed should be supported, with ‘increased clinical and virological monitoring’. The two main concerns about transmammary exposure to maternal cART are the development of HIV resistance in infants in case of HIV transmission and toxicities resulting from long‐term low‐dose exposure to antiretroviral drugs. Exclusively breastfed infants receive up to 10% of the weight‐adjusted infant dose of NRTIs and NNRTIs, whereas transfer of protease inhibitors (PIs) to the infant is low. However, genetic differences, such as CYP2B6 polymorphisms in the case of efavirenz, can result in higher infant drug exposure through breastfeeding. Individual patient data indicate active transfer of dolutegravir to the breastfed infant, with ongoing studies exploring this question. This lecture will cover an overview of the pharmacokinetic interactions between ART and hormonal contraceptives, pharmacokinetic data in pregnancy and breastfeeding, and will lead in to a discussion of unanswered priority research questions.

## O333

### HCV treatment and pregnancy

**K Lacombe**

Infectious and Tropical Diseases Department, Saint‐Antoine Hospital (AP‐HP), Paris, France

Pregnancy is a key period in the course of a woman's life and its outcome may be influenced by several factors linked to her viral status regarding HIV and hepatitis viruses. It is debated whether co‐infection with HCV alters the course of pregnancy in HIV‐infected mothers, whereas it is quite well established that the risk of HCV transmission to the newborn is impacted by the level of HCV replication and may be enhanced when HIV is uncontrolled. Where the use of peg‐interferon and ribavirin was formally contra‐indicated in pregnant women due to the therathogenic nature of ribavirin, direct antiviral agents may be prescribed if it is considered that the benefit outweighs the risk. However, the conditions of prescription are not well established to date. In this session, a concrete case will be presented and issues arising from HCV management in pregnant women will be discussed, from the natural course of HCV infection to its treatment during pregnancy.

## O34 – Late Breakers/Hot Topics

## O341

### New ARV drugs and strategies

**A Calmy**

HIV/AIDS Unit, Geneva University Hospitals, Geneva, Switzerland

**Introduction: **Thirty years after the commercialisation of the first ARV drug, more than 20 million individuals worldwide receive combination therapy. While current ARVs are capable of meeting the third “90” of the “90‐90‐90” UNAIDS target (i.e. viral suppression), new approaches are needed to ensure that this capability is realised.

**Discussion: **Durable viral suppression can only be achieved if the patient's safety and quality of life are assessed, reported and ensured. Observational cohort studies are invaluable tools for evaluating such outcomes and identifying new or unexpected safety concerns, but current clinical trials are often limited in their duration and demographic scope. Indeed, new drug formulations are not readily tested in the paediatric population and more work is needed to assess simplified maintenance regimens or dual therapies in both treatment‐naïve and ‐experienced patients. Patient‐centric trials on drug effectiveness are also missing. For instance, ample evidence supports the use of dolutegravir (DTG) as a preferred first‐line regimen in naïve patients, as well as a second‐line therapy and post‐exposure prophylaxis. However, the drug's reputation has been tarnished by teratogenic safety concerns during the preconception period. A patient‐centred approach to provide adequate information and choice concerning contraception and ARV options will be critical to appropriately assess the place of DTG in future ARV regimens.

**Conclusion: **There are strong arguments to challenge the classic sequence of triple‐based first, second and salvage regimens. New drugs and (paediatric‐adapted) formulations are theoretically capable of providing durable viral suppression, but this can only be achieved through long‐term academic‐led research focused on providing quality care to minorities and vulnerable populations, particularly in low‐income settings.

## O342

### Dolutegravir‐ versus an efavirenz 400 mg‐based regimen for the initial treatment of HIV‐infected patients in Cameroon: 48‐week efficacy results of the NAMSAL ANRS 12313 trial

**A Cournil^1^, C Kouanfack^2^, S Eymard‐Duvernay^1^, S Lem^3^, M Mpoudi‐Ngole^4^, P Omgba^5^, S Tchokonte^2^, G Edoul Mbesse^6^, E Mpoudi‐Ngole^6^, A Calmy^7^, E Delaporte^8^**

^1^TransVIHMI, IRD‐INSERM‐Université Montpellier, Montpellier, France. ^2^Hopital Central, Hopital de Jour, Yaounde, Cameroon. ^3^Site ANRS, CMG, Yaounde, Cameroon. ^4^Hopital Militaire, Hopital de Jour, Yaounde, Cameroon. ^5^Hopital Cité Verte, Hopital de Jour, Yaounde, Cameroon. ^6^CREMER, LMI PreVIHMI CREMER IRD, Yaounde, Cameroon. ^7^Geneva University Hospitals, Infectious Diseases, Geneva, Switzerland. ^8^TransVIHMI et CHU Montpellier, IRD‐INSERM‐Université Montpellier, Montpellier, France

**Background: **The updated World Health Organization 2018 guidelines for ARV treatment recommend a dolutegravir (DTG)‐based regimen as the preferred first‐line regimen (with the notable exception of women seeking to become pregnant) and efavirenz (EFV) 400 mg as an alternative option. If the superiority of DTG versus EFV‐600 has been described, there is no head‐to‐head comparison of DTG versus EFV‐400. We assessed the efficacy and safety of DTG and EFV‐400 in Cameroon, a country known for its high HIV‐1 genetic diversity, where an increasing rate of NRTI‐ and NNRTI‐transmitted resistance has been observed.

**Materials and methods: **We conducted a Phase III randomised, open label, multicentre trial in three hospitals in Yaoundé. HIV‐1 infected ARV‐naive adults with HIV‐RNA viral load (VL) >1000 copies/mL were randomised (1:1) to DTG or EFV‐400, both with tenofovir (TDF)/lamivudine (3TC). Randomisation was stratified by screening VL and by site. The primary endpoint was the proportion of patients with VL <50 copies/mL at Week 48 (FDA Snapshot algorithm). The treatment difference adjusted for the baseline VL was calculated and non‐inferiority was tested with a 10% margin. A superiority test was planned if non‐inferiority was demonstrated.

**Results: **Eight hundred and twenty participants were assessed for eligibility. Among these, 616 were randomised and 613 (310, DTG; 303, EFV‐400) received at least one dose of study medication (modified intention‐to‐treat [ITT] population). Baseline characteristics were balanced between arms. Overall, 66% were women. Median age was 37 years (29 to 44), median CD4 counts, 281 (154 to 444), median VL, 5.3 (4.8 to 5.8) log10 copies/mL; 66% of patients had a high VL (>100,000 copies/mL). In the ITT analysis snapshot, the proportion of patients with HIV RNA <50 copies/mL was 74.5% (231/310) in the DTG arm and 69.0% (209/303) in the EFV‐400 arm (difference, 5.5%; 95% CI ‐1.6 to 12.7; *p*‐value for the superiority test, 0.13). For patients with a baseline VL <100,000 copies/mL, the proportion was 91.3% (94/103) and 83.5% (86/100), respectively (difference, 7.8%; 95% CI ‐1.2 to 16.8). For patients with a VL >100,000 copies at baseline, the proportion was 66.2% (137/207) and 61.5% (123/200), respectively (difference, 4.7%; 95% CI ‐4.6 to 14.0). In ITT analysis for VL <200 copies/mL the proportion was 89% (276/310) for the DTG arm and 83.5% (253/303) in the EFA‐400 arm (difference, 5.5%; 95% CI +0.1 to 11.0), *p*‐value superiority test 0.046.

**Conclusion: **The overall viral suppression at Week 48 was 71.8%. DTG‐based regimen was non‐inferior to the EFV‐400‐based, but superiority was not demonstrated when considering a threshold of 50 copies. For patients with a VL >100,000 copies, suboptimal VL suppression at the threshold of 50 copies was observed, but with no statistical differences between the two arms. Particular attention has to be given to patients with persistent low level viraemia, and it is essential to ensure a long‐term follow‐up.

## O343

### Efficacy of MK‐8591 against diverse HIV‐1 subtypes and NRTI‐resistant clinical isolates

**J Grobler, Q Huang, D Hazuda, M Lai**

Infectious Disease Biology, Merck Sharp & Dohme, Kenilworth, NJ, USA

**Background: **MK‐8591 is a novel and potent nucleoside reverse transcriptase translocation inhibitor (NRTTI) currently in Phase II clinical development. Single oral doses of MK‐8591 as low as 0.5 mg have demonstrated HIV‐1 suppression in patients out to seven days. MK‐8591 inhibits HIV‐1 reverse transcriptase (RT) by multiple mechanisms and makes unique interactions with RT. Here, we present a comprehensive evaluation of MK‐8591 antiviral activity against a broad panel of clinical isolates harboring mutations that confer resistance to approved NRTIs as well as 11 different wild‐type HIV‐1 subtypes. 

**Materials and methods: **Antiviral activity of MK‐8591, tenofovir (TFV)‐alafenamide (TAF), zidovudine (AZT) and lamivudine (3TC) was evaluated in human PBMCs with wild‐type HIV‐1. The Monogram PhenoSense assay was employed to evaluate the susceptibility of 50 wild‐type (wt) isolates from 11 HIV‐1 subtypes and 94 NRTI‐resistant clinical isolates to MK‐8591, TFV, AZT and 3TC. Susceptibilities were determined using IC50s of wild‐type HIV in PBMCs and fold‐shifts in potencies compared to wild‐type virus.

**Results: **Against wild‐type HIV‐1 in human PBMCs, MK‐8591 (IC50 = 0.2 nM) was over 10‐fold more potent than TAF, AZT and 3TC (IC50s of 3 nM, 10 nM and 144 nM, respectively). Against the common NRTI resistance mutations M184I and M184V, MK‐8591 exhibited IC50s of 0.8 nM (3.9 fold change [FC] vs. wt) and 1.1 nM (5.0 FC), respectively. The mutations K65R, L74V and Q151M displayed hypersusceptibility to inhibition by MK‐8591. Thymidine analog mutations and 69 ins mutations decreased susceptibility to MK‐8591 by less than 4‐fold and 10‐fold, respectively. The susceptibility of these mutants was further reduced 2‐fold with M184I/V. MK‐8591 IC50s against resistant isolates were lower than the IC50 of TAF versus wild‐type HIV. All 11 different HIV‐1 subtype viruses displayed similar susceptibility to MK‐8591.

**Conclusions: **MK‐8591 is more potent against common NRTI resistant HIV‐1 isolates than any marketed NRTI is against wt HIV‐1. MK‐8591 should provide broad mutant and subtype coverage as a component of an HIV‐1 treatment regimen.

## O344A

### Week 48 safety and efficacy of the HIV‐1 attachment inhibitor prodrug fostemsavir in heavily treatment‐experienced participants (BRIGHTE study)

**J Aberg^1^, J Molina^2^, M Kozal^3^, P Cahn^4^, J Lalezari^5^, M Thompson^6^, R Diaz^7^, A Castagna^8^, G Pialoux^9^, M Gummel^10^, A Pierce^11^, P Ackerman^12^, C Llamoso^12^ and M Lataillade^12^**

^1^Infectious Diseases, Icahn School of Medicine at Mount Sinai, New York, NY, USA. ^2^Infectious Diseases, Hopital Saint‐Louis, APHP and University of Paris Diderot, Paris, France. ^3^Infectious Diseases, Yale University School of Medicine, New Haven, CT, USA. ^4^Infectious Diseases, Fundación Huesped, Buenos Aires, Argentina. ^5^Internal Medicine, Quest Research, New Haven, CT, USA. ^6^Infectious Diseases, AIDS Research Consortium of Atlanta, Atlanta, GA, USA. ^7^Infectious Diseases, Federal University of São Paulo, São Paulo, Brazil. ^8^Infectious Diseases, Ospedale San Raffaele, Milan, Italy. ^9^Infectious Diseases, Hopital Tenon, Paris, France. ^10^Statistics, GlaxoSmithKline, Upper Providence, PA, USA. ^11^Clinical Development, ViiV Healthcare, Research Triangle Park, NC, USA. ^12^Clinical Development, ViiV Healthcare, Branford, CT, USA

**Objectives: **Fostemsavir (FTR) is an investigational first‐in‐class prodrug of the active moiety temsavir, which binds to HIV‐1 gp120 and prevents attachment to CD4 receptor on T cells, and other immune cells, thereby blocking virus infection. BRIGHTE is an ongoing Phase III study evaluating FTR in heavily treatment‐experienced (HTE) patients infected with multi‐drug resistant HIV‐1 who are unable to form a viable ARV regimen.

**Methods: **HTE participants failing their current ARV regimen (confirmed HIV‐1 RNA >400 copies/mL) were assigned to the randomized cohort (RC) or non‐randomized cohort (NonRC), depending on if they had 1 to 2 or zero remaining currently approved ARV classes, respectively. Week 24 results, which included the primary efficacy endpoint (mean change in log10 HIV‐1 RNA from Day 1 at Day 8), were presented previously [1]. Week 48 results are presented here.

**Results: **Week 48 rates of virologic suppression (HIV‐1 RNA <40 copies/mL, mITT ‐ Snapshot analysis) were 54% (146/272) for the RC and 38% (38/99) for the NonRC. The Week 48 rates were maintained from Week 24 (53% and 37%, respectively), despite ongoing attrition. Sixty‐nine percent of RC participants achieved <200 copies/mL at Week 48. CD4+ T‐cell counts continued to increase for both cohorts; mean change from baseline of +139 cells/µL (RC) and +63 cells/µL (NonRC). Overall, 92% (343/371) of participants had ≥1 adverse event (AE); most were Grade 1 to 2. Seven percent of participants discontinued due to an AE. Thirty‐five percent of participants had ≥1 serious AE (SAE); most were related to infections. Compared to the RC, the NonRC experienced higher rates of SAEs (31% RC, 44% NonRC), Grade 3 to 4 AEs (26%, 47%) and deaths (4%, 14%).

**Conclusion: **Fostemsavir, in combination with OBT, maintained virologic suppression from Week 24 to Week 48 in this difficult‐to‐treat population. There was continued immunologic improvement through Week 48 across both cohorts. FTR‐containing regimens remained generally well tolerated, with few discontinuations due to AEs. NonRC participants had more severe AEs, consistent with this group being highly immune compromised and having no approved ARVs to pair with FTR at study entry.

**Reference: **[1] Kozal M, Aberg J, Pialoux G, Cahn P, Thompson M, Molina JM, et al. Phase 3 study of fostemsavir in heavily treatment‐experienced HIV‐1‐infected participants: Day 8 and Week 24 primary efficacy and safety results (BRIGHTE Study, formerly 205888/AI438‐047) [oral PS8/5]. 16th European AIDS Conference; 2017 Oct 25‐27; Milan, Italy.

## O344B

### Phase III study of fostemsavir in heavily treatment‐experienced HIV‐1 infected participants: BRIGHTE Week 48 subgroup analysis in randomised cohort participants

**J Molina^1^, J Aberg^2^, I Cassetti^3^, M Kozal^4^, G Latiff^5^, J Lalezari^6^, G Pialoux^7^, A Streinu‐Cercel^8^, M Gummel^9^, A Pierce^10^, P Ackerman^11^, C Llamoso^11^, M Lataillade^11^**

^1^Infectious Diseases, Hopital Saint‐Louis, APHP and University of Paris Diderot, Paris, France. ^2^Infectious Diseases, Icahn School of Medicine at Mount Sinai, New York, NY, USA. ^3^Infectious Diseases, Helios Salud, Ambulatory AIDS Care Center, Buenos Aires, Argentina. ^4^Infectious Diseases, Yale University School of Medicine, New Haven, CT, USA. ^5^Infectious Diseases, Maxwell Centre, Durban, South Africa. ^6^Internal Medicine, Quest Research, San Francisco, CA, USA. ^7^Infectious Diseases, Hopital Tenon, Paris, France. ^8^Infectious Diseases, Carol Davila University of Medicine and Pharmacy, Bucharest, Romania. ^9^Statistics, GlaxoSmithKline, Upper Providence, PA, USA. ^10^Clinical Development, ViiV Healthcare, Research Triangle Park, NC, USA. ^11^Clinical Development, ViiV Healthcare, Branford, CT, USA

**Background: **The BRIGHTE study evaluates fostemsavir (FTR), an investigational first‐in‐class attachment inhibitor, in HIV‐1 infected heavily treatment‐experienced (HTE) participants with limited treatment options (≤2 ARV classes remaining) who are failing their current ARV therapy. Fostemsavir demonstrated superior efficacy relative to placebo (0.8 log10 copies/mL decrease for FTR vs. 0.2 log10 copies/mL for placebo; *p* < 0.0001) after eight days of functional monotherapy [1]. Fifty‐four percent of participants in the randomised cohort (1 to 2 remaining ARV classes at baseline), receiving FTR plus optimised background therapy (OBT), were virologically suppressed (HIV‐1 RNA <40 copies/mL) at Week (W)48. The mean increase in baseline CD4+ T‐cell count through W48 was 139 cells/µL (+90 cells/µL at W24).

**Methods: **Here we present a W48 pre‐specified subgroup analysis of key efficacy and safety endpoints in participants within the randomised cohort (Table 1).

**Results: **While HTE participants with low baseline CD4+ counts <20 cells/µL treated with a FTR‐based regimen achieved numerically lower rates of virological response at W48 compared to participants with baseline CD4+ counts ≥200 cells/µL, both groups experienced comparable increases in mean CD4+ counts. For participants with baseline CD4+ counts <20 cells/µL, 35% achieved HIV‐1 RNA <40 copies/mL at W48 and had a mean increase from baseline in CD4+ counts of 145 cells/µL. For participants with baseline CD4+ counts ≥200 cells/µL, 68% achieved HIV‐1 RNA <40 copies/mL at W48 and had a mean increase from baseline in CD4+ counts of 150 cells/µL. A lower W48 virological response rate was observed in participants with a baseline VL ≥100,000 copies/mL (35%) versus VL <100,000 copies/mL (61%). Forty‐three percent of participants with baseline CD4+ counts <20 cells/µL had at least one serious adverse event (SAE) versus 26% of participants with baseline CD4+ counts ≥200 cells/µL. All 15 fatal SAEs occurred in participants with baseline CD4+ counts <20 cells/µL.

**Conclusions: **Despite the representative HTE population studied in BRIGHTE being older and more immunocompromised than a generally treatment‐experienced HIV‐1 infected population, treatment with FTR + OBT demonstrated durability of virological response across most key subgroups (including age, race and gender) through W48. Virological response was lower for those with high baseline VL (≥100,000 copies/mL) and low baseline CD4+ count (<20 cells/µL); two well‐recognised determinants of virological response. Treatment with FTR + OBT also provided notable and clinically relevant improvement in CD4+ counts for all subgroups, including participants with very low baseline CD4+ counts (<20 cells/µL). Most SAEs occurred in the subgroup with baseline CD4+ counts <20 cells/µL.

**Table:** **Abstract O344B –** Table 1. Virological and immunological response by subgroup at Week 48 (randomised cohort)

	Week 48 (FTR+OBT)			
	HIV1 RNA (40 c/mL) ‐ Snapshot analysis		CD4+ count (cells/μL)	
	N	n (%)	n	Mean ΔBL (SD)
Total randomised cohort	272	146 (54)	228	138.9 (135.06)
Subgroups
Age (years)				
35	61	31 (51)	52	185.9 (129.17)
35 to 50	101	50 (50)	84	121.1 (136.87)
≥50	110	65 (59)	92	128.5 (131.89)
Gender				
Male	200	102 (51)	168	131.7 (135.83)
Female	72	44 (61)	60	159.0 (131.90)
Race				
White	184	92 (50)	148	141.1 (143.82)
Black, African American	60	39 (65)	54	144.6 (121.40)
Geographic region				
North America	108	60 (56)	93	105.5 (111.16)
Europe	51	27 (53)	38	196.6 (179.53)
South America	105	56 (53)	91	143.6 (127.14)
BL viral load (c/mL)				
1000	31	22 (71)	27	86.7 (193.44)
1000 to 10,000	44	30 (68)	38	134.6 (124.12)
10,000 to 100,000	117	66 (56)	96	133.6 (115.38)
≥100,000	80	28 (35)	67	169.8 (134.40)
BL CD4+ (cells/μL)				
20	72	25 (35)	58	145.2 (109.33)
20 to 50	25	12 (48)	20	149.0 (93.20)
50 to 100	39	22 (56)	30	126.4 (103.62)
100 to 200	63	37 (59)	57	123.1 (101.13)
≥200	73	50 (68)	63	150.0 (195.88)
Fully active ARVs in initial OBT				
ARV=1	137	79 (58)	120	140.7 (131.63)
ARV=2	118	62 (53)	99	139.3 (142.80)

ΔBL = change from baseline; c/mL = copies per millilitre; SD = standard deviation; OBT = optimised background therapy; ARV = antiretroviral; FTR = fostemsavir.

**Reference: **[1] Kozal M, Aberg J, Pialoux G, Cahn P, Thompson M, Molina JM, et al. Phase 3 study of fostemsavir in heavily treatment‐experienced HIV‐1‐infected participants: Day 8 and Week 24 primary efficacy and safety results (BRIGHTE Study, formerly 205888/AI438‐047) [oral PS8/5]. 16th European AIDS Conference; 2017 Oct 25‐27; Milan, Italy.

## O345

### Analysis of patients completing the ibalizumab Phase III trial and expanded access program

**B Emu^1^, S Weinheimer^2^, Z Cohen^3^ and S Lewis^2^**

^1^Infectious Disease, Yale School of Medicine, New Haven, CT, USA. ^2^HIV, TaiMed Biologics, Irvine, CA, USA. ^3^Medical Affairs, Theratechnologies Inc, Montreal, Canada

**Background: **Ibalizumab (IBA) is a CD4‐directed post‐attachment HIV‐1 inhibitor that blocks HIV entry into CD4+ T cells while preserving normal immunologic function. IBA, a long‐acting humanized monoclonal antibody, binds to domain 2 of CD4 receptor, away from major histocompatibility complex II binding sites. TMB‐301 was a 25‐week, Phase III trial conducted in 40 heavily treatment‐experienced patients with multidrug resistant (MDR) HIV‐1. Eligible patients were then rolled over into an expanded access program (EAP) for an additional 24 weeks. We compare the efficacy outcomes of all patients with those who completed the 25‐week study and EAP.

**Materials and methods: **In TMB‐301, patients received a 2000 mg intravenous (IV) loading dose of IBA on Day 7 while continuing their failing regimen. On Day 14, patients initiated an optimized background regimen (OBR) and received 800 mg IV maintenance doses of IBA every 2 weeks through Week 23. Eligible patients who completed the 25‐week trial were rolled over into EAP and continued to receive 800 mg IBA IV every two weeks for an additional 24 weeks. Intent‐to‐treat – missing‐equals‐failure (ITT‐MEF) analysis was the primary method for analysis of efficacy results. In the ITT‐MEF analysis, a missing VL measurement at Week 25/48 was replaced with the baseline VL. In the completer analysis, only VL of patients who completed the Week 25/48 evaluations was considered.

**Results: **In TMB‐301, 40 patients were enrolled and received IBA, of which nine discontinued early. Four of the nine discontinued because of non‐drug‐related deaths and an additional patient due to a drug‐related serious adverse event (AE). The four other discontinuations reported were unrelated to an AE. In the EAP, 27 patients rolled over from TMB‐301, of which three discontinued prior to Week 48 – none related to an AE. At Week 25, median VL reduction was 1.8 log10 with 43% (17/40) of patients achieving full suppression (<50 copies/mL) in the ITT‐MEF analysis. In the completer analysis, median VL decrease was 2.5 log10 with 55% (17/31) of patients reaching <50 copies/mL. At Week 48, in the ITT‐MEF analysis, median VL reduction was 2.8 log10 with 59% (16/27) of patients reaching <50 copies/mL. In the 24 completers, a median VL decrease of 2.9 log10 was evidenced with 16 patients (67%) reaching <50 copies/mL. All patients that reached VL <50 copies/mL remained on treatment, with no virologic failure or breakthrough by Week 48.

**Conclusions: **IBA, in combination with OBR, demonstrates significant antiretroviral activity in patients with MDR HIV‐1 and could represent a long‐term treatment option for these patients.

## O346

### Distribution in cerebrospinal fluid (CSF) of cabotegravir (CAB) and rilpivirine (RPV) after intramuscular administration of long‐acting (LA) injectable suspensions in HIV‐1 infected patients

**S Letendre^1^, A Mills^2^, D Hagins^3^, S Swindells^4^, F Felizarta^5^, J DeVente^6^, C Bettacchi^7^, Y Lou^8^, S Ford^9^, A Cameron^10^, K Sutton^11^, J Shaik^12^, H Crauwels^13^, R D'Amico^14^ and P Patel^14^**

^1^Antiviral Research Center, University of California, San Diego, San Diego, CA, USA. ^2^Southern California Men's Medical Group, Mills Clinical Research, Los Angeles, CA, USA. ^3^Chatham CARE Center, Chatham County Health Department, Savannah, GA, USA. ^4^Division of Infectious Diseases, University of Nebraska Medical Center, Omaha, NE, USA. ^5^Office of Franco Felizarta, Bakersfield Family Medical Center, Bakersfield, CA, USA. ^6^Long Beach Education and Research Consultants, Long Beach, CA, USA. ^7^North Texas Infectious Diseases Consultants, Dallas, TX, USA. ^8^Clinical Statistics, PAREXEL International, Durham, NC, USA. ^9^Clinical Pharmacology Modeling and Simulation, GlaxoSmithKline, Research Triangle Park, NC, USA. ^10^Projects Clinical Platforms and Sciences, GlaxoSmithKline, Research Triangle Park, NC, USA. ^11^Clinical R&D, ViiV Healthcare, Research Triangle Park, NC, USA. ^12^Clinical Pharmacology Modeling and Simulation, GlaxoSmithKline, Collegeville, PA, USA. ^13^Research and Development, Janssen Pharmaceuticals, Beerse, Belgium. ^14^Clinical R&D, ViiV Healthcare, Research Triangle Park, NC, USA

**Background: **CAB and RPV, LA injectable antiretrovirals, are in development as maintenance therapy in virologically suppressed HIV‐1 infected adults. The objectives of this analysis were to evaluate CAB and RPV concentrations in CSF and plasma; determine CSF to plasma concentration ratios; and assess the impact of a dual CAB and RPV LA treatment regimen on CSF and plasma HIV‐1 RNA at steady state in a subset of HIV‐1 infected patients in the Phase IIb LATTE‐2 study.

**Methods: **Eighteen patients receiving CAB LA + RPV LA every 4 (Q4W; n = 3) or 8 weeks (Q8W; n = 15) in the extension phase of LATTE‐2, who had plasma HIV‐1 RNA <50 copies/mL at the sub‐study screening visit, were enrolled in the CSF pharmacokinetic (PK) sub‐study. Most patients were male (15/18; 83%) with a mean age of 38 years and 61% were Caucasian; 28% were Black and 11% were other ethnicities. Sixteen patients had evaluable paired CSF and plasma samples obtained 7 (+/‐3) days after an injection visit for determination of total and unbound plasma CAB concentrations, total RPV plasma concentrations and total CAB and RPV concentrations in CSF. CAB and RPV plasma and CSF concentrations were determined using validated LC/MS methods. HIV‐1 RNA was measured at the lumbar puncture (LP) visit in plasma and CSF using both the Abbott real time assay (LLOD 40 copies/mL) and the bioMONTR Labs SuperLow assay (LLOD 2 copies/mL).

**Results: **Median CAB and RPV total CSF concentrations were 0.30% to 0.34% and 1.07% to 1.32% of their paired total plasma concentrations. CAB and RPV total CSF concentrations and CSF/plasma ratios were similar between the two LA dose regimens (Q4W, Q8W). CAB and RPV total CSF concentrations were higher than their respective in‐vitro IC50 for wild‐type HIV‐1 viruses (CAB, 0.000089 to 0.0003 µg/mL; RPV, 0.066 to 0.081 ng/mL). There were no SAEs or AEs leading to discontinuation. Injection site reactions were mild or moderate with three patients reporting injection site pain. Four patients experienced a headache post‐LP including one Grade 3 headache that resolved in two days. All patients had HIV‐1 RNA <50 copies/mL in plasma and CSF.

**Table:** **Abstract O346 –** Table 1. Pharmacokinetic and antiviral activity results

Pharmacokinetics	CAB (µg/mL) median (min, max) Q8W (n = 15)	CAB (µg/mL) median (min, max) Q4W (n = 3)	RPV (ng/mL) median (min, max) Q8W (n = 15)	RPV (ng/mL) median (min, max) Q4W (n = 3)
Plasma total	3.92 (1.30, 6.41)	3.02 (2.37, 5.10)	192 (91.7, 378)	134 (83.0, 187)
Plasma unbound	0.0047 (0.0007, 0.0220)	0.0019 (0.0014, 0.0698)	NP	NP
Unbound fraction in plasma (%)	0.103 (0.056, 0.912)	0.075 (0.062, 1.45)	NP	NP
CSF total	0.0106 (0.0053, 0.0245)^a^	0.0127 (0.0082, 0.0159)	1.84 (0.00, 2.90)^a^	1.67 (1.40, 2.47)
Ratio CSF:total plasma (%)	0.304 (0.218, 0.449)^a^	0.344 (0.312, 0.421)	1.07 (0.00, 1.52)^a^	1.32 (1.25, 1.69)
**Antiviral activity**	**Abbott real time assay HIV‐1 RNA <50 copies/mL n/N (%) Q8W (n = 15)**	**Abbott real time assay HIV‐1 RNA <50 copies/mL n/N (%) Q4W (n = 3)**	**SuperLow assay HIV‐1 RNA <2 copies/mL n/N (%) Q8W (n = 15)**	**SuperLow assay HIV‐1 RNA <2 copies/mL n/N (%) Q4W (n = 3)**
Plasma HIV‐1 RNA on Day 8	15/15 (100%)	3/3 (100%)	9/15 (60%)	3/3 (100%)
CSF HIV‐1 RNA on Day 8	13/13^a^ (100%)	3/3 (100%)	12/13^a^ (92%)	3/3 (100%)

^a^N = 13, failed to collect CSF for two subjects. NP = not performed.

**Conclusions: **The concentrations of CAB and RPV in the CSF exceeded in‐vitro IC50 values for wild‐type HIV‐1 suggesting that CAB and RPV achieve therapeutic concentrations in the CSF at steady state following LA administration. A dual regimen of CAB LA + RPV LA administered every four or eight weeks achieves effective virologic control in CSF comparable to that observed in plasma.

## Poster Abstracts ARV‐BASED PREVENTION – MOTHER‐TO‐CHILD TRANSMISSION

## P001

### An analysis of congenital anomalies in pregnant women living with HIV in Canada: no signal for neural tube defects in women exposed to dolutegravir

**D Money^1^, T Lee^2^, G Farjou^3^, J Brophy^4^, W Vaudry^5^, I Boucoiran^6^, J Singer^2^, F Kakkar^7^, A Bitnun^8^ and L Sauve^9^**

^1^Obstetrics & Gynecology, University of British Columbia, Vancouver, Canada. ^2^Medicine, CIHR Canadian HIV Clinical Trials Network, Vancouver, Canada. ^3^Medicine, University of British Columbia, Vancouver, Canada. ^4^Paediatrics, Children's Hospital of Eastern Ontario, Ottawa, Canada. ^5^Paediatrics, Stollery Children's Hospital, University of Alberta, Edmonton, Canada. ^6^Obstetrics & Gynecology, CHU Ste‐Justine, Université de Montréal, Montreal, Canada. ^7^Paediatrics, CHU Ste‐Justine, Université de Montréal, Montreal, Canada. ^8^Paediatrics, Hospital for Sick Children, University of Toronto, Toronto, Canada. ^9^Paediatrics, Women's Hospital and Health Centre of British Columbia, University of British Columbia, Vancouver, Canada

**Background: **A recent Botswana surveillance study has shown a 0.9% rate of neural tube defects in the dolutegravir‐exposed pregnancies compared to 0.1% in efavirenz‐exposed pregnancies [1]. Based on these data, cautionary statements on the use of dolutegravir in pregnancy have been issued [1,2]. We interrogated the Canadian Perinatal HIV Surveillance Program (CPHSP) database to determine rates of congenital anomalies in dolutegravir‐exposed pregnancies, as well as overall rates of congenital anomalies among pregnancies exposed to all ARV classes.

**Materials and methods: **The CPHSP is an active surveillance system which has been collecting information on pregnancies of women living with HIV (WLWH); 23 Canadian pediatric and adult HIV centers report data yearly, including maternal characteristics, pregnancy and infant outcomes, and capture the majority of pregnancies in Canada each year [3]. Information on congenital anomalies has been collected from 2007 to 2017 and were classified by system. Descriptive statistics were used to examine frequency congenital anomalies.

**Results: **From 2007 to 2017, there were 2539 infants born to WLWH in the CPHSP, of which 2322 had congenital anomaly data. Overall, of 90 cases of anomalies (3.9%), 10 were associated with chromosomal abnormalities (0.43%), resulting in a non‐chromosomal congenital anomaly rate of 3.5%. Of these 80 cases, three were neural tube defects (overall rate of 0.13%). None were exposed to dolutegravir, but were exposed in the first trimester to zidovudine, lamivudine and nelfinavir; zidovudine, lamivudine, abacavir, atazanavir and ritonavir; and tenofovir, emtricitabine, atazanavir and ritonavir respectively. Of 75 dolutegravir exposures, there were four cases of non‐chromosomal congenital anomalies (5.3%), which were: urinary (n = 2), circulatory (n = 1) and musculoskeletal system (n = 1). Based on exposure to ARVs in the first trimester, the rates were: no ARV exposure in the first trimester ‐ 30/798 = 3.8%; on NRTI/NNRTI ‐ 5/211 = 2.4%; on PI ‐ 43/952 = 4.5%; on integrase inhibitors ‐  10/169 = 5.9%. There was no statistical difference between these rates.

**Conclusions: **This population level surveillance data demonstrates no signal for congenital anomalies amongst pregnancies with dolutegravir exposure in the first trimester with the overall rate of neural tube defects (0.13%) being slightly higher than Canadian population data (0.04%) [4] and comparable to the baseline rates in Botswana in non‐dolutegravir‐exposed pregnancies (0.1%) [1,5]. No other safety signals for congenital anomalies were identified due to other ARV exposure in pregnancy. This data needs to be combined with other global data to further understand any potential risks of ARV exposure in pregnancy.

**References**

[1] World Health Organization. Statement on DTG – Geneva 18 May 2018 [Internet]. 2018 May 18. Available from: http://www.who.int/medicines/publications/drugalerts/Statement_on_DTG_18May_2018final.pdf?ua=1.

[2] European Medicines Agency. New study suggests risk of birth defects in babies born to women on HIV medicine dolutegravir [Internet]. 2018 May 18. Available from: www.ema.europa.eu/ema/index.jsp?curl=pages/news_and_events/news/2018/05/news_detail_002956.jsp{00AMP00}mid=WC0b01ac058004d5c1.

[3] Bitnun A, Lee T, Brophy J, Samson LM, Kakkar F, Vaudry W, et al. for the Canadian Perinatal HIV Surveillance Program. Missed opportunities for prevention of vertical HIV transmission in Canada, 1997‐2016: a surveillance study. CMAJ Open. 2018;6:E202‐E210.

[4] Public Health Agency of Canada. Congenital anomalies in Canada 2013: a perinatal health surveillance report [Internet]. 2013. Available from: http://publications.gc.ca/collections/collection_2014/aspc‐phac/HP35‐40‐2013‐eng.pdf.

[5] Zaganjor I, Sekkarie A, Tsang BL, Williams J, Razzaghi H, Mulinare J, et al. Describing the prevalence of neural tube defects worldwide: a systematic literature review. PLoS One 2016;137:1‐31.

## P002

### Use of integrase inhibitors in HIV‐positive pregnant women: data from the Frankfurt HIV Cohort

**D Weissmann^1^, P De Leuw^1^, P Gute^2^, G Kann^1^, P Khaykin^2^, C Königs^4^, G Schüttfort^1^, C Stephan^1^, A Stücker^5^, T Wolf^1^ and A Haberl^1^**

^1^Infectious Diseases – ZIM II, University Hospital Frankfurt, Frankfurt am Main, Germany. ^2^Infectious Diseases – HIV Therapy, Infektiologikum Frankfurt, Frankfurt am Main, Germany. ^3^Infectious Diseases – HIV Therapy, MainFacharzt, Frankfurt am Main, Germany. ^4^Pediatrics, University Hospital Frankfurt, Frankfurt am Main, Germany. ^5^Gynecology and Obstetrics, University Hospital Frankfurt, Frankfurt am Main, Germany

**Background: **Integrase inhibitors (INSTI) are highly recommended for the treatment of HIV‐positive adults including women of reproductive age. However there are concerns about the use of INSTI in pregnant women. Decreased drug levels of elvitegravir/cobicistat in late pregnancy can potentially result in virological failure and increase the risk of HIV‐mother‐to‐child‐transmission (MTCT). For dolutegravir (DTG) a “red hand” letter has just been sent out in June 2018. Neural tube defects occured in neonates whose mothers had been treated with DTG in early pregnancy. In our cohort study we analyse the maternal and infant outcome of INSTI‐ and non‐INSTI‐containing ART during pregnancy.

**Method: **All women of the Frankfurt HIV Cohort who became pregnant and delivered a child between January 2008 and June 2018 were included in this retrospective study. Primary objective: virological response defined as viral load (VL) <50 copies/mL at the time of delivery. Secondary objectives: rate of HIV‐mother‐to‐child‐transmissions and the rate of malformations in the ART‐exposed children. For all parameters we performed a subanalysis of INSTI‐ and non‐INSTI‐containing regimen.

**Results: **We observed 274 pregnancies resulting in 281 children (five twins + one triplet). INSTI were used during 52 pregnancies (19%), predominantly raltegravir (RAL) (n = 48); DTG (n = 4). Three of the four DTG‐based regimen were switched to RAL during pregnancy (pregnancy week 4, 15 and 18). Table 1 shows characteristics of the INSTI and non‐INSTI groups. The three most common maternal adverse events in the INSTI group were nausea/vomiting (7.7%), elevation of liver enzymes (5.8%) and cystitis (1.9%). In the non‐INSTI group nausea/vomiting (8.1%), cystitis (4.5%) and gestational diabetes (2.7%). The preferred mode of delivery was a caesarean section in 63% of all pregnant women and there was no difference between the INSTI and non‐INSTI arm. In utero ART exposure was 226 days in the non‐INSTI and 155 days in the INSTI arm (*p* = 0.002). The malformation in the INSTI‐exposed child was a laryngomalacia. Malformations in the non‐INSTI group were: defect of the abdominal wall, dextrocardia, epicanthus, syndactile fingers/toes, ventricular septal defect. The vertical transmission was due to non‐adherence.

**Table:** Abstract P002 – Table 1. Characteristics of INSTI and non‐INSTI groups

	INSTI, n = 52	Non‐INSTI, n = 222	All, n = 274
Maternal age (mean), n (years)	31.2	32.1	32.0
African origin, n (%)	53.8	48.6	49.6
CD4 count (mean), n/mL at delivery	551	519	525
Viral load <50 copies/mL at delivery, n (%)	82.7	81.1	81.4
Start of ART >pregnancy week 28, n (%)	11.5	7.2	8.0
Pregnancy week of delivery (mean), n	38.9	38.8	38.8
HIV transmission, n (%)	0	1 (0.5)	1 (0.4)
Congenital malformations, n	1	6	7

**Conclusions: **We observed 274 pregnancies of HIV‐positive women, resulting in 281 children. Fifty‐two (19%) of the pregnant women received INSTI; 48 raltegravir and four dolutegravir. Despite the significant shorter in utero exposure to ART in the INSTI arm there were no differences between the two groups according to virological response, vertical HIV transmission and congenital malformations.

## P003

### Successes and emerging challenges in prevention of vertical HIV transmission in the UK and Ireland

**H Peters, R Sconza, K Francis, A Horn and C Thorne**

University College London, Great Ormond Street Institute of Child Health, London, UK

**Background: **In 2012–2014, the vertical HIV transmission rate (VTR) was 0.27% among diagnosed women living with HIV (WLHIV) in the UK/Ireland. The British HIV Association (BHIVA) currently recommends formula‐feeding infants born to WLHIV, eliminating postnatal transmission risk; however, BHIVA states that virologically suppressed treated women with good adherence to ART who choose/plan to breastfeed may be clinically supported to do so.

**Materials and methods: **The National Study of HIV in Pregnancy and Childhood (NSHPC) conducts comprehensive surveillance of all pregnancies to diagnosed pregnant WLHIV in the UK/Ireland. HIV‐infected children <16 years are reported through a parallel paediatric surveillance scheme. We report maternal characteristics and VTRs among singleton liveborn infants in 2015 to 2016 with infection status reported by 31 March 2018 and reports of planned and/or supported breastfeeding since 2012.

**Results: **There were 1914 singleton livebirths, 71% to Black African women and 83% to women born outside UK/Ireland. Over 99% of pregnancies were in women on ART (70% conceiving on ART). Among 1230 infants with data on maternal viral load (VL) within 30 days of delivery, VL was undetectable (<50 copies/mL) in 93%. Infection status was confirmed for 75% of infants to date, with four transmissions: two infants whose mothers were diagnosed after 20 weeks’ gestation following late antenatal presentation, where transmission occurred in utero (positive PCR aged ≤3 days); one infant born to a woman diagnosed pre‐conception with detectable delivery VL (in utero transmission); and one infant with postnatal transmission via likely breastfeeding (PCR negative at 6 weeks, positive aged 18 months). The overall VTR for 2015 to 2016 was 0.28% [95% CI 0.08% to 0.71%], and 0.17% ([0.01 to 0.92] 1/586) for women diagnosed pre‐pregnancy with undetectable VL throughout pregnancy. There have been 70 reports of planned and/or supported breastfeeding among women on fully suppressive therapy since 2012 (duration: one day to two years/ongoing); 36/70 were infants born 2015 to 2016. Infection status has not been confirmed in many cases and ongoing monitoring is required. Of note, for the likely postnatal transmission described above, breastfeeding was not communicated to clinicians.

**Conclusions: **The VTR among diagnosed WLHIV in the UK/Ireland remains very low at 0.28%; the proportion of women achieving undetectable delivery VL increased since 2012 to 2014 from 87% to 93%. The reports of breastfeeding reflect guideline updates, the current ‘U = U’ era and continued strides towards normalisation of maternity experiences for WLHIV. These require careful monitoring, enabled by our parallel paediatric surveillance scheme, ensuring identification of any late postnatal transmissions and appropriate adjustment of the VTR.

## P004

### Exposure to dolutegravir in pregnant HIV‐positive women in Central and Eastern Europe and neighbouring countries: data from the ECEE Network Group

**J Kowalska^1^, D Gökengin^2^, I Aho^3^, F Yildirim^4^, P Bukovinova^5^, A Papadopoulos^6^ and D Sedlacek^7^**

^1^Hospital for Infectious Diseases, Medical University of Warsaw, Warsaw, Poland. ^2^Ege University, Izmir, Turkey. ^3^Helsinki University Hospital, Helsinki, Finland. ^4^Health Science University Antalya Education and Training Hospital, Antalya, Turkey. ^5^Department of Infectious Diseases and Geographica, University Hospital, Bratislava, Slovakia. ^6^University Hospital ATTIKON, Athens, Greece. ^7^HIV Center, University Hospital, Pilsen, Czech Republic

**Background: **The European Medicines Agency and the World Health Organization recently issued a recommendation to use dolutegravir (DTG) in women with caution due to possible risk of neural tube defect. We aimed to identify the utilisation of DTG in real life among women in Central and Eastern Europe and neighbouring countries (CEEN) where epidemiological data are usually sparse and HIV prevalence is low to moderate level.

**Methods: **We have approached centres from 20 countries participating in the Euroguidelines in Eastern and Central Europe Network Group asking about DTG availability, the scale of its use among women and exposure to the drug in pregnancy [1]. Twelve centres confirmed DTG availability in their country and eight confirmed use of DTG in pregnancy. Six countries (seven centres) provided detailed information on the DTG exposure in pregnancy: Czech Republic, Finland, Greece, Poland, Slovakia, Turkey. Follow‐up was censored at 31 May 2018. 

**Results: **In total 415 women were exposed to DTG and of those 28 were exposed in pregnancy (four started DTG during pregnancy). In terms of conventional risks four women were smoking before pregnancy (two continued in pregnancy), two were drinking alcohol before pregnancy (one continued in pregnancy), three were using psychoactive substances (one continued in pregnancy). The status of TORCH diseases was known in 25 women and for all it was negative. Twenty‐two (78.6%) women were using folic acid during pregnancy. Median number of earlier pregnancies was 2 (IQR 1–3). Twelve women had prior obstetric procedures: seven had abortion (five spontaneous and one due to trisomia) and five had caesarean section in the past. Five (17.8%) women had prior health problems: two had diabetes, one lupus erythematosus, one Rh conflict and one autoimmune hepatitis. Concurrent use of prescribed medication was reported in seven (25%) women (thyroid hormone, azathioprine, methadone, insulin, mirtazapinum). Two (8%) women had detectable HCV RNA before pregnancy. Four pregnancies were ongoing at the time of data collection. Of 24 pregnancies with the outcome there were two (8.3%) abortions, three (12.5%) preterm births and 19 (79.2%) term deliveries.

**Conclusions: **As presented in this work many factors may contribute to pregnancy outcome in HIV‐positive women. Conventional risk factors and concurrent use of prescribed medication should be carefully investigated besides antiretroviral use in pregnancy, in order to properly weigh the risks and benefit of antiretroviral treatment. The number of women exposed to DTG in CEEN countries with low and moderate prevalence is substantial and this work does not include all pregnancies with DTG exposure in the region. Including those countries in drug safety initiatives is vital.

**Reference: **[1] Kowalska JD, Oprea C, de Witt S, Pozniak A, Gökengin D, Youle M, et al. Euroguidelines in Central and Eastern Europe (ECEE) conference and the Warsaw Declaration ‐ a comprehensive meeting report. HIV Med. 2017;18:370‐5. 

## P005

### HCV co‐infection is a strong risk factor for pre‐term birth among HIV‐positive women on cART: data from HIV Out‐Patient Clinic in Warsaw

**K Nowicka, A Wroblewska, M Kalinowska, Z Byczot, E Firląg‐Burkacka, M Marczyńska, J Kowalska**

^1^Department of Children's Infectious Diseases, Medical University of Warsaw, Warsaw, Poland. ^2^HIV Out‐Patient Clinic, Hospital for Infectious Diseases, Warsaw, Poland

**Background: **Both maternal chronic HCV infection and antiretroviral treatment for HIV infection (especially when cART is started before conception) are independent risk factors for intrauterine foetal growth disturbance and preterm delivery [1–4].

**Methods: **The HIV Out‐Patient Clinic of the Hospital for Infectious Diseases in Warsaw provides integrated gynaecological and HIV care since 1994. We reviewed all pregnancies occurring in 2006 to 2017 in the clinic. Multivariate logistic regression models investigated factors associated with preterm birth defined as delivery before 37 weeks of pregnancy.

**Results: **There were 187 pregnancies and within this 17 abortions. Of 159 with known birth outcome and cART status 19 (11.9%; 95% CI 6.9% to 16.9%) were preterm births. Median number of pregnancies were 2 (1 to 3), mother's age at delivery was 31 (IQR 27.5 to 34) years, median gestation week at delivery was 38 (37 to 39) weeks, median last CD4 count before delivery was 525 (371 to 652) cells/μL. In terms of co‐infections 43 (27.0%) women were HCV RNA positive and five (3.4%) HBsAg positive at the time of pregnancy. The most frequent backbone cART was 3TC/FTC + TDF (44.6%), followed by 3TC + AZT (44%), 3TC + ABC (5%) and other (6.3%). In terms of third drug in the regimen 143 (89.9%) women were on PI (91 on LPV/r), 10 (6.3%) on INsTI (all NVP), four (2.5%) on NNRTI (eight on RAL, three on DTG, one on EVG) and two (1.3%) on PI + INsTI based regimen. In univariate logistic regression models factors associated with preterm birth were NRTI backbone, DTG, chronic HCV infection and drug use in pregnancy. After adjustment the only factor associated with increased odds of preterm births was chronic HCV infection (Table 1).

**Table:** Abstract P005 – Table 1. Unadjusted and adjusted regression models for the risk of preterm delivery

	Unadjusted OR (95% CI)	*p*	Adjusted^a^ OR (95% CI)	*p*
ARV started before pregnancy	1.08 (0.41 to 2.82)	0.875	‐	‐
Third drug in cART regimen^b^				
LPV	0.44 (0.15 to 1.30)	0.138	‐	‐
NFV	1.98 (0.50 to 7.79)	0.326	‐	‐
SQV	2.03 (0.74 to 5.60)	0.170	‐	‐
DRV	0.50 (0.06 to 4.00)	0.51	‐	‐
NVP	2.52 (0.25 to 25.5)	0.434	‐	‐
DTG	**16.3 (1.41 to 190)**	**0.026**	21.0 (0.84 to 523)	0.064
NRTI backbone in cART regimen				
ABC+3TC vs. AZT+3TC	**1.61 (0.29 to 8.97)**	**0.228**	0.36 (0.02 to 7.47)	0.728
TDF+FTC/3TC vs. AZT+3TC	**0.29 (0.09 to 0.94)**	**0.083**	0.31 (0.09 to 1.08)	0.347
Other vs. AZT+3TC	**0.54 (0.06 to 4.64)**	**0.741**	0.79 (0.08 to 7.61)	0.691
HCV RNA positive vs. negative	**6.03 (2.19 to 16.6)**	**<0.001**	**4.31 (1.32 to 14.1)**	**0.016**
HBsAg positive vs. negative	2.00 (0.21 to 19.0)	0.546	‐	‐
Mode of HIV infection	‐	‐
IDU vs. heterosexual mode	2.59 (0.93 to 7.26)	0.159	‐	‐
IDU vs. other/unknown	0.99 (0.19 to 5.00)	0.534	‐	‐
Smoking (yes vs. no)	0.54 (0.07 to 1.40)	0.567	‐	‐
Drug use in pregnancy	**3.56 (1.33 to 9.51)**	**0.011**	1.25 (0.33 to 4.71)	0.739
Year of pregnancy by 1	0.94 (0.81 to 1.09)	0.393	‐	‐
Number of pregnancies by 1	0.95 (0.67 to 1.36)	0.797	‐	‐
Last CD4 cell count by 100	0.84 (0.65 to 1.09)	0.192	‐	‐
Mother's age at delivery	1.02 (0.92 to 1.13)	0.742	‐	‐
Detectable VL before delivery	1.02 (0.31 to 3.31)	0.973	‐	‐

^a^ all factors with *p* < 0.1 were included into adjusted model. ^b^RAL, EVG and ATZ were not included due to no preterm birth event.

**Conclusions: **We confirmed that chronic HCV infection is an independent risk factor for preterm delivery among HIV‐positive women. This factor should be considered in future studies of preterm birth risks. Due to limited study sample and confounding by indication the association with DTG should be interpreted with caution. Larger data sets with prospective follow‐up are required to further investigate this relationship.

**References**

[1] Favarato G, Townsend CL, Bailey H, Peters H, Tookey PA, Taylor GP, et al. Protease inhibitors and preterm delivery: another piece in the puzzle. AIDS. 2018;32:243‐52.

[2] Huang QT, Hang LL, Zhong M, Gao YF, Luo ML, Yu YH. Maternal HCV infection is associated with intrauterine fetal growth disturbance: a meta‐analysis of observational studies. Medicine (Baltimore). 2016;95:e4777.

[3] Jhaveri R, Swamy GK. Hepatitis C virus in pregnancy and early childhood: current understanding and knowledge deficits. J Pediatric Infect Dis Soc. 2014;3 Suppl 1:S13‐8.

[4] Uthman OA, Nachega JB, Anderson J, Kanters S, Mills EJ, Renaud F, et al. Timing of initiation of antiretroviral therapy and adverse pregnancy outcomes: a systematic review and meta‐analysis. Lancet HIV. 2017;4:e21‐30.

## P006

### Impact of interventions on the uptake of antiretroviral therapy before pregnancy and prevention of mother‐to‐child transmission of HIV: findings from four scale‐up Local Government Areas of Lagos, Nigeria

**O Akeju^1^, W Agbebaku^2^, T Badru^2^, S Orisayomi^1^, N Ajayi^1^, E James^3^, O Adegbite^2^, B Odusolu^4^, E Oladele^1^ and H Khamofu^1^**

^1^Care and Treatment, FHI 360, Abuja, Nigeria. ^2^Monitoring and Evaluation, FHI 360, Abuja, Nigeria. ^3^HIV/AIDS Care and Treatment, USAID, Abuja, Nigeria. ^4^Program Management, FHI 360, Abuja, Nigeria

**Background: **The Option B+ strategy recommends lifelong ART for all pregnant women with the premise that this will serve to protect the current and future babies from acquiring HIV infection amidst other reasons. Being on ART before a new pregnancy is important for suppressing viral replication thereby preventing mother‐to‐child transmission (PMTCT) of HIV. In 2015, there were 400 new HIV infections among children in Nigeria daily. This abstract describes the impact of interventions on ART uptake before pregnancy and PMTCT.

**Methods: **We conducted a pre‐ and post‐ intervention analysis of uptake of ART before index pregnancy across health facilities in four scale‐up Local Government Areas (LGAs) in Lagos State, Nigeria. Between October 2015 and September 2017, under the USAID‐funded Strengthening the Integrated Delivery of HIV/AIDS (SIDHAS) project, we implemented large‐scale strategic community testing and strengthened provider‐initiated testing and counselling (PITC) at hospitals in these LGAs. In April 2016, implementation of the Option B+ and treat‐all policy was commenced across these LGAs, with the advent of the new national guidelines. We selected 6 months before (April 2015 to September 2015) and after (October 2017 to March 2018) this intervention period for our analysis. Our main outcome indicators were the percentage of women with previously known HIV‐positive status or on ART at antenatal care (ANC) booking and positivity rate for DNA‐PCR among HIV‐exposed infants (HEI). Data were extracted from District Health Information System (DHIS) and descriptive analysis done using Stata.

**Results: **Before the intervention, 4628 pregnant women were tested at first ANC visit compared to 8674 after the intervention. Of the 148 and 175 pregnant women who tested positive to HIV pre‐ and post‐intervention, 24% and 71% knew their HIV‐positive status before ANC booking, respectively. HIV‐positive women on ART prior to ANC booking increased from 20% of total positive (n = 30) pre‐intervention to 66% (n = 115) post‐intervention (*p* < 0.001). DNA‐PCR results for HEI under 2 months showed 2 of 134 was positive pre‐ while none of the 165 results was positive post‐intervention (*p* > 0.1).

**Conclusion: **Our findings show that there was a three‐fold increase in proportion of pregnant women receiving ART prior to first ANC post‐intervention. This was accompanied with a drop in HIV‐positivity rates among HEIs, though not statistically significant. These findings suggest that efforts to increase uptake of antiretrovirals among HIV‐positive women of reproductive age will not only benefit individuals but may also contribute to reducing new paediatric HIV infections.

## P007

### Elimination of pediatric HIV in New York City, 2007 to 2018: missed chances to prevent mother‐to‐child transmission (MTCT) in the era of U = U

**K Beckerman**

Obstetrics & Gynecology, BronxCare Health System, New Rochelle, NY, USA

**Background: **More than 4000 children have been perinatally HIV‐infected in New York City. MTCT peaked in 1990 and fell steady to single digits by 2003. Since then, MTCT reached zero only once, in 2015, and incident pediatric HIV, although rare, persists.

**Material and methods**

We performed a retrospective review of hospital, local and New York State records to identify missed opportunities of transmission prevention among women and children in our region. In our hospital, in addition to universal maternal and newborn HIV antibody screening, all women registering for prenatal care have been asked about their partner's HIV status since 2015.

**Results: **The Bronx continues to have the lowest life expectancy and highest HIV seroprevalence in New York State. While overall HIV incidence is declining, absolute numbers of PLWHIV increase every year. We met USPHS/CDC criteria for elimination of MTCT during only one year, 2015. We identified five missed opportunities of prevention of maternal and/or pediatric HIV infection in our community (Table 1).

**Table:** Abstract P007 – Table 1. Missed opportunities for prevention of HIV infection of women and newborns, Bronx, New York, 2007 to 2018

Year	Maternal Hx	Delivery	Infant	Del VL (copies/mL)	Infant Dx
2007	28yo G3P2002 term labor, outside prenatal care, on cART, stated VL undetectable	NSVD With IV ZDV	ZDV to 6w	40,000	HIV‐1 PCR positive at 1 mo
2009	24yo G2P0 at 37w. s/p 1w AP admission for FUO at 34w, HIVAb neg. Expedited HIV not done.	NSVD No IV ZDV	No ZDV. HIVAbPos neonatal screen	Unknown	HIV‐1 PCR positive at 2 w
2013	31yo G5P2022 a 34w. Complete PNC HIVAb/Ag neg 1^st^ tri & 32w.	NSVD. No IV ZDV	No ZDV. HIVAbPos late NICU screen	Unknown	HIV‐1 PCR positive at 2 w
2017	30yo G6P5004, late to PNC at outside clini. Denied HIV exposure. HIVAb/Ag neg, L&D expedited HIV neg.	NSVD	Normal neonatal screen	Presumed undetectable	P Jiroveci positive ET aspirate at 4 mo
2018	nPEP too late	*Still*	*Pregnant*

In case #1, providers had to make delivery recommendations to their client, who presented in active labor, without access to prenatal and laboratory records. The other four cases involved acute maternal seroconversion during pregnancy and the peripartum period. In case #5, as yet undelivered, maternal seroconversion was documented during the first trimester while she was receiving nPEP. The other three occurred among women who had attended prenatal care and had both early and late negative HIV antibody screens. In retrospect, two occurred very late in pregnancy, and one during breast‐feeding. Of these three, two infected infants were identified by routine neonatal heel‐stick HIV screening. The most recently infected infant, as well as her infected parents, was identified by her endotracheal aspirate at 4 months of age when she was admitted to hospital in respiratory failure.

**Conclusions: **Elimination of MTCT will require new strategies that go beyond universal HIV antibody screening in pregnant women and newborns. When women commonly deliver at hospitals other than where they receive prenatal and/or HIV care, inter‐institutional access to records will be essential. Other strategies must include aggressive maternal/infant antibody and viral testing following recognized HIV exposures (already done in some clinics but not codified in guidelines) as well as expanded, universal, on‐site, routine couples testing as a standard part of prenatal care. Only then will we be able to fully exploit the benefits of PrEP, nPEP and HIV treatment during pregnancy.

## P008

### Efficiency of provider‐initiated HIV testing and counselling in Odessa Regional Hospital, Ukraine

**Y Lopatina^1^, Y Kovalenko^2^, A Chuykov^3^, G Tyapkin^1^, S Esipenko^4^, Y Kvasnevska^1^, A Davies^3^ and A Zakowicz^3^**

^1^AIDS Healthcare Foundation, Ukraine, Kiev, Ukraine. ^2^Odessa Regional Hospital, Odessa, Ukraine. ^3^AIDS Healthcare Foundation, Europe, Amsterdam, Netherlands. ^4^Odessa Regional AIDS Centre, Odessa, Ukraine

**Background: **According to Ukrainian Public Health Center, HIV prevalence in Odessa Region has reached 860,1 per 100,000 population in 2017. Two thousand three hundred and thirty‐four new HIV cases have been identified in Odessa Region in 2017, 74% of them were cases of late HIV diagnosis. According to the international guidelines (including World Health Organization, HIV in Europe), health care providers should recommend HIV testing and counselling to all patients who present with HIV‐related disease.

**Description: **The programme consisted of two phases. In the first phase (2016) we tested for HIV using AHF Rapid Testing Program model (RTP) all patients of Odessa Regional Hospital presenting with conditions listed in “HIV Indicator Conditions” tool by HIV in Europe. Rapid HIV tests and linkage to care support were provided by AHF. The departments in which HIV seropositivity rate was the highest were identified: pulmonology, intensive care and infectious disease unit. In the second phase HIV rapid testing and counselling were offered to patients with HIV indicator diseases in the departments chosen in the first phase.

**Lessons learned**

In 2017 a total of 699 HIV rapid tests were performed in Odessa Regional Hospital with 131 (19%) positive results in comparison to 2016 where total 2988 patients were tested with 94 (3%) positive results. In 2017 highest positivity rate was registered in the pulmonology department, 12 cases among 55 tested (22% positivity rate), in intensive care unit, 27 among 123 (22%) and in infectious diseases unit, 21 among 94 (22%). All cases were not registered previously. For the period 2016 to 2017 total 24 HIV‐positive patients passed away after several days of HIV diagnosis because of critical conditions. Eighty‐three percent of diagnosed were linked to care within 3 months.

**Conclusions: **Provider‐initiated HIV testing for patients admitted to hospitals is an effective strategy to identify people who do not know their HIV status. Pulmonology, intensive care and infectious disease departments were the units with the highest rate of new HIV cases identified in Odessa Regional Hospital. Hospitals need to put special attention to the departments, where case detection is the highest. This intervention will contribute to closing the gap in new case detection in Ukraine.

## P009

### Perinatal HIV‐1C transmitted drug resistance mutations in newly diagnosed antiretroviral‐naïve infants in Botswana

**M Mogwele^1^, K Seatla^1^, S Gaseitsewe^1^, M Leteane^2^ and S Moyo^1^**

^1^Research, Botswana Harvard AIDS Institute Partnership, Gaborone, Botswana. ^2^Biological Sciences, University of Botswana, Gaborone, Botswana

**Background: **Low and middle income countries (LMICS) continue to lag behind in preventing mother‐to‐child transmission (PMTCT) of HIV‐1 to children; Botswana has a transmission prevalence of 1.2%. More often than not, these children end up being infected with a virus harbouring drug‐resistant mutations precluding them from most readily available liquid formulated ART. In Botswana, there is scarcity of data on pre‐treatment drug resistance mutations (PDR) in antiretroviral‐naïve HIV‐1 infected infants. We sought to determine the prevalence of HIV‐1 drug resistance mutations in 27 newly diagnosed HIV‐1 infected infants prior to ART.

**Methods: **Stored HIV‐1C infected infant plasma samples of previously completed studies collected between 2002 and 2015 were analysed. In‐house protocols were used to extract total nucleic acids and a commercial genotyping assay was used to amplify protease and reverse transcriptase fragments. Sanger sequencing was done using big dye technology and drug resistance mutations were analysed using Stanford HIV drug resistance database and the International Antiviral Society‐USA (IAS‐USA) 2017 mutational list.

**Results: **The overall prevalence of HIV‐1 drug resistance mutations in analysed was 18.5% (6/27). Where the predominant mutations were conferring resistance to NNRTI a drug which was used by most HIV‐1 infected mothers in Botswana. The frequencies of mutations analysed were: Y181C n = 1, Y181YC n = 1, M230ML n = 1, K103N n = 2, E138A n = 2, V179D n = 1, Y115F n = 1, L24LF n = 1, M46L n = 1 and I47R n = 1. One infant had multiple drug resistance mutations for NNRTI, protease inhibitor and the mutations were as follows: M46L, Y181C, M230L, M46L, I47I; while another infant had K103N and E138A mutations. Most infants with resistance mutations were resistant to efavirenz, etravirine and nevirapine.

**Conclusion: **There is high prevalence of pre‐treatment drug resistance mutation in infants who become HIV‐1 infected despite the use of and this is a problem especially in consideration that ART formulation for infants is limited in cases of treatment failure. Therefore, transmission of perinatal HIV‐1 drug resistance can be prevented by strengthening the PMTCT framework.

## ARV‐BASED PREVENTION – PREP/TASP

## P010

### Generic tenofovir disoproxil fumarate and emtricitabine tablets obtained from the internet: are they what they say they are?

**X Wang^1^, W Nutland^2^, M Brady^3^, I Green^3^, M McClure^1^ and M Boffito^4^**

^1^Imperial College London, London, UK. ^2^PrEPster, London, UK. ^3^Terrence Higgins Trust, London, UK. ^4^Chelsea & Westminster Hospital, London, UK

**Background: **Pre‐exposure prophylaxis (PrEP) with tenofovir disoproxil fumarate (TDF)/emtricitabine (FTC) has been shown to reduce dramatically the risk of HIV acquisition. However, the National Health Service (NHS) in England has declined to routinely commission PrEP, leading high‐risk individuals to purchase generic versions on‐line. Our team provided therapeutic drug monitoring for tenofovir (TFV) and FTC in 2016 to 2017 for 293 individuals taking generic PrEP and reported that plasma concentrations were all above the target values. More recently concerns have arisen over the authenticity of generic PrEP purchased online. In the present study, we sampled generic PrEP from mainstream brands and suppliers and measured TDF and FTC in the purchased tablet form.

**Methods: **Generic PrEP tablets in sealed bottles of different brands and from different suppliers were obtained from the internet through test purchases. Truvada from Gilead was purchased from Imperial College Healthcare NHS Trust. The brand and supplier of the generic PrEP samples were blinded from the researcher carrying out the analysis. The active pharmaceutical ingredient TDF and FTC was quantified using ultra‐performance liquid chromatography.

**Results: **A total of nine samples were obtained for analysis, including Truvada from Gilead, PrEP used in the IMPACT trial and seven generic PrEP tablets obtained from Mylan, Cipla, Hetero Healthcare and Emcure. The suppliers were Dynamix International, In House Pharmacy, Green Cross Pharmacy and United Pharmacy. As summarised in the table, all the PrEP tablets contained 94.3% to 104.9% of the 300 mg of TDF claimed on the label and 97.3% to 104.4% of the 200 mg FTC claimed on the label.

**Table:** Abstract P010 – Table 1. Analysis of nine samples

						Tenofovir disproxil fumarate	Emtricitabine	
Drug no.	Drug name	Manufacturer	Lot no.	Expiration date	Supplier source	Measured amount in mg	% of label claim (300 mg)	Measured amount in mg	% of label claim (200 mg)
0	Truvada	Gilead	5595307D	Nov‐19	Imperial Healthcare NHS Trust	300.1	100.0	201.2	100.6
1	Emtricitabine/ tenofovir disoproxil	Mylan	1091973	not available	IMPACT trial	299.2	99.7	198.3	99.2
2	RICOVIR‐EM	Mylan	3067417	Apr‐20	Dynamix International	307.4	102.5	201.6	100.8
3	TENVIR‐RM	Cipla	GG80516	Feb‐20	In House Pharmacy	314.8	104.9	208.4	104.2
4	TENOF‐EM	Hetero Healthcare	31171625	Apr‐20	Dynamix International	306.4	102.1	205.2	102.6
5	TENOF‐EM	Hetero Healthcare	31171625	Apr‐20	Green Cross Pharmacy	313.2	104.4	207.2	103.6
6	TENVIR‐EM	Cipla	GG80516	Feb‐20	Dynamix International	312.0	104.0	208.7	104.4
7	TAVIN‐EM	Emcure	E16HX18001	Dec‐20	United Pharmacy	283.0	94.3	194.7	97.3
8	TENVIR‐EM	Cipla	GG80114	Dec‐19	United Pharmacy	307.5	102.5	203.5	101.8

**Conclusions: **All the PrEP tablets sampled in this study contained the claimed amount of TDF and FTC. We were able to confirm the claimed content of the PrEP tablets from various manufacturers and suppliers. Further testing has been planned with additional PrEP tablets being sourced from different manufacturers and suppliers. This study provides reassurance to the community purchasing generic PrEP online and is a good example of a close collaboration between academics, clinicians, HIV charities and PrEP advocates.

## P011

### Geographic barriers result in HIV pre‐exposure prophylaxis discontinuation: how to improve retention in care

**Z Greenwald^1^, K Card^2^, N Niaki, N Lachowsky^2^ and R Thomas^3^**

^1^Epidemiology, Clinique Médicale l'Actuel, Montreal, Canada. ^2^School of Public Health & Social Policy, University of Victoria, Victoria, Canada. ^3^Clinical, Clinique Médicale l'Actuel, Montreal, Canada

**Background: **The potential for pre‐exposure prophylaxis (PrEP) to reduce HIV incidence relies on equitable access to PrEP and retention in care. Despite the availability of low‐cost PrEP through high‐volume clinics in Quebec, these services are centralized in Montreal's downtown gay village – potentially threatening the effectiveness of PrEP for HIV elimination in suburban and rural areas. We aim to investigate barriers to PrEP retention including clinic access.

**Methods: **We examined factors associated with time to PrEP discontinuance using clinical data collected between January 2011 and April 2018 at Canada's largest PrEP clinic (l'Actuel). Cox proportional hazard models estimated adjusted hazard ratios (aHRs) for risk of PrEP discontinuation with censoring of patients maintained in care as of 1 December 2017. Our primary explanatory factor, clinic access, was measured by assessing driving distance from residential postal code centroids to l'Actuel. Other covariates included baseline PrEP regimen, age, income, education and behavioral risk factors (i.e. number of sexual partners within 12 months, antecedent STIs and chemsex use).

**Results: **In total, 1473 clients (median age 36, IQR 29 to 45; 98% MSM) initiated PrEP (82% daily, 18% intermittent) – providing 1460 person‐years of observation. Twelve‐month retention rate was 52%. In April 2018, 662 individuals (45%) were actively maintained in PrEP care. Half (49%) of all PrEP users resided within 5 km of l'Actuel, 28% resided 5 to 9 km away, 10% resided 10 to 19 km away, 7% resided 20 to 49 km away and 6% resided over 50 km away. In multivariate modeling, only greater distance from l'Actuel (aHR 1.002, 95% CI 1.000 to 1.003) and younger age (aHR 0.979, 95% CI 0.970 to 0.989) were associated with increased risk of PrEP discontinuation.

**Discussion: **Few significant findings predict PrEP discontinuance, only greater distance to our clinic and younger age – highlighting the need for additional research regarding patterns of clinical retention among PrEP users. In our setting, young MSM are the highest risk group for HIV acquisition, and greater efforts to initiate and retain young patients on PrEP are essential. Improved public health messages, provider training and alternative PrEP delivery options are needed to expand spatial coverage beyond that of downtown urban cores and to younger clients who may be less likely to live in urban centers or gay neighborhoods.

## P012

### The HIV continuum of care in Austria from 2010 to 2016: data and challenges

**G Leierer^1^, A van Sighem^2^, A Rieger^3^, B Schmied^4^, M Sarcletti^1^, A Öllinger^5^, B Haas^6^, A Egle^7^, M Rappold^1^ and R Zangerle^1^**

^1^Department of Dermatology and Venereology, Medical University of Innsbruck, Innsbruck, Austria. ^2^Stichting HIV Monitoring, Amsterdam, Netherlands. ^3^Department of Dermatology, Medical University of Vienna, Vienna, Austria. ^4^Otto‐Wagner Hospital, Vienna, Austria. ^5^Department of Dermatology and Venereology, Kepler University Hospital, Med Campus III, Linz, Austria. ^6^Department of Internal Medicine, General Hospital Graz Sued‐West, Graz, Austria. ^7^Department of Internal Medicine III, Paracelsus Medical University, Salzburg, Austria

**Background: **UNAIDS has set a 90‐90‐90 target to curb the HIV epidemic by 2020, but methods used are not standardised. HIV surveillance in Austria relies on a hospital‐based cohort not taking into account the transfer of care to private physicians in Vienna, which comprised 24% of all patients with cART in 2016.

**Methods: **Data from the Austrian HIV Cohort Study were used to derive the four‐stage continuum of HIV care. PLWHIV estimates were obtained using back‐calculation models (ECDC tool 1.3.0) to estimate HIV incidence and the undiagnosed fraction. The proportion ever diagnosed who ever initiated ART and the proportion of them who were virally suppressed (≤200 copies/mL) were assessed for all patients and for MSM for the years 2010 to 2016. For high estimates patients lost to follow‐up (LTFU, no contact 1.5 years before the end of the respective year) were excluded and for low estimates they were included. The preferred estimate was the mid‐point between the high and low estimate. Missing HIV‐RNA was considered as unsuppressed. Logistic regression was used to identify factors associated with LTFU.

**Results: **The fraction undiagnosed decreased from 19% (95% CI 18% to 21%) in 2010 to 10% (95% CI 9% to 13%) in 2016, among MSM from 20% (95% CI 18% to 22%) in 2010 to 8% (95% CI 6% to 11%) in 2016. The proportion of diagnosed patients who have ever started ART increased from 81% (79% among MSM) in 2010 to 93% (93% among MSM) in 2016. The proportion of individuals virally suppressed improved from 77% to 84% (76% to 85% among MSM). The fraction of the virally suppressed among PLWHIV increased from 51% to 70%, among MSM from 48% to 73%. Estimates of the number of new HIV infections decreased from 258 (95% CI 237 to 282) to 171 (95% CI 107 to 262), among MSM from 157 (95% CI 143 to 168) to 70 (95% CI 39 to 114). The overall rate of LTFU was 37.8%, multivariable logistic regression revealed three significant factors for LTFU: younger age, residency in Vienna and non‐Austrian origin.

**Conclusions: **Austria is nearing the 90‐90‐90 target. Viral suppression was comparatively low and maybe explained substantially by transfer of care in Vienna and out‐migration. This and the decrease in HIV incidence supports the hypothesis that the high estimate of being on ART and virally suppressed is the more likely scenario. For more reliable nationwide estimates there is urgent need to include data from private physicians.

## P013

### Behavioural, psychological and network characteristics of MSM eligible for PrEP enrolled by respondent‐driven sampling network strategy

**M Psichogiou^1^, M Papadopoulou^1^, S Chanos^2^, V Sypsa^3^, S Roussos^3^, D Paraskevis^3^, N Dedes^2^, G Daikos^1^, J Schneider^4^ and A Hatzakis^3^**

^1^1st Internal Medicine Department, Laiko General Hospital, National and Kapodistrian University of Athens, Athens, Greece. ^2^Positive Voice, Athens Check Point, Athens, Greece. ^3^Department of Hygiene, Epidemiology and Medical Statistics, Medical School, National and Kapodistrian University of Athens, Athens, Greece. ^4^Public Health, University of Chicago, Chicago, IL, USA

**Background: **The benefits of pre‐exposure prophylaxis (PrEP) in HIV prevention are well established. Elimination of HIV transmission could be achieved through a combination of preventive strategies taking into consideration specific population characteristics and the potential for high population coverage. We investigated demographic, behavioural, psychosocial and network characteristics of MSM at ongoing high risk for acquiring HIV infection who were willing to take PrEP.

**Materials and methods: **Sophocles‐P4G, a pilot PrEP study in Athens, Greece, was designed to identify, within MSM networks, the population at highest risk for HIV, based on specified criteria, who were eligible to be treated with PrEP. It was based on recruitment through respondent‐driven sampling (RDS) and facilitated by a community organisation (Positive Voice). The programme included rapid HIV testing and interview with a structured questionnaire.

**Results: **Between 2016 and 2018, of the 308 MSM enrolled, 21 were already known to be HIV+ seeds, five were newly HIV diagnosed and 282 were confirmed to be HIV negative. The 282 MSM at risk had a mean (SD) age of 28.3 (8.2) years, a mean (SD) of 15.7 (2.5) years of education; 54 (19.4%) were unemployed; 40 (14.2%) identified as bisexual; 211 (74.82%) reported having tested for HIV during the previous year; 71 (25.36%) had a sexually transmitted infection (STI) during the previous year. The median (25th, 75th) size in their sexual network was 10 (3, 30) people; 165 (58.5%) reported using drugs associated with chemsex and 84 (31.2%) reported symptoms of depression. PrEP enrolment criteria were reported by 129/282 (41.9%) MSM. Specifically, compared to the low‐risk MSM, they reported more frequent condomless sex (66.3% vs. 25%; *p* = 0.005), higher participation in group sex (90.7% vs. 41.2%; *p* < 0.001), higher median MSM network size (25th, 75th) (30 (10, 50) vs. 3 (1, 9), *p* < 0.001), greater use of drugs associated with chemsex (81.4% vs. 39.2%; *p* < 0.001), while no difference was observed in symptoms of depression. This high‐risk subgroup was older (mean age [SD] 32.5 [9.2] vs. 25.8 [5.7] years; *p* < 0.001) and had a higher incidence of STIs over the previous year (46.1% vs. 7.9%; *p* < 0.001). One hundred and seven out of 129 of them initiated PrEP (82.9%).

**Conclusion: **RDS recruitment of MSM eligible for PrEP is a promising enrolment strategy to increase PrEP participation and population coverage. The large number of MSM reporting chemsex and high risk sexual behaviours within large networks along with the large number of STIs highlight the population who could benefit from PrEP.

## P014

### Modelling of PrEP implementation among PWID in the Russian Federation and ARV cost savings

**G Kaminskiy, V Testov, N Levina, A Samoilova and I Vasilyeva**

National Medical Research Centre of Phthysiopulmonology and Infectious Diseases, Ministry of Public Health of Russian Federation, Moscow, Russian Federation

**Background: **Transmission computational models allow to foresee the results of aggressive intervention aimed on incidence reduction. We used Kermack‐McKendrick model without immunity with parameters characterising people who inject drugs (PWID) as a key risk population for HIV infection in the Russian Federation.

**Methods: **The equations: dY/dt = RαXY‐µY; dX/dt = −RαXY + µ‐µX; where Y ‐ prevalence of HIV infected, X ‐ ratio of susceptible (noninfected). Parameters for the PWID risk group in Russian Federation: Contact rate: (basic reproduction number) R = 3.81; Renewal rate: µ = 1/(3*365); Reverse duration of infectivity: α = 1/(2.5*365); Population number: N = 500,000. With these parameters the model predicts high and stable incidence rate: 359 new cases per day (131,000 new cases per year). The biggest part of HIV epidemic in PWID is hidden. Revealing rate which is a rate of detection + linking + retaining in care is a controlling parameter.

**Results: **Aggressive intervention programme in Russian Federation is aimed to reveal and arrest the hidden HIV epidemic. It includes NGO‐assisted HIV testing campaign with noninvasive rapid tests, on spot HIV diagnosis and immediate treatment. Number of found and managed cases per day must be at least 150 for the risk population size of 500,000 with the revealing rate 0.0003 per day. This stable rate of intervention leads to zero incidence in 55,989 days or 16.4 years. With aggressive intervention campaign HIV‐positive people from the risk group receive immediate treatment, and HIV‐negative should receive PrEP [1]. We considered PrEP rate to be twice higher than revealing rate, that means that at least two people in the surroundings of one infected will receive PrEP. Prescribing PrEP with the rate of 300 per day lessens the time to zero HIV incidence to 1845 days or 5.1 years. PrEP potentially can shorten the time of HIV spread elimination by 10 years (Figure 1). We compared ARV drug costs for both programmes (with and without PrEP) in the middle point of implementing programmes when one half of the necessary number of HIV‐positive patients were already revealed and treated. The annual cost of treatment of each programme was: with PrEP, 14.9 billion RUB (€212.0 million); without PrEP, 27.0 billion RUB (€382.3 million) (Table 1). The middle‐point ratio of programmes’ costs is 1.8 times, and cost difference increases with each subsequent day.

Abstract P014 – Figure 1. HIV incidence curves during the intervention programme in the presense and absence of PrEP.

**Table:** Abstract P014 – Table 1. ARV costs for the intervention programme in the presence and absence of PrEP

	Cost for person/year	Cost for person/year	Programme PrEP + for one year	Programme PrEP + for one year	Programme PrEP + for one year	Programme PrEP ‐ for one year	Programme PrEP ‐ for one year	Programme PrEP ‐ for one year
	Rubles	Euro	People	Billion Rubles	Million Euro	People	Billion Rubles	Million Euro
ART	60,000.0	851.1	138,375	8.3	117.8	449,175	27.0	382.3
PrEP	12,000.0	170.2	553,500	6.6	94.2
Total	14.9	212.0	27.0	382.3

**Abstract P014 – Figure 1.** HIV incidence curves during the intervention programme in the presense and absence of PrEP.

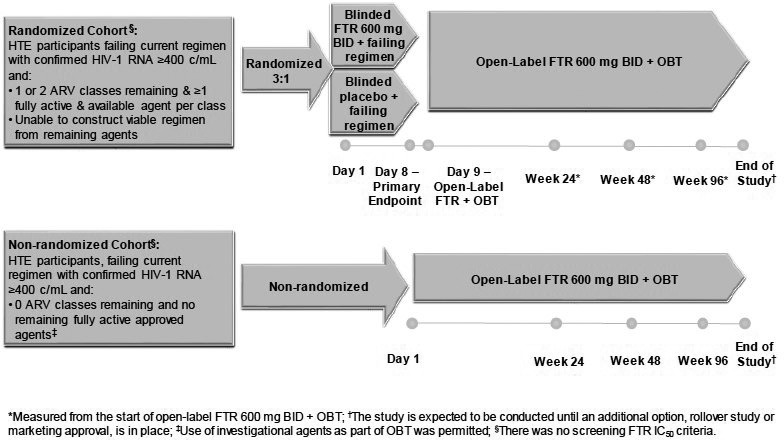

**Conclusions: **PrEP enhances intervention and is cost effective because it lessens time of the intervention programme and saves money on treatment of prevented cases at least twice.

**Reference: **[1] Cairns G, McCormack S, Molina J‐M. The European preexposure prophylaxis revolution. Curr Opin HIV AIDS. 2016;11:74‐9.

## P015

### “The PrEP You Want”: a web‐based survey of online shopping and border crossing for HIV prophylaxis medications

**B Walmsley^1^, D Gallant^2^, M Naccarato^3^, M Hull^4^, A Smith^5^ and D Tan^1^**

^1^Medicine (Infectious Diseases), St.Michael's Hospital, Toronto, Canada. ^2^Knowledge Translation, Gay Men's Sexual Health Alliance, Toronto, Canada. ^3^Clinical Pharmacy, St.Michael's Hospital, Toronto, Canada. ^4^Medicine (Infectious Diseases), University of British Columbia, Vancouver, Canada. ^5^Medicine (Infectious Diseases), Alberta Health Services, Edmonton, Canada

**Background: **In response to the high cost of HIV pre‐exposure prophylaxis (PrEP) medications in Canada, community organizations have created online guides detailing how patients can legally order generic medications online and travel to collect them in the US. Since little is known about the patients following these guides, we conducted a survey of gay, bisexual and other men who have sex with men (gbMSM) living in Ontario to determine their interest in/experience with this approach. 

**Materials and methods: **Between 20 July 2017 and 18 May 2018 we administered two online surveys of gbMSM accessing an online border‐crossing guide posted by a gay men's health organization in Ontario. Participants completed an open baseline survey posted on the border‐crossing guide webpage, and a follow‐up survey 3 months later. The questionnaires covered demographics, HIV risk behaviors, healthcare access, PrEP knowledge, PrEP experience and interest in/experience with the border‐crossing approach. Data were analyzed using descriptive statistics.

**Results: **Most of the 141 participants were young (median age 23; IQR 22 to 25), Black (82%) gbMSM who had completed a college or undergraduate degree (62%). Twenty percent reported a total family income <30,000 CAD, another 54% reported <60,000 CAD and 55% paid for medications entirely out of pocket. More than half of participants reported having heard about the border‐crossing approach from a friend (55%) and most indicated that they were likely to complete the approach (80% at baseline, 79% at follow‐up). At baseline and follow‐up respectively for the 110 participants who completed both surveys, 65% and 80% had discussed PrEP with a healthcare provider, 18% and 25% had obtained a PrEP prescription, 8% and 5% had ordered medications to a US mailbox, while 1% and 0% had crossed the border to collect them. Reported barriers included concerns about the approach's legality (56%), the security of personal health information (39%) and the safety of online vendors (38%).

**Conclusions: **Despite high interest in pursuing an online border‐crossing approach to get PrEP medications, such an approach may not be a viable option for interested gbMSM due to logistical challenges and perceptions of safety and legitimacy. Effective PrEP implementation will require alternative strategies such as public drug coverage to ensure readily accessible PrEP medications for gbMSM living in Ontario.

## P016

### High‐risk MSM are switching from PEP to PrEP

**G Mahir^1^, O El‐Koubani^1^, E Devitt^2^, K Gedela^2^, S McCormack^2^, A McOwan^2^, N Nwokolo^2^, S Patel^2^, T Suchak^2^ and G Whitlock^2^**

^1^Medical School, Imperial College, London, UK. ^2^HIV, 56 Dean Street, London, UK

**Background: **Previous use of PEP is associated with a high risk of subsequent HIV seroconversion at our service and therefore PEP users are offered web‐based interventions including education programmes promoting risk reduction including PrEP. We determine the HIV incidence and use of repeat PEP and PrEP in a cohort of MSM who were prescribed PEP at our service in 2013.

**Materials and methods: **A case‐note review of MSM who received PEP from 1 January 2013 to 30 June 2013 at a central London sexual health service. Case‐notes were reviewed from first PEP to 1 November 2017 for most recent HIV test, repeat PEP use and PrEP initiation/use.

**Results: **Of 530 MSM attending for PEP in the study period, 69 subsequently became HIV positive (13%; incidence rate: 5.4 per 100 person‐years follow‐up [100PYFU]). Of 461 remaining HIV‐negative MSM, 184 (40%) MSM re‐attended our service in the 12 months prior to 1 November 2017. Of these, 52/184 (28%) disclosed PrEP use; 48/184 (26%) received repeat PEP from our service in the previous 12 months. Of the 52 PrEP users, 12 (23%) commenced PrEP before 2016, 15 (29%) during 2016 and 25 (48%) in 2017. For the whole cohort, HIV incidence dropped from 6.1 per 100PYFU before January 2016 to 3.2 per 100PYFU after.

**Conclusions: **We have identified and targeted a cohort of MSM using our service at high risk of HIV seroconversion. In this group, risk‐reduction strategies are changing: over a quarter have initiated PrEP with half of these initiating PrEP in the last year. During follow‐up, their HIV incidence has dropped. The continuing high use of PEP suggests greater uptake of PrEP may be indicated.

## P017

### Sexual mixing and HIV risk among Greek men who have sex with men: results from SOPHOCLES

**B Bowman^1^, J Schneider^2^, D Paraskevis^3^, A Hatzakis^4^, S Chanos^5^, M Psichogyiou^6^, M Papadopoulou^4^, A Khanna^2^ and V Sypsa^4^**

^1^Pritzker School of Medicine, University of Chicago Medicine, Chicago, IL, USA. ^2^Medicine, University of Chicago Medicine, Chicago, IL, USA. ^3^Laboratory Sciences, University of Athens, Athens, Greece. ^4^Epidemiology, University of Athens, Athens, Greece. ^5^Testing, Athens Checkpoint, Athens, Greece. ^6^Internal Medicine, National and Kapodistrian University of Athens, Athens, Greece

**Background: **Greece experienced an unprecedented HIV outbreak in 2011 following economic crisis that has affected multiple subpopulations. Network level factors are increasingly understood to drive HIV transmission in emerging epidemics.

**Materials and methods: **HIV prevalence in Greece among MSM has increased as incidence among heterosexuals decreases and in the context of an effective response to the 2011 outbreak among people who inject drugs, accounting for 55.1% of new infections. We examined the relationship between HIV serostatus, risk behaviors and sexual networks among 1550 MSM in Athens, Greece. We generated networks using the chain referral structure within a HIV screening program. Network mixing coefficients (like‐with‐like or like‐with‐dislike) were calculated. Multiple logistic regression was used to assess the relationship between serostatus, sexual behaviors and sociodemographic indicators.

**Results: **One thousand five hundred and fifty samples were collected. Mixing of sexual network members who reported sex‐drug use was highly assortative (r = 0.37, σr = 0.32–0.42) and moderately assortative (r = 0.26, σr = 0.12–0.41) when stratified by HIV status. Random mixing characterized the network that reported condomless sex (r = 0.11, σr = 0.07–0.14), group sex (r = 0.00, σr = −0.02 to 0.01) and HIV status (r = 0.12, σr = 0.09–0.15). Regression analysis revealed that respondents who reported sex‐drug use were significantly less likely to report condomless sex (AOR 0.47) and vice versa (AOR 0.49). Conversely respondents who reported sex‐drug use were significantly more likely to report group sex (AOR 2.03) and vice versa (AOR 2.07).

**Conclusion: **This study represents the network analysis of the sexual mixing patterns of Greek MSM. Our findings highlight the possibility of serosorting among Athenian MSM based on sex‐drug use and HIV status, a pattern with potential to reduce transmission, slow the spread of HIV and facilitate prevention. Different levels of sexual mixing for different risk behaviors were evident with sex‐drug use demonstrating strong homophily. Notably, condomless sex and sex‐drug use tended to be mutually exclusive while sex‐drug use and group sex were strongly associated.

## P018

### Has the introduction of HIV pre‐exposure prophylaxis (PrEP) impacted on HIV post‐exposure prophylaxis for sexual exposure (PEPSE) prescriptions in MSM in Greater Glasgow and Clyde?

**L Gillespie^1^, M Lowrie^2^ and R Metcalfe^1^**

^1^Sandyford Sexual Health Service, NHS Greater Glasgow and Clyde, Glasgow, UK. ^2^School of Medicine, University of Glasgow, Glasgow, UK

**Background: **On 10 April 2017, the Scottish Medicine Consortium approved emtricitabine/tenofovir disoproxil for use as HIV PrEP, in combination with safer sex practices [1]. Greater Glasgow and Clyde (GG&C) health board is the largest health board in the UK, providing healthcare to over 1.2 million people [2]. The prevalence of HIV in MSM in GG&C is estimated at 5.4% [3]. PEPSE is available at sexual health clinics and emergency departments (ED). From July 2017, NHS‐funded HIV PrEP has been available from sexual health clinics. Between 1 July 2017 and 31 December 2017, there had been 924 PrEP prescriptions, to 435 MSM.

**Aim: **To assess whether the provision of NHS‐funded HIV PrEP has reduced the number of prescriptions of HIV PEPSE in GG&C.

**Methods: **A case note review of MSM prescribed PEPSE and meeting the national criteria [4], between 1 September and 31 December 2017, was performed and the number of prescriptions was compared to a previous audit cycle before the introduction of PrEP (between 1 November 2016 and 28 February 2017).

**Results: **Prior to the introduction of PrEP, there were 56 PEPSE prescriptions to 55 individuals meeting the criteria. Nine of 56 (16%) cases initially presented to ED. After the introduction of PrEP, there were 71 PEPSE prescriptions to 70 individuals. Fourteen of 71 (20%) cases initially presented to ED. Seven patients had been prescribed PrEP previously but not started, or were non‐adherent (Table 1).

**Table:** Abstract P018 – Table 1. PEPSE prescriptions before and after introduction of NHS‐funded PrEP

No. of PEPSE prescriptions	Overall	Started in emergency dept	Started in sexual health clinic
Pre‐introduction of PrEP	56	9	47
After introduction of PrEP	71	14	57

**Conclusions: **Despite a comprehensive, accessible, free‐of‐charge HIV PrEP service in NHS GG&C, we have seen an increase in PEPSE prescriptions. This includes an increase in presentations to emergency departments. HIV PrEP has received media attention. Third sector organisations, who lobbied for NHS‐funded PrEP in Scotland [5], have also been paramount in raising the profile of PrEP and sexual health services. MSM education, awareness of HIV risk and prior discussion/prescription of PrEP could have prompted the patient to present for PEPSE in the future. This information contributes to the literature as being the first home nation to provide NHS‐funded PrEP. It shows that despite this, numbers of patients presenting for PEPSE increased, highlighting the importance of using PrEP in combination with other risk reduction methods. We plan to re‐audit this again in 4 months.

**References**

[1] Scottish Medicines Consortium. [Internet] [cited 2018 Jun 18] Available from: http://www.scottishmedicines.org/SMC_Advice/Advice/1225_17_emtricitabine_tenofovir_disoproxil_truvada.

[2] NHS Greater Glasgow and Clyde. About us [Internet] [cited 2018 Jun 28]. Available from: http://www.nhsggc.org.uk/about‐us/.

[3] Health Protection Scotland. Blood borne viruses and sexually transmitted infections [Internet]. 2017 [cited 2018 Jun 19]. Available from: http://www.hps.scot.nhs.uk/resourcedocument.aspx?resourceid=3398.

[4] BASHH UK guideline for the use of HIV post‐exposure prophylaxis following sexual exposure. 2015.

[5] HIV Scotland. PrEP in Scotland [Internet] [cited 2018 Jun 18]. Available from: http://www.hivscotland.com/our‐work/prep‐in‐scotland/.

## P019

### Adherence of MSM participating in a partially self‐financed pilot PrEP project and its association with behavioural risk profiles

**T Kwan^1^, N Wong^2^, G Lui^3^, K Lee^2^ and S Lee^2^**

^1^Jockey Club School of Public Health & Primary Care, The Chinese University of Hong Kong, Hong Kong, China. ^2^Stanley Ho Centre for Emerging Infectious Diseases, The Chinese University of Hong Kong, Hong Kong, China. ^3^Department of Medicine and Therapeutics, The Chinese University of Hong Kong, Hong Kong, China

**Background: **HIV transmission in MSM accounts for a significant proportion of incident infections in Asia and the Pacific. Their access to pre‐exposure prophylaxis (PrEP) with tenofovir disoproxil fumarate/emtricitabine (TDF/FTC) remains extremely limited. In Hong Kong high cost of patented TDF/FTC and the uncertain degree of community acceptance are main obstacles impeding PrEP programme development. An effective and practicable service model is urgently needed.

**Materials and methods: **A pilot PrEP clinic was set up at a teaching hospital in Hong Kong to prescribe daily TDF/FTC to high‐risk MSM who were required to pay 13% of the actual drug cost over a 30‐week project period. Adherence, behavioural risk, use of psychotropic drugs for sex (chem‐sex) and sexually transmitted infections (STI) were monitored with online diary, point‐of‐care testing/sampling and the administration of tablet‐based questionnaires. Overall drug adherence was measured by the proportion of the number of days of use of TDF/FTC. Coverage of unprotected sex was defined by the use of two daily tablets taken before and after sex.

**Results: **Potentially eligible HIV‐negative PrEP‐naïve MSM were recruited from collaborating community organisations and HIV services, or self‐referred through an online platform. Between October 2017 and June 2018, 71 MSM (median age 32 years, interquartile range 27 to 40 years) joined the project following eligibility assessment. Participants’ risk profiles in the preceding 6 months were: unprotected anal intercourse (UAI) 87%, previous diagnoses of STI 18%; chem‐sex 24%. Baseline STIs were positive in 15% (syphilis 9%, urethral gonorrhoea 3%, urethral chlamydia 4%). Over a follow‐up period of 5146 person‐days with diary data, totally 746 person‐days with anal sex were recorded in the online diary, 68% of which were unprotected. Adherence to follow‐up clinic visits was 81%. Overall drug adherence was 87%. At least four daily tablets were taken in 91% (547/598) of person‐weeks with full diary data. Coverage of unprotected sex was 82%. No HIV seroconversions had occurred. There was no association between adherence and chem‐sex behaviours. Those reporting UAI had higher adherence but the difference did not reach statistical significance.

**Conclusions: **A partially self‐financed mode of PrEP delivery could be a feasible service model, as supported by high adherence of risk‐taking MSM irrespective of risk profiles. There was high though imperfect coverage of unprotected sex acts. The service has enabled regular monitoring of behavioural risk and STI/HIV to be implemented with the use of point‐of‐care testing and online diaries.

## P020

### HIV‐related stigma, motivation to adhere to ART, and ART adherence among HIV‐infected methadone‐maintained patients

**R Shrestha^1^, F Altice^2^ and M Copenhaver^1^**

^1^Allied Health Sciences, University of Connecticut, Storrs, CT, USA. ^2^Department of Internal Medicine, Yale University, New Haven, CT, USA

**Introduction: **Opioid agonist therapies with methadone are associated with higher levels of adherence to ART [1,2], yet no studies have explored factors associated with optimal ART levels in HIV‐infected patients on methadone maintenance treatment (MMT), including explanatory pathways using mediation analysis. Such findings would provide new insight to guide tailored and more effective HIV treatment as prevention (TasP) strategies [3] in this population. 

**Methods: **Enrolled HIV‐infected, methadone‐maintained patients who reported HIV‐risk behaviors (N = 133) were assessed using an audio computer‐assisted self‐interview (ACASI). Multivariable logistic regression was used to identify significant correlates and an ordinary least squares regression‐based path analytic framework to test the explanatory pathway (i.e. mediational effect) for optimal ART adherence.

**Results: **Among 133 participants, over 40% reported sub‐optimal adherence to ART. Optimal ART adherence was significantly associated with being virally suppressed (adjusted odds ratio [aOR] 6.470, *p* = 0.038), higher motivation to adhere to ART (aOR 1.171, *p* = 0.011) and lower anticipated HIV‐related stigma (aOR 0.384, *p* = 0.015). We also found a significant interaction effect that involved motivation to adherence to ART combined with drug injection to be correlated with optimal ART adherence (aOR 1.086, *p* = 0.049). Furthermore, results revealed an indirect effect of motivation on the relationship between HIV stigma and ART adherence (*B *=* *‐0.121, *p* = 0.043), thus supporting the mediation effect (Figure 1).

Abstract P020 – Figure 1. Statistical model of the mediational process.

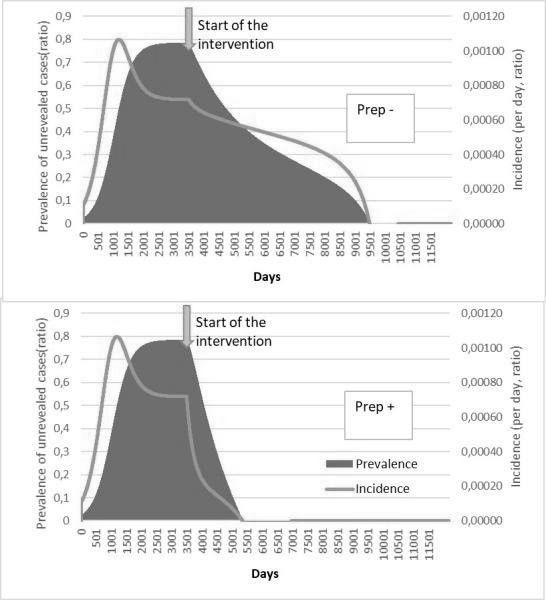

**Conclusion: **Our findings underscore the complexities surrounding ART adherence, even in patients on MMT. These findings provide insights on how to more effectively intervene to optimize HIV treatment outcomes, including HIV TasP initiatives [3], in opioid‐dependent patients on MMT.

**References**

[1] Zwahlen M, Harris R, May M, Hogg R, Costagliola D, de Wolf F, et al. Mortality of HIV‐infected patients starting potent antiretroviral therapy: comparison with the general population in nine industrialized countries. Int J Epidemiol. 2009;38:1624‐33.

[2] Low AJ, Mburu G, Welton NJ, May MT, Davies CF, French C, et al. Impact of opioid substitution therapy on antiretroviral therapy outcomes: a systematic review and meta‐analysis. Clin Infect Dis. 2016;63:1094‐104.

[3] Cohen MS, Chen YQ, McCauley M, Gamble T, Hosseinipour MC, Kumarasamy N, et al. Antiretroviral therapy for the prevention of HIV‐1 transmission. N Engl J Med. 2016;375:830‐9.

## TREATMENT STRATEGIES: NEW TREATMENTS AND TARGETS

## P021

### Two‐drug regimen of dolutegravir plus lamivudine (DTG + 3TC) is non‐inferior to dolutegravir plus tenofovir/emtricitabine (DTG + TDF/FTC) at 48 weeks in antiretroviral treatment‐naïve adults with HIV‐1 infection: subgroup analyses in the GEMINI studies

**C Orkin^1^, N Porteiro^2^, M Berhe^3^, R Dretler^4^, P Viciana^5^, Y Tseng^6^, C Oprea^7^, M Johnson^8^, V Kulagin^9^, C Man^10^, J Sievers^11^, A Currie^12^, M Underwood^13^, A Tenorio^10^, K Pappa^10^, B Wynne^10^, M Aboud^14^, K Smith^15^ and M Gartland^10^**

^1^Barts Health NHS Trust and Queen Mary University of London, London, UK. ^2^Fundacion IDEAA, Buenos Aires, Argentina. ^3^Texas Infectious Diseases Consultants, Dallas, TX, USA. ^4^Infectious Disease Specialists of Atlanta, Decatur, IL, USA. ^5^Hospital Universitario Virgen del Rocio, Sevilla, Spain. ^6^Kaohsiung Veterans General Hospital, Kaohsiung City, Taiwan. ^7^Dr. Victor Babes Clinical Hospital for Infectious and Tropical Diseases, Bucharest, Romania. ^8^Royal Free Hospital, London, UK. ^9^Clinical Center for Prevention and Treatment of AIDS and Infectious Diseases, Kasnodar, Russian Federation. ^10^Clinical Development, ViiV Healthcare, Research Triangle Park, NC, USA. ^11^Clinical Development, ViiV Healthcare, Brentford, UK. ^12^Statistics and Programming, GlaxoSmithKline, Stockley Park, UK. ^13^Clinical Virology, ViiV Healthcare, Research Triangle Park, NC, USA. ^14^Medical Affairs, ViiV Healthcare, Brentford, UK. ^15^Medical Strategy, ViiV Healthcare, Research Triangle Park, NC, USA

**Background: **Two‐drug regimens (2DR) are being evaluated against standard three‐drug regimens for their potential to reduce cumulative drug exposure and drug‐drug interactions during life‐long antiretroviral therapy in patients with HIV‐1 infection. In the GEMINI studies, the efficacy of the 2DR of DTG+3TC was recently shown to be non‐inferior to DTG + TDF/FTC at 48 weeks in treatment‐naïve adults [1].

**Methods and materials**

GEMINI‐1&2 are identical, global, double‐blind, multicenter Phase III studies evaluating efficacy and safety of DTG + 3TC once daily in treatment‐naïve HIV‐1 infected adults with screening HIV‐1 RNA ≤500,000 copies/mL (ClinicalTrials.gov: NCT02831673/NCT02831764). Participants were randomized 1:1 to treatment with DTG + 3TC or DTG + TDF/FTC, stratified by screening plasma HIV‐1 RNA and CD4 + cell count. The primary endpoint was the proportion of participants with plasma HIV‐1 RNA <50 copies/mL at Week 48 (Snapshot algorithm). We present a secondary analysis of the primary endpoint and of safety by demographic and baseline plasma HIV‐1 RNA and CD4+ cell count subgroups. For the primary endpoint, estimates and confidence intervals were based on a stratified analysis using Cochran‐Mantel‐Haenszel weights. The subgroup analysis was unadjusted.

**Results: **Seven hundred and fourteen and 719 adults were randomized and treated in GEMINI‐1&2, respectively. Participants were well matched for demographic and baseline characteristics. Based on a 10% non‐inferiority margin, DTG + 3TC was non‐inferior to DTG + TDF/FTC at Week 48 in both GEMINI‐1&2. Results were generally consistent regardless of age, gender, race or baseline HIV‐1 RNA (Table 1). Response rates in subjects with baseline HIV‐1 RNA >100,000 copies/mL were high and similar between arms. Across both studies, six participants on DTG + 3TC and four on DTG + TDF/FTC met protocol‐defined virologic‐withdrawal criteria through Week 48; none had treatment‐emergent integrase‐strand‐transfer‐inhibitor or NRTI resistance mutations. Overall rates of AEs were similar between arms, with low rates of withdrawals due to AEs in both arms [GEMINI‐1&2 pooled: DTG + 3TC 15/716 (2%) vs. DTG + TDF/FTC 16/717 (2%)]. More drug‐related AEs were reported with DTG + TDF/FTC [GEMINI‐1&2 pooled: DTG + 3TC 126/716 (18%) vs. DTG + TDF/FTC 169/717 (24%)]. The frequency of AEs was generally similar across subgroups.

**Table:** Abstract P021 – Table 1. Proportion of participants with plasma HIV‐1 RNA <50 copies/mL at Week 48: Snapshot analysis by subgroups – ITT‐E population

	GEMINI‐1		GEMINI‐2		Pooled	
	DTG + 3TC, n/N (%)	DTG + TDF/FTC, n/N (%)	DTG + 3TC, n/N (%)	DTG + TDF/FTC, n/N (%)	DTG + 3TC, n/N (%)	DTG + TDF/FTC, n/N (%)
Overall population	320/356 (90%)	332/358 (93%)	335/360 (93%)	337/359 (94%)	655/716 (91%)	669/717 (93%)
Adjusted difference and 95% CI	−2.6 (−6.7 to 1.5)	−0.7 (−4.3 to 2.9)	−1.7 (−4.4 to 1.1)
Age (years)						
<35	194/211 (92%)	191/205 (93%)	192/209 (92%)	190/203 (94%)	386/420 (92%)	381/408 (93%)
35−<50	100/116 (86%)	101/107 (94%)	111/115 (97%)	115/122 (94%)	211/231 (91%)	216/229 (94%)
≥50	26/29 (90%)	40/46 (87%)	32/36 (89%)	32/34 (94%)	58/65 (89%)	72/80 (90%)
Gender						
Female	52/59 (88%)	49/52 (94%)	48/54 (89%)	40/46 (87%)	100/113 (88%)	89/98 (91%)
Male	268/297 (90%)	283/306 (92%)	287/306 (94%)	297/313 (95%)	555/603 (92%)	580/619 (94%)
Race						
White	219/243 (90%)	232/248 (94%)	228/237 (96%)	239/249 (96%)	447/480 (93%)	471/497 (95%)
African heritage	39/44 (89%)	29/36 (81%)	44/55 (80%)	35/40 (88%)	83/99 (84%)	64/76 (84%)
Asian	34/37 (92%)	41/42 (98%)	33/34 (97%)	27/30 (90%)	67/71 (94%)	68/72 (94%)
Other	28/32 (88%)	30/32 (94%)	30/34 (88%)	36/40 (90%)	58/66 (88%)	66/72 (92%)
Baseline HIV‐1 RNA (copies/mL)						
≤100,000	255/282 (90%)	263/282 (93%)	271/294 (92%)	268/282 (95%)	526/576 (91%)	531/564 (94%)
>100,000	65/74 (88%)	69/76 (91%)	64/66 (97%)	69/77 (90%)	129/140 (92%)	138/153 (90%)
Baseline CD4 + (cells/mm^3^)						
≤200	25/31 (81%)	26/29 (90%)	25/32 (78%)	25/26 (96%)	50/63 (79%)	51/55 (93%)
>200	295/325 (91%)	306/329 (93%)	310/328 (95%)	312/333 (94%)	605/653 (93%)	618/662 (93%)

**Conclusions: **In GEMINI‐1&2, DTG + 3TC demonstrated non‐inferior efficacy to DTG + TDF/FTC in treatment‐naïve adults with screening HIV‐1 RNA ≤500,000 copies/mL at Week 48. Both regimens were well tolerated. Subgroup analyses of efficacy and safety performed based on baseline disease and demographic characteristics were generally consistent with overall study results. These results further demonstrate DTG + 3TC is an option for initial treatment of HIV‐infected patients across a spectrum of disease characteristics and patient populations. The studies are ongoing to explore long‐term durability and safety.

**Reference: **[1] Cahn P, Sierra Madero J, Arribas J, et al. Non‐inferior efficacy of dolutegravir (DTG) plus lamivudine (3TC) versus DTG plus tenofovir/emtricitabine (TDF/FTC) fixed‐dose combination in antiretroviral treatment‐naïve adults with HIV‐1 infection – 48‐week results from the GEMINI studies [abstract TUAB0106LB]. 22nd International AIDS Conference; 2018 Jul 23‐27; Amsterdam, Netherlands.

## P022

### Cerebrospinal fluid exposure of cenicriviroc in HIV‐positive individuals with cognitive impairment

**J Alagaratnam^1^, L Else^2^, S Dilly Penchala^2^, E Challenger^2^, K Legg^1^, C Petersen^3^, B Jones^4^, R Kulasegaram^5^, S Seyedkazemi^6^, E Lefebvre^7^, S Khoo^2^ and A Winston^1^**

^1^Department of Infectious Diseases, Imperial College London, London, UK. ^2^Department of Pharmacology, University of Liverpool, Liverpool, UK. ^3^Clinical Trials Centre, Imperial College London, London, UK. ^4^Department of Radiology, Imperial College Healthcare NHS Trust, London, UK. ^5^Department of GU/HIV, St Thomas’ Hospital, London, UK. ^6^Clinical Development, Allergan plc, South San Francisco, CA, USA. ^7^former employee of R&D, Allergan plc, South San Francisco, CA, USA

**Background: **Cenicriviroc, a dual C‐C chemokine receptor type 2 (CCR2) and type 5 (CCR5) antagonist, is a potential adjunctive therapy, along with ART, for the management of HIV‐associated cognitive disorders.

**Materials and methods: **Virologically suppressed PLWHIV with a clinical diagnosis of HIV‐related cognitive impairment intensified ART with cenicriviroc once daily, dose dependent on current ART, for 8 weeks. Subjects with current or previous use of CCR5 inhibitors were not eligible. We assessed cerebrospinal fluid (CSF) exposure of cenicriviroc and CSF albumin at Week 8, and changes in cognitive function over 8 weeks. Cenicriviroc concentration was determined using reverse phase high‐performance liquid chromatography (HPLC) with geometric mean (GM) and 95% CI calculated. The proposed cenicriviroc target concentration was above the 90% effective concentration (EC90) for cenicriviroc (0.17 ng/mL), with the lower limit of quantification (LLQ) 0.24 ng/mL taken as target concentration. Cognitive function assessment comprised of seven domains with composite Z‐scores reported.

**Results: **Four of seven enrolled participants completed all study procedures. Median age was 43 years (interquartile range [IQR] 39–47), current CD4 +  count 375 cells/µL (IQR 315–555) and plasma HIV RNA undetectable in all. At Week 8, CSF exposure was detected in two subjects (0.82 and 0.40 ng/mL) and below the LLQ in two (Table 1). Mean CSF: plasma cenicriviroc concentration ratio was no more than 0.18% (95% CI of the upper estimate 0.09%–0.28%). Median CSF: serum albumin ratio was 10.1 (IQR 7.2–19.5) and CSF albumin was higher in those with detectable cenicriviroc in the CSF. Overall cognitive performance Z‐score was −0.14 (95% CI −1.35 to 1.07) at baseline, and −0.27 (95% CI −1.70 to 1.17) at Week 8.

**Table:** Abstract P022 – Table 1. Individual subject blood and cerebrospinal fluid parameters at Week 8

Subject	1	2	3	4
CSF cenicriviroc concentration, ng/mL	0.82	0.40	0.24 (less than LLQ)	0.24 (less than LLQ)
Plasma cenicriviroc concentration, ng/mL	718.6	211.1	411.9	70.5
CSF: plasma cenicriviroc concentration ratio (%)	0.11	0.19	0.06	0.34
CSF albumin concentration, mg/mL	1070	453	374	202
Serum albumin concentration, g/L	38	42	40	40
CSF: serum albumin ratio	28.2	10.8	9.4	5.1
Antiretroviral therapy	abacavir, lamivudine, raltegravir	lamivudine, atazanavir, ritonavir	tenofovir DF, emtricitabine, dolutegravir	tenofovir DF, emtricitabine, raltegravir
Cenicriviroc dose	150 mg	50 mg	150 mg	150 mg

tenofovir DF = tenofovir disoproxil fumarate.

**Conclusion: **In PLWHIV with cognitive impairment, cenicriviroc CSF exposure is close to the EC90.

## P023

### Abstract withdrawn

## P024

### Abstract withdrawn

## P025

### Pharmacokinetics of MK‐8591, dolutegravir and tenofovir disoproxil fumarate are not altered after co‐administration when compared to single agent administration

**D Rudd^1^, S Zhang^1^, K Fillgrove^1^, S Fox‐Bosetti^1^, R Matthews^1^, E Friedman^1^, D Armas^2^, S Stoch^1^ and M Iwamoto^1^**

^1^Merck Research Laboratories, Merck Sharp & Dohme Corp., Kenilworth, NJ, USA. ^2^Preclinical Research, Celerion, Tempe, AZ, USA

**Background: **MK‐8591 is a highly potent nucleoside reverse transcriptase translocation inhibitor that is in development for the treatment of HIV‐1 infection. As MK‐8591 may be co‐administered with other antiretrovirals, a drug interaction study with dolutegravir (DTG) and tenofovir (TFV) disoproxil fumarate (TDF) was conducted. MK‐8591 is primarily eliminated by renal excretion and may be affected by drugs that inhibit renal transporters. DTG is primarily metabolized through glucuronidation (UGT1A1). DTG is known to inhibit renal transporters OCT2 and MATE1. TDF is a commonly used NRTI that is eliminated (as TFV) by glomerular filtration and active tubular secretion. TFV may compete with other compounds that are also renally eliminated. 

**Materials and methods: **The two‐way interaction between MK‐8591 and DTG+TDF was investigated in a two‐period, two‐way DDI study in 12 healthy adult subjects. Subjects received a single oral dose of 20 mg MK‐8591 on Day 1, followed by a washout of seven days. In Period 2, subjects received 11 days of 50 mg DTG/300 mg TDF once daily, with 20 mg MK‐8591 co‐administered on Day 8. PK for DTG and TFV was taken on Days 7 and 8, while PK for MK‐8591 was taken on Day 8.

**Results: **Administration of MK‐8591 alone and in combination with DTG and TDF was generally well tolerated. MK‐8591 AUC and Cmax were not meaningfully affected with DTG+TDF co‐administration, and the DTG and TDF AUC0‐24, C24 and Cmax were similar with and without MK‐8591 (Table 1). The apparent terminal half‐life of MK‐8591, DTG and TFV were not meaningfully affected with co‐administration.

**Table:** Abstract P025 – Table 1. Comparing MK‐8591, DTG and TFV PK post‐administration of DTG and TDF with/without MK‐8591 in healthy adult subjects

Geometric mean ratio (GMR) with 90% confidence interval, relative to single agent administration	N	MK‐8591	DTG	TDF^a^
AUC0‐24	12	1.08 (1.02 to 1.14)	1.05 (0.96 to 1.14)
AUC0‐inf	12	1.28 (1.9 to 1.37)	‐
C24	12	1.10 (1.02 to 1.17)	1.05 (0.97 to 1.14)
Cmax	12	1.07 (0.93 to 1.22)	1.02 (0.94 to 1.11)	0.98 (0.88 to 1.10)

^a^PK parameters shown are for TFV in plasma.

**Conclusions: **MK‐8591 had no clinically significant effect on the PK of DTG or TFV. While DTG is known to inhibit OCT2 and MATE1, there was no effect on the PK of MK‐8591. There also appears to be no competition between the renal elimination of MK‐8591 and TFV. These findings support co‐dosing of MK‐8591 and DTG+TDF, if indicated.

## P026

### “Treatment access cascades”: effects of viral load, resistance testing and safety in pregnancy on access to dolutegravir in low‐ and middle‐income countries

**J Levi^1^, P Clayden^2^ and A Hill^3^**

^1^Medicine, Imperial College London, London, UK. ^2^Medicine, HIV i‐Base, London, UK. ^3^Department of Pharmacology and Therapeutics, Liverpool University, Liverpool, UK

**Objectives: **Generic antiretroviral treatment with tenofovir disoproxil fumarate/lamivudine/dolutegravir (TDF/3TC/DTG; TLD) is available in eligible countries for $75/person‐year. There are plans to transition millions of HIV+ people to TLD in 2018 to 2019. The Phase III trial programme for dolutegravir excluded: pregnant women, ART‐naive with transmitted drug resistance and ART‐experienced without genotypic resistance testing. The DAWNING study excluded ART‐experienced people with no active NRTIs. Recent reports of neural tube defects (NTDs) after use of DTG at conception could limit use in women of childbearing potential.

**Methods: **Using UNAIDS 2016 data we made four cascades for each low‐/middle‐income country (LMIC) with available data. Cascade 1 shows all diagnosed HIV+ people, split into those on ART, those achieving HIV‐RNA suppression and those on ART but unsuppressed. We modelled the impact on access to TLD if patients could not be started or switched due to: lack of access to viral load testing (Cascade 2); or to resistance testing (Cascade 3); or due to risks associated with pregnancy (Cascade 4). We defined lack of access to viral load testing as <1 test in 12 months. We assumed 50% get resistance testing for Cascade 3. We defined HIV+ women of childbearing potential as those aged 15 to 49.

**Results: **Table 1 shows the effects on access to TLD after excluding patients without a HIV‐RNA test (Cascade 2), without access to resistance testing (Cascade 3) and removing women of childbearing potential (Cascade 4). Based on these exclusion criteria, the percentage of HIV+ diagnosed people able to start/switch to TLD ranged from 78% [Mexico] to 9% [Zimbabwe, Cameroon]. The largest exclusion factor was unknown safety for HIV+ women of childbearing age. There is varying access to viral load testing in LMICs ranging from 99% [Mexico, Brazil] to 1% [Swaziland].

**Table:** Abstract P026 – Table 1. Number and percentage of diagnosed HIV positive people, able to be safely started on or switched to TLD

Country	Cascade 1	Cascade 2	Cascade 3	Cascade 4	% diagnosed eligible to start TLD
Zimbabwe	975,000	178,425	168,570	84,285	9
Cameroon	420,000	75,860	74,207	36,619	9
Nigeria	1,024,000	212,992	208,712	114,791	11
DR Congo	133,200	37,176	35,656	18,541	14
Togo	63,000	20,135	19,473	10,674	17
Haiti	112,500	38,765	37,182	19,707	18
Botswana	306,000	114,138	112,655	61,960	20
Ethiopia	475,500	170,110	166,154	93,046	20
Kenya	816,000	374,381	369,291	188,339	23
Angola	112,000	52,248	52,119	25,538	23
Cote d'Ivoire	266,800	143,672	140,025	78,414	29
Vietnam	175,000	76,510	75,243	55,680	32
Malawi	935,000	618,596	603,127	325,689	35
South Africa	6,106,000	4,518,440	4,405,326	2,246,716	37
Thailand	337,500	279,788	267,299	165,725	49
Ukraine	134,400	115,772	108,589	70,583	53
Brazil	713,800	713,800	703,521	499,500	70
Mexico	138,600	137,450	132,894	107,644	78
Malaysia	92,150	85,681	85,239	75,863	82

**Conclusion: **The clinical development programme for DTG excluded pregnant women, and with low access to contraception and abortion in LMICs, along with the dangerous, ideologically driven “Global Gag Rule” limiting PEPFAR, serious changes are needed to improve options for HIV+ women. Re‐analysis of DTG pre‐conception is needed to determine whether the adverse safety signal is maintained in long‐term follow‐up. The lack of data in key populations limits the potential to transition large numbers of patients to TLD. We highlight the need for improved access to viral‐load and genotype testing in LMICs. New trials are needed to evaluate TLD in treatment‐experienced patients with limited or no NRTI options.

## P027

### Patient and provider experience of using dolutegravir in resource‐limited settings: acceptability findings from Uganda and Nigeria

**J Campbell^1^, I Amamilo^2^, V Nabitaka^3^, O Abudiore^4^, P Nawaggi^3^, W Eigege^5^, K Magambo^6^, J Conroy^7^, J Harwell^8^, C Middlecote^9^, C Amole^10^, S Akanmu^11^, O Agbaji^12^, J Abah^13^, D Anweh^13^ and J Musinguzi^14^**

^1^Applied Analytics Team, Clinton Health Access Initiative, Boston, MA, USA. ^2^HIV Program, Clinton Health Access Initiative, Abuja, Nigeria. ^3^HIV Care and Treatment, Country Program, Clinton Health Access Initiative, Kampala, Uganda. ^4^Country Program, Clinton Health Access Initiative, Abuja, Nigeria. ^5^HIV Access, Clinton Health Access Initiative, Abuja, Nigeria. ^6^Clinical Sciences Team, Clinton Health Access Initiative, Pretoria, South Africa. ^7^HIV Access, Clinton Health Access Initiative, Kampala, Uganda. ^8^Clinical Sciences Team, Clinton Health Access Initiative, Boston, MA, USA. ^9^HIV Access, Clinton Health Access Initiative, London, UK. ^10^HIV Access, Clinton Health Access Initiative, New York, NY, USA. ^11^Clinical, Lagos Teaching Hospital, Lagos, Nigeria. ^12^Clinical, Jos Teaching Hospital, Jos, Nigeria. ^13^Clinical, Federal Medical Center, Makurdi, Nigeria. ^14^HIV, Ministry of Health, Kampala, Uganda

**Background: **The World Health Organization recommends dolutegravir (DTG) as preferred first‐line therapy. Many countries are considering the fixed‐dose combination of tenofovir/lamivudine/dolutegravir (TLD) in their treatment protocols; however, information on use of this regimen in LMIC settings is limited. Pilot studies began in Uganda and Nigeria in July 2017 to learn from the experiences of patients taking DTG in these early adopter countries, and from the prescribing HCWs.

**Methods: **Patients received a side‐effect questionnaire after their initial one to two months of treatment. Treatment‐experienced patients were asked about changes in side‐effects and preferences for the new versus previous regimen. At the same time the HCWs prescribing DTG received questionnaires on their perceptions of patient management and patient experience. Study follow‐up will continue for six and twelve months for Uganda and Nigeria, respectively; these are interim findings. Data were analysed in SAS v.9.4 by country accounting for facility clustering.

**Results: **Interviews occurred August 2017 to March 2018. Three hundred and forty patients were interviewed in Uganda (159 treatment‐naïve, 181 treatment‐experienced), and 227 (25 naïve, 202 experienced) in Nigeria. Eight HCWs were interviewed in Nigeria and 12 in Uganda. Treatment‐experienced patients preferred their current (DTG) regimen (96% and 98% in Nigeria and Uganda, respectively). In Uganda, 43% of patients self‐reported experiencing at least one side‐effect, 10% reporting a severe side‐effect. In Nigeria, this was 51% and 13%, respectively. In both countries, “increase in appetite” was the most frequent side‐effect reported, followed by tiredness and headaches. Many patients acknowledged previous side‐effects were resolved (48% Uganda, 33% Nigeria) or improved after switching to TLD (36% Uganda, 61% Nigeria) and a minority reported at least one side‐effect worsened (13% Uganda, 5% Nigeria). “Bad dreams” was the most improved side‐effect in Uganda and second in Nigeria. HCWs believed their patients preferred TLD (93% in Uganda, 75% in Nigeria) and that they experienced fewer side‐effects (92% in Uganda, 88% in Nigeria). Availability of TLD beyond the study was the primary concern of HCWs (64% Uganda, 50% Nigeria).

**Discussion: **This is the first study to report patient experiences with DTG in routine clinical practice in LMIC. Treatment‐experienced patients that had intolerance to NNRTIs responded with high preference for DTG. Insomnia and neuropsychiatric side‐effects were not the most common. “Increase in appetite” was the most mentioned side‐effect and an unexpected finding. It is unclear if this is viewed as a positive or negative consequence. Longer follow‐up is needed to determine if this increased appetite leads to weight gain.

## P029

### Management of sexual health in HIV‐infected patients: a cross‐sectional survey among Dutch internist infectiologists and HIV nurses

**S de Munnik^1^, L Kraan^2^, H Ammerlaan^1^, J de Wit^3^, G Kok^4^, L Grondhuis^2^ and C den Daas^5^**

^1^Internal Medicine, Catharina Hospital, Eindhoven, Netherlands. ^2^Urology, Leiden University Medical Center, Leiden, Netherlands. ^3^Interdisciplinary Social Science, Utrecht University, Utrecht, Netherlands. ^4^Applied Social Psychology, Maastricht University, Maastricht, Netherlands. ^5^HIV/STI, National Institute for Public Health and the Environment, Bilthoven, Netherlands

**Background: **In the Netherlands, internist‐infectiologists and HIV nurses are responsible for an holistic approach of HIV care, of which sexual health is an important component. Not only because most common transmission route is via sexual contact, but most of all since studies have shown that sexual health issues among HIV patients are common. To improve sexual health among HIV patients, this topic should be addressed either by the infectiologist or by the HIV nurse or both. The study aim is to investigate whether sexual health is discussed, whose role it is to discuss and which barriers are responsible for not discussing this issue.

**Materials and methods: **A cross‐sectional survey was performed anonymously using a 40‐item questionnaire that was sent to all Dutch infectiologists (N = 110) and HIV nurses (N = 80) among 26 HIV treatment centres. The questionnaire comprised 40 questions assessing the following topics: demographic characteristics, current practice, possible barriers, presumed responsibility and factors that might contribute to implement (or not) sexual counselling in daily practice.

**Results: **In total, 64 out of 110 (58%) infectiologists completed the questionnaire, as well as 48 out of 80 (60%) HIV nurses. Most infectiologists (59%) do not screen routinely for sexual health issues, whereas 85% of HIV nurses do. A majority of the infectiologists (73%) consider themselves responsible for addressing sexuality, however more often regard HIV nurses responsible (94%). Among HIV nurses routine screening of sexual health issues is fully considered as their responsibility (100% of the HIV nurses). In total 56% of the infectiologists and 40% of the HIV nurses state that there are no guidelines which ensure routine discussion of sexual health. Both infectiologists and HIV nurses mention the presence of a third person during a consultation as a strong barrier. Furthermore, infectiologists indicate “insufficient time” (59%) and “no reason for asking” (42%) as barriers for not bringing up sexual health. In contrast, additional barriers indicated by HIV nurses are “language and ethnicity” (50%) and “culture or religion” (40%).

**Conclusions: **Sexual health is a crucial topic to address during routine consultation by health care providers within HIV care. However due to barriers sexual health issues are not discussed routinely with all patients. Health care providers seem to agree that HIV nurses are responsible for addressing this issue. Removing barriers and composing guidelines on how to address sexual health might improve the holistic care of dedicated health care providers within HIV care.

## TREATMENT STRATEGIES – TARGET POPULATIONS: WOMEN, ADOLESCENTS AND CHILDREN

## P030

### Cumulative safety review of elvitegravir and bictegravir use during pregnancy and risk of neural tube defects

**T Farrow^1^, C Deaton^2^, N Nguyen^1^, M Serejo^1^, D Muramoto^1^, A van Troostenburg^3^, L Ng^1^, L Liu^1^, H Martin^4^ and M Das^4^**

^1^Pharmacovigilance & Epidemiology, Gilead Sciences, Foster City, CA, USA. ^2^Pharmacovigilance & Epidemiology, Gilead Sciences, Cambridge, UK. ^3^Pharmacovigilance & Epidemiology, Gilead Sciences, Uxbridge, UK. ^4^Clinical Research, Gilead Sciences, Foster City, CA, USA

**Background: **The global prevalence of neural tube defects (NTDs) is estimated to be 0.18% (95% CI 0.15 to 0.23) [1]. Preliminary findings from an ongoing birth outcome surveillance study in Botswana (Tsepamo study) suggested an increased risk of NTDs in infants born to mothers treated with dolutegravir, an integrase strand transfer inhibitor (INSTI), at the time of conception [2]. Reported pregnancies in the Gilead global safety database were reviewed to evaluate the number of NTD cases and to assess the risk of NTDs associated with exposure to the INSTIs, elvitegravir (EVG) or bictegravir (BIC).

**Materials and methods: **Cumulative from beginning of clinical development to 31 May 2018, all pregnancy cases reported for women exposed to EVG‐ and BIC‐containing products were retrieved from the Gilead global safety database, which included reports from clinical trials, spontaneous postmarketing reports and literature review. Given that the exact timing of medication exposure relative to conception is often unconfirmed in spontaneous reports, cases of NTDs diagnosed either anatomically or radiologically were identified regardless of the trimester of exposure. A prevalence rate could not be derived from these data, as many cases originated from retrospective reports, drawn from a population in which the number of exposed pregnancies is unknown [3].

**Results: **For EVG‐containing products, 630 pregnancies were identified. There was one retrospectively identified case of a fetal NTD reported during the pregnancy of a 34‐year‐old woman in the US who received EVG/cobicistat/emtricitabine (FTC)/tenofovir alafenamide prior to conception and then switched to raltegravir + FTC/tenofovir disoproxil fumarate 48 days post last menstrual period (LMP). An ultrasound 19 weeks post LMP showed anencephaly. Obstetric history, other risk factors for NTD and folate supplementation were not reported. The pregnancy was ongoing and pending birth outcome. For BIC‐containing products, 25 pregnancy cases were identified. No NTDs were reported.

**Conclusions: **A search of the Gilead global safety database identified one case of NTD with pending birth outcome in a pregnancy of a woman exposed to EVG prior to conception. Viewed in the context of more than 600 pregnancy cases in women exposed to EVG, this single case cannot be distinguished from the background rate in the general population. Review of the limited data for BIC identified no cases of NTD.

**References**

[1] Blencowe H, Kancherla V, Moorthie S, Darlison MW, Modell B. Estimates of global and regional prevalence of neural tube defects for 2015: a systematic analysis. Ann N Y Acad Sci. 2018;1414:31‐46.

[2] Zash R, Makhema J, Shapiro RL. Neural‐tube defects with dolutegravir treatment from the time of conception. N Engl J Med. 2018 Jul 24;https://doi.org/10.1056/nejmc1807653. [epub ahead of print].

[3] Antiretroviral Pregnancy Registry Steering Committee. Antiretroviral pregnancy registry interim report for 1 January 1989 through 31 January 2018 [Internet]. Wilmington, NC: Registry Coordinating Center; 2017. Available from: http://www.APRegistry.com.

## P031

### Rates of pregnancy and preterm birth in Central/Eastern Europe and neighbouring countries: data from ECEE Network Group

**J Kowalska, on behalf of the ECEE Network Group**

Department of Children's Infectious Diseases, Medical University of Warsaw, Warsaw, Poland

**Background: **Epidemiological data on pregnancy and delivery outcomes among HIV‐positive women in Central/Eastern Europe and neighbouring countries (CEEN) are urgently needed in order to provide appropriate standards of care.

**Methods: **Euroguidelines in Central and Eastern Europe (ECEE) Network Group was established in February 2016 to review standards of care for HIV in the region. In 2018 information was collected regarding obstetric care standards, pregnancy rates and outcomes through an online questionnaire. All network members were invited to participate and to distribute invitation to other centres.

**Results: **Data from 24 centres in 20 countries were received; 58.3% of the centres were mainly infectious diseases clinics/hospitals, only 12.5% were exclusively HIV clinics. In half of the clinics a gynaecologist was admitting patients on a daily or weekly basis. In 78.3% of centres the most commonly advised method for contraception was condom use and unprotected intercourse was the most common recommendation for conception in 69.6%. The preferred combined antiretroviral treatment (cART) regimen used in pregnancy was integrase strand inhibitor (InSTI)+2NRTI (11; 47.8%), followed by protease inhibitor (PI)+2NRTI (six; 26.1%) and NNRTI+2NRTI (two; 8.7%). In four (17.4%) centres PI+InSTI+2NRTI was preferred option. In five centres InSTI are not available. The most commonly used 2NRTIs were TDF+FTC/3TC (87.5%). Of 70,568 patients in care 24,299 (34.4%) were women, 2132 (8.8%) newly diagnosed in 2017 and 306 (14.3%) while being pregnant. In total 1170 pregnancies were registered in 2017, which contributes to 4.8% (95% CI 4.5% to 5.1%) of the overall HIV‐positive women population (Table 1). Pregnancy outcomes were reported by 17 centres. Of 630 reported pregnancies 72 were still pregnant at the time of reporting and 33 were abortions. Of 525 live births 327 (62.3%) were term births, 89 (16.9%) were preterm births and 103 (19.6%) with unknown status.

**Table:** Abstract P031 – Table 1. Pregnancy rates in Central and Eastern Europe

Country	City	HIV+ under care	HIV+ women currently under care	New HIV+ women registered in 2017	HIV+ women registered in 2017 diagnosed HIV+ in pregnancy	Pregnancies registered in 2017 among HIV+ women
Total N		70568	24299	2132	306	1170
Russia	St. Petersburg	32205	11482	865	95	466
Republic of Moldova	Chisinau	7290	3961	368	87	220
Ukraine	Lviv	2732	1336	155	40	71
Belarus	Minsk	2830	1260	188	21	74
Ukraine	Kyiv	2869	1187	74	2	30
Romania	Bucharest	2686	1000	57	10	135
Georgia	Tbilisi	3200	950	120	12	40
Estonia	Tallin	2000	730	51	4	37
Poland	Warsaw	3358	502	39	3	13
Armenia	Yerevan	1200	430	103	22	37
Hungary	Budapest	2050	320	21	2	5
Serbia	Belgrade	1592	321	14	0	7
Albania	Tirana	530	160	21	0	2
Greece	Athens	939	136	6	2	4
Czech Republic	Prague	1627	126	13	4	8
Croatia	Zagreb	1138	118	6	0	3
Lithuania	Vilnius	385	88	23	2	10
Turkey	Izmir	517	72	1	0	5
Slovakia	Bratislava	590	61	4	0	4
Czech Republic	Plzeň	126	28	3	0	3
Bosnia and Herzegovina	Sarajevo	127	15	0	0	1
Bosnia and Herzegovina	Bania Luca	59	11	0	0	0
Kosovo	Prishtina	26	5	0	0	0

**Conclusions: **Only in 2017 and only in 24 CEEN centres there were as many as 1170 pregnancies and 525 deliveries. Preterm birth rates among HIV‐positive women are high, family planning options poor and access to modern cART unsatisfactory. More attention should be put on obstetric and gynaecological care for HIV‐positive women in this part of Europe.

## P032

### Live births, spontaneous and induced abortions in the Swiss HIV Cohort Study (SHCS): which factors may predict pregnancy outcomes?

**A Hachfeld^1^, A Atkinson^1^, A Calmy^2^, B Martinez de Tejada^3^, B Hasse^4^, P Paioni^5^, C Kahlert^6^, N Boillat‐Blanco^7^, M Stoeckle^8^ and K Aebi‐Popp^1^**

^1^Department of Infectious Diseases, Bern University Hospital, University of Bern, Bern, Switzerland. ^2^Department of Infectious Diseases, University Hospital Geneva, Geneva, Switzerland. ^3^Department of Obstetrics and Gynecology, Faculty of Medicine, University Hospital Geneva, Geneva, Switzerland. ^4^Division of Infectious Diseases and Hospital Epid., University Hospital and University of Zurich, Zurich, Switzerland. ^5^Division of Infectious Diseases and Hospital Epid., University Children's Hospital Zurich, Zurich, Switzerland. ^6^Division of Infectious Diseases, Children's Hospital of Eastern Switzerland, St. Gallen, Switzerland. ^7^Department of Infectious Diseases, University Hospital Lausanne, Lausanne, Switzerland. ^8^Department of Infectious Diseases, University Hospital Basel, Basel, Switzerland

**Background: **Since the release of the Swiss statement in 2008 we observed increasing rates of condomless sex in the Swiss HIV Cohort Study (SHCS). Despite the possibility of natural conception the total number of obstetric events (live births, spontaneous and induced abortions) did not increase. In a previous analysis we found an age‐dependent increase in spontaneous abortions over time, while the rate of induced abortions remained twice as high as in HIV‐negative women. The aim of this study was to identify risk factors for spontaneous and induced abortions and to identify predictors of pregnancies ending with a live birth.

**Materials and methods: **We assessed the occurrence of obstetric events in women aged 18 to 49 years between January 2009 and December 2016 in the SHCS. We used descriptive statistics to analyse demographic and clinical characteristics and uni‐ and multivariate logistic regression models to identify predictors of live birth and risk factors of spontaneous and induced abortions.

**Results: **We evaluated 2722 women and found 534 (65.3%) live births, 142 (17.4%) spontaneous abortions and 142 (17.4%) induced abortions over 8 years. Ethnicity, education level and CD4 cell counts did not predict pregnancy outcomes. Women with spontaneous abortions had a higher median age compared to those with live births (35 vs. 33 years, *p *≤ 0.001). Spontaneous abortions occurred more frequently among women who consumed alcohol (adjusted odds ratio (OR) 2.8, 95% CI 1.4 to 5.6, *p* = 0.004), but were less likely to occur if the HIV viral load was suppressed (adjusted OR 0.3, 95% CI 0.1 to 0.7, *p *≤ 0.001). Induced abortions were 2.4 times more likely in perinatally infected women (adjusted OR 2.4, CI 1.0 to 5.9, *p* = 0.05) but less likely among women in a stable partnership (adjusted OR 0.4, 95% CI 0.2 to 0.8, *p *≤ 0.001) and a suppressed viral load (adjusted OR 0.2, 95% CI 0.1 to 0.5, *p *≤ 0.001) (Table 1).

**Conclusions: **HIV suppression and alcohol abstinence are associated with higher odds of having a live birth. The high rate of induced abortions and the age‐dependent rate of spontaneous abortions underline the unmet needs of timely family planning and effective contraception in women living with HIV.

**Table:** Abstract P032 – Table 1. Multivariate logistic regression of live birth versus spontaneous abortion and live birth versus induced abortion adjusted for: age, mode of HIV infection, stable sex partner, viral HIV suppression, ART regimen, time on ART, oral hormonal contraception, alcohol consumption, depression, psychiatric treatment

	Live birth vs. spontaneous abortion (OR, 95% CI)	*p* value	Live birth vs. induced abortion (OR, 95% CI)	*p* value
Age at obstetric event	1.5 (1.2 to 1.8)	<0.001	not significant
Perinatally infected	not significant	2.4 (1.0 to 5.9)	0.05
Stable sex partner	not significant	0.4 (0.2 to 0.8)	<0.001
Suppressed HIV load	0.4 (0.2 to 0.6)	<0.001	0.2 (0.1 to 0.5)	<0.001
Alcohol consumption	2.8 (1.4 to 5.6)	0.004	not significant

## P033

### A shift in the risk factors of women diagnosed with HIV‐1 in Israel

**K Olshtain‐Pops^1^ and O Mor^2^**

^1^Infectious Diseases, Hadassah Medical Center, Jerusalem, Israel. ^2^National HIV Reference Center, Tel Hashomer, Tel Aviv, Israel

**Background: **Epidemiology of HIV‐1 infected women in Western countries is changing. First, the number of new infections yearly has significantly increased since the 1990s. In addition, risk factors have changed. While drug abuse was the most significant risk factor in the past, during the last decade heterosexual contact has become the leading risk factor. In Israel, epidemiological data of women with HIV are lacking. According to the Israeli Ministry of Health report for 2017, 54% of HIV‐infected women originated from a country with a generalised epidemic (OGE), mainly Ethiopia. Of the other 46%, nearly 60% belong to an unknown risk group. We evaluated the demographic characteristics and risk factors of women newly diagnosed with HIV in Israel during 2010 to 2017.

**Methods: **The National HIV Reference Center database was screened for all records of patients newly diagnosed with HIV during the years 2010 to 2017. Only female patients aged 18 years or above were selected for further analysis. Foreign citizens were excluded. Demographic criteria including age and country of origin were recorded. In addition, risk factors, as reported by the testing centre, were also assessed. The chi‐squared test was used to calculate the difference in the distribution of risk factors between the different years.

**Results: **A total of 637 women were diagnosed in Israel during the years 2010 to 2017. An average of 80 women (range 66 to 88) a year, with no significant difference between years. The average age was 38.6 years. Thirty‐two percent (179/557) of all women in the cohort were classified as OGE, 55% (308/557) were heterosexual and 11% (62/557) were IV drug users. The proportions of women in the different risk groups changed significantly over the years. During the years 2010 to 2011, 47% (40/86) of newly diagnosed women were OGE and 32% (28/86) were heterosexual. In contrast, during the years 2016 to 2017, 24% (16/66) of the newly diagnosed women were OGE and 65% (43/66) were heterosexual. These differences were statistically significant (*p* < 0.001).

**Conclusions: **According to our results, the epidemiology of HIV‐infected women in Israel is changing. In recent years, the leading risk factor has shifted from originating in a country with a generalised epidemic, to heterosexual contact. It is important to acknowledge these differences in order to plan prevention strategies appropriately.

## P034

### Assessment of the acceptability and swallowability of darunavir‐containing fixed‐dose combination (FDC) tablets in HIV‐1‐infected adolescents, using matching placebo tablets

**H Crauwels^1^, D Kurland^2^, P Chetty^3^, M Opsomer^1^ and S Vanveggel^1^**

^1^Pharmaceutica NV, Janssen, Beerse, Belgium. ^2^Research & Development LLC, Janssen, Titusville, NJ, USA. ^3^Research & Development, Janssen, High Wycombe, UK

**Background: **Two once‐daily darunavir‐containing FDC tablets are available for treating HIV‐1‐infected adults: darunavir/cobicistat 800/150 mg FDC and the single‐tablet regimen darunavir/cobicistat/emtricitabine/tenofovir alafenamide (D/C/F/TAF) 800/150/200/10 mg. Based on data for the separate agents, the respective FDCs with adult doses are also suitable for HIV‐infected adolescents. We assessed the acceptability/swallowability of both tablets (each administered as matching placebo tablets) in HIV‐1‐infected, treatment‐experienced adolescent patients.

**Methods: **TMC114FD2HTX1003 (NCT02993237) was a Phase I, open‐label, randomised, single‐dose, 2x2 crossover study in HIV‐1‐infected adolescents aged ≥12 to <18 years and weighing ≥40 kg. The study consisted of a screening period (including an assessment of the willingness and ability to swallow a reference placebo tablet matching the size of a PREZISTA tablet) and a one‐day open‐label administration phase during which patients were randomised 1:1 to one of two intake sequences for 1x D/C/F/TAF FDC placebo tablet and 1x darunavir/cobicistat FDC placebo tablet. Both intakes were separated by ≥30 minutes. Randomisation was stratified by age category (≥12 to <15 years; ≥15 to <18 years). Swallowability of the tablets was assessed using a seven‐point questionnaire to be completed immediately following each intake of drug. The same questions were asked regarding the reference placebo tablet and the current antiretroviral treatment. Swallowability scores were dichotomised into acceptability proportions with 95% confidence intervals. Acceptability for long‐term daily use was assessed using a three‐point questionnaire.

**Results: **Twenty‐seven patients (51.9% male; 33.3% Caucasian; 51.9% aged ≥12 to <15 years and 48.1% aged ≥15 to <18 years) participated. All completed the study as planned. There were no screening failures related to non‐willingness or inability to swallow the reference tablet. The D/C/F/TAF fDC placebo tablet was evaluated as easy to swallow by 25 patients (92.6%) and the darunavir/cobicistat FDC placebo tablet by 27 patients (100%), with the majority evaluating the swallowability as very easy (63.0% and 59.3%, respectively) (Table 1). Almost all patients evaluated each tablet at least acceptable to take for a longer period (92.6% and 100%, respectively) (Table 1). Most patients swallowed the D/C/F/TAF and darunavir/cobicistat FDC placebo tablets with water (88.9% and 88.9%, respectively), and none needed a second attempt at swallowing these tablets. There was no relevant difference by age group for administration of either the D/C/F/TAF fDC placebo tablet or the darunavir/cobicistat FDC placebo tablet. No adverse events were reported.

**Table:** Abstract P034 – Table 1. Swallowability/acceptability questionnaire outcomes

Parameter	Current ARV treatment^a^ (N = 27)	Screening placebo (N = 27)	D/C/F/TAF placebo (N = 27)	Darunavir/cobicistat placebo (N = 27)
How difficult/easy to swallow tablet, n (% to cumulative %)				
Very easy	18 (66.7% to 66.7%)	12 (44.4% to 44.4%)	17 (63.0% to 63.0%)	16 (59.3% to 59.3%)
Moderately easy	5 (18.5% to 85.2%)	6 (22.2% to 66.7%)	3 (11.1% to 74.1%)	4 (14.8% to 74.1%)
Slightly easy	2 (7.4% to 92.6%)	2 (7.4% to 74.1%)	4 (14.8% to 88.9%)	5 (18.5% to 92.6%)
Neither difficult nor easy	2 (7.4% to 100%)	1 (3.7% to 77.8%)	1 (3.7% to 92.6%)	2 (7.4% to 100%)
Slightly difficult	0	4 (14.8% to 92.6%)	1 (3.7% to 96.3%)	0
Moderately difficult	0	0	0	0
Very difficult	0	2 (7.4% to 100%)	1 (3.7% to 100%)	0
Swallowing acceptability, n (%) (95% CI)				
Easy^b^	27 (100%)	21 (77.8%)	25 (92.6%)	27 (100%)
(87.2% to 100%)	(57.7% to 91.4%)	(75.7% to 99.1%)	(87.2% to 100%)
Difficult^c^	0 (0.0%)	6 (22.2%)	2 (7.4%)	0 (0.0%)
(0.0% to 12.8%)	(8.6% to 42.3%)	(0.9% to 24.3%)	(0.0% to 12.8%)
Swallowing acceptability should tablet be taken once daily over a longer period, n (% to cumulative %)				
Good to take	14 (51.9% to 51.9%)	14 (51.9% to 51.9%)	15 (55.6% to 55.6%)
Acceptable	9 (33.3% to 85.2%)	11 (40.7% to 92.6%)	12 (44.4% to 100%)
Not acceptable	4 (14.8% to 100%)	2 (7.4% to 100%)	0

^a^all patients received ≥1 FDC in their current antiretroviral regimen, most commonly elvitegravir/cobicistat/emtricitabine/tenofovir disoproxil fumarate (12 [44.4%] patients), rilpivirine/emtricitabine/tenofovir disoproxil fumarate, dolutegravir/abacavir/lamivudine and abacavir/lamivudine (4[14.8%] patients each). ^b^Easy = ‘Very easy’, ‘Moderately easy’, ‘Slightly easy’, ‘Neither difficult nor easy’. ^c^Difficult = ‘Slightly difficult’, ‘Moderately difficult’, ‘Very difficult’. 95% CI – Clopper‐Pearson exact confidence interval for binomial proportion.

**Conclusions: **Both the D/C/F/TAF and darunavir/cobicistat FDC placebo tablets were considered acceptable to swallow and acceptable for use over a longer period by almost all HIV‐1‐infected adolescent patients.

## P035

### NRTI long term mitochondrial side effects in a paediatric HIV population: simplified therapy is a solution

**S Bernardi^1^, F Leone^2^, H K Tchidjou^1^, P Zangari^1^, G Polti^1^, P Palma^1^, L Palandri^3^ and A Bertoli^4^**

^1^University Immunology and Paediatric Dept, Bambino Gesu’ Children's Hospital, Rome, Italy. ^2^Paediatric, University of Rome, Rome, Italy. ^3^Hygiene and Medicine, Parma University, Parma, Italy. ^4^Virology, Tor Vergata University, Rome, Italy

**Background: **HAART in paediatric HIV infection has resulted in a substantial reduction in HIV‐associated mortality and morbidity. Although there are many evidences for side effects of long‐term treatment particularly related to NRTI use. Currently the paediatric guidelines suggest to start HAART very early with the mandatory NRTI backbone and the treatment need to continue forever. Many studies showed that NRTI‐exposed children develop symptomatic mitochondrial toxicity.

**Material and method**

We describe five paediatric HIV cases that showed after long‐time treatment a symptomatic myalgia with related high level of serum creatine phosphokinase (CPK). The customised choice of treatment NUC sparing has determined a prompt resolution of symptoms.

**Results: **In a group of 80 children born with HIV infection in HAART we describe five of these (6.25%), all male, three black with sudden myalgia without any fever and history of trauma. All five patients do not have any evidence of HIV/AIDS symptoms, HIV viral load <40 copies also CD4 was ≥25% median 500 cells/mm^3^ from more than 10 years; no evidence of other infections. All patients were in HAART – NUC included from 202 .8M . At observation none of them taking other conventional and unconventional drugs. The CPK median level was 6560.2, the other liver enzymes was normal also cardiac enzymes and renal function; the electrocardiography was normal. The ART was changed for mitochondrial toxicity hypothesis and the “Dual“ regimen was selected. All patients stopped NUC combine treatment and started integrase inhibitor (II) + protease inibitor (PI) in three patients, II + N‐Nuc in one patient and II + 3TC in another one patient. The choice of the new treatment was driven by initial resistance testing value. After stop NUC treatment all patients showed a rapid decline of serum CPK and subsequently symptoms resolution in three to seven days.

**Conclusions: **The simplifed ART was a solution of toxicities and was well tolerated in all patients. After 2 years of follow‐up there were no signs of drug‐related reactions, no toxicities and achieved virological suppression. Further studies of larger paediatric populations could be performed.

## TREATMENT STRATEGIES – TARGET POPULATIONS: LATE PRESENTERS

## P037

### Molecular analysis of HIV‐1 subtype A1 and B dispersal patterns of persons with late presentation and advanced disease in Greece

**E Kostaki^1^, N Pantazis^1^, P Gargalianos^2^, G Xylomenos^2^, M Chini^3^, N Mangafas^3^, G Metallidis^4^, O Tsachouridou^4^, A Skoutelis^5^, V Papastamopoulos^5^, D Chatzidimitriou^6^, E Kakalou^5^, A Antoniadou^7^, A Papadopoulos^7^, M Psichogiou^8^, G Daikos^8^, M Gova^1^, S Limneos^1^, D Paraskeva^9^, D Pilalas^10^, G Chrysos^11^, V Paparizos^12^, S Kourkounti^12^, H Sambatakou^13^, N Sipsas^14^, M Lada^15^, P Panagopoulos^16^, E Maltezos^16^, S Drimis^11^, A Hatzakis^1^, L Skoura^6^, M Lazanas^3^, G Touloumi^1^ and D Paraskevis^1^**

^1^Dept of Hygiene, Epidemiology & Medical Statistics, Medical School, National and Kapodistrian University of Athens, Athens, Greece. ^2^1st Department of Internal Medicine, General Hospital G. Gennimatas, Athens, Greece. ^3^Infectious Diseases Unit, Red Cross General Hospital of Athens, Athens, Greece. ^4^1st Department of Internal Medicine, AHEPA University Hospital, Aristotle University Medical School, Thessaloniki, Greece. ^5^5th Department of Medicine and Infectious Disease, Evangelismos General Hospital, Athens, Greece. ^6^Department of Microbiology, National AIDS Reference Centre of Northern Greece, Aristotle University Medical School, Thessaloniki, Greece. ^7^4th Department of Internal Medicine, Attikon University General Hospital, Medical School, National and Kapodistrian University of Athens, Athens, Greece. ^8^1st Department of Medicine, Laikon General Hospital, Medical School, National and Kapodistrian University of Athens, Athens, Greece. ^9^Office for HIV/AIDS and STDs, Hellenic Center for Diseases Control and Prevention, Marousi, Greece. ^10^School of Medicine, Aristotle University of Thessaloniki, Thessaloniki, Greece. ^11^Department of Internal Medicine, Tzaneio General Hospital, Piraeus, Greece. ^12^HIV/AIDS Unit, A. Syngros Hospital of Dermatology and Venereology, Athens, Greece. ^13^HIV Unit, 2nd Department of Internal Medicine, Hippokration General Hospital, Medical School, National and Kapodistrian University of Athens, Athens, Greece. ^14^Department of Pathophysiology, Laikon General Hospital, Medical School, National and Kapodistrian University of Athens, Athens, Greece. ^15^2nd Department of Internal Medicine, Sismanogleio General Hospital, Athens, Greece. ^16^Department of Internal Medicine, University General Hospital, Democritus University of Thrace, Alexandroupolis, Greece

**Background: **Late presentation of HIV infection is a serious challenge for the management and prevention of HIV infection in Europe. We aimed to investigate the patterns of HIV transmission among late presenters in Greece using molecular epidemiology in order to identify risk factors and gaps that need to be addressed at a national level.

**Materials and methods: **Study samples included HIV‐1 sequences isolated from 6190 patients diagnosed between 1999 and 2015 in Greece. We analysed 1777 (28.7%) and 2589 (41.8%) sequences of the subtype A1 and B, respectively, which are the most prevalent subtypes in Greece. Phylogenetic analysis was performed on subtypes A1 and B sequences from Greece along with globally sampled sequences used as references. Local transmission networks (LTNs) were considered as phylogenetic clusters including sequences from Greece at proportions >70%. Multivariable logistic regression models were applied for the statistical analysis. Late presenters were defined as persons with initial CD4 count between 200 and 350 cells/μL; those with advanced disease had an initial CD4 count <200 cells/μL or clinical AIDS regardless of CD4 count.

**Results: **Phylogenetic analysis revealed that 93.8% (1667 of 1777) of subtype A1 sequences formed 38 LTNs. For subtype B 77.1% (1996 of 2589) formed 166 LTNs. For subtype A1, the percentage of PLWHIV within LTNs was 95.2% (N = 197) for late presenters, 96.1% (N = 223) for those with advanced disease and 95.5% (N = 446) for non‐late presenters. For subtype B, the corresponding figures were 85.8% (N = 206) for late presenters, 71.8% (N = 290) for those with advanced disease and 89.8% (N = 569) for non‐late presenters. Multivariable logistic regression analysis showed that risk group (MSM vs. heterosexuals; OR 6.1; *p* < 0.001) and nationality (Greek vs. non‐Greek; OR 7.5; *p* < 0.001) were associated with regional clustering of subtype A1, while period of sampling (later sampling year; OR 1.13 per year; *p* < 0.001) was associated with regional clustering of subtype B. Late presentation or advanced disease status was not associated with regional clustering of subtype A1. Notably, PLWHIV with advanced disease had a lower probability (OR 0.48 vs. non‐late presenters; *p* < 0.001) of belonging to regional clusters of subtype B.

**Conclusions: **Most transmissions among PLWHIV with late presentation for subtypes A1 and B and those with advanced disease for subtype A1 occur locally (LTNs), calling for an intensification of testing. This is one of the few studies combining molecular and traditional epidemiology to study HIV dispersal patterns of PLWHIV with late diagnosis and advanced disease.

## P038

### Missed opportunities for HIV and viral hepatitis testing in the Danish health care system

**D Raben^1^, L Peters^1^, S Cowan^2^, M Jakobsen^1^ and A Mocroft^3^**

^1^Infectious Disease Dept, CHIP ‐ Centre of Excellence for Health, Immunity and Infections, Rigshospitalet, Copenhagen, Denmark. ^2^Infectious Diseases, Epidemiology and Prevention, Statens Serum Institut, Copenhagen, Denmark. ^3^Institute for Global Health, University College London, London, UK

**Background: **Late presentation for care for HIV and viral hepatitis results in poor outcomes for the individual and for public health [1]. We aimed to describe health care seeking behaviour of people diagnosed with HIV, HBV or HCV 2 years prior to diagnosis.

**Methods: **Data from the Danish National Patient Registry and the National Health Insurance Service Registry on all people diagnosed with HIV, HCV or HBV between 1 January 2013 and 31 December 2014 and their visits to general practitioners (GPs) and hospital departments in the 2 years prior to and up to seven days before the diagnosis was included. Very late presentation (VLP) was defined as presentation with hepatic decompensation or hepatocellular carcinoma in persons with HBV or HCV and an AIDS diagnosis in those with HIV.

**Results: **Four hundred and ninety‐five people were diagnosed (74.7% male, median age 35, IQR 27 to 43) with HIV; 32 (6.5%) were late presenters. 82.8% had visited their GP or hospital in the 2 years prior to diagnosis. An HIV test was performed in 59/407 (14.5%) GP visits. The median number of GP or hospital visits per person in the 2 years prior to diagnosis was 10 (IQR 2 to 21)). Among 32 with LP, five had presented with an AIDS‐defining condition (ADC) in the previous 2 years, five with an indicator condition (IC) strongly suggesting HIV testing and one with an indicator disease indicative of HIV testing [2] (Figure 1). One thousand eight hundred and forty people were diagnosed with hepatitis (59.6% male, median age 39, IQR 30 to 49). Thirty‐six (2.0%) presented late (28 with liver decompensation, six with hepatocellular carcinoma and two with both). Ninety‐eight percent had visited their GP or a hospital 2 years prior to diagnosis. A hepatitis test was performed in 195/1800 (10.8%) GP visits. The median number of GP or hospital visits per person in the 2 years prior to diagnosis was 18 (IQR 8 to 33). The most common reasons for a prior hospital visit were for radiological examination (N = 238, 12.9%) and observation for unspecified condition (N = 13, 0.7%).

**Conclusion: **A high percentage of people newly diagnosed with HIV or viral hepatitis have visited a GP or hospital 2 years prior to diagnosis without being tested. The results support European guidelines that call for increased normalisation of testing for HIV and viral hepatitis in health care settings to improve earlier diagnosis and entry into care for both HIV and hepatitis [3].

Abstract P038 – Figure 1. Previous diagnoses in those diagnosed HIV positive between 1 January 2013 and 31 December 2014.

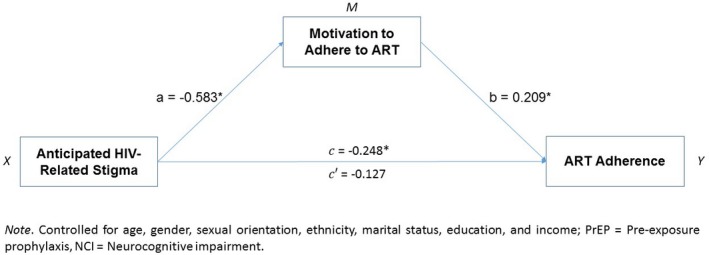

**References**

[1] Mocroft A. Late presentation to HIV/AIDS testing, treatment or continued care: clarifying the use of CD4 evaluation in the consensus definition. HIV Med. 2014;15:129.

[2] Sullivan AK, Raben D, Reekie J, Rayment M, Mocroft A, Esser S, et al. Feasibility and effectiveness of indicator condition guided testing for HIV: results from HIDES I (HIV Indicator Diseases across Europe Study). PLoS One. 2013;8:e52845.

[3] European Centre for Disease Prevention and Control. HIV testing: increasing uptake and effectiveness in the European Union. Stockholm: ECDC; 2010.

## P039

### Prevalence of chronic and acute HIV infection among febrile adults attending emergency departments in urban Tanzania

**N Boillat‐Blanco^1^, Z Mbarack^2^, J Samaka^3^, T Mlaganile^3^, A Mamin^4^, B Genton^1^, L Kaiser^4^ and V D'Acremont^5^**

^1^Infectious Diseases, University Hospital of Lausanne, Lausanne, Switzerland. ^2^Internal Medicine, Mwananyamala Hospital, Dar es Salaam, Tanzania. ^3^Epidemiology, Ifakara Health Institute, Dar es Salaam, Tanzania. ^4^Virology, University Hospital of Geneva, Geneva, Switzerland. ^5^Medicine, Swiss Tropical and Public Health Institute, Basel, Switzerland

**Background: **The World Health Organization recommends systematic HIV screening among patients seen in health facilities in countries with generalised HIV epidemics and to repeat testing 4 weeks later in the presence of a clinical indication of HIV infection. In resource‐limited settings, HIV screening is done by rapid diagnostic tests based on the detection of antibodies which will not allow to diagnose primary HIV infections that could be the reason of fever. We aimed to investigate the prevalence of chronic and acute HIV infections among outpatients with fever in urban Tanzania.

**Materials and methods: **We recruited consecutive adults with acute fever (tympanic temperature >38°C for ≤seven days) in emergency departments in Dar es Salaam between July 2013 and May 2014. Detailed medical history and clinical examination were done. Rapid diagnostic test for HIV was systematically performed and confirmed in case positivity following national recommendations. All patients with a negative HIV rapid test had an antigen p24 screening. Additional rapid, culture‐ and molecular‐based microbiological tests were performed according to pre‐defined algorithms to investigate the causes of fever.

**Results: **Among 519 patients with an acute febrile illness, HIV prevalence was 25% (N = 128; prevalence in adults in Dar es Salaam is 12%). Among these 128 patients, 122 (95%) had a positive rapid test which was confirmed by a second test. Six patients had only a positive p24 antigen suggesting an acute HIV infection in 1.2% of febrile adults: four presented with fever without focus, one having a rash and two concomitant dengue; one patient presented with a pharyngitis and one with a bronchitis. Among patients diagnosed with chronic HIV infection, 62% (N = 76) had an advanced disease (CD4 <200 cells/mm^3^) and 57% (N = 70) a newly diagnosed infection. Among those with an advanced disease, 63% were not previously known for HIV. Among patients with an advanced disease previously known for HIV (N = 28), 21% were diagnosed during the last three months, 13% three to twelve months ago and 67% for more than 1 year. Among these patients, 39% were not receiving antiretroviral therapy.

**Conclusions: **These results emphasise the need for systematic HIV screening among febrile adults attending emergency departments. They highlight the need to improve HIV screening as the majority of patients were newly diagnosed for HIV with an advanced disease. This study also shows that it is challenging to identify patients with primary HIV infection as the clinical picture is non‐specific and they can present with co‐infections.

## P040

### Evaluation of a training programme on HIV for primary care providers: impact on knowledge, barriers to HIV testing, screening rates and late diagnosis

**J Martínez‐Sanz^1^, A Muriel^2^, E Loza^3^, A Uranga^4^, C Gómez‐Ayerbe^1^, M Vivancos Gallego^1^, M Sánchez Conde^1^, M Calonge^4^, C Reyes Madridejos^5^, S del Campo Terrón^1^, A Sánchez^1^, M Merino Alejandre^6^, E Menéndez Alonso^7^, L Martínez Fuente^8^, M Seller Ripoll^9^, G Collada Holguera^10^, J Díaz Sánchez^11^, M Fuster^12^, M Galindo^13^ and M Pérez‐Elías^1^**

^1^Infectious Diseases, University Hospital Ramón y Cajal, Madrid, Spain. ^2^Clinical Biostatistics Unit, University Hospital Ramón y Cajal, Madrid, Spain. ^3^Microbiology, University Hospital Ramón y Cajal, Madrid, Spain. ^4^Primary Care, C.S. García Noblejas, Madrid, Spain. ^5^Primary Care, C.S. Benita de Ávila, Madrid, Spain. ^6^Primary Care, C.S. Alameda de Osuna, Madrid, Spain. ^7^Primary Care, C.S. Estrecho de Corea, Madrid, Spain. ^8^Primary Care, C.S. Silvano, Madrid, Spain. ^9^Primary Care, C.S. Virgen del Cortijo, Madrid, Spain. ^10^Primary Care, C.S. Rejas, Madrid, Spain. ^11^Primary Care, C.S. Barajas, Madrid, Spain. ^12^Sociedad Española Interdisciplinaria del SIDA, Madrid, Spain. ^13^Infectious Diseases, Hospital Clínico Universitario de Valencia, Valencia, Spain

**Background: **Adequate training of primary care providers (PCPs) is essential to prevent delayed HIV diagnosis [1,2]. We assessed PCPs’ knowledge and barriers to HIV testing before and after undertaking an education programme in the primary heath centres of the Basic Health Area of Ramón y Cajal Hospital (BHA‐RYCH), in Madrid (Spain). We assessed the influence of this training programme on HIV testing (HIV‐T), new HIV diagnoses (NHIVD) and late diagnosis (LD) rates.

**Materials and methods: **Pre‐experimental design based on a two‐hour training programme on HIV in the 20 health centres of the BHA‐RYCH. PCPs filled out the structured HIV testing questionnaire OptTEST before and after the intervention. We calculated global scores for both “knowledge” and “barriers” items (from 1 to 5 points). We assessed HIV‐T, NHIVD and LD rates in the six months before and after the training programme. Paired samples tests (Student's *t*‐test and Wilcoxon) were used to determine the differences between the pairs of observations. Effect sizes were estimated using Cohen's *d.*

**Results: **A total of 454 health professionals were trained, and 84 matched questionnaires were achieved. 91.7% of participants were women and the median age was 51 years. Fifty‐nine percent were medical doctors and 39% were nurses. After the training programme, the mean knowledge score was higher (*p* < 0.001), and the mean barriers score was lower (*p* = 0.002). The effect size was larger in knowledge (d = 0.89) than in barriers dimension (d = 0.32). We found statistically significant differences in 12 out of 14 individual items (Table 1). A median of 6226 patients were attended per health centre in both periods. There were 22 NHIVD (11 in each period). After finishing the training, we found a greater number of HIV‐T performed (*p* = 0.001), as well as a higher HIV‐T rate (*p* = 0.002) and a lower percentage of patients diagnosed with <200 CD4/mm^3^ (*p* = 0.092). We did not find differences between the two periods either in the number of NHIVD nor in the NHIVD rate.

**Table:** Abstract P040 – Table 1. Differences in the scores of each question before and after the intervention

	Pre‐intervention score (mean, SD)	Post‐intervention score (mean, SD)	*p* value
Knowledge			
People with undiagnosed HIV can be well with no symptoms for years	4.70 (0.53)	4.80 (0.56)	0.001
If diagnosed early HIV can be managed effectively with medication	4.69 (0.60)	4.92 (0.28)	0.011
HIV(+) on medication are less likely to transmit the infection	4.13 (1.08)	4.69 (0.69)	<0.001
It is important that people know their HIV status	4.81 (0.61)	4.93 (0.26)	0.199
HIV test should only be performed if patient asks for it	2.04 (0.99)	1.69 (0.91)	<0.001
HIV test should only be offered to people with high risk	1.93 (0.91)	1.65 (0.91)	0.007
A leaflet or brief pre‐test discussion is sufficient before offering HIV test	3.42 (1.11)	3.80 (1.33)	<0.001
Offering HIV test to people with indicator conditions is a good idea	4.13 (0.91)	4.77 (0.52)	0.003
Global Knowledge score (out of 100)	84.7 (7.9)	91.4 (7.3)	<0.001
Barriers			
I am concerned patients might ask questions I cannot answer	3.01 (1.13)	2.87 (1.21)	<0.001
I prefer that patients ask for test themselves	2.52 (0.83)	2.25 (0.97)	<0.001
I don't think that offering HIV test will be acceptable to patients	1.76 (0.73)	1.57 (0.75)	0.140
I would require additional training before offering HIV test	3.08 (1.31)	2.95 (1.23)	<0.001
I am comfortable discussing HIV testing with patients	3.73 (0.86)	3.94 (0.92)	<0.001
I am concerned that offering HIV testing will negatively affect patients’ opinion about our services	1.80 (0.84)	1.63 (0.85)	0.049
Global Barriers score (out of 100)	47.8 (11.7)	43.9 (12.9)	0.002

**Conclusions: **The educational programme for primary care health professionals achieves an improvement in the Knowledge score and a decrease in the Barriers score, assessed using the OptTEST questionnaire. After the training there is an increase in the number of HIV tests performed and a higher screening rate, as well as a lower percentage of late diagnosis. We did not find differences neither in the number of NHIVD nor in the NHIVD rate.

**References**

[1] Martínez Sanz J, Muriel A, Gómez‐Ayerbe C, Vivancos Gallego MJ, Sanchez Conde M, Pérez‐Elías P, et al. Higher knowledge on HIV predicted a better performance in HIV testing. DRIVE03 study [poster O‐0373]. 28th European Congress of Clinical Microbiology and Infectious Diseases; 2018 Apr 21‐24; Madrid, Spain.

[2] Kowalska JD, Perez‐Elias MJ, Lugo Colon R, Rae C, Jilich D, Sperle I, et al. Barriers in offering HIV testing in medical settings are likely to be addressed with short and targeted education ‐ data from the expanded staff questionnaire OptTEST project [poster PE25/21]. 16th European AIDS Conference; 2017 Oct 25‐27; Milan, Italy.

## P041

### Delayed linkage to HIV care among refugee late presenters in Montreal, Canada

**B Linthwaite, M Klein, B Lebouché, J Cox, C Frenette, C Costiniuk and N Kronfli**

Chronic Viral Illness Service, McGill University Health Centre, Montreal, Canada

**Background: **Refugees represent an increasing proportion of people living with HIV in Canada. To improve individual outcomes and prevent onward transmission, refugees should receive timely provision of comprehensive HIV care. Since 2002, the immigration medical examination (IME) includes mandatory HIV screening, with linkage to HIV care facilitated by immigration physicians. The McGill University Health Centre (MUHC) is the major HIV referral center for refugees in Quebec, Canada. We aimed to describe the trajectory from HIV screening to viral suppression for refugees referred to the MUHC for HIV care.

**Materials and methods: **We conducted a retrospective chart review of all refugees referred to the MUHC for HIV care over a 1‐year period starting 1 June 2017. The primary outcome measures were median time (in days with interquartile range [IQR]) from: (1) IME screening to notification of HIV diagnosis; (2) notification to linkage to HIV care (defined as either a CD4 cell count or viral load [VL] measure); (3) linkage to combination antiretroviral therapy (cART) prescription; and (4) cART prescription to first undetectable VL. Patients with missing data were excluded. Baseline sociodemographic and HIV‐related characteristics were assessed.

**Results: **Overall, 49% (50/102) of refugees were newly diagnosed in Canada (Table 1). Among these patients, 62% (31/50) were late presenters (CD4 <350 cells) and 22% (11/50) presented with advanced HIV (CD4 <200 cells); 24% (12/50) presented with high‐level viremia (VL >100,000 copies/mL). Over one‐third (38%) had baseline antiretroviral resistance to at least one drug class.  Among those previously diagnosed outside Canada, 31% (16/52) were late presenters and 15% (8/52) had advanced HIV; 31% (16/52) had detectable VLs at presentation. Opportunistic infections were rare among both groups. Among newly diagnosed refugees, time from IME screening to notification of diagnosis: 31 days [IQR 21 to 49]; notification to linkage to care: six days [IQR 1.5 to 17.5]; linkage to cART prescription: 11 days [IQR 5.5 to 16.5]; and cART prescription to first undetectable VL: 36 days [IQR 28 to 72]. Overall, 38% of newly diagnosed patients were linked to HIV care within 30 days, 74% within 60 days and 86% within 90 days from HIV screening. Median time from HIV screening to cART prescription was 62 days [IQR 46 to 89].

**Table:** Abstract P041 – Table 1. Characteristics of the study sample

			Overall (n = 102)	Diagnosed in Canada (n = 50)	Diagnosed outside Canada (n = 52)
Age (median [IQR])			37 [32 to 44]	37 [32 to 44]	37 [33 to 43]
Sex	Female		68 (67%)	27 (54%)	41 (78%)
	Male		34 (33%)	23 (46%)	11 (21%)
Country of origin	Africa	Nigeria	23 (23%)	9 (18%)	14 (27%)
		Other	32 (31%)	13 (26%)	19 (37%)
	Latin America	Haiti	45 (44%)	28 (56%)	17 (33%)
		Other	2 (2%)	0 (0%)	2 (4%)
CD4 at presentation in Canada, cells/µL (median, range, [IQR])			361, 11 to 1136, [229 to 407]	309, 11 to 811, [210 to 386]	446, 14 to 1136, [271 to 674]
	CD4 nadir <200		19 (19%)	11 (22%)	8 (15%)
	CD4 nadir <350		47 (46%)	31 (62%)	16 (31%)
Baseline viral load, copies/mL (median, range, [IQR])			3857, <20 ‐ >1 million, [<20 ‐ 41,368]	29,191, <20 ‐ > 1 million, [5558‐97,534]	<20, <20 ‐ > 1 million, [<20 ‐ 533]
OI at presentation			1 (1%)	1 (2%)	0 (0%)
Requiring primary prophylaxis			18 (18%)	9 (18%)	9 (17%)
cART regimens, most recent prescription	Single treatment regimens		84 (82%)	41 (82%)	43 (87%)
	3rd agent	NNRTI	3 (3%)	0 (0%)	3 (6%)
		PI	3 (3%)	0 (0%)	3 (6%)
		II	95 (93%)	49 (98%)	46 (88%)
Baseline ARV resistance	Yes		27 (26%)	19 (38%)	8 (15%)
	No		34 (33%)	26 (52%)	8 (15%)
	Unknown		41 (40%)	5 (10%)	36 (69%)
PPD	Positive	40 (39%)	16 (32%)	24 (46%)
	Negative		57 (56%)	33 (66%)	24 (46%)
	Not done		5 (5%)	1 (2%)	4 (8%)
Co‐infection with HBV			6 (6%)	4 (8%)	2 (4%)

II = integrase inhibitor; OI = opportunistic infection; PI = protease inhibitor; PPD = purified protein derivative.

**Conclusions: **While the majority (62%) of newly diagnosed refugees were late presenters, only 38% were linked to care within 30 days. Even in a system with a clear care pathway, there is a need to expedite referrals to HIV care following IME screening.

## P042

### A prospective randomised trial on abacavir/lamivudine plus darunavir/r or raltegravir in patients with CD4 +  <200 cells/uL (PRADAR study)

**C Mussini^1^, E Roncaglia^1^, L Sighinolfi^2^, S Nozza^3^, A Catellan^4^, P Bonfanti^5^, L Palvarini^6^, F Castelli^7^, A Di Biagio^8^ and F Maggiolo^9^**

^1^Clinic of Infectious Diseases, University of Modena and Reggio Emilia, Modena, Italy. ^2^Clinic of Infectious Diseases, Ferrara Hospital, Ferrara, Italy. ^3^Clinic of Infectious Diseases, San Raffaele Hospital, Milano, Italy. ^4^Clinic of Infectious Diseases, Padova Hospital, Padova, Italy. ^5^Infectious Diseases, Lecco Hospital, Lecco, Italy. ^6^Infectious Diseases, Mantova Hospital, Mantova, Italy. ^7^Clinic of Infectious Diseases, University of Brescia, Brescia, Italy. ^8^Clinic of Infectious Diseases, San Martino Hospital, Genova, Italy. ^9^Infectious Diseases, Bergamo Hospital, Bergamo, Italy

**Background: **HIV late presentation is still a major problem worldwide and patients represent a clinical challenge. Very few data are available on treatment in this population.

**Methods: **A prospective, multicentre, randomised open‐label, two‐arm, Phase III trial comparing the 48‐week virological response of two different regimens (abacavir/lamivudine + darunavir/ritonavir [DRV/r] vs. abacavir/lamivudine + raltegravir [RAL]) in antiretroviral‐naive, HIV+ individuals HLAB5701 negative, presenting for care with CD4 +  counts <200/mm^3^ and a viral load (VL) <500,000 copies/mL. Primary endpoint: proportion of patients with undetectable viraemia (VL <50 copies/mL) after 48 weeks. Secondary endpoints: change in CD4 +  cell count from baseline through Week 48 and time to virological rebound. The planned sample size for this trial was 350 patients.

**Results: **In 3 years 53 patients were screened and 46 enrolled: 22 randomised to RAL (19 males, median CD4 + 108 cells/uL, median VL 89,731 copies/mL) and 24 to DRV (CD4 + 117, VL 112,250). Seven patients were excluded, four because of a VL >500,000 copies/mL and three for HLAB5701 positivity. The snapshot analysis at 48 weeks showed a virological success of 77.3% in RAL and 66.7% in DRV. Time to starting treatment was 34.5 days in RAL and 53 days in DRV arms, respectively. At the as treated analyses the median increase in CD4 +  was 297 in RAL and 239 in DRV. No difference in total cholesterol, while triglycerides were higher in DRV arm. No statistical analyses were performed due to the low number of patients enrolled.

**Conclusions: **Patients: late presenters are frequent but very difficult to enrol in clinical trials. In these patients, the test and treat strategy is rarely applicable. The rate of virological success is similar to that described in the literature and very far from results of the recent trials in naive patients.

## P043

### Impact of a training project for primary health‐care providers (FOCO project) in the HIV screening and HIV late diagnosis

**M Pérez Elías^1^, G Sampériz^2^, D Dalmau^3^, A Romero^4^, B de la Fuente^5^, I de los Santos^6^, J Lopez^7^, P Arazo^2^, V Estrada^8^, F Lozano^9^, M Pastor^10^, A Ocampo^11^, A Arrillaga^12^, M Fuster‐Ruizdeapodaca^13^ and M Galindo^14^**

^1^Infectious Diseases, Hospital Ramón y Cajal, IRYCIS, Madrid, Spain. ^2^Infectious Diseases, Hospital Miguel Servet, Zaragoza, Spain. ^3^Infectious Diseases, Hospital Mutua Terrassa, Barcelona, Spain. ^4^Infectious Diseases, Hospital de Puerto Real, Cádiz, Spain. ^5^Infectious Diseases, Hospital Cabueñes, Gijón, Spain. ^6^Infectious Diseases, Hospital La Princesa, Madrid, Spain. ^7^Infectious Diseases, Hospital Gregorio Marañón, Madrid, Spain. ^8^Infectious Diseases, Hospital Clínico San Carlos, Madrid, Spain. ^9^Infectious Diseases, Hospital Valme, Sevilla, Spain. ^10^Management, Bizkaisida, Bilbao, Spain. ^11^Infectious Diseases, Hospital Alvaro Cunqueiro, Vigo, Spain. ^12^Coordinator, Plan del Sida del País Vasco, San Sebastian, Spain. ^13^Management, Sociedad Española Interdisciplinaria del SIDA (SEISIDA), Madrid, Spain. ^14^Infectious Diseases, Hospital Clínico Universitario, Valencia, Spain

**Background: **Reducing HIV late diagnosis remains an epidemiological challenge [1]. The objective of this project was to promote early HIV diagnosis through the training of primary health‐care providers (PHCP).

**Materials and methods: **HIV specialists conducted training sessions in 108 primary care centres (PCC) from six Spanish regions during 2016 and 2017, and with 1804 PHCP involved. The intervention was evaluated using a pre‐experimental design collecting the dependent variables both in the six months before and after the intervention. Number of requests for HIV tests from the PCC trained and clinical data of new HIV diagnosed patients were collected. Parametric and non‐parametric tests were used to assess differences between pre‐ and post‐intervention data.

**Results: **Number of HIV tests performed was higher after the intervention (16,833 vs. 19,793, *p* < 0.0001). Positive test results were 0.37% and 0.31% in the pre‐ and post‐intervention periods respectively. Clinical data of 132 HIV patients were collected (67 pre and 65 post). Percentage of lymphocytes CD4 mm^3^ were significantly higher (19.2 ± 12.7 vs. 24.6 ± 10, *p* = 0.011) and the median of absolutes lymphocytes was marginally higher (372 vs. 444, *p* = 0.083) after the intervention. A total of 44.4% versus 36.1% of the patients in the pre‐ and post‐intervention periods were diagnosed with <350 CD4 mm^3^. The number of AIDS‐related events was marginally lower after the intervention (*p* = 0.09). One patient who had visited six times PCC in the 2 previous years due to dermatological problems died two months after the diagnosis. There were no significant differences in the mean of visits to PCC during the 2 previous years to HIV diagnosis (4.2 ± 4.1 vs. 3.8 ± 3.2). In 34.3% and 38.5% of patients of pre‐ and post‐intervention periods, the pathologies because they visited PCC were related or suggestive of HIV. Data available in clinical records showed that while one patient visits PCC due to sexually transmitted infections in the pre‐intervention period, there were 12 patients in the post period. Previous negative HIV test was known in 44.8% and 33.8% of patients from both periods respectively. The pathologies of 42.3% of patients who had a previous HIV test more than 2 years were related to HIV, while they were in the 30.8% of the patients who had a previous HIV test of fewer than 2 years.

**Conclusions: **Training PHCP in the HIV screening and late diagnosis could be useful to increase HIV screening and to reduce late HIV diagnosis.

**Reference: **[1] Área de Vigilancia de VIH y Comportamientos de Riesgo. Vigilancia Epidemiológica del VIH y sida en España 2016: Sistema de Información sobre Nuevos Diagnósticos de VIH y Registro Nacional de Casos de Sida. Plan Nacional sobre el Sida ‐ S.G. de Promoción de la Salud y Epidemiología / Centro Nacional de Epidemiología ‐ ISCIII [Internet]. Madrid, Nov 2017. Available from: http://https://www.msssi.gob.es/ciudadanos/enfLesiones/enfTransmisibles/sida/vigilancia/InformeVIH_SIDA_2017_NOV2017.pdf.

## P044

### Late HIV diagnosis: identifying missed opportunities for HIV testing in North East England

**J Horsley Downie^1^, M Pegler^2^, A Price^1^, T Hardwick^1^, N Premchand^3^, J Widdrington^2^ and D Chadwick^2^**

^1^Dept of Infectious Disease & Tropical Medicine, Royal Victoria Infirmary, Newcastle Upon Tyne, UK. ^2^Centre for Clinical Infection, James Cook University Hospital, Middlesbrough, UK. ^3^Clinical Infection Service, Northumbria Specialist Emergency Care Hospital, Cramlington, UK

**Background: **Public Health England recorded 5164 new HIV diagnoses in 2016 of which 2066 (40%) were diagnosed late (CD4 <350) and 1084 (21%) very late (CD4 <200). The highest rates were found in the North of England with 47% of patients diagnosed late. BHIVA Standards of Care (2013 and 2018) recommend that HIV services should review all patients presenting with CD4 <200 or AIDS diagnosis in order to identify potential missed opportunities (MOs) for HIV testing that could have avoided late diagnosis.

**Method: **HIV treatment centres within North East England reviewed new HIV diagnoses from 2016 to 2018 in which patients had CD4 <350 or AIDS at diagnosis. Demographic data and details of potential MOs were collected from patient notes, prescribing information on the NHS Spine Summary Care Record and pathology results on the OpenNet WebICE system. The level of harm suffered by patients presenting late was determined using the National Reporting and Learning System (NRLS) grading system.

**Results: **Forty‐five patients were included across three centres, mean age 45 years, 76% male, 80% white and 33% MSM. Of these 16 (36%) had never previously had a HIV test but 34 (79%) had accessed healthcare in the past 3 years. Overall 37 patients (82%) suffered moderate or severe harm (NRLS Grade 2 to 5), 37 (82%) presented with a HIV indicator condition, 16 (36%) presented with AIDS and three (7%) died. On review 31 (69%) patients had one or more MO and 54 MOs were recorded in total, with a median of 21 months (IQR 5 to 47 months) from MO to presentation. MOs were most prevalent in GP surgeries with 28 (70%) of MOs occurring there. Twenty‐seven (87%) patients with an identified MO suffered moderate or severe harm due to the late HIV diagnosis, including 13 (76%) patients presenting with AIDS and two (67%) patients that died.

**Conclusion: **This study indicates that MOs for HIV testing can be identified in a high proportion of patients with late HIV diagnoses using a comprehensive review of hospital records, primary care prescriptions and pathology tests. In addition, we found that the majority of these late HIV presenters suffered moderate/severe harm; this harm was probably preventable in most patients with identified MOs for testing. The review mechanism that we have used has the potential to assist processes to systematically review late HIV diagnoses and identify interventions to reduce MOs for testing.

## P045

### Missed opportunities for earlier diagnosis of HIV in patients who presented with advanced HIV infection in a country where new HIV infection rate is rising, Turkey

**K Avcı, Z Yeşilbağ, S Şenoğlu, N Bold, Ö Altuntaş Aydın and H Kumbasar Karaosmanoğlu**

Infectious Diseases, Bakırköy Dr.Sadi Konuk Education and Research Hospital, İstanbul, Turkey

**Background: **Late detection of HIV infection decreases life expectancy, increases the rates of HIV transmission and treatment complexity. Unfortunately, many physicians are unaware of HIV diagnosis and testing. Our aim was to quantify and characterise missed opportunities for earlier HIV diagnosis in patients with diagnosed advanced HIV infection.

**Method: **This retrospective cohort study analysed the data of advanced late presenter HIV‐infected patients (CD4 cell count less than 200/mm^3^) with a new diagnosis, between January 2016 and May 2018. The primary endpoint was missed opportunities, the number of healthcare visits these patients made in the 3 years prior to being diagnosed with HIV infection. Information on demographics, such as age, sex,  transmission routes, initial CD4 counts, opportunistic infections and the numbers and types of healthcare visits prior to being diagnosed, were collected from medical and hospital records.

**Results: **Of the total 712 patients 90 (12.6%) were advanced late presenters. Of the advanced late presenters 75 (83%) were men and median age was 41 years. The 90 patients in the study had 190 healthcare visits during which an HIV test has not been performed. These visits were mostly missed by emergency medical doctors. The most common reason of applying for the healthcare service was dermatological complaints. In 90 patients, when the first HIV test was performed, 37 (41%) of them had AIDS‐related symptom, 16 (17%) of them were during pre‐operative test, 11 (12%) of their sexual partner was HIV infected, nine (10%) had routine checkups, four (4%) of them tested after unprotected sexual relationship, three (3%) had test during pre‐marriage test, two (2%) in blood donation, eight (8%) of the patients were not clear how they diagnosed. At the HIV infection diagnosis 29 patients had opportunistic infections, eight had AIDS‐related malignancies. The most common opportunistic infections were pulmonary tuberculosis and oropharyngeal/oesophageal candidiasis. Five of them died in fırst year of diagnosis.

**Conclusion: **The missed opportunities are the key to capturing the undiagnosed and unaware HIV‐positive individual. Being aware of clinical symptoms and physical exam presentations play key role in diagnosis of early HIV infections. However, late presenters are not always having AIDS‐related symptoms. Detectıng HIV infections early will increase patients’ life expectancy and quality. So, every country should be aware of their own epidemiological studies to build their own HIV testing guidelines.

## P046

### Long‐term survivors in a cohort of HIV+ patients diagnosed between 1985 and 1992: predictive factors associated with more than 25 years of survival

**L Anile, A Marino^1^, M Gussio^1^, M Locatelli^1^, A Pampaloni^1^, L Vinci^1^, D Scuderi^1^, B Busà^1^, R Bruno^1^, F Palermo^1^, B Cacopardo^1^ and B Celesia^1^**

^1^Unit of Infectious Diseases, ARNAS Garibaldi, Catania, Italy. ^2^Unit of Hospital Pharmacy, ARNAS Garibaldi, Catania, Italy

**Background: **HIV has radically changed the history of millions of women and men and dramatically reduced their expectancy and quality of life. Although generations of patients, diagnosed during the pre‐HAART era have been deprived of any chances to survive, a considerable number of them are still alive. Aim of this study was to evaluate the percentage of HIV long‐term survivors (LTS) (more than 25 years) in a cohort of HIV+ subjects diagnosed between 1985 and 1992 speculating on potential predictive factors associated to a so long survival.

**Materials and methods: **Single centre retrospective study. Data were collected from clinical files or historical databases. In accord with the protocol were considered epidemiological and clinical data collected at the time of HIV diagnosis. Longitudinal observation was stopped on 31 December 2017. Globally 186 subjects were enrolled; 148 (79.5%) males, all but one Caucasian, 100 (53.8%) IVDUs, 38 (20.4%) MSM, 33 (17.7%) heterosexuals. Median age 28 (IQR 24.6 to 34.5) years, median CD4 + 239 (IQR 56 to 477) cells/µL; 141 (76%) were late presenters, 58 (31.2%) AIDS presenters, 53% anti HCV+.

**Results: **Seventy‐two subjects (38.7%) were LTS, all but two actually alive, with a median survival of 350 (IQR 318 to 378) months. One hundred and four (55.9%) died before 25 years (NLTS) with a median survival of 37 (14 to 121) months. Finally 10 were untraceable and  excluded from survival analysis. Globally during the follow‐up 102 (54.8%) subjects were diagnosed with an AIDS‐defining illness (ADI). One hundred and one (57.4%) were treated with any antiretroviral treatment, all but four (55.1%) with any HAART regimen. The main cause of death (81.1%) was an HIV‐associated event. To be LTS versus NLTS was associated with some of the conditions registered at the time of diagnosis: female sex (29.1% vs. 14.4%, *p* = 0.022), median younger age [26 (22 to 29) vs. 29 (26 to 36), *p* < 0.0001], median CD4 cell number [397 (236 to 613) vs. 78 (31 to 280), *p* < 0.0001] and CD4/CD8 ratio [0.4 (0.27 to 0.73) vs. 0.24 (0.06 to 0.41), *p* = 0.024], HCV co‐infection (65.5% vs. 44%, *p* < 0.01), AIDS presentation (1.4% vs. 98.6%, *p* < 0.0001). Finally during the follow‐up respectively 75% and 100% of LTS were exposed to suboptimal ARV treatment or HAART versus 45% and 24% of NLTS (*p* < 0.0001) while 22.2% versus 82.7% developed an ADI.

**Conclusions: **More than one‐third of patients survived more than 25 years from diagnosis. Conditions traditionally associated with late presentation as male sex, older age, low CD4 and AIDS presentation are associated to bad prognosis. Access to treatment appear to be one of the principal driver of survival.

## TREATMENT STRATEGIES – TARGET POPULATIONS: NAÏVE PATIENTS

## P047

### Efficacy and safety of tenofovir disoproxil fumarate versus low‐dose stavudine over 96 weeks: a randomised, non‐inferiority trial

**F Venter^1^, A Hill^2^, A Kambugu^3^, M Chersich^1^, S Becker^4^, N Arulappan^1^, M Moorehouse^1^, M Majam^1^, G Akpomiemie^1^, S Sokhela^1^, S Poongulali^5^, C Feldman^1^, C Duncombe^6^, D Ripin^7^, A Vos^8^ and N Kumarasamy^5^**

^1^Wits Reproductive Health and HIV Institute, University of Witswatersrand, Johannesburg, South Africa^2^Translational Medicine, University of Liverpool, Liverpool, UK^3^Health Sciences, Makerere University, Kampala, Uganda^4^HIV/AIDS, Independent Consultant, Seattle, WA, USA^5^Voluntary Health Services, YRG Centre for AIDS Research and Education, Chennai, India^6^HIV/AIDS, Bill and Melinda Gates Foundation, Seattle, WA, USA^7^HIV Team, Clinton Health Access Initiative, Boston, MA, USA^8^Health Sciences, Utrecht University, Utrecht, Netherlands

**Background: **TDF/lamivudine (3TC)/efavirenz (EFV) is the current standard of care first‐line treatment in South Africa. This study was designed to determine whether dose reduction of stavudine (d4T) reduces drug toxicity, while preserving treatment efficacy, and to assess the safety profile of TDF/3TC/EFV in the control arm.

**Methods: **Phase IV, 96‐week, randomised, double‐blind, non‐inferiority trial in India, South Africa and Uganda. Methods: d4T 20 mg BD was compared with TDF, taken in combination with 3TC and EFV in 1072 HIV‐1‐infected treatment‐naive adults. There was no screening for drug resistance at baseline. The primary endpoint was the proportion of participants with HIV‐1 RNA <50 copies/mL. Adverse events assessments included measures of bone density and body fat. The trial is registered on Clinicaltrials.gov (NCT02670772).

**Results: **Between July 2012 and January 2014, 536 participants were recruited per arm. At Week 96, trial completion rates were 75.7% with d4T/3TC/EFV (*n* = 406) and 82.1% with TDF/3TC/EFV (*n* = 440, *p* = 0.011). Non‐completion was largely due to virological failure (6.2% [*n* = 33] with d4T/3TC/EFV vs 5.4% [*n* = 29] with TDF/3TC/EFV; *p* = 0.60). For the primary endpoint of HIV RNA suppression <50 copies/mL at Week 96, d4T/3TC/EFV was non‐inferior to TDF/3TC/EFV (79.3%, 425/536 vs 80.8%, 433/536; difference=‐1.49%, 95% CI ‐6.3 to 3.3). In a sub‐study, there was no correlation between drug resistance and the risk of virological failure. Drug‐related adverse event discontinuations were higher with d4T (6.7%, *n* = 36) than TDF (1.1%, *n* = 6; p<0.001). Lipodystrophy was more common in the d4T (5.6%) than TDF arm (0.2%). Drug‐related adverse event discontinuations were higher with d4T (6.7%) than TDF (1.1%). Details of the most common adverse events are shown in Table 1. Creatinine clearance increased in both arms, by 18.1 mL/min in the d4T arm and 14.2 mL/min with TDF (*p* = 0.03). Bone density, however, showed greater loss at hip with TDF.

**Table:** Abstract P047 – Table 1. Percentage of patients with Grade 1–4 adverse events by treatment arm

Adverse events	d4T/3TC/EFV, *n* = 533	TDF/3TC/EFV, *n* = 534
Discontinuation for adverse events	52 (9.8%)	19 (3.6%)
Grade 1‐4 all cause adverse events		
Dizziness	176 (33.3%)	175 (32.8%)
Peripheral neuropathy	37 (6.9%)	24 (4.5%)
Lipodystrophy	30 (5.6%)	1 (0.2%)
Nausea	20 (3.8%)	25 (4.7%)
Somnolence	27 (5.1%)	24 (4.5%)
Vomiting	20 (3.8%)	31 (5.8%)
Aesthenia	21 (3.9%)	11 (2.1%)
Diarrhoea	32 (6.0%)	35 (6.6%)
Pyrexia	26 (4.9%)	12 (2.2%)
Urinary tract infection	69 (13.0%)	91 (18.5%)
Respiratory tract infections	104 (19.5%)	119 (22.3%)

**Conclusions: **In this 96‐week randomised trial in 1073 treatment‐naïve patients, low‐dose d4T combined with 3TC/EFV demonstrated non‐inferior virological efficacy compared to TDF/3TC/EFV, but with a significantly higher risk of lipoatrophy. Little renal toxicity was noted in either arm.

## P048

### Carotid wall thickness evolution after 2 years of first‐line therapy with dolutegravir/abacavir/lamivudine or elvitegravir/cobicistat/emtricitabine

**I Gallazzi, S Restelli, M Piscaglia, M Schiavini, M Cossu, L Paladini, G Rizzardini and A Capetti**

1st Division of Infectious Diseases, ASST Fatebenefratelli Sacco, Milano, Italy

**Background: **The effect of integrase inhibitor (INSTI)‐based regimens on the carotid wall thickness has not been widely investigated [1]. Apart from the ACTG A5260s, comparing raltegravir, darunavir and atazanavir, and a substudy of the NEAT022 [2] on the switch from protease inhibitors to dolutegravir, nothing has been presented to date on the international arena.

**Materials and methods: **All subjects initiating a first‐line regimen with dolutegravir/abacavir/lamivudine (D/A/L, Triumeq^TM^) or elvitegravir/cobicistat/emtricitabine (E/C/F/T, Stribild^TM^) between 1 January 2015 and 1 January 2016 have been retrospectively investigated, acquiring from the patients’ record forms demographic and clinical data. Those who had undergone carotid Doppler ultrasound at baseline and within 96 to 120 weeks were analysed through the two‐tailed paired Student's t‐test for intra‐patient variations and through the unpaired t‐test for comparison between the two groups. The exam was performed with a high‐resolution B‐mode linear scanner measuring three points within 1 cm below the carotid bifurcation, on the common carotid artery (CCA) wall. Inflammatory and atherogenic biomarkers were also analysed.

**Results: **Of 84 subjects taking D/A/L and 69 taking E/C/F/T, 22 and 20 had carotid ultrasound performed in window. The two populations differed for higher proportions in the E/C/F/T group of history of drug addiction, CDC stage C, more comorbidities, more cardiovascular agents and statin intake, but lower baseline serum glucose. The intima–media thickening at 2 years was comparable between the two groups, much lower than described in the ACTG A5260s. The D/A/L arm had sharper HIV‐1 suppression (88.1% vs. 56.6% with no virus detected at Week 96, 92.9% and 95.7%, respectively, having achieved <50 copies/mL), better reduction of inflammation, in terms of hs‐RCP, while the E/C/F/T arm had better impact on Apo‐A1, probably related to the activity of tenofovir. The main results are displayed in Table 1.

**Table:** Abstract P048 – Table 1. Main metabolic results at Week 96 in the CARINSTI study

D/A/L, n = 22	IMT CCA 1st point	IMT CCA 2nd point	IMT CCA 3rd point	Homocistein	hs‐RCP	Apo‐A1	Apo‐B
Mean±SD							
Baseline	0.625 ± 0.17	0.58 ± 0.16	0.575 ± 0.16	7.15 ± 2.95	1.7 ± 1.36	1.47 ± 0.12	1.3 ± 0.34
Week 96	0.63 ± 0.17	0.588 ± 0.15	0.585 ± 0.15	7.05 ± 2.36	1.4 ± 1.03	1.52 ± 0.09	1.23 ± 0.34
D	0.005	0.008	0.010	−0.1	−0.3	0.05	−0.07
E/C/F/T, n = 20	IMT CCA 1st point	IMT CCA 2nd point	IMT CCA 3rd point	Homocistein	hs‐RCP	Apo‐A1	Apo‐B
Mean±SD							
Baseline	0.5 ± 0.13	0.6 ± 0.16	0.51 ± 0.16	6.1 ± 4.08	1.7 ± 1.28	1.2 ± 0.29	1.41 ± 0.44
Week 96	0.5 ± 0.16	0.605 ± 0.16	0.52 ± 0.15	6.7 ± 2.23	2.1 ± 0.94	1.29 ± 0.42	1.38 ± 0.43
D	0	0.005	0.010	0.6	0.4	0.09	−0.03
Paired *t*‐test for D D/A/L	0.473	0.429	0.381	0.8	<0.0001	0.176	0.066
Paired *t*‐test for D E/C/F/T	1	0.475	0.384	0.117	<0.0001	0.017	0.245
Unpaired t‐test for D D/A/L vs E/C/F/T							
D	0.005	0.003	0	−0.7	−0.7	−0.04	−0.04
P	0.462	0.639	1	<0.0001	<0.0001	<0.0001	<0.0001

**Conclusions: **Both INSTI‐based regimens showed optimal viral suppression, good tolerability and a favourable impact on carotid intima–media thickness, which evolved within the physiological age‐adjusted rate [3].

**References**

[1] Stein JH, Ribaudo HJ, Hodis HN, Brown TT, Tran TT, Yan M, et al. A prospective, randomized clinical trial of antiretroviral therapies on carotid wall thickness. AIDS. 2015;29:1775‐83.

[2] Martinez E, Assoumou L, Camafort M, Domenech M, Guaraldi G, Domingo P, et al. Switching from boosted protease inhibitors (PI/r) to dolutegravir (DTG) in virologically suppressed HIV‐infected patients with high cardiovascular risk: 48‐week effects on subclinical cardiovascular disease [abstract 2/6]. 16th European AIDS Conference; 2017 Oct 25‐27; Milan, Italy.

[3] Juonala M, Viikari JS, Kähönen M, Taittonen L, Laitinen T, Hutri‐Kähönen N, et al. Life‐time risk factors and progression of carotid atherosclerosis in young adults: the Cardiovascular Risk in Young Finns study. Eur Heart J. 2010;31:1745‐51.

## P049

### High levels of patient satisfaction during rapidly initiated therapy with darunavir/cobicistat/emtricitabine/tenofovir alafenamide (D/C/F/TAF) for treatment of HIV‐1 infection through 24 weeks of the DIAMOND study

**C Benson^1^, R Simonson^1^, C Bicer^2^ and K Dunn^1^**

^1^Janssen Scientific Affairs, Titusville, NJ, USA. ^2^BICER Consulting & Research, Beerse, Belgium

**Background: **Some patients or clinicians are hesitant to rapidly start ART due to concerns regarding side effects, dosing requirements and need for life‐long therapy. Patient‐reported outcomes (PROs) are not currently available for any ART regimen in rapid initiation scenarios. We report PROs of the D/C/F/TAF single‐tablet regimen (STR) in a rapid initiation scenario.

**Materials and methods: **DIAMOND (ClinicalTrials.gov: NCT03227861) is an ongoing, Phase III, single‐arm, open‐label, prospective, multicenter study assessing the efficacy and safety of D/C/F/TAF 800/150/200/10 mg in a rapid initiation scenario over 48 weeks. Adults newly diagnosed with HIV‐1 infection within 14 days of screening/baseline were immediately enrolled and started on D/C/F/TAF without screening/baseline laboratory information. Screening/baseline laboratory results were reviewed as results became available; patients not meeting predefined safety or resistance stopping rules continued treatment. PROs were assessed using the validated 10‐item HIV Treatment Satisfaction Questionnaire (HIVTSQs) six‐point ordinal scale (v.2006) at Weeks 4, 24 and 48, or time of early study treatment discontinuation (ESTD). An interim analysis was conducted at Week 24 to assess safety and efficacy. HIVTSQs scores were analyzed according to total score, clinical subscale and individual items.

**Results: **Baseline demographics are listed in Table 1. At the interim analysis, 90.8% (99/109) of patients continued D/C/F/TAF and 10 discontinued (three due to safety stopping rules, one protocol violation, one adverse event, two withdrawals of consent and three lost to follow‐up). In an ITT‐FDA Snapshot analysis, 80.7% of patients achieved virologic suppression at Week 24. The mean HIVTSQs total score was 56.5 (n = 103) and 57.9 (n = 98) (out of a total of 60) at Week 4 and 24, respectively. Subscale scores were also high at both timepoints for patients continuing (Figure 1). Four patients completed the questionnaire at ESTD: three prior to Week 2 due to predefined protocol stopping rules, and one patient withdrew consent at Week 36. The mean HIVTSQs total score for these patients was 49.3.

**Table:** Abstract P049 – Table 1. Baseline demographic characteristics

Parameter	N = 109
Age, median (range), years	28 (19 to 66)
Women, %	13
African American, %	32
HIV‐1 RNA, median (range), log10 copies/mL	4.6 (1.3 to 8.2)
HIV‐1 RNA ≥100,000 copies/mL, %	24
CD4 + count, median (range), cells/mm^3^	369 (7 to 1082)
CD4 + count <200 cells/mm^3^, %	21
Time from diagnosis to screening/baseline, median (range), days	5 (0 to 14)
Enrolled within 48 hours of diagnosis, %	29

Abstract P049 – Figure 1. HIVTSQs scores by timepoint.*

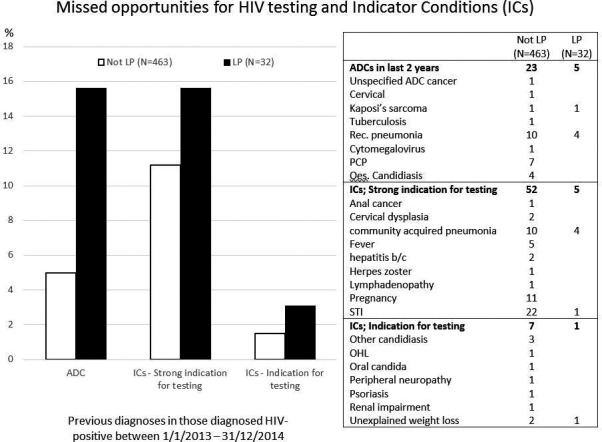

**Conclusions: **Patients rapidly starting and continuing D/C/F/TAF demonstrated high satisfaction scores. Patients who discontinued demonstrated lower scores; however, in three of four patients, exposure to study drug was brief and discontinuation was mandated by protocol design rather than patient preference. As regimens used in rapid initiation should optimally be an STR, abacavir‐sparing, have a high genetic barrier to resistance, be well tolerated and have demonstrated high satisfaction scores, D/C/F/TAF should be considered a preferred treatment for rapid initiation.

## P050

### Safety and efficacy of doravirine/lamivudine/tenofovir disoproxil fumarate (DOR/3TC/TDF) in treatment‐naïve HIV‐1 infected adults with transmitted NNRTI resistance mutations

**A Wong^1^, D Goldstein^2^, J Mallolas^3^, E DeJesus^4^, M Johnson^5^, J Molina^6^, A Pozniak^7^, A Rodgers^8^, V Teal^8^, D Hepler^8^, S Kumar^8^, P Sklar^8^, G Hanna^8^, C Hwang^8^, C Badshah^8^ and H Teppler^8^**

^1^Department of Medicine, University of Saskatchewan, Regina, Canada. ^2^Department of Clinical Research, Whitman‐Walker Institute, Washington, DC, USA. ^3^Hospital Clinic‐IDIBAPS, University of Barcelona, Barcelona, Spain. ^4^Department of Clinical Research, Orlando Immunology Center, Orlando, FL, USA. ^5^Department of HIV Medicine, Royal Free Hospital, London, UK. ^6^Hôpital Saint‐Louis, University of Paris Diderot, Paris, France. ^7^NHS Foundation Trust, Chelsea and Westminster Hospital, London, UK. ^8^Research Laboratories, Merck & Co., Inc., Kenilworth, NJ, USA

**Background: **Doravirine is a novel NNRTI with in‐vitro activity against HIV‐1 variants with most commonly transmitted NNRTI mutations. This trial was designed to evaluate the safety and efficacy of DOR/3TC/TDF, a single‐tablet regimen of doravirine 100 mg, lamivudine 300 mg and tenofovir disoproxil fumarate 300 mg, in treatment‐naive adults with HIV‐1 infection and transmitted NNRTI resistance.

**Materials and methods: **Phase II, multicenter, open‐label, single‐arm trial of DOR/3TC/TDF once daily. Participants were adults with screening HIV‐1 RNA ≥1000 copies/mL, CD4+ T‐cell count ≥100/mm^3^, no prior antiretroviral treatment for HIV‐1, a single NNRTI resistance mutation (RT K103N, Y181C or G190A) and no genotypic resistance to doravirine, lamivudine or tenofovir. The primary endpoint was the proportion of participants achieving HIV‐1 RNA <50 copies/mL at Week 48. Enrollment was halted due to projected inability to recruit the planned 60 participants in a reasonable time period. Due to the limited sample size, the efficacy analyses included observed data only; missing data were not imputed.

**Results: **Of the 18 participants screened, 10 (eight with K103N, two with G190A) were enrolled and treated; the median age was 32.5 years, and most participants (8/10) were male. At baseline, the median CD4+ T‐cell count was 408/mm^3^ (range 213 to 607) and the median HIV‐1 RNA level was 17,281 copies/mL (range 1366 to 295,604). Two participants discontinued before Week 48: one with G190A met criteria for virologic failure (rebound after suppression <50 copies/mL) at Week 24 due to treatment non‐adherence, and one with K103N was lost to follow‐up after Week 16 (and was excluded from the efficacy analysis because the baseline mutation could not be confirmed). HIV‐1 RNA <50 copies/mL was achieved in all eight participants who completed Week 48, and the mean increase from baseline in CD4+ T‐cell count was 132/mm^3^. No participant developed additional resistance mutations during the trial, including the participant with virologic rebound after non‐adherence. Six participants reported adverse events that were judged by the investigator to be related to study therapy; all of these events were non‐serious. One participant had a serious adverse event (allergy to arthropod sting) that was not drug related. None of the adverse events led to treatment discontinuation.

**Conclusions: **Although the targeted enrollment was not reached, a favorable efficacy response was observed at Week 48 in participants with selected high‐prevalence NNRTI mutations. DOR/3TC/TDF was generally well tolerated in this population.

## P051

### Success and failure of initial ART in adults: an updated systematic review including 77,999 subjects from 1994 to 2017

**A Carr^1^, R Richardson^2^ and Z Liu^3^**

^1^HIV, Immunology and Infectious Diseases Unit, St Vincent's Hospital, Sydney, Australia. ^2^Centre for Applied Medical Research, St Vincent's Hospital, Sydney, Australia. ^3^Stats Central, University of New South Wales, Sydney, Australia

**Background: **We updated a prior (1994 to 2012) systematic review of adult initial ART efficacy through Week 144.

**Materials and methods: **Studies (1/2013 to 7/2017) were drawn from PubMed, ClinicalTrials.gov, Cochrane Library and major HIV conferences; study design, eligibility, subject and ART data were abstracted. Summary measures are expressed as group size‐weighted means. Mixed‐effects, meta‐regression was used to identify sources of efficacy heterogeneity.

**Results: **We analysed 354 groups (181 studies, 77,999 subjects [37,875 new]): baseline mean age 36.9 years, 74.7% men, 61.0% white, CD4 262 cells/mm^3^, HIV viral load 4.8 logs. Subjects took 4.8 pills (including placebo) in 1.6 doses/day. Principal backbones were tenofovir (TDF/TAF)/emtricitabine (44.2%), thymidine‐based (27.7%) and abacavir‐lamivudine (9.7%). Principal anchors were non‐nucleoside analogue (49.7%), boosted protease inhibitor (28.1%) and integrase inhibitor (INSTI; 11.5%). Data were highly heterogeneous (*I2 *= 96.1%). Mean ITT efficacy (RNA < 50 copies/mL) was 71.3%, 63.5% (145 groups) and 61.8% (48 groups) at Weeks 48, 96 and 144, respectively. Week 48 efficacy increased substantially over time (*p* < 0.0001). For post‐2010 studies, Week 48, 96 and 144 efficacy was 83.8%, 79.9% and 77.1%, respectively. Independent predictors of greater efficacy at Week 48 were pre‐ART resistance genotyping (vs. none: adjusted difference 4.3% [1.4 to 7.2], *p *= 0.0003); higher baseline CD4 counts (per 100 CD4‐cell increment: 2.2% [1.0 to 3.4], *p *= 0.0003); once‐daily ART (vs. two doses/day: 3.4% [0.9 to 5.9], *p *= 0.008); INSTI‐based ART (vs. other anchor classes: *p* < 0.001); and ART including TDF/TAF‐FTC (vs. other nucleosides, *p *= 0.02). Independent predictors of greater efficacy at both Weeks 96 and 144 were INSTI‐based ART (*p* ≤ 0.003), TDF/TAF‐FTC‐based ART (*p *= 0.0003) and fewer ART pills per day (*p *= 0.001), although TDF/TAF‐FTC was not superior to ABC‐3TC after exclusion of studies that did not incorporate HLA‐B*5701 screening. Phase III studies yielded progressively superior efficacy to Phase IV studies at Week 48 (adjusted difference 5.1%, *p *= 0.003), Week 96 (11.5%, *p *= 0.0004) and Week 144 (15.8%, *p *= 0.001). ART cessation at Weeks 48, 96 and 144 was 20.5%, 26.9% and 29.4%, respectively. Cessation by Week 144 overall and for adverse events (8.9%) declined substantially over time, but cessation for virological failure (5.2%) did not.

**Conclusions: **Initial ART efficacy continues to improve, but >20% of post‐2010 subjects failed over 3 years. Few demographic factors predicted failure. Phase III trials yield overly optimistic impression of real‐world efficacy. All guidelines should list INSTI‐based initial ART as preferred. Strategies are needed to improve access to pre‐ART genotyping and to increase early initiation of once‐daily ART.

## P052

### Effectiveness, persistence and safety of E/C/F/TAF, F/TAF+3rd agent or R/F/TAF use in treatment‐naive HIV‐1 infected patients: 12‐month results from the German TAFNES cohort study

**T Heuchel^1^, H Hillenbrand^2^, H Jessen^3^, R Pauli^4^, N Postel^5^, R Haubrich^6^, M Heinzkill^7^, K Goerner^7^ and H Stellbrink^8^**

^1^Clinical Care, Praxis Heuchel, Chemnitz, Germany. ^2^Clinical Care, MVZ Praxis City Ost, Berlin, Germany. ^3^Clinical Care, Gemeinschaftspraxis Jessen, Berlin, Germany. ^4^Clinical Care, Gemeinschaftspraxis Becker/Pauli, Munich, Germany. ^5^Clinical Care, Prinzmed Private Practice, Munich, Germany. ^6^Clinical Science, Gilead Sciences, Foster City, CA, USA. ^7^Clinical Science, Gilead Sciences, Munich, Germany. ^8^Clinical Care, ICH Study Center, Hamburg, Germany

**Background: **Based on controlled clinical trials, tenofovir alafenamide (TAF)‐based regimens are among recommended regimens for first‐ and further‐line ART of HIV infection in Germany. To evaluate the effectiveness and safety of TAF‐based single‐tablet (STR) or multi‐tablet regimens (MTR) when used in treatment‐naïve (TN) or treatment‐experienced adult HIV‐infected patients in a real‐life setting, the non‐interventional 24‐month prospective TAFNES cohort study was initiated. 

**Methods: **Month‐12 (M12) evaluation of using TAF‐based regimens, i.e. E/C/F/TAF, F/TAF+3rd agent or R/F/TAF, in TN patients of the TAFNES cohort. The analysis population consisted of patients starting treatment at least 9 months prior to data‐cut (May 2018). Outcome measures included ART persistence (using Kaplan‐Meier analyses), virological effectiveness (HIV‐RNA < 50 copies/mL, modified ITT‐analyses (mITT), discontinuation* *= failure, loss‐to‐follow‐up and missing* *= excluded), incident serious/non‐serious adverse drug reactions (SADRs/ADRs) and health‐related quality of life (HRQoL) using validated questionnaires, namely the SF‐36 and the HIV Symptom Index (HIV‐SI).

**Results: **Of 239 TN patients (94% men, median age 36 years) who were eligible for analysis, 143 patients initiated E/C/F/TAF, 65 on F/TAF+3rd agent (85% dolutegravir, 5% raltegravir, 10% other) and 31 on R/F/TAF. Late presentation was particularly common in the E/C/F/TAF and F/TAF+3rd agent groups (Table 1). 13.4% (n* *= 32/239) of patients discontinued the study before M12 visit, after a median treatment time of 21 weeks (including discontinuation of F/TAF‐based regimens due to ADRs (1.7%), drug‐drug‐interaction (1.7%), virological failure (0.8%), patient decision (0.8%) or other reasons (1.3%) and loss‐to‐follow‐up (7.1%)). There was no significant difference in persistence between treatment groups (Figure 1). At M12 visit, 86.9% (n* *= 185/213) of patients had HIV‐RNA levels <50 copies/mL (mITT); 88.3% of patients treated with E/C/F/TAF (n* *= 113/128), 83.9% of patients treated with F/TAF+3rd agent (n* *= 47/56) and 86.2% of patients treated with R/F/TAF (n* *= 25/29). By M12, 19 ADRs in 11 patients (5%) and two SADRs in two patients (0.8%; both diarrhoea while on F/TAF+3rd agent) were documented. Overall HRQoL outcomes indicated significant improvements in symptom distress and in the mental and physical components of the SF‐36.

**Table:** Abstract P052 – Table 1. Baseline characteristics and reason for discontinuation

Overall	E/C/F/TAF	F/TAF+3rd agent	R/F/TAF
N (%)	239 (100)	143 (60)	65 (27)	31 (13)
Male gender, n (%)	225 (94)	136 (95)	61 (94)	28 (90)
Age, years, median (IQR)	36 (30 to 46)	36 (31 to 46)	39 (30 to 47)	36 (30 to 43)
CDC stage C, n (%)	20 (8)	10 (7)	9 (14)	1 (3)
Late presentation^a^	80 (34)	45 (32)	31 (49)	4 (13)
CD4 count, cells/µL, median (IQR)	462 (282 to 629)	494 (310 to 629)	353 (161 to 582)	504 (428 to 642)
CD4 <200 cells/µL, n (%)	36 (15)	18 (13)	18 (29)	0 (0)
HIV‐RNA, log copies/mL, median (IQR)	4.4 (3.9 to 5.1)	4.3 (3.8 to 4.9)	5.1 (4.3 to 5.6)	4.2 (3.7 to 4.5)
HIV‐1 RNA >100,000 copies/mL, n (%)	67 (28)	31 (22)	36 (55)	0 (0)
Reason for discontinuation, n (%)				
ADRs	4 (1.7)	2 (1.4)	2 (3.1)	0 (0.0)
Drug‐drug interaction	4 (1.7)	3 (2.1)	0 (0.0)	1 (3.2)
Virological failure	2 (0.8)	1 (0.7)	0 (0.0)	1 (3.2)
Patient decision	2 (0.8)	0 (0.0)	2 (3.1)	0 (0.0)
Other	3 (1.3)	1 (0.7)	2 (3.1)	0 (0.0)
Lost to follow‐up	17 (7.1)	8 (5.6)	7 (10.8)	2 (6.5)

^a^defined as CD4 cell count <350 cells/µL and/or CDC stage C (AIDS). IQR, interquartile range.

Abstract P052 – Figure 1.** **Kaplan‐Meier analyses ‐ Time on study drug for E/C/F/TAF, F/TAF+3rd agent and R/F/TAF.

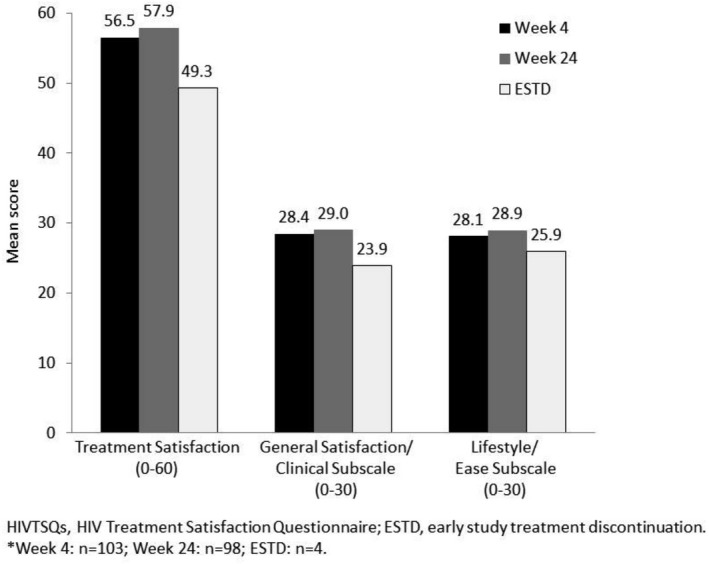

**Conclusion: **F/TAF‐based regimens for initial ART showed good persistence in this observational cohort of treatment‐naïve adults. Low discontinuation rates due to ADRs (<2%) and significant improvements in HRQoL confirm the safety of using F/TAF in clinical routine care. Evaluation of SF‐36 and HIV‐SI indicated significant improvements in mental and physical HRQoL, as well as in symptom distress.

## P053

### Adverse outcomes of first‐line integrase inhibitor‐based single‐tablet antiretroviral regimens in the Spanish VACH cohort

**R Teira^1^, E Deig^2^, T Puig^3^, P Galindo^4^, L Merino^5^, V Estrada^6^, E Ribera^7^, M Sepúlveda^8^, J García^9^, M Montero^10^, P Muñoz‐Sánchez^11^, J Peraire^12^, M Castaño^13^, N Espinosa^14^, E Martínez^15^, F Téllez^16^, B de la Fuente^17^, A Terrón^18^, F Lozano^19^, B Roca^20^, A Muñoz‐Sanz^21^ and P Geijo^22^**

^1^Internal Medicine, Hospital de Sierrallana, Torrelavega, Spain. ^2^Internal Medicine, Hospital General, Granollers, Spain. ^3^Internal Medicine, Hospital Arnau de Vilanova, Lleida, Spain. ^4^Infectious Diseases, Hospital Clínico, Valencia, Spain. ^5^Infectious Diseases, Hospital Infanta Elena, Huelva, Spain. ^6^Infectious Diseases, Hospital Clínico de San Carlos, Madrid, Spain. ^7^Infectious Diseases, Hospital Vall d´Hebrón, Barcelona, Spain. ^8^Internal Medicine, Hospital Virgen de la Salud, Toledo, Spain. ^9^Internal Medicine, Hospital Santa Lucía, Cartagena, Spain. ^10^Infectious Diseases, Hospital La Fe, Valencia, Spain. ^11^Infectious Diseases, Hospital de Basurto, Bilbao, Spain. ^12^Infectious Diseases, Hospital Joan XXIII, Tarragona, Spain. ^13^Infectious Diseases, Hospital Carlos Haya, Málaga, Spain. ^14^Infectious Diseases, Hospital Virgen del Rocío, Sevilla, Spain. ^15^Infectious Diseases, Complejo Hospitalario, Albacete, Spain. ^16^Infectious Diseases, Hospital Clínico, Puerto Real, Spain. ^17^Infectious Diseases, Hospital de Cabueñes, Gijón, Spain. ^18^Internal Medicine, Hospital del SAS, Jerez de la Frontera, Spain. ^19^Infectious Diseases, Hospital de Valme, Sevilla, Spain. ^20^Internal Medicine, Hospital General, Castellón, Spain. ^21^Internal Medicine, Hospital Infanta Cristina, Badajoz, Spain. ^22^Internal Medicine, Hospital Virgen de la Luz, Cuenca, Spain

**Background: **Coformulations of cobicistat‐boosted elvitegravir plus emtricitabine and tenofovir‐alafenamide (Genvoya^®^) and dolutegravir plus lamivudine and abacavir (Triumeq^®^) are the only preferred ART regimes available in a single tablet, but they have not been directly compared neither are there registered plans to do so.

**Methods: **From the Spanish VACH cohort database we selected those patients who started first‐line ART with Genvoya or Triumeq and who had at least 1 year of follow‐up and one additional visit to the clinic. We studied their adverse outcomes (AOs), which we defined as: 1) any treatment change, 2) losses to follow‐up, 3) AIDS or death more than 30 days after initiation. We studied also viral loads at the end of follow‐up. We constructed Kaplan‐Meier curves of time until AO for each regimen and Cox regression models to control for differences in other prognostic variables.

**Results: **Out of 1233 patients who had started ART with Genvoya or Triumeq, 807 fufilled the other selection criteria. Three hundred and forty‐three received Genvoya and 464 Triumeq. There were 683 males and 124 females. Sexual transmission of HIV was presumed for 690 (486 between males) patients, drug use for 42 and other or an unknown mechanism for an additional 75. Median age, log10 baseline viral load and nadir CD4 cell counts were, repectively, 36, 4.49 and 394. During follow‐up 130 patients experienced an AO: 56 of those starting with Genvoya and 74 with Triumeq. Figure 1 shows the Kaplan‐Meier curves of time until an AO (*p *= 0.244, log‐rank test). Nadir CD4 cell count, baseline (log10) viral load and mechanism of HIV transmission were independently associated with outcomes, but not sex, age, treatment regimen or ethnics.

Abstract P053 – Figure 1. Kaplan‐Meier curves of time until an adverse outcome, by treatment group.

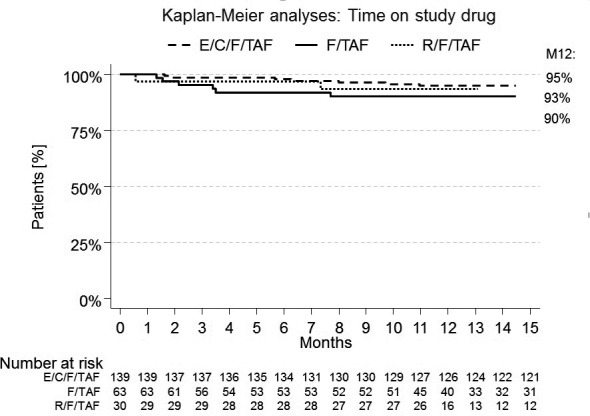

**Conclusions: **We observed non‐significant differences in AO between preferred first‐line, integrase inhibitor‐based, single‐tablet regimens for HIV infection (Genvoya and Triumeq) in the real‐life setting of the Spanish VACH cohort.

## P054

### Comparative efficacy of dolutegravir relative to common core agents in treatment‐naïve HIV‐1‐infected patients: a systematic review and network meta‐analysis

**S Snedecor^1^, T Inocencio^1^, R Grove^2^, M Radford^3^ and Y Punekar^3^**

^1^Health Economics Outcomes Research, Pharmerit International, Bethesda, MD, USA. ^2^Statistics, ViiV Healthcare, Brentford, UK. ^3^Health Outcomes, ViiV Healthcare, Brentford, UK

**Objective: **Data from randomised controlled trials (RCTs) provide comparative estimates of efficacy and safety for one treatment versus another (or placebo). However, not all desired comparisons of interest in clinical practice have been studied in head‐to‐head RCTs. This study compares the efficacy of core agent antiretrovirals commonly used in clinical practice for treatment‐naïve HIV‐1‐infected patients.

**Methods: **A systematic review identified Phase III RCTs (up to September 2017) of treatment‐naïve HIV‐1 patients treated with one of the core agent ARTs of interest, based on DHHS and EACS treatment guidelines: boosted PIs (ATV/r, DRV/r, LPV/r), NNRTIs (EFV, RPV) and INSTIs (DTG, RAL, EVG/c, BIC). Efficacy outcomes analysed were viral suppression (VS) and CD4 change from baseline at 48 weeks. Bayesian network meta‐analysis methodology was used to compare efficacy outcomes of the nine core agents of interest. The analysis examined fixed effects and random effects models both with and without adjustment for different NRTI treatment backbones (TD(A)F/FTC, ABC/3TC or other). Scenario analyses using an enlarged network of RCTs comparing one of the core agents of interest to another treatment were also examined.

**Results: **Up to 26 trials were included in the analyses. Odds ratios of VS for DTG versus other core agents ranged from 1.25 (RAL) to 2.29 (LPV/r) in favour of DTG. The absolute VS risk difference between DTG and other core agents ranged from 0.02 (RAL) to 0.12 (LPV/r). DTG also had a statistically greater increase in CD4 cells compared to all comparators except RAL and BIC (see unadjusted analysis in Table 1). Adjusting for the NRTI backbone used in each of the regimens resulted in DTG having statistically significantly higher odds of achieving VS at 48 weeks compared to all PIs, NNRTIs and EVG/c and was numerically better than RAL and BIC (Table 1). A probabilistic analysis estimated that there is a >90% probability that DTG has greater VS at 48 weeks versus all core agents analysed.

**Table:** Abstract P054 – Table 1. Odds ratios (OR) of virologic suppression <50 copies/mL at 48 weeks and mean difference in CD4 cell change from baseline to Week 48 (fixed effect model)

	Mean OR of virologic suppression (95% Crl)		Mean increase in CD4 cells (95% Crl)	
DTG compared to	Backbone unadjusted	Backbone adjusted	Backbone unadjusted	Backbone adjusted
ATV/r	**1.95 (1.45, 2.61)**	**2.14 (1.56, 2.93)**	**39.5 (23.3, 55.9)**	**30.3 (12.9, 47.8)**
BIC	1.34 (0.89, 2.01)	1.41 (0.93, 2.14)	7.2 (−10.7, 25.2)	0.6 (−17.6, 18.8)
DRV/r	**1.64 (1.14, 2.38)**	**1.73 (1.19, 2.53)**	**28 (5.9, 50.1)**	**23.5 (1.8, 45.3)**
EFV	**1.74 (1.34, 2.27)**	**1.9 (1.43, 2.53)**	**45 (29.9, 60.4)**	**35.9 (19.3, 52.6)**
EVG/c	1.38 (0.93, 2.06)	**1.51 (1.0, 2.28)**	**26.4 (3.9, 48.8)**	17.4 (−5.7, 40.4)
LPV/r	**2.29 (1.69, 3.12)**	**2.48 (1.8, 3.43)**	**28 (6.2, 49.8)**	20.7 (−1.2, 42.5)
RAL	1.25 (0.9, 1.73)	1.30 (0.93, 1.81)	6.6 (−11.8, 24.8)	2.9 (−15.1, 21)
RPV	1.41 (1.0, 1.99)	**1.53 (1.07, 2.21)**	**30.3 (9.8, 50.9)**	**21.3 (0, 42.8)**

Crl = Credible Interval; **bold** **=  statistically significantly different comparisons where lower Crl is >1 for ORs of viral suppression and >0 for mean increase in CD4 cells.

**Conclusions: **Study results showed higher probabilities of VS and larger CD4 increases for DTG compared to some treatments and similar outcomes relative to others. These results suggest DTG is among the most effective treatments available for the initial treatment of HIV‐1 infection.

## P055

### Trends in modification and discontinuation of initial antiretroviral treatment in Turkish HIV‐TR cohort, 2011‐2017

**V Korten^1^, D Gökengin^2^, M Fincancı^3^, T Yıldırmak^4^, S Gencer^5^, H Eraksoy^6^, D İnan^7^, F Kaptan^8^, B Dokuzoğuz^9^, Karaoğlan^10^, A Willke^11^, Ö Ergönül^12^ and HIV‐TR study group^13^**

^1^Infectious Diseases, Marmara University Hospital, Istanbul, Turkey. ^2^Infectious Diseases, Ege University Hospital, Izmir, Turkey. ^3^Infectious Diseases, Istanbul Education and Research Hospital, Istanbul, Turkey. ^4^Infectious Diseases, Okmeydanı Education and Research Hospital, Istanbul, Turkey. ^5^Infectious Diseases, Lütfi Kırdar Education and Research Hospital, Istanbul, Turkey. ^6^Infectious Diseases, Istanbul University, Istanbul School of Medicine, Istanbul, Turkey. ^7^Infectious Diseases, Akdeniz University Hospital, Antalya, Turkey. ^8^Infectious Diseases, Izmir Katip Çelebi University Atatürk Education Training and Research Hospital, Izmir, Turkey. ^9^Infectious Diseases, Ankara Numune Education and Research Hospital, Ankara, Turkey. ^10^Infectious Diseases, Gaziantep University Hospital, Gaziantep, Turkey. ^11^Infectious Diseases, Kocaeli University Hospital, İzmit, Turkey. ^12^Infectious Diseases, Koç University School of Medicine, Istanbul, Turkey. ^13^HIV‐TR Study Group

**Background: **The aim of this study was to compare the frequency, reasons and the predictors for discontinuation and modification of ART before and after the availability of better tolerated and less complex regimens.

**Methods: **A total of 3019 antiretroviral‐naive patients registered in the HIV‐TR cohort who started ART between January 2011 and February 2017 were studied. Only the first modification for each patient within 1 year was included in the analyses. Reasons were classified as listed in the coded form in the web‐based database. Time to treatment modification was analysed using Kaplan‐Meier curves and log‐rank tests, and factors associated with regimen modification were examined using Cox proportional hazards models.

**Results: **Of 3019 patients, initial regimen was modified or discontinued in 379 patients (12.6%) within the first year. The main reason for modification or discontinuation was intolerance/toxicity (41.7%), followed by treatment simplification (9%), death (7.4%), patient's willingness (6.9%), poor compliance (6.6%), to prevent future toxicities (5.5%), virological failure (5%) and provider's preference (5%). Median time to treatment modification was shorter in patients treated with NNRTI‐ compared with integrase strand transfer inhibitor (InSTI)‐ and protease inhibitor (PI)‐based regimens (3.9, 4.9 and 5.6 months, respectively; *p *= 0.049). In a multivariable Cox model, only predictor of discontinuation was baseline AIDS diagnosis, (aHR 1.4, 95% CI 1.1 to 1.8; *p *= 0.01). Discontinuation rate was higher among PI (17.8%) and NNRTI (14.3%) when compared to InSTI‐based regimens (6.3%) (*p* < 0.001). A lower rate of treatment modification for intolerance/toxicity was observed with InSTI‐based regimens (2%) than with NNRTI‐based (6.6%) and PI‐based regimens (7.5%) (*p* < 0.001). However, patients receiving InSTI‐based regimens had less severe disease, indicated by fewer baseline AIDS diagnoses (CD4 < 200/mm^3^ or CDC stage C) and lower HIV RNA levels than those on PI‐based and NNRTI‐based regimens. Similarly, those on InSTI‐based single tablet regimens (STRs) had fewer baseline AIDS diagnoses, but similar HIV RNA levels compared to those on non‐STR InSTI‐based regimens. Patients who achieved HIV RNA <50 and <200 copies/mL within 12 months of ART initiation were 85% and 91% in the ART modified group versus 87% and 93.9% in the continued group (*p *> 0.05).

**Conclusion: **There was a relatively low rate of modification and discontinuation of ART regimens within the first 12 months as compared with other countries [1–4]. InSTI‐based regimens were less likely to be modified than PI‐ and NNRTI‐based ART.

**References**

[1] Vo TT, Ledergerber B, Keiser O, Hirschel B, Furrer H, Battegay M, et al. Durability and outcome of initial antiretroviral treatments received during 2000‐‐2005 by patients in the Swiss HIV Cohort Study. J Infect Dis. 2008;197:1685‐94.

[2] Di Biagio A, Cozzi‐Lepri A, Prinapori R, Angarano G, Gori A, Quirino T, et al. Discontinuation of initial antiretroviral therapy in clinical practice: moving toward individualized therapy. J Acquir Immune Defic Syndr. 2016;71:263‐71.

[3] Sun J, Liu L, Shen J, Qi T, Wang Z, Song W, et al. Reasons and risk factors for the initial regimen modification in Chinese treatment‐naïve patients with HIV infection: a retrospective cohort analysis. PLoS One. 2015;10:e0133242.

[4] Sheth AN, Ofotokun I, Buchacz K, Armon C, Chmiel JS, Hart RL, et al. Antiretroviral regimen durability and success in treatment‐naive and treatment‐experienced patients by year of treatment initiation, United States, 1996‐2011. JAIDS. 2016;71:47‐56.

## P056

### Reasons for choosing darunavir/ritonavir 600/100 mg BID versus 800/100 mg QD in ART‐naïve patients

**A Tavelli^1^, M Palma^2^, S Lo Caputo^3^, G Madeddu^4^, P Bonfanti^5^, B Menzaghi^6^, S Nozza^7^, A Antinori^8^, R Termini^2^, A d'Arminio Monforte^9^, on behalf of Icona Foundation Study Group**

^1^Icona Foundation, Milan, Italy. ^2^Janssen‐Cilag SpA, Cologno Monzese, Italy. ^3^Clinic of Infectious Diseases, Policlinic of Bari, Bari, Italy. ^4^Unit of Infectious Diseases, University of Sassari, Sassari, Italy. ^5^Unit of Infectious Diseases, A Manzoni Hospital, Lecco, Italy. ^6^Unit of Infectious Diseases, ASST della Valle Olona, Busto Arsizio, Italy. ^7^Clinic of Infectious Diseases, San Raffaele Scientific Institute, Milan, Italy. ^8^HIV/AIDS Unit, INMI L. Spallanzani IRCCS, Rome, Italy. ^9^Clinic of Infectious and Tropical Diseases, ASST Santi Paolo e Carlo University of Milan, Milan, Italy

**Background: **Ritonavir‐boosted darunavir (DRV/r) is recommended in ART‐naïve, specifically in those with perceived low adherence and before results of resistance test. DRV/r 600/100 mg twice daily (BID) is licensed for ART‐experienced, while 800/100 mg once daily formulation (QD) is recommended in first‐line and in experienced patient without DRV RAMs. In clinical practice, however, a non‐negligible proportion of ART‐naïve subjects started with DRV/r BID. The aim of this study is to identify patterns of prescription of DRV/r BID in ART‐naive, analysing predictors of DRV/r BID start versus QD.

**Materials and methods: **All patients from Icona cohort that started a DRV/r‐based regimen from ART‐naïve in 2008 to 2017 were included. A cross‐sectional analysis was performed comparing demographics, clinical and lifestyle factors at the time of starting DRV/r in BID or QD, using chi‐square and Wilcoxon test as appropriate. Univariable and multivariable logistic regression models were used to identify predictors of DRV/r BID start.

**Results: **One thousand five hundred and three ART‐naive patients were included: 1297 started DRV/r QD (86%) and 206 DRV/r BID (14%). Eighty‐two percent male, median age 40 years, 80% Italian, 80% in triple therapy, 6% in two‐drug regimen and 14% in combination with >3 ARV drugs (69% with raltegravir and 13% with maraviroc). Median HIV‐RNA in DRV/r QD and BID groups were 5.1 and 5.3 log10 copies/mL (*p *= 0.02), with 31% DRV/r BID with HIV‐RNA >500,000 copies/mL (vs. 22% QD; *p* < 0.01); median CD4 counts were 260 cells/mm^3^ for DRV/r QD and 215 cells/mm^3^ for BID (*p *= 0.07); 48% of DRV/r BID patients had CD4 < 200/mm^3^ (vs. 40% QD; *p *= 0.04). Fitting a logistic regression model, after controlling for potential confounders, older patients (>40 years: adjusted OR [aOR] 1.44; *p *= 0.019), subjects with CD4 < 200 cells/mm^3^ (aOR 1.48; *p *= 0.019), patients given >3 ARV drugs (aOR 2.11; *p *= 0.001), centres from southern Italy (aOR 2.57; *p* < 0.001), patients starting first cART in 2012 to 2013 (aOR 2.46; *p *= 0.001) showed a significantly higher probability of starting DRV/r BID. A decreased probability of DRV/r BID start was observed in AIDS presenters (aOR 0.63; *p *= 0.037). There was evidence that the use of regimens with >3 ARV was greater in people with HIV‐RNA >500,000 than in those with HIV‐RNA ≤500,000 (OR 5.9; *p* < 0.001).

**Conclusions: **Italian clinicians allocate DRV/r BID as first‐line regimen in several scenarios: elderly, severely immune‐suppressed and highly viraemic patients, with more than three ARV drugs and with a peak of use in 2012 to 2013. Subjects with AIDS are mostly given DRV/r QD probably due to the the pill burden of concomitant medications for opportunistic infections.

## P057

### Network meta‐analysis of darunavir/cobicistat/emtricitabine/tenofovir‐alafenamide and elvitegravir/cobicistat/emtricitabine/tenofovir‐alafenamide in treatment‐naive patients with HIV‐1 infection

**S Van Sanden^1^, B Schweikert^2^, C Moecklinghoff^3^ and K Tronczyński^4^**

^1^Health Economics Market Access and Reimbursement, Janssen EMEA, Beerse, Belgium. ^2^RWESA, ICON, Munich, Germany. ^3^Janssen, Medical Affairs, Neuss, Germany. ^4^Health Economics Market Access and Reimbursement, Janssen, Warsaw, Poland

**Background: **In recent years, several single‐tablet regimens (STRs) have been developed for HIV, with the aim of simplifying treatment and improving adherence whilst maintaining safety and efficacy. Two recently approved STR treatment options combine the boosting agent cobicistat and the common nucleotide/nucleoside reverse transcriptase inhibitors (NRTI) backbone F/TAF with the third agent darunavir (D/C/F/TAF) or elvitegravir (E/C/F/TAF). In absence of comparative head‐to‐head trials, we performed an indirect treatment comparison to compare the efficacy and safety between D/C/F/TAF and E/C/F/TAF.

**Methods: **A systematic literature review (SLR) was conducted to identify RCTs including EVG or DRV in combination with two NRTIs. Bayesian fixed and random effect network meta‐analyses (NMA) were performed to compare virological suppression (HIV‐RNA suppression to <50 copies/mL according to FDA Snapshot algorithm) and discontinuation due to the adverse events (DAE), both at Week 48 in treatment‐naïve HIV‐1 infected population. In addition to the SLR, 48‐week results of the AMBER study comparing D/C/F/TAF versus darunavir‐boosted cobicistat (DRV/c) plus emtricitabine and tenofovir disoproxil (TDF/TAF) in a treatment‐naïve population were included in the network by, for the base case, assuming equality between DRV/c and darunavir‐boosted ritonavir (DRV/r). Several sensitivity analyses were performed to assess the impact of alternative definitions of the virological suppression endpoint, inclusion/exclusion of trials related to different patient populations or backbone used or an alternative way to connect the AMBER study to the network.

**Results: **Twenty‐two RCTs were identified by the SLR and selected for inclusion in at least one network. Based on the deviance information criterion (DIC), the fixed effects model was considered as the base case for both endpoints and random effects models were performed as sensitivity analyses. In the base case analysis, no substantial differences in virological suppression were found between D/C/F/TAF and E/C/F/TAF: OR [95% CI] was 1.06 [0.48 to 2.33], with a probability (*p*) of 55% of D/C/F/TAF to be better than E/C/F/TAF. The OR for the DAE was 0.43 [0.08 to 2.39] in the base case analysis, with a probability of 83% of D/C/F/TAF to be better than E/C/F/TAF. Results of the NMA were stable across sensitivity analyses, supporting the robustness of the base case analysis findings.

**Conclusions: **These NMA results suggest similar efficacy between D/C/F/TAF and E/C/F/TAF while for DAE the results suggest that the number of patients who discontinue D/C/F/TAF treatment due to adverse events may be lower or at least similar compared to E/C/F/TAF.

## **TREATMENT STRATEGIES – TARGET POPULATIONS: PRIMARY INFECTION**

## P058

### Comparative virological efficacy, tolerance and immunological recovery at 3 years of different antiretroviral regimens initiated during acute/recent HIV infection

**J Ambrosioni^1^, J Farrera^2^, E de Lazzari^1^, D Nicolás^1^, C Manzardo^1^, M Mosquera^1^, C Ligero^1^, M Marcos^1^, S Sánchez‐Palomino^1^, E Fernández^1^, M Plana^1^ and J Miró^1^**

^1^Infectious Diseases, Hospital Clínic de Barcelona, Barcelona, Spain. ^2^School of Medicine, University of Barcelona, Barcelona, Spain

**Background: **Acute HIV infection is defined as infection of <30 days, and recent infection as <180 days post‐infection. ART in this period reduces the viral reservoir, preserves the immune system, decreases transmission and optimises immune recovery. Guidelines recommend starting ART in all patients; however, the optimal antiretroviral regimen in terms of immunological recovery and tolerability in this setting is unknown.

**Objectives: **To analyse the virological efficacy, tolerance and the immunological reconstitution at 1 and 3 years after starting ART during recent HIV infection. Regimens based on boosted‐protease inhibitors (PI), integrase inhibitors (InSTI) and NNRTI were compared.

**Materials and methods: **Retrospective study of 137 patients with confirmed acute/recent infection who started ART within 6 months post‐infection, between 2003 and 2017. We compared regimens based on: ritonavir/cobicistat boosted‐PI (darunavir, atazanavir, N* *= 28), InSTI (elvitegravir/cobicistat, raltegravir, dolutegravir, N* *= 87) and NNRTI (efavirenz, rilpivirine, N* *= 22), all combined with two nucleoside reverse transcriptase inhibitors (abacavir/lamivudine or TDF‐TAF/emtricitabine). Primary endpoints were virological suppression (viral load [VL] <50 copies/mL) and immune reconstitution (CD4+T cell count * *> 900 cells/µL and CD4/CD8 ratio* *> 1) at 1 and 3 years. Secondary endpoints were adverse events (AEs) leading to ART discontinuation at 1 and 3 years. ITT and PP analysis were performed.

**Results: **Viral suppression (overall suppression of 96% at 1 year and 99% at 3 years) was comparable in all ART regimens. Among the InSTI group, levels of viral suppression were comparable for dolutegravir‐ and elvitegravir‐based regimens. At 1 year there was an increment of 350 CD4+T cells/µL, which was comparable in all ART regimens. Overall 36% and 39% achieved CD4 >900 cells/µL and 43% and 66% a CD4/CD8 >1 at 1 and 3 years, respectively. In a subanalysis of immune recovery comparing Fiebig stages I to V with Fiebig stage VI, starting ART during the earliest Fiebig stages was associated with higher rates of CD4 >900 cells/µL at 3 years (*p *= 0.027). Discontinuation due to AEs was more frequent with NNRTI compared to other ART families (*p *= 0.036 at 1 year, *p *= 0.040 at 3 years) with high rates of neuropsychiatric AEs (Table 1).

**Conclusions: **Viral suppression and immunological recovery were excellent in acute/recent patients, with no differences between ART regimens. Earlier ART initiation (<100 days) was associated with a higher proportion of immunological recovery, favouring Fiebig stages I to V compared to stage VI. NNRTI‐based regimens were associated with higher treatment discontinuation rates due to AEs. Dolutegravir‐ and elvitegravir‐based regimens showed comparable efficacy.

**Table:** Abstract P058 – Table 1. Baseline characteristics and outcome of included patients according to ART regimen

	Total N* *= 137	NNRTI N* *= 22	PI^a^ N* *= 28	InSTI N* *= 87	*p* value
Baseline characteristics					
Age, median (IQR)	34 (31 to 40)	34 (32 to 38)	37 (29 to 44)	34 (30 to 39)	0.718
Gender, n (%)					0.546
Male	130 (95%)	22 (100%)	26 (93%)	82 (94%)	
Female	7 (5%)	0	2 (7%)	5 (6%)	
Transmission group, n (%) N* *= 128					0.931
MSM	115 (90%)	21 (95%)	23 (85%)	71 (90%)	
HTSX	10 (8%)	1 (5%)	4 (15%)	5 (6%)	
IDU	2 (2%)	0	0	2 (3%)	
Unknown	1 (1%)	0	0	1 (1%)	
CD4+T cells absolute count cells/mm^3^, median (IQR)	475 (335 to 583)	491 (368 to 572)	538 (262 to 638)	470 (333 to 578)	0.904
CD4/CD8 ratio, median (IQR)	0.48 (0.31 to 0.72)	0.67 (0.35 to 0.82)	0.38 (0.17 to 0.69)	0.50 (0.33 to 0.71)	0.061
HIV RNA, log10 copies/mL, median (IQR)	4.80 (3.81 to 5.52)	4.53 (3.78 to 5.05)	5.20 (4.78 to 5.83)	4.67 (3.56 to 5.52)	0.063
Fiebig at cohort inclusion, median (IQR)	5 (4 to 6)	6 (4 to 6)	5 (5 to 6)	5 (4 to 6)	0.241
Fiebig at ART initiation, median (IQR)	6 (5 to 6)	6 (5 to 6)	6 (5 to 6)	6 (5 to 6)	0.400
Virological outcomes					
VL <50 at 1 year of follow‐up, n (%) N* *= 119	114 (96%)	21 (95%)	27 (100%)	66 (94%)	0.686
VL <50 at 3 years of follow‐up, n (%) N* *= 57	70 (99%)	18 (100%)	25 (100%)	27 (96%)	1.000
Immunological outcomes					
CD4+ T cell delta at 1 year, mean (SD) N* *= 114	350 (274)	330 (264)	332 (319)	364 (261)	0.705
CD4+ T cell delta at 3 years, mean (SD) N* *= 57	348 (279)	367 (268)	383 (266)	298 (304)	0.518
CD4+ T cell >900 at 1 year, n (%) N* *= 114	41 (36%)	6 (29%)	8 (31%)	27 (40%)	0.419
CD4+ T cell >900 at 3 years, n (%) N* *= 57	22 (39%)	6 (40%)	7 (33%)	9 (43%)	0.745
CD4/CD8 >1 at 1 year, n (%) N* *= 114	52 (46%)	10 (48%)	11 (42%)	31 (46%)	0.986
CD4/CD8 >1 at 3 years, n (%) N* *= 57	36 (63%)	8 (53%)	13 (62%)	15 (71%)	0.518
Toxicity					
At least one adverse event at 1 year, n (%)	36 (26%)	8 (36%)	10 (36%)	18 (21%)	0.146
At least one adverse event at 3 years, n (%)	49 (36%)	11 (50%)	12 (43%)	26 (30%)	0.145
Discontinuation rate^b^ at 1 year, n (%)	13 (9%)	5 (23%)	3 (11%)	5 (6%)	0.036
Discontinuation rate^b^ at 3 years, n (%)	20 (15%)	6 (27%)	6 (31%)	8 (9%)	0.040

Kruskal‐Wallis test was used to assess for quantitative variables; Fisher's exact test and chi‐squared for categorical variables. ^a^boosted with either ritonavir or cobicistat. ^b^discontinuation rate applies to any drug, even replaced by another drug of the same family. HTSX = heterosexual; IDU = intravenous drug user.

## P059

### Comparison of raltegravir (RAL) and boosted darunavir (DRV/b) versus dolutegravir (DTG) both associated with tenofovir/emtricitabine (TDF/FTC) in primary HIV infection (PHI): viro‐immunological outcomes of two different integrase inhibitor (INSTI)‐based strategies

**A Mondi^1^, C Pinnetti^1^, P Lorenzini^1^, M Plazzi^1^, I Abbate^2^, G Rozera^2^, C Agrati^3^, R Libertone^1^, S Menichetti^1^, I Mastrorosa^1^, A Ammassari^1^ and A Antinori^1^**

^1^HIV/AIDS Department, National Institute for Infectious Diseases, Lazzaro Spallanzani IRCCS, Rome, Italy. ^2^Laboratory of Virology, National Institute for Infectious Diseases, Lazzaro Spallanzani IRCCS, Rome, Italy. ^3^Cellular Immunology and Pharmacology Laboratory, National Institute for Infectious Diseases, Lazzaro Spallanzani IRCCS, Rome, Italy

**Background: **The aim of this study was to compare the viro‐immunological response to a four‐ versus three‐drug, both INSTI‐based, regimens in the setting of PHI.

**Material and methods**

Monocentric, prospective, observational study including all patients (pts) diagnosed with PHI from July 2013 to April 2018. ART was started, before GRT results, with TDF/FTC and either RAL+DRV/b or DTG. Data were collected from ART start (baseline, BL) until the last observation. Probability and predictors of achieving virological suppression (VS) (HIV‐RNA <40 copies/mL) and CD4/CD8 >1 were assessed by Kaplan‐Meier curves and Cox regression analysis, respectively.

**Results: **One hundred and forty‐four pts included. At BL: median HIV‐RNA and HIV‐DNA were 5.6 log10 copies/mL (IQR 4.8 to 6.6) and 4.4 log10 10^6^ PBMC/mL (IQR 3.8 to 4.8), respectively; 58% pts had CD4 >500/mm^3^ and 25% CD4/CD8 >1. Fiebig stage was: II/III in 15%, IV in 31%, V in 29% and VI in 23% pts. ART was started, within a median of 5 days (IQR 3 to 9) from diagnosis, with four drugs in 110 pts (76%) or three drugs in 34 pts (24%). BL characteristics did not substantially differ between the two treatment groups. Over a median follow‐up of 18 months (IQR 8 to 23), 139 pts (97%) reached VS. The 12‐month probability of achieving VS was high and similar between the four‐drug and three‐drug groups (98% vs. 96%, *p *= 0.232). After stratification by pre‐ART viraemia, the three‐drug group compared to the four‐drug group showed an increased probability of achieving VS in the stratum with BL HIV‐RNA <500,000 copies/mL (*p *= 0.026), whereas no difference was observed in the higher BL viraemia stratum (Figure 1 a,b). At multivariable analysis, probability of achieving VS was decreased in pts with higher BL HIV‐RNA (aHR 0.56 for each log higher, *p* < 0.001) and HIV‐DNA (aHR 0.60 for each log higher, *p *= 0.015). Conversely, three‐drug versus four‐drug ART (aHR 1.75, *p *= 0.022), BL CD4 > 500/mm^3^ (aHR 1.73, *p *= 0.021) and CD4/CD8 >1 (aHR 1.95, *p *= 0.014) predicted VS achievement. The 12‐month probability of reaching CD4/CD8 >1 did not significantly differ between four‐drug and three‐drug groups (58% vs. 74%, *p *= 0.169). At multivariable analysis, a higher BL CD4/CD8 (aHR 5.89 for each point higher; *p *= 0.003) and BL CD4 >500/mm^3^ (aHR 2.25; *p *= 0.003) predicted the achievement of CD4/CD8 >1.

**Abstract P059 – Figure 1.** Kaplan‐Meier curves estimating the cumulative probability of achieving virological suppression according to pre‐ART viral load.

**Conclusions: **In PHI, INSTI‐including regimens achieved VS in almost all treated patients. In subjects with HIV‐RNA <500,000 copies/mL, the three‐drug DTG‐based ART compared to the four‐drug regimen was associated to a more rapid therapeutic success. Medication adherence, among other factors, may explain this finding but further evaluations are needed.

## P060

### Viro‐immunological efficacy and tolerability of dolutegravir‐based regimens compared to regimens based on other INI, PI, NNRTI in patients with acute HIV infection: a multicentre cohort study

**F Lagi^1^, G Baldin^2^, M Colafigli^3^, A Capetti^4^, G Madeddu^5^, S Tekle Kiros^1^, S Di Giambenedetto^2^ and G Sterrantino^6^**

^1^Department of Experimental and Clinical Medicine, Infectious Disease Unit, AOU Careggi, Florence, Italy. ^2^Institute of Clinical Infectious Diseases, Catholic University of Sacred Heart, Rome, Italy. ^3^Infectious Dermatology and Allergology, IFO S. Gallicano Institute (IRCCS), Rome, Italy. ^4^Department of Infectious Diseases, Luigi Sacco University Hospital, Milan, Italy. ^5^Dept of Medical, University of Sassari, Surgical & Experimental Sciences, Sassari, Italy. ^6^Infectious and Tropical Disease Unit, Azienda Ospedaliero‐Universitaria Careggi, Florence, Italy

**Background: **Dolutegravir (DTG)‐based ART is recommended in first‐line regimens for all individuals with HIV‐1 infection. However, limited data are currently available in HIV‐1 infected patients with acute HIV‐1 infection (AHI). This study aims to compare the tolerability and viro‐immunological efficacy of dolutegravir‐based regimens (DTG group) with regimens based on INI, PI, NNRTI (NODTG) in patients with AHI.

**Material and methods**

Patients diagnosed with AHI between 2015 and 2017 from five different centres in Italy who started ART were included and followed up to 30 April 2018. AHI was defined by the presence of the positive p24 antigen or indeterminate western blot with positive p24 antigen. We collected antiretroviral history, resistance tests and viro‐immunological outcome. Categorical variables were analysed with X2/Fisher's exact test and continuous variables with Wilcoxon sign rank test. Kaplan‐Meier method was employed to assess the probability of virological failure (>50 copies/mL).

**Results: **Data from 43 patients with AHI were retrospectively collected: 20 on DTG, 23 not in NODTG. No difference in the follow‐up in the two groups was observed (median 1.8 for both groups). Overall in the cohort 81.4% were Italian and 83.7% males with a median age of 41 years (IQR 31 to 48). Thirty‐one (72.1%) were MSM. The median time from diagnosis to treatment initiation was 12 days [IQR 5 to 28]. Median days from diagnosis to start therapy was 12 [IQR 5 to 28]. Differences between the two groups were reported in Table 1. Three patients (7.0%) had detectable viraemia at the end of follow‐up (EOF) with no difference between the two groups (*p *= 0.468). Nineteen subjects modified, 15 for simplification, four for toxicity (two on DTG for neurological toxicity, two on elvitegravir for gastrointestinal toxicity). Six patients had transmitted mutations at baseline (none for INI) all in DTG group (*p *= 0.005). The 184V mutation was detected in two patients on 3TC/ABC, both undetectable at the end of follow‐up. The probability of achieving virological suppression during the follow‐up is shown in Figure 1 (log rank: *p *= 0.5672). One patient on DTG with 184V achieved virological suppression after 2 years. CD4+ cell count, and CD4+/CD8+ ratio increased significantly within groups at 3, 6, 12, 24 and 36 months (*p* < 0.05 for all comparisons), without significant differences between the two groups.

**Table:** Abstract P060 – Table 1. Baseline characteristics of the study population

	DTG regimes N = 20	NODTG regimes N = 23	*p* value
Sex Male, n (%)	17 (85.0)	19 (82.6)	0.832
Median age, years [IQR]	33.5 [28 to 42]	45 [40 to 53]	0.006
Risk, n (%) Heterosexual	4 (20.0)	8 (34.8)	0.281
Risk, n (%) MSM	16 (80.0)	15 (65.2)	
Country of origin, n (%) Italy	17 (85.0)	18 (78.3)	0.573
Country of origin, n (%) Romania	2 (10.0)	2 (8.7)	
Country of origin, n (%) Africa	0 (0.0)	1 (4.4)	
Country of origin, n (%) Norway	1 (5.0)	0 (0.0)	
Country of origin, n (%) Portugal	0 (0.0)	1 (4.4)	
Country of origin, n (%) Peru	0 (0.0)	1 (4.4)	
Reason for HIV test, n (%) Risk perception	8 (40.0)	6 (26.1)	0.441
Reason for HIV test, n (%) Flu‐like syndrome	6 (30.0)	6 (26.1)	
Reason for HIV test, n (%) Positive partner	3 (15.0)	2 (8.7)	
Reason for HIV test, n (%) Blood donation	0 (0.0)	1 (4.4)	
Reason for HIV test, n (%) Screening for other diseases	0 (0.0)	3 (13.0)	
Reason for HIV test, n (%) Unknown	3 (15.0)	5 (21.7)	
HCV Ab positive, n (%)	1 (5.0)	0 (0.0)	0.278
HBsAg positive, n (%) Negative	19 (95.0)	14 (60.9)	0.028
HBsAg positive, n (%) Positive	1 (5.0)	6 (26.1)	
HBsAg positive, n (%) Unknown	0 (0.0)	3 (13.0)	
Resistance at baseline, n (%) Negative	14 (60.0)	23 (100)	0.005
Resistance at baseline, n (%) NRTI	2 (10.0)	0 (0.0)	
Resistance at baseline, n (%) NNRTI	2 (10.0)	0 (0.0)	
Resistance at baseline, n (%) NRTI+NNRTI	1 (5.0)	0 (0.0)	
Resistance at baseline, n (%) PI	1 (5.0)	0 (0.0)	
Median CD4 diagnosis cells/µL [IQR]	504 [311 to 710]	557 [339 to 717]	0.792
Median HIV‐RNA Log 10 copies/mL [IQR]	6.0 [5.4 to 6.4]	5.5 [4.9 to 6.3]	0.173
Median days from diagnosis to start therapy [IQR]	10 [5 to 18]	22 [4 to 28]	0.387
Median time to achieve <50 copies/mL (day)	103 [58 to 190]	121 [60 to 197]	0.903
Number of drugs 2	1 (5.0)	0 (0.0)	0.554
Number of drugs 3	15 (75.0)	18 (78.3)	
Number of drugs 4	4 (20.0)	5 (21.7)	
Single tablet regimen, n (%)	6 (30.0)	11 (47.8)	0.233
Fever >38°, n (%)	10 (50.0)	9 (39.1)	0.430
Lymphoadenopathy, n (%)	10 (50.0)	12 (52.2)	0.887
GI symptoms (diarrhoea/vomit), n (%)	5 (25.0)	2 (8.7)	0.149
CD4 < 350 cells/mm^3^ at diagnosis, n (%)	6 (30)	6 (26.1)	0.081
Lue serology positive at the diagnosis, n (%)	3 (15.0)	3 (13.0)	0.853

**Conclusion: **In our setting, antiretroviral therapy in AHI is started very early. DTG shows excellent viro‐immunological efficacy even when NRTI transmitted mutations are present, interruptions rarely occur due to neurological toxicity.

**Abstract P060 – Figure 1.Kaplan‐Meier for the probability of virologic suppression by group.**

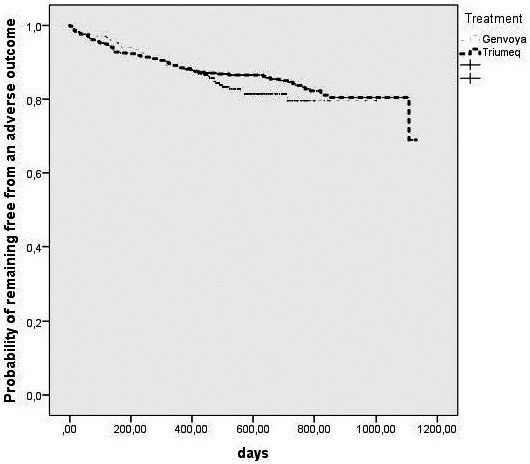

## P061

### Acute HIV infection detection: rapid fourth‐generation test or rapid molecular point‐of‐care HIV test?

**L Carvalho Rocha, D Medina, R Guerreiro, H Correia, J Rojas, F Ferreira, L Veríssimo, N Pinto, J Brito, L Mendão**

CheckpointLX, GAT Portugal, Lisbon, Portugal

**Background: **Acute HIV infection (AHI) is defined by the presence of p24 Ag and/or HIV‐RNA in the absence of HIV Ab [1]. Standard third‐generation tests do not detect AHI. High HIV incidence (2.8%) [2] and prevalence (17.1%) [3] estimates in Portuguese MSM and regular HIV testing (≈7 months between visits) [2], peer counsellor training for AHI syndrome recognition, rapid linkage to care (<72 hours) and access to anonymous partner notification [4] at CheckpointLX (community‐based sexual health centre for MSM) increase the possibility of finding AHI cases [5]. This study aims to compare two screening algorithms for AHI detection at CheckpointLX.

**Methods: **Between November 2016 and November 2017 (enrolment Σ* *= 9.5 months), adult MSM were tested with a combined rapid Ag/Ab test (algorithm 1, AlereTMHIV Combo [6]) and all adult MSM with AHI syndrome OR reactive Ag/Ab test OR whose sexual partner was diagnosed with HIV at CheckpointLX in the prior 6 weeks were tested with rapid molecular HIV‐RNA test (algorithm 2, AlereTMq HIV‐1/2 Detect [7]). All cases were assessed for CD4 cells count (AlereTMPima [8]) and linked to care (<48 hours).

**Results: **Algorithm 1: 0% AHI detected (0 in 2890 tests, 88 reactive: one for Ag [confirmed negative]; one for Ab/Ag [confirmed positive]; 86 for Ab [four confirmed negative]; five for Ag [all confirmed negative] were not considered due to be part of faulty lots, according to manufacturer notice). Algorithm 2: 0.87% (one in 115 tests: 27 non‐reactive rapid Ag/Ab tests [one confirmed positive], one for Ab/Ag [confirmed positive], one for Ag [confirmed negative], 86 for Ab [four confirmed negative, nine refusals to onsite confirmation]). Twelve percent of people confirmed positive were linked to care earlier due to CD4 count <250 cells/mm^3^. 6.33% of people were confirmed negative.

**Conclusions: **Fourth‐generation tests did not add value in AHI detection. Targeted molecular HIV‐RNA allowed AHI detection and spared clients from unnecessary medical appointments and anxiety when reactive tests were confirmed negative immediately. Onsite confirmation reduced the lag time between a reactive test and diagnosis from 6 weeks to 1 hour. Onsite CD4 count enabled priority referrals when immunosuppression was found. HIV testing centres screening algorithms can benefit from onsite HIV‐RNA and CD4 count POC technologies.

**References**

[1] European AIDS Clinical Society (EACS). EACS guidelines version 9.0 [Internet]. October 2017. Available from: http://www.eacsociety.org/guidelines/eacs‐guidelines/eacs‐guidelines.html.

[2] Meireles P, Lucas R, Carvalho C, Fuertes R, Brito J, Campos MJ, et al. Incident risk factors as predictors of HIV seroconversion in the Lisbon cohort of men who have sex with men: first results, 2011–2014. Euro Surveill. 2015;20:pii: 21091.

[3] Mirandola M, Gios L, Davis R, Sherriff N, Zohrabyan L, Toskin I, et al, editors. The Sialon II project. Report on a bio‐behavioural survey among MSM in 13 European cities. Rome: Cierre Grafica; 2016.

[4] Rocha M, Guerreiro R, Pinto N, Rojas J, Ferreira F, Esteves J, et al. Digital partner notification service at a community‐based voluntary counselling and testing centre for men who have sex with men: CheckpointLX, Lisbon, Portugal [Internet]. Available from: http://www.checkpointlx.com/public/uploads/posters/2016/05_CheckOUT_AIDS2016.pdf.

[5] Campos MJ, Rocha M, Rojas J, Ferreira F, Esteves J, Guerreiro R, et al. Impact in HIV care continuum of a tailored community‐based HIV voluntary counseling testing centre for men who have sex with men: CheckpointLX, Lisbon, Portugal [Internet]. Available from: http://www.checkpointlx.com/public/uploads/posters/2016/06_CheckpointLX_AIDS2016.pdf.

[6] Alere™ HIV Combo package insert. January 2017. Available from: http://https://ensur.invmed.com/ensur/broker/ensurbroker.aspx?code=120001736{00AMP00}cs=26729868.

[7] http://https://www.alere.com/en/home/product‐details/alere‐q‐hiv‐12‐detect.html.

[8] http://https://www.alere.com/en/home/product‐details/PimaAnalyserOUS.html.

## P062

### Primary HIV: clinical experience from an outpatient HIV clinic in Portugal

**I Abreu, P Palma, L Graça, R Ruas, R Filipe, E Branco, M Tavares, C Caldas, C Piñeiro, J Soares, R Serrão, A Sarmento**

Infectious Diseases, Centro Hospitalar São João, Porto, Portugal

**Background: **Diagnosis of early HIV infection provides an opportunity to start antiretroviral therapy during the earliest stages of the infection, with benefits on the establishment of the viral set point and immune function preservation as well as reducing the risk of transmission to other individuals [1]. Our aim with this study was to estimate the prevalence of HIV primary infection in a cohort of naïve HIV patients starting antiretroviral therapy and to identify associated sociodemographic, clinical and outcome characteristics.

**Materials and methods: **Observational retrospective study. We selected all newly diagnosed HIV patients that started antiretroviral therapy after enrolment between January 2015 and December 2017 in our HIV outpatient clinic in Centro Hospitalar São João in Oporto, Portugal. Primary HIV infection was considered when there was a positive plasma p24 antigen or HIV‐RNA; negative/indeterminate initial anti‐HIV antibody and clinical symptoms compatible with HIV acute infection (when present). We recovered clinical data regarding baseline characteristics, mode of transmission of HIV, baseline CD4+ count and HIV viral load at 1 month after the beginning of antiretroviral therapy.

**Results: **We identified 332 patients with newly diagnosed HIV infection. The mean age (±SD) was 40.3 (±12.5) years and 254 (76.5%) were men. From these patients, there were 28 cases of primary HIV infection (8.4%, 95% CI 5.68 to 11.66). Patients who were diagnosed with a primary HIV infection had a significantly lower mean (±SD) age (35.9 ± 10.5 vs. 40.7 ± 12.6 years; *p *= 0.073), were male (96.4% vs. 74.7%; *p *= 0.004), were MSM (60.7% vs. 44.4%; *p *= 0.072) and were admitted from the emergency department (50.0% vs. 12.5%; *p* < 0.001). They also had higher median [IQR] CD4 cell count at admission (434.5 [361.5 to 532.5] vs. 386 [149.5 to 545]; *p *= 0.069). Most started INSTI (85.7% vs. 65.5%; *p *= 0.02). After the first month under ART primary HIV patients had a significantly higher median CD4 count increase (210 [91 to 304] vs. 110 [38 to 209]; *p *= 0.013). At the end of follow‐up (mean of 17 months, not significantly different between groups) primary infected patients attained a higher median CD4 cell count (830 [644 to 943] vs. 626 [395 to 859]; *p *= 0.002).

**Conclusions: **Patients with early HIV infection and subsequent early diagnosis and beginning of ART appear to do better than the other patients in terms of immune function recovery after ART, supporting the current recommendation to start ART immediately in these cases.

**Reference: **[1] Cohen MS, Shaw GM, McMichael AJ, Haynes BF. Acute HIV‐1 infection. N Engl J Med. 2011;364:1943‐54.

## TREATMENT STRATEGIES – TARGET POPULATIONS: EXPERIENCED PATIENTS

## P063

### Factors associated with viral load completion in a subset of European countries

**P Adjei^1^, A Cournil^2^, A Stengaard^3^, S Bertagnolio^4^, M Dara^5^, E Vovc^6^, M Jordan^7^ and M Doherty^6^**

^1^Infectious Diseases Division, Tufts Medical Center, Boston, MA, USA. ^2^Unite TransVIHMI, IRD UMI 233, INSERM U1175, Université de Montpellier, Montpellier, France. ^3^Joint Tuberculosis, HIV and Hepatitis Programme, World Health Organization Regional Office for Europe, Copenhagen, Denmark. ^4^Dept of HIV/AIDS & Global Hepatitis Programme, World Health Organization, Geneva, Switzerland. ^5^Division Health Emergencies, Communicable Disease, World Health Organization Regional Office for Europe, Copenhagen, Denmark. ^6^Department of HIV/AIDS, World Health Organization, Geneva, Switzerland. ^7^Department of Public Health and Community Medicine, Tufts University School of Medicine, Boston, MA, USA

**Background: **The World Health Organization (WHO) recommends HIV viral load (VL) testing as the preferred method for monitoring responses to ART. Identifying factors associated with VL completion amongst people on ART may lead to public health and ART program improvements.

**Methods: **De‐identified individual‐level data reported to the joint European Centre for Disease Prevention and Control/WHO database for HIV/AIDS surveillance of The European Surveillance System (TESSy) on people diagnosed with HIV, restricted to those on ART with last clinic attendance reported during 2014 to 2016, were used. VL completion was defined as the percentage with a VL test reported in the year of or the year prior to the year of their last reported clinic attendance. Country, gender, age, year of diagnosis and mode of HIV transmission were assessed in a multivariate model in three geographical areas (East, West, Centre) of the WHO European Region.

**Results: **Thirty‐eight thousand and fifty‐two records (70.6% male) from 16 countries were included: East (N* *= 13,896), West (N* *= 17,754), Centre (N* *= 6402) (Table 1). Heterosexual transmission predominated (41% of diagnoses); 32%, 16% and 11% were due to sex between men, IDU and other, respectively. Overall VL completion was 84.9%, with variability observed between geographical areas (80.0%, 98.6%, 57.7% in East, West, Centre, respectively [*p* < 0.0001]). In the Centre, people on ART aged 20 to 39 years were more likely to have a VL test compared to those <20 years (OR 1.31 [1.01 to 1.71]). Conversely in the East, people over age 20 were less likely to have a VL test (OR 0.52 [0.35 to 0.79] and 0.57 [0.38 to 0.87] for 20 to 39 and 40+ age categories, respectively), and men were less likely to have VL than women (OR 0.83 [0.72 to 0.95]) as were injecting drug users (OR 0.85 [0.74 to 0.99]). No factors analyzed were associated with VL completion in the West. VL completion versus VL suppression is shown in Figure 1.

**Table:** Abstract P063 – Table 1. Viral load completion by country and gender among people on ART with viral load test result reported during the period 2014 to 2016 (n* *= 38,052)

Country (de‐identified)	Total n (%)	Female n (%)	Male n (%)
**East**	13,896 (80.0)		
E1	1021 (96.3)	363 (97.8)	658 (95.4)
E2	2104 (89.7)	721 (93.2)	1383 (87.9)
E3	1059 (96.7)	284 (97.5)	775 (96.4)
E4	6831 (92.5)	2875 (93.6)	3956 (91.8)
E5	571 (42.2)	269 (47.6)	302 (37.4)
E6	436 (84.6)	209 (83.7)	227 (85.5)
E7	1874 (15.3)	811 (15.4)	1063 (15.2)
**West**	17,754 (98.6)		
W1	3235 (97.6)	774 (97.8)	2459 (97.6)
W2	595 (95.5)	174 (95.4)	418 (95.5)
W3	13,896 (99.1)	2585 (99.1	11,311 (99.1)
**Centre**	6402 (57.7)		
C1	453 (26)	138 (30.4)	315 (24.1)
C2	174 (58)	35 (62.9)	139 (56.8)
C3	1281 (100)	149 (100)	1132 (100)
C4	95 (91.6)	13 (100)	82 (90.2)
C5	4267 (46.7)	1783 (47.2)	2484 (46.4)
C6	132 (86.4)	4 (100)	128 (85.9)
**Total**	38,052 (84.9)	11,189 (80.2)	26,858 (86.8)

E1 to E7 represents seven anonymized countries in East of WHO European Region; W1 to W3, three countries in West; and C1 to C6, six countries in the Centre.

Abstract P063 – Figure 1. Viral load testing completion by country* amongst clinic attendees with data reported versus viral load suppression weighted by epidemic size, 2014 to 2016.

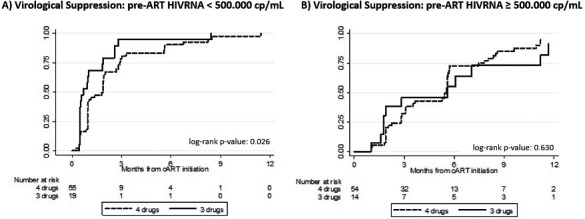

**Conclusion: **Reported VL completion among people on ART is heterogeneous across the region, as are associated factors. Findings highlight disparities in VL completion as well as reporting bias, signaling the need for population‐specific interventions to improve VL completion and geographic‐specific interventions to strengthen surveillance systems’ ability to capture VL completion.

## P064

### Outcomes of patients not achieving primary endpoint from an ibalizumab Phase III trial

**E DeJesus^1^, B Emu^2^, S Weinheimer^3^, Z Cohen^4^, B Cash^5^ and S Lewis^3^**

^1^Infectious Disease, Orlando Immunology Center, Orlando, FL, USA. ^2^Infectious Disease, Yale School of Medicine, New Haven, CT, USA. ^3^HIV, TaiMed Biologics, Irvine, CA, USA. ^4^HIV, Theratechnologies, Montreal, Canada. ^5^HIV, Syneos Health, Somerset, NJ, USA

**Background: **Ibalizumab (IBA) is a humanized monoclonal antibody, a CD4‐directed post‐attachment HIV‐1 inhibitor, recently approved by the US FDA for the treatment of multidrug resistant (MDR) HIV‐1. IBA has shown potent activity against a broad spectrum of primary clinical isolates with no evidence of cross‐resistance with existing antiretrovirals or drug‐drug interactions. TMB‐301 was a 24‐week, Phase III trial conducted in 40 heavily treatment‐experienced patients with MDR HIV‐1. Eighty‐three percent of patients reached the primary efficacy endpoint of ≥0.5 log10 decrease in viral load (VL) 7 days after receiving a 2000 mg IV loading dose of IBA (mean decrease 1.1 log10). Outcomes in patients not reaching the primary endpoint are important given the significant resistance and potentially limited options in these patients.

**Materials and methods: **Three study periods included: Control (Days 0 to 6) – patients continuing on failing therapy; Functional Monotherapy (Days 7 to 13) – failing therapy plus 2000 mg IV loading dose of IBA on Day 7 to measure primary endpoint at Day 14; and Maintenance (Day 14 to Week 25) – optimized background therapy (OBR) initiated on Day 14 and combined with 800 mg IV maintenance doses of IBA every 2 weeks.  

**Results: **Of the 40 patients, seven (18%) did not reach at least a 0.5 log10 VL reduction 7 days after receiving an IBA loading dose of 2000 mg IV. In this group of seven patients, median baseline VL and CD4+ cell count were 21,700 copies/mL and 63 cells/mL, respectively. Three of these seven patients achieved <50 copies/mL and one patient achieved a 1.1 log10 reduction by Week 25. There were two patients that reached VL reductions of 2.1 log10 at Week 9 and 1.6 log10 at Week 21; these patients experienced VL increases from nadir yet remained below baseline VLs at study end. The only patient not completing the trial in this group achieved a 0.5 log10 reduction at Day 21 before withdrawing consent. Mean VL reduction in the seven patients at Week 25 was 1.3 log10 as compared to a 0.1 log10 reduction 7 days after loading dose.

**Conclusion: **A small percentage of patients did not achieve a ≥0.5 log10 reduction in VL 7 days after loading dose of IBA. Some went on to experience viral suppression (VL <50 copies) at Week 25 with IBA in combination with an OBR. Virologic responses in patients with MDR HIV‐1 may take longer to achieve.

## P065

### Psychometric development and preliminary analyses of a new symptom measure for individuals living with HIV: HIV Symptom Rating Questionnaire (HIVSRQ)

**J Romaine^1^, M Murray^2^ and C Bradley^1^**

^1^Health Psychology Research Unit, Royal Holloway, University of London, London, UK. ^2^Medical Affairs, ViiV Healthcare Ltd, London, UK

**Background: **Improvement in HIV survival rates has led to an increasing need for patient‐reported outcome measures, including up‐to‐date symptom measures associated with HIV and current treatments. Using a template for condition‐specific SRQs developed for people with other long‐term conditions (e.g. ThyroidSRQ) this abstract reports on the psychometric development and initial analyses from a new HIV‐specific symptom measure: the HIVSRQ.

**Materials and methods: **Two hundred and fifty‐five participants (UK N* *= 128, US N* *= 127) recruited by Opinion Health completed the questionnaire individually (via post) or with a researcher (via phone). Prior to analysis the HIVSRQ included 64 items drafted in consultation with HIV specialists and tested in 25 qualitative interviews with people living with HIV (14 UK‐English speaking and 11 US‐English speaking). Each item asked if a particular symptom had been experienced in recent weeks and if ‘yes’, was rated for how much this symptom had bothered the respondent on a scale of 1 (not at all) to 4 (a lot).

**Results: **Mean ages of UK participants was 46 years (SD 9.19) and US 51 years (SD 11.69). Mean time since diagnosis: UK 12 years (SD 8.30), USA 18 years (SD 9.29). The male/female ratio was 99/29 in the UK and 104/20 in the US. Exploratory factor analysis (EFA) revealed a clean six‐factor structure comprising 25 items, all items loaded >0.4 and explained 50% of the variance in the data. The six factors were neuromuscular (Cronbach's α* *= 0.84), sexual problems (α* *= 0.82), emotional/mood (α* *= 0.85), minor illnesses (α* *= 0.66), skin problems (α* *= 0.73) and gastrointestinal symptoms (α* *= 0.75). Recognising the pragmatic benefits of a single symptom scale a forced one‐factor EFA was also run. Stepwise removal of low‐loading items resulted in a 38‐item composite subscale. All items loaded >0.4 and explained 28% of data variance. Preliminary analyses found UK participants reported significantly more bother on the composite score (*p* < 0.001), and on sex life (*p *= 0.001), emotion/mood (*p* < 0.001) and minor illness (*p *= 0.002) subscales. Women reported more bother than men on neuromuscular (*p *= 0.04) and emotion/mood (*p *= 0.02) subscales. Interaction effects between country and gender (*p *= 0.056) on the emotion/mood subscale suggest men in the UK (M 8.14, SD 4.93) and women in both countries (UK: M 8.45, SD 5.64; US: M 8.30, SD 5.51) experience emotion/mood symptoms differently from men in the US (M 4.94, SD 4.54).

**Conclusion: **The HIVSRQ is both comprehensive and quick to complete. It has sound psychometric properties, is suitable for use in clinical trials, other research and in routine clinical practice to evaluate key symptoms and help clinicians understand patients’ experiences.

## P066

### Impact of HIV on quality of life: preliminary data exploring differences by sex and country (UK and US) using the HIV Dependent Quality of Life (HIVDQoL) questionnaire

**J Romaine^1^, M Murray^2^ and C Bradley^1^**

^1^Health Psychology Research Unit, Royal Holloway, University of London, London, UK. ^2^Medical Affairs, ViiV Healthcare Ltd, London, UK

**Background: **The few studies to attempt to measure quality of life in people with HIV have relied on measures of treatment satisfaction or health status rather than genuine measures of quality of life. Based on a questionnaire template first developed for people with diabetes (Audit of Diabetes Dependent QoL: ADDQoL) [1,2] the HIVDQoL (HIV Dependent Quality of Life) questionnaire provides a QoL measure that recognises individuals differ both in the aspects of life relevant to them, and in the importance each aspect of life has for their QoL, as well as differing in the level of impact HIV has on these aspects of life. This abstract reports initial data analysis using the newly developed HIVDQoL.

**Materials and methods: **Two hundred and fifty‐five participants (UK 128, US 127), recruited via the internet, completed the HIVDQoL individually (via post) or with a researcher (via phone). The HIVDQoL includes two overview items (generic ‘present QoL’ and ‘HIV‐specific QoL’) and 26 domain‐specific two‐part items measuring HIV impact on the domain and domain importance for QoL. Twelve items have a not‐applicable option. Impact scores (−3 to +1) are multiplied by importance (3 to 0) to give weighted impact (WI) scores. WI scores are summed and divided by the number of applicable items giving an average weighted impact (AWI) score (−9 greatest negative impact to +3 greatest positive impact).

**Results: **Mean ages of participants: UK 46 (SD 9.19), US 51 (SD 11.69). Time since diagnosis: UK 12 years (SD 8.30), US 18 years (SD 9.29): male/female ratio 99/29 (UK), 104/20 (US). All 26 domains impacted negatively on QoL for both men and women; however, in 19 domains women reported greater negative impact on QoL than men. No significant differences were found. UK participants reported greater negative impact of HIV on QoL than US participants in 22 domains. Nine differences were significant (Figure 1). Generic QoL was significantly better in the US (*p* < 0.001). For overall AWI scores, a significant difference was found between countries when time since diagnosis was controlled for (*p *= 0.014). In both countries the greatest negative impact on QoL was the stigma associated with HIV.

Abstract P066 – Figure 1. Comparison of UK and US individual domain item mean weighted impact scores (HIVDQoL).

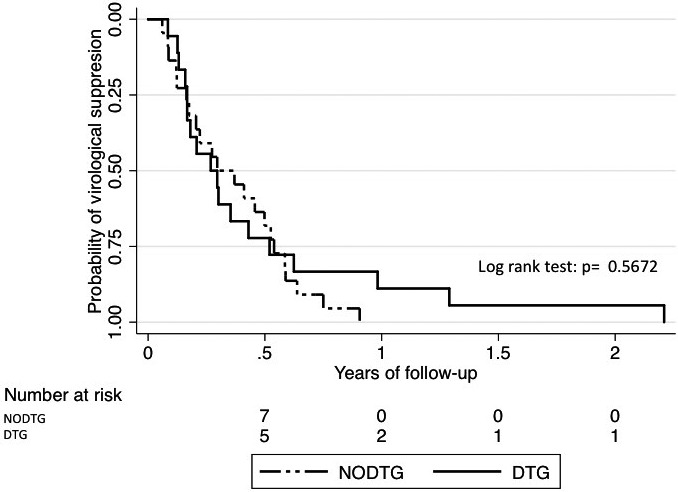

**Conclusion: **The HIVDQoL shows worse generic QoL and greater negative impact of HIV on the QoL of UK participants versus US participants and highlights potential concerns about the impact on women. The HIVDQoL reveals specific areas of life most negatively impacted by HIV, highlighting how efforts may be prioritised to meet the greatest challenges for individuals and populations with HIV.

**References**

[1] Bradley C, Todd C, Gorton T, Symonds E, Martin A, Plowright R. The development of an individualized questionnaire measure of perceived impact of diabetes on quality of life: the ADDQoL. Qual Life Res. 1999;8:79‐91.

[2] Wee HL, Tan CE, Goh SY, Li SC. Usefulness of the Audit of Diabetes‐Dependant Quality of Life (ADDQoL) questionnaire in patients with diabetes in a multi‐ethnic Asian country. Pharmacoecon. 2006;24:673‐82.

## P067

### Viral load suppression rates among HIV‐infected adult patients using optimised health care worker delivery model in Western Nigeria

**S Usman**

Laboratory Services, APIN Public Health Initiatives, Abuja, Nigeria

**Background: **In sub‐Saharan Africa where genotypic drug resistance testing is rarely performed and poor adherence is blamed for the inability to achieve viral suppression and treatment failure, programmatic approaches to preventing and handling these are thus essential. Hypothesis tested was antiretroviral therapy adherence effect on viral load outcome. This study was aimed at determining and monitoring HIV/AIDS disease progression using viral load to provide prognostic information and evaluate patients for viral suppression using the World Health Organization guideline strategies.

**Materials and methods: **This study was an observational, longitudinal, prospective cohort study of subjects living with HIV already initiated on antiretroviral therapy for at least 6 months, enrolled in health facilities across Ekiti State, Western Nigeria, during a 12‐month observation period starting October 2016 till September 2017. Quantitative viral load analysis was done using polymerase chain reaction, Roche COBAS TaqMan 96 Analyzer. All data were statistically analysed, using SPSS Statistics, with multiple comparisons done using post hoc Bonferroni test.

**Results: **A total of 3920 (1005 males and 2915 females) subjects eligible for the study were recruited. Most of them are in the age range of 25 to 54 years, with a mean age of 39.35 ± 10.41 years. Three thousand and eighty‐six (78.7%) and 2363 (60.3%) of the subjects had viral suppression of <1000 RNA copies/mL and <50 RNA copies/mL, respectively. The 834 subjects went through intensive adherence counselling optimised health care worker delivery model for 3 months and viral load test repeated 3 further months after, which made 3377 (86.1%) and 2578 (65.8%) of the subjects have <1000 RNA copies/mL and <50 RNA copies/mL, respectively, during the period of observation. ART adherence has significant effect on viral load outcome from the study hypothesis tested.

**Conclusion: **HIV treatment intensive adherence counselling is key to the achieving viral suppression and determine infection prognosis; thus, routine viral load monitoring will ultimately help in HIV/AIDS disease progression follow‐up and reduce treatment failure tendencies. This will help more patients stay on first‐line regimen and prolong their life expectancy, indicating that the UNAIDS last 90 target is achievable.

## P068

### Determinants of switching to two‐drug combinations with HIV‐RNA ≤50 copies/mL in a cohort of HIV‐infected individuals seen for care in Italy

**A Cozzi‐Lepri^1^, H Diaz‐Cuervo^2^, N Gianotti^3^, G Lapadula^4^, A De Luca^5^, F Maggiolo^6^, S Rusconi^7^, N Bobbio^8^, V Esposito^9^, M Moioli^10^, G Madeddu^11^, A Antinori^12^, A d'Arminio Monforte^13^, on behalf of Icona Foundation Study Group**

^1^Institute for Global Health, University College London, London, UK. ^2^Health Outcomes Research, Gilead Sciences UK, London, UK. ^3^Infectious Diseases, San Raffaele Hospital, Milano, Italy. ^4^Infectious Diseases, AO San Gerardo, Monza, Italy. ^5^Infectious Diseases, University of Siena, Siena, Italy. ^6^Infectious Diseases, AO Papa Giovanni XXIII, Bergamo, Italy. ^7^Infectious Diseases, Sacco Hospital, Milano, Italy. ^8^Infectious Diseases, Ospedali Galliera, Genova, Italy. ^9^Infectious Diseases, AORN Ospedali dei Colli, Napoli, Italy. ^10^Infectious Diseases, Niguarda Hospital, Milano, Italy. ^11^Infectious Diseases, Sassari University, Sassari, Italy. ^12^Infectious Diseases, INMI Spallanzani, Roma, Italy. ^13^Infectious Diseases, San Paolo Hospital, Milano, Italy

**Background: **Although some two‐drug combinations (2DC) are now recommended as alternative in guidelines for use in specific contexts, there is little data documenting how frequently and in which patients these regimens are used in clinical practice.

**Methods: **The study includes data of HIV patients in the Icona Foundation Study cohort with a therapy switch with viral load (VL) ≤50 copies/mL to either triple therapy (TT) or 2DC (first switch after achieving VL ≤50 copies/mL considered baseline) over January 2004–June 2018. 2DC were grouped as DTG‐based (3TC+DTG or RPV+DTG), PI‐based (3TC+DRV+r or cobi, 3TC+LPV+r and 3TC+ATV±r) and other 2DC. Chi‐square test was used to compare categorical factors and Kruskal‐Wallis test to compare medians. Unadjusted percentages in CD4 and eGFR groups were calculated for each treatment group. Multinomial logistic regression was used to identify factors associated with the probability of switching to DTG‐ and PI‐2DC versus TT. For factors with global *p *≤ 0.5 specific contrasts (DTG‐2DC vs. TT; PI‐2DC vs. TT) were calculated.

**Results: **Four thousand one hundred and ninety‐eight switches were included. Median age of patients was 44 years, baseline CD4 571 cells/mm^3^, 22% female, 13% of non‐Italian origins. 4.6% switched to DTG‐2DC (3.3% 3TC+DTG, 1.2% RPV+DTG), 7% to PI‐2DC (3.0% 3TC+DRV+r or cobi, 0.4% 3TC+LPV+r, 3.3% 3TC+ATV±r), 8% to other 2DC and 81% to TT. In the unadjusted analysis, compared to patients switched to TT (6% with CD4 at switch 0 to 200; 13% 200 to 350; 81% >350 cells/mm^3^), those on DTG‐2DC (3%, 6% and 91%, respectively) and PI‐2DC (1%, 9% and 90%, respectively) were switched with higher CD4 (*p* < 0.001) and lower eGFR (3% eGFR <60; 32% 60 to 90; 65% >90 min/mL/1.73 m^2^ in TT vs. 15%, 47% and 38% in DTG‐2DC; 10%, 47% and 43% in PI‐2DC; *p* < 0.001). In the adjusted analysis (Table 1), compared to TT switches to 2DC occurred more frequently in recent years, in older participants and in those with less history of virological failure; participants switched to PI‐2DC showed higher CD4 and lower eGFR; DTG‐2DC occurred more commonly in people without hypertension.

**Table:** Abstract P068 – Table 1. Adjusted^a^ odds ratio (aOR) of switching to 2DC versus TT from fitting a multinomial multivariable logistic regression

Factors	DTG‐based^b^	PI‐based^c^	Global *p* value^d^
Age			0.003
Per 10 years older	1.21 (0.75 to 1.95)	1.20 (1.03 to 1.39)	
*p* value^e^	0.004	0.02	
CD4 count cells/mm^3^			0.002
201 to 350	0.70 (0.35 to 1.42)	0.60 (0.37 to 0.98)	
0 to 200 vs. 350+	0.85 (0.30 to 2.37)	0.26 (0.09 to 0.76)	
*p* value^e^	0.99	0.02	
Year of switch			<0.001
Per more recent	1.95 (1.72 to 2.22)	1.14 (1.09 to 1.19)
*p* value^e^	<0.001	<0.001	
Number of ARVs previously failed			<0.001
Per 3 additional	0.67 (0.57 to 0.79)	0.71 (0.63 to 0.80)	
*p* value^e^	<0.001	<0.001	
Hypertension			0.01
Yes vs. no	0.56 (0.39 to 0.81)	0.90 (0.67 to 1.21)	
*p* value^e^	0.002	0.5	
eGFR (CKD‐EPI)			0.001
Per 10 mL/min/1.73 m^2^ lower	1.08 (0.96 to 1.21)	1.14 (1.04 to 1.25)	
*p* value^e^	0.18	0.007	

^a^adjusted for gender, mode of HIV transmission, origin, HBV‐status, HCV‐status, smoking, duration of exposure to ART, AIDS diagnosis, diabetes and cardiovascular disease. ^b^3TC‐DTG or RPV‐DTG. ^c^3TC‐DRV+r or cobi or 3TC‐LPV+r or 3TC‐ATV±r. ^d^global chi‐square for the association of factors with one of the switch strategies (triple therapy as the comparator). ^e^contrasts chi‐square *p* values.

**Conclusions: **Although switches to 2DC occurred more frequently in recent years, over 80% of suppressed patients switched to TT. Patients appeared to be selected to 2DC based on older age and less previous failure. Selection to specific 2DC groups is also based on specific comorbidity profile, while changes to PI‐2DC are more frequent in patients with higher CD4. Further research is necessary to assess the clinical outcomes of these strategies.

## P069

### Effectiveness, persistence and safety of E/C/F/TAF, F/TAF+3rd agent or R/F/TAF in treatment‐experienced HIV‐1 infected patients: 12‐month results from the German TAFNES cohort study

**H Hillenbrand^1^, H Knechten^2^, T Kuemmerle^3^, S Scholten^4^, N Schuebel^5^, R Haubrich^6^, M Heinzkill^7^, K Goerner^7^ and H Stellbrink^8^**

^1^Clinical Care, MVZ Praxis City Ost, Berlin, Germany. ^2^Clinical Care, Praxis Dr. Knechten, Aachen, Germany. ^3^Clinical Care, Praxis am Ebertplatz, Cologne, Germany. ^4^Praxis Hohenstaufenring, Cologne, Germany. ^5^Clinical Care, Klinikum Osnabrück, Osnabrück, Germany. ^6^Clinical Science, Gilead Sciences, Foster City, CA, USA. ^7^Clinical Science, Gilead Sciences, Munich, Germany. ^8^Clinical Care, ICH Study Center, Hamburg, Germany

**Background: **Successful ART has converted HIV infection into chronic disease. Minimising side effects and optimising long‐term tolerability of ART are essential requirements for achieving durable healthy ageing. In clinical trials, tenofovir alafenamide (TAF) showed comparable effectiveness and less off‐target effects on renal and bone markers than tenofovir disoproxil fumarate (TDF). The 24‐month prospective TAFNES cohort study was initiated to provide real‐world data and evaluate the effectiveness and safety of TAF‐based regimens when used in routine clinical care.

**Methods: **Evaluation of Month‐12 (M12) outcomes in treatment‐experienced adults of the TAFNES cohort who were switched to either E/C/F/TAF, F/TAF+3rd agent or R/F/TAF at least 9 months prior to data‐cut (May 2018). Outcome measures included ART persistence, virological effectiveness (HIV‐RNA < 50 copies/mL, modified ITT analyses [mITT], discontinuation* *= failure, loss‐to‐follow‐up/missing* *= excluded), serious/non‐serious adverse drug reactions (SADRs/ADRs) and health‐related quality of life (HRQoL) using validated questionnaires (SF‐36, HIV Symptom Index [HIV‐SI], treatment satisfaction [TS]). 

**Results: **Four hundred and thirty‐four patients (90.6% men, median age 51 years) were eligible for analysis, 154 patients switched to E/C/F/TAF, 146 to F/TAF+3rd agent (30% dolutegravir, 16% nevirapine, 13% raltegravir, 11% darunavir/ritonavir) and 134 to R/F/TAF. Baseline characteristics and previous antiretroviral regimens are shown in Table 1; 95.3% were on suppressive ART prior to switch, 92.6% were switched from TDF‐based ART. Reasons for switch (multiple responses allowed) to F/TAF‐based ART were simplification (n* *= 127, 29.3%), patient wish (n* *= 138, 31.8%), side effects on previous ART (n* *= 185, 42.6%) and other (n* *= 77, 17.7%; including aiming at minimising long‐term toxicity (n* *= 55, 12.7%)). ART persistence did not differ significantly between groups (Figure 1); 8.1% (n* *= 35/434) discontinued from the study before M12 visit, after a median time of 26 weeks (including discontinuation of F/TAF‐based regimens due to ADRs (2.1%), virological failure (0.7%), death (0.5%), patient decision (1.4%) or other reasons (2.1%), and loss‐to‐follow‐up (1.4%)). At M12 visit, 89.9% (n* *= 372/414) had HIV‐RNA levels <50 copies/mL (mITT), i.e. 94.4% of patients treated with E/C/F/TAF (n* *= 134/142), 86.6% of patients on F/TAF + 3rd agent (n* *= 123/142) and 88.5% of patients on R/F/TAF (n* *= 115/130). By M12, 16 ADRs (in 2.8% of patients (N* *= 12) and seven SADRs (in 0.9% of patients (N* *= 4)) were documented. SF‐36 and HIV‐SI scores remained stable, TS increased significantly.

**Table:** Abstract P069 – Table 1. Baseline characteristics and reasons for discontinuation of F/TAF‐based ART

	Overall	E/C/F/TAF	F/TAF+3rd agent	R/F/TAF
N (%)	434 (100)	154 (36)	146 (33)	134 (31)
Male gender, n (%)	393 (91)	137 (89)	137 (93)	119 (89)
Age, years, median (IQR)	51 (40 to 57)	45 (36 to 54)	56 (53 to 61)	44 (35 to 52)
Age ≥50 years, n (%)	251 (58)	58 (38)	146 (100)^a^	47 (35)
CDC stage C, n (%)	90 (21)	34 (22)	38 (26)	18 (13)
CD4 count, cells/µL, median (IQR)	629 (472 to 831)	633 (487 to 882)	577 (431 to 800)	678 (521 to 816)
HIV‐RNA, log copies/mL, median (IQR)	1.3 (1.3 to 1.6)	1.3 (1.3 to 1.6)	1.3 (1.2 to 1.6)	1.3 (1.2 to 1.6)
HIV‐1 RNA <50 copies/mL, n (%)	405 (95)	140 (93)	140 (97)	125 (96)
Previous antiretroviral regimen, n (%)				
INI‐based	153 (35)	96 (62)	53 (36)	4 (3)
NNRTI‐based	164 (38)	26 (17)	25 (17)	113 (84)
PI‐based	75 (17)	25 (16)	39 (27)	11 (8)
Other	42 (10)	7 (5)	29 (20)	6 (5)
Discontinuations by Month 12, n (%)	35 (8.1)	10 (6.5)	11 (7.5)	14 (10.4)
Reason for discontinuation of F/TAF‐based ART, n (%)				
ADRs	9 (2.1)	3 (1.9)	1 (0.7)	5 (3.7)
Patient decision	6 (1.4)	1 (0.6)	3 (2.1)	2 (1.5)
Virological failure	3 (0.7)	1 (0.6)	0 (0.0)	2 (1.5)
Death	2 (0.5)	0 (0.0)	1 (0.7)	1 (0.7)
Other	9 (2.1)	1 (0.6)	6 (4.1)	2 (1.5)
Loss‐to‐follow‐up	6 (1.4)	4 (2.6)	0 (0.0)	2 (1.5)

^a^inclusion criteria age ≥50 years. IQR = interquartile range.

**Abstract P069 – Figure 1**. Kaplan‐Meier analyses: time on study drug.

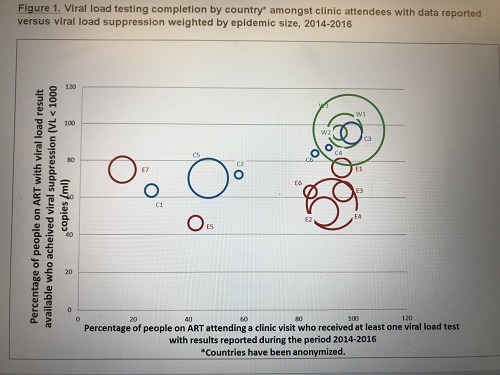

**Conclusion: **F/TAF‐based regimens showed good persistence in this observational cohort of adult treatment‐experienced patients with low discontinuation rates due to ADRs (2%) or virological failure (<1%). Significant improvements in treatment satisfaction demonstrate a high degree of patient acceptability of using F/TAF as part of single‐or multi‐tablet regimens.

## P070

### Determinants of switching to TAF‐based cART or two‐drug combinations with HIV‐RNA ≤50 copies/mL in a cohort of HIV‐infected individuals seen for care in Italy

**A Cozzi‐Lepri^1^, A d'Arminio Monforte^2^, A Capetti^3^, A De Luca^4^, A Castagna^5^, D Bernacchia^6^, F Bai^2^, M Zaccarelli^7^, A Cingolani^8^, C Mussini^9^, C Perno^10^, A Antinori^7^, on behalf of Icona Foundation Study Group**

^1^IGH, University College London, London, UK. ^2^Infectious Diseases, San Paolo Hospital, Milano, Italy. ^3^1st Division Infectious Diseases, Sacco Hospital, Milano, Italy. ^4^Infectious Diseases, University of Siena, Siena, Italy. ^5^Infectious Diseases, San Raffaele Hospital, Milano, Italy. ^6^3rd Division Infectious Diseases, Sacco Hospital, Milano, Italy. ^7^Infectious Diseases, INMI Spallanzani, Roma, Italy. ^8^Infectious Diseases, UCSC Roma, Roma, Italy. ^9^Infectious Diseases, University of Modena, Modena, Italy. ^10^University of Milano, Virology, Milano, Italy

**Background: **Randomised studies have shown that switching to a TAF‐based regimen is generally safer than continuing to take TDF‐containing regimens, particularly for bone/kidney health. How these trial results might have impacted on daily prescriptions and the determinants of switching to TAF‐based regimens have not been thoroughly investigated.

**Material and methods**

The analysis includes data of HIV patients in the Icona Foundation Study cohort who showed a stable viral load (VL) ≤50 copies/mL while on triple cART after 1 January 2016 (baseline). We investigated the incidence of switch to dual combinations (2DC) or TAF‐based cART. Standard survival analysis of time to switch by means of Kaplan‐Meier (KM) curves were used. Separate models were used for the endpoints of switching to 2DC or TAF‐based cART. Cox regression models were used to identify independent predictors of time to switch. A competing risk KM analysis was conducted to jointly model the two type of switches.

**Results: **A total of 1471 participants were included, 1320 (90%) currently on TDF‐based cART and 151 (10%) on TDF‐sparing cART, all with a HIV‐RNA ≤50 copies/mL. Median (IQR) age was 36 (29 to 43) years, CD4 count 530 (322 to 752) cells/mm^3^ (14% with < 200 cells/mm^3^), CKD‐EPI eGFR 99 (85 to 111) mL/min/1.73 m^2^, total cholesterol 168 (143 to 193) mg/dL, 21% female, 49% acquired HIV through MSM, 30% of foreign origin, 6% were co‐infected with HCV, 12% had been diagnosed with AIDS before baseline. In the TDF‐based regimen group, the most common anchor drugs besides FTC were EVG (27%), RPV (25%), DTG (18%) and DRV/r (9%). In the TDF‐sparing group, the most common anchor was DTG (54%), RTG (13%) or DRV/r (13%) with a backbone of 3TC/ABC. In the separate endpoint approach to analysis, by 2 years from baseline, the probability of switch to 2DC was 14% (95% CI 11 to 17%) and 26% (95% CI 23 to 29%) to TAF‐based cART. Figure 1 shows the percentages using the competing event approach. Table 1 shows factors found to be independently associated with the probability of switching stratified by switch type.

Abstract P070 – Figure 1. Competing risk KM curves of switching to 2DC or TAF‐based cART.

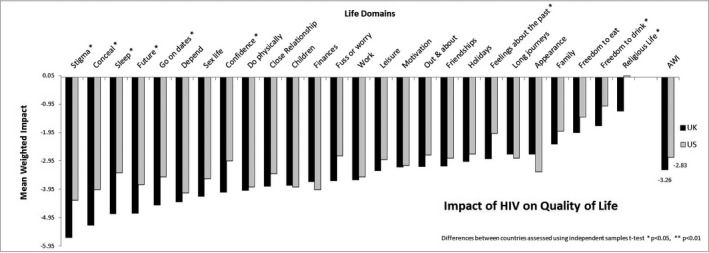

**Table:** Abstract P070 – Table 1. Relative hazards of switching with a VL ≤50 copies/mL by switch type (TAF‐based cART vs. 2DC) from fitting two separate Cox regression models

Factors	TAF cART	*p* value	2DC	*p* value
Baseline regimen				
TDF‐sparing	1.00		1.00	
TDF‐based	37.59 (9.30, 152.0)	<0.001	4.75 (1.69, 13.35)	0.003
eGFR (CKD‐EPI)				
60+	1.00		1.00	
0 to 59	0.59 (0.28, 1.24)	0.16	5.64 (2.27, 13.98)	<0.001
Calendar year of baseline				
Per more recent	9.16 (6.68, 12.55)	<0.001	9.14 (5.59, 14.95)	<0.001
Anchor drug class				
Other	1.00		1.00	
INSTI	9.48 (5.98, 15.03)	<0.001	0.24 (0.14, 0.40)	<0.001
PI/r	3.72 (2.12, 6.53)	<0.001	0.56 (0.32, 0.96)	0.04

Also adjusted for: gender, mode of HIV transmission, nationality, AIDS diagnosis, HCV co‐infection status, age, CD4 count at baseline, total cholesterol at baseline, use of blood pressure lowering drugs, number of ART drugs previously virologically failed, anchor drug of regimen at baseline (INSTI‐ vs. PI/r‐ vs. RPV‐based) which all failed to be independently associated with any of the studied endpoints.

**Conclusions: **The majority of switches to TAF/2DC were from TDF‐based regimens. A lower eGFR led to a greater probability of switching to 2DC but not to TAF‐based regimens. Patients appear to be switched away from their successful regimen more frequently in recent periods. Selection to specific 2DC groups is also based on whether a person was already on a less potent cART (e.g. RPV‐based regimens).

## P071

### Effectiveness and safety of a dual therapy with boosted darunavir and dolutegravir in patients with an advanced HIV infection

**J Pasquau^1^, C Garcia^1^, L Muñoz^2^, S Ferra^3^, T Brieva^4^, G Verdejo^5^, J Garcia^6^, V Sanchez^2^, S De Jesus^1^, M Galvez^3^, D Vinuesa^2^, M Lopez^1^ and C Hidalgo^1^**

^1^Infectious Diseases, Hospital Universitario Virgen de las Nieves, Granada, Spain. ^2^Infectious Diseases, Hospital Universitario San Cecilio, Granada, Spain. ^3^Infectious Diseases, Hospital Torrecardenas, Almeria, Spain. ^4^Infectious Diseases, Hospital Reina Sofia, Cordoba, Spain. ^5^Infectious Diseases, Hospital Lozano Blesa, Zaragoza, Spain. ^6^Infectious Diseases, Hospital Costa del Sol, Marbella, Spain

**Introduction: **The benefits of new ARV drugs allow treatment simplification and a reduction of potential toxicity associated wih ART in patients who have previously been exposed to many ART combinations and have a long‐term, difficult‐to‐manage HIV infection. The objective of this study was to analyse the effectiveness and safety of a dual therapy (DT) with dolutegravir plus boosted darunavir (DTG + bDRV) in these patients.

**Methods: **Observational, retrospective, multicentre study to analyse the effectiveness of DTG+bDRV, defined as the ability to achieve or maintain complete viral suppression, i.e. viral load (VL) < 50 copies/mL. Two analyses were carried out: “ITT‐Snapshot” (proportion of patients with VL < 50 copies/mL at or after Week 24 of all patients with complete follow‐up, including treatment discontinuations) and “Observed Data” (proportion of last VL < 50 copies/mL of all patients with any VL determinations after switch to DT).

**Results: **In a first analysis, we analysed 109 patients with a median age of 50 years, a known HIV infection for 21 years, 12 years of ART treatment and six previous ART combinations, with a CD4 nadir of 76 cells/uL and 56% had a history of AIDS. 58.7% had a history of virological failures (VF). Baseline VL was >50 copies/mL in 39.4% of patients and >200 copies/mL in 21.1%. The reason for switch was simplification/optimisation in 61.5%, VF in 23.9%, toxicity/intolerance 7.3% and other 6.4%. Eighty of 109 have completed 24 weeks of follow up and of these, 75 (93.75%) had VL < 50 copies/mL. Of the five patients with detectable VL, two achieved viral resuppression (VL < 50) without switching from the DT and the other three were patients with evident poor treatment adherence, with no drug resistance mutations. Of the other 29/109 patients: two were lost to follow‐up, eight still have no VL determinations after the switch, three have discontinued the DT due to adverse events (insomnia and digestive issues), 12 have not completed 24 weeks yet and four have not come to the Week 24 visit. The effectiveness of the DT was 84.3% (75/89) according to “ITT‐Snapshot” analysis and 94.9% (93/98) according to the “Observed Data” analysis.

**Conclusions: **Dual therapy with DTG + bDRV is an attractive and highly effective simplification/rescue strategy for patients with an advanced and difficult‐to‐treat HIV infection.

## P072

### Dual therapy with dolutegravir plus ritonavir‐boosted or unboosted atazanavir as a maintenance treatment in highly experienced HIV‐infected patients

**A Fayçal^1^, B Abdi^2^, G Peytavin^3^, G Tebano^1^, L Schneider^1^, S Seang^1^, A Simon^4^, R Tubiana^1^, M Valantin^1^, A Marcelin^2^, C Katlama^1^ and R Palich^1^**

^1^Infectious Diseases, Pitié‐Salpetrière Hospital, Paris, France. ^2^Virology, Pitié‐Salpetrière Hospital, Paris, France. ^3^Toxicology‐Pharmacology, Bichat Hospital, Paris, France. ^4^Clinical Immunology, Pitié‐Salpetrière Hospital, Paris, France

**Background: **Dolutegravir (DTG) and atazanavir (ATV) are two drugs with high antiviral potency, good tolerability profile and synergistic pharmacological interaction with a boosting effect of ATV on DTG. We aimed to evaluate whether a dual therapy based on the association DTG/ATV±ritonavir (DTG/ATV±r) can control HIV replication in pretreated and virologically controlled HIV‐infected patients. 

**Materials and methods: **This observational study included all HIV‐infected patients having started DTG/ATV±r combination between June 2014 and December 2017 at Pitié‐Salpêtrière Hospital. The primary endpoint was the proportion of patients without virological failure (one HIV plasma viral load [pVL] >200 copies/mL or two consecutive pVL >50 copies/mL) at Week (W)24. The secondary endpoint was the proportion of patients without virological failure at W48 for patients having completed at least 48 weeks from the switch. 

**Results: **Twenty patients were included, including 13 having completed at least 48 weeks from the switch. Fifteen of 20 (75%) were men. The median (IQR) characteristics at baseline were: age, 58 years (52 to 64); time since HIV diagnosis, 26 years (20 to 28) with CD4 nadir, 154 cells/mm^3^ (56 to 197); CD4 count, 450 cells/mm^3^ (350 to 551); CD4/CD8 ratio, 0.55 (0.38 to 0.70). All patients had pVL < 0 copies/mL at baseline, with median (IQR) duration of virological suppression, 7 years (3 to 9). The proportion of patients with previous exposition to NRTIs, NNRTIs, protease inhibitors (PIs) and integrase strand transfer inhibitors (INSTIs) was 100%, 90%, 100% and 85%, respectively. The proportion of resistance‐associated mutations (RAMs) at baseline for the different drugs is given in Table 1. ATV at baseline was ritonavir‐boosted in 10/20 patients (50%). The proportion of patients without virological failure was 100% (95% CI 80 to 100) at W24, and 92% (95% CI 77 to 100) at W48. Only one virological failure occured, at W48: pVL* *= 6760 copies/mL (treatment breakage during 2 months; no drug detected in plasma), without acquired RAM, and recovered virological control 2 months after the resumption of the same treatment. Two patients had discontinuated the studied strategy before W24 for adverse events (alopecia and rash), and two others between W24 and W48 (one non‐treatment‐related death and one lost‐to‐follow‐up).

**Table:** Abstract P072 – Table 1. Proportion of HIV strains with resistance‐associated drugs (RAMs) at baseline, from history of RNA genotypes for each patient

NRTIs		NNRTIs		PIs		INSTIs	
Lamivudine/emtricitabine	14/16 (88%)	Nevirapine	11/16 (69%)	Lopinavir/r	2/16 (13%)	Raltegravir	3/9 (33%)
Abacavir	14/16 (88%)	Efavirenz	11/16 (69%)	Atazanavir/r	3/16 (19%)	Elvitegravir/c	4/9 (44%)
Tenofovir	10/16 (63%)	Rilpivirine	11/16 (69%)	Darunavir/r QD	2/16 (13%)	Dolutegravir QD	3/9 (33%)
		Etravirine	10/16 (63%)	Darunavir/r BID	1/16 (6%)	Dolutegravir BID	1/9 (11%)

Data are given as Number of HIV strains with RAMs / Number of patients with availabe genotype(s) for analysis (%). ANRS algorithm, v.28, April 2018, was used to perfom the analysis. BID * *=  twice a day (600 mg 2/d for darunavir/r and 50 mg 2/d for dolutegravir); QD * *=  once a day (800 mg 1/d for darunavir/r and 50 mg 1/d for dolutegravir).

**Conclusions: **This pilot study shows that DTG/ATV±r dual therapy is able to maintain sustained virological control in patients with multiresistance. This strategy could be relevant to lighten the treatment of highly experienced patients, for whom NRTI and NNRTI use is not possible.

## P073

### Virological outcomes on two‐drug antiretroviral therapy regimens in treatment‐experienced HIV+ patients in Pune, Western India

**R Gawali^1^, C Saraf^2^ and A Dravid^1^**

^1^Medicine, Poona Hospital and Research Center, Pune, India. ^2^Medicine, Precision Diagnostics and Biosciences, Pune, India

**Introduction: **Two‐drug ART has the potential to reduce toxicity, preserve future treatment options and decrease cost [1]. Data about virological outcomes on two‐drug ART regimens (2DR) in treatment‐experienced HIV patients in resource‐limited settings like India are scarce. Our objective was to describe virological outcomes on 2DR in three tertiary level private hospitals in Pune, India.

**Methods: **In this retrospective study conducted between 1 March 2009 and 1 March 2018, ART‐experienced patients starting protease inhibitor (PI)‐based ART (two NRTI plus one PI, double boosted PI or one PI with one integrase inhibitor) were identified. Those who started 2DR (double boosted PI or one PI with one integrase inhibitor) and completed minimum 6 months of follow‐up were included as cases. Patients exposed to three‐drug PI‐based ART (3DR) were taken as controls. Patients were observed from regimen start date (baseline) until regimen discontinuation, loss to follow‐up, death or study end (1 March 2018). Outcomes were stratified by viral load (VL) at baseline (virological failures [VF]: ≥20 copies/mL; virological suppression [VS]: <20 copies/mL). Virological responses during follow‐up were defined as follows: VS, VL < 20 copies/mL; and VF, two consecutive VL ≥20 copies/mL.

**Results: **Out of 413 treatment‐experienced patients starting PI‐based ART, 101 (24%) were started or switched on to 2DR. Median age was 42 (IQR 35 to 47) years, and cohort included 31.3% females. Median CD4 count and VL prior to starting 2DR was 87 (IQR 40.5 to 266) cells/mm^3^ and 78,154 (IQR 12,000 to 144,000) copies/mL respectively. 89.9% patients were VF while 11.1% were VS at baseline. The most common 2DR used was double boosted PI (40%) or ritonavir‐boosted PI plus integrase inhibitor (60%). 70.7% patients used 2DR as second‐line ART (after failure of NNRTI‐based first‐line ART) while 29.3% used it as third‐line ART (after failure of NNRTI‐ and PI‐based ART). Median duration of follow‐up on 2DR was 18 (IQR 12 to 32) months. Amongst patients with baseline viraemia, 78.7% achieved VS on 2DR. VF was seen in 21.3% patients. Thirty‐seven percent patients with VF had persistent low level viraemia (plasma viral load between 20 and 1000 copies/mL). There was no difference in virological suppression rates between treatment‐experienced patients exposed to 2DR versus 3DR (78.7% vs. 76.5%, *p *= 0.12). All patients with suppression at baseline maintained VS at follow‐up on 2DR. 

**Conclusion: **PI‐based 2DR show excellent virological response in a real‐world setting of ART‐experienced patients in India.

**Reference: **[1] Pierone G, Henegar C, Fusco J, Vannappagari V, Aboud M, Ragone L, et al. Virologic response to 2‐drug ART regimens among treatment‐experienced HIV+ patients [abstract 510]. 25th Conference on Retroviruses and Opportunistic infections; 2018 Mar 4‐7; Boston (MA), USA.

## P074

### Hospital admissions due to medical conditions in a public health care system with free access to antiretroviral treatment

**À Andreu‐Crespo^1^, F Sala‐Piñol^1^, A Vilariño^1^, G Cardona‐Peitx^1^, J Santos Fernandez^2^, M Lorenzo Górriz^1^, J Módol Deltell^3^, B Clotet Sala^4^, X Bonafont Pujol^1^ and J Llibre Codina^2^**

^1^Pharmacy, Hospital Universitari Germans Trias i Pujol, Badalona, Spain. ^2^Infectious Diseases HIV Unit, Hospital Universitari Germans Trias i Pujol, Badalona, Spain. ^3^Medical Direction, Hospital Universitari Germans Trias i Pujol, Badalona, Spain. ^4^Infectious Diseases, Hospital Universitari Germans Trias i Pujol, Badalona, Spain

**Background: **To characterise the hospital admissions of HIV‐infected subjects due to medical (non‐surgical) conditions in a university hospital in an scenario of free access to health care and to ART.

**Methods: **We retrospectively identified all HIV‐infected subjects admitted to internal medicine and infectious diseases services from  1 October 2016 to 31 December 2017 in Barcelona. Surgical and gynaecological conditions were excluded. All medical discharge reports were thoroughly reviewed to identify the diagnostics, ART, hospital stay length and complications and patient baseline characteristics. The overall cost was also evaluated.

**Results: **We identified 139 hospital admissions from 102 patients. The median length of hospitalisation was 8.0 days (IQR 12 days). Of these, 77 (75.4%) were men, 12 (11.7%) were MSM. The mean age was 49.3 years. Fourteen (13%) patients were active intravenous drug injectors, and 52 (50%) were prior intravenous users. The mean number of admissions per patient was 1.35 (range 1 to 5) and 21 patients were admitted more than once. In only nine (8.8%) subjects the HIV infection was newly diagnosed. The mean CD4 count at admission among those with known HIV infection was 399 cells/mL and 82 cells/mL for naïves. At admission 67 (48%) patients had an undetectable viral load (<50 copies/mL). The previous ART remained unchanged in most subjects. Among those who changed their ART, 75% started an integrase inhibitor (INI)‐ and 25% a protease inhibitor‐based regimen. All treatment‐naives started an INI‐based regimen. The most common causes of admission were non‐AIDS defining infectious conditions (n = 67) and AIDS‐defining conditions (n = 31). Only seven subjects were admitted due to non‐AIDS defining neoplasia. Other non‐AIDS defining conditions (non‐infectious, non‐neoplasia) were seen in 34 subjects. Six patients (4.3%) died during their hospital admission: four non‐AIDS defining infections, one AIDS‐defining neoplasia (linfoma) and one non‐AIDS defining neoplasia. We did not observe a correlation between HIV‐RNA plasma suppression and hospital stay length: 12.2 and 16.0 days for those with HIV‐RNA <50 or >200 copies/mL at admission, respectively (p = NS). According to current hospital fares in Spain, the hospital stays generated a whole cost of 880.402 €.

**Conclusions: **Non‐AIDS defining infectious diseases were the main reason for admission due to medical conditions. Most subjects initiating or changing their ART during the hospital stay were placed on an INI‐based regimen. Despite having access to free medical attention, most infected subjects admitted their lack to link to care.

## P075

### HIV‐EVOL: changes in ART during hospitalisation from 2009 to 2017 in a tertiary hospital in Madrid (Spain)

**A Díaz‐de Santiago^1^, S De La Fuente^1^, L Biscari^2^, P Martin^2^, C Folguera^3^ and A Ángel‐Moreno^1^**

^1^Internal Medicine, Hospital Universitario Puerta de Hierro Majadahonda, Madrid, Spain. ^2^Medicine, Autónoma de Madrid University, Madrid, Spain. ^3^Pharmacy, Hospital Universitario Puerta de Hierro Majadahonda, Madrid, Spain

**Background: **Our main objective is to evaluate HIV treatment during hospital stay for any reason at University Hospital Puerta de Hierro (tertiary Public Health System centre) during 2009 to 2017.

**Materials and methods: **Observational retrospective and descriptive study (AEMPS code: EVOL‐VIH. ADS‐TEN‐2018‐01). We used Stata programme (v.12.0) for statistical analyses.

**Results: **We observed 597 admissions from 260 patients in last nine years (840 patients followed up in our HIV unit, admission rate 4.85 per 100 patient‐years). 74.6% were male, with median age of 48 years. Eighty‐five percent Spanish, IDU 38%, MSM 21%, heterosexual 19%. HIV infection median time was 18 years. Mean time since first ART was 16.2 years and mean time since last ART regimen before admission was 2.5 years. Fifty‐one percent AIDS CDC stage. Median time with undetectable HIV plasma load was 11 years. Mean nadir and baseline TCD4 +  cell count were 164 and 364, respectively. Baseline CD4/CD8 ratio mean was 0.54. Forty‐five percent showed HCV co‐infection (58% if admissions were analysed instead of patients). Sixty‐one percent had undetectable HIV load before hospitalisation (72% on ART). Twenty‐one percent (126/597) changed ART regimen during hospital stay. Reasons for change were: virological failure (47.2%), naive (29.6%), toxicity (11.2%), drug‐drug interactions (6.4%), simplification (5.6%). ART regimen types were: monotherapy 0%, dual therapy 8%, triple therapy 82.4%, quadruple therapy 9.6%. Dual therapies were used between 2015 and 2017 (from 18.7% in 2015 to 14.3% in 2017). Triple treatments decayed (from 100% in 2009 to 78.6% in 2017, *p* = 0.040). Four‐drug regimens diminished too (from 25% in 2010 to 7.1% in 2017, *p* = 0.040). Twenty‐eight percent of patients changed to a single‐tablet regimen (STR) during admission. In triple standard therapies, third drug represented: NNRTI 25%, boosted PI 36%, INSTI 38%, but this proportions varied among study period: NNRTI decreased from 44% in 2009 to 10% in 2017; boosted PI decayed from 55% in 2009 to 20% in 2017; and INSTI increased its proportion from 0% in 2009 to 70% in 2017 (all *p* values <0.0001).

**Conclusions: **ART is changed in 1/5 patients during hospitalisation, due to virological failure specially. Dual therapies increased its representation, although three‐drug regimens still continue being most prevalent ART during hospital stay. INSTI increased markedly and has become ART election in more than 2/3 of patients at the end of study period.

## TREATMENT STRATEGIES – TARGET POPULATIONS: IDU

## P076

### Chemsex drugs on the rise among MSM: a longitudinal analysis of the Swiss HIV Cohort Study from 2007 to 2017

**B Hampel^1^, K Kusejko^1^, R Kouyos^1^, J Boeni^2^, M Flepp^3^, M Stoeckle^4^, A Conen^5^, C Béguelin^6^, P Künzler‐Heule^7^, D Nicca^7^, A Schmidt^8^, H Nguyen^1^, J Delaloye^9^, M Rougemont^10^, E Bernasconi^11^, A Rauch^6^, H Günthard^1^, D Braun^1^ and J Fehr^1,2^**

^1^Infectious Diseases and Hospital Epidemiology, University Hospital Zurich, Zurich, Switzerland. ^2^Institute of Medical Virology, University of Zurich, Zurich, Switzerland. ^3^Infectious Diseases, Center of Infectious Diseases Zurich, Zurich, Switzerland. ^4^Infectious Diseases and Hospital Epidemiology, University Hospital Basel, Basel, Switzerland. ^5^Infectious Diseases and Hospital Hygiene, Cantonal Hospital Aarau, Aarau, Switzerland. ^6^Infectious Diseases, University Hospital Bern, Bern, Switzerland. ^7^Institute of Nursing Science, University of Basel, Basel, Switzerland. ^8^Division of Infectious Diseases, Cantonal Hospital St. Gallen, St. Gallen, Switzerland. ^9^Division of Infectious Diseases, University Hospital Lausanne, Lausanne, Switzerland. ^10^Division of Infectious Diseases, University Hospital Geneva, Geneva, Switzerland. ^11^Division of Infectious Diseases, Regional Hospital Lugano, Lugano, Switzerland. ^12^Public Health, University of Zurich, Zurich, Switzerland

**Background: **Chemsex refers to the use of sex‐enhancing drugs among MSM in combination with specific sexual and social behaviour. Little data on this development and the associated health risks exist. We used longitudinal data of the Swiss HIV Cohort Study (SHCS) to quantify the use of chemsex drugs and factors associated with their use in MSM living in Switzerland.

**Methods: **We analysed data about all recreational drugs reported in the SHCS from 2007 to 2017 and compared drug use among MSM and non‐MSM. We analysed potential associations between the consumption of methamphetamine, γ‐hydroxybutric‐acid/γ‐butyrolactone (GHB/GBL), 3,4‐methylenedioxymethamphetamine (MDMA/XTC), cocaine and amphetamine, and the adherence to antiretroviral therapy, condomless sex, depression, level of education and prevalence of syphilis and HCV infection. In addition, we performed a data quality check with 109 questionnaires on sexual behaviour and drug use, which were completed in parallel to the SHCS questionnaires by MSM who participated in an HCV elimination trial.

**Results: **Overall, we observed a stable rate (9.0%) of recreational drug use in the SHCS. For MSM, however, there was an increase in overall drug use from 19.7% in 2007 up to 24.8% in 2017, especially for methamphetamine (0.2% to 2.0%) and GHB/GBL (1.0% to 2.9%). This trend was almost exclusively seen in the region of Zurich (Figure 1). The use of each of the drugs, methamphetamine, GHB/GBL, cocaine, XTC/MDMA and amphetamine, was significantly associated with condomless sex, higher incidence of depression, syphilis and HCV co‐infection. The data quality check between the two data sources showed that participants provided more information about their drug use when the questionnaire was filled out by the patients themselves (HCV elimination trial) compared to when it was administered by a health care professional (SHCS).

**Conclusions: **We found an alarming increase in the use of chemsex drugs, especially methamphetamine and GHB/GBL, among MSM living with HIV in Switzerland, and a high association with co‐infections and depression. Health care professionals need to develop awareness for these new substances and should be trained in how to address this topic. Harm reduction programmes need to be installed for the MSM population.

**Abstract P076 – Figure 1**. Total number of MSM taking chemsex drugs, by center.

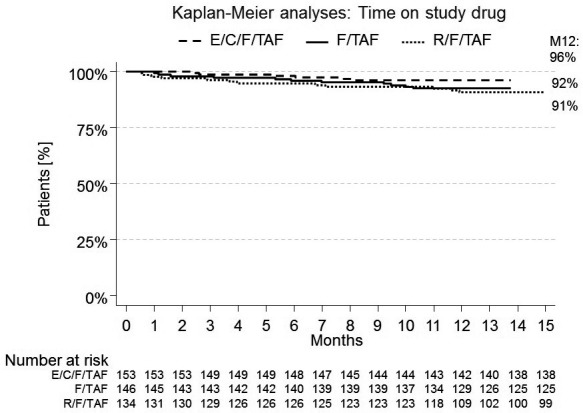

## P077

### Using real‐time phylodynamic analysis to assess and guide public health interventions in an HIV outbreak among people who inject drugs in Scotland

**M Ragonnet‐Cronin^1^, A Bradley‐Stewart^2^, R Metcalfe^3^, E Peters^4^, R Gunson^2^, A McAuley^5^, C Milosevic^6^ and A Leigh Brown^7^**

^1^Medicine, University of California, San Diego, San Diego, CA, USA. ^2^Virology, NHS Greater Glasgow and Clyde, Glasgow, UK. ^3^Medicine, NHS Greater Glasgow and Clyde, Glasgow, UK. ^4^Brownlee Centre for Infectious Diseases, NHS Greater Glasgow and Clyde, Glasgow, UK. ^5^School of Health and Life Sciences, Glasgow Caledonian University, Glasgow, UK. ^6^Public Health, NHS Greater Glasgow and Clyde, Glasgow, UK. ^7^Institute for Evolutionary Biology, University of Edinburgh, Edinburgh, UK

**Background: **From 1995 to 2015 fewer than 10 HIV diagnoses per year had been recorded among people who inject drugs (PWID) in Scotland, but in November 2014, a sharp rise in cases was recorded. The majority of infections were subtype C, presented the same two drug‐resistant mutations and were part of a single genetically‐linked outbreak based on their genetic sequences.

**Materials and methods: **All HIV pol genetic sequences associated with the outbreak were collated against a background of UK and international HIV subtype C sequences. Phylodynamic analysis was used to estimate the average number of onward transmissions within the outbreak for each infected individual over time (the reproductive ratio, Re) according to a birth‐death SIR model, implemented in BEAST. We then estimated the probability of transmission between each pair of individuals in the outbreak using source attribution methods.

**Results: **The outbreak included 145 individuals with very closely related viruses (<1% genetic distance). The average number of onward transmissions (Re) was estimated to currently be below 1, but to have been above 1 for the majority of the duration of the outbreak. When Re is above 1, each case is leading to ≥1 additional case and an epidemic is not under control whereas when Re is below 1, an outbreak is likely to die out over time. Based on a Bayesian phylodynamic analysis, we found that the most likely date at which Re began to fall was mid‐2015, before additional public health measures in response to the outbreak were fully developed. Transmission prevention measures including provision of injecting equipment and opioid substitution therapy were in place prior to the outbreak. For each potential transmission pair in the outbreak, we estimated the probability of a direct transmission event. Less than 25% of individuals were responsible for >50% of the transmission events inferred, highlighting the potential of source attribution methods for helping to guide real‐time phylogenetic interventions towards those most likely to transmit.

**Conclusions: **Individuals could be prioritized based on the number of transmissions they are associated with (high outdegree); however, we do not currently know how outdegree changes over time. We are currently identifying whether the key set of transmitters in the outbreak changed over time, and estimating the proportion of infections coming from unsampled individuals. Importantly, the ethics and acceptability of this type of intervention remain to be established.

## P078

### An outbreak of HIV amongst homeless people who inject drugs (PWIDs): an innovative HIV clinical service model adaptation leading to successful clinical outcomes

**R Metcalfe, C Glover, K Brown, E Peters**

Brownlee Centre, Gartnaval General Hospital, NHS Greater Glasgow and Clyde, Glasgow, UK

**Background: **Since November 2014, Glasgow has witnessed a significant rise in HIV diagnoses amongst homeless PWIDs, almost exclusively using heroin and cocaine, with 125 new diagnoses. In Glasgow, injecting equipment provision (IEP) is free and provided at multiple sites across the city alongside a comprehensive and accessible addictions service providing free opiate substitution therapy. The existing model of hospital‐based HIV care is not suitable for this cohort and a new service model to target this under‐served population has been implemented.

**Methods: **We reviewed the cohort to describe the epidemic and measured effectiveness of the new service with clinical outcome measures. The model includes: a weekly consultant‐led HIV clinic within the homeless health facility, also providing sexual and reproductive health and soft tissue infection expertise; a dedicated BBV specialist outreach nurse; ART dispensed via community pharmacies.

**Results: **One hundred and twenty‐five PWIDs have been diagnosed with HIV, of whom 112 are confirmed clade C virus with primary NNRTI mutations. There were more new diagnoses in 2017 than 2016 (37 vs. 30), with the majority, 54/125 (43.2%), diagnosed in hospital settings. The mean age is 42 and 46/125 (36.8%) are female. Seventeen of 125 (13.6%) are deceased, 4/125 (3.2%) have moved. Of those with results, 38/117 (32.4%) had avidity <40%. Of those who were diagnosed out with acute HIV infection, 33/85 (38.9%) had a baseline CD4 count <350 cells/mm^3^ (mean 250; range 88 to 349). Sixty‐nine of 122 (56.6%) have current hepatitis C co‐infection. Of those with complete information, 61/83 (73.4%) have reported sexual contacts alongside a history of IDU. Within the current cohort, 69/104 (66%) have attended the consultant‐led BBV clinic in the homeless health facility, heavily supported by the BBV outreach specialist nurse. One hundred and two of 104 (98.1%) have ever received ART, with 99/104 (95.1%) on a current prescription, via hospital or community‐based pharmacy care. Ninety of 104 (86.5%) had an HIV viral load <200 copies/mL at last check.

**Conclusion: **Despite comprehensive IEP and addictions services, HIV is spreading rapidly amongst homeless PWIDs in Glasgow. At the time of diagnosis, there is a mix of acute HIV infection and long‐term undiagnosed HIV infection and females are disproportionately affected. Information suggests both parenteral and sexual transmission. Traditional service models are not suitable for this group and we have developed a new model of care, with a holistic approach. Excellent clinical outcomes demonstrate its success. The adaptation of clinical HIV services is vital to improve health outcomes and reduce onward transmission to control the epidemic in this highly complex and multiply disadvantaged group.

## P079

### Is early ART achievable in people who inject drugs (PWIDs) living with HIV?

**R Metcalfe^1^, A McAuley^2^, L Wallace^2^, S Hutchinson^2^ and D Goldberg^2^**

^1^Brownlee Centre, Gartnaval General Hospital, NHS Greater Glasgow and Clyde, Glasgow, UK. ^2^Blood Borne Viruses/Sexually Transmitted Infection, Health Protection Scotland, Glasgow, UK

**Background: **Evidence for ART as HIV treatment as prevention (TasP) in parenteral transmission is limited [1] with modelling studies suggesting scale up of ART in this population plays a smaller role in reducing onward transmission than reducing injecting risk [2,3]. However, early ART initiation also has significant individual health benefits [4] with international guidance changing to recommend ART regardless of CD4 count from September 2015. Studies have demonstrated delay in ART initiated to PWIDs, compared to other risk groups [5,6], which may be due to perceived or actual poor engagement in care and ART adherence. Since the transition to providing early ART worldwide, there is a lack of real‐world evidence on whether this is achievable in PWIDs. We sought to compare time from HIV diagnosis to ART initiation in PWIDs with another risk group, MSM, before and after September 2015, which coincided with TasP being implemented as an intervention to tackle an HIV outbreak amongst PWIDs in Glasgow City.

**Method: **The clinical HIV database was interrogated to determine all those diagnosed with HIV from 1 June 2012 to 31 May 2018, with parenteral drug use or MSM identified as a risk factor for HIV acquisition, and who attended our service for first contact with HIV services. Data were collected on basic demographics, date of diagnosis, date of ART start and CD4 count at diagnosis. Analysis focussed on those with baseline CD4 count over 350 cells/mm^3^, in each group.

**Results: **See Table 1.

**Results: **

**Table:** Abstract P079 – Table 1. Time to ART for PWIDs and MSM, with baseline CD4 counts over 350 cells/mm^3^

	PWIDs	PWIDs	MSM	MSM
	Pre‐Sept 2015	Post‐Sept 2015	Pre‐Sept 2015	Post‐Sept 2015
Total	32	45	72	38
Gender (male)	23/32 (71.8%)	31/45 (68.9%)	72/72 (100%)	38/38 (100%)
Mean age at diagnosis (years)	37.7	39.5	34.2	35.0
Mean CD4 at diagnosis (cells/mm^3^)	615.6	563.9	614.7	572.1
Median time from diagnosis to ART start (days)	384.5 (IQR 568.8)	111 (IQR 189)	154 (IQR 506.5)	21.5 (IQR 12)

**Conclusion: **Within our cohort, PWID and MSM risk groups are similar in terms of age and baseline CD4 count. After September 2015, an 86% reduction in time from diagnosis to ART initiation was observed in MSM, compared to 71% reduction in the PWID group, with a much larger individual variation seen in the PWID group. These results demonstrate that early ART is achievable in this complex group but inequity remains between PWIDs and MSM in early ART provision, despite TasP being implemented as an intervention during an outbreak of HIV amongst PWIDs. This is likely due to the complexity of managing HIV in PWIDs within the traditional HIV service model, which requires more resource and the development of innovative models of HIV care to deliver early ART to this multiply disadvantaged group. We aim to further this work by reviewing data from these groups for the whole of Scotland.

**References**

[1] Wood E, Milloy MJ, Montaner JS. HIV treatment as prevention among injection drug users. Curr Opin HIV AIDS. 2012;7:151‐6.

[2] Fraser H, Mukandavire C, Martin NK, Hickman M, Cohen MS, Miller WC, et al. HIV treatment as prevention among people who inject drugs a re‐evaluation of the evidence. Int J Epidemiol. 2017;46:466‐78.

[3] Nosyk B, Zang X, Min JE, Krebs E, Lima VD, Milloy MJ, et al. Relative effects of antiretroviral therapy and harm reduction initiatives on HIV incidence in British Columbia, Canada, 1996–2013: a modeling study. Lancet HIV. 2017;4:PE303‐10.

[4] The INSIGHT START Study Group, Lundgren JD, Babiker AG, Gordin F, Emery S, Grund B, Sharma S, et al. Initiation of antiretroviral therapy in early asymptomatic HIV infection. N Engl J Med. 2015;373:795‐807.

[5] Lert F, Kazatchkine MD. Antiretroviral HIV treatment and care for injecting drug users: An evidence‐based overview. Int J Drug Policy. 2007;18:255‐61.

[6] van Asten L, Zangerle R, Hernández Aguado I, Boufassa F, Broers B, Brettle RP, et al. Do HIV disease progression and HAART response vary among injecting drug users in Europe? Eur J Epidemiol.

## P080

### Providing HIV ARVs via community pharmacies alongside opiate replacement therapy (ORT) during an HIV outbreak amongst people who inject drugs (PWIDs)

**R O'Hara^1^, L Murphy^1^, R Metcalfe^1^, E Peters^1^, A Harrison^2^ and K Brown^1^**

^1^Brownlee Outpatients, Gartnavel General Hospital, Glasgow, UK. ^2^Community Pharmacy Development Team, Prescribing and Pharmacy Policy GGC, Glasgow, UK

**Background: **Since November 2014, there is an ongoing outbreak of HIV among homeless PWIDs in Glasgow. Within this cohort, ongoing substance misuse and homelessness are common. The existing model of pharmacy care required patients to attend outpatient clinic appointments and collect medication from the hospital pharmacy on site. This cohort have difficulties engaging with the traditional hospital‐based HIV service and therefore, since July 2016, ARV medication has been provided via community pharmacies alongside ORT, supervised where required. This service has been extended to all those who will benefit, including those not within the outbreak cohort. Close working between HIV hospital pharmacy and community pharmacy development team (CPDT) created a bridge between primary and secondary care pharmacies. Patients are identified by medical and nursing staff to receive ARVs via community pharmacy and the hospital team arrange provision of medication to community pharmacy (if required). Documentation includes a patient registration form from the hospital to CPDT, then CPDT organise registration process of the community pharmacy and finance. ARV prescriptions are provided by prescribers in the HIV team, which are clinically screened by a HIV pharmacist and sent to community pharmacy.

**Method: **The existing pharmacy and HIV clinical databases were interrogated to identify patients who have received ARVs via this method from July 2016 to the end of March 2018.

**Results: **As of end of March 2018, 77 patients have registered at 34 pharmacies for community pharmacy provision of ARVs; of these, 28/77 (36%) are no longer using this method of receiving ARVs; 12/28 (43%) stopped due to incarceration, 6/28 (21%) have died, 4/28 (14%) are collecting from hospital pharmacy, 2/28 (7%) discontinued due to side effects (one headaches, one nausea) and 4/28 (14%) other. Forty‐nine of 77 (64%) are currently receiving ARVs via this route and of those with an HIV viral load check within the last six months, 36/48 (75%) have a viral load <40 copies/mL.

**Conclusion: **In the midst of an HIV epidemic we have initiated a new model of pharmacy ARV provision creating links between hospital and community pharmacies, to benefit people with complex needs. The high level of uptake of this service suggests feasibility and acceptability within this group. High level of viral load suppression indicates high levels of adherence but we plan to prospectively monitor adherence of ARVs to this method of ARV provision.

## P081

### Nurses at the forefront: a new HIV service model for people who inject drugs (PWIDs) in Glasgow

**C Glover^1^, S Curtis^2^, S Kirkwood^1^, P McGinness^1^ and P Anderson^1^**

^1^Brownlee Centre, Gartnavel General Hospital, Glasgow, UK. ^2^Community Addictions Team, Hunter Street Homeless Health Centre, Glasgow, UK

**Background: **There is an ongoing outbreak of HIV amongst homeless PWIDs in Glasgow: 125 cases since November 2014. The traditional model of care for people living with HIV is a centralised hospital‐based service, which can be a barrier to accessing care and can impact on engagement in care and adherence to ARVs. Hospital‐based blood‐borne virus (BBV) clinical nurse specialists (CNS) have developed a new outreach model of care for this group.

**Materials and methods: **A review of electronic records of 104 in current cohort (deaths excluded) was conducted to achieve the following aims: 1. Describe the innovative nursing approaches and interventions implemented to engage with patients and support them into HIV care; 2. Illustrate the effectiveness of the role of the CNS in the complex needs of these patients using surrogate markers.

**Results: **An outreach nursing model was initiated in November 2015. This service has adapted to suit their multiple complex needs by providing the following: a weekly drop‐in nurse‐led clinic within the Homeless Health Centre, offering treatment of HIV, HCV and HBV; working with pharmacies to support ARVs dispensed via community pharmacies alongside opiate replacement therapy (ORT); liaison with other NHS teams (addictions, sexual health, GPs, inpatient specialty teams, rehabilitation centres) involved with the cohort; links with social work, prisons and third sector organisations; assertive ‘street’ outreach actively seeking out patients in the most deprived conditions, e.g. rough sleeping and temporary accommodations; supporting the wider HIV multidisciplinary team. Effectiveness of the service was measured using the following surrogate markers: 69/104 (66%) of cohort using outreach service, with remaining 35/104 (33%) of cohort exclusively attending hospital care; 12 additional HIV patients (outwith outbreak cohort) attending outreach service; 99/104 (95%) treated with ARVs, with 41/104 (39%) ARVs via community pharmacy with ORT and 16/104 (15%) ARVs dispensed by CNS; 13 patients commenced on HCV treatment.

**Conclusion: **People with multiple complex needs require an innovative and flexible model of HIV care. Nurses are integral to outreach work and key in supporting and advocating on behalf of underserved populations. Nurses are highly trained and experienced in communication skills, negotiation skills and compassion, which is essential in supporting this vulnerable group and the many services which they are linked with. Assertive outreach and adapting clinic setting has achieved better links with support services and improved provision and retention of care for homeless PWIDs in Glasgow.

## P082

### Successful treatment of hepatitis C in a HIV co‐infected underserved people who inject drugs (PWID) population in Glasgow, UK

**H Black, P Anderson, C Chung, V Pickard, K Perrow, S Peters**

Infectious Diseases, Brownlee Centre, Gartnavel General Hospital, Glasgow, UK

**Background: **There is an ongoing outbreak of HIV in Glasgow among people who inject drugs (PWIDs). Over 50% are co‐infected with HCV. Despite high risk of onward transmission there is a reluctance to treat PWIDs. We have developed a novel approach to engage with this traditionally hard‐to‐reach cohort. With recent advances, HCV treatments are now oral, effective, short duration with minimal drug‐drug interactions and toxicity. This allows for community pharmacy administration linked with daily dispensing of opiate substitution therapy (OST) and/or HIV ART.

**Methods: **We conducted a clinical review of this cohort identifying individuals with a positive HCV antibody (HCVAb) test. Those who were HCV RNA positive were then targeted for assessment and treatment consideration. Active drug use was not a contraindication to HCV treatment assuming the patient was actively engaging and thought stable enough for therapy adherence. The outreach clinic in the homeless addictions service provided all care, assessments and HCV treatment alongside HIV care. Data collection and review is an ongoing part of clinical care.

**Results: **At the time of analysis there were 122 PWID HIV outbreak patients. We excluded 21 (17 deceased and four moved health boards). The 101 patients included were all HCVAb positive. Forty‐five of 101 (44%) were HCV RNA positive and not treated or planned for treatment. Twenty‐six of 101 (25%) had been treated or planned for treatment. Thirty of 101 (29%) were HCV RNA negative and never treated likely representing self‐clearance. Of the treatment group 18/26 had completed treatment, 4/26 currently on treatment and 4/26 had a treatment start date documented. Of those patients completed 17/18 were HCV RNA negative at end of treatment (EOT). Two of 18 were HCV RNA positive: one treatment failure and one re‐infection over one year post‐treatment. Table 1 contains patient characteristics. Treatment numbers have increased exponentially from one patient in 2015 to three in 2016, five in 2017 and reaching 17 so far in 2018.

**Table:** Abstract P082 – Table 1. Cohort patient characteristics

		N	%
Gender	Male	66/101	65
	Female	35/101	35
Age	20 to 30	5/101	5
	31 to 40	55/101	55
	41 to 50	30/101	29
	>50	11/101	11
Genotype	1A	51/101	51
	2	2/101	2
	3	17/101	17
	Unknown	30/101	29
Previous hepatitis C treatment		1/101	1
Patients on antiretroviral therapy		97/101	96
Viral suppression (viral load <40)		82/97	85
Patients on opiate substitution therapy		75/101	74

**Conclusions: **Hepatitis C treatment can be successfully delivered in underserved populations but the care model has to be support engagement. We have shown increasing numbers of patients receiving HCV treatment representing the growing PWID cohort in addition to the superior reach of the outreach model. Our treated patients are predominately HCV RNA negative at EOT with low re‐infection rates to date. This has important public health implications for prevention of onward transmission and reducing future liver disease and related morbidity and mortality.

## TREATMENT STRATEGIES: ADHERENCE

## P083

### Real‐world persistence of E/C/F/TAF versus DTG+ABC/3TC regimens for treatment of HIV in a large Spanish cohort: VACH

**R Teira^1^, A Romero^2^, B Roca^3^, M Munoz‐Sanchez^4^, M Sepulveda^5^, T Puig^6^, N Espinosa^7^, M Merino^8^, P Geijo^9^, M Castano^10^, V Estrada^11^, E Ribera^12^, P Domingo^13^, B De la Fuente^14^, M Montero^15^, M Galindo^16^, J Peraire^17^, E Martinez^18^, F Lozano^19^, A Terron^20^, J Garcia^21^, E Deig^22^, A Munoz‐Sanz^23^ and M Gutierrez^13^**

^1^Hospital de Sierrallana, Torrelavega, Spain. ^2^Hospital Clinico, Puerto Real, Spain. ^3^Hospital General, Castellon, Spain. ^4^Hospital de Basurto, Bilbao, Spain. ^5^Hospital Virgen de la Salud, Toledo, Spain. ^6^Hospital Arnau de Vilanova, Lleida, Spain. ^7^Virgen del Rocio, Sevilla, Spain. ^8^Hospital Infanta Elena, Huelva, Spain. ^9^Hospital Virgen de la Luz, Cuenca, Spain. ^10^Hospital Carlos Haya, Malaga, Spain. ^11^Hospital Clinico San Carlos, Madrid, Spain. ^12^Hospital Vall d´Hebron, Barcelona, Spain. ^13^Hospital Santa Creu i Sant Pau, Barcelona, Spain. ^14^Hospital de Cabuenes, Gijon, Spain. ^15^Hospital de la Fe, Valencia, Spain. ^16^Hospital Clinico, Valencia, Spain. ^17^Hospital Joan XXIII, Tarragona, Spain. ^18^Hospital de Albacete, Albacete, Spain. ^19^Hospital de Valme, Sevilla, Spain. ^20^Hospital del SAS, Jerez de la Frontera, Spain. ^21^Hospital Santa Lucia, Cartagena, Spain. ^22^Hospital General, Granollers, Spain. ^23^Hospital Infanta Cristina, Badajoz, Spain

**Background: **Persistence and adherence have been correlated with improved patient outcomes. E/C/F/TAF and DTG+ABC/3TC are the guidelines‐preferred combinations, but the information about their relative real‐world performance is scarce. The aim of this study was to compare real‐world persistence of commonly used triple therapies and risk of discontinuation due to virological failure and adverse events in a large Spanish cohort.

**Materials and methods: **A retrospective analysis was performed using data from the VACH cohort ‐ a prospective multicentre Spanish cohort of adult HIV patients. All treatment‐experienced patients, between 1 August 2016 (introduction of E/C/F/TAF) and 1 June 2017, initiating DTG+ABC/3TC or E/C/F/TAF were included. Unit of analysis was patient‐regimen. Time to non‐persistence was defined as the time from patient‐regimen initiation to discontinuation (for any reason), loss‐to‐follow‐up, death or censoring, whichever occurred first. Time to discontinuation due specifically to virological failure and adverse events, as reported by the clinician, were also studied. Kaplan‐Meier curves and Cox proportional hazard models (controlling for demographics, comorbidities, viral load, CD4, number of previous regimens and years on antiretroviral therapy – all at patient‐regimen initiation) were conducted.

**Results: **A total of 1879 patient‐regimens were included, 1279 on E/C/F/TAF and 600 on DTG+ABC/3TC (96% were on DTG/ABC/3TC). Baseline patient‐regimen characteristics differed in the two groups (Table 1); patients on E/C/F/TAF were younger and were less treatment experienced; the proportion of patients with AIDS and HCV was lower in the E/C/F/TAF group, whereas the proportion of HBV co‐infection was higher. When controlling for differences in patient characteristics, a hazard ratio (HR) of 2.1 (*p* = 0.0002) was observed for discontinuation due to any reason in DTG+ABC/3TC regimens versus E/C/F/TAF. No significant difference was observed for risk of discontinuation due to virological failure (*p* = 0.16) whereas the HR of discontinuation due to AE in DTG+ABC/3TC regimens versus E/C/F/TAF was 3.3 (*p* = 0.001) (Figure 1).

**Table:** Abstract P083 – Table 1. Patient and clinical characteristics at patient‐regimen initiation

	DTG+ABC/3TC (n = 600)	E/C/F/TAF (n = 1279)	*p* value
Age (years), mean (SD)	49.6 (10.5)	46.2 (10.8)	<0.0001
Gender, % male	73.0	76.9	0.064
AIDS diagnosis, % yes	25.0	19.9	0.011
CD4 count at treatment initiation, % <350 cells/microL	14.7	11.6	0.203
Number of previous ART regimens, mean (SD)	4.22 (3.3)	4.03 (3.6)	0.245
Duration of ART regimens (years), mean (SD)	12.2 (7.4)	10.1 (7.4)	<0.0001
HCV, % yes	34.2	26.9	0.001
HBV, % yes	1.6	8.0	<0.0001
VL at treatment initiation, % <50 cells/mm^3^	79.1	84.6	0.003
Previous VF, mean (SD)	0.9 (1.4)	0.7 (1.5)	0.007
eGFR, % <60 min/mL/1.73 m^2^	5.9	4.3	0.206

VF = virological failure; VL = viral load.

**Conclusions: **In this analysis of current triple therapy regimens, persistence remains significantly higher in patients on E/C/F/TAF versus DTG+ABC/3TC, with a three‐fold higher risk of discontinuation due to AE in DTG+ABC/3TC and no differences in discontinuation due to virological failure.

Abstract P083 – Figure 1. Kaplan‐Meier curves for persistence (log‐rank test: *p* < 0.0001).

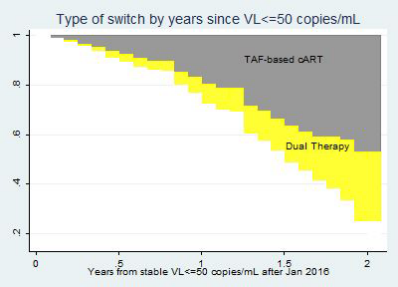

## P084

### Assessing the impact of food insecurity on HIV medication adherence in the context of an integrated care facility for people living with HIV in Vancouver, Canada

**K Koehn^1^, T McLinden^1^, A Collins^2^, P McDougall^3^, R Baltzer‐Turje^3^, C Wang^1^, J Li^1^, K Salters^1^, S Parashar^1^ and R Hogg^1^**

^1^Epidemiology and Population Health Program, British Columbia Centre for Excellence in HIV/AIDS, Vancouver, Canada. ^2^Research, British Columbia Centre on Substance Use, Vancouver, Canada. ^3^Research & Evaluation, Dr. Peter AIDS Foundation, Vancouver, Canada

**Background: **Despite provision of universal HIV care, PLWHIV in British Columbia (BC) may experience substantial barriers to optimal adherence of combination antiretroviral therapy (cART). Food insecurity, or experiences of uncertain or inadequate food access due to limited financial resources, has been associated with poor adherence and remains highly prevalent among PLWHIV [1]. We examined the impact of food insecurity on cART adherence in the context of an integrated care facility that provides multiple services (e.g. art therapy, counseling), including the provision of two meals per day.

**Materials and methods: **Survey data collected between February 2014 and March 2016 from the Dr. Peter Centre (DPC) study and clinical data from BC's HIV treatment program were examined. PLWHIV who were on cART at baseline were included in this analysis. The outcome variable was optimal cART adherence based on pharmacy refill compliance at ≥95%. The primary explanatory variable was food insecurity (food secure vs. food insecure) measured by Health Canada's Household Food Security Survey Module (HFSSM). Adjusted odds ratios (aOR) were estimated by generalized estimating equations, quantifying the relationship between food insecurity and cART adherence with logistic regression. The confounders (illicit drug use, self‐reported anxiety and/or depression, hepatitis C diagnosis, current living status, age and cumulative months on cART) were selected using a change‐in‐estimate approach.

**Results: **This study included 116 PLWHIV at baseline, with 99 participants completing the follow‐up survey after 12 months. At baseline, 74% (n = 86) of participants reported experiencing food insecurity (≥2 affirmative responses on the HFSSM) and 67% (n = 78) were ≥95% cART adherent. The median age was 46 years (IQR 39 to 52), 82% (n = 95) were biologically male at birth and 53% (n = 62) had used illicit drugs in the past six months. In the adjusted analysis, food insecurity was significantly associated with suboptimal cART adherence (aOR 0.47, 95% CI 0.24 to 0.93).

**Conclusions: **The high prevalence of food insecurity among DPC clients was similar to that documented in other Canadian studies of PLWHIV [1]. In addition, participants who were food insecure were approximately half as likely to be adherent to cART. Therefore, while integrated interventions that include food may be effective at reducing hunger or providing entry points to care [2], they may not necessarily address income‐related food insecurity. Future studies that elucidate effective strategies to mitigate food insecurity and poverty among PLWHIV in this integrated care setting are necessary.

**References**

[1] Anema A, Fielden SJ, Shurgold S, Ding E, Messina J, Jones JE, et al. Association between food insecurity and procurement methods among people living with HIV in a high resource setting. PLoS One. 2016;11:1‐20.

[2] Collins AB, Parashar S, Hogg RS, Fernando S, Worthington C, McDougall P, et al. Integrated HIV care and service engagement among people living with HIV who use drugs in a setting with a community‐wide treatment as prevention initiative: a qualitative study in Vancouver, Canada. J Int AIDS Soc. 2017;20:21407.

## P085

### Attention deficit and hyperactivity disorder in HIV‐infected individuals: is it associated with non‐adherence to treatment?

**S Uysal^1^, H Elbi^2^, G Mermut^1^, Ö Önen Sertöz^2^, F Kaptan^3^, D Gulpek^4^ and D Gökengin^1^**

^1^Infectious Diseases and Clinical Microbiology, Ege University Faculty of Medicine, İzmir, Turkey. ^2^Department of Psychiatry, Ege University Faculty of Medicine, İzmir, Turkey. ^3^Infectious Diseases and Clinical Microbiology, Katip Celebi University Atatürk Training and Research Hospital, İzmir, Turkey. ^4^Department of Psychiatry, Katip Celebi University Atatürk Training and Research Hospital, İzmir, Turkey

**Aim: **This study aims to determine the prevalence of attention deficit and hyperactivity disorder (ADHD) among PLWHIV and its association with adherence to ART.

**Methods: **The study group included PLWHIV from the Ege University Hospital and Atatürk Training and Research Hospital cohorts recruited between January 2012 and April 2018. The Ege University Ethical Board approved the study (8/9 November – 22 November 2011). Participants that gave informed consent were subjected to a structured clinical interview (SCID) for DSM‐IV and Hamilton Depression Scale plus a semi‐structured interview for DSM‐IV ADHD. In addition, they self‐completed the State‐Trait Anxiety Inventory (STAI) and the Wender Utah Rating Scale. The total follow‐up time since the HIV diagnosis, total duration of ART, adherence to ART and to scheduled visits, lost‐to follow‐up and survival were recorded for each participant. Participants who had used ART for at least 15 days and had interrupted any of their antiretroviral drugs for at least 3 consecutive weeks or missed any of their drugs for at least 3 consecutive days were defined as Type A non‐adherence. Patients who did not show up for scheduled visits without any excuse at least twice for at least 15 days were defined as Type B non‐adherence. Patients who experienced either or both types of non‐adherence were defined as Type C non‐adherence. Chi‐square and Fisher's exact tests were used to analyse categorical data. Time until Type A, B or C non‐adherence and its association with ADHD were analysed with logrank test and Kaplan‐Meier survival curves.

**Results: **The study included 85 patients; 25 (29.41%) were diagnosed with ADHD which was significantly higher than the highest prevalence (6%) for ADHD among the general population (chi‐square; *p* < 0.001). Overall, the prevalence of Type A, B and C non‐adherence was 23.5%, 22.4% and 31.8%, respectively. The time until Type A, B and C non‐adherence was significantly shorter among cases with ADHD compared to those without.

**Conclusion: **ADHD is significantly more common among PLWHIV than in the general population. ADHD may have a negative effect on adherence to ART and to scheduled visits and should be diagnosed early and managed accordingly.

## P086

### The “Doctor Apollo” chatbot: a digital health tool to improve engagement of people living with HIV

**S Vita^1^, R Marocco^1^, I Pozzetto^2^, G Morlino^3^, E Vigilante^4^, V Palmacci^1^, L Fondaco^2^, B Kertusha^2^, M Renzelli^1^, V Mercurio^5^, V Vullo^6^, C Mastroianni^6^ and M Lichtner^1^**

^1^Infectious Diseases Unit, Santa Maria Goretti, Latina, Italy. ^2^Public Health and Infectious Diseases Dept, Santa Maria Goretti, Latina, Italy. ^3^Business Development Unit, Snapback Srl, Roma, Italy. ^4^Product Development Unit, Snapback Srl, Roma, Italy. ^5^Infectious Diseases Unit, Santa Maria Goretti, Roma, Italy. ^6^Public Health and Infectious Diseases Dept, Sapienza University, Roma, Italy

**Background: **Integration of mobile phone technology into HIV care has been proposed not only to increase ART adherence bur also to facilitate the long‐term follow‐up of people living with HIV. Aim of the project is to design, develop and evaluate a digital health personnel‐patient interface.

**Materials and methods: **The project phases (analysis of needs and expectations, development, enrolments of study population and evaluation) have been conducted with a user‐centred approach. More specifically, focus groups, design thinking and lean programming methodologies, and agile project management have been employed. Inclusion criteria were: own a smartphone, age >18 years old, to be under ART for at least six months. A semi‐structured questionnaire before and after Doctor Apollo use to evaluate the current interaction modalities with the clinical centre and the degree of satisfaction was administered.

**Results: **The needs of HIV people identified were: a diversified level of required privacy, the possibility to receive the results of blood test in real time, have a contact with the centre even when it is closed, and a leaner appointment management. The instant messaging platform chosen to develop and deliver Doctor Apollo has been Telegram. Thirty‐four patients have been enrolled into the study (four declined to sign informed consent for privacy reason). Characteristics of the study population are listed in Table 1. In one month period 216 messages between patients and Doctor Apollo were exchanged, many of those outside working hours. The most used function was “Results” (20.3%), followed by “Book appointment” (18.8%) and “Start” (15.6%) (Figure 1). Unexpectedly, the “Medication reminder” function has been used in only 10% of days.

**Table:** Abstract P086 –Table 1. Characteristics of the study population

Sex	17F, 17M
Age, median (min‐max)	46 (21 to 65)
Heterosexual, n (%)	15 (44%)
Men who have sex with men, n (%)	19 (56%)
CD4 mm^3^, median (min‐max)	527 (170 to 1207)
HIV‐RNA copies/mL	<40

Abstract P086 – Figure 1. Doctor Apollo usage.

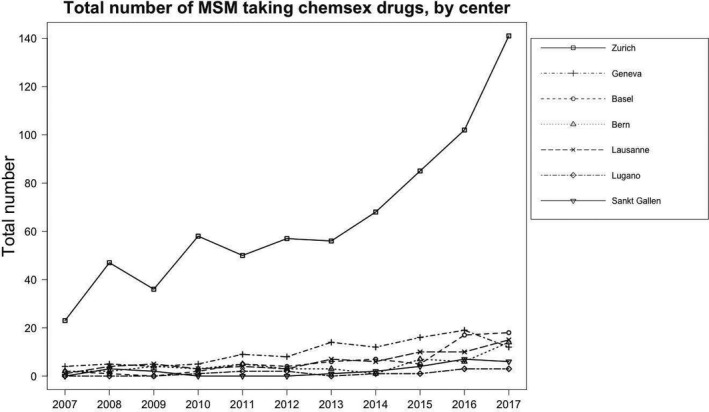

**Conclusions: **A chatbot interface between patient and clinical centre seems to be an effective tool for its flexibility. Respect for privacy was already a major issue for enrolled patients, together with the need to be in continuous close contact with the clinical centre. Apparently, drug reminder is not a need for patients and it is being perceived as invasive. A larger use of Doctor Apollo in different settings is necessary to better understand the role of chatbots in the long‐term management of HIV infection in everyday life.

## P087

### Health resources consumption and persistence in patients with HIV treated with standard of care triple therapy or two‐drug combination regimens

**D Sangiorgi, V Perrone and L Degli Esposti**

Health Economics and Outcomes Research, CliCon Srl, Ravenna, Italy

**Background: **Guideline‐recommended triple therapy (TT) is the standard of care for HIV and consists of a combination of three ARV drugs: a backbone of two nucleotide/nucleoside reverse transcriptase inhibitors (NRTIs) and a third drug of another class. Single tablet regimens (STRs) are a TT in a single pill, administered once a day. Two‐drug combinations (2DC) are alternative, used when optimal TT cannot be used, e.g. due to toxicity or drug‐drug interaction. Our aim was to describe how treatment persistence and resource use vary between these strategies.

**Materials and methods: **An observational retrospective cohort analysis based on three local health units’ administrative databases was conducted. Patients treated with ARVs between 1 January 2012 and 30 June 2015 were included. Patients were segmented into three groups: MTR (at least 2 NRTI + a third drug, but not in STR form), STR (TT in STR form) and 2DC (patients taking any regimen made of only two drugs). Patients without any ARV prescription in the 12 months before the index date were classified as naïve. A Kaplan‐Meier curve was used to analyse persistence in each of the three groups. Adherence was estimated by calculating the ratio between the days covered by the prescribed drug and the total number of therapy days. Average healthcare costs per group were calculated by including the cost of drugs, hospitalisation, outpatient visits, test and laboratory.

**Results: **A total of 568 patients were identified: 392 MTR (mean age 48.0, male 68.6%, naïve 23%), 106 STR (mean age 47.2, male 67%, naïve 38.7%) and 70 2DC (mean age 49.6, male 62.9%, naïve 32.9%). The Kaplan‐Meier curve at three years follow‐up revealed a higher proportion of persistence for STR patients: persistence was 77.3% for MTR, 93.8% for STR and 68.9% for 2DC (*p* = 0.001). Annual expenditure for patient management, including ARV cost, was €10,248 per MTR patient, €9345 per STR patient and €10,844 per 2DC patient. ARV‐related cost per patient was €8528 for MTR, €7918 for STR and €9588 for 2DC. Adherence of ≥95% was found in 59.7% of MTR patients, 78.3% of STR patients and 88.6% of 2DC patients.

**Conclusions: **Treatment persistence was greatest for STR, whilst expenditure was lowest for STR, suggesting that this is the optimal strategy. Conversely, 2DC had the lowest persistence and the highest per patient expenditure, suggesting that the 2DC patients were a carefully selected high‐risk group with very high adherence.

## P089

### Impact of recreational drug use on people living with HIV's health

**V Castro^1^, L Leal^2^, N Garín^3^, J Casado^4^, S Cenoz^5^, A Jaén^6^, M Galindo^7^ and M Fuster‐RuizdeApodaca^1^**

^1^Pharmacy, Hospital de la Marina Baixa, Villajoyosa, Spain. ^2^Infectious Diseases, Hospital Clínico, Barcelona, Spain. ^3^Pharmacy, Hospital de la Santa Creu y Sant Pau, Barcelona, Spain. ^4^Infectious Diseases, Hospital Ramón y Cajal, Madrid, Spain. ^5^Medical Manager, ViiV Healthcare, Madrid, Spain. ^6^Coordinator, Fundació Reserca i Docència Mutua Terrassa, Barcelona, Spain. ^7^Infectious Diseases, Hospital Clínico Universitario, Valencia, Spain. ^8^Management, SEISIDA, Madrid, Spain

**Background: **Use of recreational drugs (RD) may have relevant clinical consequences for PLWHIV. This study explored the impact of RD use on HIV clinical and patient‐reported outcomes.

**Methods: **A multicentric observational retrospective cohort study was conducted between April 2017 and May 2018. PLWHIV on ART for at least one year were included. The sample consisted of two cohorts of PLWHIV according to their RD use: frequent users (consumption ≥1 drug ≥10 times a year, not morphine or methadone, not cannabis as a single drug) versus not users (included consumption cannabis ≤10 times a year). Retrospective last 12‐month clinical data were collected from clinical records. Patient‐reported outcomes were collected through a cross‐sectional online survey, containing items related to drug use, self‐reported health data and use of health services (hospitalisations and emergency care). It also included the following validated measures: ART adherence (CEAT‐VIH), health‐related quality of life (HRQoL) (WHOQoL‐HIV‐bref) and psychological well‐being (GHQ‐12). Differences between drug and non‐drug users were analysed through parametric techniques according to the nature of data. Analyses were performed with SPSS Statistics v.22.

**Results: **A total of 276 participants were included in the study; 146 (52.9%) consumed RD and 130 (47.1%) did not consume them. Differences in the characteristics of both groups are displayed in Table 1. Compared to non‐drug users, drug users obtained lower scores in ART adherence (87.8 ± 9.5 vs. 90.6 ± 6.7; *p* = 0.004) and also in most domains of HRQoL (*p* = 0.005) except in the social relationships domain. The highest difference was found in the psychological health domain (70.2 ± 17.3 vs. 79.0 ± 14.5; *p* = 0.000). They also presented higher scores in depression (*p* = 0.006) and anxiety (*p* = 0.008) and had acquired more sexually transmitted infections (*p* = 0.012) compared to non‐drug users. The number of hospital admissions (*p* = 0.043) and emergency room visits (*p* = 0.016) was significantly higher in the group of drug users.

**Table:** Abstract P089 – Table 1. Differences in the characteristics of PLWHIV who use and who do not use drugs

Variables	Drug users n = 146 (52.9%)	Non‐drug users n = 130 (47.1%)	*p* value
Gender			0.002
Men	144 (98.6%)	115 (88.5%)	
Women	2 (1.4%)	14 (10.8%)	
Transexual	0 (0%)	1 (0.8%)	
Sexual orientation			<0.0001
Heterosexual	8 (5.6%)	29 (22.8%)	
Men who have sex with men	134 (94.4%)	98 (77.2%)	
Years since diagnosis (mean ± SD)	10.9 ± 6.0	13.9 ± 8.8	0.002
Years taking ART (mean ± SD)	9.2 ± 5.8	11.0 ± 7.2	0.028
Age (mean ± SD)	41.3 ± 8.2	48.5 ± 12.0	<0.0001

**Conclusions: **RD use in PLWHIV has a negative impact on health outcome at various levels, including HIV clinical results, HRQoL and the use of health services. Interventions to address problematic drug use and to improve health outcomes of PLWHIV who use drugs should be conducted.

## P090

### Antiretroviral therapy retention times and predictors of retention to care among HIV‐infected patients in Ukraine

**T Kyrychenko**

Outpatient Clinic, Poltava Regional HIV/AIDS Prevention and Control Center, Poltava, Ukraine

**Background: **Studying drug retention times and factors associated with engagement in care of HIV‐infected patients are essential, because HIV infection requires lifelong treatment. Data regarding retention to care of HIV‐positive individuals in Ukraine are scarce. The objective of this study was to assess retention to antiretroviral therapy care and identify factors associated with complete and incomplete retention in a longitudinal cohort of HIV‐infected patients in Ukraine.

**Materials and methods: **We conducted a retrospective cohort study in Poltava, Central Ukraine of 2303 newly diagnosed HIV patients >18 years old who entered care and started antiretroviral treatment from 2004 to 2016 and followed at least 12 months. Retention to antiretroviral therapy care was defined as having at least one visit each three months of care throughout the entire follow‐up period. Potential risk factors associated with dropout during treatment have been identified by using multivariate logistic regression models. SPSS Statistics v.22.0 was used for statistical analysis.

**Results:** ****It has been found that the proportion of HIV‐infected patients being under antiretroviral therapy in the cohort during the observation period was maintained at 66% to 85%. Thus, the analysis of ART monitoring has shown that there was a decrease of staying in antiretroviral care among the patients within 2004 to 2016 in Poltava region (from 85% among patients under treatment during one year to 66% among patients receiving ART 12 years), and the AIDS‐death level in patients receiving ART increased (from 7% among patients receiving ART during one year to 20% among patients being under ART during 12 years). Retention in care during ART treatment was best predicted by attending clinic of integrated services with access to opioid replacement therapy (OR 1.1, 95% CI 1.0 to 1.1), social support (OR 1.4, 95% CI 1.0 to 2.1) and evidence of previous tuberculosis (TB) treatment (OR 1.2, 95% CI 1.1 to 2.8). The risk for discontinuing in care was significantly higher in HIV‐infected patients who injected drugs and women who diagnosed during the pregnancy (OR 1.6, 95% CI 1.1 to 1.8; OR 1.9, 95% CI 1.5 to 2.3). Engaged HIV‐positive patients had significantly better mean quality of life scores than unengaged patients in the physical (72.08 ± 21.13 vs. 47 ± 23.14, *p* = 0.013) and psychological domains (77.12 ± 13.03 vs. 52.22 ± 17.12, *p* = 0.023).

**Conclusion: **The main factors associated with complete retention to care among HIV‐positive patients under ART in Ukraine were good access to integrated services clinics with opioid replacement therapy, social support, high quality of life scores in the physical and psychological domains, evidence of previous TB treatment.

## P091

### The influence of education and employment on ART adherence among PLWHIV aged 25 to 44 in Tashkent: an exploratory research

**D Karimov**

Infectious and Pediatric Infectious Diseases, Tashkent Medical Academy, Tashkent, Uzbekistan

**Background: **At present, there are no reliable data on the level of ART adherence among PLWHIV in Tashkent. Because of unemployment and a low level of education, the influence of social factors on ART adherence is highly relevant. This study was conducted to analyse the relationship between social factors such as employment, education and ART adherence. The study aims to develop recommendations for improving ART adherence in key populations.

**Materials and methods: **The study was conducted at the AIDS Center of Tashkent from August to December 2017 and included 146 HIV‐positive patients. A cluster method for classifying patients by adherence, based on questionnaires, and a retrospective analysis of outpatient card data were used. The patients were surveyed on the basis of the questionnaire which included questions about missed doses, compliance with the time of medicine intake, the connection of taking medications with food intake. Patients had to answer each question by writing “yes” or “no”. Each answer “yes” was estimated from 1 to 3 points. Then the points were summed up and all the respondents were categorised according to the World Health Organization classification of ART adherence: 24 to 23 points (≥95%), good adherence (GA); 22 to 21 (85 to 94%), insufficient adherence (IA); 20 and below (≤84%), low adherence (LA). The participants were divided by the level of education into two groups: having higher education/students in the university (group 1A) and without education (group 1B); and by their employment status: officially working (group 2A) and officially unemployed patients (group 2B). Participants in each group were divided into clusters.

**Results: **The average age was 29.7 years. Seventy‐nine (54.1%) patients were female and 67 (45.9%) male. In the group 1A (n = 60): people with GA 75.0% (n = 45), IA 16.6% (n = 10), LA 8.3% (n = 5); and in the group 1B (n = 86): GA 55.8% (n = 48), IA 24.4% (n = 21), LA 19.8% (n = 17), respectively (*p* < 0.05). The clusters in the group 2A (n = 102): GA 70.6% (n = 72), IA 21.5% (n = 22), LA 7.8% (n = 8); and in the group 2B (n = 44): GA 52.3% (n = 23), IA almost a third (29.5%, n = 13), LA 15.9% (n = 7), respectively (*p* < 0.05). The percentage of people with LA in the group of officially unemployed patients is almost two times higher than in the group of employed PLWHIV (*p* = 0.05).

**Conclusion: **This study showed that education and employment have a positive impact on ART adherence among PLWHIV. Patients with higher education were more likely to adhere to ART than patients without higher education. The higher level of education helped them to understand the purposes of the therapy much better, and consequently, to be more committed. Patients in employment were more likely to adhere to ART than unemployed patients who are probably less responsible for their health and do not lead disciplined lifestyle. It is recommended to open the “Patient's School” and create favourable conditions for employing PLWHIV in Tashkent.

## P092

### Analysis of impediments to the maintenance in care of people with HIV in Italy

**A Battistella^1^, M Errico^2^, B Rossetti^3^, E Pontali^4^, M Bassetti^5^, M Lichtner^6^, M Rizzi^7^, V Manfrin^8^, A Gori^9^, B Celesia^10^, C Viscoli^11^, A Comelli^12^, G Angarano^13^, A Lazzarin^14^, A Castagna^14^, G Borgia^15^, B Menzaghi^16^, G Nunnari^17^, R Corsini^18^, L Calza^19^, F Vichi^20^, L Fontanelli Sulekova^21^, A Giannetti^22^, G Magnani^23^, A d'Arminio Monforte^24^, M Iardino^25^ and G Rizzardini^26^**

^1^Research Center, NPS Italia Onlus, Milano, Italy. ^2^President, NPS Italia Onlus, Milano, Italy. ^3^Infectious Diseases, Azienda Ospedaliera Universitaria Senese, Siena, Italy. ^4^Infectious Diseases, Ospedali Galliera, Genova, Italy. ^5^Infectious Diseases, A.O.U. Santa Maria della Misericordia, Udine, Italy. ^6^Infectious Diseases, Ospedale Santa Maria Goretti, Latina, Italy. ^7^Infectious Diseases, Azienda Ospedaliera Papa Giovanni XXIII, Bergamo, Italy. ^8^Infectious Diseases, Ospedale Civile S. Bortolo, Vicenza, Italy. ^9^Infectious Diseases, Ospedale Maggiore Policlinico, Milano, Italy. ^10^Infectious Diseases, Ospedale Garibaldi Presidio Ospedaliero Nesima, Catania, Italy. ^11^Infectious Diseases, Ospedale San Martino, Genova, Italy. ^12^Infectious Diseases, Spedali Civili di Brescia, Brescia, Italy. ^13^Infectious Diseases, Università degli Studi di Bari, Policlinico, Bari, Italy. ^14^Infectious Diseases, IRCCS San Raffaele, Milano, Italy. ^15^Infectious Diseases, Azienda Ospedaliera Universitaria Federico II, Napoli, Italy. ^16^Infectious Diseases, Ospedale di Circolo, Busto Arsizio, Italy. ^17^Infectious Diseases, Policlinico G. Martino, Messina, Italy. ^18^Infectious Diseases, Arcispedale S. Maria Nuova, Reggio Emilia, Italy. ^19^Infectious Diseases, Policlinico Sant'Orsola‐Malpighi, Bologna, Italy. ^20^Infectious Diseases, USL Centro, Firenze, Italy. ^21^Infectious Diseases, Policlinico Umberto I, Roma, Italy. ^22^Infectious Diseases, IINMI Lazzaro Spallanzani, Roma, Italy. ^23^Infectious Diseases, Ausl Reggio Emilia, Reggio Emilia, Italy. ^24^Presidente, Fondazione Icona, Milano, Italy. ^25^Partner, NPS Italia Onlus, Milano, Italy. ^26^Infectious Diseases Unit n.1, Fatebenefratelli Sacco, Milano, Italy

**Background: **In Italy, the phenomenon of non‐retention in care among HIV+ people has been so far monitored, but still not analysed in its predictive elements.

**Methods: **This research analysed some organisational aspects of infectiology centres and some others related to individual characteristics of HIV+ people in care, to identify possible predictive elements of a potential lack of retention in care. Data refer to 23,491 HIV+ patients, in 18 infectiology centres in 10 representative regions of North‐Central and Southern Italy. The analysis covered the period between 2010 and 2017. Clinicians and nurses were asked to take questionnaires for investigating, on their opinions, about possible predictive elements of lack of retention in care; questionnaires were answered by 32 infectious diseases doctors and 55 nurses in centres which joined the project.

**Results: **It emerged that the issue of non‐retention in care concerns particularly immigrant people, from European and extra‐European countries, whereas Italian people in care tend to rank low “lost to follow‐up” rates. A critical element has instead emerged according to young patients, under 30 years of age, who ranked 10% of “lost to follow‐up” rates (Figure 1). This appears to be certainly high, especially considering their mobility among treatment centres. Age and origin of patients, however, even in the absence of research data that could highlight certain correlations, seem to be elements that can be reasonably linked to each other, considering the type of patients in care. On the other way, gender was found not to affect retention in care any way. It also emerged how dimension of centres affects the retention of patients; it is easier to lose patients in small centres rather than in the larger ones. Figures 2 and 3 show consistent differences, concerning individual elements of patients, that represent an experiential datum shared by clinicians and nurses.

**Conclusions: **The research underlined a cognitive element that so far has not been registered on the relationship between patients’ young age and risk of fail the retention in care. It also highlighted how new tools for tracing patients’ mobility among centres, a significant phenomenon that makes quantitative data more difficult to interpret, should be considered for a further detailed analysis.

Abstract P092 – Figure 1. HIV+ people lost in follow up, by origin in %.

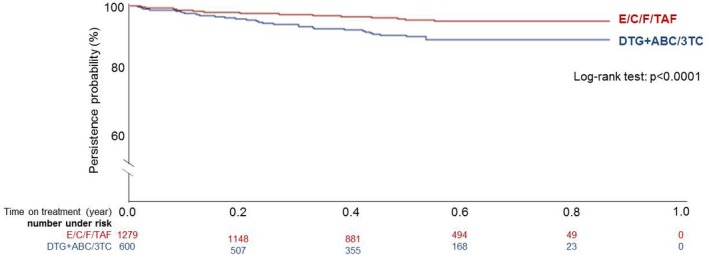

Abstract P092 – Figure 2. Individual predictive characteristics of a lack of retention in care.

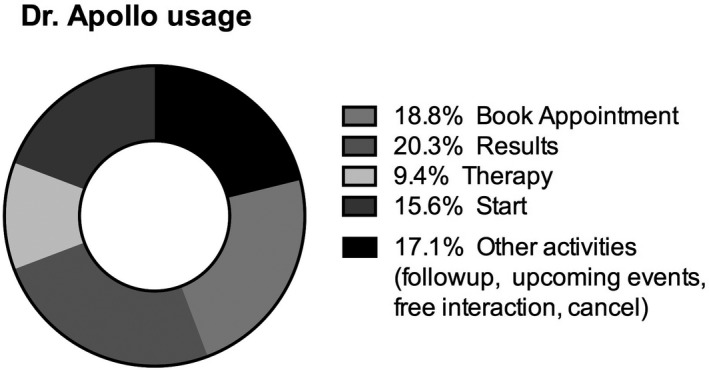

Abstract P092 – Figure 3. Predictive life situations of a lack of retention in care.

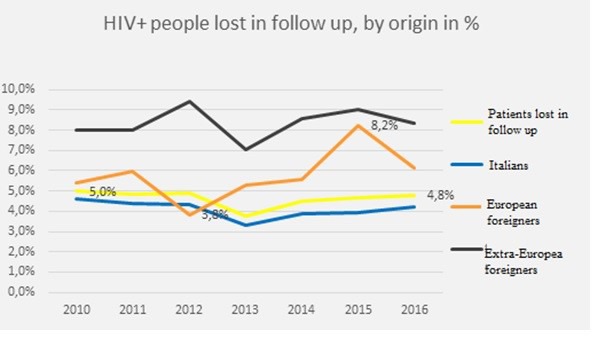

**Reference: **[1] Analysis of impediments to the maintenance in care of people with HIV, NPS Italia Onlus, 2018.

## TREATMENT STRATEGIES: SIMPLIFICATION

## P093

### Efficacy of intermittent short cycles of integrase inhibitor‐based maintenance antiretroviral therapy in virologically suppressed HIV patients

**R Calin, S Landowski^2^, R Agher^3^, M Marcou^2^, C Blanc^3^, M Valantin^3^, F Caby^4^, C Katlama^3^ and P De Truchis^2^**

^1^Infectious Diseases, AP‐HP, Tenon Hospital, Paris, France. ^2^Infectious Diseases, AP‐HP, Raymond‐Poincaré Hospital, Garches, France. ^3^Infectious Diseases, AP‐HP, Pitié‐Salpêtrière Hospital, Paris, France. ^4^Infectious Diseases, Hospital Center D'Argenteuil, Argenteuil, France

**Background: **Several studies (FOTO, BREATHER, ANRS162‐4D) have shown that NNRTI‐ or PI‐based triple therapy could be safely administered as a 5 days (5D) or 4 days (4D) a week maintenance strategy. We report here our experience of using an integrase inhibitor‐based 5D/4D intermittent regimen in virologically suppressed HIV patients.

**Materials and methods: **This cohort study from two clinical centres has enrolled all patients >18 years old, on ART with VL <50 for more than one year, who switched to an INSTI‐based triple regimen given 4D/5D a week. HIV‐RNA and CD4‐count were assessed at 6, 12, 18, 24 months (M) and thereafter. Primary endpoint was the virological efficacy rate at M12, with virological failure defined as confirmed VL >50 copies/mL.

**Results: **A total of 49 patients were included between January 2008 and December 2017, with the following baseline characteristics at time of switch: 35 men (71%), median (IQR) age 50 (44 to 55) years, time since HIV diagnosis 13 (8 to 22) years, CD4 count nadir 214/mm^3^ (77 to 312), ART duration 10 (6 to 17) years, duration of viral suppression with VL <50 copies/mL 3 (1.5 to 7) years. ART regimen consisted of two NRTIs plus either EVG/c in 45% (n = 22), DTG in 35% (n = 17) or RAL in 16% (n = 8) of patients. Two patients had a triple therapy with DTG+NNRTI+NRTI. Forty (81.6%) and nine (18.4%) patients had a 4D and 5D regimen, respectively. As of 30 June 2018, the median follow‐up under intermittent regimen was 23 (12 to 33) months; all patients have reached the M6 visit, 38 (77%) patients M12, 29 (59%) M18 and 26 (53%) M24. Two patients discontinued the strategy for the following reasons: virological failure (n = 1) at M6 and switch for renal toxicity in one other patient. Virological failure occurred under ABC/3TC/RAL therapy with the emergence of the N155H resistance mutation. HIV‐RNA was resuppressed under TDF/FTC/DRV/r. All remaining patients were still suppressed under INSTI intermittent therapy until the last visit. Overall the rate of virological success was 98% (95% CI 89% to 99%) at M6 (n = 49), 97% (95% CI 86% to 99%) at M12 (n = 38) and 96% (95% CI 81% to 99%) at M24 (n = 26).

**Conclusion: **Our results suggest that the use of an intermittent maintenance triple drug regimen given as week‐end (2 or 3 days)‐off is as effective with an INSTI‐based regimen as with PI or NNRTI. The large randomised QUATUOR ANRS‐170 study will bring a definite answer on whether this drug‐reducing strategy is a safe option in HIV‐infected patients.

## P094

### DOLAM study: effectiveness and safety of a dual therapy with dolutegravir plus lamivudine in treatment‐experienced HIV patients

**C Hidalgo‐Tenorio^1^, S De Jesus^1^, L López Cortés^2^, J Santos^3^, M Gomez^4^, S Ferra^5^, C Garcia Vallecillos^1^ and J Pasquau^1^**

^1^Infectious Disease Service, University Hospital Virgen de las Nieves, Granada, Spain. ^2^Infectious Disease Service, University Hospitals Virgen del Rocío, Sevilla, Spain. ^3^Infectious Disease Service, University Hospital Virgen de la Victoria, Malaga, Spain. ^4^Infectious Disease Unit, Complejo Hospitalario Jaen, Jaen, Spain. ^5^Infectious Disease Unit, Hospital Torrecardenas, Almería, Spain

**Background: **Dual therapies (DT) with lamivudine (3TC) plus a protease inhibitor (OLE, SALT and DUAL trails) or raltegravir, an integrase inhibitor [1], have proven to be as effective as triple therapy as a simplification strategy. Dolutegravir (DTG) is an integrase inhibitor that has proven to be effective in dual therapy with rilpivirine (SWORD). The objective of this study was to analyse the effectiveness and safety of the DT with 3TC+DTG in real‐life treatment‐experienced HIV patients.

**Methods: **Observational, retrospective, multicentre study of treatment‐experienced HIV patients, with over six months of previous ART, who switched to 3TC+DTG for any reason before 30 June 2017. Viral loads (VL) during exposure to the DT were analysed and the percentage of VL <50 copies/mL at 24 weeks was calculated. The adverse events were also notified.

**Results: **One hundred and seventy‐eight HIV+ patients were included, with a median age of 50 years, 77.5% were men, with a CD4 nadir of 182 cells/uL, 15 years since diagnosis of HIV and 13 years since the initiation of ART, with five previous treatment combinations. 96.6% had a baseline VL <50 copies/mL and a CD4 lymphocyte count of 674 cells/uL. Of the 178, 20 (11.2%) did not complete the follow‐up for the following reasons: six due to adverse events, seven clinical decisions (five patients initiated anti‐HCV treatment, another had drug‐drug interactions and another switched back to triple therapy after adding abacavir), two patients wished to switch back to their previous STR (on Week 12 and 24), two were lost to follow‐up and three patients died of non‐related causes (lung carcinoma, oesophagus carcinoma, decompensated liver cirrhosis). Of the 158 patients who completed the follow‐up, 151 (95.6%) had VL <50 copies/mL and seven had VL >50 copies/mL. Of the seven with detectable VL, three were confirmed as virological failures (one at Week 4 and the other two at Week 24) and the other four were classified as blips. Overall, the efficacy of 3TC+DTG at 24 weeks was 98.1%. Of the three patients who presented virological failure, only one drug resistance test was carried out successfully, detecting the mutations K103R and S147G (both mutations that do not limit integrase inhibitor activity).

**Conclusion: **These data suggest that the combination of lamivudine plus dolutegravir in treatment‐experienced and virologically stable patients is a new and attractive simplification strategy for antiretroviral treatment, as effective as the triple therapy.

**Reference: **[1] Cuchetto G, et al. J Antimicrob Chemother. 2017.

## P095

### Long‐term follow‐up of dolutegravir as single antiretroviral agent in patients with suppressed HIV viraemia

**G Tebano^1^, L Schneider^1^, C Soulié^2^, C Blanc^1^, S Seang^1^, M Valantin^1^, R Tubiana^1^, A Marcelin^2^ and C Katlama^1^**

^1^Infectious Diseases Department, Pitié‐Salpêtrière Hospital, AP‐PH, Sorbonne University, UPMC Univ Paris 06, INSERM, IPLESP (UMRS 1136), Paris, France. ^2^Department of Virology, Pitié‐Salpêtrière Hospital, AP‐PH, Sorbonne University, UPMC Univ Paris 06, INSERM, IPLESP (UMRS 1136), Paris, France

**Background: **HIV‐infected patients are exposed to decades of antiretroviral treatment. Drug‐reduced regimens (dual therapy/monotherapy) are increasingly investigated to reduce drug exposure and long‐term toxicity. Dolutegravir (DTG) has been evaluated in monotherapy, due to its potency, long half‐life and high genetic barrier to resistance. However, concerns have been raised about emergence of resistance in case of virological failure. We report here our experience with long‐term follow‐up in patients receiving DTG monotherapy.

**Materials and methods: **This is an ongoing single centre, observational cohort study, that included all HIV‐1 positive/HBV‐negative patients who had been switched to monotherapy with DTG 50 mg once daily (mono‐DTG), after a period of viral suppression ≥six months (VS, <50 copies/mL), with no virological failure on any integrase inhibitor (INI)‐based regimen. Outcomes include rate of VS at Week (W)12, W24, W48, W96; time to VS loss; resistance profile in case of rebound.

**Results: **A total of 63 patients had been switched to mono‐DTG between May 2014 and June 2018. At time of switch, baseline characteristics (median [IQR]) were: ART duration, 15 years [6 to 20]; viral suppression, six years [3 to 10]; CD4 count, 679 [487 to 847]. ART regimen consisted in: PI monotherapy, 24/63 (38%); dual therapy, 17/63 (27%); triple therapy, 22/63 (35%). Twenty‐five of 63 (40%) had been exposed to INIs and 14/63 (22%) were on an INI‐based regimen at time of switch. Overall median duration of mono‐DTG was 18 months [6 to 30]. Thirty‐nine patients (62%) are still on mono‐DTG, while 21 (33%) had been switched to dual/triple therapy for reasons other than virological failure: decision to intensify treatment (11/21, 52%); side effects (5/21, 24%), blips with 54 and 67 copies/mL (2/21, 10%); other reason (3/21, 14%). Overall VS was maintained in 98% of patients at W12 (61/62, 95% CI 91 to 99); 95% at W24 (52/55, 95% CI 85 to 99); 100% at W48 (43/43, 95% CI 92 to 100); 95% at W96 (21/22, 95% CI 77 to 100). Virological failure occurred in three patients (5%, 95% CI 1 to 13): at W12 (n = 1) and W24 (n = 2), with emergence of INI resistance (previously reported [1]). All have been exposed to INIs, including one having INI resistance‐associated mutation retrospectively detected on baseline HIV‐DNA genotype resistance.

**Conclusion: **Although DTG is not recommended as monotherapy, long‐term follow‐up is reassuring in terms of sustainability of efficacy in a population with a long duration of viral suppression and mostly on drug‐reduced regimen prior to switch to mono‐DTG. Predictive factors of success with drug‐reduced strategies should be investigated to optimise treatment individualisation.

**Reference: **[1] Katlama C, Soulié C, Caby F, Denis A, Blanc C, Schneider L, et al. Dolutegravir as monotherapy in HIV‐1‐infected individuals with suppressed HIV viraemia. J Antimicrob Chemother. 2016;71:2646‐50.

## P096

### Switching to the combination of dolutegravir plus rilpivirine as dual therapy in the clinical setting: a prospective cohort study

**M Monsalvo Hernando^1^, M Fontecha^1^, M Vivancos^1^, M Rodriguez‐Sagrado^1^, A Moreno^1^, M Pérez‐Elías^1^ and J Casado^1^**

^1^Infectious Diseases, Ramón y Cajal, Madrid, Spain. ^2^Microbiology, Ramón y Cajal, Madrid, Spain

**Background: **Switching to the combination of dolutegravir (DTG) plus rilpivirine (RPV) could be useful in hard‐to‐treat patients. We evaluate the safety and efficacy of this dual regimen in patients with toxicity or drug‐drug interactions, a population not included in clinical trials.

**Methods: **Prospective cohort study of suppressed HIV‐infected patients, without previous failure to integrase inhibitors or non‐nucleoside, who switched to this combination because of toxicity or interactions (EC 280/15; NCT02491242).

**Results: **Overall, 102 patients (mean age 54 years, 29 women, 28%) were included and followed for a median of 25.7 months (208.2 patient‐years). Median time of HIV infection was 252.7 months (170 to 297) and 57 patients were HCV co‐infected, with fibrosis 4‐cirrhosis in 47% (27 cases, 14 with previous liver decompensation, one liver transplantation). They had received a mean of 6.1 regimens (1 to 11, in 39 with primary mutations against NRTIs or/and PIs), and were receiving an effective regimen (56 TDF, 34 PI‐based, 50 NNRTI‐based) for 52 months. At Week 48, only one patient had virological failure (HIV RNA level 1.93 log, CD4/CD8 ratio 0.2). Six additional patients (6%) left the regimen (three CNS symptoms, one pregnancy, one diarrhoea due to metformin, one alcoholism). Thus, efficacy was 93% at Week 48 (ITT‐e, Snapshot analysis; 96% PP analysis). Lipid parameters improved (total cholesterol ‐2%, CT/HDL ratio ‐6%; *p* = 0.03). There was a significant decrease in eGFR (−8.4 mL/min, −18.2 to +2.2), independently from previous TDF, but urine parameters improved or did not change (uricosuria; *p* = 0.03). The CD4/CD8 ratio improved from 0.76 to 0.8 (*p* = 0.22). DXA scan in 89 patients showed improvement in spine in 72 patients (mean +1.15%; −0.57 to +3.3%) and in femoral neck in 56 patients (mean +0.4%; −3.3% to +2.57%), associated to previous TDF. Two additional patients discontinued the regimen before Week 96 (one non‐adherent with virological failure and emergence of E138A mutation; one who need prolonged omeprazole).

**Conclusion: **Switching to the combination of dolutegravir plus rilpivirine is effective and safe in patients with toxicity or interactions, even in patients with cirrhosis. Our data corroborate the improvement in renal function as measured by urine parameters, in spite of a moderate eGFR worsening.

## P097

### Higher rate of virological failure in women compared to men when starting dual antiretroviral treatment: sex‐specific analysis from the Frankfurt HIV Cohort

**B Koenigs^1^, M Bickel^2^, S Goepel^1^, P Gute^2^, E Herrmann^3^, P de Leuw^1^, G Schuettfort^1^, C Stephan^1^, T Wolf^1^ and A Haberl^1^**

^1^HIV Center, University Hospital Frankfurt, Frankfurt, Germany. ^2^Infektiologikum Frankfurt, Frankfurt, Germany. ^3^Institute of Biostatistics & Mathematical Modeling, University Frankfurt, Frankfurt, Germany

**Background: **Even though dual ART is not recommended for naive patients yet there is a need for dual nuke‐sparing regimen in pretreated patients. The total number of dual ART will more probably increase in the near future since the first fixed‐dose INSTI/NNRTI combination has been licensed in November 2017. Nevertheless, the typical characteristics of patients on dual ART in clinical everyday life remain unclear. Our study aims to create a profile of patients on dual ART under special consideration of possible sex‐specific differences.

**Method: **All patients of the Frankfurt HIV Cohort receiving dual ART between January 2008 and January 2015 were included in a retrospective study. In this sub‐analysis we looked at patients who stayed on the dual regimen for at least one year. Primary study objective: virological response defined as viral load <50 copies/mL after 12 months of treatment. Secondary objectives: sex‐specific differences in baseline characteristics, retroviral regimen, virological and immunological response.

**Results: **In the observational period 547 patients, 415 men (75.9%) and 132 women (24.1%), received dual ART. Complete sets with baseline and 12 months data were available for 341 patients, 258 men (75.7%) and 83 women (24.3%). Baseline data of these patients is shown in Table 1. Women were more likely to be of African origin (*p* = 0.000), and a higher rate of female than male patients had a detectable viral load at baseline (*p* = 0.0113). After one year on dual ART 53 women (63.9%) and 188 men (72.9%) had an undetectable viral load (n.s.). The three most common dual combinations in men were INSTI+PI (50.8%), PI+PI (27.2%) and CCR5 antagonist+PI (15.5%); in women INSTI+PI (43.4%), PI+PI (33.7%) and CCR5 antagonist+PI (18.1%). The virological response rate in patients treated with PI+PI (45.9%) was lower compared to the other combinations (70.7%).

**Table:** Abstract P097 – Table 1. Baseline data

Baseline data	Male patients, N = 258	Female patients, N = 83	All patients, N = 341
Age (mean), N = years	49.7	43.7	48.2
African origin, n (%)	22 (8.5%)	49 (34.9%)	51 (15.0%)
Time on ART, years	10.6	10.5	10.6
CD4 count (mean), n/µL	301	364	322
Viral load <50 copies/mL, n (%)	114 (44.1%)	23 (27.7%)	137 (40.2%)

**Conclusions: **In this analysis of 341 pretreated patients starting dual ART virological failure at baseline was more common in women (72.3%) than in men (55.9%). This is contrary to the prevalent opinion that women switch ART predominantly due to tolerability reasons. Even though after one year on dual ART virological response rates were similar in men (72.9%) and women (63.9%) clinicians should consider possible sex differences in ART outcome.

## P098

### Determinants and outcomes of the choice to switch to dolutegravir within different three‐ or two‐drug regimens in a single‐centre cohort: the DOLUTILITY study

**S Restelli^1^, F Romeri^2^, M Piscaglia^1^, D Rizzelli^2^, I Gallazzi^1^, L Paladini^1^, M Cossu^1^, V Micheli^2^ and A Capetti^1^**

^1^1st Division of Infectious Diseases, ASST Fatebenefratelli Sacco, Milano, Italy. ^2^Clinical Microbiology Laboratory, ASST Fatebenefratelli Sacco, Milano, Italy

**Background: **Dolutegravir (DTG) was approved for antiretroviral therapy mainly on data from triple association with two nucleosides; however, very early physicians started to use it within different regimens, including two‐drug regimens [1,2]. This analysis aims to investigate whether various DTG‐based regimens have their own specificity and to describe them.

**Materials and methods: **The DOLUTILITY study is a single‐centre part of the ODOACRE cohort. For all the patients who started DTG in any combination from 10 November 2014 to 30 April 2017, we collected baseline demographic, pharmacological, virological, immunological and metabolic data, routine clinical data and blood work and outcomes. Only those subjects who had started DTG at least 96 weeks before the analysis (30 April 2016) were included. The statistical analysis is based on the Mann‐Whitney test and the Wilcoxon test for continuous variables and on Fisher's exact test for contingency.

**Results: **One thousand and thirty‐nine patients were included. The six regimens that were studied are: abacavir/lamivudine/DTG (ABC/3TC/DTG), n = 614, DTG plus 3TC, n = 47, DTG plus rilpivirine (RPV), n = 132, DTG plus boosted darunavir (bDRV), n = 95, DTG plus boosted/unboosted atazanavir (b/uATV), n = 59 and DTG plus tenofovir/emtricitabine (TFV/FTC), n = 92. Table 1 summarises the baseline demographic and epidemiologic characteristics. Overall, DTG+bDRV and DTG+TFV/FTC had longer time from HIV diagnosis and longer time on therapy (*p* < 0.0001 for both), while DTG+bDRV and DTG+RPV had more CDC stage C diagnosis and history of treatment failure (*p* < 0.0001 for both), DTG+RPV had often been chosen for concomitant treatment of HCV and HBV co‐infection was present only in the DTG+TFV/FTC group. The analysis of past exposure to antiretrovirals and baseline and historical resistance‐associated mutations (RAMs) revealed that DTG+bDRV and DTG+RPV had the heaviest burden, while DTG+3TC was only slightly affected. Table 2 describes the main reasons for the switch and their statistical relevance, compared to the choice for ABC/3TC/DTG. All the regimens showed >92% efficacy and the few viral failures (12 overall) were not accompanied by the selection of new mutations.

**Table:** Abstract P098 – Table 1. Demographic and epidemiologic determinants of the treatment groups

Companion drug(s)		ABC/3TC	3TC	RPV	bDRV	b/uATV	TFV/FTC
Sex	M	485 (79%)	34 (72%)	85 (64%)	61 (64%)	34 (58%)	73 (79%)
	F	128 (21%)	13 (28%)	47 (36%)	44 (36%)	25 (42%)	19 (21%)
Ethnicity	Caucasian	500 (81%)	42 (89.4%)	121 (91.7%)	80 (84.2%)	47 (79.7%)	82 (89.1%)
	African	35 (5.7%)	3 (6.4%)	9 (6.8%)	4 (4.2%)	5 (8.5%)	2 (2.2%)
	Asian	11 (1.8%)	2 (4.2%)	2 (1.5%)	0	0	1 (1.1%)
	Hispanic/Latino	67 (10.9%)	0	0	6 (6.3%)	6 (10.1%)	
	Other	4 (0.6%)	0	0	5 (5.3%)	1 (1.7%)	1 (1.1%)
Risk factor	Heterosexual	215 (35%)	19 (40.5%)	46 (34.8%)	37 (38.9%)	22 (37.3%)	20 (21.6%)
	Male having sex with males	264 (43%)	20 (42.5%)	43 (32.7%)	19 (20%)	18 (30.5%)	35 (38.3%)
	Intravenous drug addiction	129 (21%)	8 (17%)	41 (31%)	38 (40%)	19 (32.2%)	37 (40.1%)
	Other	6 (1%)	0	2 (1.5%)	1 (1.1%)	0	0
Mean age, years	49.7	39	47	56.5	54.5	50.3

**Table:** Abstract P098 – Table 2. Reasons to switch to each specific treatment group

ABC/3TC/DTG vs	DTG+3TC	DTG+RPV	DTG+bDRV	DTG+b/uATV	DTG+TFV/FTC
Simplification	*p* < 0.0001 favours ABC/3TC/DTG	*p* < 0.0001 favours ABC/3TC/DTG	*p* = 0.26	*p* < 0.0001 favours ABC/3TC/DTG	*p* < 0.0001 favours ABC/3TC/DTG
Proactive switch	*p* < 0.0001 favours DTG+3TC	*p* < 0.0001 favours DTG+RPV	*p* < 0.0001 favours DTG+bDRV	*p* < 0.0001 favours DTG+b/uATV	*p* < 0.0001 favours DTG+TFV/FTC
Toxicity	*p* < 0.0001 favours DTG+3TC	*p* = 0.136	*p* < 0.0001 favours ABC/3TC/DTG	*p* = 1	*p* = 0.269
Virological failure	*p* = 0.6197	*p* < 0.0001 favours DTG+RPV	*p* < 0.0001 favours DTG+bDRV	*p* = 0.0009 favours DTG+b/uATV	*p* = 0.176
Adherence	*p* < 0.0001 favours ABC/3TC/DTG	*p* < 0.0001 favours ABC/3TC/DTG	*p* < 0.0001 favours ABC/3TC/DTG	*p* < 0.0001 favours ABC/3TC/DTG	*p* = 0.877
Drug‐drug interactions	*p* < 0.0001 favours DTG+3TC	*p* < 0.0001 favours DTG+RPV	*p* = 1	*p* = 1	*p* < 0.0001 favours DTG+TFV/FTC

**Conclusions: **ABC/3TC/DTG being the main choice, DTG/3TC is the choice for subjects with cardiovascular risk, short drug experience and few or no mutations, DTG/RPV for drug‐experienced subjects who retain sensitivity to both drugs and need such regimen to avoid or correct metabolic problems, DTG/bDRV is a regimen for salvage or simplification of salvage, while DTG/b/uATV and DTG/TFV/FTC have intermediate profiles.

**References**

[1] Capetti AF, Cossu MV, Paladini L, Rizzardini G. Dolutegravir plus rilpivirine dual therapy in treating HIV‐1 infection. Expert Opin Pharmacother. 2018;19:65‐77.

[2] Maggiolo F, Gulminetti R, Pagnucco L, Digaetano M, Benatti S, Valenti D, et al. Lamivudine/dolutegravir dual therapy in HIV‐infected, virologically suppressed patients. BMC Infect Dis. 2017;17:215. 

## P099

### Ethical challenges to maintaining or stopping ART at the end of life: implication for HIV cure research

**J Routy^1^, R Ponte^2^, R Reinhard^3^, J Chen^2^, N Chomont^4^, P Ancuta^4^, M Jenabian^5^, C Costiniuk^6^, E Cohen^7^, J Estaquier^8^, J Angel^9^ and B Lebouché^10^**

^1^Chronic Viral Illness Service and Hematology, Research Institute of the McGill University Health Centre, Montréal, Canada. ^2^Chronic Viral Illness Service, Research Institute of the McGill University Health Centre, Montréal, Canada. ^3^University of Toronto and Canadian HIV Cure Enterprise, (Can CURE), Montreal, Canada. ^4^Immunopathologie, CR‐CHUM and Universite de Montreal, Montréal, Canada. ^5^Universite du Quebec a Montreal, and BIOMED, Montréal, Canada. ^6^Chronic Viral Illness Service, McGill University Health Centre, Montréal, Canada. ^7^Laboratory of Human Retrovirology, Montreal Clinical Research Institute, Montréal, Canada. ^8^Infectious and Immune Diseases, Universite Laval and Centre de Recherche du CHU de Quebec, Quebec, Canada. ^9^Biochemistry, Microbiology and Immunology, University of Ottawa, Ottawa, Canada. ^10^Family Medicine, Research Institute of the McGill University Health Centre, Montréal, Canada

**Background: **Although ART is the standard of care for PLWHIV, viral persistence represents a barrier to cure. PLWHIV who knowingly expect end of life face difficult choices with regards to ART continuation. PLWHIV must weigh pill burden and toxicity during a time of emotional complexity. Recent efforts to study viral persistence in tissues only accessible through autopsy raise an alternative decision factor of altruism. Individuals may wish to donate tissues/organs where HIV remains suppressed on ART, allowing identification of anatomical sites of viral persistence. The possibility under Canadian law to opt for Medical Assistance in Death provides a potential for research autopsy to yield important data under controlled conditions. Ethical questions of seeking permission for research autopsy and conditions of ART maintenance have led our group to begin a wide‐ranging discussion.

**Materials and methods: **Meetings were organized in Ottawa, Montreal and Vancouver with clinicians, virologists, ethicists and community spokespersons from Canada. Participants were asked to examine the merits of sustaining ART until close to the time of death. Participants were also questioned about strategies to better inform patients and physicians on options related to research on viral persistence after autopsy.

**Results: **Arguments in favor of maintaining ART at the end of life were: (1) most ART combinations are well tolerated even at times close to death, (2) to reduce complications associated with stopping ART such as retroviral syndrome, (3) participating in research on HIV persistence may be an opportunity to give back to the community. Negative arguments included: (1) to improve quality at the end of life by alleviating ART toxicity and pill burden, (2) to mitigate accumulated feelings of stigma, (3) to maintain ART for research purposes even when treatment benefits are no longer viable.

**Conclusions: **Researchers interested in obtaining tissues following autopsies for the purpose of understanding viral persistence need to develop approaches in regards to continuation of ART at the end of life. A coordinated stakeholder dialog should address informed consent and values of personal and social contribution throughout the life course, including its final moments.

## TREATMENT STRATEGIES: SWITCH STUDIES

## P100

### Incidence and determinants of antiretroviral switching away from TDF‐based backbone in recent years in the Icona Foundation Cohort

**A Vergori^1^, P Lorenzini^1^, A Cozzi Lepri^2^, F Maggiolo^3^, G Lapadula^4^, A De Luca^5^, A Cingolani^6^, M Galli^7^, G Mazzarello^8^, P Milini^9^, A D'Arminio Monforte^10^, A Antinori^1^, on behalf of Icona Foundation Study Group**

^1^HIV/AIDS Unit, National Institute for Infectious Diseases, L.Spallanzani, IRCCS, Rome, Italy. ^2^Institute for Global Health, University College London, London, UK. ^3^Division of Infectious Diseases, ASST Papa Giovanni XXIII, Bergamo, Italy. ^4^Division of Infectious Diseases, ASST di Monza University of Milano‐Bicocca, Monza, Italy. ^5^University Division of Infectious Diseases, Siena University Hospital, Siena, Italy. ^6^Institute of Clinical Infectious Diseases, Policlinic A. Gemelli Catholic University of the Sacred Heart, Rome, Italy. ^7^3rd Division of Infectious Diseases, ASST FB‐Sacco University of Milan, Milan, Italy. ^8^Infectious Disease Clinic, IRCCS University Hospital, San Martino‐IST, Genoa, Italy. ^9^Infectious Diseases Unit, Macerata General Hospital, Macerata, Italy. ^10^Clinic of Infectious and Tropical Diseases, ASST Santi Paolo e Carlo, University of Milan, Milan, Italy

**Background: **In the past years, NRTI backbone based on tenofovir disoproxil fumarate (TDF) and emtricitabine (FTC) has represented the standard of care in the antiretroviral setting. Increasing concerns about TDF renal and bone toxicity and availability of effective and safe alternatives, as abacavir (ABC), tenofovir alafenamide (TAF) or NRTI‐sparing less‐drug regimens (LDRs), enhances chances of TDF substitution. Rate and predictors of TDF discontinuation may have public health implications in the view of availability of TDF/FTC generic formulation.

**Materials and methods: **Patients from the Icona Foundation Cohort initiating their first cART regimen with TDF‐based backbone plus a third drug from January 2009 onwards were included. As primary endpoint, incidence rate (IR) for 100 PYFU of TDF discontinuation for any reasons was calculated. The adjusted risk of TDF discontinuation was estimated by Cox regression according to main interest covariates.

**Results: **Five thousand, five hundred and forty‐four ART‐naïve included: 81% males, median age 39 years; 2296 (41%) started a NNRTI‐based, 2015 (36%) a PI/b‐based and 1233 (22%) an INSTI‐based regimen. Two thousand five hundred and forty‐six discontinued TDF after a median of 2.3 years (IQR 1.1 to 3.9). IR of TDF discontinuation for any reason significantly increased from 10.3 (95% CI 9.5 to 11.1) per 100 PYFU in 2009 to 2011, to 14.3 (13.4 to 15.2) in 2012 to 2014 and to 34.9 (32.7 to 37.7) in 2015 to 2017 (*p* for trend <0.001). Using NNRTI as reference, an increased risk of TDF discontinuation was found both for PI/b (aHR 1.58; 95% CI 1.43 to 1.74) and INSTI (1.99; 1.77 to 2.24) and also for DTG (2.41; 2.02 to 2.87), EVG/c (2.05; 1.77 to 2.37) and RAL (1.64; 1.36 to 1.97), separately. An increased risk was found also for boosted drugs (aHR 1.40; 95% CI 1.28 to 1.52). In Figure 1, IRs of TDF discontinuation by current eGFR in the three time periods were displayed. By multivariable Cox regression, a lower current eGFR was associated to a higher risk of discontinuing TDF in all time periods, whereas non‐communicable comorbidities (NCC) were only variably and marginally associated (Table 1). The risks of TDF discontinuation with specific INSTI drugs or boosted regimens in the three time periods is summarised in Table 1.

**Abstract P100 – Figure 1. Adjusted hazard ratios of TDF discontinuation according to calendar year of TDF initiation and current eGFR estimated by CKD‐EPI formula.**

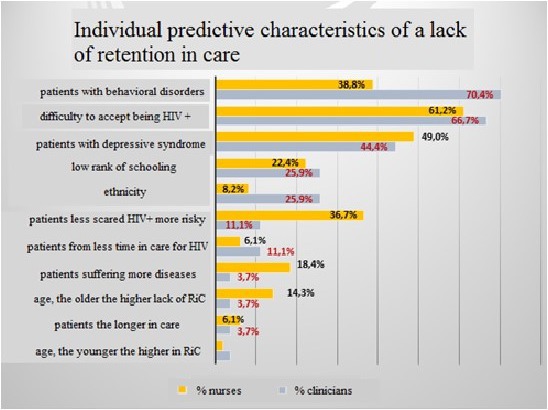

**Table:** Abstract P100 – Table 1. Factors independently associated to TDF discontinuation according to three time periods (2009 to 2011; 2012 to 2014; 2015 to 2017) of cART initiation. Significant results are reported in bold

	2009 to 2011	2012 to 2014	2015 to 2017
	aHR^a^ (95% CI)	aHR^a^ (95% CI)	aHR^a^ (95% CI)
Current eGFR mL/min/1.73 m^2^ (by CKD‐EPI)			
>90 (ref.)	1.00	1.00	1.00
60 to 90	**1.81 (1.53** to **2.13)**	**1.53 (1.33** to **1.75)**	**1.20 (1.04** to **1.39)**
<60	**5.18 (3.61** to **7.42)**	**4.85 (3.61** to **6.52)**	**1.84 (1.26** to **2.70)**
NCC			
0 (ref.)	1.00	1.0	1.00
1	**1.44 (1.05** to **1.96)**	1.21 (0.92 to 1.57)	0.97 (0.72 to 1.30)
2+	1.47 (0.69 to 3.15)	**1.87 (1.10** to **3.18)**	1.10 (0.61 to 1.96)
ARV third drug class			
NNRTI (ref.)	1.0	1.0	1.0
PI/b	**1.64 (1.39** to **1.94)**	**1.33 (1.15** to **1.53)**	**1.38 (1.10** to **1.75)**
INSTI	1.07 (0.67 to 1.72)	**1.58 (1.27** to **1.97)**	**2.11 (1.77** to **2.51)**
ARV third drug			
NNRTI (ref.)	1.0	1.0	1.0
PI/b	**1.64 (1.39** to **1.94)**	**1.33 (1.15** to **1.53)**	**1.41 (1.11** to **1.77)**
DTG	2.72 (1.00 to 7.42)	1.36 (0.70 to 2.64)	**2.65 (2.13** to **3.28)**
EVG/c	2.81 (0.69 to 11.47)	**1.86 (1.34** to **2.59)**	**1.93 (1.61** to **2.33)**
RAL	0.83 (0.47 to 1.46)	**1.46 (1.10** to **1.95)**	**2.01 (1.50** to **2.69)**
ARV third drug by booster use			
Unboosted drugs (ref.)	1.0	1.0	1.0
Boosted drugs	**2.02 (1.70** to **2.40)**	**1.35 (1.18** to **1.55)**	1.14 (0.99 to 1.32)

^a^multivariable model adjusted for gender, age, black race, mode of HIV transmission, race, CDC stage, HCV‐Ab, baseline CD4 count, baseline CD8 count, baseline HIV‐1 RNA, total and HDL cholesterol, current eGFR, NCC, ARV third drug class. NCC = non‐communicable comorbidities (diabetes, hypertension, cerebrovascular and cardiovascular disease, end stage liver disease).

**Conclusions: **In our cohort, a significant increase of TDF discontinuation was found after 2015. Associated drugs (PI/b and INSTI) and eGFR decline mainly predicted drug change, with a lower risk of switching away from TDF at declining eGFR levels in the last period. The remarkable risk of TDF switching in people receiving INSTI, increasingly in the last three years, may suggest physicians’ attitudes towards coformulated regimens more than TDF safety concerns in clinical decision.

## P101

### Patient‐reported outcomes after switching to a two‐drug regimen of dolutegravir + rilpivirine: Week 100 results from the SWORD‐1 and SWORD‐2 studies

**A Oglesby^1^, K Angelis^2^, Y Punekar^3^, S Lopes^3^, A Antela^4^, M Aboud^5^, E Blair^6^, L Kahl^7^, M Gartland^6^, B Wynne^8^ and M Murray^31^**

^1^Health Outcomes, ViiV Healthcare, Research Triangle Park, NC, USA. ^2^Statistics, GlaxoSmithKline, Uxbridge, UK. ^3^Health Outcomes, ViiV Healthcare, Brentford, UK. ^4^Infectious Diseases Unit, Hospital Clinico de Santiago, La Coruna, Spain. ^5^Global Medical Affairs, ViiV Healthcare, Brentford, UK. ^6^Research and Development, ViiV Healthcare, Research Triangle Park, NC, USA. ^7^Research and Development, ViiV Healthcare, Brentford, UK. ^8^Research and Development, ViiV Healthcare, Collegeville, PA, USA

**Background: **The SWORD‐1 and SWORD‐2 studies previously demonstrated non‐inferior efficacy and modest improvements in patient‐reported symptom bother and treatment satisfaction at 48 weeks after switching virologically suppressed HIV‐1 infected adults from their current three‐/four‐drug antiretroviral regimen (CAR) to the two‐drug regimen (2DR) of dolutegravir + rilpivirine on Day 1 (ES DTG + RPV group). Longer‐term Week 100 data demonstrated that a high level of viral suppression was maintained in the ES DTG + RPV group. This abstract reports the pooled SWORD‐1/2 results of patient‐reported outcomes (PRO) measures through Week 100.

**Materials and methods: **HIV Treatment Satisfaction Questionnaire (HIVTSQ) and Symptom Distress Module (SDM) were secondary endpoints in the SWORD trials. The EQ‐5D‐5L measure of general health status was assessed as an exploratory endpoint. Change from baseline was calculated for the ES DTG + RPV subjects (over 100 weeks) and CAR subjects (over 48 weeks). Subjects randomized to CAR switched to DTG + RPV at Week 52 (LS DTG + RPV group) and change from LS baseline (i.e. last pre‐switch assessment) was calculated. Patients were also asked their reasons for participating in the study at baseline. For HIVTSQ, high scores represent greater treatment satisfaction (range 0 to 60). SDM was assessed using the symptom bother score with low values indicating less symptom bother (range 0 to 80). Maximum utility score of 1 for EQ‐5D‐5L indicates perfect health.

**Results: **Low levels of symptom burden and a high degree of treatment satisfaction were reported at baseline. Despite this, 27% of subjects cited concern about long‐term side effects of CAR as a reason for participating in SWORD‐1/2. ES DTG + RPV subjects reported modest improvements from baseline in both symptom burden and overall treatment satisfaction in all visits through Week 100 (Table 1). Among the LS DTG + RPV group, there was little change in symptom burden but similar improvement in treatment satisfaction. Baseline health status was high (EQ‐5D mean utility: 0.96) and remained stable (mean change: −0.01 [95% CI −0.02 to 0.00]) at both Weeks 48 and 100 (n = 509) in ES DTG + RPV group.

**Table:** Abstract P101 – Table 1. Change from baseline/LS baseline in symptom bother score and HIVTSQ total score by study visit

	Visit	ES DTG + RPV			CAR			LS DTG + RPV		
		Day 1 to Week 100 (N = 513)			Day 1 to Week 48 (N = 511)			Week 52 to Week 100 (N = 477)		
		n	Mean (SD)	95% CI	n	Mean (SD)	95% CI	n	Mean (SD)	95% CI
SDM ‐ bother score	Baseline	446	9.6 (10.0)	434	11.0 (11.2)	NA	NA
	Week 4	436	−2.9	−3.56 to −2.17	426	−1.3	−2.02 to −0.58	NA	NA
	Week 24	442	−1.8	−2.59 to −0.99	433	−1.5	−2.32 to −0.68	NA	NA
	Week 48 / LS baseline	442	−1.5	−2.23 to −0.71	432	−0.7	−1.57 to 0.17	409	10.3 (11.0)
	Week 56	442	−1.7	−2.56 to −0.90	NA	NA	402	−2.1	−2.82 to −1.29
	Week 76	442	−1.3	−2.10 to −0.47	NA	NA	408	−0.8	−1.54 to 0.00
	Week 100	442	−1.1	−1.95 to −0.25	NA	NA	408	−0.6	−1.48 to 0.23
HIVTSQ ‐ total score	Baseline	513	54.4 (6.4)	507	53.9 (6.6)	NA	NA
	Week 4	503	1.4	0.81 to 1.88	499	0.1	−0.35 to 0.55	NA	NA
	Week 24	509	1.8	1.20 to 2.32	506	0.2	−0.32 to 0.72	NA	NA
	Week 48 / LS baseline	509	1.5	0.90 to 2.12	505	0.4	−0.13 to 0.93	477	54.3 (7.2)
	Week 56	509	1.4	0.73 to 2.06	NA	NA	471	1.1	0.51 to 1.76
	Week 76	509	1.6	0.92 to 2.20	NA	NA	475	1.3	0.57 to 1.97
	Week 100	509	1.5	0.86 to 2.10	NA	NA	475	1.4	0.71 to 2.01

Baseline/LS baseline values are actual values. Week X rows show changes from baseline/LS baseline.

**Conclusion: **High treatment satisfaction levels and low symptom burden were observed in patients entering the study which were slightly improved and maintained 100 weeks after switching to DTG + RPV. Similar results for CAR subjects switching to DTG + RPV at Week 52 were observed through Week 100. These results corroborate DTG + RPV as a well‐tolerated 2DR alternative treatment option in patients with suppressed viral load while on other three‐/four‐drug regimens and no previous virologic failure.

## P102

### Switch to dolutegravir from a boosted protease inhibitor associated with significant weight gain over 48 weeks in NEAT‐022, a randomised 96‐week trial

**L Waters^1^, L Assoumou^2^, S Rusconi^3^, P Domingo^4^, M Gompels^5^, S de Wit^6^, F Raffi^7^, C Stephan^8^, J Rockstroh^9^, C Katlama^10^, G Behrens^11^, J Gatell^12^, A Pozniak^13^ and E Martinez^14^**

^1^GU/HIV Medicine, Central and North West London NHS Foundation Trust, London, UK. ^2^INSERM ‐ Unit of Clinical Epidemiology, Institut Pierre Louis d’Épidémiologie et de Santé Publique, Paris, France. ^3^Infectious Diseases Unit, University of Milan, Milan, Italy. ^4^Department of Medicine, Hospital de Sant Pau, Barcelona, Spain. ^5^Immunology, North Bristol NHS Trust, Bristol, UK. ^6^Department of Infectious Diseases, Centre Hospitalier Universitaire Saint‐Pierre, Brussels, Belgium. ^7^Department of Infectious Diseases, University of Nantes, Nantes, France. ^8^Department of Infectious Diseases, Klinikum der Goethe Universitat, Frankfurt, Germany. ^9^Department of Medicine, University of Bonn, Bonn, Germany. ^10^Department of Infectious Diseases, Pitie‐Salpetriere Hospital, Paris, France. ^11^Department for Clinical Immunology & Rheumatology, Hannover Medical School, Hannover, Germany. ^12^Department of Medicine, University of Barcelona, Barcelona, Spain. ^13^Department of HIV Medicine, Chelsea & Westminster Hospital, London, UK. ^14^Infectious Diseases Unit, University of Barcelona, Barcelona, Spain

**Background: **Several trials and cohorts report weight gain on integrase inhibitors. SPRING‐1, a Phase IIb trial, demonstrated numerically greater weight gain on dolutegravir (DTG) versus efavirenz; weight was not recorded in Phase III DTG trials. NEAT‐022, an open‐label, randomised trial, compared efficacy, safety and change in lipids when switching from ritonavir‐boosted protease inhibitor (PI/r) to DTG‐based therapy in patients with high cardiovascular risk. We performed a post‐hoc weight analysis.

**Materials and methods: **Four hundred and fifteen patients aged ≥50 years or with Framingham risk >10%, suppressed on PI/r‐based cART, were randomised: 205 to immediate switch to DTG (DTG‐I), 210 to deferred switch to DTG at Week (W)48 (DTG‐D). After 96 weeks, DTG‐I and DTG‐D arm participants had received DTG for 96 and 48 weeks, respectively. Primary endpoints (published) demonstrated non‐inferior virological efficacy and significant lipid improvements in the DTG‐I arm at W48. We calculated mean body mass index (BMI, kg/m^2^) and weight (kg) change over 48/96 weeks (DTG‐I vs. DTG‐D arm) and change within arms. BMI/weight change over time and associated baseline factors were estimated using mixed models with random effects; mixed models were used to compare BMI slopes between arms.

**Results: **Baseline median BMI was 25.9 (IQR 23.7 to 28.3). Mean BMI change from W0 to W48 was +0.272/+0.064 in the DTG‐I/DTG‐D arms, significant for DTG‐I but not DTG‐D (*p* = 0.003/0.471 respectively); difference between arms was statistically significant (*p* = 0.008). From W48 to W96 mean BMI change was −0.002/+0.332 on DTG‐I/DTG‐D arms, significant for DTG‐D but not DTG‐I (*p* = 0.984/0.004 respectively); difference between arms was statistically significant (*p* = 0.002). Median weight change was statistically significant for W0 to 48 (+0.82 kg in DTG‐I vs. +0.25 kg in DTG‐D; *p* = 0.008) and W48 to 96 (+0.03 kg in DTG‐I vs. +0.98 kg in DTG‐D; *p* = 0.002). In multivariable analysis, baseline factors associated with higher BMI gain on DTG were Framingham risk >15% (*p* = 0.042) and high blood pressure (*p* = 0.035), while protective factors were switching from PIs other than darunavir or atazanavir (*p* = 0.032), current smoking (*p* = 0.006), daily exercise (*p* = 0.036) and HDL‐cholesterol (*p* < 0.001). After adjustment for baseline BMI, switching from darunavir was the only independent factor associated with BMI gain (*p* = 0.018).

**Conclusions: **In virologically suppressed patients with high cardiovascular risk, significant weight/BMI gain occurred over the first 48 weeks of switch from PI/r to DTG. Risk was higher switching from darunavir compared to other protease inhibitors. These findings confirm weight gain as a potential result of integrase inhibitor‐based therapy; our findings warrant further analyses of other randomised trials and studies to elucidate body mass composition changes on DTG, and the pathogenesis of weight gain.

## P103

### Soluble CD14 levels decrease after switching from a dual regimen with 3TC+PI/r to 3TC+DTG in virologically suppressed HIV‐infected patients

**F Lombardi^1^, S Belmonti^1^, A Borghetti^1^, S Lamonica^1^, A Ciccullo^1^, C Picarelli^1^, G Baldin^1^, A Emiliozzi^1^, D Moschese^1^, A Dusina^1^, M Fabbiani^2^, Roberto Cauda^1^ and S Di Giambenedetto^1^**

^1^Institute of Clinical, Infectious Diseases, Catholic University of Sacred Heart, Rome, Italy. ^2^Department of Infectious Diseases, Fondazione IRCCS Policlinico San Matteo, Pavia, Italy

**Background: **Understanding the effects of different antiretroviral regimens on HIV‐related residual systemic inflammation is a topic of interest because heightened inflammation and immune activation have been associated with morbidity and mortality in virologically suppressed patients. The aim of our study was to evaluate the impact of switching treatment from a dual regimen with ritonavir‐boosted protease inhibitors (PI/r) plus lamivudine (3TC) to dolutegravir (DTG) + 3TC on a marker of monocyte activation, soluble CD14 (sCD14), two markers of inflammation, interleukin‐6 (IL‐6) and C‐reactive protein (CRP) and a marker of coagulation, D‐dimer.

**Materials and methods: **We performed a retrospective case‐crossover study on integrase‐naïve patients with virological suppression (HIV‐RNA <50 copies/mL) while on 3TC + PI/r dual therapy for ≥48 weeks who switched to 3TC + DTG and maintained this regimen for ≥48 weeks. Plasma levels of sCD14, IL‐6, CRP and D‐dimer were tested by standardised ELISA assays on stored samples at three time points: at switch (BL), 48 weeks before (−48W) and 48 weeks after switch (+48W). We performed a mixed model for repeated measures to evaluate the changes in biomarkers over time.

**Results: **A total of 67 patients were included. At BL 73% were males, median age was 49 (interquartile range [IQR] 41 to 55) years, time on ART was 11 (IQR 4 to 16) years, CD4 count was 700 (IQR 571 to 920) cells/μL, nadir CD4 count was 237 (IQR 64 to 306) cells/μL, zenith HIV‐RNA was 4.8 (IQR 4.4 to 5.4) log10 copies/mL. For 64% of the patients, the previous Pl/r regimen was based on darunavir, for 27% on atazanavir and for 9% on lopinavir. The main reason for switching was dyslipidaemia. The evolution of biological parameters over time is shown in Table 1. Mean sCD14 levels were stable from −48W to BL (from 6.05 to 6.03 log10 pg/mL, *p* = 0.422) but showed a statistically significant decrease after switch: from 6.03 (95% CI 5.99 to 6.05) at BL to 5.96 (95% CI 5.92–5.98) log10 pg/mL at W48 (*p* < 0.001). The levels of IL‐6, CRP and D‐dimer remained stable before and after the switch. An improvement in lipid profile (total cholesterol and triglycerides) was observed after the switch to 3TC + DTG compared to 3TC + PI/r. No variation in comorbidities (HCV co‐infection, diabetes, hypertension) occurred over time.

**Table:** Abstract P103 – Table 1. Evolution of biological parameters over time

	−48 W	BL	*p* (between −48W and BL)	+48 W	*p* (between BL and +48 W)
Current CD4 count (cells/µL), median (IQR)	6894 (559 to 860)	700 (571 to 920)	0.248	730 (600 to 895)	0.233
Viral load <50 copies/mL, n (%)	67 (100)	67 (100)		67 (100)	
Lipid profile (mg/dL), median (IQR)					
Total cholesterol	208 (186 to 244)	208 (176 to 246)	0.684	183 (162 to 219)	<0.001
HDL	45 (37 to 56)	46 (36 to 55)	0.359	46 (35 to 59)	0.821
LDL	130 (102 to 161)	122 (99 to 156)	0.321	109 (92 to 146)	0.113
Triglycerides	147 (106 to 221)	137 (107 to 218)	0.695	107 (70 to 160)	<0.001
CD4/CD8, median (IQR)	0.77 (0.61 to 1.00)	0.83 (0.70 to 1.00)	0.174	0.85 (0.67 to 1.07)	0.085
Log10 IL‐6 pg/L, mean (SD)	3.25 (0.30)	3.18 (0.31)	0.141	3.18 (0.31)	0.897
Log10 sCD14 pg/mL, mean (SD)	6.05 (0.13)	6.03 (0.14)	0.422	5.96 (0.15)	<0.001
Log10 CRP pg/mL, mean (SD)	7.15 (0.43)	7.07 (0.50)	0.593	6.96 (0.44)	0.098
Log10 D‐dimer pg/mL, mean (SD)	5.39 (0.40)	5.46 (0.40)	0.429	5.39 (0.41)	0.147

**Conclusions: **In virologically suppressed HIV‐infected patients on a 3TC + PI/r dual therapy, switching to 3TC + DTG was associated with a significant decline in sCD14. These data suggest reduced monocyte activation following substitution of boosted PI with DTG, which could have important implications for morbidity and mortality.

## P104

### Dolutegravir + lamivudine dual therapy in patients with suppressed HIV‐RNA: long term virological and immunological results of a multicentre cohort

**F Maggiolo^1^, L Comi^1^, R Gulminetti^2^, L Pagnucco^2^, M Digaetano^3^, E Di Filippo^1^, D Valenti^1^, P Lorenzini^4^ and C Mussini^3^**

^1^Infectious Diseases, ASST Papa Giovanni XXIII, Bergamo, Italy. ^2^Infectious Diseases, Fondazione IRCCS Policlinico San Matteo, Pavia, Italy. ^3^Infectious Diseases, University of Modena, Modena, Italy. ^4^Infectious Diseases, National Institute for Infectious Diseases L. Spallanzani, Roma, Italy

**Objectives: **Although cART is usually based on the use of three drugs, availability of new potent drugs allows the study of less‐drug regimens and their durability. 

**Methods: **Prospective, multi‐centre, cohort study in patients on stable cART, with a confirmed (>6 months) viraemia <50 copies/mL, absence of M184V mutation or HBsAg. Patients switched to a dual DTG + 3TC regimen and were prospectively monitored. Survival analysis (Kaplan‐Meier curves) and mixed model analysis were performed.

**Abstract P104 – Figure 1.** CD8 + CD38 + HLA*DR+ cells variation over time in a subgroup of 85 patients.

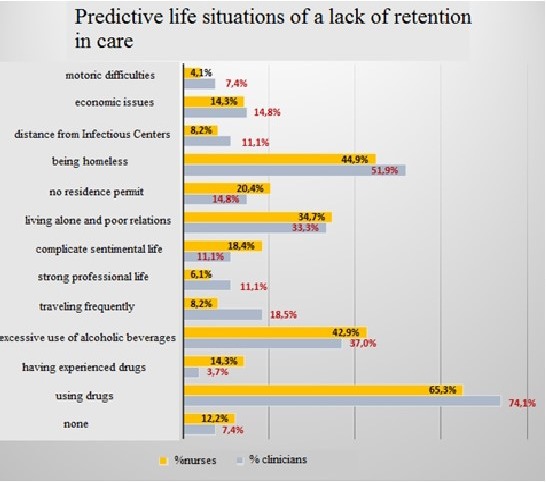

**Results: **We enrolled 218 patients, 75% males, median age 52 years (IQR 12). At switch, patients were on cART for a median of 10.2 years (IQR 13) and with suppressed VL for a median of 75 (IQR 217) months. They had experienced a median of 3 cART lines (IQR 3). HIV‐RNA was <50 copies/mL in all patients and the median CD4 count was 669 cells/mcL (IQR 446). Patients presented 409 comorbidities of the cardiovascular system (36.7%), bone (34.4%), liver (24.3%), CNS (18.3%), kidney (11.0%) or lipids (21.1%) and glucose (12.4%) homeostasis for which they took 538 chronic therapies. Over 449 patient‐years no virological failure occurred. We observed two viral blips <200 copies/mL (after 6 and 18 months). Treatment was discontinued in 17 (7.8%) subjects because of death (four neoplastic diseases and two cirrhosis), of intolerance (two muscle pain and stiffness, one headache, one dizziness and one increased AST/ALT), patient's decision or lost to follow‐up (two cases each). Mean KM estimate of treatment duration was 3.19 years (95% CI 3.0 to 3.3). Over 24 months the CD4 median increment was of 207 cells/mcL (*p* < 0.0001), while median CD8 counts decreased of 30 cells/mcL (*p* = 0.021) with a substantial maintenance of the CD4/CD8 ratio around 0.9 (median). When the mixed model was applied the monthly CD4 increment was of 4.3 cells/mcL (95% CI 2.4 to 6.2, *p* < 0.0001), while CD8 increased by 2.8 cells/mcL (95% CI −0.04 to 5.6, *p* = 0.054) and the CD4/CD8 ratio increment was 0.004 (95% CI 0.0005 to 0.007, *p* < 0.0001). In a subset of 85 patients we performed a CD8 + CD38 + HLA*DR+ count that decreased from a baseline median 6.3% to a 24‐month value of 3.1% (*p* < 0.0001) (Figure 1). Tolerability was high and besides AEs leading to treatment discontinuation previously described, only the following adverse events not related to DTG‐3TC treatment were reported: myocardial infarction (two), subdural haematoma (one).

**Conclusion: **Dual 3TC + DTG is a safe, effective and durable alternative cART in virologically controlled patients. In a relevant sub‐group of patients, the regimen is not associated with an increased risk of immune‐activation.

## P105

### Improvements in patient‐reported outcomes of dolutegravir (DTG)‐based second‐line treatment compared to lopinavir/ritonavir (LPV/r)‐based treatment: results from the DAWNING study

**M Murray^1^, J Hopking^2^, J Sievers^3^, M Aboud^3^, D Brown^3^, M Nascimento^3^, M Gartland^4^, K Smith^4^ and Y Punekar^3^**

^1^Health Outcomes, ViiV Healthcare, Brentford, UK. ^2^Statistics, GSK, Stockley Park, UK. ^3^Chief Scientific Medical Office, ViiV Healthcare, Brentford, UK. ^4^Chief Scientific Medical Office, ViiV Healthcare, Research Triangle Park, NC, USA

**Objectives: **The DAWNING study has demonstrated superior efficacy of DTG + 2NRTIs compared to lopinavir (LPV/r) + 2NRTIs at Week 48 in HIV‐1 infected adults failing first‐line therapy (HIV‐1 RNA ≥400 copies/mL) of a non‐nucleoside reverse transcriptase inhibitor (NNRTI) + 2 NRTIs (ClinicalTrials.gov: NCT02227238). This abstract reports on the results of the patient‐reported outcome (PRO) measures, from DAWNING, conducted in resource‐constrained settings.

**Methods: **PROs included in the trials were: HIV Treatment Satisfaction Questionnaire (HIVTSQ), Morisky Medication Adherence Scale (MMAS‐8) and the Gastrointestinal Symptom Rating Scale (GSRS). PROs were administered at baseline, Weeks 4, 24 and 48 or study withdrawal. In a post‐hoc analysis, change from baseline in PRO total scores were compared between DTG and LPV/r arms for each measure using last observation carried forward (LOCF).

**Results: **A total of 624 participants, failing a first‐line regimen of NNRTI + 2 NRTIs, were randomised to DTG or LPV/r and treated. Low levels of gastric symptoms, moderate levels of adherence and a high degree of treatment satisfaction were reported at baseline. Treatment satisfaction improved in both study arms with patients on DTG reporting improvement from baseline at all timepoints that reached statistical significance at Week 4 (*p* = 0.009) and Week 48 (*p* = 0.023) compared to LPV/r. At Week 48, gastrointestinal symptoms such as diarrhoea, indigestion, abdominal pain and reflux improved from baseline in the DTG arm and worsened in the LPV/r arm. The difference between the two arms was statistically significant for diarrhoea, indigestion, abdominal pain (*p* < 0.001 for all) and reflux (*p* = 0.005) but not for constipation (*p* = 0.203). At baseline, 44% of patients in both arms reported high adherence. There was a significant difference in the levels of adherence at Week 48 in favour of DTG (*p* = 0.005) (Table 1). The proportion of patients with high adherence at Week 48 increased in both arms with more patients in the DTG arm reporting high adherence compared to the LPV/r arm (67% vs. 56%).

**Conclusion: **Patient‐reported outcomes were maintained or slightly improved after starting DTG compared to LPV/r, with marked improvements in gastrointestinal symptoms which may have led to improved adherence. These results support DTG as a treatment option with improved tolerability after first‐line treatment failure in resource‐constrained settings.

**Table:** Abstract P105 – Table 1. Change from baseline at Week 48 for PROs

	Dolutegravir				Lopinavir			
	Baseline		Week 48		Baseline		Week 48	
	Median (IQR)	Min‐max	Median (IQR)	Min‐max	Median (IQR)	Min‐max	Median (IQR)	Min‐max
HIVTSQ ‐ Total score	57 (18)	9 to 66	5 (14)	−14 to 53	56 (19)	5 to 66	3 (11)	−54 to 45
GSRS ‐ Diarrhoea^a^	1.00 (0.67)	1.0 to 7.0	0.00 (0.33)	−6.0 to 2.7	1.00 (0.67)	1.0 to 5.7	0.00 (0.67)	−4.7 to 6.0
GSRS ‐ Indigestion^a^	1.25 (1.00)	1.0 to 6.0	0.00 (0.50)	−4.3 to 3.8	1.25 (1.00)	1.0 to 6.8	0.00 (0.75)	−4.0 to 4.3
GSRS ‐ Abdominal pain^a^	1.00 (1.00)	1.0 to 5.0	0.00 (0.67)	−3.7 to 3.0	1.00 (1.00)	1.0 to 6.0	0.00 (0.33)	−4.3 to 3.7
GSRS ‐ Reflux^a^	1.00 (0.50)	1.0 to 6.5	0.00 (0.00)	−5.0 to 4.0	1.00 (1.00)	1.0 to 6.0	0.00 (0.50)	−3.5 to 4.0
GSRS ‐ Constipation^a^	1.00 (0.67)	1.0 to 5.7	0.00 (0.00)	−3.7 to 3.0	1.00 (0.67)	1.0 to 5.7	0.00 (0.33)	−4.0 to 3.3

^a^ negative number suggests improvement.

## P106

### Virologic outcome after switching a suppressive HAART to dolutegravir (DTG) with 2NRTIs among HIV‐1 infected patients: potential effects of previous suboptimal therapies or previous virologic failures

**M Sangare^1^, J Baril^2^, A De Pokomandy^3^, C Laprise^4^, R Thomas^5^, M Klein^3^, C Tremblay^6^, Z Greenwald^5^, C Pexos^3^, N Machouf^7^, M Durand^8^ and H Trottier^1^**

^1^Department of Social and Preventive Medicine, Université de Montréal and Sainte Justine University Hospital Center, Montreal, Canada. ^2^Clinique de Médecine Urbaine du Quartier Latin, Centre Hospitalier de l'Université de Montréal, Montreal, Canada. ^3^Chronic Viral Illness Service/Dept Family Medicine, McGill University/Health Centre, Montreal, Canada. ^4^Cancer Epidemiology, Oral Health & Society, McGill University, Montreal, Canada. ^5^Clinique Médicale L'Actuel, Montreal, Canada. ^6^Department Microbiology/Infectious Diseases & Immunology, Université de Montréal/CHUM, Montreal, Canada. ^7^Clinique de Médecine Urbaine du Quartier Latin, Montreal, Canada. ^8^Department of Medicine, Université de Montréal/CHUM, Montreal, Canada

**Background and objective**

Switching antiretroviral regimen in the context of suppressed HIV viral load since at least 6 months but previous history of virologic failure or suboptimal therapy is often desired for simplification or toxicity reasons. However, few studies documented the safety of these switches which remains uncertain with regards to the risk of failure. The objective was to verify the virologic outcome following a switch to DTG + 2NRTIs in patients virologically suppressed for at least 6 months, and to compare whether previous virologic failure or past suboptimal regimen increased the risk of post‐switch virologic failure.

**Materials and methods: **Data collected through regular monitoring of patients living with HIV in the Montreal Cohort were analyzed (n = 10,448 patients). Previous virologic failure was defined as a VL >1000 copies/mL after 16 weeks of therapy, or VL >400 copies/mL after 24 weeks or confirmed (two consecutive) VL >50 copies/mL after 48 weeks or after having been suppressed. Blips were excluded. Suboptimal therapies consisted of a single‐NRTI or 2NRTIs. Cox models were used to estimate the effect of previous virologic failure or suboptimal therapies on post‐switch virologic failure (defined as any confirmed VL >50 copies, or the last VL available >50 copies/mL). Hazard ratios (HR) were adjusted for age at switch.

**Results: **A switch to DTG + 2NRTIs was observed among 1209 patients who had undetectable VLs for ≥6 months before treatment change. These patients had a median age of 50.9 years (IQR 43.4 to 57.9) and CD4 count of 660 cells/µL (IQR 500 to 843), respectively. The median number of previous regimens before switch was 5 (IQR 3 to 9). Among these patients, 496 (41.0%) had pre‐switch experienced at least one virologic failure or suboptimal therapy in their prior antiretroviral treatment history whereas 713 (58.9%) did not. Mean follow‐up time after DTG switch was 531.0 days (SD 321.8) and 592.8 days (SD 316.4) among patients with and without previous virologic failure or suboptimal therapy, respectively. Post‐switch virologic failure was observed in 13 (2.8%) patients with previous virologic failure or suboptimal treatment and 18 (2.6%) patients without previous virologic failure or suboptimal therapies. The crude and adjusted HRs for the association between post‐switch virologic outcomes and previous suboptimal therapies or virologic failure were respectively 1.21 (95% CI 0.59 to 2.48) and 1.57 (95% CI 0.74 to 3.30).

**Conclusion: **In this study, patients with a history of virologic failure or suboptimal therapies did not experience significantly increased risks of virologic failures when switched to DTG + 2NRTIs.

## P107

### Efficacy of rilpivirine‐based regimens as switch therapy from nevirapine‐based regimens in HIV‐infected patients with complete virological suppression: a randomised controlled trial

**P Petchkum^1^, S Sungkanuparp^2^, S Kiertiburanakul^1^ and A Phuphuakrat^1^**

^1^Dept of Medicine, Division of Infectious Diseases, Ramathibodi Hospital, Faculty of Medicine, Bangkok, Thailand. ^2^Faculty of Medicine, Chakri Naruebodindra Medical Institute, Ramathibodi Hospital, Samut Prakan, Thailand

**Background: **Nevirapine (NVP)‐based ART remains to be used in HIV‐infected patients in resource‐limited countries despite its compliance and adverse effect concerns. Rilpivirine (RPV), a newer non‐nucleoside reverse transcriptase inhibitor, could be used as an alternative to NVP in virologically suppressed patients. However, there has been limited experience with switching from NVP‐based to RPV‐based regimens. We aimed to study efficacy and adverse events after ART switching from NVP‐based to RPV‐based regimens.

**Methods: **A randomised controlled non‐inferiority trial was conducted in HIV‐infected patients who received NVP‐based regimens and had undetectable plasma HIV RNA for more than 6 months. Patients were randomised 1:1 to continuation arm (NVP‐based regimens were continued) or switch arm (NVP‐based regimens were switched to RPV‐based regimens). Tenofovir disoproxil fumarate (TDF) plus lamivudine (3TC) or emtricitabine (FTC) remained as backbone of the regimens. Primary endpoint was HIV RNA <40 copies/mL at 24 weeks, with a non‐inferiority margin of 12%. Changes of CD4 cell counts and lipid profiles from baseline were analysed.

**Results: **One hundred and six patients were enrolled. Fifty‐five were in the continuation arm and 51 were in the switch arm. Mean (standard deviation) age was 49.14 (9.2) years and 51.90% were females. Median (interquartile range) baseline CD4 cell counts was 561 (443 to 732) cells/mm^3^. Baseline characteristics including age, gender, CD4 and ART duration were similar between the two groups (*p* > 0.05). At 24 weeks, 53 patients (96.36%) in the continuation arm and 49 patients (96.07%) in the switch arm had virological success. The switch arm was non‐inferior to the continuation arm [efficacy difference 0.29%, 95% CI −6.98% to 7.56%, *p* > 0.999]. Both regimens were generally well tolerated, although one patient developed gastrointestinal adverse events and resumed NVP‐based regimen. A significant decrease in mean total cholesterol was observed in the switch arm (−15.33 mg/dL, 95% CI −24.07 to −0.60, *p* = 0.001).

**Conclusions: **In HIV‐infected patients virologically suppressed with NVP‐based regimens, once‐daily RPV‐based regimens are an alternative switch option. These regimens can maintain virological suppression and decrease total cholesterol. Further study of long‐term efficacy of this switching strategy should be pursued.

## P108

### Patient‐reported outcomes in an observational cohort of adult HIV‐1 positive patients after 48 weeks of treatment of darunavir/cobicistat‐based regimens (TMC114FD1HTX4003: ST.O.RE. study)

**A Antinori^1^, A Gori^2^, D Ripamonti^3^, S Rusconi^4^, N Gianotti^5^, R Maserati^6^, A Muscatello^7^, V Di Cristo^8^, A Castagna^9^, G Rizzardini^10^, A Cattelan^11^, B Menzaghi^12^, G Sterrantino^13^, S Kiros^14^, F Castelli^15^, E Focà^15^, B Saccani^15^, G Orofino^16^, M Farenga^16^, R Cauda^17^, S La Monica^17^, V Vullo^18^, A De Luca^19^, B Rossetti^20^, E Manzillo^21^, C Gioè^22^, B Celesia^23^, M Locatelli^23^, G Madeddu^24^, P Bagella^24^, T Santantonio^25^, S Ferrara^25^, L Cosco^26^, E Pontali^27^, A d'Arminio Monforte^28^, R Curetti^29^, M Andreoni^30^, C Stingone^30^, A Uglietti^31^, R Termini^31^ and D Mancusi^31^**

^1^HIV/AIDS Department, National Institute for Infectious Diseases Lazzaro Spallanzani IRCCS, Roma, Italy. ^2^Infectious Diseases Unit, Fondazione IRCCS Ca’ Granda, Ospedale Maggiore Policlinico, University of Milan, Milano, Italy. ^3^Division of Infectious Diseases, ASST Papa Giovanni XXIII, Bergamo, Italy. ^4^Divisione Malattie Infettive, DIBIC Luigi Sacco, Università degli Studi di Milano, Milano, Italy. ^5^Dipartimento di Malattie Infettive, San Raffaele Scientific Institute, Milano, Italy. ^6^Clinica Malattie Infettive, Fondazione Policlinico San Matteo, Pavia, Italy. ^7^Infectious Diseases, Fondazione IRCCS Ca’ Granda, Ospedale Maggiore Policlinico, Milano, Italy. ^8^Infectious Diseases, DIBIC Luigi Sacco and University of Milan, Milano, Italy. ^9^Unit of Management & ARV Treatment of HIV Infection, Faculty of Medicine and Surgery, Vita‐Salute San Raffaele University and IRCCS San Raffaele Hospital, Milano, Italy. ^10^1st Division of Infectious Diseases, ASST Fatebenefratelli‐Sacco, Milano, Italy. ^11^Division of Infectious and Tropical Diseases, Azienda Ospedaliero‐Universitaria di Padova, Padova, Italy. ^12^Infectious Diseases, Azienda Socio Sanitaria Territoriale della Valle Olona‐Busto Arsizio, Busto Arsizio, Italy. ^13^Division of Tropical and Infectious Diseases, Azienda Ospedaliero‐Universitaria Careggi, Firenze, Italy. ^14^Infectious Diseases, University of Firenze, Firenze, Italy. ^15^Department of Infectious and Tropical Diseases, University of Brescia and Spedali Civili General Hospital, Brescia, Italy. ^16^Unit of Infectious Diseases, Amedeo di Savoia Hospital, Torino, Italy. ^17^Institute of Clinical Infectious Diseases, Catholic University of the Sacred Heart of Roma, Roma, Italy. ^18^Department of Public Health and Infectious Disease, Sapienza University, Roma, Italy. ^19^Department of Medical Biotechnologies, University of Siena, Siena, Italy. ^20^University Division of Infectious Diseases, Siena University Hospital, Siena, Italy. ^21^VIII Divisione di Malattie Infettive, A.O.R.N. Cotugno, Napoli, Italy. ^22^Infectious Diseases Division, Policlinico Universitario, Palermo, Italy. ^23^UOC Malattie Infettive, ARNAS “Garibaldi”, Catania, Italy. ^24^Unit of Infectious Diseases, University of Sassari, Sassari, Italy. ^25^Department of Clinical and Experimental Medicine, University of Foggia, Foggia, Italy. ^26^Unit of Infectious Diseases, Pugliese‐Ciaccio Hospital, Catanzaro, Italy. ^27^Infectious Diseases, Ospedali Galliera, Genova, Italy. ^28^Dipartimento di Scienze della Salute, ASST Santi Paolo e Carlo, Milano, Italy. ^29^Clinica Malattie Infettive e Tropicali, ASST Santi Paolo e Carlo, Milano, Italy. ^30^Department of Medicine of Systems, University of Roma “Tor Vergata”, Roma, Italy. ^31^Medical Affairs, Janssen‐Cilag SpA, Cologno Monzese, Italy

**Background: **Current ART for the treatment of HIV‐1‐infected patients has the objective to provide control of viral load while simplifying drugs’ administration. Darunavir/cobicistat (DRV/c) is a fixed‐dose combination including the protease inhibitor (PI) DRV and its booster cobicistat, developed to reduce pill burden, making the PI easier to take and possibly avoiding mistakes in drug administration. The ST.O.RE. study was designed to prospectively collect clinical practice data on effectiveness of ART DRV/c‐based and to assess also the patients’ satisfaction and actual symptoms by means of two validated patient‐reported outcomes (PROs) questionnaires: HIV Treatment Satisfaction (HIV‐TSQ) and HIV Symptoms Distress Module (HIV‐SDM).  

**Materials and methods: **Three hundred and forty‐eight virosuppressed patients, coming from a PI/r‐based ART and switching to DRV/c were enrolled in this single‐arm, prospective, non‐interventional study. Of them, 336 were evaluable. The questionnaires were administered to patients at fixed study timepoints: baseline (Visit [V]1); after 4 to 8 weeks from baseline (V2); and at last visit (V4) 48 ± 6 weeks from baseline. For HIV‐TSQ, an improvement is shown by an increased score, while for HIV‐SDM questionnaire, symptoms improvement is connected to a decreased score. Here we show the results obtained comparing the questionnaires scores registered at study baseline (V1) with the scores at last visit (48 weeks of treatment, V4).

**Results: **In total, 250 patients (174 males and 76 females) filled both the HIV‐TS and the HIV‐SDM questionnaires at baseline and at V4. Their demographic characteristics are shown in Table 1. Regarding the HIV‐TSQ, the patients’ satisfaction for their treatment was good at enrolment (67% of patients were very satisfied) but it further improved in 87.4% of patients at V4. Regarding the HIV‐SDM scores, the overall burden of symptoms decreased from V1 to V4: the mean value (SD) was 10.1 (9.9) at baseline, while it was 9.3 (10.4) at V4 (overall). Gender (*p* = 0.0055) and SDM baseline score (*p* < 0.0001) have statistically significant effect on SDM score at V4. Regarding specific symptoms, a reduction in percentages of patients experiencing fatigue (from 46.8 to 42.0, *p* < 0.0001) and gastrointestinal symptoms (diarrhoea and bloating) was observed at V4 (Figure 1).

**Table:** Abstract P108 – Table 1. Demographic characteristics

Baseline characteristics
Males, N (%)	174 (69.6)
Females, N (%)	76 (30.4)
Age, mean (SD)	48.6 (9.7)
White race, N (%)	239 (95.6)
HCV positive, N (%)	66 (26.4)
No. of years from the first HIV‐1 positive test, mean (SD)	14.0 (9.4)
No. of years from the first ARV treatment, mean (SD)	11.6 (7.7)
No. of years from the first ARV treatment PI/r based, mean (SD)	6.9 (5.2)
No. of years from virosuppression, mean (SD)	5.2 (4.2)
CDC category C, N (%)	70 (28.0)
CD4 nadir (cell/mm^3^), mean (SD)	213.2 (162.9)

Abstract P108 – Figure 1. Percentage of patients experiencing symptoms ‐ V4 versus V1.

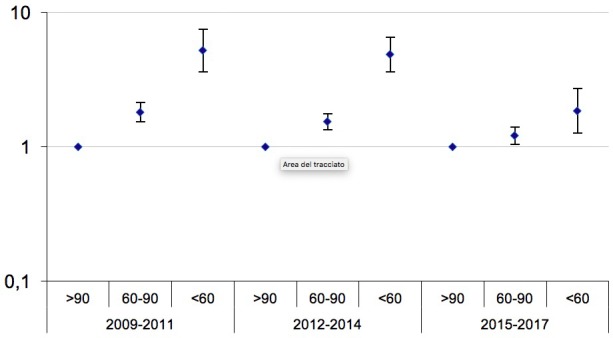

**Conclusions: **The results here reported show that the switch from a PI/r‐based therapy to a DRV/c‐based ART lead to an overall improvement of patients’ satisfaction and reduction of the burden of symptoms related to previous regimens, thus improving the patients’ quality of life.

## P109

### Effectiveness and safety of cobicistat‐boosted darunavir‐based antiretroviral treatment in an Italian observational cohort: the TMC114FD1HTX4003 (ST.O.RE.) study

**A Gori^1^, A Antinori^2^, D Ripamonti^3^, S Rusconi^4^, N Gianotti^5^, R Maserati^6^, A Muscatello^7^, V Di Cristo^8^, A Castagna^9^, G Rizzardini^10^, A Cattelan^11^, B Menzaghi^12^, G Sterrantino^13^, S Kiros^14^, F Castelli^15^, E Focà^15^, B Saccani^15^, G Orofino^16^, M Farenga^16^, R Cauda^17^, S La Monica^17^, V Vullo^18^, A De Luca^19^, B Rossetti^20^, E Manzillo^21^, C Gioè^22^, B Celesia^23^, M Locatelli^23^, G Madeddu^24^, P Bagella^24^, T Santantonio^25^, S Ferrara^25^, L Cosco^26^, E Pontali^27^, A d'Arminio Monforte^28^, R Curetti^29^, M Andreoni^30^, C Stingone^30^, A Uglietti^31^, R Termini^31^ and D Mancusi^31^**

^1^Infectious Diseases Unit, Fondazione IRCCS Ca’ Granda, Ospedale Maggiore Policlinico, University of Milan, Milano, Italy. ^2^HIV/AIDS Department, National Institute for Infectious Diseases “Lazzaro Spallanzani” IRCCS, Rome, Italy. ^3^Division of Infectious Diseases, ASST Papa Giovanni XXIII, Bergamo, Italy. ^4^Divisione Malattie Infettive, DIBIC Luigi Sacco, Università degli Studi di Milano, Milano, Italy. ^5^Dipartimento di Malattie Infettive, San Raffaele Scientific Institute, Milano, Italy. ^6^Clinica Malattie Infettive, Fondazione Policlinico San Matteo, Pavia, Italy. ^7^Infectious Diseases Unit, Fondazione IRCCS Ca’ Granda, Ospedale Maggiore Policlinico, Milano, Italy. ^8^Infectious Diseases, DIBIC Luigi Sacco and University of Milan, Milano, Italy. ^9^Unit of Management & ARV Treatment of HIV Infection, Faculty of Medicine and Surgery, Vita‐Salute San Raffaele University and IRCCS San Raffaele Hospital, Milano, Italy. ^10^1st Division of Infectious Diseases, ASST Fatebenefratelli‐Sacco, Milano, Italy. ^11^Division of Infectious and Tropical Diseases, Azienda Ospedaliero‐Universitaria di Padova, Padova, Italy. ^12^Infectious Diseases, Azienda Socio Sanitaria Territoriale della Valle Olona‐Busto Arsizio, Busto Arsizio, Italy. ^13^Division of Tropical and Infectious Diseases, Azienda Ospedaliero‐Universitaria Careggi, Firenze, Italy. ^14^Infectious Disease Unit, University of Firenze, Firenze, Italy. ^15^Department of Infectious and Tropical Diseases, University of Brescia and Spedali Civili General Hospital, Brescia, Italy. ^16^Unit of Infectious Diseases, Amedeo di Savoia Hospital, Torino, Italy. ^17^Institute of Clinical Infectious Diseases, Catholic University of the Sacred Heart of Roma, Roma, Italy. ^18^Department of Public Health and Infectious Disease, Sapienza University, Roma, Italy. ^19^Department of Medical Biotechnologies, University of Siena, Siena, Italy. ^20^University Division of Infectious Diseases, Siena University Hospital, Siena, Italy. ^21^VIII Divisione di Malattie Infettive, A.O.R.N. Cotugno, Napoli, Italy. ^22^II Infectious Diseases Division, Policlinico Universitario “P.Giaccone”, Palermo, Italy. ^23^UOC Malattie Infettive, ARNAS “Garibaldi”, Catania, Italy. ^24^Unit of Infectious Diseases, University of Sassari, Sassari, Italy. ^25^Department of Clinical and Experimental Medicine, University of Foggia, Foggia, Italy. ^26^Unit of Infectious Diseases, “Pugliese‐Ciaccio” Hospital, Catanzaro, Italy. ^27^Infectious Diseases, Ospedali Galliera, Genova, Italy. ^28^Dipartimento di Scienze della Salute, ASST Santi Paolo e Carlo, Milano, Italy. ^29^Clinica Malattie Infettive e Tropicali, ASST Santi Paolo e Carlo, Milano, Italy. ^30^Department of Medicine of Systems, University of Roma “Tor Vergata”, Roma, Italy. ^31^Medical Affairs, Janssen‐Cilag SpA, Cologno Monzese, Italy

**Background: **Darunavir/cobicistat (DRV/c) is a protease inhibitor coformulated with its booster cobicistat in fixed‐dose combination (FDC), approved for the treatment of HIV‐1 infection. This FDC allows reducing the pill burden of ART, thus allowing the reduction of mistakes in drug administration. ST.O.RE. study is an Italian prospective, single‐arm, multicentre, non‐interventional study aimed at collecting data from clinical practice regarding the effectiveness and safety of DRV/c‐based regimen in HIV‐1 infected, virologically suppressed outpatients, previously in treatment with an ART based on ritonavir‐boosted protease inhibitors (PI/r), switching to DRV/c 800/150 mg taken once daily.

**Materials and methods: **Twenty‐five Italian infectious diseases centres participated in this study. The patients were enrolled according to DRV/c Summary of Product Characteristics and they were prospectively observed, as per clinical practice, for 48 ± 6 weeks after starting DRV/c‐based regimen. The primary objective of this study was to describe the effectiveness of this regimen, defined as virological suppression at 48 weeks, measured as maintenance of HIV‐RNA <50 copies/mL as per FDA Snapshot algorithm; virological failure (VF) was defined as HIV‐RNA ≥50 copies/mL. In addition, the study protocol established to analyse virological suppression according to TLOVR algorithm. Reasons for discontinuation and adverse events occurred during the study were also reported.

**Results: **A total of 348 patients were enrolled; 336 of them were evaluable. Demographics are shown in Table 1. Patients’ disposition and results are shown in Figure 1. Two hundred and eighty‐two of 336 (84%) patients completed the study; of them, 235 had data in window (48 ± 6 weeks) and 231 had an HIV‐RNA <50 copies/mL. According to TLOVR algorithm, 278/336 patients (83%) were virosuppressed at Week 48. Only six virological failures occurred (1.8%), all of them due to lack of adherence (two before Week 48); one patient changed therapy due to VF after Week 48 (81 copies/mL). Regarding safety, 19 serious adverse events were reported during the study, none related to DRV/c. Fifteen patients (4.5%) discontinued the study due to adverse events (including one pregnancy); six patients (1.8%) discontinued the study due to adverse drug reaction to DRV/c.

**Table:** Abstract P109 – Table 1. Demographic characteristics

Main characteristics
Gender, N (%)	
Male	229 (68.2)
Female	107 (31.8)
Age, mean (SD)	49.2 (9.6)
White race, N (%)	318 (94.6)
No. of years from the first HIV‐1 positive test, mean (SD)	14.4 (9.5)
No. of years from the first ARV treatment, mean (SD)	11.7 (7.6)
No. of years from the first ARV treatment PI/r based, mean (SD)	7.4 (5.7)
No. of years from virosuppression, mean (SD)	5.1 (4.2)
CDC category C, N (%)	97 (28.9)
CD4 nadir (cells/mm^3^), mean (SD)	213.8 (165.3)
Backbone at enrolment (%)	
TDF/FTC	42.4
ABC/3TC	16.9
Dual therapies	34.5
Other	6.2

Abstract P109 – Figure 1. Patients’ disposition and results.

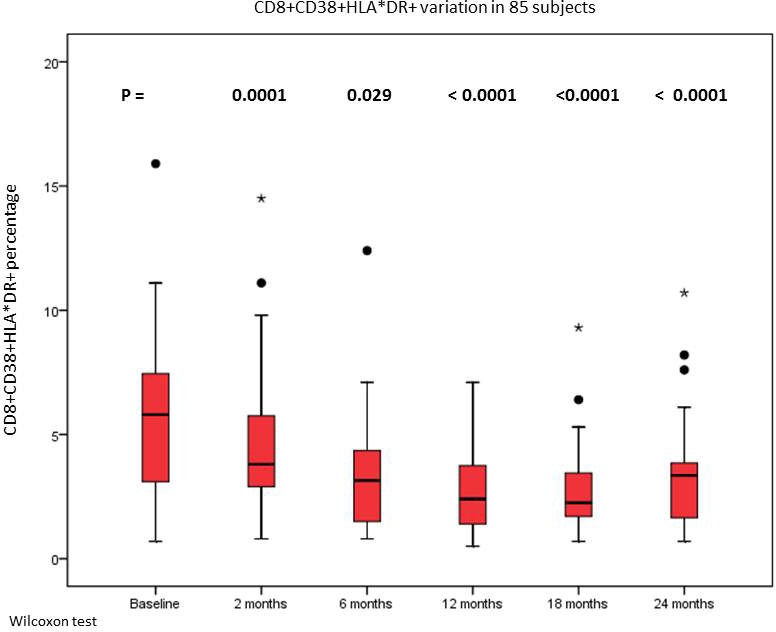

**Conclusions: **This study has confirmed, in clinical practice, the high virological suppression and good safety profile according to DRV/c pivotal trials, thus showing how an ARV regimen based on DRV/c is an effective treatment option for patients, ensuring maintenance of virosuppression together with good tolerability outcomes.

## P110

### Switching to a dual regimen with the combination of boosted darunavir plus raltegravir in severely experienced patients: a multicentre, retrospective analysis

**J Casado^1^, R Montejano^2^, E Negredo^3^, J Blanco^4^, N Espinosa^5^, A Mena^6^, M Montero^7^, R Palacios^8^, J Lopez^9^, J Vergas^10^, M Galindo^11^, M García del Toro^12^, A Cabello^13^ and A Diaz de Santiago^14^**

^1^Infectious Diseases, Ramon y Cajal Hospital, Madrid, Spain. ^2^Infectious Diseases, La Paz Hospital, Madrid, Spain. ^3^Infectious Diseases, Germans Trias i Pujol, Badalona, Spain. ^4^Infectious Diseases Clinic, Barcelona, Spain. ^5^Infectious Diseases, Virgen del Rocio, Sevilla, Spain. ^6^Infectious Diseases, CHU A Coruña, A Coruña, Spain. ^7^Infectious Diseases, La Fe, Valencia, Spain. ^8^Infectious Diseases, Virgen de la Victoria, Málaga, Spain. ^9^Infectious Diseases, Gregorio Marañon, Madrid, Spain. ^10^Infectious Diseases, Clinico San Carlos, Madrid, Spain. ^11^Infectious Diseases, Clínico Universitario, Valencia, Spain. ^12^Infectious Diseases, General Universitario, Valencia, Spain. ^13^Infectious Diseases, Jimenez Diaz, Madrid, Spain. ^14^Infectious Diseases, Puerta de Hierro, Madrid, Spain

**Background: **There are few data about the safety and efficacy of the combination of a dual regimen with raltegravir (RAL) plus boosted darunavir (b/DRV), as simplification or switching strategy in case of toxicity, especially in patients who had failed successive lines of therapy.

**Methods: **A Phase IV, multicentre, retrospective analysis (NCT03348449), including consecutive patients without previous failure to integrase inhibitors suppressed for more than 48 weeks, was performed in 14 HIV units. The primary endpoint was the proportion of patients maintaining virological suppression at 48 and 96 weeks.

**Results: **A total of 340 patients (mean age 51 years, 25% females) were followed for a median of 28 months (14.4 to 48.7; 185.6 patient‐years of follow‐up). Patients were highly pretreated (median time of HIV infection 18.2 years, nadir CD4 +  count 138 cells/mL, mean of 9.9 antiretroviral regimens), and were receiving a suppressive regimen for a median of 20.6 months (TDF in 48% of cases, DRV or RAL in 49% each). Previous genotypic profiles in 54% of cases showed a median of 5 NRTI and 3 NNRTI mutations. Of note, GSS showed reduced susceptibility to DRV in 18% of cases. Overall, discontinuation because of virological failure (VF) throughout the study was rare (0.8%; 95% CI −0.1% to 1.8%). At 48 weeks, nine patients had detectable HIV RNA level (2.6%), and efficacy (Snapshot analysis) was 91% (95% CI 88 to 94%): 20 patients changed therapy because of toxicity (six patients, one death due to sepsis, one increased CK, one anxiety, three diarrhoea), pregnancy (one), drug‐drug interactions (eight) or were lost to follow‐up (five). At 96 weeks, efficacy was 86% (95% CI 83 to 90%; no additional cases of VF were observed). Median CD4/CD8 ratio increased from 0.59 to 0.66 (*p* = 0.02) and to 0.69, CD4+ count from 560 to 596 cells/mL (*p* < 0.01), and mean estimated glomerular filtration rate (eGFR) increased from 85.6 to 87.4 and to 88.5 mL/min at 48 and 96 weeks. This improvement was greater for patients previously receiving TDF (eGFR +3.6 mL/min, *p* = 0.04; serum phosphate +0.27 mg/dL, *p* = 0.05; DXA in 33 patients with spine T score from −1.43 to −1.29, *p* = 0.13). Total cholesterol and LDL‐c increased for +5% (*p* = 0.02) and +9.1% at 48 weeks, partly corrected at 96 weeks.

**Conclusions: **A dual treatment with the combination of raltegravir and boosted darunavir is associated with maintenance of virological suppression, even in severely experienced patients, and with improvements in CD4+ count, CD4/CD8 ratio and in renal and bone toxicity.

## P111

### Switching from atazanavir/ritonavir (ATV/RTV) to ATV/cobicistat (ATV/COBI) is associated with decrease of plasma lipids and liver fibrosis

**A Trentalange, C Polifroni, C Carcieri, C Alcantarini, C Montrucchio, L Marinaro, N Forni, A Palazzo, A Barco, C Costa, A Lazzaro, M Tettoni, A De Nicolò, A D'Avolio, G Di Perri, A Calcagno, S Bonora**

Infectious Diseases, University of Turin, Amedeo di Savoia Hospital, Torino, Italy

**Background: **Previous data suggested a lower dyslipidaemic potential for COBI compared to RTV. While switch from darunavir (DRV)/RTV to DRV/COBI has been associated with total cholesterol (TC) and triglycerides (TG) decrease, no virological, metabolic and PK data of switching from RTV to COBI as booster of ATV have been yet obtained in the clinical setting. Therefore, our aim was to study efficacy, safety and PK of switching from ATV/RTV to ATV/COBI. 

**Materials and methods: **Adult HIV‐positive patients who switched from ATV 300 mg/RTV 100 mg to ATV 300 mg/COBI 150 mg without any other therapeutic change were enrolled; demographics, virological, metabolic and PK data were recorded at baseline (BL) and 24 weeks after switching (W24). Hypercholesterolaemia was considered as TC >190 mg/dL and/or LDL cholesterol >115 mg/dL, hypertriglyceridaemia as TG >180 mg/dL. Plasma concentrations were measured by a validated HPLC methods. FIB‐4 score was calculated as (age*AST)/(platelets*SQR(ALT)). Variables were described as medians (interquartile range [IQR]) or number (percentage) and were compared using paired non‐parametric tests (Wilcoxon and McNemar's).

**Results: **We included 121 patients: baseline characteristics are shown in Table 1. Fifteen (12.3%) patients discontinued. BL and W24 HIV RNA was <20 copies/mL in 88.2% and 82.1% of participants (*p* = 0.38). A small although significant increase of serum creatinine at W24 (0.1 mg/dL [*p *≤ 0.001]) was found, while total or indirect bilirubin did not change. In whole population variations of TC [from 183 mg/dL (151 to 210) to 173 mg/dL (147 to 204)], LDL‐C [117 mg/dL (87 to 140) to 118 mg/dL (94 to 146)] and TG [125 mg/dL (92 to 192) to 121 mg/dL (81 to 175)] were not significant (*p* = 0.08, *p* = 0.1 and *p* = 0.1, respectively). As opposite, in patients with BL hypercholesterolaemia (44.6%) TC decreased from 213 mg/dL (196 to 228) to 194 mg/dL (175 to 213) (*p* < 0.001) and LDL‐C from 140 mg/dL (130 to 155) to 137 mg/dL (120 to 155) (*p* = 0.05). In patients with BL hypertriglyceridaemia (27.5%) TGL decreased from 240 mg/dL (207 to 276) to 204 mg/dL (159 to 252) (*p* = 0.01). No significant differences were found in ATV Ctrough, pre‐ and post‐switch (*p* = 0.56). FIB‐4 value decreased from 1.04 (0.73 to 1.41) to 0.93 (0.67 to 1.21) (*p* = 0.001), and this finding was confirmed even in HCV/HBV‐negative patients [0.96 (0.68 to 1.39) to 0.90 (0.63 to 1.20), *p* = 0.01].

**Table:** Abstract P111 – Table 1. Demographic, immunovirological and clinical variables of study participants

N	121
Age, years	48 (41 to 55)
Male gender, n (%)	84 (68.9)
European ancestry, n (%)	100 (81.9)
BMI, kg/m^2^	24.2 (21.1 to 26.4)
CD4 T lymphocytes, cells/μL	601 (437 to 820)
CD4 T lymphocytes, %	31.4 (24.7 to 38)
CD4 T lymphocytes, ratio	0.8 (0.5 to 1.1)
CD4 T lymphocytes nadir, cells/μL	224 (129 to 290)
HCV+, n (%)	25 (21.9)
HBV+, n (%)	3 (2.5)
Plasma HIV RNA <20 copies/mL, n (%)	105 (88.2)
Duration of HIV infection, years	14 (8 to 20.5)
Associated antiretrovirals, NNRTIs n (%)	1 (0.8)
INSTI n (%)	26 (21.5)
NRTI n (%)	96 (79.3)
CCR5 n (%)	5 (4.1)
Comorbidities Hypertension, n (%)	21 (21)
Diabetes, n (%)	8 (8.1)

Data are described with number (percentage) or median (IQR). BMI = body mass index; INSTI = integrase strand‐transfer inhibitor; CCR5 = C‐C chemokine receptor type 5.

**Conclusions: **This is the first report on switching from ATV/RTV to ATV/COBI in the clinical setting. Virological efficacy was maintained and substantial PK equivalence was confirmed. As compared to RTV, COBI associated with ATV showed a decrease of lipids in dyslipidaemic patients and a general decrease of liver fibrosis.

## P112

### Switch from tenofovir disoproxil fumarate (TDF)‐ to tenofovir alafenamide (TAF)‐based regimens in clinical practice: real‐world data of the German PROPHET cohort study

**M Bickel^1^, C Wyen^2^, C Spinner^3^, A Baumgarten^4^, H Jaeger^5^, N Postel^6^, E Wolf^7^, C Hoffmann^8^, S Esser^9^, S Klauke^1^ and K Schewe^10^**

^1^Clinical Care, Infektiologikum Frankfurt, Frankfurt, Germany. ^2^Private Practice Ebertplatz, Drs Kuemmerle/Voigt/Wyen, Cologne, Germany. ^3^Department of Medicine II, University Hospital Klinikum rechts der Isar, Munich, Germany. ^4^Clinical Care, Center for Infectiology Berlin Prenzlauer Berg GmbH (Zibp), Berlin, Germany. ^5^HIV Research and Clinical Care Centre, MVZ Karlsplatz, Munich, Germany. ^6^Clinical Care, Prinzmed, Munich, Germany. ^7^Clinical Research, MUC Research, Munich, Germany. ^8^Clinical Research, ICH Studycenter, Hamburg, Germany. ^9^University HIV/STD Center Essen, Essen, Germany. ^10^Association of Physicians specialized in HIV Care, Dagnae e.V., Berlin, Germany

**Background: **PROPHET is a prospective, nationwide, 2‐year, multicentre cohort study in chronically HIV‐infected adults initiated on ART between August 2014 and September 2015. Inclusion criterion was the use of a regimen recommended by treatment guidelines in Germany (TDF/FTC or ABC/3TC plus either INI, NNRTI or PI). Primary objectives included pharmacoeconomic and clinical outcomes of different ART strategies. During the study new antiretroviral options such as TAF‐based fixed‐dose combinations became available (EVG/COBI/FTC/TAF (November 2015), FTC/TAF (April 2016), RPV/FTC/TAF (June 2016)). Here we focus on characteristics and outcomes in PROPHET participants switched from TDF‐ to TAF‐based ART.

**Methods: **Variables of interest included prior regimens, reasons for switch, persistence of TAF, maintenance of viral suppression and health‐related quality of life (HRQoL) measured by ASDM (ACTG symptom distress module) and SF‐12 physical and mental component summary scores.

**Results: **PROPHET included 444 patients (91% males) initiated on INI‐ (n = 170, 84% DTG), NNRTI‐ (n = 133, 95% RPV) or PI‐based ART (n = 141, 93% DRV). FTC/TDF was used in 346 patients (78%). During the study, 150 patients (34% of the cohort; 91% males, 95% with HIV‐RNA <50 copies/mL) were switched from TDF‐based to TAF‐based ART, i.e. FTC/TAF + 3rd agent (n = 58), RPV/FTC/TAF (n = 51) and EVG/COBI/FTC/TAF (n = 41). Prior regimens are shown in Table 1. Primary reasons for switch (>5%) were prevention of renal/bone toxicity (51%), use of TAF as TDF successor drug (12%), adverse drug reactions (ADRs) on prior ART (10%), ART simplification (9%) and patient request (7%). Until study end, seven patients (5%) discontinued TAF‐based regimens (four patients due to ADRs (3%)); in addition, four study discontinuations (unrelated to the use of TAF‐based ART) were reported. Kaplan‐Meier estimates of persistence on TAF at months 6, 12 and 18 were 96%, 92% and 92%, respectively. At last follow‐up, after a median of 7.6 months on TAF‐based ART (IQR 4.9 to 11.9, max. 20.3), HIV‐RNA levels were <50 copies/mL in 91% of patients (115/126; on‐treatment). Ninety‐five percent of patients with <50 copies/mL prior to switch (114/120) maintained viral suppression after switch. Overall, HRQoL improved after ART initiation and remained relatively stable in patients switched to TAF‐based ART after Month 12.

**Table:** Abstract P112 – Table 1. Prior ART, background and characteristics of patients switched from TDF‐ to TAF‐based ART

TAF‐based ART after switch		
	Total	FTC/TAF + 3rd agent^a^	RPV/FTC/TAF	EVG/COBI/FTC/TAF
	(n = 150)	(n = 58, 39%)	(n = 51, 34%)	(n = 41, 27%)
TDF‐based ART prior to switch
FTC/TDF/RPV	(n = 55)	‐‐	50 (91%)	5 (9%)
TDF/FTC + DRV/RTV (or COBI)	(n = 40)	25 (63%)	‐‐	15 (38%)
FTC/TDF + DTG	(n = 32)	28 (88%)	1 (3%)	3 (9%)
FTC/TDF/EVG/COBI	(n = 17)	‐‐	‐‐	17 (100%)
Other	(n = 6)	5 (83%)	‐‐	1 (17%)
Male gender, N (%)	137 (91)	55 (95)	47 (92)	35 (85)
Age at switch, years, median (IQR)	43 (34 to 51)	45 (34 to 53)	42 (35 to 50)	40 (33 to 50)
CDC stage C prior to switch, N (%)	20 (13)	14 (24)	3 (6)	3 (7)
CD4 cell count prior to switch, cells/µL, median (IQR)	586 (416 to 780)	491 (360 to 757)	674 (530 to 880)	586 (392 to 756)
CD4 cell count prior to ART initiation, cells/µL, median (IQR)	386 (230 to 566)	306 (161 to 468)	479 (335 to 638)	367 (154 to 529)
HIV‐1 RNA <50 copies/mL prior to switch, N (%)	142 (95)	54 (93)	51 (100)	37 (90)
HIV‐1 RNA >100,000 prior to ART initiation, N (%)	54 (36)	32 (55)	9 (18)	13 (32)
Late presentation at ART initiation^b^, N (%)	70 (47)	36 (62)	14 (27)	20 (49)

^a^other than RPV/FTC/TAF or EVG/COBI/FTC/TAF. ^b^CD4 < 350 cells/µL and/or CDC stage C.

**Conclusions: **After availability of TAF‐based regimens, one‐third of patients included in PROPHET were switched from TDF‐ to TAF‐based ART. The main reason for switch was prevention of renal and bone toxicity. Experience from clinical trials concerning treatment retention, safety and efficacy was confirmed by a low rate of TAF discontinuations due to ADRs and maintenance of viral suppression in 95% of patients.

## P113

### Soluble activation and inflammation markers in HIV dual therapy in the setting of virological suppression: Trilobithe study

**M Molano, M Monsalvo, F Hernandez, M Fontecha, A Vallejo, J Casado**

Infectious Diseases, Hospital Ramon y Cajal, Madrid, Spain

**Background: **Dual therapies could reduce the toxicity of antiretroviral treatment without reducing its effectiveness. Nevertheless, it is important to know if reducing the number of drugs does not facilitate an increase in inflammation and activation markers. We evaluated the plasma levels of activation, inflammation and blood coagulation in HIV‐1 patients receiving dual therapy.

**Methods: **Cross‐sectional study of three groups of patients with HIV infection: patients in triple therapy (N = 24) and patients after 24 weeks (N = 12) or after 48 weeks (N = 32) from the switching from triple therapy to dual therapy.

**Results: **The main dual therapies were dolutegravir (DTG) + rilpivirine (RPV, 77%), lamivudine + ritonavir/darunavir (r/DRV, 14%), r/DRV + RPV (7%) and r/DRV + raltegravir (2%). Age, sex, risk factors, HIV diagnosis time, time on ART, time on previous triple therapy, nadir CD4 count and infection by HCV were similar in the three groups of patients. The adherence during dual therapy was 100% and the HIV viral load remained undetectable during dual therapy. The levels of CD4 count and CD4:CD8 ratio were higher after 48 weeks with dual therapy compared with patients under triple therapy (690 [481 to 874] vs. 577 [441 to 909] and 0.85 [0.61 to 1.08] vs. 0.69 [0.45 to 0.88], respectively), although without statistical significance. The levels of IL6 and sCD14 were significantly lower in patients after 48 weeks under dual therapy (Table 1), while the levels of hsCRP, IP‐10, sCD163 and D‐dimer were similar. Six patients analysed sequentially from triple therapy to 48 weeks under dual therapy showed significant decrease in the levels of sCD14 (Wilcoxon‐rank test, *p* = 0.028), without changes in the rest of the parameters.

**Table:** Abstract P113 – Table 1. Soluble markers

	Patients under triple therapy, N = 24 (1)	Patients after 24 weeks dual therapy, N = 12 (2)	Patients after 48 weeks dual therapy, N = 32 (3)	1 vs. 2 *p* value	2 vs. 3 *p* value	1 vs. 3 *p* value
IL6 (pg/mL)	3.23 (1.71 to 4.85)	3.24 (1.90 to 12,0)	1.45 (0.96 to 4.17)	0.585	0.010	0.009
IP10 (pg/mL)	229 (135 to 298)	342 (131 to 432)	139 (46 to 291)	0.280	0.021	0.060
hs‐CRP (mg/L)	1.37 (0.58 to 1.90)	1.17 (0.66 to 2.01)	1.31 (0.63 to 2.25)	0.804	0.969	0.817
sCD14 (pg/mL)	4.40 (4.17 to 4.98)	4.43 (4.20 to 4.64)	4.05 (3.39 to 4.34)	0.518	0.008	<0.001
sCD163 (pg/mL)	626 (490 to 802)	506 (415 to 937)	511 (238 to 726)	0.631	0.442	0.141
D‐dimer (µg/mL)	1.72 (1.53 to 1.91)	1.80 (1.59 to 2.01)	1.83 (1.53 to 2.08)	0.497	0.948	0.380

**Conclusions: **HIV patients in dual therapy show improvement or stability of different markers of inflammation and activation compared with patients under triple therapy.

## P114

### Clinical observations of ART switching in HIV‐suppressed patients after availability of TAF

**R Elion, J Eron^2^, S Santiago^3^, P Sax^4^, M Rampogal^5^, G Huhn^6^, A Musallam^7^, K Althoff^8^ and J Winston^9^**

^1^Medicine, George Washington University School of Medicine, Bethesda, MD, USA. ^2^Division of Infectious Diseases, UNC Center for AIDS Research, Chapel Hill, NC, USA. ^3^Medicine, Care Resource, Miami, FL, USA. ^4^Division of Infectious Diseases, Brigham and Women's Hospital, Boston, MA, USA. ^5^Medicine, Midway Immunology and Research, Fort Pierce, FL, USA. ^6^Infectious Diseases, The Ruth M. Rothstein Core Center, Rush University Medical Center, Chicago, IL, USA. ^7^Analytics, Trio Health, La Jolla, CA, USA. ^8^Epidemiology, Johns Hopkins Bloomberg School of Public Health, Baltimore, MD, USA. ^9^Department of Medicine, The Mount Sinai Hospital, New York, NY, USA

**Background: **In HIV‐suppressed patients, DHHS guidelines support switching ART for tolerability and adherence. Tenofovir alafenamide (TAF) was approved in November 2015 as a component of E/C/F/TAF, and in 2016 as F/TAF and R/F/TAF. This study evaluated ART switching in HIV‐suppressed patients in the first year of TAF availability.

**Methods: **EMR, prescription and dispensing data were collected from four HIV treatment centers as of November 2015 through October 2016 for HIV patients with virologic suppression (HIV <200 copies/mL) and followed for at least 365 days (Table 1).

**Results: **In the first year of TAF availability, 50% (470/931) of patients switched regimens with 86% (404/470) containing TAF. Fifty‐five percent (348/628) of white patients switched compared to 40% (80/202) black patients (*p* < 0.001). Patients who switched were more likely to have lower eGFR (<90 mL/min) than those who did not switch (81% [369/455] vs. 66% [255/386] [*p* < 0.001]). Those who switched to TAF‐based regimens were numerically but not significantly more likely to maintain virologic suppression than those who switched to non‐TAF regimens (91% [312/344] vs. 81% [30/37] [*p* = 0.057]). Of the 669 patients on TDF‐containing regimens, 59% (397/669) switched therapy with 93% (371/397) switching to a TAF‐based regimen. MTR use in the switch group declined from 45% (211/470) pre‐switch to 35% (166/470) post‐switch. MTR use was 44% (201/461) in the non‐switch group.

**Conclusion: **This study utilized EMR, prescription and dispensing data to assess ART switching in HIV‐suppressed patients at four HIV centers in the US. In the first year of TAF availability, 50% patients switched therapies with 85% switching to TAF. Switching to TAF was associated with a pre‐switch lower eGFR and a post‐switch trend to higher virologic suppression. Black patients were less likely to switch ART compared to whites. Further assessments of virologic suppression between TAF and non‐TAF switching should be explored in future observational studies.

**Table:** Abstract P114 – Table 1. Demographic and baseline measures

No. (%) unless indicated	Switch patients n = 470			Non‐switch patients n = 641	Total n = 931
	TAF switch n = 404	Non‐TAF switch n = 66	Total n = 470		
Age ‐ mean (SD)^a^	50 (12)	53 (11)	50 (12)	51 (12)	50 (12)
Male^a^	349 (86%)	49 (74%)	398 (85%)	375 (81%)	773 (83%)
Race					
Asian	‐	‐	‐	3 (1%)	3 (0%)
Black ^a^^,^^b^	62 (15%)	18 (27%)	80 (17%)	122 (26%)	202 (22%)
Hispanic^b^	17 (4%)	3 (5%)	20 (4%)	33 (7%)	53 (6%)
White^b^	303 (75%)	45 (68%)	348 (74%)	280 (61%)	628 (67%)
Other	22 (5%)	‐	22 (5%)	23 (5%)	45 (5%)
Baseline measures					
CD4 count 200 (cells/mcL)^b^	14/364 (4%)	2/59 (3%)	16/423 (4%)	40/389 (10%)	56/812 (7%)
eGFR 90 (mL/min)^b^	316/391 (81%)	53/64 (83%)	369/455 (81%)	255/386 (66%)	624/841 (74%)

^a^ comparison between TAF switch and non‐TAF switch is statistically significant based on chi‐square or t‐test where appropriate. ^b^comparison between switch and non‐switch is statistically significant based on chi‐square or t‐test where appropriate.

## P115

### Antiretroviral therapy without nucleoside reverse transcriptase inhibitors: dual therapy with darunavir/p and rilpivirine. Safety and efficacy in clinical practice. RIDAR 2

**P Arazo^1^, M Galindo^2^, M Montero^3^, C Tornero^4^ and J Pasquau^5^**

^1^Infectious Diseases, Hospital Miguel Servet, Zaragoza, Spain. ^2^Infectious Diseases, Hospital Clínico, Valencia, Spain. ^3^Infectious Diseases, Hospital Universitari i Politecnic la Fe, Valencia, Spain. ^4^Infectious Diseases, Hospital Francesc de Borja, Gandía, Spain. ^5^Infectious Diseases, Hospital Virgen de las Nieves, Granada, Spain

**Introduction: **Long‐term toxicity of antiretrovirals, ageing of people with HIV and multiple comorbidities made necessary the development of new strategies without nucleoside inhibitors. One option is double therapy with darunavir/p (DRV/p) and rilpivirine (RIL). We do not have data from clinical trials, but we do have real‐life data. In this context of clinical practice, we will retrospectively review the histories of patients who started this combination and were followed for 48 weeks.

**Patients and methods**: This is a multicentre observational retrospective study (19 hospitals). We reviewed the clinical history of all patients with HIV who began treatment with DRV/p and RIL because of adverse events, avoid toxicity, intolerance or simplification; and were followed for 48 weeks. The previous therapeutic history or the mutations associated with resistance to antiretroviral drugs did not allow a change to simpler antiretroviral treatment. We collected sociodemographic data, information related to HIV infection and comorbidities. The statistical analysis is done with SPSS Statistics.

**Results: **We included 281 patients with an average age of 52 years (SD 45 to 57), 76% men, with a median follow‐up since the diagnosis of 17 years (IQR 9 to 24); stage C3 118 (43%); median of previous treatments 3.5 (IQR 2 to 8). When they start this regimen 190 (68%) were undetectable and 65 (23%) had a viral load between 50 and 1000 copies/mL; the median basal CD4 was 610 cells/mm^3^ (410 to 839). Regarding the reasons for starting this regimen were: 81 (28.82%) adherence problems, 82 (29.1%) toxicity, 97 (34.5%) simplification and 79 (28.1%) prevention of complications. In Week 24, we have data from 251 patients: 221 remain undetectable (88.04%) and seven (2.78%) changed the treatment. In Week 48, we have data from 208 patients, 186 remain undetectable (89.42%). Sixteen patients (7.69%) had changed the treatment at the end of the study. The reasons for change were basically: toxicity (n = 4), lack of adherence (n = 4), interactions (n = 2).

**Conclusions: **The combination with DRV/p and RIL is a necessity in daily clinical practice. It is an effective and safe option and may be another treatment strategy in patients who cannot tolerate traditional transcriptase inhibitors and who have resistance mutations that do not allow other, simpler therapeutic options.

## P116

### Changes in liver steatosis after switching from efavirenz to rilpivirine among HIV‐infected patients: the RIFLE study

**C Sayago, J Macías, M Conde, N Merchante, J Gómez‐Mateos, J Pineda**

Infecciosos, Hospital de Valme, Sevilla, Spain

**Background: **Non‐alcoholic fatty liver disease (NAFLD) could be an emerging problem in HIV infection and ART may play a role in its appearance.

**Objective: **To evaluate changes in hepatic steatosis (HS), measured by controlled attenuation parameter (CAP), in HIV‐infected patients who switched from efavirenz (EFV)‐containing regimens to rilpivirine (RPV)‐based combinations.

**Method: **This was a retrospective observational study, including HIV‐infected patients followed at one hospital from Spain. All of them met these criteria: 1) prior treatment with a regimen including EFV for ≥4 weeks; 2) switching from EFV to RPV in routine clinical practice; 3) plasma HIV‐RNA <50 copies/mL, at least during the 24 weeks before switching; 4) available CAP measurements within 6 months before and 12 ± 3 months after switching to RPV. HS was assessed using transient elastography (TE) examination with CAP measurement. A 238 dB/m cutoff was selected to define the presence of significant HS and 290 dB/m for severe HS. The primary clinical endpoint was the change in CAP value from baseline to the 12‐month evaluation.

**Results: **Sixty individuals met the inclusion criteria. Of them, 48 (80%) were male. The median (Q1 to Q3) age was 48 (40 to 50) years. The median baseline CD4 cell counts was 576 cells/µL and 55 (92%) individuals showed plasma HIV‐RNA below the detection level at the switch. Twenty‐five (42%) were actively co‐infected with HCV. Thirty‐four (57%) patients had a BMI from 25 to 30 and six (10%) from 30 to 35. Fifty‐four (90%) were on tenofovir (TDF)/FTC/EFV and four (6.7%) on abacavir (ABC)/3TC + EFV before switching. Fifty‐one (85%) and five (8.3%) received TDF/FTC/RPV and ABC/3TC/RPV, respectively, during the study period. Changes in CAP, as well as in metabolic and liver function parameters, are displayed in Table 1.

**Table:** Abstract P116 – Table 1. Changes in CAP, metabolic and liver function parameters between baseline and month 12 on RPV

Value	Baseline	After 12 months of RPV	*p* value
CAP	257 (201 to 305)	237 (203 to 274)	0.042
Liver stiffness	5.7 (4.4 to 7.6)	5.9 (4.7 to 8.2)	0.838
GGT	63 (42 to 180)	32 (21 to 53)	<0.001
GPT	32 (21 to 57)	25 (17 to 39)	0.013
Plasma triglycerides	102 (73 to 148)	89 (70 to 119)	0.003
Fasting plasma glucose	91 (82 to 100)	88 (82 to 96)	0.026

Thirty‐six (60%) and 30 (50%) (*p* = 0.210) patients showed significant HS at baseline and at the end of follow‐up, respectively. The corresponding figures for severe HS were 30% and 18% (*p* = 0.022).

**Conclusions: **Switching from EFV‐ to RPV‐containing combinations leads to an improvement in HS in HIV‐infected patients. The greater the degree of HS, the more marked the effect of switching.

## P117

### Triumeq® versus Genvoya®, real‐life experience in pretreated patients

**N Roda Puchalt^1^, L Ventayol Aguiló^1^, M Arrizabalaga Asenjo^1^, J Asensio Rodriguez^2^, A Ferre Beltran^2^, M Riera Jaume^2^ and A Payeras Cifre^1^**

^1^Internal Medicine – Infectious Diseases, Hospital Universitari Son Llàtzer, Palma de Mallorca, Spain. ^2^Internal Medicine – Infectious Diseases, Hospital Universitari Son Espases, Palma de Mallorca, Spain

**Background: **Switching strategies are justified for several reasons, including toxicity or simplification. STR scheme used in naïve patients like DTG/ABC/3TC (Triumeq^®^) and EVG/c + FTC/TAF (Genvoya^®^) may be an option. We aimed to compare our real‐life use experience of both combinations Triumeq^®^ and Genvoya^®^ and assess safety and security in pretreated patients.

**Materials and methods: **This retrospective, descriptive study from our cohort (eVIHA), which includes 3500 patients from two centres in Palma (Illes Balears, Spain), Hospital Universitari Son Espases and Hospital Universitari Son Llàtzer, analyse all switches from any previous treatment to DTG/ABC/3TC or EVG/c + FTC/TAF carried out from June 2016 to June 2017. We selected only those patients who had registered at least three visits: basal (previously to the switch), 24th and 48th week and we gather clinical and epidemiological data.

**Results: **We selected 199 patients who met these criteria. There were 61 patients who switched to Triumeq^®^. Their baseline characteristics were: mean age: 52 (SD12), 82% male, methods of infection were: 44.3% heterosexual, 26.2% MSM, 24.5% IDU. CD4 mean cell count were 662 cells/µL (SD293) and 91.8% had undetectable viral load (<50 copies/mL).  Estimated glomerular filtrate (CKD‐EPI) was 90.3 (SD19).  Total cholesterol mean was 189.4 mg/dL (SD42.5), HDL: 47.7 mg/dL (SD23) and triglycerides: 145.5 mg/dL (SD72.8).  There were 138 patients who switched to Genvoya^®^. Their baseline characteristics were: mean age 48 (SD 10), 78% male, methods of infection: 41.3% heterosexual, 34.8% MSM, 14.5% IDU. CD4 mean cell count was 681 cells/µL (SD299) and 94.2% had undetectable viral load.  Estimated glomerular filtrate was 93.7 (SD17).  Total cholesterol was 186.8 mg/dL (SD39.3), HDL: 45.7 mg/dL (SD19.2) and triglycerides: 132.7 mg/dL (SD75). *p* value was statistically nonsignificant in all the parameters compared. Previous more frequent treatment in those who switched to Triumeq^®^ was: TDF/FTC/EFV (18.1%), TDF/FTC/RPV (11.5%) and TDF/FTC + DRV/c (6.5%).  Previous more frequent treatment in those who switched to Genvoya^®^ was: TDF/FTC/EVG/c (45.6%), TDF/FTC/EFV (23.9%) and TDF/FTC/RPV (7.2%).  Causes of switch (times) to Triumeq^®^ were: simplification: 46; adverse event: 20; self discontinuation: 9 and failure: 2. Number of CD4 (cells/µL) before treatment, in 24th and 48th week for both groups was: Triumeq^®^: basal, 662; Week 24, 686; Week 48, 717; Genvoya^®^: basal, 681; Week 24, 686; Week 48, 682. Viral load (% patients with viral load <50 copies/mL) variation in both groups was: Triumeq^®^: basal, 91.8%; Week 24, 95.10%; Week 48, 98.3%; Genvoya^®^: basal, 94.2%; Week 24, 98.5%; Week 48, 96.9%. Total cholesterol, HDL cholesterol and triglycerides level evolution, glomerular filtration rate evolution and Framingham score changes are described in Table 1.

**Table:** Abstract P117 – Table 1. Evolution of total cholesterol, HDL cholesterol and triglycerides levels in 24th and 48th week and evolution of glomerular filtration rate and Framingham score in 24th and 48th week

		Basal	Week 24	Week 48
Total cholesterol (mg/dL)	Triumeq	189.4 (SD 42.5)	190.2 (SD 48)	190.5 (SD 41.3)
	Genvoya	86.8 (SD 39.3)	200.5 (SD 46.5)	200.9 (SD 42.9)
HDL cholesterol (mg/dL)	Triumeq	47.7 (SD 23)	43.7 (SD 11)	43.8 (SD 10)
	Genvoya	45.7 (SD 19.2)	47.6 (SD 13)	47.5 (SD 16)
Triglycerides (mg/dL)	Triumeq	45.5 (SD 72.8)	144.6 (SD 86)	141.1 (SD 79)
	Genvoya	132.7 (SD 75)	144.4 (SD 101)	144.3 (SD 96)
Estimated glomerular filtration (CKD‐EPI) (mL/min/1.73 m^2^)	Triumeq	90.3 (SD 19)	85.4 (SD 18)	85.8 (SD 19)
	Genvoya	93.7 (SD 17)	92.9 (SD 21)	92.7 (SD 19)
Framingham (%)	Triumeq	11.8 (SD 11.2)	12.7 (SD 12)	12.9 (SD 11.4)
	Genvoya	9.7 (SD 8.1)	9.9 (SD 7.3)	10.7 (SD 8.2)

**Conclusions: **Baseline clinical and epidemiological characteristics in patients who switched to Triumeq^®^ or Genvoya^®^ were similar in both groups of treatment. There were more patients who switched to Genvoya^®^ who had received only one treatment previously; however, the differences were statistically nonsignificant. Most of patients who switched to Genvoya^®^ had TDF/FTC/EVG/c as previous treatment, that means an expected change; conversely we didn't see any switch from ABC/3TC + DTG to Triumeq^®^. After switching there's an increase in suppression rate and better CD4 levels in both groups. The lipid profile tendency to get worse in Genvoya^®^ group may be due to TAF; however, filtrate decreases more in Triumeq^®^ group, perhaps due to the use of a formula (CKD‐EPI) that includes creatinine levels, which may be increased in this group because of dolutegravir.

## TREATMENT STRATEGIES: OTHER

## P118

### Safety, efficacy and durability of long‐acting CAB and RPV as two‐drug IM maintenance therapy for HIV‐1 infection: LATTE‐2 Week 160 results

**D Margolis^1^, J Gonzalez Garcia^2^, H Stellbrink^3^, Y Yazdanpanah^4^, G Richmond^5^, G Smith^6^, K Sutton^1^, D Dorey^7^, F Zhang^8^, K Smith^9^, P Williams^10^ and W Spreen^11^**

^1^Clinical Development, ViiV Healthcare, Research Triangle Park, NC, USA. ^2^Division of Internal Medicine, Hospital La Paz, Madrid, Spain. ^3^Infectious Diseases, ICH Study Center, Hamburg, Germany. ^4^Infectious Diseases Department, Hôpital Bichat Claude Bernard, Paris, France. ^5^Gary J. Richmond, Fort Lauderdale, FL, USA. ^6^Maple Leaf Research, Ontario, Canada. ^7^Statistics, GlaxoSmithKline, Ontario, Canada. ^8^Statistics, GlaxoSmithKline, King of Prussia, PA, USA. ^9^Global Research & Medical Strategy, ViiV Healthcare, Research Triangle Park, NC, USA. ^10^Compound Development, Janssen Research and Development, Beerse, Belgium

**Background: **Long‐acting (LA) injectable suspensions of cabotegravir (CAB) and rilpivirine (RPV) are in Phase III development. Week (W)160 analysis evaluated long‐term efficacy, safety and tolerability of both IM dosing regimens. At W96, response rates were comparable between treatment arms: injections every 8 weeks (Q8W), every 4 weeks (Q4W) and daily oral CAB 30 mg + ABC/3TC (PO).

**Materials and methods: **Phase IIb, multicenter, parallel‐group, open‐label study in ART‐naïve HIV‐infected adults. Patients with plasma HIV‐1 RNA <50 copies/mL at conclusion of the 20‐week induction period on QD oral CAB + ABC/3TC were randomized 2:2:1 to IM CAB LA + RPV LA Q8W, Q4W or PO in the maintenance period (MP). After W96, patients on IM regimens continued their current MP regimen. Patients randomized to PO in MP chose a Q8W or Q4W IM regimen in the extension period (EP). Data are presented from the MP and EP. Evaluations included: virologic success <50 copies/mL (FDA Snapshot analysis), protocol‐defined virologic failure (PDVF) and safety at the pre‐specified W160 exploratory endpoint (ITT‐maintenance exposed [ME]).

**Results: **Three hundred and nine patients were enrolled (ITT‐exposed): 91% male, 20% non‐white and 19% >100,000 copies/mL HIV‐1 RNA. Two hundred and eighty‐six patients were randomized into the MP, 258 completed MP with 252 entering EP. At W160, 90% (104/115; Q8W) and 83% (95/115; Q4W) of randomized IM patients remained suppressed <50 copies/mL (ITT‐ME) (Table 1). Ninety‐eight percent (43/44) of PO to Q8W/Q4W IM patients remained suppressed. No patient developed PDVF after W48. Excluding injection site reactions (ISRs), nasopharyngitis (38%), diarrhea (22%) and headache (22%) were the most common AEs, with 21% (24/115; Q8W) and 25% (29/115; Q4W) of patients reporting AEs ≥ Grade 3, of which 2% (2/115; Q8W) and 5% (6/115; Q4W) reported drug‐related AEs ≥ Grade 3. Three percent (3/115; Q8W) and 10% (12/115; Q4W) of patients had AEs leading to withdrawal or permanent discontinuation of investigational product. Only 2/230 patients had ISRs leading to discontinuation, none since W24, and no serious ISRs were reported. Majority of ISRs were mild/moderate pain and discomfort with <1% of ISRs classified severe. Fifteen percent (17/115; Q8W) and 18% (21/115; Q4W) reported SAEs, one patient had an SAE that was reported drug related.

**Conclusions: **LA injectable two‐drug therapy, either Q8W or Q4W IM, demonstrated high rates of virologic response, long‐term 3‐year durability and overall tolerability. Differences in outcomes between Q8W and Q4W were primarily due to non‐virologic reasons. Both Q8W and Q4W IM dosing regimens are under evaluation in Phase III studies.

**Table:** Abstract P118 – Table 1. LATTE‐2 week 160 Snapshot study outcomes

Week 160 Snapshot study outcomes (ITT‐ME)^a^	CAB LA + RPV LA Q8W^b^ (n = 115)	CAB LA + RPV LA Q4W^c^ (n = 115)	CAB LA + RPV LA EP‐only Q8W^b^^,^^d^ (n = 34)	CAB LA + RPV LA EP‐only Q4W^c^^,^^d^ (n = 10)
% HIV‐1 RNA <50 copies/mL at W160	104 (90%)	95 (83%)	33 (97%)	10 (100%)
Snapshot virologic non‐response	5 (4%)	0	1 (3%)	0
Data in window not <50 copies/mL	1 (<1%)	0	0	0
Discontinued due to lack of efficacy	1 (<1%)	0	1 (3%)	0
Discontinued due to other reasons while not suppressed	3 (3%)^e^	0	0	0
Snapshot no virologic data	6 (5%)	20 (17%)	0	0
Discontinued due to AE or death^f^	1 (<1%)	12 (10%)	0	0
Discontinued due to other reasons while suppressed	5 (4%)	8 (7%)	0	0

^a^W160 represents 180 weeks on study (20‐week induction with oral CAB 30 mg + ABC/3TC followed by 160‐week maintenance). ^b^Q8W: CAB LA 600 mg + RPV LA 900 mg IM. ^c^Q4W: CAB LA 400 mg + RPV LA 600 mg IM. ^d^patients completing 96‐week maintenance with oral CAB 30 mg + ABC/3TC could continue in extension by switching to IM dosing regimen of their choice (Q8W or Q4W). ^e^includes withdrawn consent (n = 1, intolerance to injections). ^f^Q8W: ISR/chills/body pain (n = 1); Q4W: hepatitis C, rash, depressive reaction, psychotic state, Churg‐Strauss vasculitis, epilepsy (death), mesenteric vein thrombosis, QT prolongation/sinus tachycardia, met liver stopping criteria, coronary artery disease, MI (death), motor neuron disease (all n = 1).

## P119

### B/F/TAF versus ABC/DTG/3TC or DTG + F/TAF in treatment‐naïve adults with high baseline viral load or low baseline CD4 count in two Phase III randomized, controlled clinical trials: Week 96 results

**D Podzamczer^1^, H Stellbrink^2^, C Orkin^3^, A Pozniak^4^, J Arribas^5^, E Koenig^6^, M Ramgopal^7^, A Baumgarten^8^, X Wei^9^, A Cheng^10^, D SenGupta^10^ and H Martin^10^**

^1^Servei de Malalties Infeccioses, Hospital Universitari de Bellvitge, Barcelona, Spain. ^2^ICH Infectious Diseases Center, ICH Study Center, Hamburg, Germany. ^3^Barts Health NHS Trust, Royal London Hospital, London, UK. ^4^HIV Services, Chelsea & Westminster Hospital, London, UK. ^5^Infectious Diseases Unit, Hospital Universitario La Paz, Madrid, Spain. ^6^Instituto Dominicano de Estudios Virologicos, Santo Domingo, Dominican Republic. ^7^Midway Immunology and Research Center, Fort Pierce, FL, USA. ^8^Zibp Zentrum fur Infektiologie Berlin Prenzlauer Berg, Berlin, Germany. ^9^Biometrics, Gilead Sciences, Foster City, CA, USA. ^10^HIV Clinical Research, Gilead Sciences, Foster City, CA, USA

**Background: **Treatment‐naïve, HIV‐1‐infected individuals with high viral load (HIV‐1 RNA) and/or low CD4 count may be difficult to treat. In two Phase III studies of fixed‐dose combination bictegravir/emtricitabine/tenofovir alafenamide (B/F/TAF) versus dolutegravir comparators, there were no treatment differences between arms for subgroups with HIV‐1 RNA >100,000 copies (c)/mL or CD4 < 200 cells/µL at baseline. B/F/TAF was non‐inferior to comparator arms by Snapshot at the primary endpoint, Week (W) 48, and W96. No participant failed with resistance. To further characterize efficacy of B/F/TAF, we analyzed pooled results from these trials for those with high viral load or low CD4 count at baseline. Results were similar among treatment groups at W48; herein, we present results at W96.

**Materials and methods: **Treatment‐naïve, HIV‐1‐infected adults were randomized 1:1 to receive blinded treatment with B/F/TAF (50/200/25 mg) versus dolutegravir/abacavir/lamivudine (DTG/ABC/3TC) (study 1489) or DTG (50 mg) + F/TAF (200/25 mg) (study 1490). Participants were recruited in North America, Europe and Australia. To evaluate the real efficacy of B/F/TAF in these populations, we conducted a per‐protocol (PP) analysis, which included all participants randomized who received ≥1 dose of study medication but excluded those without on‐treatment results in the W96 window (unless discontinued for lack of efficacy) or who had low medication adherence (<2.5th percentile). We present W96 virologic responses by FDA Snapshot algorithm for participants with baseline viral load >100,000 c/mL or CD4 count <200 cells/µL or both using the W96 PP analysis set.

**Results: **Six hundred and twenty‐nine adults were randomized in study 1489 (B/F/TAF n = 314, DTG/ABC/3TC n = 315) and 645 in study 1490 (B/F/TAF n = 320, DTG + F/TAF n = 325). Pooled, 184 participants (PP analysis set) had baseline viral load >100,000 c/mL (B/F/TAF n = 95/634 [15%], DTG/ABC/3TC n = 43/315 [14%], DTG + F/TAF n = 46/325 [14%]), and 122 (B/F/TAF n = 65/634 [10%], DTG/ABC/3TC n = 26/315 [8%], DTG + F/TAF n = 31/325 [10%]) had baseline CD4 count <200 cells/µL. For both high viral load and low CD4 subgroups, virologic suppression (HIV‐1 RNA <50 c/mL) at W96 was similarly high for B/F/TAF, DTG/ABC/3TC and DTG + F/TAF. No participant failed with resistance to any components of study drug.

**Conclusions: **B/F/TAF demonstrated potent viral suppression with no treatment‐emergent resistance in treatment‐naïve adults with high baseline viral load and/or low CD4 count through W96. These data provide further evidence that B/F/TAF is an appropriate treatment for a wide range of patients, including late presenters who have been historically more difficult to treat.

## P120

### Asymptomatic sexually transmitted infections in French HIV MSM: a predictive risk score for screening ‐ ANRS DRIVER study

**M Duracinsky^1^, S Dimi^2^, O Chassany^3^, P Carrieri^4^, V Villes^5^, J Timsit^6^, S Fouéré^6^ amd D Zucman^2^**

^1^Medecine Interne – Patient‐Reported Outcomes, Hôpital Kremlin‐Bicêtre, University Paris‐Diderot, Paris, France. ^2^Medecine Interne, Hôpital Foch, Suresnes, France. ^3^Patient‐Reported Outcomes & Clinical Endpoints, University Paris‐Diderot, Paris, France. ^4^Sciences, Aix Marseille University, INSERM, IRD, SESSTIM, Marseille, France. ^5^Observatoire Régional de la Santé, Provence‐Alpes‐Côte d'Azur, Marseille, France. ^6^Dermatologie ‐ MST, CHU St Louis, Paris, France

**Introduction: **Strategies of screening HIV MSM for asymptomatic sexually transmitted infections (ASTI) vary between recommendations and practice. We aimed to validate a self‐reported predictive score targeting this costly screening.

**Method: **Seven hundred and eighty‐one HIV MSM followed up in hospital provided clinical data and filled a self‐administered questionnaire. We used a binary logistic regression model to evaluate factors associated with ASTI which was defined as screening of at least one of asymptomatic syphilis, chlamydia or gonorrhoea infection. Correlates included clinical, sociodemographic and behavioural characteristics and sexual practices. The accuracy of the model was calculated by non‐parametric area (AUC) under the receiver‐operating‐characteristic (ROC) curve to find the optimal discriminant threshold for screening.

**Results: **One hundred and three patients (13.2%) had a diagnosis of ASTI including syphilis (4.2%), chlamydia (7.6%) and gonorrhoea (6.8%). In the multivariable model, patients who had detectable plasma HIV RNA level (6.8%) were significantly more likely to have an ASTI (OR [95% CI] 2.54 [1.23 to 5.25], *p* = 0.012), after adjustment for sensation seeking behaviour e.g. “I don't like watching porn videos” (OR [95% CI] 1.61 [1.01 to 2.59], *p* = 0.047), no use of condom (and/or latex square) for anal penetration (OR [95% CI] 2.20 [1.36 to 3.56], *p* = 0.001) and for oro‐genital practices (OR [95% CI] 1.83 [1.12 to 3.01], *p* = 0.016), no stable relationship (OR [95% CI] 1.70 [1.01 to 2.66], *p* = 0.019) and risk at group sexual intercourse (OR [95% CI] 2.00 [1.15 to 3.45], *p* = 0.014), during the last six months. AUC was 0.7. With this model sensitivity was 61% and specificity was 31%.

**Conclusion: **We propose predictive model improving ASTI screening strategy among HIV MSM in clinical practice.

## P121

### Integrase inhibitors and virological failure: a cohort analysis

**R Montejano, R De Miguel, J Bernardino, I Perez‐Valero, M Montes, E Valencia, L Martin‐Carbonero, V Moreno, J Gonzalez‐Garcia, J Arribas**

HIV Unit, Hospital La Paz, Madrid, Spain

**Background: **Guidelines recommend integrase inhibitors (INSTI) [in combination with 2NRTIs] as the preferred initial regimen for HIV treatment, owing to their great efficacy and tolerability. Virological failure (VF) in clinical trials of naïve infected patients is less than 2%. However, data of VF in PLWHIV with INSTI‐based regimens (either naive or experienced) in real‐life setting are still scarce. We analysed a large cohort with current INSTI‐based ART, aiming to describe VF in these patients.

**Materials and methods: **Observational retrospective analysis (June 2006 to June 2018) of outpatients attending Hospital La Paz (Madrid). Patients participating in clinical trials were excluded. Relevant clinical information was obtained from our database. VF was defined as two consecutive viral loads (VL) >200 copies/mL in patients with a prior VL <50 copies/mL.

**Results: **We identified 2391 patients with treatments based on INI (418, 17.48% naïve), 83% in combination with 2NRTIs. In order of frequency, RAL was the more frequently prescribed INSTI 41.7%, followed by DTG 39% and EVG 19.2%. FV was detected in 55 patients, of whom 11 had more than one FV, all of them previously treated with RAL. Experienced patients presented more frequently VF, 62.7% versus 7.3% in naïve, *p* = 0.044. Patients with VF were 69.6% males, mean age 45 (IQR 38 to 51) years, Caucasians 85.7%, with time of known HIV infection 15 (IQR 10 to 19) years, 45.5% sexual acquisition, nadir CD4 97 cells/mm^3^ and 69.1% AIDS stage, without finding differences between INSTI. VF occurred in 42 patients with RAL, nine with DTG and four with EVG, respectively 4.2%, 0.9% and 0.9% of all the prescribed treatments that included these drugs (*p* < 0.001). Genotypic testing was obtained in 22/55 patients, of whom 14 had mutations in 14 (64%). The most frequently detected mutations were 151I/L (4/22), 155H (3/22), followed by the combination 140S + 148H (±3rd mutation) (2/22). R263K was detected only in one patient. According to Stanford HIV database, detected mutations determined high resistance to RAL in six patients, to EVG in seven patients and to DTG only in one patient. After the FV, 58.2% currently have CV <50 copies/mL, with median CD4 347 cells/mm^3^ (180 to 638) and 76.4% maintain treatment with INI (19 with 2NRTIs and 15 with analogous‐free regimens), with DTG being the most used 32/42 patients.

**Conclusions: **In our retrospective analysis, VF in INI‐based treatments was infrequent in both naïve and experienced. After VF the majority continued with INI and more than half have undetectable VL currently.

## P122

### Virological outcome after choice of antiretroviral regimen guided by proviral HIV‐1 DNA genotyping in a real‐life cohort of HIV‐infected patients

**A Meybeck^1^, O Robineau^1^, E Alidjinou^2^, T Huleux^1^, A Boucher^1^, P Choisy^1^, L Bocket^2^ and F Ajana^1^**

^1^Infectious Diseases, CH Dron, Tourcoing, France. ^2^Virology, CHRU Lille, Lille, France

**Background:** ****Issues have been raised concerning clinical relevance of HIV‐1 proviral DNA genotyping. To assess impact of proviral DNA resistance test in addition to analysis of historical RNA gentotyping test on choice of ART and subsequent virological outcome, we retrospectively reviewed decision‐making and viral load (VL) evolution following the realisation of each test performed in our centre.

**Materials and methods:** ****We performed a retrospective analysis of all HIV proviral DNA genotypic tests performed in our centre, between January 2012 and December 2017, except those prescribed within the framework of a clinical trial. Variables taken into account were virological success (defined as VL <20 copies/mL) or failure at the time of the test, ART change in the six months following the test, and VL six months after ART change or after the genotypic test in the absence of ART change.

**Results: **A total of 340 tests were performed, 182 (54%) in a context of virological success of the current therapy. By comparison with historical HIV‐RNA resistance test results, 135 (40%) DNA tests did not detect a resistance previously known, but 96 (28%) revealed at least one new resistance. Only 112 (34%) tests were followed by ART change, more frequently in situation of virological success (39% vs. 27%, *p* = 0.03). Changes were mainly a simplification for a single tablet regimen (STR) (33%), a simplification to dual therapy (21%) or a change in the third agent (16%). In situation of virological success, ART change guided by DNA genotyping led to VL >20 copies/mL after six months in 4/73 cases (5%). In situation of virological failure, change guided by DNA genotyping led to VL <20 copies/mL after six months in 19/39 cases (49%). In this last situation, decision to maintain the same treatment led to VL <20 copies/mL in 73/103 cases (71%).

**Conclusions: **Use of HIV‐1 proviral DNA genotyping to guide ART optimisation in situation of virological success seems safe with a low rate of virological failure at six months. DNA genotyping performed in situation of virological failure led less frequently to ART change. In this last situation, choice guided by DNA genotyping to modify or not ART led to virological success in almost two‐thirds of cases. Thus, DNA genotyping seems of interest in both context associated with an analysis of historical RNA genotyping test.

## P123

### Providing evidence from real‐world data ‐ three‐year follow‐up of dolutegravir‐based regimens in routine clinical care in Germany: the final analysis of the DOL‐ART cohort

**N Postel^1^, C Wyen^2^, H Hillenbrand^3^, T Lutz^4^, A Moll^5^, R Pauli^6^, M Sych^7^, B Westermayer^7^, D Lueftenegger^8^ and R Walli^8^**

^1^Prinzmed, Munich, Germany. ^2^Praxis am Ebertplatz, Cologne, Germany. ^3^Praxis City Ost, Berlin, Germany. ^4^Infektiologikum, Frankfurt am Main, Germany. ^5^Praxiszentrum Kaiserdamm, Berlin, Germany. ^6^Isarpraxis, Munich, Germany. ^7^GlaxoSmithKline, Munich, Germany. ^8^ViiV Healthcare, Munich, Germany

**Background: **DOL‐ART is a prospective, three‐year observational German cohort study in HIV‐infected patients initiated on Tivicay (dolutegravir, DTG)‐based ART ≥4 weeks prior to study enrolment. The goal was to provide insights into health care resource utilisation, effectiveness and safety of using DTG in routine HIV care.

**Methods: **Here we present the final results of DOL‐ART focusing on number and type of monitoring measures for patient management, virological effectiveness, persistence of DTG‐based ART, (serious (S)) adverse events (AEs) and adverse drug reactions (ADRs).

**Results: **Four hundred and ten patients (pts) were included in DOL‐ART: 87.1% men, median age 45 years (IQR 36 to 52), 23.2% CDC stage C, 24.1% ART‐naïve prior to DTG‐based ART. Of ART‐naïve pts, 18.2% had <200 CD4/µL, 26.3% harboured ≥100,000 HIV‐RNA copies/mL. Of pretreated pts, 72.0% had <50 HIV‐RNA copies/mL. Main reasons for switch to DTG‐based ART were (multiple responses permitted): side effects on previous ART (31.8%), ART simplification (30.2%), patient wish (24.8%), comorbidities/concomitant medication (12.2%), and virological failure (9.3%). Comorbidities were documented in 55.6% of pts (35.4% of ART‐naïve, 62.1% of pretreated): most prevalent at baseline were depression (29.3%), hypertension (15.6%) and cardiovascular diseases (9.5%). 84.6% of pts received triple ART consisting of DTG plus either TDF/FTC (45.1%) or ABC/3TC (39.5%). Median observation time was 34.8 months with 72.0% of pts remaining under observation for three years. In 28.0% of pts premature study discontinuation was reported, including 14.1% of pts discontinuing DTG. Reasons for stopping DTG are shown in Table 1. In 6.8% of pts, ADRs led to DTG discontinuation. Of pts under follow‐up until final analysis, HIV‐RNA was <50 copies/mL (≤200 copies/mL) in 88.3% (94.9%) (on‐treatment analysis). In total, 258 (S)AEs were reported resulting in an event rate of 0.25 per patient‐year (PPY). Of AEs, 125 were categorised as ADRs (including two SADRs); 66.4% of ADRs occurred in Year 1, 16.8% and 8.0% in Years 2 and 3. Documented physician visits and monitoring measures are shown in Table 2.

**Table:** Abstract P123 – Table 1. Reasons for stopping DTG

DTG discontinuations during three‐year follow‐up and related reasons	% (n/N)
Patients stopping DTG	14.1 (58/410)
Reasons for stopping DTG (multiple responses permitted)	
ADRs	6.8 (28/410)
Patient wish	5.1 (21/410)
Simplification	1.0 (4/410)
Comorbidity/comedication	1.0 (4/410)
Virological failure	0.7 (3/410)
Pregnancy	0.2 (1/410)
Other	2.4 (10/410)

**Table:** Abstract P123 – Table 2. Physician visits and monitoring measures PPY

Documented physician visits PPY; median (IQR, interquartile range)	4.5 (4.2 to 5.1)
Monitoring measures PPY; median (IQR)	
HIV‐RNA/CD4 cell	3.9 (3.4 to 4.2)
Blood count	4.0 (3.5 to 4.3)
Serum chemistry	4.0 (3.5 to 4.3)
Urine tests	1.0 (0.0 to 2.9)
Microbiological tests (including one or multiple tests)	0.7 (0.0 to 2.0)

**Conclusion: **During the course of this three‐year cohort, monitoring measures were mainly related to routine quarterly controls of HIV disease, reflecting local HIV treatment guidelines. DTG discontinuation rates due to ADRs or virological failure were low with 6.8% and 0.7%, respectively – showing a good safety profile and good effectiveness of DTG use in clinical routine. Moreover, ADR rates markedly decreased over time.

## P124

### Effectiveness and persistence of Triumeq (DTG/ABC/3TC) in routine clinical care in Germany: second interim analysis of the prospective German TRIUMPH cohort

**T Wolf^1^, T Heuchel^2^, H Jessen^3^, A Meurer^4^, M Mueller^5^, B Schappert^6^, C Schulz^7^, C Wyen^8^, B Westermayer^9^, D Lueftenegger^10^ and R Walli^10^**

^1^Medizinische Klinik II – Infektiologie, Universitätsklinikum Frankfurt, Frankfurt am Main, Germany. ^2^MedCenter, Chemnitz, Germany. ^3^Praxis Jessen, Berlin, Germany. ^4^Zentrum für Innere Medizin und Infektiologie, Munich, Germany. ^5^Gemeinschaftspraxis Dr. Ulmer, Dr. Müller, Dr. Frietsch, Dr. Roll, Stuttgart, Germany. ^6^Zentrum für Allgemeinmedizin und Geriatrie, Universitätsmedizin Mainz, Mainz, Germany. ^7^Med. Klinik und Poliklinik 2, Klinikum der Universität München, Munich, Germany. ^8^Praxis am Ebertplatz, Cologne, Germany. ^9^GlaxoSmithKline, Munich, Germany. ^10^ViiV Healthcare, Munich, Germany

**Background: **TRIUMPH is a prospective, three‐year observational German cohort study in ART‐naïve and pretreated adult HIV‐1 infected patients (pts) receiving Triumeq, a one‐pill regimen consisting of DTG/ABC/3TC. Primary and secondary outcomes include health care resource utilisation, effectiveness and safety of Triumeq use in routine clinical care.

**Methods: **Here we present the results of the second interim analysis (data cut 31 December 2017), i.e. 27 months after last patient in. Outcomes of interest were frequency/type of monitoring measures (laboratory tests, referrals to specialists), virological effectiveness and persistence of Triumeq.

**Results: **Three hundred and ninety‐one pts (90.0% men, median age 42 years) were eligible for analysis; 40.4% of pts were ART naïve (158/391) before starting Triumeq (17.7% with HIV‐RNA ≥100,000 copies/mL); 59.6% of pts were pretreated (84.5% with HIV‐RNA <50 copies/mL) of whom 19.7% had ≥3 prior regimens and 48.1% switched from PI‐based regimens. Median observation time until data cut was 30.0 months (IQR 27.3 to 32.2 months), with 73.9% of pts remaining under observation. The most common reasons for study discontinuation (multiple responses permitted) were stopping Triumeq (14.8%, 58/391; reasons see Table 1), patient wish (4.6%, 18/391) and lost to follow‐up (7.4%, 29/391). Time to study discontinuation due to adverse drug reactions (ADRs) is shown in Figure 1. HIV‐RNA was <50 copies/mL in 91.4% of pts (288/315) under follow‐up at second data cut (on‐treatment analysis) (ART‐naïve 92.2% (118/128), pretreated 90.9% (170/187)); intention‐to‐treat analysis (mITT, discontinuation=failure, missings excluded) revealed 77.2% (288/373) (ART‐naïve 78.1% (118/151), pretreated 76.6% (170/222)). The median number of documented visits to HIV specialists was 4.5 (IQR 3.9 to 5.2) per patient‐year (PPY). Referrals to other medical specialists (excluding infectiologists) were documented in 61.4% of pts (ART‐naïve 58.2%, pretreated 63.5%) with a median number of 0.9 PPY visits (IQR 0.4 to 1.6) (ART‐naïve 0.8 PPY (0.4 to 1.6), pretreated 1.0 PPY (0.4 to 1.6)).

**Table:** Abstract P124 – Table 1. Reasons for study discontinuation

Triumeq discontinuations until data cut and related reasons	% (n/N)
Patients stopping Triumeq	14.8 (58/391)
Reasons for stopping Triumeq (multiple responses permitted)	
Adverse drug reactions	6.9 (27/391)
Patient wish	6.9 (27/391)
Comorbidity/comedication	0.8 (3/391)
Virological failure	0.8 (3/391)
Simplification	0.0 (0/391)
Pregnancy	0.0 (0/391)
Other	2.8 (11/391)

Abstract P124 – Figure 1. Time to study discontinuation due to ADRs. Circles indicate censoring at the last observation time point.

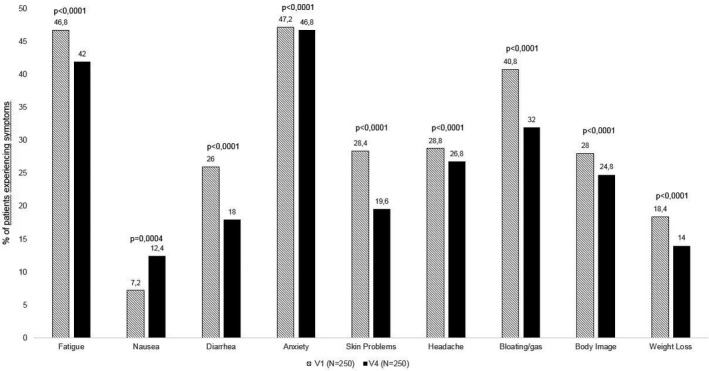

**Conclusion: **The second interim analysis of the TRIUMPH cohort confirmed the high virological effectiveness of Triumeq use in real life over a period of approximately 30 months, showing low discontinuation rates for intolerance (6.9%) and virological failure (0.8%). Moreover, TRIUMPH provides insights in resource utilisation in HIV care such as the need for specialist consultations in about two‐thirds of pts and quarterly routine visits, as recommended in local HIV treatment guidelines.

## P125

### Long‐term outcomes in patients receiving a NVP‐containing regimen for a median of 17 years

**J Tiraboschi^1^, N Latour^1^, H Knobel^2^, P Domingo^3^, E Ribera^4^, D Giralt^5^ and D Podzamczer^1^**

^1^Infectious Disease, HIV & STD Unit, Bellvitge University Hospital, Barcelona, Spain. ^2^Infectious Disease Service, Hospital del Mar, Barcelona, Spain. ^3^Infectious Disease Service, Hospital Sant Pau, Barcelona, Spain. ^4^Infectious Disease Service, Hospital Vall d´Hebron, Barcelona, Spain. ^5^HIV & STD Unit, Bellvitge University Hospital, Barcelona, Spain

**Background: **To evaluate long‐term outcomes in patients maintaining a NVP‐based regimen for more than 10 years.

**Materials and methods: **Retrospective, multicentre, cohort study including virologically suppressed patients, currently receiving a NVP‐based regimen that had been started at least 10 years previously. Demographic, clinical and analytical variables were recorded.

**Results: **Two hundred and seventy‐four subjects were included. Median (IQR) follow‐up was 17.1 (13.8 to 18.5) years. Mean age at baseline was 40 years, mean (SD) HIV viral load (VL) and CD4 count  was 40,827 (175,570) copies/mL and CD4 492 (328)/μL respectively. One‐third of the patients initiated a first ARV treatment including NVP (33%), 25% suppressed patients switched to NVP while in 42% pretreated subjects initiated a NVP‐containing regimen with detectable HIV VL. At baseline, NVP was frequently combined with lamivudine (3TC)/zidovudine (36.6%) or 3TC/stavudine (25.2%). Dyslipidaemia (29.9%), hypertension (11.4%) and diabetes (8%) were the most common reported comorbidities at baseline and during follow‐up. Coronary artery disease (2.9%), chronic kidney disease (2.2%) and significant liver damage (2.5%) were less prevalent in this group. Twenty‐one percent of the subjects were HCV+ and 4.7% presented with chronic hepatitis B. At baseline, mean (SD) haemoglobin was 136.8 (26.7) g/L; platelets 218.9 (64.8); ALT 37.8 (35.7) IU/L; total cholesterol 5.1 (1.2) mmol/L; HDL‐c 1.2 (0.3) mmol/L; LDL‐c 3.2 (1.0) mmol/L; triglycerides 2.5 (2.6) mmol/L and serum creatinine 95.6 (21.4) mmol/L. After a median of 17 years of follow‐up we observed a significant increase in general health markers such as haemoglobin and CD4 cells (all *p* < 0.001) as well as a significant reduction in CD8 and ALT (*p* < 0.001). LDL‐c and serum triglyceride levels decreased significantly [−0.2 (1.1) *p* < 0.001 and −0.73 (2.72) *p* = 0.002 respectively]. Median (IQR) time with persistent HIV VL <50 copies was 16 (13 to 18) years. During follow‐up, subjects presented with median (IQR) 1 (0 to 2) blip (HIV VL <1000 copies/mL). Most frequent current combinations are NVP + ABC/3TC in 48.5%, +FTC/TAF 17.5% and +TDF/FTC in 9.1%.

**Conclusions: **Patients receiving NVP therapy for long term present a significant improvement in general health status markers, CD4 response and a durable HIV viral suppression. A favourable lipid profile as well as liver markers improvement was also observed. Based on the extensive experience as well as a good tolerance and efficacy profile, NVP should be considered for treatment continuation in those patients already receiving this inexpensive generic drug.

## P126

### First real‐life data of tenofovir alafenamide (TAF)‐based ART in adult HIV‐1‐infected patients enrolled in the French TARANIS cohort: results on the use of E/C/F/TAF (Genvoya®)

**J Meynard^1^, C Duvivier^2^, J Molina^3^, F Ajana^4^, G Pichancourt^5^, P Morlat^6^, M Revest^7^, I Poizot‐Martin^8^, L Hocqueloux^9^, C Janssen^10^, P Genet^11^, C Katlama^12^, P Leclercq^13^, R Haubrich^14^, F Durand^15^ and S Sahali^15^**

^1^Infectious Disease, Saint Antoine, Paris, France. ^2^Infectious Diseases, Institut Necker, Paris, France. ^3^Infectious Diseases, Saint Louis, Paris, France. ^4^Infectious Diseases, CH de Tourcoing, Tourcoing, France. ^5^Infectious Diseases, CH d'Avignon, Avignon, France. ^6^Infectious Diseases, Saint André, Bordeaux, France. ^7^Infectious Diseases, Pontchaillou, Rennes, France. ^8^Immuno‐Haematology and HIV, Sainte Marguerite, Marseille, France. ^9^Infectious Diseases, La Source, Orléans, France. ^10^Infectious Diseases, CH Annecy Gennevois, Metz Tessy, France. ^11^Haematology and Immunology, Argenteuil, France. ^12^Infectious Diseases, La Pitié Salpétrière, Paris, France. ^13^Infectious Disease, Albert Michallon, La Tronche, France. ^14^Medical Affairs, Gilead Sciences, Inc, Foster City, CA, USA. ^15^Medical Affairs, Gilead Sciences France, Boulogne Billancourt, France

**Background: **Tenofovir alafenamide (TAF), a novel prodrug of tenofovir (TFV) with at least comparable virological potency of TDF, 91% lower circulating levels of plasma TFV and fewer off‐target effects on renal and bone, was approved based on large controlled clinical trials in naïve and experienced subjects. Interim results of a non‐interventional study (TAFNES) on the use of E/C/F/TAF in Germany were communicated at the 2017 EACS conference. As no data are available for patients in routine clinical practice in France, the TARANIS study was developed to evaluate the effectiveness and safety of F/TAF‐based regimens, starting with (Genvoya^®^) E/C/F/TAF, the first approved TAF‐containing combination, in treatment‐experienced (TE) and treatment‐naive (TN) HIV‐infected patients.

**Materials and methods: **TARANIS is an ongoing prospective, observational cohort study in France, which plans to enrol 600 HIV‐1‐infected patients taking E/C/F/TAF (300) or R/F/TAF (300) either as initial therapy or switch from a suppressive regimen in routine care in accordance with the SmPC. Of clinical outcome variables, only data assessed during the routine management of patients were captured in the eCRF. The study objectives are: HIV‐1 RNA and CD4 cell count changes during 24 months and self‐reported health‐related quality of life (HRQoL) using the HIV Symptom Index (SI), SF‐36 and HIV Treatment Satisfaction (TS) questionnaires. Here we present preliminary results in patients taking E/C/F/TAF with 3‐month follow‐up at time of data cut.

**Results: **The analysis population consists of 298 patients taking E/C/F/TAF. The majority of patients (78%) were TE: 30% with (Stribild^®^) E/C/F/Tenofovir disoproxil fumarate (TDF), 39% with other TDF‐containing regimens. Baseline characteristics for TN and TE patients are in Table 1. At Month 3, HIV RNA was <50 copies/mL in 86% of TN and 95% of TE patients. Median changes in CrCl (eGFR mL/mn) were −6.5 and −2.4 in TN and TE respectively. Overall, mean HIV SI changes were −5.2 in TN and −3.2 in TE. Mean changes in SF‐36 were respectively: mental, +0.8 and +0.7 in TN and TE; physical, +4.1 and +0.6 in TN and TE. For the TE population, the mean Month 3 TS change was +19.3 (general satisfaction/clinical subscale +9.8; lifestyle/ease subscale +9.5).

**Table:** Abstract P126 – Table 1. Baseline characteristics in patients initiating or switching treatment to E/C/F/TAF

Treatment naive	Treatment experienced
N (%)	65 (21.8)	233 (78.2)
Male gender, n (%)	59 (90.8)	178 (76.4)
Median age, years (IQR)	37 (29 to 49)	49 (41 to 55)
Median CD4 count, cells/µL (IQR)	404.5 (205.0 to 578.0)	687.0 (459.0 to 860.0)
CD4 count <200 cells/µL, n (%)	12 (23.1)	11 (5.8)
Median log HIV RNA (IQR)	4.6 (3.9 to 5.1)	NA
CV ≤100,000 copies/mL, n (%)	45 (72.6)	NA
CV >100,000 copies/mL, n (%)	17 (27.4)	NA
HIV‐RNA level <50 copies/mL, n (%)	NA	198 (89.2)
Median serum creatinine, µmol/L (IQR)	77.30 (68.00 to 88.00)	83.95 (72.30 to 94.00)
Median eGFR (MDRD), mL/min/1.73 m^2^ (IQR)	101.65 (87.00 to 118.01)	84.92 (74.74 to 97.66)
eGFR (MDRD) <60 mL/min/1.73 m^2^, n (%)	0 (0.0)	12 (5.6)
Median CrCl (Cockroft‐Gault), mL/min (IQR)	112.38 (95.00 to 131.49)	95.13 (80.69 to 113.69)
CrCl (Cockroft‐Gault) <60 mL/min, n (%)	0 (0.0)	4 (1.9)
Reasons for switch to E/C/F/TAF, n (%) (multiple responses allowed)	NA
Simplification of ART	NA	100 (42.9)
Patients preference	NA	27 (11.6)
Side effects of current ART	NA	82 (35.2)
Other	NA	74 (31.8)

IQR = interquartile range; NA = not applicable.

**Conclusion: **In 298 patients treated with E/C/F/TAF, 69% were pretreated with TDF‐based regimens. In this preliminary analysis of 3‐months follow‐up data, E/C/F/TAF showed a high level of efficacy consistent with previous studies. While eGFR/CrCl remained stable, HIV symptom index and treatment satisfaction improved.

## P127

### Long term non‐progressors and elite controllers: starting antiretroviral therapy in routine HIV care

**A Akhlaq, J Schafers, H Farooq, C Van Halsema**

Infectious Diseases, North Manchester General Hospital, Manchester, UK

**Background: **All HIV‐positive individuals are now advised to take ART [1], based on evidence from the START study and others [2]. However, there is little evidence for benefits of immediate ART for those with very low viral loads (VL) and stable CD4 counts, known as long‐term non‐progressors (LTNP) and elite controllers (EC) [3].

**Methods: **We identified those defined by clinicians as LTNP/EC by searching clinical records from 2000 to 2015 for “progressor” or “elite” in a regional centre providing care for around 2600 HIV‐positive individuals. Limiting to those fulfilling definitions for LTNP (CD4 > 500 for seven years off ART) and EC (undetectable VL off ART, ≥2 readings in 12 months). Data were collected by medical staff using a proforma, including demographics, disease progression, ART start and reasons for starting. Analysis is descriptive, using Excel and Stata.

**Results: **We identified 52 individuals; median age at diagnosis 32 years (IQR 28 to 38), 62% male. HIV was diagnosed between 1982 and 2012, with median baseline VL 779 copies/mL (IQR 326 to 10,300) and CD4 count 680 cells/µL (IQR 521 to 919). Available follow‐up time was median 13 years (range 7 to 35). The latest median VL was <40 and CD4 649. Of the group, 50/52 were LTNP (96%), of whom 34/50 started ART during observation time. Two EC did not start ART. The LTNP group held LTNP status for median 10 years (range 7 to 25). The primary reason for commencing ART was loss of LTNP status (26, 50%), with additional factors, e.g. cardiovascular risk, contributing. One started treatment as prevention and two because of START trial findings (4%, starting in 2016 to 2017 with CD4 counts 806 to 1000 at the time). Ten individuals (19%) developed opportunistic infections, including one *Pneumocystis* pneumonia, two TB lymphadenitis, one severe pneumococcal pneumonia, the latter after ART start. Three developed malignancy and two had died by the time of data collection.

**Conclusions: **We identified 50 LTNP and two EC; most starting ART during the time of observation. The START study prompted some change in management, while others had evidence of disease progression and lost LTNP status. Using routine data has intrinsic limitations and we may not have identified all LTNP/EC in our patient group. Some who seemed likely to be LTNP transferred care elsewhere before sufficient observation time had accrued. Immediate ART may contribute to preventing OIs and malignancies among this patient group.

**References**

[1] British HIV Association. Guidelines for the treatment of HIV‐1 positive adults with antiretroviral therapy [Internet]. 2015 (2016 interim update) [cited 2018 Jun 28]. Available from: http://www.bhiva.org/.

[2] INSIGHT START Study Group; Lundgren D, Babiker A, Gordin F, Emery S, Grund B, Sharma S, et al. Initiation of antiretroviral therapy in early asymptomatic HIV infection. N Engl J Med. 2015;373:795‐807.

[3] Gurdasani D, Iles L, Dillon DG, Young EH, Olson AD, Naranbhai V, et al. A systematic review of definitions of extreme phenotypes of HIV control and progression. AIDS. 2014;28:149‐62.

## P128

### Failure of dolutegravir first‐line cART with selection of R263K and G118R

**N Lübke^1^, B Jensen^2^, F Hüttig^2^, M Obermeier^3^, A Thielen^4^, R Kaiser^5^, J Timm^1^ and D Häussinger^2^**

^1^Institute of Virology, University Hospital Düsseldorf, Düsseldorf, Germany. ^2^Gastroenterology, Hepatology & Infectious Diseases, Heinrich‐Heine‐University, University Hospital, Düsseldorf, Germany. ^3^Medical Center for Infectious Diseases, Berlin, Germany. ^4^Institute of Immunology and Genetics, Kaiserslautern, Germany. ^5^Institute of Virology, University of Cologne, Cologne, Germany

**Background: **The HIV‐1 integrase inhibitor dolutegravir (DTG) is characterised by a high antiviral potency and a high barrier to resistance. Thus, only few cases of virological failure of DTG initial therapy were reported. In these failing patients no DTG‐specific major RAMs were detected [1‐3]. Here, we describe a virological failure in a therapy‐naïve patient receiving FTC, TDF and DTG associated with the emergence of the DTG‐RAMs R263K and the G118R.

**Material and methods**

HIV plasma resistance analyses were performed by ultra‐deep sequencing with the Illumia MiSeq. Drug levels were determined for DTG, FTC and TDF for all available plasma samples.

**Results:** ****A 27‐year‐old MSM infected with an HIV‐subtype F was admitted to the hospital with advanced HIV disease, high HIV viral load (1.4x106 copies/mL) and low CD4 count (22 cells/µL). ART with TDF/FTC plus DTG was initiated with an excellent therapy response indicated by a 3 log decline of VL within the first 3 weeks during hospitalisation and a slow recovery of the CD4 cells. When disseminated tuberculosis (TB) including tuberculous pericarditis was diagnosed a TB treatment comprising isoniazide, rifabutin, ethambutol and pyrazinamide was initiated 1 week after ART start. After discharge from hospital the HIV VL increased to 311,894 copies/mL initially interpreted as an adherence problem. In the course of therapy VL decreased continuously to ˜500 copies/mL, but viral suppression below the detection limit was not achieved. Resistance analysis 8 months after therapy initiation revealed the FTC‐RAM M184V and the DTG‐RAMs G118R and R263K. Retrospective resistance analyses showed the R263K already in Week 14, while the variants carrying the G118R were first selected in Week 26. Both DTG‐RAMs were detected in different frequencies by ultra‐deep sequencing indicating evolution of distinct variants within the quasispecies consistent with distinct resistance pathways [4]. ART drug levels during therapy were adequate between Week 10 and 40 excluding a negative impact of the TB medication [5] on the ART plasma levels. Thus, the low plasma drug levels in Weeks 6 and 38 were most likely caused by insufficient drug adherence. After switching the cART to TDF/FTC/RPV plus DRV/r stable viral suppression was achieved.****

**Conclusion: **Resistance to DTG, especially in therapy‐naïve patients, is a rare event but needs to be considered. Risk factors like HIV‐1‐non‐B subtype [4], high HIV‐load and low CD4 cell count [3], insufficient adherence and co‐infections influencing the drug levels can possibly facilitate the selection of DTG‐resistant variants, in our case the R263K and the G118R substitutions.

**References**

[1] Lepik KJ, Harrigan PR, Yip B, Wang L, Robbins MA, Zhang WW, et al. Emergent drug resistance with integrase strand transfer inhibitor‐based regimens. AIDS. 2017;31:1425‐34.

[2] Sax PE, DeJesus E, Crofoot G, Ward D, Benson P, Dretler R, et al. Bictegravir versus dolutegravir, each with emtricitabine and tenofovir alafenamide, for initial treatment of HIV‐1 infection: a randomised, double‐blind, phase 2 trial. Lancet HIV. 2017;4:e154‐60.

[3] Fulcher AJ, Du Y, Sun R, Landovitz RJ. Emergence of integrase resistance mutations during initial therapy with TDF/FTC/DTG. Conference on Retroviruses and Opportunistic Infections; 2017 Feb 13‐16; Seattle (WA), USA.

[4] Quashie PK, Oliviera M, Veres T, Osman N, Han YS, Hassounah S, et al. Differential effects of the G118R, H51Y, and E138K resistance substitutions in different subtypes of HIV integrase. J Virol. 2015;89:3163‐75.

[5] Dooley KE, Sayre P, Borland J, Purdy E, Chen S, Song I, et al. Safety, tolerability, and pharmacokinetics of the HIV integrase inhibitor dolutegravir given twice daily with rifampin or once daily with rifabutin: results of a phase 1 study among healthy subjects. J Acquir Immune Defic Syndr. 2013 Jan 1;62(1):21‐7

## P129

### Disinvestment strategies for HIV therapies: the role of HTA, an opportunity for patients and clinicians?

**E Garagiola^1^, E Foglia^1^, L Ferrario^1^, G Cenderello^2^, A Di Biagio^3^, B Menzaghi^4^, G Rizzardini^5^ and D Croce^1^**

^1^Centre for Research on Health Economics, LIUC Business School, Cattaneo University, Castellanza, Italy. ^2^Department of Infectious Diseases, Galliera Hospital, Genova, Italy. ^3^Unit of Infectious Diseases, IRCCS San Martino IST Hospital, Genova, Italy. ^4^Department of Infectious Diseases, Valle Olona Hospital, Busto Arsizio, Italy. ^5^Department of Infectious Diseases, Fatebenefratelli Sacco Hospital, Milano, Italy

**Objectives: **Two‐NRTI backbones plus a third agent represents the standard of care for HIV treatment. The introduction of emtricitabine/tenofovir alafenamide (FTC/TAF) improves the therapeutic strategies available in the Italian drugs market. The effects of FTC/TAF introduction and emtricitabine/tenofovir disoproxil fumarate (FTC/TDF) disinvestment was analysed with a health technology assessment (HTA) methodology.

**Methods: **The HTA involved 18 Italian infectious disease department clinicians: they were asked to prioritise the nine EUnetHTA (European Network for Health Technology Assessment) Core Model dimensions. The assessment compared the most used two‐NRTI backbones available in Italy (FTC/TAF, FTC/TDF), and abacavir/lamivudine (ABC/3TC). For each investigated dimension, perceptions were collected using validated questionnaires (a seven‐level Likert scale, from −3 to +3), and economic modelling (budget impact analysis) was performed. Differences among the groups were studied using ANOVA tests. A final appraisal phase, according to the multi‐criteria decision analysis, and involving four decision makers, was simulated. A final score of comparison among three technologies indicated the more advantageous one.

**Results: **Results from questionnaires showed that FTC/TAF ensures a higher percentage of virological and immunological control compared to FTC/TDF and ABC/3TC (1.36 vs. 0.89 and 0.68 respectively; *p* = 0.023). FTC/TAF also presents a better safety profile compared to both FTC/TDF and ABC/3TC (0.93 vs. −0.09 and 0.14 respectively; *p* = 0.04). FTC/TAF decreases the burden of adverse events management (0.85 vs. 0.10 [FTC/TDF] and 0.26 [ABC/3TC], *p* = 0.016). FTC/TAF has a positive social impact (1.13 vs. 0.23 FTC/TDF vs. 0.40 ABC/3TC, *p* < 0.001). From an organisational perspective, FTC/TAF could simplify the overall management of drug complications for hospitals, both in the short term (0.60 vs. −0.13 [FTC/TDF] and 0.01 [ABC/3TC], *p* < 0.001) and in the long run (0.65 vs. −0.18 [FTC/TDF] and 0.03 [ABC/3TC], *p* < 0.001). The economic modelling revealed a 6% cost saving, for the NHS, introducing FTC/TAF and disinvesting in FTC/TDF. FTC/TAF reached the highest appraisal score (0.82 vs. −0.59 [FTC/TDF] and 0.55 [ABC/3TC]).

**Conclusions: **The introduction of FTC/TAF could deliver improvements in efficacy, with a greater chance of virological control, and safety compared with the previous strategies FTC/TDF and ABC/3TC. The presented results suggested FTC/TAF is a dominant alternative. The TDF/FTC disinvestment strategy in the Italian market seems a cost saving solution for the NHS, thus allowing a consequent re‐allocation of economic resources, covering the investment in innovation.

## P130

### HIV‐positive patients with persistent low‐level viraemia in the University Hospital of La Princesa (Spain)

**E Roy, M Ciudad, M Cárdenas, L García‐Fraile, J Sanz, I Santos**

Infectious Diseases, Hospital Universitario de La Princesa, Madrid, Spain

**Background: **The purpose of HIV treatment is to achieve and mantain an undetectable viral load (VL; <50 copies/mL). Despite a greater number of antiretroviral drugs, 4 to 10% of patients [1] have persistent low level viraemia (LLV). The meaning of persistent LLV is not clearly defined specially in terms of prognosis and management [2,3].

**Objective: **To describe the population of patients with persistent LLV of the infectious diseases consultations of the University Hospital of La Princesa. The secondary endpoint was to compare patients with LLV 50 to 199 copies/mL versus LLV 200 to 499 copies/mL. 

**Materials and methods: **Descriptive retrospective study that included patients with al least two consecutive VL between 50 and 499 copies/mL, from 1 January 2017 until 31 December 2017. Patients without antiretroviral treatment, elite controllers and blips were excluded. Demographic, clinical, immunological, virological and analytical variables were analysed. Resistance test and treatment modifications were collected. 

**Results: **One thousand three hundred and fifty‐four patients had VL available in 2017, 84 of them met LLV criteria which corresponds to a prevalence of 6.2% (4.3% with 50 to 199 copies/mL and 1.9% with LLV 200 to 499 copies/mL). 42.9% were on ART with 2 NRTI + 1 INI and 33.3% had previous virological failure. Mean follow‐up was 11 months (interquartile range of 6). Sixty‐nine percent of patients had undetectable viral load during the follow‐up. There were differences between LLV 50 to 199 and LLV 200 to 499 in terms of diabetes prevalence, viral load prior to ART, previous mutations, virogram performance and appearance of INI mutations.  Outcomes at follow‐up by subgroups are shown in Table 1.

**Conclusions: **The prevalence of persistent LLV in our sample is similar to that described in previous series [4]. The appearance of persistent LLV is not negligible and the VL should be monitored closely, as well as evaluate adherence and the possible interactions. In most cases LLV is not associated with ART resistance but it can lead to virological failure, with special attention to the development of INI resistance.

**Table:** Abstract P130 – Table 1. Follow‐up by subgroups

LLV 50‐199 copies/mL (N = 58)	LLV 200‐499 copies/mL (N = 26)
<50 copies/mL	44 (75.9%)	14 (53.8%)
50 to 199 copies/mL	9 (15.5%)	6 (23.1%)
200 to 499 copies/mL	3 (5.2%)	4 (15.4%)
>500 copies/mL	1 (1.7%)	0
Lost	1 (1.7%)	2 (7.7%)
AIDS event	0	0
Non‐AIDS event	4 (6.9%)	4 (15.4%)
Death	0	1 (3.8%)

**References**

[1] Antiretroviral Therapy Cohort Collaboration (ART‐CC); Vandenhende MA, Ingle S, May M, Chene G, Zangerle R, Van Sighem A, et al. Impact of low‐level viremia on clinical and virological outcomes in treated HIV‐1‐infected patients. AIDS. 2015;29:373‐83.

[2] AIDSinfo (Department of Health and Human Services). Guidelines for the use of antiretroviral agents in HIV‐1‐infected adults and adolescents [Internet]. Available from: http://www.aidsinfo.nih.gov/ContentFiles/AdultandAdolescentGL.pdf.

[3] European AIDS Clinical Society (EACS). Guía Clínica EACS [EACS Guidelines] versión 9.0 [Internet]. 2017. Available from: http://www.eacsociety.org/files/guidlines‐9.0‐spanish.pdf.

[4] Bernal E, Gómez JM, Jarrín I, Cano A, Muñoz A, Alcaraz A, et al. Low level viremia is associated with clinical progression in HIV‐infected patients receiving antiretroviral treatment. J Acquir Immune Defic Syndr. 2018;78:329‐37.

## P131

### When to start antiretroviral therapy in HIV‐2: the challenge remains

**M Cardoso, B Pimentel, J Granado, J Vasconcelos, A Miranda, S Peres, T Baptista, K Mansinho**

Serviço de Infeciologia e Medicina Tropical, Hospital de Egas Moniz, Centro Hospitalar de Lisboa Ocidental, Lisbon, Portugal

**Background: ** The prevalence of HIV‐2 in Portugal is 3.3% [1]. HIV‐2 treatment is limited, facing intrinsic resistance to NNRTI and fusion inhibitors, and different response to protease inhibitors. While current guidelines for HIV‐1 recommend treatment for all, that endpoint is not properly defined for HIV‐2 [2,3].

**Materials and methods: **Retrospective observational study of HIV‐2 patients diagnosed between 1985 and 2017 followed at an infectious disease clinic. Statistical analysis processed by Microsoft Excel.

**Results: ** This cohort included 121 patients. Female predominance (66%) was observed and mean age was 58 years. Most patients (69%) are originally from west Africa and 29% are Portuguese. Mean time since diagnosis was 15 years and main reasons that led to diagnosis were: routine blood screen (41%) and pregnancy (21%). The most frequent transmission route was heterosexual contact (88%). At diagnosis, 79% were asymptomatic, mean TCD4 +  count was 515 cells/mm^3^ and 53% had undetectable HIV‐2 RNA (<40 copies/mL). During the study period, 21% were lost to follow‐up, 2% changed hospital and 2% died. Presently 91 patients (75%) are retained in care. From those, 26 (29%) have not started ARV therapy so far. They have been followed for a mean time of 15 years; mean TCD4 +  count at diagnosis was 828 cells/mm^3^ and at the last evaluation 875 cells/mm^3^ (Δ + 47 cells/mm^3^). All of them presented undetectable viral load at diagnosis and presently only one patient has detectable RNA. The remaining 65 (71%) started ARV therapy, by contrast presented a mean TCD4 +  count of 384 cells/mm^3^ at diagnosis (*p* < 0.05) and 40% had undetectable viral load. Mean time between diagnosis and ARV initiation was six years, pointing out that 35% started ARV at diagnosis. During this interval, a mean TCD4+ drop of 31 cells/mm^3^ per year was registered. Concerning current ARV regimens: 40% are on ABC/3TC and 52% on TDF/FTC NRTI backbone, associated with a third ARV – 43% protease inhibitors and 43% integrase inhibitors. Regarding last immunological evaluation, mean TCD4 +  count was 617 cells/mm^3^ (Δ + 233 cells/mm^3^) and 97% presented undetectable viral load. 

**Conclusions: **This cohort revealed a predominance of female patients, most originated from west Africa, infected by heterosexual transmission. After a mean time of follow‐up of 15 years, 29% have not yet required ARV and those who have initiated therapy 71%, after a mean period of six years, showed a significant immunological improvement and virological suppression. This cohort presented a reasonable rate of retention in health (75%).

**References**

[1] Serviço Nacional de Saúde. Relatório VIH e SIDA – situação em Portugal em 2016 [Internet]. 2017. Available from: http://www.insa.min‐saude.pt/relatorio‐infecao‐vih‐e‐sida‐situacao‐em‐portugal‐em‐2016/.

[2] Marlink R, Kanki P, Thior I, Travers K, Eisen G, Siby T, et al. Reduced rate of disease development after HIV‐2 infection as compared to HIV‐1. Science. 994;265:1587‐90.

[3] Miranda AC, Mansinho K. HIV‐2 infection in Europe, epidemiology of. In: Hope T, Richman D, Stevenson M, editors. Encyclopedia of AIDS. New York (NY): Springer; 2016.

## P132

### HIV elimination from reservoirs: viral dynamics during suppressive ART

**F Otte^1^, K Metzner^2^ and T Klimkait^1^**

^1^Department of Biomedicine, University of Basel, Basel, Switzerland. ^2^Institute of Medical Virology, University Hospital Zurich, Zurich, Switzerland

**Background: **With HIV‐related health issues rising globally, new approaches for sustainable therapy and efforts towards a cure have reached research agendas worldwide. Cure strategies like latency reversal have garnered much interest, but are quite unselective yet. Our research strategy takes a different approach by following the changing viral properties of HIV‐1 over the course of infection and during suppressive therapy. Recently our laboratory demonstrated that envelope properties of HIV correlate with immunological recovery and disease outcome. In particular, although the presence of CXCR4‐tropic (X4) virus correlates with poorer outcomes, effective therapy seems to facilitate a superior control of X4 viruses [1]. Based on these unexpected observations, this study intends to undertake a detailed cellular characterisation of viral properties of key T‐cell populations in order to identify critical lymphoid compartments responsible for the observed selective elimination of X4‐infected cells during ART.

**Materials and methods: **Our preliminary study used MACS technology for the analysis of peripheral blood of arbitrarily chosen HIV‐positive patient samples from the Swiss HIV cohort study. Non‐relevant CD8 +  (CTLs) and CD19 +  (B‐cells) were depleted and CD8‐CD19‐ cells were selected for CD4 and integrin B7 (gut homing) or CCR7 (lymph node homing) (Figure 1). Proviral loads (pVLs) were determined by validated qPCR. For multi‐dimensional data visualisation, the tSNE plugin of FlowJo was used.

**Results: **Taking total HIV DNA as a proxy for reservoir size, preliminary results obtained by MACS show a significant enrichment of proviruses in CCR7 + /ß7 + (CD8‐CD19‐) cell fractions. For further FACS studies on living cell sorts we have expanded our cells of interest after separation in order to yield sufficient numbers for subsequent analysis, and first results indicate that the expansion barely affects the expression of our receptors of interest. Preliminary analysis with tSNE confirmed that ≤0.5% Gag positive cells are needed to evaluate the relevance of our markers of interest.

Abstract P132 – Figure 1. (A) MACS CD4 + CCR7 selected cells: shown are proviral loads of four individual HIV‐positive patients illustrated by boxplots. X‐axis indicates cell fraction ID (baseline means pre‐sort condition, CD8 + CD19 +  have still minority of CD4 +  cells present). Y‐axis shows HIV‐1 DNA/10^6 cells. Patient ID is illustrated by greyscale colours. (B) MACS CD4 +  integrin ß7 selected cells: shown are proviral loads of four individual HIV‐positive patients illustrated by boxplots. X‐axis indicates cell fraction ID (baseline means pre‐sort condition, CD8 + CD19 + have still minority of CD4 + cells present). Y‐axis shows HIV‐1 DNA/10^6 cells. Patient ID is illustrated by greyscale colours.

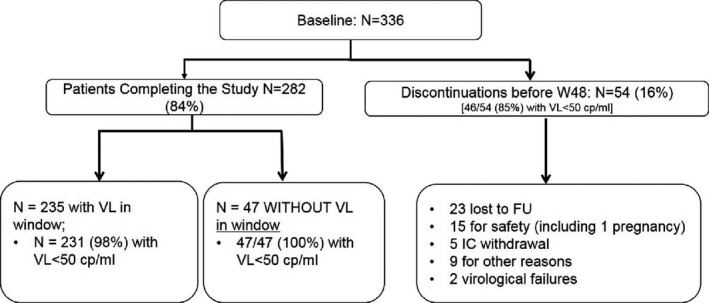

**Conclusions: **Our first cell selection approach already suffices to assess central markers for viral sanctuaries. With the optimised culture conditions, we will widen our marker panel using beside other markers intracellular Gag staining as a proxy for proviral intactness on specifically selected patient samples (suppressed, high pVL) at pre‐ and post‐cART era. Relevant markers identified by tSNE will be further used for live cell sorts. We intend to reactivate viruses from sorted cell fractions to determine viral phylogeny and tropism to find potential links to cellular compartmentalisation and HIV‐1 viral dynamics. Ultimately, we believe our results may contribute to strategies towards targeted cellular virus elimination and HIV eradication.

**Reference: **[1] Bader J, Däumer M, Schöni‐Affolter F, et al. CID. 2016.

## P133

### ART improves most domains of HRQoL by both SF‐36 and HAT‐QoL scales

**L Lins, A Ledo, E Luz, B Soares Dutra, I Rodriguez Prieto, C Brites**

Research Laboratory of Infectious Diseases, Edgard Santos Federal University Hospital, Salvador, Brazil

**Introduction: **HIV+ patients face psychological problems, depression, social stigma and social‐economical disparities that can affect their health‐related quality of life (HRQoL). A few longitudinal studies have evaluated the effect of antiretroviral therapy on the quality of life of HIV+ patients.

**Objective: **To evaluate the quality of life in HIV patients before and after one year of ART.

**Methods: **A prospective cohort study included 91 HIV+ treatment‐naïve patients. We accessed a generic and a disease‐targeted instrument to measure the HRQoL of HIV+ patients, the 36‐Item Short‐Form Health Survey (SF‐36) and the HIV/AIDS‐Targeted Quality of Life Instrument (HAT‐QoL), respectively. The HRQoL and HAT‐QoL of patients aged ≥18 years was evaluated before and one year after initiation of ART. SF‐36 scores were normalised and aggregated into either Physical Component Summary (PCS) or Mental Component Summary (MCS). We collected information on demographics, socioeconomic status, clinical history, HIV‐1 RNA plasma viral load and CD4/CD8 cells count. Dependent t‐tests were used to compare differences between levels of HRQoL before and after one year of ART. Multiple regression technique was used to analyse the relationship between covariables and HRQoL.

**Results: **Most patients (78.0%) were male, mean age of 35.0 ± 10.7 years. Only 18 (19.8%) had a stable relationship, 41 (45.1%) received less than one minimum wage and 32 (35.2%) lived alone. Most of SF‐36 mean scores were significantly higher after one year, particularly Physical Function (*p* = 0.0001), General Health (*p* = 0.0001), SF (*p* = 0.0001), Mental Health (*p* = 0.001) and MCS (*p* = 0.004). Life Satisfaction (*p* = 0.0001) and Disclosure Worries (*p* = 0.0001) HAT‐QoL domains scores were lower at the second evaluation, while Overall Function and Sexual Function were higher (*p* = 0.0001) than that observed at baseline. Age (*p* = 0.002; *p* = 0.045), sex (*p* = 0.019; *p* = 0.029) and having a stable relationship (*p* = 0.010; *p* = 0.022) were associated with the variation in BP and GH, respectively, in the multiple regression analyses; sex (*p* = 0.001) and having a stable relationship (*p* = 0.040) were good predictors of the Mental Component Summary. Sex (*p* = 0.038) and age (*p* = 0.001) predicted the Physical Component Summary. Physical Function and Overall Function showed the only strong correlation between the two scales domains (*r* = 0.73).

**Conclusions: **ART improved HRQoL scores after one year of use. HATQoL and SF‐36 are good tools for evaluation of HRQoL in HIV patients, but they measure different aspects. Use of both scales is recommended for an accurate evaluation of HRQoL in HIV patients.

## P134

### Using Climate‐HIV to describe real‐world clinical outcomes for people living with HIV on dolutegravir‐based regimens

**C Okoli^1^, A Schwenk^2^, M Radford^3^, M Myland^4^, S Taylor^5^, J van Wyk^1^, J Barnes^5^, A Fox^6^, I Reeves^7^, S Munshi^8^, A Croucher^7^, F Grimson^4^, A Paice^1^, N Boxall^4^, A A Darley^6^ and P Benn^1^**

^1^Medical, ViiV Healthcare, Middlesex, UK. ^2^Alexander Pringle Centre, North Middlesex University Hospital NHS Trust, London, UK. ^3^Health Outcomes, ViiV Healthcare, Middlesex, UK. ^4^Real World Insights, IQVIA, UK & Ireland, London, UK. ^5^Birmingham Heartlands HIV Service, Birmingham Heartlands Hospital, Birmingham, UK. ^6^Nottingham University Hospitals NHS Trust, Nottingham, UK. ^7^Homerton University Hospital NHS Foundation Trust, London, UK. ^8^Pharmacy, Homerton University Hospital NHS Foundation Trust, London, UK

**Background: **Dolutegravir (DTG) is a second‐generation integrase strand transfer inhibitor (INSTI) indicated for the treatment of HIV‐1 infection. With several Phase III clinical trials reporting viral load suppression rates >80% and low discontinuation rates at primary endpoint, DTG‐based regimens (DBR) are a recommended first‐line treatment option in international HIV treatment guidelines. Climate‐HIV is an electronic patient record designed to support the management of PLWHIV. The retrospective study aims to describe the real‐world use, effectiveness and safety of DTG in routine clinical practice in several UK HIV services.

**Methods: **Patients prescribed a DBR were included if they were aged ≥18 years and attended one of four large UK HIV units using Climate‐HIV between December 2012 and February 2018. Data for these patients were included from DBR initiation until their last recorded visit, i.e. until subjects were lost to follow‐up (LTFU) or switched from or discontinued DTG. Data regarding demographics, ARV regimens, virological outcomes, adverse events (AEs) and reasons for switch were collected.

**Results: **Nine hundred and thirty‐four of 5590 (17%) patients were prescribed a DBR, 880 (94%) once daily. Two hundred and seventeen (23%) were naive and 717 (77%) were treatment experienced (TE) at DBR initiation. Baseline characteristics are shown in Table 1. The most commonly prescribed DBR was DTG/ABC/3TC (Triumeq^®^) in 533 (61%) of 880. Of the 934 patients initiating DBR, 809 (87%) remained on DBR at last visit; the median duration was 12.6 months [interquartile range (IQR) 4.4 to 22.8]. Ninety‐four (10%) patients switched to a non‐DBR and 31 (3%) patients discontinued ARV treatment without restarting and were classed as LTFU. Median time to switching off DBR was 101 days (IQR 30 to 245); common reasons for switching included adverse events [16/934 (1.7%)], clinician's decision [15/934 (1.6%)] and cost reduction [13/934 (1.4%)]. At last visit, 797/934 (85.3%) patients had an undetectable viral load.

**Table:** Abstract P134 – Table 1. Baseline characteristics

	Overall (N = 924)	DTG as first regimen N = 217 (23.5%)	Subjects who switched to a DTG regimen N = 717 (77.5%)
Sex, male, n (%)	597 (63.9)	159 (27)	438 (73)
Age, years, median (IQR)	43 (34 to 51)	37 (30 to 45)	44 (35 to 52)
Median time (m) since diagnosis to initiation of DTG	90.1 (21 to 161)	8.5 (0.8 to 57)	119.5 (44 to 177)
Proportion of black and minority ethnicities, n (%)	520 (57)	107 (21)	413 (79)
First database recorded HIV viral load	18278.5 (589 to 100509)	24945 (3637.5 to 123800)	14648 (168 to 96152)
Median HIV‐1 RNA at start of DTG regimen (IQR) (copies/mL)	40 (40 to 6868)	23801 (4177 to 115848)	40 (40 to 107)
First database recorded CD4 count (IQR)	258 (20 to 491)	309 (1 to 564)	240 (39 to 459)
Median CD4 at start of DTG regimen (IQR) (cells/mm^3^)	258 (20 to 491)	309 (0.8 to 564)	240 (39 to 459)
Age < 50 yrs, n (%)	667 (71)	178 (27)	489 (73)
≥ 50 yrs, n (%)	267 (29)	39 (15)	228 (85)
Once daily DTG, n (%)	880 (94)	199 (23)	681 (77)
Vl at last recorded visit			
Undetectable (<50 copies/mL)	797 (85.3)	170 (78.34)	627 (87.45%)
Suppressed (50 to 200 copies/mL)	49 (5.25%)	13 (5.99%)	36 (5.02%)
Unsuppressed (>200 copies/mL)	88 (9.42%)	34 (15.67%)	54 (7.53%)
Most common DTG ART‐combination partners			
Abacavir/lamivudine, n (%)	533 (61)	136 (26)	397 (74)
Emtricitabine/tenofovir, n (%)	178 (20)	43 (24)	135 (76)

Abstract P134 – Figure 1. Kaplan‐Meier analysis for time to switch or discontinue for DTG based regimens.

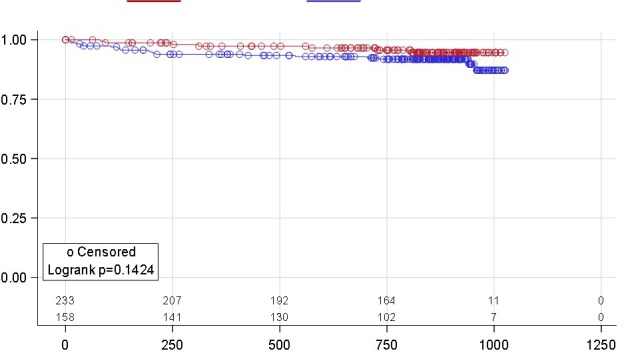

**Conclusion: **This study observed low discontinuation and high effectiveness of DBR in a diverse population within the UK. These real‐world data are broadly consistent with the safety and efficacy data from Phase III clinical trials. Overall persistence of DTG was 87% over a median duration of 12.6 months.

## P135

### NNRTI raltegravir lamivudine (NRL): the NatuRAL choice for ageing patients

**I Cormack**

HIV Department Heath Clinic, Croydon University Hospital, Croydon, UK

**Background: **Ageing patients with HIV may not be suitable for abacavir (ABC)‐ or tenofovir (TDF)‐based HAART. There is a growing need for new treatment strategies.

**Methods: **The joint HIV renal clinic identified 23 HIV‐positive patients on NNRTI raltegravir lamivudine (3TC) HAART and one patient who was on NNRTI + raltegravir (no 3TC). We calculated eGFR using CKD‐EPI on all patients pre‐starting NRL and compared this to current eGFR or eGFR before switching off NRL. Demographic, clinical and baseline data were also collected from patient records. There are 11 men and 13 women average age 64 years (31 to 94) 88% over age 50 years. Ten of 24 (42%) diabetic, 14/24 (58%) hypertensive, 4/24 (16%) HIVAN and 11/24 (46%) had CKD3 or worse pre‐switch to NRL. Fifteen of 24 (58%) had a baseline HIV VL >100,000.

**Results: **All 24 patients are currently virologically suppressed on NRL including one patient who is not on 3TC. Total length of time on NRL is 3121 weeks, average 130 weeks (19 to 375) 18/24 > 48 weeks (75%). Three patients were new starters with pre‐treatment VLs 12,436, 22,000, 60,500 and have been on NRL for 92, 213 and 251 weeks respectively. Ten switched to NRL from TDF and 11 switched from ABC or protease inhibitors (PIs). NNRTIs: efavirenz (14) nevirapine (five) rilpivirine (three) etravirine (two). Pre‐switch 11/24 (46%) had eGFR <60 mL/min. Post‐switch 7/24 (29%) had eGFR <60 mL/min. Overall 15/24 (62.5%) patients had eGFR improvement post‐switch. Of the 10 patients who switched off TDF 80% showed eGFR increase. Interestingly four of these 10 were later switched from NRL to tenofovir alafenamide (TAF)‐based single tablet regimen (STR). All four were eGFR >60 mL/min on NRL. Post‐TAF STR switch all showed loss in eGFR on average 18 mL/min (9 to 27) with average length TAF STR treatment 55 weeks (33 to 84 weeks). Three of four (75%) of these patients now have an eGFR <60 mL/min. Tolerability: all four patients were switched off NRL for simplification reasons after an average time of 136 weeks (range 19 to 375 weeks). Twenty of 24 (83%) patients have remained on NRL for average time 128 weeks (15 to 336 weeks) showing they are able to tolerate the pill burden of 4 to 5 tablets.

**Conclusion: **NRL is an effective HAART option that is well tolerated with good preservation of renal function making it an ideal choice for ageing patients. Seventy‐nine percent of these NRL patients are on generic NNRTI and 3TC is generic making NRL extremely cost‐effective.

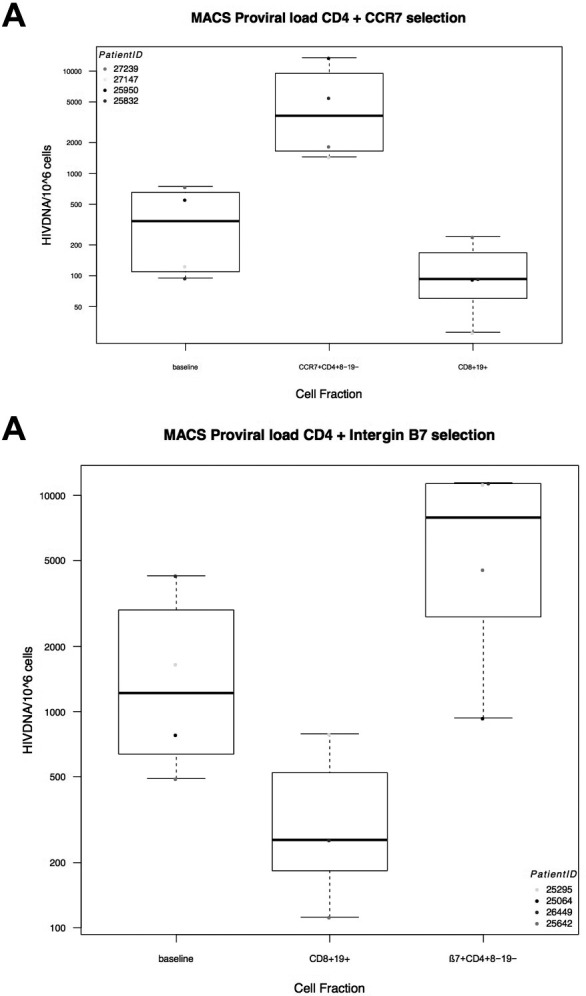

## P136

### Virological response in HIV‐1‐infected patients treated with dolutegravir‐containing ART regimens: a real‐world study

**I Furtado, S R de Valdoleiros, J Fragoso, O Vasconcelos, M Gonçalves and R Sarmentoe Castro**

Serviço de Infeciologia, Centro Hospitalar do Porto, Porto, Portugal

**Background: **Dolutegravir (DTG) is an effective antiretroviral, associated with rapid virological responses [1]. Intermittent viraemia has been linked to a higher risk of virological failure [2] and immune activation [3]. The precise consequences and factors responsible for this phenomenon remain controversial.

**Material and methods**

Retrospective, observational study of HIV‐1‐infected adults starting DTG between May 2015 and May 2017, aiming to evaluate the virological responses. Baseline, 4, 12, 24 and 48‐week data were analysed, including the incidence of blips and low‐level viraemia (LLV), immunological progression and tolerability. The population was divided into three groups: ART‐naïve, ART‐experienced virologically suppressed at switch to DTG and ART‐experienced not suppressed at switch to DTG, defined as a viral load (VL) ≥200 copies/mL. Patients with insufficient follow‐up information and irregular adherence were excluded (n = 65).

**Results: **Within the 227‐patient population, 55 (24.2%) were ART‐naïve and 172 (75.7%) switched from other regimens (Table 1). Naïve patients had a mean VL at baseline of 568,573 copies/mL. A VL <50 copies/mL at Week 48 was observed in 94.3% of them, but <20 copies/mL in only 70.3% (Figures 1 and 2). Of the 164 (72.2%) experienced suppressed patients, 95.1% showed a VL <50 copies/mL at Week 48 and 83.7% <20 copies/mL. During follow‐up, 21.3% had blips >20 copies/mL (5.4% >50 copies/mL) and 6.7% maintained a LLV >20 copies/mL (1.8% >50 copies/mL). A mean VL of 87,977 copies/mL was observed in the eight (3.5%) experienced non‐suppressed patients. Virological suppression below 50 copies/mL at Week 48 was seen in 75.0% and 62.5% under 20 copies/mL. Of the patients with a VL >100,000 copies/mL (24 naïve and two experienced; 65% under regimens with ABC/3TC), six (23.1%) were suppressed <20 copies/mL at Week 48 and 23 (88%) <50 copies/mL. Patients with a VL <100,000 copies/mL had a higher probability of suppressing below 20 copies/mL at Week 48 (*p* < 0.05). Fourteen patients (6.2%) presented adverse effects, leading to discontinuation in four. Eight patients stopped DTG for other reasons and four developed cancer, one of which died.

**Table:** Abstract P136 – Table 1. ART regimens initiated

ART regimen	ART‐naïve (n = 55) (%)	ART‐experienced, virologically suppressed (n = 164) (%)	ART‐experienced, not suppressed at switch (n = 8) (%)
ABC/3TC + DTG	44 (80.0)	113 (68.9)	2 (25.0)
TDF/FTC + DTG	9 (16.4)	22 (13.4)	1 (12.5)
Other regimens	2 (3.6)	29 (17.7)	5 (62.5)

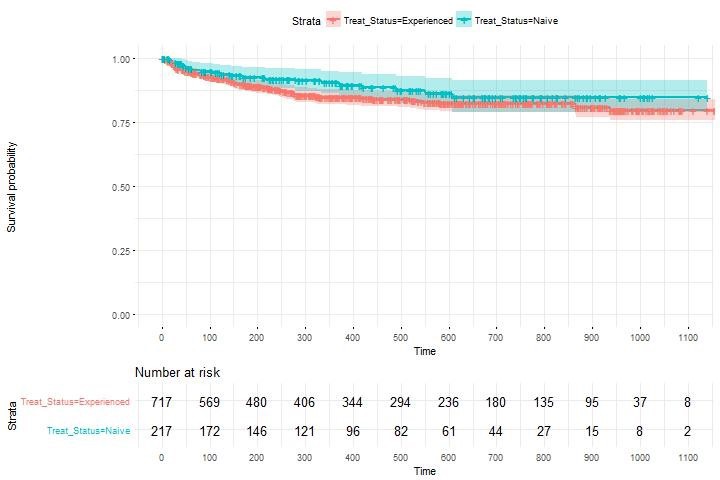

**P136 – Figure 1.** Viral load evolution.

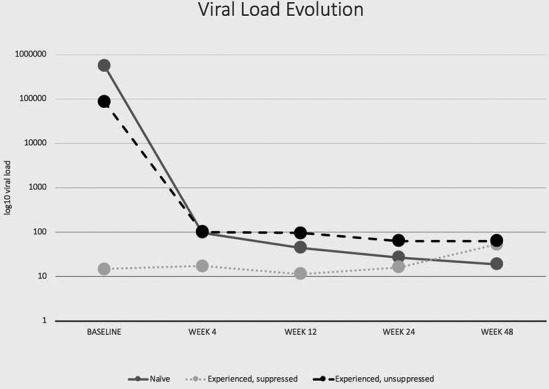

**Abstract P136 – Figure 2.** **CD4** lymphocytes evolution.

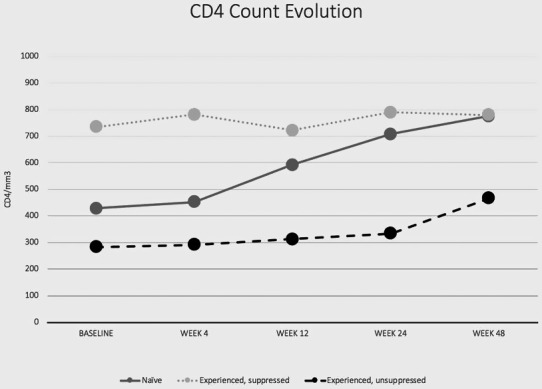

**Conclusions: **The use of dolutegravir in naïve patients was associated with a 70.3% rate of viral suppression under 20 copies/mL at Week 48, and patients with a VL below 100,000 copies/mL appeared to have a higher probability of suppressing below 20 copies/mL. Experienced suppressed patients frequently (28.0%) developed intermittent viraemia above 20 copies/mL. Further investigations are needed to clarify its significance.

**References**

[1] Todd S, Rafferty P, Walker E, Hunter M, Dinsmore WW, Donnelly CM, et al. Early clinical experience of dolutegravir in an HIV cohort in a larger teaching hospital. Int J STD AIDS. 2017;28:1074‐81.

[2] Laprise C, de Pokomandy A, Baril JG, Dufresne S, Trottier H. Virologic failure following persistent low‐level viremia in a cohort of HIV‐positive patients: results from 12 years of observation. Clin Infect Dis. 2013;57:1489‐96.

[3] Zoufaly A, Kiepe JG, Hertling S, Hüfner A, Degen O, Feldt T, et al. Immune activation despite suppressive highly active antiretroviral therapy is associated with higher risk of viral blips in HIV‐1‐infected individuals. HIV Med. 2014;15:449‐57.

## P137

### The rate of HAART initiation according to the WHO/DHHS guidelines during the years 2002‐2016 in a major HIV/AIDS centre in Israel

**D Elbirt, M Otman, S Bezalel‐Rosenberg, K Mahlab‐Guri, S Nemet, M Burke, I Asher and Z Sthoeger**

Neve Or AIDS Center, Kaplan Medical Center, Rehovot, Israel

**Background: **Along the years, the indications for HAART initiation (according to the CD4 cell counts) had been changed. In the present study we define the rate of HIV patients in a major Israeli AIDS centre who initiated HAART according to the WHO/DHHS guidelines.

**Materials and methods: **A retrospective study of all patients with a new diagnosis of HIV during the years 2002 to 2016 in a major Israeli AIDS centre. Patients younger than 18 years of age, with acute HIV or with less than one year of follow‐up were excluded.  Treatment initiation according to the WHO/DHHS guidelines (“Treatment at target”) was defined as HAART initiation within 10% of the recommended CD4 cell counts for each study year.

**Results: **Five hundred and twelve patients (mean 34.13 yearly), 57% males, 43% females, participated in our study. Their mean age at the time of HIV diagnosis was 38.04 ± 13.01 (18 to 81) years and the mean follow‐up period was 7.5 ± 3.84 (1 to 16) years. Overall, 85% of our patients initiated HAART within 3 months of HIV diagnosis. The latter rate increased from 60% at 2002 to 2004 to 94% at 2014 to 2016. However, only 14% and 8% of our patients initiated HAART according to the WHO or the DHHS guidelines, respectively (“Treatment at target”). The above low rates did not increase along the years of the study. The main (89%) reason for “late” HAART initiation was low CD4 cell counts at the time of HIV diagnosis (mean CD4 cells 257 ± 210 [2 to 1140] cells/µL; 46% of the patients with less than 200 CD4 cells/µL). The number of CD4 cell counts at the time of HIV diagnosis did not increase along the years of the study. No significant differences in mortality and hospitalisation rates were observed between the group that initiated HAART according to the WHO/DHHS guidelines and the patients with late treatment initiation. However, the former group of patients revealed a better immune reconstitution with significantly higher CD4 cell counts at the end of the study.   

**Conclusions: **Most of our new HIV patients who were diagnosed with HIV during the years 2002 to 2016 did not initiate HAART according to the WHO/DHHS guidelines due to late presentation with low CD4 cell counts at the time of HIV diagnosis. An immediate, comprehensive and intensive national screening programme(s) for early diagnosis of HIV is mandatory in order to prevent new HIV infections as well as for the benefit of the patients.

## P138

### Raltegravir, elvitegravir, dolutegravir comparison and CD4/CD8 ratio normalisation in HIV‐infected patients

**F Sarigul, U User and N Oztoprak**

Infectious Disease and Clinical Microbiology, Health Sciences University, Antalya Education and Research Hospital, Antalya, Turkey

**Objectives: **In the current era of effective combination antiretroviral regimens (cART), optimal immune restoration and viral suppression have become the primary goals. Here, we aimed to investigate comparison of three integrase strand transfer inhibitors (INSTI) with CD4/CD8 ratio normalisation and virological suppression in HIV‐1 infected patients.

**Methods: **This retrospective comparative case series study was carried out in HIV‐1 positive treatment‐naive patients who initiated cART between 2016 and 2018 with raltegravir (RAL), elvitegravir/cobicistat (EVG) and dolutegravir (DTG) at the clinic of HIV in Antalya‐Turkey. Study drugs were given with coformulated tenofovir/emtricitabine. Patients who completed 12 months of treatment were included in the study (Table 1). We used one‐way analysis of variance (ANOVA) to analyse the differences among group means of three INSTI. In the post‐hoc analysis, Scheffe's method was used for multiple comparisons across INSTI groups.

**Results: **Laboratory and clinical characteristics of the patients used RAL (n = 28), EVG (n = 29) and DTG (n = 32); gender (F/M): 0.11, 0.10, 0.22 (*p* = 0.3549), median age (IQR) 40.9 (19 to 80), 40.8 (22 to 73), 42 (21 to 65) (*p* = 0.9391), median basal CD4 +  T‐cell count (IQR) 416.8 (12 to 1215), 353 (9 to 1218), 376.4 (8 to 819) cells/mm^3^ (*p* = 0.6331), median basal CD4/CD8 ratio (IQR) 0.42 (0.01 to 1.33), 0.44 (0.01 to 1.5), 0.44 (0.03 to 1.15), median basal HIV‐1 RNA (IQR) 2.5 + E5 (5 + E2 to 2.4 + E6), 3.3 + E6 (1.4 + E4 to 6.6 + E6), 7.9 + E6 (65 to 7.8 + E6) copies/mL (*p* = 0.2669) respectively. Therapy response rate at 12th month in patients; did not reveal a statistically significant difference between the CD4/CD8 ratio (*p* = 0.4345), did reveal a statistically significant difference at 1% between HIV RNA levels (*p* = 0.0003). RAL was inferior to the others about virological response (VR) at months 3, 6 and 12. On the other hand, there was no statistically significant difference between EVG and DTG in VR.

**Conclusion: **This study was the first real‐life comparison of three INSTI. Although we had a low number of treatment‐naive subjects and short follow‐up, they had similar activity in immunological response but RAL showed inferior activity in VR at 3, 6 and 12 months.

**Table:** Abstract P138 – Table 1. Baseline demographic and laboratory characteristics of the study patients

Characteristics	Raltegravir	Elvitegravir	Dolutegravir	*p* value
Patient, n	28	29	32	0.3675
Gender (F), n (%)	26 (93)	26 (90)	27 (84)	0.3549
Age, median years (range)	41 (19 to 80)	41 (22 to 73)	42 (21 to 65)	0.9391
Baseline CD4 + T‐cell count, median (range)	417 (12 to 1215)	353 (9 to 1218)	376 (8 to 819)	0.6331
Baseline CD4/CD8 ratio, median (range)	0.42 (0.01 to 1.33)	0.44 (0.01 to 1.5)	0.44 (0.03 to 1.15)	0.9513
Baseline HIV‐1 RNA load, median copies/mL (range)	2.5 + E5 (5 + E3‐2.4 E6)	3.3 + E5 (1.4 + E3‐6.6 + E6)	7.9 + E5 (6.5 + E3‐7.8 + E6)	0.2669

## OPPORTUNISTIC INFECTIONS

## P139

### Efficacy and safety of dolutegravir‐based regimens in advanced HIV‐infected naïve patients: results from a multicentre cohort study

**B Rossetti^1^, G Baldin^2^, G Sterrantino^3^, S Rusconi^4^, A De Vito^5^, A Giacometti^6^, R Gagliardini^7^, M Colafigli^8^, A Capetti^9^, G d'Ettorre^10^, L Celani^10^, F Lagi^3^, A Ciccullo^11^, A De Luca^7^, S Di Giambenedetto^11^ and G Madeddu^5^**

^1^Infectious Diseases Unit, Siena University Hospital, Siena, Italy. ^2^Institute of Clinical Infectious Diseases, Catholic University of Sacred Heart, Policlinico Gemelli, Rome, Italy. ^3^Division of Tropical and Infectious Diseases, Careggi Hospital, Florence, Italy. ^4^Infectious Diseases Unit, DIBIC Luigi Sacco, University of Milan, Milan, Italy. ^5^Unit of Infectious Diseases, University of Sassari, Sassari, Italy. ^6^Infectious Diseases, Ancona Hospital, Ancona, Italy. ^7^Department of Medical Biotechnologies, University of Siena, Siena, Italy. ^8^Infectious Dermatology and Allergology Unit, IFO S. Gallicano Institute (IRCCS), Roma, Italy. ^9^Division of Infectious Diseases, Luigi Sacco University Hospital, Milan, Italy. ^10^Department of Infectious Diseases Public Health, Sapienza University of Rome, Rome, Italy. ^11^Institute of Clinical Infectious Diseases, Catholic University of Sacred Heart, Rome, Italy

**Background: **Dolutegravir (DTG) is recommended in first‐line regimens but limited data are available in advanced naïve HIV‐1 infected patients. We aimed to describe efficacy and tolerability of DTG‐including regimens in ART‐naïve AIDS‐ and late‐presenters in clinical practice.

**Material and methods**

We describe the frequency of immune reconstitution inflammatory syndrome (IRIS) events and estimate (survival analysis) the time to first‐line discontinuation for any reason and to virological suppression (HIV‐1 RNA VL <50 copies/mL) in a multicentre cohort of advanced naïve (AIDS‐presenters or late‐presenters with CD4 < 200/µL) HIV‐1 infected patients starting ART. CD4 changes from baseline during follow‐up were assessed using paired t‐test.

**Results: **We included 272 patients: 120 (44%) AIDS‐presenters and 152 (56%) late‐presenters. One hundred and eighty‐seven (69%) were males, 217 (80%) Caucasian, median age was 48 years (interquartile range [IQR] 38 to 65), VL log10 5.2 (4.8 to 5.7), CD4 + 114 cells/µL (40 to 241). The most frequent AIDS‐defining event was *Pneumocystis jirovecii *pneumonia (PCP) in 23 (8.5%). Sixteen (6%) patients started four‐drugs, 200 (73%) an INSTI‐based three‐drug regimen, 132 (66%) including DTG, 56 (21%) a PI‐based three‐drug regimen (Table 1). One hundred and eighty‐two (67%) patients changed their first‐line regimen: 109 (60%) for simplification, 32 (18%) for toxicity, four (2%) for drug‐drug interactions, 36 (20%) for other reasons. DTG was interrupted in 19/132 (14%) patients: in 11 (8%) for toxicity, of which four CNS adverse events. IRIS was reported in 13 (5%) AIDS‐presenters without differences between DTG‐based and other ARV regimens. Ten patients died: three for PCP, two for Kaposi's sarcoma, one for PML, one for non‐Hodgkin lymphoma and three for non‐AIDS related events (Table 2). The 12‐month estimated probability of first‐line interruption was 14% (95% CI 11 to 17) for DTG, 40% (36 to 44) for any drug in regimens without DTG and 44% (40 to 48) in DTG‐based regimen. During a median observation time of 64 weeks (IQR 23 to 96), VL <50 copies/mL was achieved in 95/132 (72%) patients on DTG‐based regimen and in 92/140 (66%) patients with other regimens. After DTG‐based regimen initiation the proportion of patients with VL <50 copies/mL was 36% (36/100) at one month, 81% (68/84) at six months, 81% (56/69) at 12 months versus 17% (18/107) at one month, 77% (76/98) at six months, 78% (75/96) at 12 months among those without DTG. Significant increases of CD4+ cells count and CD4+/CD8+ ratio from baseline were observed at every time point (*p* < 0.05 for all comparisons).

**Table:** Abstract P139 – Table 1. Baseline characteristics of patients

Overall (n = 272)	ART with DTG (n = 132)	ART without DTG (n = 140)
Age, years^a^	44 (41 to 52)	44 (37 to 52)	44 (35 to 56)
Female gender, n (%)	85/272 (31)	28/132 (21)	57/140 (41)
Caucasian ethnicity, n (%)	217/272 (80)	106/132 (80)	111/140 (79)
AIDS‐presenters, n (%)	120/272 (44)	58/132 (44)	62/140 (44)
Late‐presenters, n (%)	152/272 (56)	74/132 (56)	78/140 (56)
Risk factor, n (%)	Heterosexuals 147/272 (54)	Heterosexuals 56/132 (42)	Other/unknown 20/132 (16)
	MSM 59/272 (22)	MSM 50/132 (38)	MSM 9/140 (6)
	Injecting drug user 14/272 (5)	Injecting drug user 6/132 (4)	Injecting drug user 8/140 (6)
	Other/unknown 52/272 (19)	Other/unknown 20/132 (16)	Other/unknown 31/140 (24)
Baseline CD4 count, cells/µL^a^	114 (40 to 241)	93 (30 to 192)	154 (50 to 270)
Baseline CD4 < 200 cells/µL, n (%)	180/259 (66)	100/129 (77)	80/130 (61)
Viral subtype B, n (%)	89/126 (71)	45/62 (73)	44/64 (69)
HIV‐1 RNA log10 (copies/mL) (on 114 available)^a^	5.2 (4.8 to 5.7)	5.2 (4.8 to 5.8)	5.2 (4.8 to 5.7)
HIV‐RNA >100,000 (copies/mL), n (%)	201/250 (74)	84/124 (68)	117/126 (93)
HIV‐RNA >500,000 (copies/mL), n (%)	60/250 (22)	32/124 (26)	28/126 (22)

^a^values are expressed as n (%) except for median (IQR).

**Table:** Abstract P139 – Table 2. Diagnosed AIDS‐defining events, IRIS and deaths according to antiretroviral regimen

	Overall (n = 272) (%)	ART with DTG (n = 132) (%)	ART without DTG (n = 140) (%)
Pneumocystis jirovecii pneumonia	23 (8)	22 (17)	1 (1)
Pulmonary tuberculosis	22 (8)	5 (4)	17 (12)
Progressive multifocal leukoencephalopathy	18 (7)	3 (2)	15 (11)
Wasting syndrome	16 (6)	5 (4)	11 (8)
Candida oesophagitis	15 (5)	12 (9)	3 (2)
Cytomegalovirus symptomatic infection	12 (4)	12 (9)	0
Non‐Hodgkin lymphoma	12 (4)	1 (1)	11 (8)
Kaposi's sarcoma	11 (4)	7 (5)	4 (3)
Cerebral toxoplasmosis	9 (3)	6 (4)	3 (2)
Cryptosporidium infection	7 (3)	2 (1)	5 (3)
Cervical cancer	5 (2)	1 (1)	4 (3)
AIDS dementia complex	4 (2)	3 (2)	1 (1)
Disseminated herpetis virus infection	4 (2)	0	4 (3)
Disseminated cryptococcosis	3 (1)	1 (1)	2 (2)
Non‐tubercular mycobacteriosis	2 (1)	1 (1)	1 (1)
IRIS	13 (5)	5 (4)	8 (6)
Deaths	10 (4)	5 (4)	5 (4)

**Conclusions: **The results confirm the high potency, the good tolerability and safety of DTG in advanced naïve patients, with a low risk for IRIS.

## P140

### Malaria in HIV‐infected patients: a matched case‐control study in a non‐endemic setting

**E Lam^1^, S De Wit^2^, M Delforge^2^ and C Martin^2^**

^1^Internal Medicine, ULB, Brussels, Belgium. ^2^Infectious Diseases, CHU Saint‐Pierre, Brussels, Belgium

**Background: **The impact of HIV infection on malaria is unclear in malaria non‐endemic areas. In endemic territories, it has been reported to be a risk factor for acquisition of malaria higher morbidity and parasitaemia and malaria treatment failure. In the context of HIV‐infected patients having a better quality of life and travelling more, in particular as VFR (visiting friends and relatives) represent a large proportion of travelling HIV‐infected patients, it is important to assess the impact of HIV on imported malaria.

**Material and methods**

This unicentric retrospective case‐control study collected data on HIV‐infected patients with malaria defined as positive thick smears with *P. falciparum* identification on microscopy and matched them with two controls based on age, sex and ethnicity. Clinical and biological parameters were collected and compared and different severity scores were applied on cases and controls.

**Results: **We identified 47 cases and 94 controls. Malaria prophylaxis use and delay before medical contact did not differ between cases and controls. Comparing each of the World Health Organization (WHO) 2014 severity criteria, hyperparasitaemia above 10% (*p* = 0.006), icterus (*p* = 0.042), acute renal failure (*p* = 0.022) and bacteraemia (*p* = 0.014) were significantly more present in HIV‐infected patients than in controls, with a trend to more neuromalaria (12.8% vs. 6.4%). The severity of malaria (defined by WHO 2014) was inversely related to CD4 T‐cell count. HIV‐infected patients were hospitalised more frequently and for longer periods and tended to stay more in intensive care unit. Death rate was significantly higher in cases (6.4% vs. 0). De novo HIV diagnosis was obtained in 17% of cases during the malaria episode. These differences in occurrence of severe malaria disappeared when patients with CD4 T‐cells count >500/µL (n = 9) were compared to controls but small size of this subgroup does not allow to draw firm conclusion.

**Conclusion: **HIV infection has an impact on the imported malaria profile, as well as in endemic areas. It is unclear if well‐controlled HIV‐infected patients have a higher risk of developing severe malaria. HIV‐infected patients should be particularly targeted for pre‐travel advice and physicians should always perform HIV testing during a malaria episode.

## P141

### Concomitant syphilis infection in patients with diagnosed HIV/AIDS: a retrospective multicentre study

**F Sarigul^1^, M Sayan^2,3^, D Inan^4^, A Deveci^5^, N Ceran^6^, M Celen^7^, A Cagatay^8^, H Ozkan Ozdemir^9^, F Kuscu^10^, G Karagoz^11^, Y Heper^12^, O Karabay^13^, B Dokuzoguz^14^, S Kaya^15^, N Erben^16^, I Karaoglan^17^, G Munis Ersoz^18^, O Gunal^19^, C Hatipoglu^20^, S Sayın Kutlu^21^, A Akbulut^22^, R Saba^23^, A Sener^24^ and S Buyuktuna^25^**

^1^Infectious Disease and Clinical Microbiology, Health Sciences University, Antalya Education and Research Hospital, Antalya, Turkey. ^2^Clinical Laboratory, PCR Unit, Kocaeli University, Faculty of Medicine, Kocaeli, Turkey. ^3^Research Center of Experimental Health Sciences, Near East University, Nicosia, Northern Cyprus. ^4^Infectious Disease and Clinical Microbiology, Akdeniz University, Faculty of Medicine, Antalya, Turkey. ^5^Infectious Disease and Clinical Microbiology, Samsun 19 Mayıs University, Faculty of Medicine, Samsun, Turkey. ^6^Infectious Disease and Clinical Microbiology, Health Sciences University, Istanbul Haydarpasa Numune Education and Research Hospital, Istanbul, Turkey. ^7^Infectious Disease and Clinical Microbiology, Diyarbakir University, Faculty of Medicine, Diyarbakir, Turkey. ^8^Infectious Disease and Clinical Microbiology, Istanbul Unıversity, Faculty of Medicine, Istanbul, Turkey. ^9^Infectious Disease and Clinical Microbiology, Health Sciences University, Izmir, Education and Research Hospital, Izmir, Turkey. ^10^Infectious Disease and Clinical Microbiology, Cukurova University, Faculty of Medicine, Adana, Turkey. ^11^Infectious Disease and Clinical Microbiology, Health Sciences University, Istanbul Umraniye Education and Research Hospital, Istanbul, Turkey. ^12^Infectious Disease and Clinical Microbiology, Bursa University, Faculty of Medicine, Bursa, Turkey. ^13^Infectious Disease and Clinical Microbiology, Sakarya University, Faculty of Medicine, Sakarya, Turkey. ^14^Infectious Disease and Clinical Microbiology, Health Sciences University, Ankara Numune Education and Research Hospital, Ankara, Turkey. ^15^Infectious Disease and Clinical Microbiology, Karadeniz Technical University, Faculty of Medicine, Trabzon, Turkey. ^16^Infectious Disease and Clinical Microbiology, Eskisehir University, Faculty of Medicine, Eskisehir, Turkey. ^17^Infectious Disease and Clinical Microbiology, Gaziantep University, Faculty of Medicine, Gaziantep, Turkey. ^18^Infectious Disease and Clinical Microbiology, Mersin University, Faculty of Medicine, Mersin, Turkey. ^19^Infectious Disease and Clinical Microbiology, Health Sciences University, Samsun Education and Research Hospital, Samsun, Turkey. ^20^Infectious Disease and Clinical Microbiology, Health Sciences University, Ankara Education and Research Hospital, Ankara, Turkey. ^21^Infectious Disease and Clinical Microbiology, Pamukkale University, Faculty of Medicine, Denizli, Turkey. ^22^Infectious Disease and Clinical Microbiology, Elazıg University, Faculty of Medicine, Elazıg, Turkey. ^23^Infectious Disease and Clinical Microbiology, Private Medstar Antalya Hospital, Antalya, Turkey. ^24^Infectious Disease and Clinical Microbiology, Onsekiz Mart University, Faculty of Medicine, Canakkale, Turkey. ^25^Infectious Disease and Clinical Microbiology, Cumhuriyet University, Faculty of Medicine, Sıvas, Turkey

**Background:** *Treponema pallidum* and HIV are both sexually transmitted agents of these infectious diseases with epidemiological similarities, and therefore co‐infect the same host [1,2]. The current number of HIV‐infected cases in Turkey is increasing significantly. For this reason, we aimed to reveal the syphilis status in HIV‐1 positive cases.

**Materials and methods: **A retrospective descriptive case series study, patients (aged ≥18 years) were followed up by 24 clinics from 16 cities of Turkey between January 2010 and April 2018. Those clinics are from all seven regions of Turkey. We examined the sociodemographic, including age, gender, nationality, marital status, partner HIV status, education, number of sexual partners, probable transmission route of HIV and syphilis infections, years of HIV seropositivity, clinical stage of syphilis and HIV/AIDS, use of ART and laboratory parameters such as results of CD4 +  T lymphocyte count and HIV viral load, quantative venereal diseases laboratory (VDRL) and *T. Pallidum* haemagglutination assay (TPHA) results and neurosyphilis association.

**Results: **A total of 3641 patients were followed with HIV‐1 infection, and 291 (8%) patients were diagnosed with syphilis co‐infection during eight years (Table 1). Most patients were older than 25 years old (92%) including 96% were males, 74% were working, 23% were unemployed and 3% were students. Laboratory characteristics of patients with HIV/AIDS/syphilis is shown in Table 2. The sexual predilections consisted of heterosexual (46%), homosexual (23%), bisexual (19%) and no data (36%). The three highest prevalence of syphilis were in Marmara (33%), Mediterranean (26%), Black Sea Regions (18%) (Figure 1).

**Table:** Abstract P141 – Table 1. Demographic characteristics of patients with HIV/AIDS/syphilis and probable routes of transmission

Characteristic	Study group
Patient, HIV/AIDS/syphilis, n (%)	3641/291 (8)
Gender, n (%) Female, Male	13 (4), 278 (96)
Age, median years (range)	41 (18‐90)
Ethnicity, n (%) Turkish, Others^a^	279 (96), 12 (4)
Socio‐economic status, n (%) Employed, Unemployed, Student	214 (74), 67 (23), 10 (3)
Education, n (%) Illiterate, Primary School, Secondary School, High School, University	3 (1), 91 (31), 39 (13), 97 (33), 63 (22)
Marital status, n (%) Married, Single, Widowed	106 (36), 179 (62), 6 (2)
Partner HIV status, n (%) Negative, Positive, Unknown	28 (10), 250 (86), 13 (4)
Number of sexual partners, n (%) Single, Multiple, Unknown	28 (10), 250 (86), 13 (4)
Condom usage, n (%) Yes, No, Unknown	22 (8), 166 (57), 103 (35)

^a^other nationality; Macedonia (n = 2), Turkmenistan (n = 2), Thailand (n = 1), Germany (n = 1), South Africa (n = 1), Kenya (n = 1), Brazil (n = 1), Egypt (n = 1), Russian Federation (n = 1), Afghanistan (n = 1).

**Table:** Abstract P141 – Table 2. Laboratory characteristics of patients with HIV/AIDS/syphilis

Characteristic	Study group
Patient, HIV/AIDS/syphilis, n (%)	3641/291 (8)
Duration of HIV and syphilis diagnosis, n (%) At the same time, 1 to 2 years, 3 to 4 years, 4 years	211 (73), 26 (9), 25 (8), 29 (10)
Antiretroviral usage, n (%) Yes, No	50 (17), 241 (83)
AIDS, n (%) Yes, No	45 (15), 246 (85)
Basal HIV‐1 RNA, median copy/mL (range)	9.7 + E5 (58‐1.0 + E9)
Basal CD4 + T lymphocyte count, median cell/mm^3^ (range)	384 (5 to 1520)
Late presenter status, n (%) ≤350 cells/mm^3^, >350 cells/mm^3^	134 (46), 157 (54)
Serum VDRL, median titer (range)	1/64 (1/2‐1024)
Serum TPHA, median titer (range)	1/640 (1/40‐1/5240)
Syphilis stage, n (%) Primary, Secondary, Latent	30 (10), 65 (22), 196 (68)
Neurosyphilis, n (%) Yes, No	25 (9), 266 (91)

**P141 – Figure 1.** Distribution by region of HIV/syphilis patients.

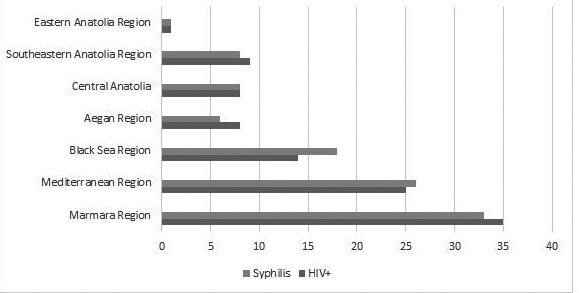

**Conclusion: **Turkey poses a risk in terms of geographical position about sexually transmitted diseases. On the other hand, Turkey is on international travel and trade route. Some countries with a high incidence of HIV/AIDS are also close. Additionally, syphilis and HIV/AIDS were closely related [3,4]. HIV/AIDS could increase the incidence of syphilis and vice versa, the increasing of syphilis could elevate HIV/AIDS.

**References**

[1] Braxton J, Davis D, Flagg E, Grey J, Grier L, et al. US Department of Health and Human Services Centers for Disease Control and Prevention, National Center for HIV/AIDS, Viral Hepatitis, STD, and TB Prevention, Division of STD Prevention [Internet]. Atlanta, Georgia, 30329‐4027 [cited 2017 Sep]. Available from: http://https://www.cdc.gov/std/stats.

[2] European Centre for Disease Prevention and Control. Syphilis. In: ECDC. Annual epidemiological report for 2016 [Internet]. Stockholm: ECDC; 2017 [cited 2018 Apr 19]. Available from: https://ecdc.europa.eu/en/annual‐epidemiological‐reports.

[3] European AIDS Clinical Society (EACS) guidelines for the clinical management and treatment of HIV‐infected adults [Internet]. 2017 [cited 2018 Apr 30]. Available from: http://www.eacsociety.org/files/guidelines_9.0‐english revised.

[4] Harnanti DV, Hidayati AN, Miftahussurur M. Concomitant sexually transmitted diseases in patients with diagnosed HIV/AIDS: a retrospective study. Afr J Infect Dis. 2018;12:83‐9.

## P142

### Overview of epidemiological, clinical and therapeutic features of visceral leishmaniasis/HIV co‐infection in Albania

**A Gjataj^1^, A Harxhi^2^, E Meta^3^ and D Kraja^2^**

^1^University Hospital Center of Mother Theresa, Department of Infectious Diseases Service, Tirana, Albania^2^University Hospital Center of Tirana, Faculty of Medicine, Infectious Disease, Tirana, Albania^3^University Hospital Center of Tirana, Infectious Disease, Tirana, Albania

**Background: **Cases of visceral leishmaniasis (VL) in the course of HIV have regularly been recorded especially in endemic areas such as the Mediterranean. It is a zoonotic infection developed in HIV patients who suffer from severe immunosuppression.

**Materials/methods**

This is a retrospective study which analyses epidemiological, clinical and therapeutic data of VL/HIV co‐infection from 2007 to 2017 in 12 HIV patients followed up at ambulatory HIV clinic at University Hospital Center of Tirana “Mother Theresa”.

**Results: **We describe the occurrence of this co‐infection in 12 patients from Albania. In total there were 66% males and 34% females and the mean age was 42 years old (range 25–50 years old). In half number of patients VL was diagnosed at the same time with HIV infection and only one case was reported as VL before HIV diagnosis. Mean CD4 at the first episode of VL was 14.5%‐135.6 cells/mm^3^. The main clinical signs and symptoms at admission were: fever (75%), sweat (83%), weight loss (91%). In objective examination we observed lymphadenopathy (83%), splenomegaly in all cases and hepatomegaly in 91% of them. Laboratoric findings were: anaemia in 83% of patients, leukopenia (91%), thrombocytopenia (66%) and increased levels of gamma globulinaemia in 75%. In most of cases the diagnosis of VL is based on bone marrow aspiration and in four cases it is isolated *Leishmania infantum*. Treatment of patients consisted in amfotericine B liposomale (58% of cases) and Glucantime in 41% of them. The outcomes of treatment resulted in a good clinical course and only three cases or 24% of patients developed relapses of VL even taking regularly antiretroviral therapy (the highest number of relapses was 5).

**Conclusions: **VL affecting HIV patients is considered a challenging condition because of its high levels of mortality and relapse rates. Even for middle income countries like Albania AmBisome should be regarded as the most effective first‐line therapy for co‐infected patients.

## P143

### Reduction of mortality rate among HIV/TB patients as a result of comprehensive approach of HIV/TB integration in the Kyiv Regional Tuberculosis Hospital, Ukraine

**A Chuykov^1^, Y Lopatina^2^, A Zakowicz^1^, G Tyapkin^2^, O Holub^3^ and O Shevchenko^3^**

^1^Europe Bureau, AIDS Healthcare Foundation, Amsterdam, Netherlands. ^2^Europe Bureau, AIDS Healthcare Foundation, Kiev, Ukraine. ^3^HIV/TB, Kiev Regional TB Hospital, Kiev, Ukraine

**Background: **In accordance with the World Health Organization and UNAIDS recommendations in the settings with high TB and HIV prevalence the HIV and TB services should be integrated. Studies also show that early ART initiation in TB/HIV co‐infected patients lowers mortality. We would like to present a model of full TB/HIV integration and estimate its effect on ART coverage, the time of ART initiation and mortality rate.

**Materials and methods: **We retrospectively reviewed TB registers and medical records of 988 TB/HIV co‐infected adults, registered for TB inpatient treatment at the Kyiv Regional Tuberculosis Hospital in the period between 2011 and 2017. The first HIV services were introduced into the TB Clinic in 2012; in 2012 AHF supported introduction of HIV rapid testing, PCP prophylaxis and HIV physician services at the clinic; in 2014 the clinic started provision of ART on its premises, OI diagnostic and treatment and CD4 POC (PIMA); in 2015 GeneXpert technology was implemented; in 2017 mental health component was added.

**Results: **The analysis included 988 patients. Baseline characteristics of patients have not changed in 2011 to 2017. Eighty‐six percent of patients had CD4 count less than 200 cells, 44% of patients had MRTB. Mortality rate decreased almost two times from 265.3 per 1000 patients in 2011 to 144.5 in 2017; coverage of ART increased from 43.5% in 2011 to 73.5% in 2017 and median time of ART initiation decreased almost five times from 170 days in 2011 to 26 days in 2017. Impact of the interventions is shown in Figure 1.

**Abstract P143 – Figure 1**. ARV coverage and impact of clinical interventions on mortality rate among HIV/TB co‐infected patients in Kyiv Regional Tuberculosis Hospital.

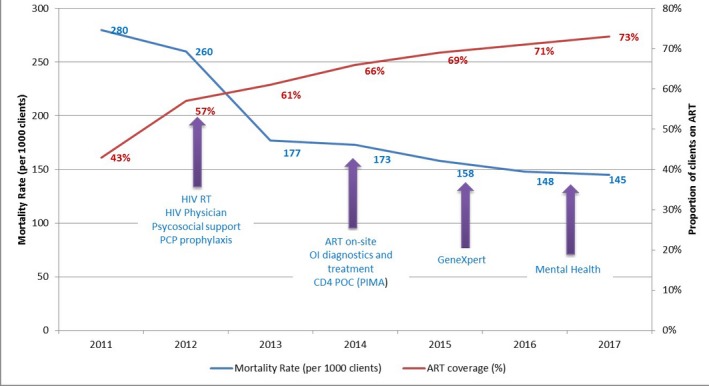

**Conclusions: **Full TB/HIV care integration in AHF‐supported clinic led to increased coverage of ART in 1.7 times, reduction of time of ART initiation by an average of 144 days and 1.8 times decreased mortality. The intervention, which resulted in patients increased access to testing and treatment, better patient management and earlier ARV initiation, has a potential to be successfully used as an effective HIV/TB service delivery model.

## P144

### Impact of geneXpert MTB/RIF for rapid tuberculosis diagnosis and rifampicin resistance detection among PLWHIV in South‐Western Nigeria

**S Usman**

Laboratory Services, APIN Public Health Initiatives, Abuja, Nigeria

**Background: **Xpert MTB/RIF is described as a game changer in tuberculosis control as tuberculosis due to *Mycobacterium tuberculosis* (MTB) still remains a major public health issue, with the disease estimated to cause approximately 9 million cases annually and 1.5 million deaths. This study thus aimed at assessing the impact of using Xpert for tuberculosis diagnosis and the efficiency in terms of tuberculosis treatment initiation in selected tuberculosis clinics in South‐Western Nigeria.

**Materials and methods: **A total of 300 consenting patients between 15 and 60 years of age attending tuberculosis clinic were recruited into this prospective cohort study from January 2015 to June 2017 and evaluated for pulmonary or extrapulmonary tuberculosis. Written consent was obtained from every participant and ethical clearance was obtained from the Ethical Review Committee of the Federal Teaching Hospital Ido Ekiti, Nigeria. Data were analysed using SPSS Statistics, with significance fixed at *p* < 0.05.

**Results: **The mean age ± SD is 37.11 ± 15.27 years. One hundred and thirty‐five (45.0%) of them are males while 165 (55.0%) are females. Two hundred and eighty‐four patients (94.7%) were diagnosed of pulmonary tuberculosis (PTB), with 282 (94.0%) being new tuberculosis patient. MTB detected; rifampicin (RIF) resistance not detected was resulted in 276 patients (92.0%) while MTB detected; RIF resistance detected was reported in two (0.7%) of the patients who tested smear microscopy positive at varying levels before treatment commencement. There was a significant association between site of infection and tuberculosis treatment outcome, as 233 (77.7%) of the patients were declared cured.

**Conclusion: **The Xpert MTB/RIF technique has great performance for rapid diagnosis of *Mycobacterium tuberculosis* with 93% sensitivity especially as it helps fast track tuberculosis treatment initiation; however, it cannot still be used as a standalone pulmonary tuberculosis diagnostic tool.

## **COMORBIDITIES AND COMPLICATIONS OF DISEASE AND/OR TREATMENT: AGEING**

## P145

### Phase IIIb, randomized, open‐label study to evaluate switching from a tenofovir disoproxil fumarate (TDF)‐containing regimen to elvitegravir/cobicistat/emtricitabine/tenofovir alafenamide (E/C/F/TAF) in virologically suppressed, HIV‐1 infected participants aged ≥60

**F Maggiolo^1^, G Rizzardini^2^, F Raffi^3^, F Pulido^4^, G Mateo Garcia^5^, J Molina^6^, E Ong^7^, Y Shao^8^, R Corales^9^, I McNicholl^10^, D Piontkowsky^10^, M Das^11^ and R Haubrich^10^**

^1^USC Malattie Infettive, Azienda Ospedaliera Papa Giovanni XXIII, Bergamo, Italy. ^2^Divisione Di Malattie Infettive E Servizio Di Allergologia, Via G. B. Grassi 74 I, Milan, Italy. ^3^Infectiologie, CHU Hotel, Nantes, France. ^4^Servicio Enfermedades Infecciosas, Hospital Universitario 12 de Octubre, Madrid, Spain. ^5^Internal Medicine, Hospital de Dia Infecciosas, Barcelona, Spain. ^6^Department of Infectious Diseases, Saint‐Louis Hospital and University of Paris, Paris, France. ^7^Department of Genitourinary Medicine, Royal Victoria Infirmary, Newcastle Upon Tyne, UK. ^8^Biometrics, Gilead Sciences, Foster City, CA, USA. ^9^Medical Sciences, Gilead Sciences, Foster City, CA, USA. ^10^Medical Affairs, Gilead Sciences, Foster City, CA, USA. ^11^Clinical Research, Gilead Sciences, Foster City, CA, USA

**Background: **With many people living with HIV who are now >50 years old, the long‐term safety in addition to efficacy of HIV treatment continues to be paramount. TAF is a tenofovir prodrug associated with 90% lower tenofovir plasma levels than TDF resulting in less renal and bone toxicity. We evaluated bone mineral density (BMD) changes after switching participants 60 years and older from a TDF‐ to a TAF‐containing regimen.

**Material and methods**

Virologically suppressed (HIV‐1 RNA <50 copies/mL) participants, age >60 years on a TDF‐containing regimen, were randomized (2:1) to open‐label E/C/F/TAF or continued TDF‐based regimen (TDF). The primary endpoint was the percent change from baseline to Week (W) 48 in spine and hip BMD. Differences in percentage changes from baseline in spine and hip BMD were analyzed using ANCOVA models with treatment as fixed effect and baseline BMD and sex as covariates. Secondary endpoints were HIV‐1 RNA <50 copies/mL at W24 and W48 (FDA Snapshot) and adverse events.

**Results: **Of 166 participants, characteristics were well balanced between E/C/F/TAF and TDF arms with a median age of 65 years (range 60 to 80), 11% female and 92% White. Baseline regimens consisted of two NRTIs combined with an NNRTI 78% (130/166), INSTI 12% (20/166) or boosted PI 9.6% (16/166). BMD (mean percent change from baseline) increased in E/C/F/TAF group and decreased in TDF group at both W24 and W48. At W48, spine BMD increased +2.2% and decreased −0.1% (*p* < 0.001 for between group comparisons) and hip BMD increased +1.3% and decreased −0.7% (*p* < 0.001) in the E/C/F/TAF and TDF groups, respectively (Figure 1). At W48, more patients who switched to E/C/F/TAF had normal hip T scores (>−1.0; at baseline 51% and at W48 58%), compared to continued TDF (at baseline 51% and at W48 46%). At W48, HIV RNA <50 copies/mL was maintained in 94% of participants in both arms. Confirmed HIV RNA >50 copies/mL was found in one participant in each arm; neither had drug resistance. There were no study drug‐related Grade 3 to 4 AEs. AEs leading to premature study drug discontinuation were similar: 3.6% with E/C/F/TAF and 1.8% with TDF.

**Abstract P145 – Figure 1.** Spine and hip BMD changes over 48 weeks. *p* values are comparison between randomized groups.

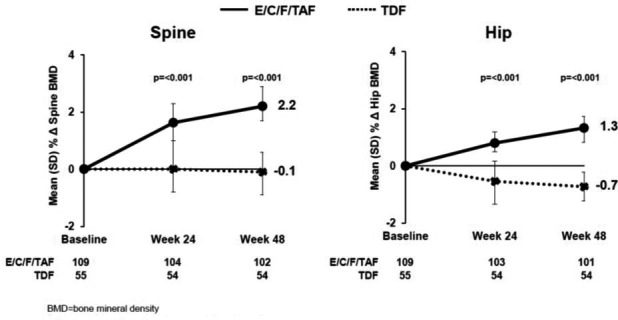

**Conclusion: **Through W48, spine and hip BMD significantly increased in older participants who switched to E/C/F/TAF compared to those who continued a TDF‐containing regimen. HIV‐1 RNA suppression was maintained through W48. The W48 BMD, safety and efficacy data support the switch to E/C/F/TAF in suppressed HIV‐infected participants aged ≥60 years.

## P146

### Effect of age on efficacy and safety of elvitegravir, cobicistat, emtricitabine and tenofovir alafenamide (E/C/F/TAF) in virologically‐suppressed, HIV‐1‐infected participants aged ≥65 years: pooled analysis of two Phase III trials

**F Maggiolo^1^, G Rizzardini^2^, F Raffi^3^, F Pulido^4^, G Mateo Garcia^5^, J Molina^6^, E Ong^7^, Y Shao^8^, S Chuck^9^, I McNicholl^9^, D Piontkowsky^9^, M Das^10^ and R Haubrich^9^**

^1^USC Malattie Infettive, Azienda Ospedaliera Papa Giovanni XXIII, Bergamo, Italy. ^2^Divisione Di Malattie Infettive, Ospedale Luigi Sacco, Milan, Italy. ^3^Infectiologie, CHU Hotel, Nantes, France. ^4^Servicio Enfermedades Infecciosas, Hospital Universitario 12 de Octubre, Madrid, Spain. ^5^Internal Medicine, Hospital de Dia Infecciosas, Barcelona, Spain. ^6^Department of Infectious Diseases, Saint‐Louis Hospital and University of Paris, Paris, France. ^7^Department of Genitourinary Medicine, Royal Victoria Infirmary, Newcastle Upon Tyne, UK. ^8^Biometrics, Gilead Sciences, Foster City, CA, USA. ^9^Medical Affairs, Gilead Sciences, Foster City, CA, USA. ^10^Clinical Research, Gilead Sciences, Foster City, CA, USA

**Background: **As the HIV population ages, analyzing safety and efficacy data for ARV agents in older adults living with HIV is increasingly important. TAF is a tenofovir prodrug associated with 90% lower tenofovir plasma levels and greater renal and bone safety than tenofovir disoproxil fumarate (TDF). We evaluated the efficacy and safety of E/C/F/TAF in individuals < and ≥65 years of age.

**Material and methods**

In two international, multicenter, Phase III trials, ARV‐experienced participants with HIV RNA <50 copies/mL were randomized 2:1 to receive: (1) E/C/F/TAF for 48 weeks or continued current abacavir/lamivudine (ABC/3TC)‐based regimen for 24 weeks followed by a delayed switch to E/C/F/TAF For another 24 weeks (292 to 1823); or (2) E/C/F/TAF or continued TDF‐based regimen for 48 weeks (292 to 1826, all subjects were ≥60 years). This pooled analysis of the E/C/F/TAF arms evaluated efficacy (HIV‐1 RNA <50 copies/mL, FDA Snapshot analysis) and safety through Week 48 for participants categorized by age (< and ≥65 years). Randomization was not stratified by age.

**Results: **A total of 293 participants were included in this analysis. Of the 74 participants ≥65 years, median age was 69 (range 65 to 80), 81% were male, 89% were White, median CD4 was 608 cells/mm^3^ compared to <65 years with a median age 51 years (range 25 to 64), 88% male, 85% White and median CD4 651 cells/mm^3^. Baseline regimens consisted of two NRTIs combined with an NNRTI 60% (175/293), INSTI 25% (73/293) or boosted PI 15% (45/293). At Week 48, HIV RNA <50 copies/mL was 89% in each age group. An HIV RNA ≥50 copies/mL was seen in 1 (0.5%) and 0 participants <65 and ≥65, respectively; no participant had virologic failure with resistance. Week 48 CD4 count was not significantly different between age groups. Adverse event (AE) profile was similar between both groups (Table 1). There were no discontinuations of E/C/F/TAF due to renal or bone AEs. Median change from baseline in eGFR was −3.0 mL/min in the <65 subgroup compared to −1.2 mL/min in the ≥65. Urine albumin:creatinine, urine beta‐2‐microglobulin:creatinine and urine retinol binding protein:creatinine ratios all improved more in the ≥65 than in younger participants.

**Table:** Abstract P146 – Table 1. Adverse events

Safety analysis population, % (n)	<65 years (n = 219)	≥65 years (n = 74)
Any Grade 2, 3 and 4 study drug‐related AEs	6.4 (14)	5.4 (4)
Any Grade 3‐4 study drug‐related AEs	0.5 (1)	1.4 (1)
Grade 3 or 4 lab AEs	17 (37)	9.5 (7)
Any study drug‐related serious AEs	0	0
AEs leading to study drug discontinuation	3.7 (8)	5.4 (4)^a^
Renal AEs leading to study drug discontinuation	0	0

^a^(1) constipation, arthralgia, myalgia; (2) diarrhea; (3) flatulence; (4) hepatocellular injury.

**Conclusion: **Through Week 48, rates of virologic suppression were high and similar between participants <65 and ≥65 years. AE, medication‐related discontinuation and tolerability were not significantly different between groups. Improved renal biomarkers were noted in those ≥65. The Week 48 efficacy and safety data support the switch to E/C/F/TAF in HIV‐infected, treatment‐experienced, HIV‐1 RNA suppressed people ≥65 years old.

## P147

### HIV comorbidities and their impact on attendance frequency at HIV clinics in the UK: findings from the Positive Voices survey 2017

**P Kirwan, M Kall, C Chau, A Brown and V Delpech**

HIV & STI Department, Public Health England, London, UK

**Background: **As people with HIV live longer due to effective treatment, increasing numbers of patients may experience comorbidity, which could impact HIV service use. Using national cohort and patient survey data we examine the impact of comorbidity on HIV service attendance patterns.

**Methods: **Positive Voices is a cross‐sectional survey of 4422 people attending 73 HIV clinics in England and Wales, recruited January to September 2017. Comorbidities were self‐reported as ever diagnosed from a list of 24 conditions. Survey responses were linked to data on adults attending specialist HIV clinics in England in 2017. Those newly diagnosed with HIV or starting ART within the previous 12 months were excluded (n = 33). Consultation frequency was compared in patients with/without selected comorbidities by multivariable linear regression. Suppressed viral load (VL) was defined as VL ≤200 copies/mL.

**Results: **HIV clinic attendance information was available for 87% (3861/4422) of respondents. Seventy‐two percent were men, 39% of non‐white ethnicity, the median age was 49 [IQR 41 to 56] and there was a median of 11 [6 to 16] years since diagnosis. Where information was available, 97% (2658/2751) were on ART with suppressed VL. The total number of HIV clinic visits in 2017 was 12,602. Median number of attendances was 3 [2–4]; however, attendances were highly skewed with a third attending four or more times. Patients in the highest decile of HIV consultations attended a median of 7 [6–9] (range 6 to 27) times in a year and accounted for 25% of all HIV consultations. Seventy‐four percent self‐reported at least one comorbidity with 36% reporting three or more conditions. The most common conditions were depression (31%), high cholesterol (27%), anxiety (24%), high blood pressure (21%) and sleep disorder/insomnia (14%). In multivariable regression controlling for age, gender, ethnicity, VL suppression and HIV risk group, there was a significant positive association between consultation frequency and number of comorbidities reported by patients (β=0.16 [0.12 to 0.20], *p* < 0.001) (β refers to the change in consultations for a given characteristic, after controlling for other factors, i.e. β of 0.16 means 0.16 additional consultations). Dementia was most strongly associated with consultation frequency, followed by psychosis/schizophrenia, neuropathy/peripheral neuropathy, cancer and anxiety (Figure 1).

**Conclusions: **In this engaged and ageing cohort of people with HIV, almost three‐quarters report comorbidities. Our results suggest an association between comorbidity and increased clinic attendance. Monitoring the increasing burden of comorbidities is needed to plan the future of HIV services.

**Abstract P147 – Figure 1.** Results of multivariable regression model adjusted for age, gender, ethnicity and HIV risk group.

*including peripheral neuropathy. Results are given as regression coefficient (95% confidence interval boundaries) and *p* value. Regression coefficient (β) is the change in consultations for a given characteristic, after controlling for other factors, i.e. coefficient of 2.87 means 2.87 additional consultations per year.

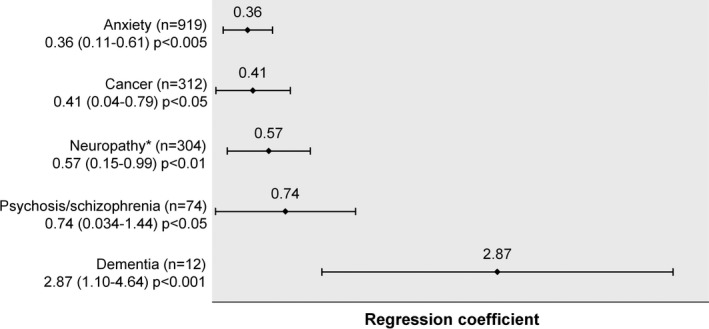

## P148

### Significant clinical and epidemiological differences in adults ageing with HIV‐1 compared to those with HIV‐1 diagnosis at older age

**O Tsachouridou^1^, M Papagiannis^1^, C Gogou^1^, T Chrysanthidis^1^, D Chatzidimitriou^2^, D Valagkouti^1^, P Kollaras^1^, P Zebekakis^1^, L Skoura^3^ and S Metallidis^1^**

^1^1st Internal Medicine, Infectious Diseases Unit, AHEPA University Hospital, Aristotle University of Thessaloniki, Thessaloniki, Greece. ^2^National AIDS Reference Centre of Northern Greece, Medical School, Aristotle University of Thessaloniki, Thessaloniki, Greece. ^3^Microbiology Department, AHEPA University Hospital, Aristotle University of Thessaloniki, Thessaloniki, Greece

**Background: **The widespread use of ART has markedly improved the survival of HIV‐1‐infected patients leading to an increase in the number of people ≥50 years living with HIV. Prolonged exposure to ART along with ageing may increase risk of developing comorbidities. In Greece there has been a recent increase in people who are diagnosed after 50 years of age. The purpose of this study was to describe the characteristics and comorbidity profile among people ageing with HIV versus those who were diagnosed at the age of ≥50 years.

**Materials and methods: **Retrospective study that enrolled HIV‐infected patients from January 1996 to June 2017 (single centre cohort in Northern Greece). Two groups were identified: patients aged less than 50 years at diagnosis who reached this age during the course of HIV infection and those aged ≥50 years at diagnosis. Baseline demographics, clinical characteristics and concomitant medications were recorded. Hospitalisations, related diagnoses and retention in care were also assessed.

**Results: **Six hundred and thirteen patients were enrolled (394: group diagnosis <50 and 219: group diagnosis >50). Most patients in both groups were males, but mode of transmission differed, with more patients reporting heterosexual contact as their only risk behaviour in the second group. Patients in the second group started ART significantly sooner. Additionally, patients diagnosed at older age were more frequently hospitalised, but had better retention in care. Furthermore, these adults were more likely to have hypertension, cardiovascular and renal disease; there was no difference with respect to bone disease. Hyperlipidaemia was more frequent in patients diagnosed at a younger age. Mortality rate was also significantly higher in the second group, while these patients were on one, two or three concomitant medications in addition to ART approximately twice as often compared to patients diagnosed at a younger age. Demographic and clinical data of both groups are summarised in Table 1.

**Table:** Abstract P148 – Table 1. Demographic and clinical data of patients enrolled

Diagnosis <50 y.o. (N = 394 pts)	Diagnosis ≥50 y.o. (N = 219 pts)	*p*
Mean age at study enrollment	39.4	58.7	0.003
SD±	7.0	6.8	
Sex			
Male	311 (78.9%)	189 (86.3%)	
Female	83 (21.0%)	30 (13.6%)	
Mode of transmission			
Heterosexual	113 (28.6%)	103 (47.0%)	
Homosexual	227 (57.6%)	68 (31.0%)	
PWID	25 (6.9%)	9 (4.1%)	
Other/Unknown	29 (7.3%)	39 (17.8%)	
ART			
Yes	287 (75.3%)	180 (82.1%)	
No	97 (24.6%)	38 (17.3%)	
Time till ART			
Months	31.4	10.4	0.005
SD±	45.0	18.2	
Hospitalization			
Yes	88 (22.4%)	108 (49.5%)	0.005
No	306 (77.6%)	111 (75.3%)	
Comorbidities			
CVD	11.9%	38.3%	0.004
Renal disease	7.6%	17.3%	0.002
Hypertension	12%	31.5%	0.01
Hyperlipidemia	35.8%	27.8%	
Bone disease	8.8%	9.1%	
Non‐AIDS‐malignancy	7.3%	15.9%	0.003
N of co‐medications			
1	127 (32.2%)	131 (59.8%)	0.002
2	97 (24.5%)	113 (51.4%)	
3	69 (17.4%)	86 (39.2%)	
Mortality rate (%)	15 (3.8%)	49 (22.3%)	0.001

CVD = cardiovascular disease; Renal disease = reduced level of glomerular filtration rate of proteinuria; Bone disease = osteopenia, osteoporosis or recorded bone fracture of any position.

**Conclusions: **There are significant differences between patients ageing with HIV and those diagnosed at an older age, leading to the need of individualising health care management of the elderly HIV population.

## P149

### High frequency of polypharmacy and drug‐drug interactions in an elderly HIV population on antiretroviral therapy

**D Bourneau‐Martin^1^, A Ruellan^1^, C Joyau^2^, S Sécher^3^ and C Allavena^4^**

^1^Regional Centre of Pharmacovigilance, CHU Angers, Angers, France. ^2^Regional Centre of Pharmacovigilance, CHU Nantes, Nantes, France. ^3^COREVIH Pays de la Loire, CHU Nantes, Nantes, France. ^4^Infectiology, CHU Nantes, Nantes, France

**Background: **Comorbidities and polypharmacy are frequent in the ageing population and have been associated with increased risk of adverse drug reactions (ADRs) and drug‐drug interactions (DDIs). Few data are available on DDIs between ARVs and comedications in an elderly HIV population.

**Materials and methods: **All prescribed treatments (ARVs and comedications) of HIV‐infected subjects aged 65 years and older followed in five French HIV centres were collected during an HIV routine visit. Three expert databases, Summary of Product Characteristics (SPC), national DDI Thesaurus and Liverpool HIV DDI website were used to define each DDI and specific grade. Relevant DDIs were defined as DDI mentioned in SPC and/or Thesaurus whatever the grade and/or moderate/high quality of evidence of interaction in Liverpool website. The objective was to describe all relevant DDIs between ARVs and comedications.

**Results: **From January to March 2017, prescriptions of 280 subjects (median age 70 years [interquartile range (IQR) 68 to 74], male 77.1%), receiving a median of 3 ARVs [3 to 3] and 4 comedications [1 to 7] were analysed; 41 prescriptions (14.6%) were not analysed, since no ARVs or no comedications were reported. Among the 239 subjects with potential DDI, 129 DDIs in 61 patients (25.5%) were identified (1 DDI n = 26, 2 DDIs n = 20, >3 DDIs n = 15). These 61 patients were treated with a median of 7 comedications [3 to 9] and 3 ARVs [3 to 4] and 80% (49/61) had at least one comorbidity, of whom most frequent were hypertension (33.6%), cardiovascular disease (28.9%) and dyslipidaemia (19.3%). Most frequent suspected ARVs were boosted protease inhibitors (PIs) (n = 81; 63%), non‐nucleoside reverse transcriptase inhibitors (n = 22; 17%), integrase inhibitor (n = 9; 7%) and cobicistat (n = 8; 6%). The major suspected comedications were statins (n = 41; 31.8%), calcic inhibitor (n = 14; 10.9%), antithrombotics (n = 12; 9.3%), alpha‐1 blocker (n = 8; 6.2%) and metformin (n = 7; 5.4%). Rosuvastatin and darunavir/ritonavir coprescription was the most frequently identified DDI (n = 11). Ten of 129 (7.8%) DDIs in seven subjects were contra‐indicated in at least one of the three expert databases: darunavir/ritonavir + alfuzosin in two subjects, darunavir/ritonavir + ticagrelor (n = 1), darunavir/ritonavir + amiodarone (n = 1), elvitegravir/cobicistat + budesonide (n = 1), nevirapine + ketoconazole (n = 1) and ritonavir + flecainide (n = 1).

**Conclusions: **At least one relevant DDI was evidenced in one out of four elderly patients receiving ARVs plus comedications. PIs and statins were the most frequent drugs involved in DDI. These results highlight the increased risk of polypharmacy and DDIs, the need to consistently collect comedications to pinpoint drug‐drug interactions and to strive for simplified regimens in the elderly HIV population.

## P150

### Prevalence of polypharmacy and potential drug‐drug interactions in PLWHIV: a cross‐sectional study from the Modena HIV Metabolic Clinic cohort

**A Raimondi, S Zona, I Franconi, F Carli, M Menozzi, G Nardini, B Beghetto, M Mancini, V Masi, C Mussini and G Guaraldi**

Clinic of Infectious Diseases, Policlinico di Modena, University of Modena and Reggio Emilia, Modena, Italy

**Introduction: **Comorbidities parallel the ageing epidemic affecting PLWHIV and implies an increasing number of comedications and polypharmacy (PP). PP increases the risk for potential drug‐drug interactions (DDIs), adverse drug reactions and grows pill burden. Our aim was to describe more frequently used comedications, assess PP prevalence and estimate the number of potential drug‐drug interactions in PLWHIV attending Modena HIV Metabolic Clinic (MHMC) in 2017. Secondary objective was to estimate independent predictors of potential DDIs.

**Materials and methods: **Cross‐sectional study targeting HIV+ patients on ART, attending MHMC in 2017. Comprehensive drug therapy was collected from patient charts using the fifth level of Anatomical Therapeutic Chemical (ATC) classification system and divided into categories. PP was defined in patients with ≥5 medications not including ART. Potential DDIs were assessed according to University of Liverpool HIV drug interactions database. For each category of drugs, the highest degree of interaction with every ART classes was reported as critical; the presence of minor interaction was reported as potential. Factors associated with DDIs were identified using univariate X2‐test for categorical variables and t‐test or Mann‐Whitney U test for normally or non‐normally distributed continuous variables, respectively.

**Results: **One thousand eight hundred and thirty‐four patients were included, 1349 (73.56%) were men, mean age was 53 (SD 8.3), 408 (22.5%) had PP of concomitant medications and 819 (44.66%) had potential interactions with ongoing ART regimen, 166 of them with critical interaction. As shown in Figure 1, the most represented classes of concomitant medications were vitamins such as vitamin D supplementation (1415 patients, 77.15%), lipid lowering (640, 34.90%) and antihypertensive (630, 34.35%). The difference in use by age was statistically significant for all classes of drugs except steroids and drugs for thyroid dysfunction. Potential DDIs, as illustrated in Figure 2, were significantly associated with age, comorbidities, type of ART and CD4 +  nadir (all *p* < 0.001). No association was observed between DDIs and sex or current immunological situation. PP was more prevalent in older patients: only 2% of ≤40‐year‐old patients presented PP, 11% in 41 to 50 years, 24% in 51 to 60 years and 48% in >60‐year‐old patients (*p* < 0.001).

**Abstract P150 – Figure 1.** Concomitant medications by class.

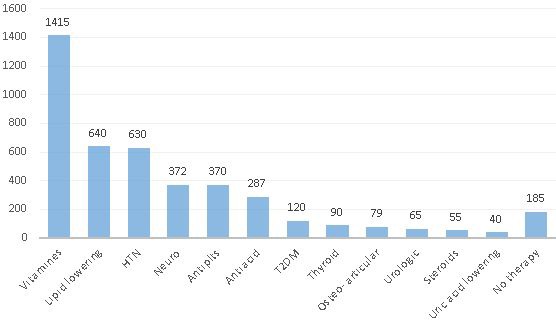

**Abstract P150 – Figure 2.** Sample characteristics by potential interactions (significant covariates).

*after Bonferroni adjustment.

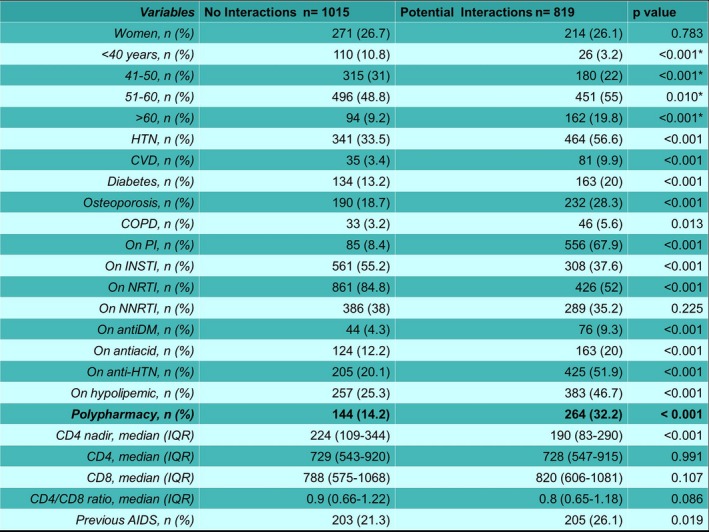

**Conclusion: **In a well‐described cohort of PLWHIV, we found a PP prevalence of 22.5%. Prevalence of PP increased in older subgroups reaching 48% in patients older than 60 years, as previously reported from other study groups. The high prevalence of PP resulted in a high prevalence of potential DDIs, especially in older patients.

## P151

### Comorbidities, comedication and polypharmacy burden in patients with HIV: retrospective claims data in Germany

**S Lopes^1^, K Meyer^2^, S Braun^3^, Y Punekar^1^, M Radford^4^ and J Haas^3^**

^1^Global Health Outcomes, Viiv Healthcare, London, UK. ^2^Global Health Economics and Outcomes Research, Xcenda LLC, Palm Harbor, FL, USA. ^3^Real World Evidence, Xcenda GmbH, Hannover, Germany. ^4^Health Outcomes Europe, Viiv Healthcare, London, UK

**Background: **ART has increased life expectancy of PLWHIV, transforming HIV management into chronic care. In ageing PLWHIV, the prevalence of comorbidities is increasing. Data on the prevalence of comorbidities, related prescription patterns of comedication and polypharmacy burden in PLWHIV are scarce, and may be relevant when selecting optimal ART for PLWHIV. The objective of this study was to characterise the demographics, comorbidities, polypharmacy and prescription patterns of non‐ART comedications among adult patients in Germany with HIV who were treated with ART in 2016.

**Materials and methods: **Demographics, comorbidities and prescription patterns of non‐ART comedications were retrieved from the German InGef health insurance claims database during 2016, using a retrospective, cross‐sectional cohort study design. Adult patients were included if they had an ICD‐10 diagnosis code for HIV and were prescribed any ART in 2016. Patients were stratified by gender, age, comorbidities and time on ART. Comorbidities were defined by ICD‐10 codes, and comedications were identified by ATC codes.

**Results: **Two thousand six hundred and eighty patients were included in the study, 86% were male, with a mean age of 45.6 years, 34% were aged ≥50 years and 29% had been on ART ≥5 years. The mean number of non‐HIV comorbid conditions per patient was 10.7 (standard deviation [SD] ± 7.55) for the overall population, and 13.3 (SD ± 8.6) for the subgroup of patients aged ≥50 years. The most prevalent comorbidities in the overall population included upper respiratory disorders (33%), anxiety and non‐psychotic mental disorders (31%) and mood disorders (29%); for patients aged ≥50 years, these were hypertensive diseases (41%), metabolic disorders (40%) and other dorsopathies (35%). Results for comorbidities were consistent with results for prescription patterns of comedications; where, on average, each patient took 7.0 (SD ± 10.14) different non‐ART medications, and for patients aged ≥50 years, the mean number of non‐ART drugs per patient was 10.2 (SD ± 11.78). The most commonly prescribed drugs were antibacterials (85%), anti‐inflammatory drugs (33%) and analgesics (25%) for the overall population; for those aged ≥50 years, they were antibacterials (74%), antihypertensives (36%) and anti‐inflammatory drugs (36%).

**Conclusions: **Results suggest that comorbidities and polypharmacy in PLWHIV are highly prevalent. With such high prevalence, ART selection is critical to reduce risk of drug‐drug interactions. These are increasingly relevant factors not only for current HIV management and appropriate ART selection, but also to achieve continuous improvement in the health status and health outcomes for these patients, reducing the overall burden on healthcare systems.

## P152

### Social determinants contribute to frailty in HIV‐positive patients in Bahia, Brazil

**D Zeballos Rivas^1^, W Howell and^2^C Brites Alves^1^**

^1^Postgraduate Program in Medicine and Health, Federal University of Bahia, Salvador, Brazil. ^2^School of Public Health, University of Michigan, Ann Arbor, MI, USA

**Background: **Frailty is a syndrome related to ageing that can develop earlier in the presence of HIV. Frail individuals are more vulnerable to adverse health outcomes. The magnitude of this problem in people living with HIV in Brazil is unknown. This research aimed to determine the prevalence and associated factors of frailty.

**Material and methods**

This was a cross‐sectional study conducted on 201 adults living with HIV, aged 50 years or older, receiving outpatient care between April and November 2017 at a referral centre in Salvador, Brazil. Demographic characteristics, HIV history, comorbidities and frailty markers were collected using a structured questionnaire and medical records. Frailty was defined according to Fried's frailty phenotype. Data were analysed using descriptive statistics. Differences and associations among groups were determined using t‐test, non‐parametric tests or *X* ^*2*^ test. Magnitude of associations was measured using prevalence ratio (PR).

**Results: **All but one patient were on ART. Mean age was 57.5 ± 6.7 years (range 50 to 83 years), 63.7% were male, 91% self‐identified as black or racially mixed, the median CD4 cell count was 669 (IQR 483 to 922) and 88.1% had undetectable viral load. Frailty and pre‐frailty prevalence was 18.9% (95% CI 14.1 to 24.9) and 49.7% (95% CI 42.9 to 56.6) respectively. When compared with robust individuals, frail individuals were more likely to be female (PR 2.24, 95% CI 1.34 to 3.75, *p* = 0.002), to have an individual income less than one minimum wage (PR 2.64, 95% CI 1.44 to 4.84, *p* = 0.001), to have less than 8 years of formal education (PR 4.93, 95% CI 1.30 to 18.78, *p* = 0.002) and to have three or more comorbidities (PR 2.08, 95% CI 1.11 to 3.90, *p* = 0.036). There was a significant difference in the mean of age at diagnosis between frail (45.5 ± 11.1 years) and robust (41.5 ± 8.4 years) groups, t(99)=2065, *p* = 0.041. Frail and pre‐frail patients had significantly lower CD4 nadir (median 114 vs 162, *p* = 0.033) and first CD4 cell count (median 179 vs 286, *p* = 0.018).

**Conclusions: **Frailty is common in elderly patients living with HIV. Half of the participants were pre‐frail, indicating a need for improved long‐term outpatient care. Being diagnosed at older ages and common variables associated with social vulnerabilities in Brazil are related to frailty in this patient population. Late presentation to care/treatment was strongly associated with frailty, indicating that early interventions can prevent its occurrence.

## P153

### Polypharmacy and polymorbidities in a specialised HIV clinic for patients over the age of 50: need for geriatrician intervention?

**K Khalid^1^, J Palmer^2^, B Pereira^1^, B Patterson^1^, A Milinkovic^1^, T Tong^3^, P Lee^3^, P Holmes^1^ and M Boffito^1^**

^1^Department of HIV/GUM, Chelsea and Westminster Hospital, London, UK. ^2^Faculty of Medicine, Imperial College, London, UK. ^3^Department of Care of the Elderly, Chelsea and Westminster Hospital, London, UK

**Background: **As PLWHIV live longer, the implications of ageing with HIV are becoming more apparent; >1/3 of PLWHIV accessing HIV care in the UK are now aged ≥50 years. Challenges specific to the management of older PLWHIV include polymorbidities, frailty, polypharmacy and drug‐drug interactions (DDI).

**Materials and methods: **We aimed at determining the prevalence of polymorbidities (≥2 comorbidities) and polypharmacy (≥5 medications) in PLWHIV attending the HIV over50 clinic during a 12‐month period (1 May 2017 to 30 April 2018). PLWHIV were stratified into three groups based on polypharmacy assessment: no polypharmacy (G1), polypharmacy (≥5 medications) (G2), heavy polypharmacy (≥10 medications) (G3). Potential DDI between antiretrovirals and non‐antiretrovirals were assessed using the University of Liverpool HIV Drug Interactions website. Frailty assessment was performed using the Rockwood Clinical Frailty Scale (RCFS) from 1 February 2018 to 30 April 2018. Sub‐analyses were conducted to assess changes in patients’ interaction profiles and frailty scales with increasing polypharmacy.

**Results: **Out of the 229 PLWHIV referred to the HIV over50 clinic, 120 were included in the analysis, the majority of them were Caucasian MSM. Polypharmacy and polymorbidities were recorded in >70%, e.g. hypertension in 52% and diabetes in 26%, with a significant correlation between being on polypharmacy and presenting with polymorbidities (*p* < 0.001). The proportion of significant DDI between antiretrovirals and non‐antiretrovirals significantly increased according to the extent of polypharmacy (G1 0.0% vs G2 11.1% vs G3 14.3%, *p* = 0.006). Twenty‐seven PLWHIV were screened for frailty and showed: a RCFS of 1 (48.1%), followed by scale 2 (25.9%) and scale 3 (22.2%). One patient had scale 4. No significant correlation was seen between polypharmacy assessment and frailty scales in this small sub‐group analysis.

**Conclusions: **Although we have only recently started to assess frailty in our HIV over50 service, we observed a high number of PLWHIV on polypharmacy and with polymorbidities, suggesting that the rate of medical problems/elderly syndromes (e.g. frailty, falls, etc.) is increasing as PLWHIV become older. Therefore we have set up an HIV/Care of the Elderly co‐specialty clinic to identify measures to prevent/reduce risk of progression to more advanced frail states.

## P154

### Experience of managing women aged 40 and over in a region with a moderate HIV prevalence

**D Ogbonmwan^1^, C Macfadyen^2^, B Ghavami‐Kia^3^, M Chauhan^1^, M Pinder^1^, A Wardropper^4^, C White^4^, S Ralph^5^, O Hotonu^6^, A Price^7^, J Hussey^8^ and S Duncan^9^**

^1^Genito‐Urinary Medicine, New Croft Centre, Newcastle Upon Tyne, UK. ^2^Medical School, Newcastle University, Newcastle Upon Tyne, UK. ^3^Microbiology, Royal Victoria Infirmary, Newcastle Upon Tyne, UK. ^4^Genito‐Urinary Medicine, University Hospital of North Durham, Durham, UK. ^5^Genito‐Urinary Medicine, Bishop Auckland Hospital, Bishop Auckland, UK. ^6^Genito‐Urinary Medicine, Morpeth Sexual Health Clinic, Morpeth, UK. ^7^Infectious Diseases, Royal Victoria Infirmary, Newcastle Upon Tyne, UK. ^8^Genito‐Urinary Medicine, Sunderland Royal Hospital, Sunderland, UK. ^9^Genito‐Urinary Medicine, Darlington Memorial Hospital, Darlington, UK

**Background: **An increasing number of women living with HIV (WLHIV) aged 40 and over access treatment and care in the United Kingdom (UK). Alongside tackling traditional comorbidities, the need to consider how best to manage the menopause is increasingly recognised as general practitioners have admitted to a lack of confidence when managing this cohort [1]. Some studies suggest that WLHIV encounter an earlier menopause [2] and therefore are at an increased risk of cardiovascular disease, dyslipidaemia and reduced bone health at a younger chronological age, alongside an increase in the severity of menopausal symptoms. There is a lack of data to inform clinical guidelines on assessing and managing this population. 

**Materials and methods: **We carried out a retrospective case note review of women aged 40 and over accessing HIV care in seven geographically diverse sites across our region. Patients were identified via the HIV and AIDS Reporting System and the data analysed using Excel. A focus group of women also completed a qualitative questionnaire regarding their experience of care.

**Results: **One hundred and twenty‐four case notes were studied; the mean age was 48 and 40% were aged 50 and over. In total, 25% of women were post‐menopausal, and 5% were perimenopausal. Over 92% of those that were menopausal were aged 51 and over, the average age of the menopause in the UK. Only 12% of peri‐ and post‐menopausal women reported using hormone replacement therapy. Screening for cardiovascular disease occurred in 71% with an average QRISK2 score of 3.3%. Only 15% of women aged 50 and over were documented as having had a mammography and only 14% had their FRAX score calculated. Twenty‐nine percent of those with psychological issues were either peri‐ or post‐menopausal. During our focus group, WLHIV described a lack of support from general practitioners and a preference of HIV physicians managing their menopause.

**Conclusions: **A significant proportion of WLHIV aged 40 and over in our region are transitioning though the menopause; however, a relatively small number access hormone replacement therapy. That women would prefer to have their menopause managed within the HIV service emphasises the need to consider specific multidisciplinary clinics or increased shared care between primary and secondary care to optimise patient experience and clinical outcomes.

**References**

[1] Chirwa M, Ma R, Guallar C, Tariq S. Managing the menopause in women living with HIV: a survey of general practitioners and practice nurses. HIV Med. 2016;17 Suppl 1:4.

[2] Fan MD, Maslow BS, Santoro N, Schoenbaum E. HIV and the menopause. Menopause Int. 2008;14:163‐8.

## P155

### Use of integrase strand transfer inhibitors (INSTIs) in a cohort of HIV‐infected geriatric patients (GEPPO cohort)

**S Nozza^1^, S Calza^2^, G Guaraldi^3^, E Gervasi^4^, A Riva^4^, G De Socio^5^, S Piconi^6^, G Orofino^7^, A Castagna^8^, G Di Perri^9^, A Cattelan^10^, P Magro^11^, B Celesia^12^, A Calcagno^9^ and E Focà^11^**

^1^Infectious Diseases, San Raffaele Scientific Institute, Milan, Italy. ^2^Unit of Biostatistics and Biomathematics, University of Brescia, Brescia, Italy. ^3^Infectious Diseases, University of Modena and Reggio Emilia, Modena, Italy. ^4^3rd Division of Infectious Diseases, University of Milano, Ospedale L. Sacco, Milan, Italy. ^5^Infectious Diseases, Azienda Ospedaliero‐Universitaria di Perugia, Perugia, Italy. ^6^1st Division of Infectious Diseases, University of Milano, Ospedale L. Sacco, Milan, Italy. ^7^Unit of Infectious Diseases, ‘Divisione A’, Turin, Italy. ^8^San Raffaele Scientific Institute, Università Vita‐Salute, Milan, Italy. ^9^Infectious Diseases, University of Torino, Turin, Italy. ^10^Infectious Diseases, Azienda Ospedaliero‐Universitaria di Padova, Padova, Italy. ^11^Infectious Diseases, University of Brescia, Brescia, Italy. ^12^Infectious Diseases, University of Catania, ARNAS Garibaldi, Catania, Italy

**Background: **There are increasing concerns about long‐term toxicity of antiretroviral treatment in elder HIV population with a high rate of polypharmacy (PP) and multimorbidity (MM). The INSTI‐based regimens are an intriguing antiretroviral therapy in this setting. The aim of the study is to assess the use of INSTI‐based regimens in a cohort of HIV‐positive patients more than 65 years (GEPPO cohort) old and the INSTI prescription trends from 2015 to 2017.

**Materials and methods: **Multicentre, non‐interventional, observational, retrospective, single arm study of HIV geriatric patients who started, for the first time, INSTI‐based regimen, from August 2015 to November 2017. We enrolled patients in antiretroviral therapy and we considered the last registered HIV RNA. Patients’ characteristics were described by median (quartiles) or frequency (%). INSTI regimens were compared by Mann‐Whitney test or chi‐square test. MM was defined as the presence of at least three comorbidities; PP was defined as the use of at least five drugs.

**Results: **One thousand five hundred and twenty‐six HIV‐positive patients aged more than 65 years old were included in GEPPO cohort. In Table 1 we show demographic, immunovirological, comorbidities and concomitant drugs characteristics of patients treated with INSTI‐based regimen both in 2015 and 2017. Data are available for 241 patients in 2015 and 257 patients in 2017. Viral load was undetectable in 71.6% in 2015 versus 77.4% in 2017. In Figure 1 we show the total prescription of INSTIs. INSTI‐based regimens were more commonly used in 2017 (726/1526, 47.6%) versus in 2015 (247/1183, 20.9%), *p* < 0.001. Among the INSTI‐based regimens, we observed a substantial increase in DTG use (60.7% vs 3.6%, *p* < 0.001) and a decrease of RAL use (30.6% vs 78.9%, *p* < 0.001). There was no change in the use of INSTI‐based dual regimens (42.5% vs 40.5%, *p* = 0.63). In 2017 the most prescribed dual therapies were DTG plus either XTC (23.1%), NNRTI (22.1%) or protease inhibitors (PI) (21.1%).

**Table:** Abstract P155 – Table 1. Characteristics of patients treated with INSTI‐based regimen

	2015, N = 214	2017, N = 257
Variable		
Sex (F)	20%	33%
Age	70.5	71.7
BMI	24.3	24.9
Years of HIV infection	18.7	16.3
Years of ART	15.6	16
CD4 nadir (cells/mm^3^)	177	198
CD4 cells count (cells/mm^3^)	564	583
Age >75 years old	22.8%	28.9%
Hypertension	66.7%	69.2%
Diabetes mellitus type 2	37.2%	30.9%
Cardiovascular disease	24.5%	25.3%
Chronic kidney disease	32.4%	37.8%
Chronic obstructive pulmonary disease	11.4%	7.8%
Dyslipidaemia	69.3%	72.9%
Cancer	21.4%	19.5%
Cirrhosis	5.5%	7%
Multimorbidity	59.8%	66.9%
Polypharmacy	14.3%	31.7%
Most representative ARV regimens		
INSTI + 1 PI	29%	22%
INSTI + 1 NNRTI	19.2%	17.1%
INSTI + 1 NRTI	31.8%	31.9%

BMI = body mass index.

**Abstract P155 – Figure 1.** Prescriptions of INSTIs in GEPPO cohort.

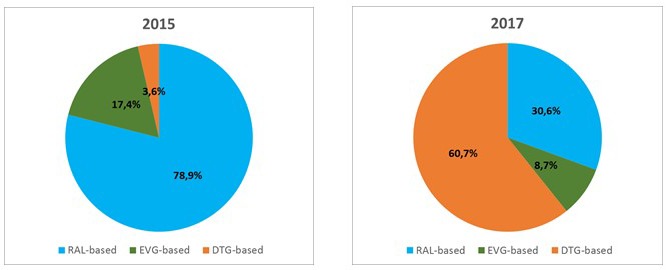

**Conclusions: **These data could be interpreted as a personalisation of ARV in a cohort of ageing patients with comorbidities and concomitant therapies. The newest dual regimens could reduce toxicity and spare drug options for the future. Dual therapy DTG based have high genetic barrier and could save options and toxicity in patients with long ARV experience, multimorbidities and polypharmacy.

## P156

### Disturbance in iron metabolism parameters: a possible marker of preterm ageing in HIV‐infected males

**G Dragovic^1^, B Dimitrijevic^1^, B Toljic^2^, J Milasin^2^, S De Luka^3^, D Jevtovic^4^ and A Trbovich^3^**

^1^Department of Clinical Pharmacology, Pharmacology, University of Belgrade, School of Medicine, Belgrade, Serbia. ^2^Department of Human Genetics, University of Belgrade, School of Dental Medicine, Belgrade, Serbia. ^3^Department of Pathological Physiology, University of Belgrade, School of Medicine, Belgrade, Serbia. ^4^HIV/AIDS Center/Infective/Tropical Disease Clinic, University of Belgrade, School of Medicine, Belgrade, Serbia

**Background: **Number of studies have documented age‐related iron accumulation in animal models. There are still not enough data in humans, especially in HIV‐infected patients. The aim of this study is to estimate levels of iron metabolism parameters and their correlation with multimorbidity occurrence in HIV‐infected ageing subjects.

**Materials and methods: **In this cross‐sectional study we included 50 non‐HIV infected blood donor volunteers and 50 HIV‐infected patients on cART: 2NRTIs+NNRTI or 2NRTIs+PI. Both groups of patients were at age of 50 years and older, all males, all Caucasians. In each group iron metabolism parameters: serum iron concentration, transferrin iron binding capacity (TIBC), transferrin saturation, serum transferrin and ferritin concentrations were determined. In both groups occurrence of multimorbidity was recorded. Multimorbidity was defined as the occurrence of two or more chronic conditions. Comparisons of the two cohorts were made using a chi‐square test or Fisher's exact test for categorical variables and using a Mann‐Whitney U test for continuous variables. All *p* values less than 0.05 were considered significant.

**Results: **HIV‐infected patients’ characteristics were: mean age was 59.6 ± 1.1 years, median CD4 +  T‐count 567.0 ± 317.7 cells/mm^3^, HIV RNA pVL was <50 copies/mL in all patients. All iron metabolism parameters, except transferrin levels, were significantly different between HIV‐infected and non‐HIV infected group. Mean plasma levels of serum iron (81.83 ± 29.03 vs 188.25 ± 119.99 mg/dL), TIBC (201.28 ± 82.51 µg/dL vs 309.28 ± 136.02 µg/dL) and the percentage of transferrin saturation (41.97 ± 12.37 vs 57.97 ± 14.14) were significantly lower (*p* < 0.001) in HIV‐infected versus non‐HIV group. Plasma transferrin levels were also lower in HIV‐infected group (36.70 ± 16.08 vs 44.66 ± 16.34 µmol/L), but with no significant difference (*p* < 0.061). In contrast with this plasma ferritin levels were significantly higher (386.94 ± 180.31 µg/L vs 143.57 ± 48.29 µg/L) in HIV‐infected versus non‐HIV infected (*p* < 0.001). Lower levels of serum iron, TIBC, transferrin and transferrin saturation together with higher ferritin levels significantly correlate with CD4 +  T‐count <350 cells/µL and with occurrence of multimorbidity (*p* = 0.001) in HIV‐infected group and in contrast to non‐HIV group.

**Conclusions: **Since lower levels of serum iron, TIBC, transferrin and transferrin saturation as well as with higher ferritin levels significantly correlate with multimorbidity occurrence in HIV/AIDS patients these parameters could possibly serve as surrogate markers of preterm ageing in HIV‐infected patients.

## P157

### Prevalence of cognitive impairment in a cohort of HIV‐infected patients older than 60 years

**A Gonzalez‐Baeza, G Rua‐Cebrian, R Montejano, J Bernardino, J Gonzalez and I Perez‐Valero**

HIV Unit, Hospital Universitario La Paz, IdiPAZ, Madrid, Spain

**Background: **Few studies have systematically evaluated the prevalence of neurocognitive impairment in samples of elderly HIV‐infected patients [1].

**Materials and methods: **We offered to participate in this study to all HIV patients older than 60 years, routinely attended in a HIV clinic in Madrid during 2016 to 2017. All patients underwent a comprehensive neuropsychological test battery: covering seven domains, measures of daily functionality (PAOFI) and screening for depression (HADS). Patients were classified as neurocognitively impaired (GDS>0.5), borderline neurocognitively impaired (0.3 > GDS<0.5) or neurocognitively intact (GDS<0.3) [2]. We calculated prevalence of neurocognitive impairment and we explored the association between cognitive status and sociodemographics and HIV‐related variables.

**Results: **All the participants (n = 100) were receiving effective antiretroviral therapy, were mostly men (84%), Caucasian (92%), middle educated (years of education: median [IQR] 13 [9.3 to 17]) and with good current immunological status (CD4 cell count: median [IQR] 703 [511 to 888]). Rates according to neurocognitive impairment status were: neurocognitively impaired (30%), borderline neurocognitively impaired (16%) and neurocognitively intact (54%). Half of the neurocognitively impaired patients reported interference in daily activities in the PAOFI scale and 16.7% of patients had a positive screening for depression. Table 1 shows comparisons among patients with different neurocognitive impairment status. After adjustments, only years of education was independently associated to NCI (OR 0.85 [CI 0.76 to 0.95], *p* = 0.004).

**Table:** Abstract P157 – Table 1. Comparisons among patients with different neurocognitive impairment status

	Neurocognitively intact (G1)	Borderline neurocognitively impaired (G2)	Neurocognitively impaired (G3)	Group comparisons (*p < 0.10; **p < 0.05)
Gender, N (%)	48 (88.9)	10 (62.5)	26 (86.7)	G1 > G2*; G2 < g3**< td=““></g3**<>
Age, X (SD)	65.2 (3.8)	66.7 (5.5)	68.2 (6.9)	G1 < g3**< td=““></g3**<>
Years of education, X (SD)	14 (4.3)	12.3 (5.2)	10.1 (4.5)	G1 > G3**
Years living with HIV, X (SD)	18.7 (9)	16.9 (10.6)	18.4 (7)	N/S
Years on ART, X (SD)	16.8 (7.2)	15.9 (8.2)	19.8 (19.4)	N/S
AIDS status, N (%)	19 (45.2)	9 (64.3)	12 (54.5)	N/S
IDU HIV transmission, N (%)	5 (9.3)	2 (12.5%)	4 (13.3)	N/S
Nadir CD4 < 200, N (%)	21 (45.7)	7 (58.3)	16 (66.7)	G1 < g3*< td=““></g3*<>
History of chronic HCV, N (%)	8 (15.4)	3 (20)	6 (21.4)	N/S
Positive depression screening, N (%)	6 (11.8)	0 (0)	5 (17.2)	G2 < g3**< td=““></g3**<>
PAOFI daily function alteration, N (%)	10 (19.6)	4 (26.7)	15 (50)	G1 < g3**< td=““></g3**<>

**Conclusions: **The prevalence of neurocognitive impairment in our cohort of HIV patients older than 60 years did not differ widely from rates we found in virologically suppressed patients of other age groups [3]. Years of education appear to protect of neurocognitive impairment in older HIV patients. We consider essential to conduct neurological and neurocognitive follow‐up, particularly in elderly patients neurocognitively impaired and those borderline neurocognitively impaired.

**References**

[1] Milanini B, Wendelken LA, Esmaeili‐Firidouni P, Chartier M, Crouch PC, Valcour V. The Montreal cognitive assessment to screen for cognitive impairment in HIV patients older than 60 years. J Acquir Immune Defic Syndr. 2014;67:67‐70.

[2] Carey CL, Woods SP, Gonzalez R, Conover E, Marcotte TD, Grant I, et al. Predictive validity of global deficit scores in detecting neuropsychological impairment in HIV infection. J Clin Exp Neuropsychol. 2004;26:307‐19.

[3] Pérez‐Valero I, González‐Baeza A, Estébanez M, Montes‐Ramírez ML, Bayón C, Pulido F, et al. Neurocognitive impairment in patients treated with protease inhibitor monotherapy or triple drug antiretroviral therapy. PLoS One. 2013;8:e69493.

## COMORBIDITIES AND COMPLICATIONS OF DISEASE AND/OR TREATMENT: BONE

## P158

### Incidence and risk factors associated with osteoporosis‐related fractures (ORF) among PLWHIV in British Columbia (BC), Canada

**J Barletta^1^, M Ye^2^, M Lu^2^, M Kibel^2^, R Hogg^2^, V Lima^2^ and S Guillemi^3^**

^1^Infectious Diseases, Hospital Juan A. Fernández, Buenos Aires, Argentina. ^2^Epidemiology and Population Health, British Columbia Centre for Excellence in HIV/AIDS, Vancouver, Canada. ^3^Clinical Education and Training Program, British Columbia Centre for Excellence in HIV/AIDS, Vancouver, Canada

**Background: **Increased longevity of PLWHIV receiving ART is accompanied by higher prevalence of age‐related comorbidities including bone disease [1]. HIV‐related factors and antiretroviral drugs have been found to play a role in pathogenesis of bone disease in PLWHIV. We investigated incidence and risk factors associated with ORF among PLWHIV in BC.

**Materials and methods: **These analyses were based on the Comparative Outcomes and Service Utilization Trends  (COAST) cohort study, a population‐based retrospective cohort study examining health outcomes and service use of PLWHIV and a 10% random sample of HIV‐negative individuals (HIV‐) in BC [2]. We examined incidence of ORF among PLWHIV from 1996 to 2013 considering wrist, vertebrae, humerus and hip fractures [3]; using physician and hospital‐based administrative data and International Classification of Diseases 9/10. Age‐ and sex‐adjusted incidence rates were calculated using 2011 Canada population as reference. The effect of the variables on the risk of ORF was assessed by logistic generalised estimating equation (GEE) model. For our GEE model, only ORFs occurring after ART initiation were considered; sex, age at ART initiation, previous injuries (including motor vehicle collision, land transportation injuries, self‐harm and assault), injection drug use (IDU), ART initiation era and length of time (LOT) on antiretroviral drug classes were covariates.

**Results: **A total of 6846 PLWHIV and 514,619 HIV‐ were included in the incidence analysis. ORF occurred in 416 PLWHIV and 28,028 HIV‐ (6.08% vs 5.45%, *p* = 0.02). PLWHIV were younger at first ORF (median age 47 years Q1 to Q3 38 to 53 vs 62 years Q1 to Q3 43 to 79, *p* < 0.0001). Age‐/sex‐adjusted ORF incidence rate was higher among PLWHIV (14.85 vs 6.43 per 1000 person‐years, rate ratio 2.31, 95% CI 1.97 to 2.65). In a multivariate analysis, female sex, older age at ART initiation, IDU, previous injuries and ART initiation before 2008 were associated with increased likelihood of ORF. Neither NNRTI or protease inhibitor (PI) exposure were associated with having an ORF in our population. Cumulative exposure to NRTIs was negatively associated with having an ORF (Table 1).

**Table:** Abstract P158 – Table 1. Multivariable factors associated with ORF among PLWHIV who initiated ART after 2001

OR [95% CI]
Female sex [ref: male]	1.49 [1.02 to 2.18]^b^
IDU [ref: no]	1.89 [1.26 to 2.84]^b^
Injuries before ORF [ref: no]	3.83 [2.60 to 5.64]^b^
Age at ART initiation (10 years)	1.63 [1.37 to 1.94]^b^
ART initiation era	
2001 to 2003 [ref]	1.00
2004 to 2007	0.57 [0.38 to 0.86]^b^
≥2008	0.14 [0.09 to 0.22]^b^
LOT on NRTI until ORF (1 year)^a^	0.75 [0.69 to 0.82]^b^
LOT on PI until ORF (1 year)^a^	1.07 [0.98 to 1.17]

^a^are time‐varying variables. ^b^are statistically significant (*p* < 0.05).

**Conclusions: **Higher rates of ORF were found among PLWHIV versus HIV‐. In our study, ORFs were associated with traditional risk factors in both populations. PLWHIV starting ART after 2008 and those with longer exposure to NRTIs were less likely to have an ORF.

**References**

[1] Moore AL, Vashisht A, Sabin CA, Mocroft A, Madge S, Phillips AN, et al. Reduced bone mineral density in HIV‐positive individuals. AIDS. 2001;15:1731‐3.

[2] Eyawo O, Hull MW, Salters K, Samji H, Cescon A, Sereda P, et al. Cohort profile: the Comparative Outcomes And Service Utilization Trends (COAST) study among people living with and without HIV in British Columbia, Canada. BMJ Open. 2018;8:e019115.

[3] Womack JA, Goulet JL, Gibert C, Brandt C, Chang CC, Gulanski B, et al. Increased risk of fragility fractures among HIV infected compared to uninfected male veterans. PLoS One. 2011;6:e17217.

## P159

### Association of lean mass with bone mineral density in young, recently diagnosed HIV‐infected patients

**J Gallego Galiana, M Vivancos, F Gioia, C Sobrino, M Pérez‐Elías, A Moreno, S Moreno, M Vázquez and J Casado**

Infectious Diseases, Hospital Universitario Ramón y Cajal, Madrid, Spain

**Background: **Low peak bone mass (PBM), the amount of bone at the end of skeletal maturation, is of paramount importance in HIV patients. However, the causes of a low PBM could be different in HIV‐infected patients, by considering time of HIV infection or use of combination antiretroviral therapy (cART).

**Method: **Cross‐sectional study of HIV‐infected patients younger than 40 years of age (EC 039/14; NCT02116751). A dual X‐ray absorptiometry (DXA) was perfomed at diagnosis or early after cART initiation. Bone mineral density (BMD) and Z and T‐scores were recorded for the lumbar spine (L1 to L4) and femoral neck. Demographic and HIV‐related factors were correlated with fat and lean mass.

**Results: **Overall, 151 patients were included. Mean age was 35.3 years, 85% were male, mean BMI was 24.2 kg/m^2^ (17.7 to 31.8), and 68% were MSM. CD4+ count nadir was 337/mm^3^ (196 to 430), and median time of HIV infection was 41.3 months. Only nine patients were HCV co‐infected (6%), and 27% had vitamin D levels (25OHD) below 25 ng/mL. A total of 52 patients (34%) had not initiated cART, whereas the remaining were receiving cART (no TDF in 29%, TDF plus non‐nucleoside in 58%) for 9 months before DXA evaluation. DXA scan showed a Z score <−2 in 2% in femoral neck (1% osteoporosis, 38% osteopenia) and 16% in spine (9% osteoporosis, 39% osteopenia). Total fat mass was 18.37 kgs (13.1 to 22.2), and total lean mass was 46.36 kgs (41.8 to 51.7). BMD at hip was significantly correlated with age (*r* = −0.21), BMI (*r* = 0.25), nadir CD4+ (*r* = 0.22) and with lean mass in all the body areas (lean mass to height squared ratio; *r* = 0.32; *p* < 0.01) but not with fat mass, whereas BMD in spine was correlated only with lean mass (*r* = 0.22), and not with age (*p* = 0.059), CD4+ nadir, BMI (*p* = 0.16), or rest of variables. Of note, in patients with a recent diagnosis who had not initiated cART, the strongest correlation was observed between BMD at hip and lean mass (*r* = 0.41; *p* < 0.01), with a trend for CD4+ count nadir (*r* = 0.28; *p* = 0.06).

**Conclusions: **In young, recently diagnosed HIV‐infected patients, lean mass is one of the most important factors determining the peak bone mass, more important than time of HIV infection or nadir CD4 +  count.

## P160

### The development of a tool for preventing and managing bone disease in HIV‐infected adults

**M Foisy^1^, C Hughes^2^, N Yuksel^2^**

^1^Northern Alberta Program, Royal Alexandra Hospital, Edmonton, Canada. ^2^Faculty of Pharmacy & Pharmaceutical Sciences, University of Alberta, Edmonton, Canada

**Background: **As the HIV‐infected population ages, numerous chronic comorbidities are emerging including bone disease. HIV clinicians are faced with managing a variety of conditions that require additional knowledge and skills. The objective is to describe the development and content of a practical tool to assist clinicians in preventing and managing bone disease in HIV‐infected adults.

**Materials and methods: **Development of the tool involved a group of three pharmacists with expertise in HIV and osteoporosis. The content was based on published literature, HIV conference abstracts, osteoporosis guidelines and expert consultation with an HIV endocrinologist. Input was also sought from the team dietitian. The tool was then reviewed for content, readability and applicability by several Canadian HIV/AIDS Pharmacists Network (CHAP) members. The final tool was published in the form of a compact 10‐page fold‐out pocket card. An electronic version of the tool was also posted on the CHAP website.

**Results: **The tool has four main sections: (1) Risk Factors for Fractures and Bone Loss: includes a concise table with key factors that predispose HIV+ patients to bone loss or fractures including significant patient characteristics, concurrent disease states, nutritional status, substance use, other medications and certain antiretrovirals associated with bone loss. (2) Patient Assessment: includes recommendations on initial screening, indications for bone mineral density testing, fracture risk assessment and diagnostic work‐up. (3) Treatment of Osteoporosis: outlines which patients are candidates for treatment of osteoporosis based on risk assessment and suggests treatment options, including drugs to treat osteoporosis, risk reduction strategies and monitoring of therapy. (4) Prevention: summarizes general prevention measures (i.e. non‐pharmacologic and lifestyle interventions) and pharmacologic measures (i.e. vitamin D and calcium supplementation). The pocket card has been distributed to the local HIV interdisciplinary team and primary care physicians involved in HIV care. The card was also distributed nationally to CHAP members for dissemination to their tertiary care and primary care team members. Through partnership with a national HIV drug information center, the cards are available nationally for order free of charge.

**Conclusions: **A clinical tool for the prevention and treatment of bone disease in HIV was developed by a group of expert pharmacists to provide practical guidance to clinicians and to standardize an approach to patient care. Further study is needed to evaluate the clinical utility of the tool and impact on prevention and detection of osteoporosis and disease management in HIV‐infected patients.

## P161

### Relationship between bone composition and fat indexes in HIV‐infected patients

**A Bonjoch^1^, N Perez‐Alvarez^1^, C Estany^1^, J Rosales^2^, P Echeverria^1^ and B Clotet^1^**

^1^Infectious Disease Service HIV Unit, Hospital Germans Trias i Pujol, Badalona, Spain. ^2^DIGEST, Center, Barcelona, Spain

**Background: **Body composition among the HIV‐infected people is influenced by several factors including antiretrovirals and the virus itself. This study aims to determine the relations between bone and fat distribution of this population.

**Method: **We collected T and Z scores and bone mineral density (BMD) from lumbar spine (L1 to L4) and femoral neck, and the overall BMD by dual energy X‐ray absorptiometry (DXA) from our cohort of HIV‐infected people. The following fat indexes were included: fat mass index (FMI), fat mass ratio (FMR), total and percentages of trunk fat/limb fat, of trunk fat/total fat, of limb fat/total fat and leg fat/body mass index (BMI). Linear regression analyses were performed.

**Results: **One thousand four hundred and eighty subjects were included, 75% were male and the median (IQR) of age was 45.6 (45 to 53) in men and 46.9 (41 to 52) in women. Significant results from the univariate model were incorporated to the multivariate analysis. Results are showed at Table 1. In summary, a positive relation were observed in both genders regarding total BMD and FMI (*p* < 0.001, *B* = +0.009 in women; *p* < 0.001, *B* = +0.015 in men) and a negative relation between total BMD and percentage of left leg fat/BMI (*p* < 0.001, *B* = −0.058 in women; *p* < 0.001, *B* = −0.097 in men). In women, L1 to L4 T score was related with % left leg fat/BMI (*p* = 0.001, *B* = −0.581); Z score with FMI (*p* = 0.014, *B* = −0.042) and limb fat/total fat index (*p* < 0.001, *B* = −2.342); and BMD in lumbar spine with % left leg fat/BMI (*p* = 0.001, *B* = −0.072). Femoral neck T score presented a relation with FMI (*p* < 0.001, *B* = +0.07) with % left leg fat/BMI (*p* = 0.008, *B* = −0.412), and BMD of femoral neck with FMI (*p* = 0.001, *B* = 0.007). For men, lumbar spine T score, Z score and lumbar BMD were related with % left leg fat/BMI (*p* = 0.001, *B* = −0.386; *p* = 0.001, *B* = −0.242; and *p* = 0.002, *B* = −0.044, respectively). The variable related with femoral neck T score was trunk fat/limb fat (*p* < 0.001, *B* = −0.203); Z score was related with % left leg fat/BMI (*p* < 0.001, *B* = −0.334), and BMD of femoral neck with trunk fat/total fat (*p* < 0.001, *B* = −0.115) and leg fat/total fat (*p* < 0.001, *B *= +0.159).

**Table:** Abstract P161 – Table 1. Multiple models for assessed bone measure by gender

B‐model coefficients (standard error)	*p*‐value	R^2^ or coefficient of determination
Women			
L1 to L4 T score			
% left leg fat/BMI	−0.581 (0.172)	0.001	0.031
L1 to L4 Z score			
FMI	−0.042 (0.017)	0.014	
Limb fat/total fat index (gr)	−2.342 (0.660)	0.001	0.051
L1 to L4 BMD			
% left leg fat/BMI	−0.072 (0.020)	0.001	0.035
Femoral neck T score			
FMI	0.07 (0.018)	0.001	
% left leg fat/BMI	−0.412 (0.155)	0.008	0.046
Femoral neck BMD			
FMI	0.007 (0.002)	0.001	0.028
DMO total			
FMI	0.009 (0.002)	0.001	
% left leg fat/BMI	−0.058 (0.014)	0.001	0.096
Men
L1 to L4 T score			
% left leg fat/BMI	−0.386 (0.120)	0.001	0.009
L1 to L4 Z score			
% left leg fat/BMI	−0.242 (0.087)	0.001	0.032
L1 to L4 BMD			
% left leg fat/BMI	−0.044 (0.014)	0.002	0.008
Femoral neck T score			
Trunk fat/limb fat (gr)	−0.203 (0.031)	0.001	0.038
Femoral neck Z score			
% left leg fat/BMI	−0.334 (0.088)	0.001	0.013
Femoral neck BMD			
Trunk fat/total fat (gr)	−0.115 (0.021)	0.001	
Leg fat/total fat (gr)	0.159 (0.034)	0.001	0.032
Total BMD			
FMI	0.015 (0.001)	0.001	
% left leg fat/BMI	−0.097 (0.012)	0.001	0.085

BMI = body mass index; FMI = fat mass index; BMD = bone mineral density. The indexes were expressed in grammes (gr) or percentage (%).

**Conclusions: **Percentage of left leg fat/BMI exhibit the most consistent correlation with bone indexes in both genders. The index that more positive correlation showed was FMI in women (more values in FMI corresponding to more values in the bone index). In men, negative correlation was observed in left leg fat/BMI index.

## P162

### Impact on bone mineral density after 2 years of switching to four dolutegravir‐based triple or dual regimens

**M Piscaglia^1^, I Gallazzi^1^, S Restelli^1^, G Baldin^2^, L Paladini^1^, M Cossu^1^, S Di Giambenedetto^2^, G Rizzardini^1^ and A Capetti^1^**

^1^1st Division of Infectious Diseases, ASST Fatebenefratelli Sacco, Milano, Italy. ^2^2nd Division of Infectious Diseases, Policlinico Universitario Agostino Gemelli, Roma, Italy

**Background: **Data on the impact of dolutegravir (DTG)‐based regimens on bone mineral density (BMD) are limited to the substudy of the SWORD trials [1], on the association of DTG with rilpivirine (RPV) and on a Spanish observation on the switch from PIs [2]. Having retrospectively analysed all the patients who consecutively took at least once DTG from November 2014 to April 2017 (DOLUTILITY study), we sought to describe such aspect as well.

**Materials and methods: **Of the 1039 subjects of the DOLUTILITY study, we selected those who had had a DEXA scan within 6 months prior to switching to DTG and a control 96 weeks ±4 weeks later with the same Hologic^TM^ machine and operator. The DTG plus lamivudine (3TC) cohort, absent in our substudy, comes from a twin cohort from Rome within the ODOACRE study group, of which the DOLUTILITY study is part. Wilcoxon test was used for repeated analyses, and Mann‐Whitney test for non‐parametric analyses.

**Result: **Only 83 subjects fitted the above specified criteria, and were divided by treatment regimen: 22 patients were on abacavir (ABC)/3TC/DTG, 21 on 3TC plus DTG, 20 on DTG plus boosted darunavir (bDRV) and 20 on DTG plus tenofovir/emtricitabine (TFV/FTC; initial 9 to 15 months on tenofovir disoproxil [TDF] then switched to alafenamide [TAF] plus FTC). The four groups differed by time from known HIV‐1 infection, CDC stage C, time on ART, HCV co‐infection and past use of TDF, with DTG plus bDRV and DTG plus TFV/FTC being the most vulnerable groups. The ABC/3TC/DTG group showed significant improvement at both hip and spine levels, while subjects on DTG plus 3TC benefited only at the spine level, those on DTG plus bDRV beyond the BMD gain at the spine also showed some recovery at the hip level, and finally, despite having switched in the second half period to TAF, the DTG plus TFV/FTC group worsened at both levels. Data are presented in Table 1. The q‐fracture and Frax score slightly worsened in all, mainly as an effect of ageing and accumulating comorbidities. The analysis of concordance of radiometric parameters with plasma colecalcipherol levels failed to indicate a significant relationship.

**Table:** Abstract P162 – Table 1. Evolution of bone parameters between baseline and Week 96 by treatment group

ABC/3TC/DTG	Parameter	Switch	Week 96	Delta	95% CI	*p* value
Spine	T‐score	−1.03 (1.156)	−0.96 (1.133)	0.068	[−0.017 to 0.15]	NS
	Z‐score	−0.565 (1.568)	−0.464 (1.17)	0.102	[0.057‐0.154]	0.0001
	BMD	0.979 (0.125)	0.987 (0.116)	+0.73%	[−0.014 to 0.023]	NS
Hip	T‐score	−1.045 (0.960)	−0.925 (0.932)	0.121	[0.057 to 0.185]	<0.001
	Z‐score	−0.448 (0.955)	−0.363 (1.013)	0.085	[0.035 to 0.136]	0.002
	BMD	0.766 (0.120)	0.782 (0.121)	+2.04%	[0.009 to 0.022]	<0.0001
DTG + 3TC						
Spine	T‐score	−1.43 (1.398)	−1.145 (1.285)	0.285	[0.14 to 0.43]	<0.001
	Z‐score	−0.785 (1.302)	−0.49 (1.231)	0.295	[0.16 to 0.42]	<0.001
	BMD	0.925 (0.161)	0.955 (0.149)	+3.17%	[0.014 to 0.045]	<0.001
Hip	T‐score	−1.4 (1.157)	−1.31 (1.284)	0.09	[−0.12 to 0.3]	NS
	Z‐score	−0.525 (1.054)	−0.39 (1.111)	0.135	[−0.018 to 0.29]	NS
	BMD	0.743 (0.15)	0.763 (0.185)	+2.69%	[−0.02 to 0.06]	NS
DTG + bDRV						
Spine	T‐score	−1.558 (0.74)	−1.458 (0.678)	0.1	[0.057 to 0.15]	0.0002
	Z‐score	−1.495 (0.77)	−1.365 (0.744)	0.13	[0.08 to 0.175]	<0.0001
	BMD	0.948 (0.121)	0.958 (0.116)	+1.14%	[0.003 to 0.018]	<0.02
Hip	T‐score	−1.065 (0.933)	−0.988 (0.932)	0.078	[−0.012 to 0.166]	NS
	Z‐score	−0.958 (1.022)	−0.883 (0.95)	0.075	[−0.012 to 0.162]	NS
	BMD	0.878 (0.088)	0.889 (0.093)	+1.26%	[0.0009 to 0.21]	<0.05
DTG + TFV/FTC						
Spine	T‐score	−1.413 (0.654)	−1.459 (0.630)	−0.045	[−0.098 to 0.008]	NS
	Z‐score	−1.164 (0.570)	−1.203 (0.594)	−0.039	[−0.08 to 0.002]	NS
	BMD	0.953 (0.423)	0.937 (0.274)	−1.68%	[−0.04 to 0.004]	NS
Hip	T‐score	−1.317 (0.395)	−1.405 (0.263)	−0.088	[−0.13 to −0.05]	<0.001
	Z‐score	−0.642 (0.164)	−0.689 (0.160)	−0.047	[−0.07 to −0.02]	<0.002
	BMD	0.752 (0.139)	0.728 (0.168)	−1.69%	[−0.04 to −0.004]	<0.05

NS = non significant.

**Conclusion: **Triple and dual regimens based on DTG and excluding TDF lead to significant gains in BMD. Even the simplification of salvage regimens to DTG plus bDRV, though maintaining a PI, yielded some improvement at the spine and hip level.

**References**

[1] McComsey GA, Lupo S, Parks D, Poggio MC, De Wet J, Kahl LP, et al. Switch from tenofovir disoproxil fumarate combination to dolutegravir with rilpivirine improves parameters of bone health. AIDS. 2018;32:477‐85.

[2] Negredo E, Estrada V, Domingo P, Gutiérrez MD, Mateo GM, Puig J, et al. Switching from a ritonavir‐boosted PI to dolutegravir as an alternative strategy in virologically suppressed HIV‐infected individuals. J Antimicrob Chemother. 2017;72:844‐9.

## P163

### Performance of fracture risk assessment tools in HIV‐positive individuals aged ≥45 years who were receiving suppressive antiretroviral therapy

**M Tsai^1^, J Zhang^1^, P Wu^1^, W Liu^1^, C Yang^1^ and C Hung^1^**

^1^Department of Internal Medicine, Far Eastern Memorial Hospital, New Taipei City, Taiwan. ^2^Center of Infection Control, National Taiwan University Hospital, Taipei, Taiwan. ^3^Department of Internal Medicine, National Taiwan University Hospital, Taipei, Taiwan

**Background: **To guide management of patients with reduced bone mineral density (BMD), an age‐specific evaluation and management algorithm is suggested for HIV‐positive patients without major risk factors. Whether a combination of dual‐energy X‐ray absorptiometry (DXA) and FRAX may detect more individuals for therapeutic interventions remains unclear.

**Materials and methods: **HIV‐positive Taiwanese aged ≥45 years who received combination ART (cART) were recruited. Patients with pregnancy, malignancy, AIDS status, pre‐existing bone disease or immobilisation were excluded. Information on clinical and demographic characteristics, FRAX questionnaire, BMD and serum 25(OH) Vit D was obtained. The physical activity was estimated using the short form of the International Physical Activity Questionnaire (IPAQ), Taiwan version, which was categorised into three classes: low (<600 MET‐minutes/week), moderate (600 to 3000) and high (>3000). FRAX scores combined with BMD (FRAX/BMD) and without BMD (FRAX) were calculated. Subjects were separated on the basis of major risk factors for fragility fracture and age. Two groups were defined: one group of subjects who received identical treatment recommendations from FRAX and FRAX/BMD, and another group who received a different treatment recommendation when BMD was included in the FRAX calculation.

**Result: **Three hundred and fifty‐nine HIV‐positive patients were enrolled: 76 at high risk for fracture (with major risk factors for fragility fracture), 154 intermediate risk (FRAX >10% 10‐year risk of major osteoporotic fracture) and 129 low risk (FRAX ≤10%). The subjects with distinct risk had different results fulfilling treatment criteria: high‐risk, 14 (FRAX) versus 29 (FRAX/BMD) (difference 19.7%, 95% CI 5.8 to 33.7%), and intermediate‐risk, 16 (FRAX) versus 58 (FRAX/BMD) (difference 27.3%, 95% CI 18.2 to 36.3%), and low‐risk, 0 (FRAX) versus 16 (FRAX/BMD) (difference 12.4%, 95% CI 6.7 to 18.1%). Body mass index (BMI) <22 kg/m^2^ was significantly different between prediction groups (OR 2.73, 95% CI 1.64 to 4.55). Of patients at high risk, BMI and low physical activity were predictors. BMI and aged 60 years or over were predictors in patients with intermediate risk.

**Conclusions: **By incorporating DXA screening as a complementary approach, the FRAX may detect more candidates eligible for further investigations or therapeutic management. The need for DXA among individuals with BMI <22 kg/m^2^, those with major risk factors and low level of physical activity, or those aged >60 years but without major risk factors can be higher on the priority list for investigations or therapeutic management.

## P164

### Bone turnover markers evolution after treatment initiation with Atripla in comparison to Truvada raltegravir: another glimpse to osteoporosis in HIV

**Y Oster^1^, M Cohen^2^, R Dresner Pollak^3^ and H Elinav^1^**

^1^Clinical Microbiology and Infectious Diseases, Hadassah Medical Center, Hebrew University, Jerusalem, Israel. ^2^Jerusalem District, Clalit Health Services, Jerusalem, Israel. ^3^Endocrinology and Metabolism, Hadassah Medical Center, Hebrew University, Jerusalem, Israel

**Background: **Accelerated osteoporosis, one of the comorbidities related to HIV, is multifactorial and was shown to be drug related as well. Bone turnover markers as P1NP, CTX, osteocalcin and P1CP can potentially improve our understanding of mechanisms of osteoporosis in HIV. As therapies targeting factors involved in osteoporosis are evolving, identifying factors that can potentially be blocked might improve or prevent this condition in people living with HIV.

**Aims: **The aim of our study was to identify bone turnover markers that play a role in osteoporosis in people living with HIV, and to compare the dynamic of their levels after initiation of two different and commonly used antiretroviral regimens.

**Method: **Male patients that initiated treatment with Truvada+raltegravir or Truvada+efavirenz and maintained the same regimen for at least 12 months were included in the study. Control group included HIV patients that did not receive antiretroviral treatment. Fifteen patients were included in each group. Levels of P1NP, CTX were measured in frozen serum samples using standard immunoassays at treatment initiation, and 1, 6 and 12 months following treatment initiation of the two study groups, and at 0, 6 and 12 months for the control group.

**Results: **Mean age of patients was 42.2 years and similar in all groups. Mean CD4 counts were similar in the treatment groups (205, 216 cells/mL) but higher in the control group (516 cells/mL) As expected, patients in the two treatment regimen groups showed a decrease in HIV viral load and similar increase in CD4 levels, while the control group had stable parameters during the follow‐up period. Baseline levels of CTX and P1NP levels were similar in all groups. While both treatment groups had showed significant increase over time of both markers, these parameters were stable in the control group. Levels of P1NP were statistically higher in the 6‐ and 12‐month time points (*p* = 0.002, *p* = 0.004, respectively) while CTX levels were statistically higher only at the 6‐month time point and then plateaued (*p* = 0.039).

**Conclusions: **Bone loss after HAART initiation is likely due to a high bone turnover state as both CTX and P1NP increase in the first 6 to 12 months of treatment. As high bone turnover often leads to bone loss, therapy with bisphosphonates or anti RANKL antibody which reduce turnover should be considered in the first year of HAART, thus reducing bone loss at this vulnerable period and potentially improving long‐term bone density.

## P165

### Audit of use of IV bisphosphonates in people living with HIV

**S Shah^1^, M Bracchi^1^, W Hurt^1^, Q Zhang^1^ and A Milinkovic^1^**

^1^Department of HIV Medicine, Chelsea and Westminster Hospital NHS Foundation Trust, London, UK. ^2^School of Medicine, Imperial College, London, UK

**Introduction: **PLWHIV are at increased risk of osteopenia, osteoporosis and subsequent fragility fractures. National and international guidelines help recognise and manage those at greatest risk and control comparative trials had shown that use of IV bisphosphonate therapy is more effective than switching tenofovir disoproxil fumarate (TDF) at increasing BMD in HIV‐positive adults with low bone mass. Clear prescribing policy for use of IV bisphosphonate in the setting of HIV has not yet been established.

**Method: **Our audit consists of a retrospective data collection on PLWHIV receiving IV bisphosphonate therapy on the Gazzard Day Unit at Chelsea and Westminster Hospital. The search included all patients who had received at least one dose of IV ibandronic or zoledronic acid between January 2015 and December 2017.

**Result: **Ninety patients were identified, of which 71 (79%) patients were male, with an average age of 56 years. Only two patients had a FRAX score documented before starting IV bisphosphonate therapy; the majority of patients (88/90) had a pre‐treatment dual X‐ray absorptiometry (DXA) scan, of which 67 (74%) patients had confirmed osteoporosis. Twenty‐three patients (26%) were started on IV bisphosphonate therapy for other reasons (e.g. myeloma, vertebral wedge compression). Twenty‐five of 90 (28%) patients had regular DXA scans performed during treatment. At the time of auditing, 39 patients (43%) were still on antiretrovirals associated with an increased risk of osteoporosis, including combinations containing TDF (n = 19) (21%), or protease inhibitors (PIs) (n = 31) (34%), and combinations containing both TDF and boosted PIs (n = 6) (7%). At the time of the audit 60% of patients were actively receiving IV bisphosphonate therapy, with average treatment duration of 24 months. Fifteen (28%) patients had exceeded the recommended 5‐year duration of therapy. Renal function, calcium and vitamin D levels were regularly monitored during treatment for all patients on IV bisphosphonate therapy. Half of the patients have been given vitamin D supplementation; 17% have been prescribed both calcium and vitamin D. In our patient population no patients were found to have experienced adverse effects secondary to IV bisphosphonate therapy, such as osteonecrosis of the jaw or atypical bone fractures.

**Conclusions: **Our audit has shown that guidance is needed to ensure appropriate use of IV bisphosphonate in PLWHIV. As a result of our findings, in our centre a multidisciplinary approach was taken to implement strategies aimed at improving management and monitoring of IV bisphosphonate administration.

## COMORBIDITIES AND COMPLICATIONS OF DISEASE AND/OR TREATMENT: CARDIOVASCULAR

## P166

### Immune and inflammatory biomarkers in naïve HIV‐infected patients starting antiretroviral therapy: a prospective study

**M Saumoy^1^, S Di Yacovo^1^, J Sanchez‐Quesada^2^, D Sviridov^3^, R Vila^4^, B Garcia^1^, A Navarro^1^, A Vernet^5^, D Giralt^1^, J Ordoñez‐Llanos^2^ and D Podzamczer^1^**

^1^HIV and STD Unit, Infectious Disease Service, Hospital Universitari de Bellvitge, Hospitalet de Llobregat, Spain. ^2^Biochemistry and Molecular Biology Department, Biomedical Research Institute IIB Sant Pau, Barcelona, Spain. ^3^Laboratory of Lipoproteins and Atherosclerosis, Baker Heart and Diabetes Institute, Melbourne, Australia. ^4^Vascular Surgery, Hospital Universitari de Bellvitge, Hospitalet de Llobregat, Spain. ^5^Department of Mechanical Engineering, Universitat Rovira i Virgili, Tarragona, Spain

**Background: **Atherogenesis in HIV patients is multifactorial. Our aim was to assess the effect of HIV infection and ART on several proatherogenic biomarkers and its relationship with subclinical atherosclerosis in a cohort of HIV‐infected naive patients.

**Methods: **Multicentre prospective comparative study. Two groups of naive HIV patients (group A: CD4 > 500, not starting ART at baseline; group B: CD4 < 500, starting ART at baseline) were compared with healthy controls (HC), matched by age and sex. At baseline, Months 12 and 24, laboratory data and carotid echography were performed. Low density lipoprotein (LDL) particle phenotype was measured by gel electrophoresis; total lipoprotein‐associated phospholipase A2 (Lp‐PLA2) by 2‐thio‐PAF assay; interleukin‐6 (IL‐6), high sensitivity C‐reactive protein (hs‐CRP), sCD14, sCD163 and ADMA by ELISA.

**Results: **Eighty‐four participants: 62 HIV patients (group A, n = 31; group B, n = 31) and 22 HC. Median age was 37 (30 to 43) years, 81% men. At baseline HIV patients had higher plasma concentrations of hs‐CRP (*p *< 0.001), sCD14 (*p* = 0.001), sCD163 (*p *< 0.001) and LDL‐Lp‐PLA2 (*p* = 0.004) and a worse LDL particle phenotype (smaller LDL size; *p* = 0.011) compared to HC group. Moreover, group B had higher levels of sCD14 (*p* = 0.001), sCD163 (*p* = 0.032) and ADMA (*p* = 0.003) compared to group A. No differences in common carotid‐intima media thickness (cc‐IMT) were found. During follow‐up 11 participants in group A started ART (not included in following analyses). In group B, at Month 24, there was an improvement in LDL phenotype: an increase in LDL particle size (*p* = 0.015) and a decrease in the percentage of sd‐LDL particles (*p* = 0.032). In group B at Month 12, sCD14 (*p* = 0.001), sCD163 (*p *< 0.001) and ADMA (*p* = 0.001) decreased, whereas IL‐6 decreased at Month 24 (*p* = 0.096), achieving all biomarkers similar values to those observed in HC; only CRP (*p* = 0.037) remained higher in group B at Month 24 compared to HC. No biomarkers changes were observed in group A nor in HC. Only group A showed a significant increase in common c‐IMT at Month 24 (*p* = 0.006). sCD14 and sCD163 levels correlated with those of several lipid variables (TC, HDL‐c, LDL‐c) at baseline and at Month 24. Change in CD4 and HIV viral load correlated with IL‐6, hs‐CRP, sCD14 and c‐IMT.

**Conclusions: **In HIV‐infected naive patients, ART was associated with improvements in LDL particle phenotype and inflammatory/immune biomarkers, reaching values similar to those of the controls, and preventing c‐IMT increase. Biomarkers were associated with lipid disturbances.

## P167

### Incidence of dyslipidaemia and modification of atherosclerotic cardiovascular disease (ASCVD) risk in HIV‐infected patients who switch away from tenofovir disoproxil fumarate (TDF)‐based to TDF‐sparing regimens in the Icona Foundation Cohort

**S Cicalini^1^, P Lorenzini^1^, A Cozzi‐Lepri^2^, F Maggiolo^3^, N Gianotti^4^, S Rusconi^5^, G Lapadula^6^, O Cirioni^7^, A Castagna^4^, C Mussini^8^, S Lo Caputo^9^, A Antinori^1^, on behalf of Icona Foundation Study Group**

^1^Clinical Department, National Institute for Infectious Diseases “L. Spallanzani”, Rome, Italy. ^2^Institute of Global Health, University College of London, London, UK. ^3^Division of Infectious Diseases, ASST Papa Giovanni XXIII, Bergamo, Italy. ^4^Clinic of Infectious Diseases, San Raffaele Scientific Institute, Milan, Italy. ^5^3rd Division of Infectious Diseases, DIBIC Luigi Sacco, University of Milan, Milan, Italy. ^6^Clinic of Infectious Diseases, San Gerardo Hospital University of Milano‐Bicocca, Monza, Italy. ^7^Clinic of Infectious Diseases, Polytechnic University of Marche, Ospedali Riuniti, Ancona, Italy. ^8^Clinic of Infectious Diseases, University of Modena and Reggio Emilia, Policlinico Hospital, Modena, Italy. ^9^Clinic of Infectious Diseases, Policlinic of Bari, Bari, Italy

**Background: **Increasing values of total cholesterol (TC), high‐density lipoproteins (HDL), low‐density lipoproteins (LDL) and triglycerides (TG) were observed among HIV patients on combination ART (cART) switching away from TDF–based regimens in randomised trials. However, the impact of these changes in terms of modification of ASCVD risk or need for lipid‐lowering therapy has not been assessed. This study aimed to characterise changes in lipid profile and ASCVD risk after switching away from a TDF‐based to a TDF‐sparing cART in a real‐world setting.

**Materials and methods: **Patients who started TDF‐based cART from 2008 and switched to a TDF‐sparing regimen were selected. We analysed changes of TC, HDL, LDL, non‐HDL, TG in patients who discontinued TDF, in the period before [−12; 0] and after [+4; +12] TDF switch. Paired *t*‐test was used to compare two values before and before/after TDF switch and ANCOVA to test the effect on lipid variations of third drug combined with TDF and type of regimen started after. We calculated proportion of patients, not recommended before, to be treated according to strong/moderate level of recommendation of 2013 AACE guidelines after TDF stop. In a subgroup, 10‐year ASCVD risk was calculated by Framingham Global Score.

**Results: **Two thousand five hundred and forty‐three patients included. Data showed stability before and increase after TDF interruption. TC and non‐HDL increased of +19 and +14 mg/dL, respectively (Table 1). No difference in TC and non‐HDL was observed according to third drug combined with TDF at switching (NNRTI +18, PI/b +19, INSTI +17, p at ANCOVA =0.932). Receiving PI/b after TDF discontinuing was associated to increase in TC (+28 vs. +17, *p *< 0.01) and non‐HDL (+24 vs. +11, *p *< 0.01). Conversely, receiving INSTI after TDF switch predicted lower increase in TC (+14 vs. +21, *p* = 0.02) and in non‐HDL (+9 vs. +15, *p* = 0.03). No difference was observed according to backbone after TDF change (TAF +23, ABC +19, less drug regimen +19, *p* = 0.963). Over 201 subjects, last value of ASCVD risk during TDF was 6.7% and 7.5% after (*p *< 0.01). Twenty‐two of 201 (11.0%) passed from a risk low (<10%) to intermediate (10 to 20%) or high (>20%) or from intermediate to high after TDF stop. The percentage of patients who became eligible for statin within one year from TDF discontinuation was estimated as 3.8% (95% CI 3.1 to 4.6).

**Table:** Abstract P167 – Table 1. Mean values and differences between two values of lipids before and before/after TDF discontinuation

Biomarker	N	T0	T1	Difference	*p* value	N	T1	T2	Difference	*p* value
		Mean 1 (SD1)	Mean 2 (SD2)				Mean 1 (SD1)	Mean 2 (SD2)		
LDL	1019	112.7 (33.0)	110.6 (33.0)	−2.1	0.178	557	111.5 (56.8)	121.7 (37.1)	+10.2	<0.01
	1361	44.9 (13.2)	44.8 (13.7)	−0.1	0.105	713	43.7 (13.3)	48.6 (15.6)	+4.9	<0.01
TC	1691	179.2 (38.5)	178.3 (38.6)	−0.9	0.550	879	180.4 (40.3)	199.4 (42.8)	+19.0	<0.01
Non‐HDL	1358	134.7 (37.8)	134.3 (36.9)	−0.4	0.618	710	137.0 (38.0)	150.6 (41.8)	+13.6	<0.01
TG	1484	132.6 (96.8)	138.0 (231.9)	−5.4	0.365	866	146.5 (85.8)	155.7 (106.8)	+9.2	<0.01

T0 and T1 = pre TDF discontinuation; TD2 = post TDF discontinuation.

**Conclusion: **We found evidence for a significant increase in lipids following TDF discontinuation. This variation did not appear to have a major impact on the 10‐years estimated CVD risk.

## P168

### Serum trimethylamine‐N‐oxide concentrations are associated with cardiovascular risk factors and subclinical vascular damage in HIV‐positive patients

**C Montrucchio^1^, A De Nicolò^2^, G D'Ettorre^3^, V Vullo^3^, L Celani^3^, C Costa^1^, A Trentalange^1^, C Alcantarini^1^, V Avataneo^2^, M Tettoni^1^, A D'Avolio^2^, S Bonora^1^, G Di Perri^1^ and A Calcagno^1^**

^1^Medical Science, Ospedale Amedeo di Savoia Unit of Infectious Disease, Torino, Italy. ^2^Medical Science, Ospedale Amedeo di Savoia Laboratory of Clinical Pharmacology and Pharmacogenetics, Torino, Italy. ^3^Infectious Diseases and Public Health, Sapienza University of Rome, Rome, Italy

**Introduction: **All cardiovascular (CV) risk scores under predict the incidence of CV events in HIV+ subjects and there are no clinically validated markers to increase disease prediction [1]. Trimethylamine‐N‐oxide (TMAO) has been shown to promote atherosclerosis and higher levels have been associated with CV events [2,3]. Being a modifiable risk factor we aimed at assessing the association between serum TMAO concentrations and CV risk as well as carotid intima media thickness (cIMT).

**Methods: **A fasting blood sample was collected and TMAO measured through a LC‐MS method in consecutive HIV‐positive patients from two Italian hospitals (Rome and Turin). Anthropometric, clinical and biochemical data were recorded; 10‐year cardiovascular risk score was calculated according to the ASCVD algorithm (<7.5%, 7.5 to 20%, >20%). The cIMT was performed by the same operator at 1 cm from carotid bifurcation as the average of three measurements (abnormal >0.9 mm). Polypharmacy was defined as ≥5 drugs other than ARV treatment. Data are expressed as medians (interquartile ranges).

**Results: **One hundred and eighty patients were included: age, gender (% male) and ethnicity (% Caucasian) were 50 years (42.2 to 55.5), 80% and 92.7%, respectively. Current and nadir CD4+ cell count were 566/μL (389 to 756) and 201/uL (85 to 330); treatment duration was 9.6 years (3.6 to 16) with 159 patients (89.9%) showing HIV RNA <50 copies/mL. Patients’ CV risk scores fell in the low (37%), intermediate (15.1%) and high (47.9%) strata. TMAO levels were 165.8 ng/mL (103 to 281.8). Higher TMAO quartile included 44 (24.4%) patients with higher CV risk (77.1% vs. 22.9%, rho = 0.26, *p* = 0.001), abnormal IMT value and plaque evidence (80% vs. 20%, *p *< 0.0001) and polypharmacy (47% vs. 14.7%, *p* = 0.05). Conversely, lower TMAO concentrations were observed in smokers (70.5%, *p *= 0.004). (20.5% vs. 70.5%, *p* = 0.004, OR 2.4). No immunovirological or ARV therapy‐related characteristics seem to predict higher TMAO values.

**Conclusion and Discussion**

TMAO higher concentrations were observed in patients with higher CV risk score and subclinical vascular damage measured by abnormal IMT/plaque presence. HIV‐positive patients with higher TMAO concentrations may benefit from targeted nutritional interventions.

**References**

[1] Thompson‐Paul AM, Lichtenstein KA, Armon C, Palella FJ Jr, Skarbinski J, Chmiel JS, et al. Cardiovascular disease risk prediction in the HIV Outpatient Study. Clin Infect Dis. 2016;63:1508‐16.

[2] Srinivasa S, Fitch KV, Lo J, Kadar H, Knight R, Wong K, et al. Plaque burden in HIV‐infected patients is associated with serum intestinal microbiota‐generated trimethylamine. AIDS. 2015;29:443‐52.

[3] Dillon SM, Frank DN, Wilson CC. The gut microbiome and HIV‐1 pathogenesis: a two‐way street. AIDS. 2016;30:2737‐51.

## P169

### Association between osteogenesis and inflammation evaluated by 18F‐NaF and 18F‐FDG PET/CT in HIV‐infected patients

**G Guaraldi^1^, N Prandini^2^, F Esposito^1^, A Malagoli^1^, J Milic^1^, B Beghetto^1^, G Nardini^1^, E Roncaglia^1^ and P Raggi^3^**

^1^Modena HIV Metabolic Clinic, University of Modena and Reggio Emilia, Modena, Italy. ^2^Department of Nuclear Medicine, Policlinico di Modena, Modena, Italy. ^3^Division of Cardiology, University of Alberta, Edmonton, Canada

**Background: **Initiation and progression of atherosclerotic plaque is a dynamic and complex process involving various pathophysiological steps including inflammation and calcification. We aimed to analyse the association between inflammation and vascular calcification at different stages of atherosclerosis, as well as the interrelationship between these two processes during HIV disease progression.

**Material and methods**

Eighty‐two patients who underwent two coronary CT at least 2 years apart for evaluation of coronary artery calcium (CAC) progression were enrolled. Fifty patients were examined by whole‐body18F‐sodium fluoride (18F‐NaF) PET, and 32 with 18F‐FDG PET. Tracer uptake in various arterial segments was analysed both qualitatively and semi‐quantitatively by measuring the blood‐pool–corrected standardised uptake value (target‐to‐background ratio [TBR]) using 1.6 cut‐off value for both 18F‐NaF PET and 18F‐FDG PET. The Fisher exact test and the Spearman correlation coefficient were used for statistical correlation of tracer uptake with CAC = 0, progression and non‐progression of CAC.

**Results: ** In our cohort 18F‐NaF uptake was observed in 149 (49.66%) of 300 coronary artery sites, in 49 (98%) of the 50 study patients, and the mean TBR was 1.75 ± 0.62. 18F‐FDG uptake was observed in 31 sites of 192 (16.14%), in 17 (53.12%) of 32 study patients, and the mean TBR was 1.35 ± 0.36. FDG uptake was present in 17 patients (53.12%) without CAC and in 15 (46.88%) patients with CAC. NaF uptake was detected 92 times (61.74%) in areas without CC and 57 times (38.26%) in areas with CAC >0. Non‐calcified atherosclerotic lesions were observed at 109 of 173 (35.16%) CAC = 0 sites in 56 (68.29%) patients. In 18F‐NaF cohort 92 (89.32%) of the non‐calcified sites presented 18F‐NaF uptake in 46 patients (92%) whereas in 18F‐FDG cohort 15 (21.42%) of the non‐calcified sites presented. 18F‐FDG uptake in 10 (31.25%). FDG uptake was observed 10 (31.25%) in CAC non‐progressor and 22 (68.75%) times in CAC progressor patients whereas 18F‐NaF was observed 47 (31.54%) in CAC non‐progressor and 102 (68.46%) times in CAC progressor patients. Spearman correlation coefficient *rS* between CAC non‐progression and positive 18F‐NaF was −0.56 (*p* = 0.01, in 19 patients) while the correlation between CAC non‐progression and positive FDG was not evaluated (all CAC values were zero). Spearman correlation coefficient *rS* between CAC progression and positive 18F‐NaF was 0.20 (*p* = 0.33, in 30 patients) while CAC progression and positive FDG was −0.65 (*p* = 0.02, in 14 patients).

**Conclusion: ** PET/CT with 18F‐FDG and 18F‐NaF may allow evaluation of distinct pathophysiological processes in atherosclerotic lesions and might provide information on the complex interactions involved in formation and progression of atherosclerotic plaque.

## P170

### Comparison between cardiovascular disease risk scores and observed rates of cardiovascular disease in people living with HIV

**M Lee^1^, C Smith^2^, J Mok^1^, A Duncan^1^, R Kulasegaram^1^ and A Wierzbicki^3^**

^1^Harrison Wing Department, Guy's and St Thomas Hospital NHS Foundation Trust, London, UK. ^2^Institute of Epidemiology and Health Care, University College London, London, UK. ^3^Department of Chemical Pathology, Guy's and St Thomas Hospital NHS Foundation Trust, London, UK

**Background: **It is unclear which risk calculator best predicts cardiovascular disease (CVD) risk in HIV‐positive patients; current UK guidelines recommend QRISK2 [1] but this has never been validated in HIV‐positive populations. We assessed commonly used risk calculators against the D:A:D equation, previously validated in US, European and Australian HIV‐positive cohorts [2], and observed CVD events during follow‐up.

**Materials and methods: **A representative sample of 245 HIV‐positive patients attending a London hospital aged >40, without a previous history of CVD, had baseline 10‐year CVD risk scores calculated using QRISK3, QRISK2, Framingham CVD equation and D:A:D risk calculators. D:A:D was re‐scaled from five‐ to ten‐year risk, assuming same rates in both five‐year periods. Scores were classified into low (<10%), moderate (10 to <20%) and high risk (≥20%). CVD events (myocardial infarctions, cerebrovascular events, invasive cardiac procedures, cardiac‐related deaths) occurring between enrolment in 2014 to 31 May 2018 were recorded. Agreement between scores using Bland‐Altman analysis was performed.

**Results: **Differences between risk calculators increased with increasing risk scores. Against D:A:D, mean difference for QRISK2 was 2.55 (95% limits of agreement (LOA) ‐7.77, 12.87), QRISK3 2.88 (LOA −8.78, 14.53), Framingham 5.52 (LOA −3.64, 14.69). Correlation was highest between QRISK2 and QRISK3 (*r* = 0.97), then Framingham and D:A:D (*r* = 0.92), QRISK2 and D:A:D (*r* = 0.91), QRISK2 and Framingham (*r* = 0.85). Switching from QRISK2 to QRISK3, QRISK2 to D:A:D, number of low‐risk patients re‐classified were 3 (1.8%) and 2 (1.2%), but for QRISK2 to Framingham, D:A:D to Framingham were 47 (28.3%) and 64 (34.0%) respectively (Table 1). Following re‐classification, 1 (33.3%), 0 (0%), 9 (19.1%) and 11 (17.2%) patients would require antiretroviral changes for each system respectively. Nine CVD events occurred during the study period (914.9 person‐years over 4.5 years after losses to follow‐up). Rates of CVD events, recorded per 100 person‐years, were stratified by low, moderate and high‐risk classifications: QRISK2 0.65, 1.21, 2.27; Framingham 0.22, 1.50, 2.15; and D:A:D 0.85, 1.25, 1.96 respectively.

**Table:** Abstract P170 – Table 1. Comparison of CVD risk calculators stratified by risk categories

	QRISK3 <10%	10% ≤ QRISK3 < 20%	QRISK3 ≥20%	Correlation
QRISK2 <10%	163	3	0	r = 0.97
10% ≤ QRISK2 < 20%	5	32	8	
QRISK2 ≥20%	0	4	30	
	D:A:D <10%	10% ≤ D:A:D < 20%	D:A:D ≥20%	
QRISK2 <10%	164	2	0	r = 0.91
10% ≤ QRISK2 < 20%	22	23	0	
QRISK2 ≥20%	2	19	13	
	Framingham <10%	10% ≤ Framingham <20%	Framingham ≥20%	
QRISK2 <10%	119	46	1	r = 0.85
10% ≤ QRISK2 < 20%	5	22	18	
QRISK2 ≥20%	0	3	31	
	Framingham <10%	10% ≤ Framingham < 20%	Framingham ≥ 20%	
D:A:D <10%	124	59	5	r = 0.92
10% ≤ D:A:D < 20%	0	12	32	
D:A:D ≥20%	0	0	13	

**Conclusion: **All other CVD risk calculators overestimated CVD risk against D:A:D. Correlation between QRISK2 and QRISK3 was high. Switching between calculators resulted in minimal risk upgrades or treatment changes between QRISK2 to QRISK3 or D:A:D, but resulted in larger variations from QRISK2 or D:A:D to Framingham. Despite low rates of CVD events observed, these followed risk classifications closely. Larger cohort studies may further inform the influence of chronic HIV infection on CVD risk in the era of modern antiretroviral choices and comorbidities monitoring.

**References**

[1] Angus B, Brook G, Awosusi F, Barker G, Boffito M, Das S, et al. BHIVA guidelines for the routine investigation and monitoring of adult HIV‐1‐positive individuals [Internet]. 2016 [cited 2018 Jul 3]. Available from: http://www.bhiva.org/monitoring‐guidelines.aspx.

[2] Friis‐Moller N, Ryom L, Smith C, Weber R, Reiss P, Dabis F, et al. An updated prediction model of the global risk of cardiovascular disease in HIV‐positive persons: the Data‐collection on Adverse Effects of Anti‐HIV Drugs (D:A:D) study. Eur J Prev Cardiol. 2016;23:214‐23.

## P171

### Inflammatory biomarkers related with subclinical atherosclerosis in suppressed HIV‐infected patients

**M Saumoy^1^, J Sanchez‐Quesada^2^, S Di Yacovo^1^, E Ferrer^1^, A Imaz^1^, B Garcia^1^, D Giralt^1^, J Ordoñez‐Llanos^2^ and D Podzamczer^1^**

^1^HIV and STD Unit, Infectious Disease Service, Hospital Universitari de Bellvitge, Hospitalet de Llobregat, Spain. ^2^Biochemistry and Molecular Biology Department, Biomedical Research Institute IIB Sant Pau, Barcelona, Spain

**Background: **Inflammation and immune activation persist in HIV patients despite an optimal virological control and can accelerate atherosclerosis. The objective of the study was to assess associations of risk factors and inflammatory biomarkers with subclinical atherosclerosis (SA) in virologically suppressed HIV patients.

**Methodology: **Observational cross‐sectional cohort study. Participants were randomly selected from an HIV unit. Inclusion criteria: >18 years, without cardiovascular (CV) disease, receiving combined antiretroviral therapy (cART) and undetectable viral load in the last six months. Common carotid intima‐media thickness (cc‐IMT) and presence of plaque were assessed. SA defined: carotid plaque or cc‐IMT > percentile 75 (P75) of a reference population. Demographic, ART history, CV risk (CVR), lipid profile and plasma biomarkers (sCD163, sCD14, IL‐6, hs‐CRP, D‐dimer, sVCAM and lipoprotein‐phospholipase A2[Lp‐PLA2]) were evaluated. Multivariate logistic regression including all variables with *p* = 0.1 in univariate analyses was used to assess factors related with SA. Continuous variables are expressed as mean (SD).

**Results: **Four hundred and fifty patients were included: age 50.4 (10), men 81%, 78% ever smoker, 24% hypertension, 7% diabetes, 23% used lipid lowering drugs. Duration of cART 14.7 (6.6) years, CD4 cell count 693 cells/uL. Mean cc‐IMT was 0.63 (0.13) mm; 30.5% had cc‐IMT >P75 of a reference population; 34.1% had at least one carotid plaque (mainly in bulb). SA prevalence was 49.2%. An increased CD4 was associated with a lower sCD163 (*p *< 0.001), D‐dimer (*p *= 0.004) and sVCAM (*p *< 0.001). A weak association was found between cc‐IMT and IL‐6 (*r* = 0.109; *p *= 0.029) or D‐dimer (*r* = 0.106; *p* = 0.034). Carotid plaque was associated with higher levels of sCD163 (*p *< 0.001), IL‐6 (*p* = 0.038), D‐dimer (*p* = 0.001) and sVCAM (*p* = 0.003). SA was associated with higher levels of sCD163 (*p *< 0.001), IL‐6 (*p* = 0.043), D‐dimer (*p* = 0.036), sVCAM (*p* = 0.041) and Lp‐PLA2 (*p* = 0.027). In multivariate analyses, use of thymidine analogues (*p* = 0.032), ever smoker (*p* = 0.027), hypertension (*p* = 0.001), cholesterol non‐HDL (*p* = 0.001) and sCD163 (*p* = 0.028) were associated with SA.

**Conclusion: **We have found a high prevalence of SA associated with traditional CVR factors and HIV. sCD163 was associated with SA independently of traditional CVR factors. In our study, inflammatory biomarkers were more strongly associated with carotid plaque than with cc‐IMT. Strategies to decrease immune activation may be useful to prevent atherogenesis in HIV patients besides an optimal control of CVR factors.

## P172

### Suggested targets in modifiable cardiovascular risk factors are seldom attained in HIV‐positive patients

**C Montrucchio^1^, A Lazzaro^1^, N Forni^1^, V Pirriatore^1^, C Alcantarini^1^, F D'Ascenzo^2^, M Maremmani^2^, V Cusenza^2^, M Peyracchia^2^, M Tettoni^1^, W Grosso Marra^2^, S Bonora^1^, G Di Perri^1^ and A Calcagno^1^**

^1^Medical Science, Ospedale Amedeo di Savoia Unit of Infectious Disease, Torino, Italy. ^2^Medical Science, Ospedale Città della Scienza e della Salute Unit of Cardiology, Torino, Italy

**Introduction: **The European AIDS Clinical Society (EACS) guidelines [1] for treatment of HIV‐positive adults suggest the targets to pursue for preventing cardiovascular disease (CVD). Beginning with underlying modifiable risk factors, the preventive efforts include smoking cessation, systolic blood pressure (SBP) control, glucose and lipids lowering and acetylsalicylic acid (ASA) start in primary prevention in high‐risk individuals.

**Methods: **HIV‐positive patients with no previous CVD were included. Ten‐year CV risk (CVR) score was calculated according to the ASCVD algorithm (low [L] <7.5%, intermediate [I] 7.5 to 20, high [H] >20%). EACS targets were <140 mmHg for SBP, 6.5% HbA1C for glucose tolerance, <190 mg/dL and <115 mg/dL for total (TC) and LDL cholesterol (LDL‐C). The measurement of common carotid IMT performed at 1 cm from bifurcation as the average of three measurements (abnormal >0.9 mm). Data are expressed as medians (interquartile ranges).

**Results: **Five hundred and three patients were enrolled: age, gender (% male) and ethnicity (% Caucasian) were 49.7 years (42.4 to 57.4), 78.1% and 93.8%, respectively. Four hundred and sixty patients (91.4%) were on HAART, 388 patients (80.3%) HIV RNA. Current and nadir CD4+ cell count were 554/uL (374 to 718) and 198/uL (87 to 316.7); treatment duration was 9.8 years (3 to 16.3); in 10yy ASCVD risk strata were: L 58.5%, I 27.3% and H 14.3%. Two hundred and forty‐four (49.6%) patients were active smokers: y29 (15%) were classified as having a H CVR. Despite 156 patients (31.5%) were receiving lipid‐lowering treatment TC and LDL‐C targets were not attained in 128 (82%) and 118 (77.6%) [*p *< 0.0001]. This percentage was higher in patients in the high CVR strata (respectively 80% and 67%). Additionally, despite 145 patients (29.2%) were on antihypertensive therapy, 45 (44.1%) were not on target for SBP (44.4%, *p *< 0.0001) in particular in H risk strata (73.3%). Out of 39 patients (7.9%) treated with hypoglycaemic therapy, 21 (53.8%) were not attained HbA1C target, 50% in H risk group (*p *< 0.001). In H risk patients, 29.2% were on PI‐based, 21.5% abacavir‐containing regimen; 18.5% in prevention with ASA. Out of 325 IMT performed, abnormal values were recorded in 91 (28%) out of 325 patients: observed more frequently in patients in H risk strata (44.9% vs. 40.5% I vs. 14.5% L, *p *< 0.001).

**Conclusion and Discussion**

A large proportion of HIV‐positive patients did not attain the suggested targets in modifiable CVR factors. In particular, those who are classified as I and H risk (41.6%) did not reach adequate control of TC, LDL‐C and SBP despite the use of lipid‐lowering (82%) and/or antihypertensive (44.1%) comedications.

**Reference: **[1] European AIDS Clinical Society Guidelines v.9.0 October 2017.

## P173

### Circulating annexin V and annexin A1 plasma levels and cardiovascular risk score in HIV subjects

**C Ucciferri^1^, A Auricchio^1^, F Vignale^1^, E Costantini^2^, C D'Angelo^2^, m Reale^2^, J Vecchiet^1^ and K Falasca^1^**

^1^Department of Medicine and Science of Aging, Clinic of Infectious Diseases, Chieti, Italy. ^2^Dept of Medical, Oral & Biotechnological Sciences, Unit of Immunodiagnostic and Molecular Pathology, Chieti, Italy

**Background: **The vascular endothelium plays a pivotal role in the pathogenesis of atherosclerosis and its clinical manifestations of the cardiovascular disease (CVD), myocardial infarction, heart failure, stroke and peripheral artery disease. Experimental and clinical studies in general population suggest that endothelial dysfunction as an independent predictor of adverse events in CVD patients [1] can be assessed quantitatively by measurement of CD31+/annexin (Anx)V plasma levels [2]. Also the AnxA1 has consistently been found to play an inhibitory role in innate forms of inappropriate inflammation. HIV‐1 disease progression is paradoxically characterised by systemic chronic immune activation and gut mucosal immune dysfunction, which is not fully defined. AnxA1, an inflammation modulator, is a potential link between systemic inflammation and immune dysfunction during the simian immunodeficiency virus (SIV) infection [3]. The aim of this study was evaluated to correlation between AnxV and AnxA1 plasma levels and cardiovascular risk scores in patients with HIV infection and viro‐immunological stable.

**Methods: **We enrolled 74 HIV‐positive patients in cART at the Infectious Diseases Clinics of Chieti. Demographic and anamnestic data were collected, blood and immunological parameters were measured in addition to the cystatin C, PCR, microalbuminuria and AnxA1 and V1 were analysed (Table 1). Different CV risk scores by Framingham, ASCVD, DAD and PROCAM risk scores were calculated.

**Results: **We found levels of AnxA1 (15.04 ± 12.16 ng/mL) and AnxV with levels of 2.80 ± 2.00 ng/mL. We had a negative association between AnxV1 level and Framingham score (*r* = −0.22, *p* = 0.05), ASCVD (*r* = −0.23, *p* = 0.04), DAD score (*r* = −0.24, *p* = 0.03) and PROCAM score (*r* = −0.30, *p* = 0.008). Also the AnxA1 was associated to cardiovascular risk score, in fact we found a negative correlation between AnxA1 and Framingham score (*r* = −0.40, *p* = 0.001), ASCVD (*r* = −0.48, *p* = 0.001), DAD score (*r* = −0.39, *p* = 0.001) and PROCAM score (*r* = −0.49, *p* = 0.001). Therefore an association between AnxV and microalbuminuria (*r* = −0.26, *p* = 0.02), between AnxA1 and cystatin C (*r* = −0.41, *p* = 0.001) and microalbuminuria (*r* = −0.55, *p* = 0.001) was found.

**Conclusion: **Our work shows that exist a correlation between the inflammatory annexins and the results obtained from the cardiovascular risk scores HIV correlated. Indeed low levels annexins were significantly correlated with high risk CVD, highlighting how the inflammatory process participates in the pathogenesis of cardiovascular damage in the HIV‐positive population.

**Table:** Abstract P173 – Table 1. Metabolic parameters

	Mean	DS
Age (y)	49.18	10.73
Sex (M%)	58 (78.4)	
BMI	26.66	4.33
CD4 (cells/uL)	640.74	333.46
CD4/CD8 (cells/uL)	6.36	34.87
Cystatin C (mg/L)	1.03	0.22
Microalbuminuria (mg/L)	3.06	4.55
HOMA IR triglycerides (mg/dL)	2.45	2.30
Total cholesterol (mg/dL)	185.31	34.34
LDL (mg/dL)	107.84	29.90
HDL (mg/dL)	45.72	17.51
Triglycerides (mg/dL)	158.26	96.39
Framingham %	8.37	6.62
ASCVD score %	8.56	6.50
DAD score %	3.40	3.12
PROCAM %	8.07	7.06

**References**

[1] Heitzer T, Schlinzig T, Krohn K, Meinertz T, Munzel T. Endothelial dysfunction, oxidative stress, and risk of cardiovascular events in patients with coronary artery disease. Circulation 2001;104:2673.

[2] Werner N, Wassmann S, Ahlers P, Kosiol S, Nickenig G. Circulating CD31+/annexin V+ apoptotic microparticles correlate with coronary endothelial function in patients with coronary artery disease. Arterioscler Thromb Vasc Biol 2006;26:112‐6.

[3] Sinning JM, Losch J, Walenta K, Böhm M, Nickenig G, Werner N. Circulating CD311/annexin V1 microparticles correlate with cardiovascular outcomes. Eur Heart J. 2011;32:2034‐41.

## P174

### Prevalence of abnormal echocardiographic findings in Thai HIV‐infected and non‐infected ageing population after receiving therapy: ECHO THAI‐HAART study

**W Thimaporn^1^, P Chattranukulchai^1^, S Siwamogsatham^2^, S Satitthummanid^1^, A Sangarlangkarn^3^, S Boonyaratavej^1^, A Avihingsanon^3^, on behalf of the HIV‐NAT 207 team**

^1^Division of Cardiovascular/Internal Medicine, Chulalongkorn University, Faculty of Medicine, Bangkok, Thailand. ^2^Division of Ambulatory and Hospital Medicine, Department of Internal Medicine, Faculty of Medicine, Chulalongkorn University, Bangkok, Thailand. ^3^HIV Netherlands Australia Thailand (HIV‐NAT) Research Collaboration, Thai Red Cross AIDS Research Centre, Bangkok, Thailand

**Background: **In the highly active antiretroviral therapy era, cardiovascular disease is the leading problem in HIV‐infected individuals. Various abnormal echocardiographic findings including diastolic dysfunction have been reported [1,2]. Little echocardiographic data was known in Thai ageing HIV‐infected population.

**Objective: **To study the prevalence of echocardiographic abnormalities in asymptomatic, virally suppressed HIV‐infected Thai ageing individuals in comparison with comparable age and gender non HIV‐infected control.

**Materials and methods: **A cross‐sectional study with comparable control selection using quota sampling based on age and gender. Total of 398 participants without established cardiovascular disease (298, 75% HIV‐infected individuals) were enrolled and underwent standardised two‐dimensional transthoracic echocardiography and were interpreted by single experienced reader blinded to the study.

**Result: **In HIV‐infected patients, a median CD4 cell count was 614 cells/mm^3^, 97.35% were virally suppressed and a median 16.2 years of antiretroviral exposure. Of these, there were 1.1% with left ventricular systolic dysfunction, 22.4% with diastolic dysfunction and 3.2% with pulmonary hypertension which were not significantly different from non HIV‐infected patients (Figure 1). Age >60 years, BMI >23 kg/m^2^, high ASCVD risk, hypertension, diabetes, metabolic syndrome were associated with diastolic dysfunction while female, statin exposure and LAVI >34 mL/m^2^ were associated with pulmonary hypertension, *p *< 0.05.

**Conclusion: ** In this large echocardiographic study, the prevalence of asymptomatic left ventricular systolic dysfunction and pulmonary hypertension were low. In the new HAART era, the prevalence of structural cardiac abnormalities in HIV‐infected patients were not different from age‐ and gender‐based quota sampling control.

**Abstract P174 – Figure 1.** Percentage of abnormal echocardiographic findings between HIV infected and non‐HIV infected groups.

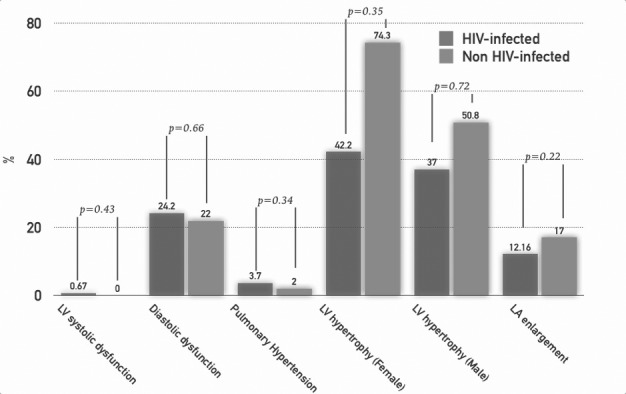

**References**

[1] Mondy KE, Gottdiener J, Overton ET, Henry K, Bush T, Conley L, et al. High prevalence of echocardiographic abnormalities among HIV‐infected persons in the era of highly active antiretroviral therapy. Clin Infect Dis. 2011;52:378‐86.

[2] Reinsch N, Neuhaus K, Esser S, Potthoff A, Hower M, Brockmeyer NH, et al. Prevalence of cardiac diastolic dysfunction in HIV‐infected patients: results of the HIV‐HEART study. HIV Clin Trials. 2010;11:156‐62.

## P175

### Estimated eGFR and risk of cardiovascular events in a large cohort of chronically infected HIV subjects

**F Maggiolo^1^, R Teocchi^2^, M Borderi^3^, E Di Filippo^1^, L Comi^1^, D Valenti^1^ and M Rizzi^1^**

^1^Infectious Diseases, ASST Papa Giovanni XXIII, Bergamo, Italy. ^2^Informatics, ASST Papa Giovanni XXIII, Bergamo, Italy. ^3^Infectious Diseases, S. Orsola Malpighi Hospital, University of Bologna, Bologna, Italy

**Background: **End‐stage renal disease substantially increases the risks of death and cardiovascular disease, but the effects of less severe kidney dysfunction on these outcomes in HIV‐infected subjects are less well defined.

**Methods: **A retrospective study was performed on the cohort of a large reference hospital in Northern Italy. eGFR was computed for HIV+ subjects followed in 2006 and the occurrence of cardiovascular diseases was checked in the following 10 years. Cardiovascular diseases was defined as any of the following: hospitalisation for coronary disease, heart failure, stroke or peripheral arterial disease and death because of cardiovascular reasons. Furthermore, the occurrence of hypertension and/or chronic kidney insufficiency was recorded. Logistic regression was used for the analysis.

**Results: **One thousand four hundred and eighty‐two subjects performed at least a visit in 2006; 12 (0.8%) died because of other causes, 42 (2.8%) were lost to follow‐up and 69 (4.6%) already presented a cardiovascular disease in 2006. All these patients were excluded from analysis. The remaining 1359 subjects were mostly males (72%) with a median age of 41.6 years (IQR 10.8) and a median time from first HIV diagnosis of 9.6 years (IQR 6.2). The proportion of patients with a HIV‐RNA value <50 copies/mL was 57.8% in 2006 and the proportion of severely immune‐depressed patients (CD4 <200 cells/mcL) was 9.3%. Median serum creatinine levels were 0.8 mg/dL (IQR 0.2) eGFR from 109.6 (IQR 15.4). The proportion of patients with altered eGFR (<60 mL/min/1.73 m^2^) was a modest 1.2%. Over 10 years 90 subjects (6.7%) presented a major cardiovascular event the proportion raised to 21.2% including hypertension, while renal insufficiency developed in 43 subjects (3.2%). Grouping subjects according to eGFR values 5th percentile (<80 mL/min/1.73 m^2^) 25th percentile (80 to 100 mL/min/1.73 m^2^) or above we observed a linear association with the risk of cardiovascular events lowering from 18.5% to 10.5% to 6.8% (*p* = 0.029). Including hypertension the same values were 40.7%, 28.9% and 19.7% (*p* = 0.003). The association was stronger when renal insufficiency was the endpoint: 26.9%, 11.4% and 3.8% (*p *< 0.0001). Finally, the eGFR decrement was faster and significantly greater during follow‐up in patients developing either cardiovascular events or renal insufficiency than in those without the comorbidity (Figure 1).

**Abstract P175 – Figure 1.** eGFR variation over 10 years.

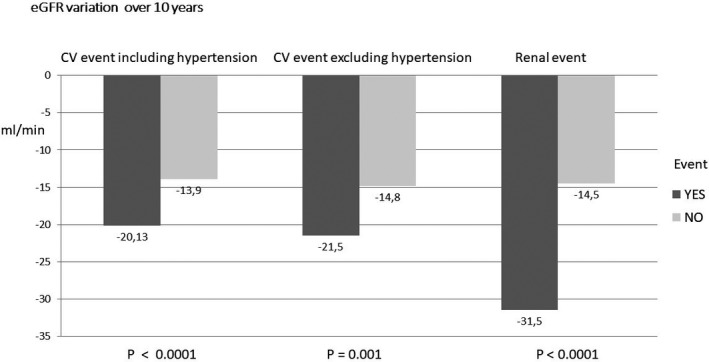

**Conclusion: **A relevant, graded association was observed between a reduced estimated GFR and the risk of death, cardiovascular events or renal insufficiency. These findings highlight the clinical importance of closely monitoring chronic renal insufficiency in the HIV‐infected population.

## P176

### Cardiovascular risk in HIV‐positive population: serological markers and cardiovascular risk calculators

**C Ucciferri^1^, A Auricchio^2^, F Vignale^1^, J Vecchiet^1^ and K Falasca^1^**

^1^Department of Medicine and Science of Aging, University “G. d'Annunzio”, Clinic of Infectious Diseases, Chieti, Italy. ^2^Department of Medicine and Health Sciences, University of Molise, Campobasso, Italy

**Background: **At to date cardiovascular disease is an important cause of death in the HIV‐positive population [1] and this phenomenon can be explained by the presence of an HIV‐related chronic inflammatory state. A lot of algorithms have been used to predict cardiovascular risk (CVR): Framingham Risk Score (FRS), Atherosclerotic Cardiovascular Disease (ASCVD), the Prospective Cardiovascular Münster study score (PROCAM) and the DAD Five Years Estimated Risk, but none of these considers the inflammatory state in the assessment [2–4]. The aim of the study is to show the relationship between plasma inflammatory markers and CVR scores.

**Materials and methods: **We enrolled 90 HIV‐positive patients in cART at the Infectious Diseases Clinics of Chieti. Demographic and anamnestic data were collected, blood and immunological parameters were measured in addition to the cystatin C, PCR, microalbuminuria, IL‐18, IL‐2, IL‐4, IL‐6, IL‐10, TNF‐α and IFN‐γ and CVR scores.

**Results: **Our population was made up 90 HIV‐positive patients: 70 males (77.8%) and 20 females (22.2%) with a mean age of 48.86 ± 10.01 years and a mean BMI of 25.97 ± 3.94 kg/m^2^. Biochemical data showed a mean of CD4+ lymphocytes of 686.09 ± 311.51 cells/mL, CD4/CD8 ratio of 0.81 ± 0.12, PCR of 0.41 ± 0.23 mg/dL, eGFR of 88.22 ± 22.02 mL/min/1.73 m^2^, total cholesterol of 184.14 ± 34.58 mg/dL while cystatin C was 1.02 ± 0.25 mg/dL. Interleukin levels showed the following mean values: IL‐18 of 270.10 ± 7.44 pg/mL, IL‐2 of 1.69 ± 1.33 pg/mL, IL‐4 of 1.92 ± 3.02 pg/mL, IL‐6 of 3.87 ± 2.58 pg/mL, IL‐10 of 1.17 ± 1.75 pg/mL whereas TNF‐α was 1.31 ± 0.8 pg/mL and IFN‐γ equal to 32.65 ± 17.1 IU/mL. The study of cardiovascular risk scores showed a mean of FRS of 6.98 ± 6.11%, ASCVD of 7.18 ± 6.25%, PROCAM of 6.7 ± 7.4% and DAD Five Years Estimated Risk of 3.10 ± 3.41%. There was a correlation between all the scores for CVR prediction and the years of HIV diagnosis (*p *< 0.001); a correlation between all the CVR scores and IL‐18 (*p *< 0.001); a correlation between circulating IL‐2 with both the FRS and the DAD Five Years Estimated Risk; a correlation between these scores and levels of cystatin C (*p *< 0.001), PCR (*p *< 0.01) and microalbuminuria (*p* = 0.01).

**Conclusion: **Our study shows that exist a correlation between the inflammatory markers and the results obtained from the CVR scores, highlighting how the inflammatory process participates in the pathogenesis of cardiovascular damage in the HIV‐positive population. Therefore the use of these markers could be a valid tool to be used in association with the calculators to highlight the populations at greater risk that require targeted and priority interventions, aimed at reducing future cardiovascular events.

**References**

[1] Smith CJ, Ryom L, Weber R, Morlat P, Pradier C, Reiss P, et al. Trends in underlying causes of death in people with HIV from 1999 to 2011 (D:A:D): a multicohort collaboration. Lancet. 2014;384:241‐8.

[2] Falasca K, Ucciferri C, Mancino P, Di Iorio A, Vignale F, Pizzigallo E, et al. Cystatin C, adipokines and cardiovascular risk in HIV infected patients. Curr HIV Res. 2010;8:405‐10.

[3] Onen NF, Overton ET, Seyfried W, Stumm ER, Snell M, Mondy K, et al. Aging and HIV infection: a comparison between older HIV‐infected persons and the general population. HIV Clin Trials. 2010;11:100‐9.

[4] Ucciferri C, Falasca K, Vecchiet J. Hypertension in HIV: management and treatment. AIDS Rev. 2017;19:198‐211.

## P177

### Impact of HIV infection and antiretrovirals on QT interval: the HIMPAQT study

**C Allavena^1^, N Jacob^2^, J Gourrault^2^, E Billaud^1^, S Sécher^1^, F Raffi^1^ and G Lamirault^2^**

^1^Infectiology, CHU Nantes, Nantes, France. ^2^Cardiology, CHU Nantes, Nantes, France

**Background: **Prevalence of prolonged QT interval on electrocardiograms (ECGs) of HIV‐infected individuals has been described as being higher than in the general population (13 to 20%). Various factors might explain cardiac repolarisation changes in HIV‐infected individuals, including direct effect of HIV, immunodepression, drug toxicity (protease inhibitors [PIs]) and potential confounding cardiovascular cofactors. The objectives of the study were to assess the prevalence of prolonged QTc interval in both ART‐naive and ART‐exposed individuals and to investigate the relation between prolonged corrected QT (QTc) interval and HIV infection or ART.

**Materials and methods: **ECGs of HIV‐infected adults followed between January 2010 and February 2016 in a French tertiary hospital were obtained pre‐ART and on‐ART during a routine visit. ECGs were retrospectively analysed manually by a single investigator. The effect of HIV infection and ART on Bazett QTc interval in the pre‐ART and on‐ART periods was assessed, by matched and multivariable analysis.

**Results: **Of the 255 ECGs in ART‐naïve adults (median age 36.4 years, 72.6% male, median CD4 430/mm^3^, median HIV RNA 4.6 log10 copies/mL, smoker 42.5%, IVDU 5.1%, CDC stage C 14%), a prolonged QTc interval (>440 ms for male and >460 ms for female) was evidenced in 2.3% (n = 6) with values ranging from 443 to 474 ms. Of the 165 individuals with an ECG on‐ART (median duration on ART of 32.5 months, HIV RNA <50 copies/mL in 93%), ECGs on ART were obtained within a median delay between the two ECGs of 35.6 (IQR 19.9 to 58.0) months; a prolonged QTc interval was identified in 4/165 individuals (2.4%). ART regimen included a PI in 24% of cases, a boost (ritonavir or cobicistat) in 39%, TDF/FTC in 62% and ABC/3TC in 27%. The median (IQR) QTc was not significantly different in the pre‐ and on‐ART periods, at 387 (370 to 404) ms and 391 (370 to 404) ms, respectively (*p* = 0.76). In multivariable analysis, the only variable associated to QTc interval for both pre‐ and on‐ART ECGs was female gender. Age was associated to QTc interval duration on pre‐ART ECG (mean QTc difference per year: 0.57 (0.27 to 0.87) ms, *p* = 0.0002). Neither comorbidities nor current ART exposure showed statistical association to QTc interval.

**Conclusion: **Prevalence of QTc prolongation was low both in ART‐naïve and on‐ART individuals. Manually analysed pre‐ART and on‐ART ECGs showed no evidence of impact of HIV infection or ART, including protease inhibitors, on QTc interval.

## COMORBIDITIES AND COMPLICATIONS OF DISEASE AND/OR TREATMENT: MALIGNANCIES

## P178

### Anal cancer risk and use of protease inhibitor: a nested case‐control study within the ANRS CO4‐FHDH cohort

**S Grabar^1^, H Selinger‐Leneman^1^, L Abramowitz^2^, F Boue^3^, M Mary‐Krause^1^, C Duvivier^4^, E Rouveix^5^, I Poizot‐Martin^6^ and D Costagliola^1^**

^1^Sorbonne Université, INSERM, Institut Pierre Louis d'Epidémiologie et de Santé Publique, U1136, Paris, France. ^2^Proctology, Bichat University Hospital, APHP, Paris, France. ^3^Internal Medicine, Immunology, Béclère University Hospital, APHP, Clamart, France. ^4^Service de Maladies Infectieuses et Tropicales, APHP‐Hôpital Necker‐Enfants Malades, Paris, France. ^5^Internal Medicine, Ambroise Paré University Hospital, Paris, France. ^6^Service d'Immuno‐Hématologie Clinique, Aix‐Marseille Univ, APHM Hôpital Sainte‐Marguerite, Marseille, France

**Background: **Several cohort studies have shown an increased risk of anal cancer associated with protease inhibitor (PI) use. However, the analyses were often not adjusted for CD4 nadir, while it is associated both with the risk of anal cancer and ARV treatment initiated with a PI‐based regimen at treatment initiation nor adjusted for the whole ARV treatment history. We aimed at studying the associations between anal cancer risk and ARV uses conducting a nested case‐control study.

**Materials and methods: **Cases were patients enrolled in ANRS CO4‐FHDH while ARV‐naïve who were diagnosed with an incident anal cancer, validated on histological reports, between 1997 and 2008. Up to five controls were selected among patients with no history of anal cancer, followed at the time of anal cancer diagnosis (index date), matched with the case for age (±three years), sex transmission group, centre, CD4 and viral load (VL) at index date and period of inclusion in FHDH (before 1997 or after 1997). We conducted several conditional logistic regression analyses adjusted for the same pool of variables (geographic origin, AIDS stage, hepatitis B and hepatitis C infection, VL at index date and cumulative duration of PI, of NNRTI and of other treatment use per five years of exposure) and with or without additional adjustment for CD4 cell count nadir at treatment initiation or index date if not treated and for cumulative duration of NRTI use.

**Results: **A total of 164 cases and 816 controls were included. Patients were mostly MSM (64%) and aged 44 years at index date. Median year of index date was 2003. Median CD4 cell count nadir was 173/mm^3^ [IQR 76 to 271] for cases and 294/mm^3^ [IQR 180 to 400] for controls. Low CD4 cell count nadir was significantly associated with higher risk of anal cancer. The risk of anal cancer associated with longer PI use ranged from 1.52 to 1.09 in multivariable analyses (Figure 1). It was no longer significant when adjusted for both CD4 nadir and NRTI use (OR 1.09 per year increase, 95% CI 0.97 to 1.22). Main changes in the estimates occurred when analyses were adjusted for NRTI use.

**Abstract P178 – Figure 1.** Risk of anal cancer according to cumulated years of PI use. Results from univariable and multivariable conditional logistic regression models.

Note: OR (95% CI): adjusted OR per year increase of PI use.

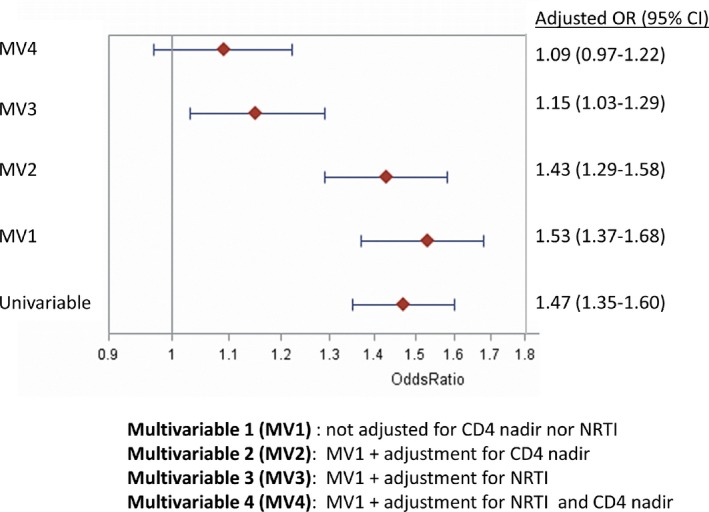

**Conclusion: **Our study shows the importance of taking into account complete ARV exposure as well as the immune depression history of patients when evaluating the risk of developing an anal cancer.

## P179

### Prospective study of the efficacy of 5% imiquimod versus excision of anal HSIL in patients infected by HIV (2010 to 2018)

**C Hidalgo‐Tenorio^1^, C Garcia‐Martinez^1^, C Gil^2^, I Segura^3^, J Esquivias^4^, M Lopez‐Ruz^1^ and J Pasquau^1^**

^1^Infectious Disease Service, University Hospital Virgen de las Nieves, Granada, Spain. ^2^Infectious Disease Unit, Marina Baixa Hospital, Alicante, Spain. ^3^Surgery Service, University Hospital Virgen de las Nieves, Granada, Spain. ^4^Pathology Service, University Hospital Virgen de las Nieves, Granada, Spain

**Background: **Anal squamous cell carcinoma (ASCC) is one of the most frequent non‐AIDS defining malignancies in HIV population. There are different recommendations on the management of these lesions, but they are often based on small observational studies. The main objective of this study were to analyse the real‐life efficacy and safety of the surgery versus 5% imiquimod (IMQ) for the treatment of anal HSIL in our cohort of HIV+ patients; as well as to calculate the prevalance and incidence of anal HSIL, the spontaneous regression of these lesions and the progression rate towards ASCC, and to evaluate the clearance rate of oncogenic HPV genotypes from anal mucosa.

**Methods: **Prospective study (May 2010–May 2018) in HIV+ patients diagnosed with HSIL belonging to a cohort of 486 HIV+ subjects. At the baseline (V1) and follow‐up visits, a cytology, HPV PCR and high resolution anoscopy (HRA) were performed. The patients diagnosed with HSIL were offered surgical treatment, excision (electrocoagulation) or topical 5% IMQ (this was self‐applied in the anal canal three times/week for 16 weeks). Response to treatment was analysed at three to six months after finalising treatment by carrying out a cytology, HPV PCR and HRA. If HRA was normal or LSIL, the patients were followed up annually. If the HSIL lesion persisted, repetition of the surgical excision or another cycle of IMQ was considered. The cytological classification was Bethesda and histological the LAST Project for HPV‐Associated Lesions. 

**Results: **Of the 486 HIV+ patients included in the cohort, 84 were diagnosed with anal HSIL, 86.9% were MSM and 13.1% were women, with an average age of 36 years, 29.8% had history of AIDS, CD4 nadir of 346.5 cells/uL, 83.3% received ART of which 84.2% had viral load HIV <50 copies/μL with 652 CD4+ lymphocytes cells/uL. The prevalance of anal HSIL: 17.2% and the incidence: 8.457 x 1000 patient‐years. 7.4% cases of HSIL regressed spontaneously, 1.2% progressed to ASCC and 1.2% presented new lesion of ASCC five years after surgical intervention of HSIL. 2.3% died before receiving treatment (one lung cancer, one NHL). 43.9% were treated with surgery (SG) as a first option, 46.1% with IMQ. Cure rate was: 75% SG versus 87.1% IMQ, *p* = 0.2. The rate of retreatments: 36.5% SG versus 12.5% IMQ, *p* = 0.02. Adverse events: 95.1% SG versus 4.3% IMQ, *p* = 0.046. The clearance rate of oncogenic HPV genotypes: 64.5% SG versus 35.5% IMQ, *p* = 0.09.

**Conclusion: **Self‐administration of 5% IMQ was an option with a lower rate of retreatments, more comfortable and safer than surgery for the treatment of anal HSIL.

## P180

### Results of HPV testing for anal screening in men who have sex with men

**A Popova^1^, O Shipulina^2^, M Dmitryukova^2^, M Deulina^1^ and V Pokrovsky^1^**

^1^Russian Federal AIDS Center, Central Research Institute of Epidemiology, Moscow, Russian Federation. ^2^Department of Molecular Diagnostics, Central Research Institute of Epidemiology, Moscow, Russian Federation

**Background: **Anal cancer is very rare and accounts for approximately 1 to 2% of all tumours of the gastrointestinal tract in the general population. The cancer of the anal canal is usually associated with the human papillomavirus (HPV). The risk group for this disease is MSM. HIV‐infected MSM have a higher risk of HPV infection, as well as a higher risk of persistence and malignancy.

**Objectives: **To study the prevalence of HPV high carcinogenic risk (HCR) among men.

**Materials and methods: **The work was conducted during the period from February to June 2018. One hundred and fifteen MSM were examined: 58 HIV infected and 57 HIV negative. All men underwent an HPV test with the determination of 14 HPV types of HCR: 16, 18, 31, 33, 35, 39, 45, 51, 52, 56, 58, 59, 66, 68. AmpliSens reagent kits were used. The results were interpreted according to the instructions to the test systems and the software supplied with them.

**Results: **Among the 115 examined men predominated young men aged 32.9 ± 7.58 (min 18, max 50) in both groups. In 75 (65.2%) of them were diagnosed with HPV of HCR by the HPV Pap test. In the HIV‐positive MSM group the percentage of HPV detection was 74.1% (43/58), and 56.1% in the HIV‐negative MSM group (32/57). HIV‐negative MSM in 24.6% (14/57) had mixed genotypes of HPV HCR (maximum five genotypes in one sample). HIV‐infected MSM in 53.4% (31/58) had HPV HCR mixed genotypes (maximum nine genotypes in one sample). The structure of the genotypes of HPV differed in the study groups. In the group of HIV‐infected MSM prevailed 16 (25.9%), 18 (20.7%) and 68 (19%) genotypes of HPV. In the group of HIV‐negative MSM, 68 (14%) and 31 (10.5%) genotypes of HPV prevailed.

**Conclusion: **MSM have a high incidence of HPV infection and a wide range of detectable genotypes of HCR HPV with the highest incidence of HPV infection in HIV‐infected MSM. The results should be used to develop anal screening using HPV testing for at least 13 HCR HPV genotypes.

## P181

### Kaposi's sarcoma in HIV‐1 infected patients: a multicentre cohort experience in Rome

**M Colafigli^1^, A Borghetti^2^, I Fanti^2^, F Di Sora^3^, A Bonadies^4^, V Ferraresi^5^, R Tonachella^5^, F Montella^3^, R Cauda^2^, A Cristaudo^1^, S Di Giambenedetto^2^ and A Latini^1^**

^1^Infectious Dermatology and Allergology, IFO San Gallicano, Rome, Italy. ^2^Clinical Infectious Diseases, Catholic University of S. Heart, Rome, Italy. ^3^Infectious Diseases, AO S. Giovanni, Rome, Italy. ^4^Plastic Surgery, IFO Regina Elena, Rome, Italy. ^5^Clinical Oncology, IFO Regina Elena, Rome, Italy

**Background: **Kaposi's sarcoma (KS) remains a relevant malignancy in HIV‐infected patients. Aim of this work is to describe the patient population with AIDS‐related KS and their management, and to evaluate the incidence of recurrences.

**Methods: **We collected retrospectively data regarding HIV‐infected patients with a diagnosis of KS last seen in our services after 2000. The exposure to combination ART (cART), chemotherapy (CT), electrochemotherapy (ECT) or α‐interferon (α‐IFN) were recorded. Baseline (BL) was set at the diagnosis of KS and follow‐up was censored at last observation or 30 April 2018. Descriptive statistical analysis was performed. The incidence of recurrences was calculated as the number of events during the follow‐up time. The survival free from recurrences was evaluated by Kaplan‐Meier (KM) estimate.

**Results: **A total of 153 patients were included in the database for 1435.39 patient‐year follow‐up (PYFU). Median (IQR) BL calendar year was 2005 (2001 to 2011). At BL, 53 (34.9%) were on cART; of those, 18 (34%) had been exposed to suboptimal therapy, 40 (75.5%) were receiving a standard cART, four (7.5%) a dual regimen and four (7.5%) a monotherapy (less drug regimen [LDR]); 30 (61%) patients were receiving PIs, 12 (24.5%) NNRTIs and two (4.1%) integrase inhibitors. Twenty‐three (15.3%) patients had visceral localisations. Thirty‐eight (25.2%) patients were treated with a median (IQR) of 9.2 (4 to 14.83) cycles of CT (68.4% liposomal doxorubicin, 15.7% poliCT) for a median (IQR) time of 5.4 (2.6 to 9.9) months, four (2.6%) with ECT and 14 (9.2%) with α‐IFN for a median (IQR) time of 13.2 (6.8 to 42.7) months. At last observation 32.5% were receiving a PIr‐based, 20.5% a NNRTI‐based and 17.2% an INI‐based standard cART, 24.5% a LDR and 4% a MegacART. Recurrences were observed in 12 (7.8%) patients for an incidence of 0.84% PYFU with a median (IQR, range) time free from new episodes of 107.8 (51.3 to 169.3, 0.63 to 1433.4) months; of those eight (72.7) were receiving a standard cART, one (9.1%) a dual regimen and one (9.1%) a MegacART; eight (72.7%) were receiving ritonavir‐boosted PIs, one (9.1%) NNRTIs and one (9,1%) a MegacART. Three patients had >1 episode of recurrence. The KM‐estimated time free from recurrences is shown in Figure 1.

**Abstract P181 – Figure 1.** Kaplan‐Meier‐estimated time free from recurrences.

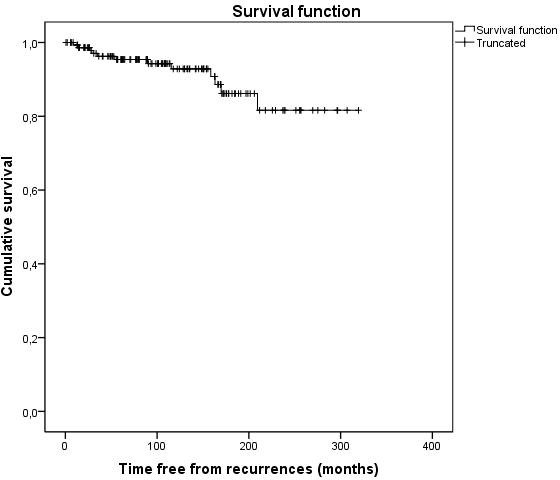

**Conclusions: **KS remains a relevant AIDS‐related event and new diagnoses are still frequent even in recent years. In our experience recurrences are rare and different strategies for its management appeared to be effective and safe.

## P182

### HPV infection among HIV‐positive women in some countries of Eastern Europe and Central Asia

**A Popova^1^, O Shipulina^2^, M Deulina^1^, E Almamedova^3^, A Rzayeva^4^, A Kadyrova^5^, S Grigoryan^6^, A Asmaryan^7^, A Pepanyan^7^, A Davidyan^7^, L Ermolenko^8^, T Nevmerzhitskaya^8^, I Tavtyn^9^, U Kadyrbekov^10^, N Abylgazieva^11^, Z Zhaanbaeva^11^, A Spirin^12^, O Agafonova^13^, M Chesnokov^14^, L Kalenik^14^, S Karimov^15^, R Rahimova^16^, M Rustamova^17^, Z Nurlyaminova^18^, M Dmitryukova^2^ and V Pokrovsky^1^**

^1^Russian Federal AIDS Center, Central Research Institute of Epidemiology, Moscow, Russian Federation. ^2^Department of Molecular Diagnostics, Central Research Institute of Epidemiology, Moscow, Russian Federation. ^3^Director, Republic Center of the Struggle Against AIDS, Baku, Azerbaijan. ^4^Reception Department, Republic Center of the Struggle Against AIDS, Baku, Azerbaijan. ^5^Director, Scientific Research Institute of Lung Diseases, Baku, Azerbaijan. ^6^Director, National Center for AIDS Prevention, Erevan, Armenia. ^7^Medical Care Department, National Center for AIDS Prevention, Erevan, Armenia. ^8^Clinical Department, Svetlogorsk Central Region Hospital, Svetlogorsk, Belarus. ^9^Director, Svetlogorsk Central Region Hospital, Svetlogorsk, Belarus. ^10^Director, Republican AIDS Center, Bishkek, Kyrgyztan. ^11^Department of Dispensary Observation, Republican AIDS Center, Bishkek, Kyrgyztan. ^12^Director, Samara Regional Clinical AIDS Centre, Samara, Russian Federation. ^13^Deputy Chief, Samara Regional Clinical AIDS Centre, Samara, Russian Federation. ^14^Outpatient Department, Samara Regional Clinical AIDS Centre, Samara, Russian Federation. ^15^Director, Republican Center of Prevention and Control HIV, Dushanbe, Tajikistan. ^16^Head, Ministry of Health and Social Protection of Population of Tajikistan, Dushanbe, Tajikistan. ^17^Head, Clinical Medicine of Academy of Medical Sciences of the Ministry of Health and Social Protection of Population, Dushanbe, Tajikistan. ^18^Dispensary Department, Republican Center of Prevention and Control HIV, Dushanbe, Tajikistan

**Background: **The frequency of detection of HPV varies greatly depending on the region of residence of the woman. Also HIV‐infected women have a higher risk of HPV infection than HIV‐negative women, and a higher risk of persistence and malignancy. The aim of the study was: to study the prevalence of human papillomavirus of high carcinogenic risk (HPV HCR) in HIV‐infected women in some countries of Eastern Europe and Central Asia.

**Materials and methods: **Six hundred and forty‐seven HIV‐infected women from Russia, Belorussia, Armenia, Azerbaijan, Tajikistan and Kyrgyzstan were examined from September 2017 to December 2017. All women underwent HPV‐test with the determination of 14 types of HPV HCR (16, 18, 31, 33, 35, 39, 45, 51, 52, 56, 58, 59, 66, 68).

**Results: **Among the 647 women surveyed, mostly young people (under 40 years) predominated. As a result of the HPV‐test, 265 (41%) of HIV‐infected women were diagnosed with HPV HCR. The percentage of HPV detection ranged from 28% to 48%: Armenia 39%, Azerbaijan 43%, Belarus 28%, Kyrgyzstan 46.5%, Tajikistan 37.8%, Russia (Samara) 48%. All 14 HPV HCR genotypes were diagnosed in HIV‐positive women in the region. The distribution of HPV genotypes is different for these countries: 16 and 68 HPV genotypes are registered in Armenia (33.3% and 23%, respectively), in Azerbaijan 16, 18 and 56 HPV genotypes (32.6%, 20.9%, 20.9%), in the Republic of Belarus 16 (28.6%) and 56 (28.6%), in the Republic of Kirghizia 16, 31 and 68 (23.9%, 21.7% and 21.7% respectively), in Tajikistan 16, 31, 56 (29.7%, 21.6%, 21.6%), in Samara (Russian Federation) 52 and 16 (23.6% and 22.2%). HPV infection was caused by a combination of several genotypes in 49.1%. The leading genotypes amond 265 HIV‐infected women with HPV were: 16 genotype 26.4%, 31 genotype 13.6% and 18 genotype 9.4%.

**Conclusion: **There is a high incidence of HPV infection in HIV‐infected women. Given the high risk of developing cervical cancer and the wide spectrum of detectable genotypes of HPV HCR in this group, it is necessary to use a test system to diagnose the 14 genotypes of HPV. The results should be taken into account when planning vaccination in the region.

## P183

### Characteristics of AIDS‐related and non‐AIDS‐related cancers in an Italian cohort of HIV patients in the period 1996 to 2018

**S Nicolè^1^, C Mengoli^2^, G Marini^1^, D Coletto^1^, S Cavinato^1^, S Marinello^1^ and A Cattelan^1^**

^1^Infectious Diseases Department, University Hospital of Padova, Padova, Italy. ^2^Infectious Diseases Department, University of Padova, Padova, Italy

**Background: **The advent of the combined antiretroviral therapy (cART) led to a strong reduction of the incidence of AIDS‐defining cancers (ADCs) and raised life expectancy among PLWHIV [1]. At the same time, non‐AIDS‐defining cancers (NADCs) increased [2,3]. The aim of this study was to define from an epidemiological and prognostic point of view the neoplastic pathology in PLWHIV.

**Materials and methods: **This retrospective cohort study has been carried out at the Infectious Diseases Department of Padova, on PLWHIV with a cancer diagnosis occurred between January 1996 and March 2018. The clinical and immunovirological characteristics and survival rates were analysed comparing two periods of observation (1996 to 2006 vs. 2007 to 2018).

**Results: **One hundred and eighty‐eight patients (35F/153M) were enrolled, for a total of 204 cancer diagnoses; 15 patients had more than one diagnosis. We had 104 ADCs diagnosis and 100 NADCs: 97 cancers from 1996 to 2006, 107 from 2007 to 2018. The most common cancers were NHL (26.0%) and KS (25.0%); among the NADCs, the most frequent were hepatocellular carcinoma (HCC) (8.3%), anal cancer (5.9%) and lung tumour (4.9%). During the 20 years of observation, the ADCs incidence showed a significantly decreasing trend (*p *< 0.01) and the NADCs incidence showed a significantly increasing trend (*p *< 0.01). Mean age at tumour presentation was 43 and 51 years, respectively in the two observational periods. HIV‐RNA at cancer diagnosis was found to be significantly higher in the first period (12,301 copies/mL vs. 151 copies/mL, *p *< 0.01). The CD4 mean count at the tumour diagnosis showed a significant difference (188 and 417, *p *< 0.01), reflecting the major number of patients under cART during the second period. Female sex correlated significantly with both earlier HIV diagnosis and lower HIV‐viraemia at tumour diagnosis. Survival analysis of the entire population is shown in Figure 1 and divided for cancer type in Figure 2. HCC and NHL have been shown to be associated with greater risk of death, while KS and cART at the time of cancer diagnosis were associated with a lower risk of death.

Abstract P183 – Figure 1. Probability of survival over time, expressed in months, of the whole series. Kaplan‐Meier method.

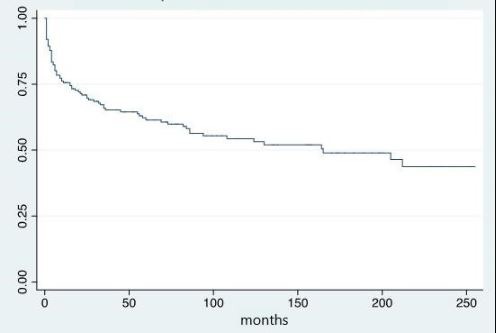

Abstract P183 – Figure 2. Effect on the probability of survival over time (months) exerted by the three most common types of cancer, and by the other types. Kaplan‐Meier method.

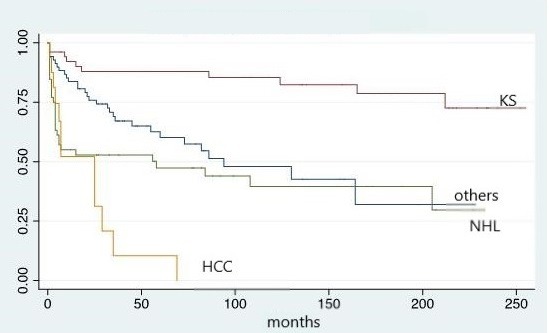

**Conclusions: **In PLWHIV, malignancies remain a major cause of morbidity and mortality and the incidence of NADCs should be expected to increase further as this population continues to age. Although overall cancer survival was significantly improved in the last decade, immunovirological response to cART was not found to be associated with a reduced risk of NADCs. Finally, cancer screening, especially in male population, should be promoted [4].

**References**

[1] Yarchoan R, Uldrick TS. HIV‐associated cancers and related diseases. N Engl J Med. 2018;378:1029‐41.

[2] Rubinstein PG, Aboulafia DM, Zloza A. Malignancies in HIV/AIDS: from epidemiology to therapeutic challenges. AIDS. 2014;28:453‐65.

[3] Brickman C, Palefsky JM. Cancer in the HIV‐infected host: epidemiology and pathogenesis in the antiretroviral era. Curr HIV/AIDS Rep. 2015;12:388‐96.

[4] Goedert J, Hosgood HD, Biggar RJ, Strickler HD, Rabkin CS. Screening for cancer in persons living with HIV infection. Trends Cancer. 2016;2:416‐28.

## P184

### Cancer screening among HIV‐positive patients

**P Wu^1^, M Chen^2^, W Sheng^2^, S Hsieh^2^, Y Chuang^2^, H Chang^1^, Y Luo^1^, S Yang^1^, J Zhang^1^, H Sun^2^ and C Hung^2^**

^1^Center of Infection Control, National Taiwan University Hospital, Taipei, Taiwan. ^2^Internal Medicine, National Taiwan University Hospital, Taipei, Taiwan

**Background: **Access to combination antiretroviral therapy has markedly decreased mortality and morbidity among HIV‐infected patients. HIV‐infected patients are living longer, and cancer has emerged as a leading cause of morbidity and mortality in this population. Based on the practices in the general population, cancer screening of HIV‐infected patients may also confer benefits in decreasing cancer‐related morbidity and mortality. Our study aimed to investigate the utilisation rate of national cancer screening among HIV‐infected patients in Taiwan.

**Methods: **The Taiwan Health Promotion Administration has been expanding the free‐of‐charge screening services for four cancers since 1999, including oral, colorectal, cervical and breast cancers. Taiwan residents aged 50 to 75 years are eligible for biennial, single‐sample faecal immunochemical test (FIT) screening for colorectal cancer; women aged ≥30 years are eligible for annual Pap smear; women aged ≥45 years are eligible for digital breast tomosynthesis on a biennial basis; and individuals aged ≥30 years who are active smokers and betel nut users are eligible for oral examination biennially. Patients seeking care at the HIV clinics of the university hospital were advised to undergo cancer screening from March 2018 to June 2018.

**Results: **During the three‐month study period, 1483 patients met the screening criteria and 1181 (79.6%) completed questionnaire interviews. However, 577 patients (48.9%) refused cancer screening because 164 (28.4%) reported having screening before, 210 (36.4%) having no time and 71 (12.3%) other reasons. In total, 44.1% (522/1181) of the patients completed cancer screening, which included 53.8% (275/511) with FIT screening for colorectal cancer, 33.0% (31/94) of women with Pap smear examination for cervical cancer, 25.4% (17/67) of women with digital breast tomosynthesis for breast cancer and 35.7% (295/287) with oral cancer screening. In multivariate analyses, an older age (adjusted OR 1.044, 95% CI 1.031 to 1.057) and having a family history of cancers (adjusted OR 1.392, 95% CI 1.079 to 1.796) were associated with participation in cancer screening.

**Conclusion: **While cancer screening is provided free of charge in Taiwan, the rate of participating in the programme remains low among HIV‐positive patients. Improving awareness of and accessibility to cancer screening is needed to increase the utilisation rate in this population.

## P185

### HPV‐related pre‐cancerous screening in HIV‐infected MSM accessing the Infectious Diseases Clinic of Modena

**C Rogati^1^, M Digaetano^1^, A Bonazza^1^, M Menozzi^1^, V Borghi^1^, M Pecorari^2^, R Iachetta^3^, R Villani^3^, A Farinetti^4^, F Spatafora^4^ and C Mussini^1^**

^1^Infectious Disease Clinic, University of Modena e Reggio Emilia, Policlinico Hospital, Modena, Italy. ^2^Microbiology and Virology, Policlinico Hospital, Modena, Italy. ^3^Proctology, Nuovo Ospedale Civile di Sassuolo, Sassuolo, Italy. ^4^Proctology, Policlinico Hospital, Modena, Italy

**Background: **Squamous cell carcinoma of anus (SCCA) is strongly associated with HPV infection, particularly with high‐risk HPV genotypes (HR‐HPV). SCCA is one of the most frequent non‐AIDS defining malignancies in HIV patients, especially MSM. According to international guidelines, MSM patients with HIV infection should yearly undergo an anal pap test with cytological analysis. Then, a high‐resolution anoscopy (HRA) is recommended in case of positive cytology. We analysed the prevalence of HPV infection and pre‐cancer lesions and the distribution of HPV genotypes in our cohort, to evaluate any epidemiological and viro‐immunological risk factors associated.

**Materials and methods: **We retrospectively analysed HIV data, anal pap test, HPV genotypes and HRA results of a cohort of 121 HIV‐infected MSM patients accessing our clinic between December 2015 and January 2018, as explained in Figure 1.

**Abstract P185 – Figure 1.** Flowchart of the screening programme.

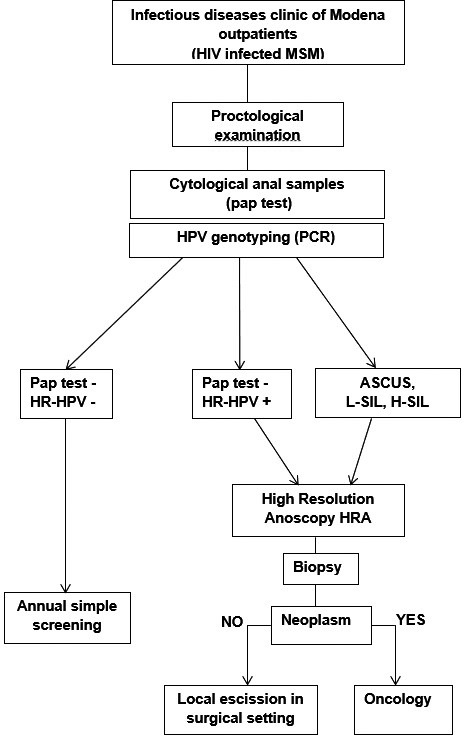

**Results:** ****In Table 1 clinical characteristics of our cohort are shown. Fifty out of 121 swabs (41.3%) resulted positive to cytological analysis for HPV‐related lesions. Among negatives (71), 42 resulted positive for at least one HPV genotype. HR‐HPV genotype were detected in 67% of analysed samples. 65.2% of the screened resulted eligible to HRA (pap‐test+/HR‐HPV+). Atypia or squamous cells of undetermined significance (ASCUS) was found in 2% of the patients, low grade squamous intraepithelial lesion (LSIL) in 35%, high grade squamous intraepithelial lesion (HSIL) in 2%. Focusing on HR‐HPV, HPV‐16 was the most frequent genotype in LSIL and HSIL, meanwhile HPV‐52 and HPV‐58 were the most frequent in negative pap tests. Nineteen patients performed HRA and biopsies, and 14/19 (73.7%) resulted positive for HPV‐related alterations. HRA was determinant for HSIL diagnosis in two cases. We diagnosed two cases of anal carcinoma in situ (CIS) in the follow‐up of patients previously positive for HPV‐related lesions in HRA. Regarding the risk factors, we found that 22 patients with positive pap test suffered from condyloma (44%, *p* = 0.0006). None of HIV‐related features appeared linked to HPV screening positivity.

**Table:** Abstract P185 – Table 1. Characteristics of the 121 enrolled patients

Characteristics	Number of patients, n = 121
Age ± SD	47.2 ± 11
<30 years, n, %	6 (4.9)
30 to 50 years, n, %	71 (58.7)
>50 years, n, %	44 (36.4)
Previous syphilis, %	53 (43.8)
Previous hepatitis A, %	18 (14.8)
Previous condylomatosis, %	33 (27.2)
Previous AIDS‐defining events, %	15 (12.3)
Co‐infections (HBV, HCV), %	8 (6.6)
Nadir CD4, cells/mm^3^ ± SD	326 ± 199
Nadir CD4 <200/mm^3^, %	30 (24.8)
HIV NR, months ± SD	75 ± 134
Current CD4 count,cells/mm^3^ ± SD	793 ± 297
CD4 <200/mm^3^, %	2 (1,6)
Current VL, copies/mL ± SD	19 ± 122
VL >40 copies/mL, %	4 (3.3)
CD/CD8 ratio <0.8, %	53 (43.8)

**Conclusion: **Our results show similar rates of HPV‐related lesions to those reported in literature, and stress the importance of HR‐HPV genotyping in subjects with no evident lesions at a first level evaluation. In our population the occurrence of HPV‐related infections does not correlate with immuno‐virological characteristics, emphasising the importance of primary prevention.

## P186

### The burden of chronic comorbidities among HIV‐infected adults in a large urban HIV clinic in Uganda

**R Musomba Zimaze^1^, N Owarwo^1^, M Nabaggala^2^, M Nsumba^1^, J Matovu^3^, M Lamorde^4^ and B Castelnuovo^1^**

^1^Research, Infectious Diseases Institute, Kampala, Uganda. ^2^Statistics, Infectious Diseases Institute, Kampala, Uganda. ^3^Data Management, Infectious Diseases Institute, Kampala, Uganda. ^4^Clinic, Infectious Diseases Institute, Kampala, Uganda

**Background: **Chronic comorbidities among HIV‐infected persons have increased due to increased access to antiretroviral treatment in Sub‐Saharan Africa [1]. Understanding the epidemiology of such conditions among HIV‐infected persons is essential in planning for health care interventions and optimising clinical care. The objective of the study was to determine the prevalence and risk factors of hypertension (HTN) and diabetes mellitus (DM) among HIV‐infected adults at the Infectious Diseases Institute (IDI), Uganda.

**Methodology: **We included all HIV‐positive patients in care at the IDI between 2014 (when a specialised non‐communicable diseases [NCD] clinic was established) and 31 December 2017. We extracted data from the IDI electronic medical database. Disease definitions were: (1) HTN: systolic >140 mm Hg and diastolic >90 mm Hg; and (2) DM: blood sugar <11 mmol/L. Descriptive statistics and frequency were used. Binary logistic regression was used at the multivariable level adjusting for gender, age, ART status, CD4 count, duration in care, ART duration and ART regimen.

**Results: **Overall 8449 HIV‐infected adults were included, of whom 5278 (62.4%) were females and 8387 (99%) were on ART. The overall prevalence of hypertension was 9.1% (females 55.4%, males 44.6%, *p *< 0.001) while that of diabetes mellitus was 2.5% (females 59.6%, males 40.4%, *p *< 0.001). Medium duration on ART was 9.3 years (IQR 5.2 to 12.3) and 11.3 years (IQR 5.3 to 12.6) for HTM and DM respectively. Predictors of HTM were: male gender (OR 1.09, CI 0.93 to 1.29), age >50 years (OR 4.54, CI 3.85 to 5.35), having CD4 >500 compared to CD4 <500 (OR 1.16, CI 0.831 to 1.61), longer duration in care (OR 1.07, CI 1.02 to 1.11) and longer duration on ART in years (OR 1.03, CI 1.00 to 1.05). Further, predictors of DM were: age >50 years (OR 4.68, CI 4.38 to 5.00), longer duration on ART (OR 1.11, CI 1.10 to 1.12), having CD4 <500 compared to CD4 >500 (OR 1.05, CI 0.92 to 1.19), being on second‐line ART regimen (OR 1.10, CI 1.02 to 1.18) and third‐line ART regimen (OR 2.12, CI 1.53 to 2.93) compared to first‐line ART regimen. One hundred and forty‐nine (1.8%) patients had both HTN and DM.

**Conclusion: **The results demonstrate that hypertension and diabetes mellitus and their risk factors are a growing public health problem especially among male elderly HIV‐positive patients. The findings emphasise need for health care intervention to reduce the growing burden of chronic comorbidities.

**Reference: **[1] Kharsany AB, Karim QA. HIV infection and AIDS in Sub‐Saharan Africa: current status, challenges and opportunities. Open AIDS J. 2016;10:34‐48.

## COMORBIDITIES AND COMPLICATIONS OF DISEASE AND/OR TREATMENT: METABOLIC

## P187

### Increases in lipid profile after switch from TDF to TAF‐based HAART regimens in a cohort of HIV‐positive patients: is it clinically relevant?

**L Gazzola^1^, G Tagliaferri^1^, D Mondatore^1^, A De Bona^1^, C Borsino^2^, T Bini^1^, G Marchetti^1^ and A d'Arminio Monforte^1^**

^1^Infectious Disease, ASST Santi Paolo e Carlo, Milan, Italy. ^2^Pharmacy Unit, ASST Santi Paolo e Carlo, Milan, Italy

**Background: **Switching from TDF to TAF increases lipid profiles; aim of our study is to evaluate whether this is clinically relevant and results in an increased frequency of patients out‐of‐target‐LDL, according to their CV risk score.

**Materials and methods: **All HIV patients switching from TDF to TAF, with no changes of the third drug, and plasma lipids available within six months before and after the switch, were included. Ongoing statin therapy was an exclusion criterion. Demographic, HIV‐related parameters, CV risk factors and lipid profile on TDF and on TAF were collected for each patient. The CV risk SCORE and the target of LDL for each SCORE strata were calculated according to 2016 ESC/EAS guidelines for the management of dyslipidaemias. Modifications in lipid profiles and in the frequency of patients out‐of‐target‐LDL were evaluated after switch to TAF.

**Results: **Two hundred and twenty‐one patients were included: 13 on PI/cobi, 101 on INSTI/cobi, 11 on INSTI, 96 on NNRTI. Median CD4 count at switch was 640 cells/mm^3^ (IQR 493 to 849); HIV‐RNA was <40 copies/mL in 98% of patients. They were classified according to ESC/EAS CV risk SCORE as follows: 111 low, 68 moderate, 10 high and 13 very high. Lipids data on TDF and changes after switch to TAF are represented in Table 1. By analysing lipid profiles according to CV score, 37.5% of patients on TDF had LDL out of target, this proportion increases to 59.5% after switch to TAF (*p *< 0.0001). By univariate analysis, patients on TAF‐based regimens had a double risk of out‐of‐target‐LDL, as compared to TDF‐based regimens [OR 2.4 (95% CI 1.6 to 3.6), *p *< 0.0001]. By analysing data according to cobicistat, switching TDF to TAF in cobicistat‐free regimens results in an increase from 28% to 45% of patients out‐of‐target‐LDL (*p* = 0.01); switching strategy in cobicistat‐based regimens increase this frequency from 46% to 73% (*p* = 0.0001). At univariate analysis, patients switching to TAF in cobicistat‐free regimens had a double risk of out‐of‐target‐LDL, as compared to TDF [OR 2.1 (95% CI 1.2 to 3.8), *p* = 0.01], this relative risk increased to 3.1 (95% CI 1.8 to 5.9, *p *< 0.0001) in cobicistat‐based regimens.

**Conclusion: **Switching strategy from TDF to TAF worsens the lipid profile in HIV‐positive patients, leading to an increasing proportion of patients having LDL over their CV‐related target. This effect is more evident in regimens containing cobicistat. Population intervention on lifestyle and increased prescription of therapeutic intervention has to be considered in the TAF era.

**Table:** Abstract P187 – Table 1. Median changes in lipid profiles after switch from TDF to TAF, according to the third drug

		TDF	TAF	Δ	*p*
PI/c	Total cholesterol	198 (184 to 219)	209 (204 to 236)	+19 (+5 +37)	0.02
	LDL	122 (96 to 133)	129 (115 to 152)	+15 (−9 +25)	0.06
	HDL	47 (33 to 56)	50 (42 to 66)	+7 (+1 +10)	0.04
	Triglycerides	121 (90 to 187)	134 (95 to 159)	+9 (−10 +15)	0.6
INSTI/c	Total cholesterol	181 (163 to 201)	209 (182 to 228)	+26 (+8 +40)	<0.0001
	LDL	108 (93 to 126)	124 (109 to 148)	+18 (+2 +30)	<0.0001
	HDL	46 (38 to 53)	50 (40 to 59)	+4 (−1 +8)	<0.0001
	Triglycerides	117 (88 to 156)	135 (94 to 186)	+12 (−10 +53)	0.0009
INSTI	Total cholesterol	162 (124 to 201)	195 (153 to 217)	+34 (+19 +50)	0.003
	LDL	76 (56 to 115)	100 (83 to 125)	+25 (+10 +32)	0.04
	HDL	44 (38 to 51)	51 (48 to 56)	+4 (+0 +18)	0.1
	Triglycerides	82 (54 to 127)	98 (83 to 139)	+21 (+7 +52)	0.09
NNRTI	Total cholesterol	167 (146 to 185)	186 (165 to 204)	+19 (+6 +34)	<0.0001
	LDL	99 (83 to 117)	114 (95 to 131)	+14 (+0 +25)	<0.0001
	HDL	43 (37 to 50)	49 (40 to 61)	+7 (+2 +14)	<0.0001
	Triglycerides	91 (74 to 127)	111 (82 to 144)	+7 (−16 +41)	0.04

## P188

### Effect of tenofovir alafenamide (TAF) initiation on lipid profile in La Paz Hospital cohort: consequence of TDF withdrawal or TAF initiation?

**R Montejano^1^, B Alejos^2^, R De Miguel^1^, V Moreno^1^, E Valencia^1^, L Martin‐Carbonero^1^, M Montes^1^, C Busca^1^, R Mican^1^, I Perez‐Valero^1^, J Gonzalez‐Garcia^1^, J Arribas^1^, J Bernardino^1^**

^1^HIV Unit, Hospital La Paz, Madrid, Spain. ^2^Thematic Networking (RETICS), Instituto Salud Carlos III, Madrid, Spain

**Background: **TAF is increasingly substituting tenofovir disoproxyl fumarate (TDF) because its advantages on renal function and bone mineral density. TDF has a well‐known lipid‐lowering effect and switching to TAF‐based regimens has been associated with increases in cholesterol fractions in pivotal clinical trials. The objective of this study was to assess if lipid changes after TAF initiation are consequence of TDF withdrawal or TAF initiation.

**Patients and methods**

We performed a retrospective analysis on the La Paz Hospital HIV cohort. Inclusion criteria were: TAF‐based regimen for at least one year, with two blood tests available after switching by June 2018. Participants in clinical trials were excluded. We compared subjects switching to TAF from three different settings: TDF‐based regimens, abacavir‐based regimens and nuke‐sparing regimens. Main outcomes were lipid profile changes at 24 ± 4 and 48 ± 12 weeks from baseline. Linear regression was performed to compare changes by previous regimen group.

**Results: **A total of 323 subjects were included: 276 in the TDF group (50.3% NNRTI, 42.4% INSTI and 7.2% bPI), 28 in the ABC (78.5% INSTI) and 19 in the nuke‐sparing group (84.2% bPI‐based). All TAF/FTC regimens were INSTI‐based (EVG 98.7%). Main baseline characteristics were: median age 48 years (40 to 54), 58 female (18%), Caucasian race 273 (86.6%), sexual acquisition of HIV 70.1%, median HIV duration 13 years (6 to 21), CD4 count 604 cells/mm^3^ (402 to 724) and 92.4% with viral suppression. At 24 weeks, there was a significant increase in total cholesterol (TC) +22.1 ± 31.1 mg/dL, HDL +3.3 ± 8.9 mg/dL, LDL +14.0 ± 25.4 mg/dL, TG +11.5 ± 56.9 while no changes in TC:HDL ratio. At 48 weeks, there was a significant increase in TC +20.9 ± 40.7 mg/dL, HDL +2.7 ± 9.2 mg/dL, LDL +14.5 ± 28.31 mg/dL, TG +22.0 ± 80.4 and TC:HDL +0.23 ± 1.2. Changes intragroup and comparisons between groups are shown in Table 1.

**Table:** Abstract P188 – Table 1. Changes in lipid profile at follow‐up by group (mean ± SD)

		24 weeks (N = 152)					48 weeks (N = 290)		
	ABC/3TC	Nuke‐sparing	TDF/FTC	*p* value^a^	ABC/3TC	Nuke‐sparing	TDF/FTC	*p* value^a^	
TC	+5.2 ± 22.9	+40 ± 57.1	+22.6 ± 29.8^b^	0.08	+16.4 ± 33.1^b^	−4.9 ± 30.7	+23.29 ± 41.4^b^	0.01	
HDL	−1.2 ± 7.6	−0.4 ± 9.05	+3.75 ± 9^b^	0.177	+0.3 ± 9.1	+3.2 ± 7.8	+3.0 ± 9.2^b^	0.38	
LDL	+2.44 ± 18.8	+32 ± 60.3	+14 ± 23.6^b^	0.111	+5.41 ± 28.6	−7.1 ± 23.1	+16.8 ± 27.9^b^	0.001	
TC:HDL	+0.2 ± 0.34	+0.9 ± 1.1	+0.04 ± 0.99	0.12	+0.29 ± 0.79	−0.6 ± 0.6	+0.28 ± 1.28	0.01	
TG	+22.5 ± 43.6	+35.2 ± 37.4	+9.81 ± 58.26	0.52	+13.7 ± 45.3	−10.7 ± 64.2	+24.9 ± 83.4^b^	0.21	
Glucose	−0.8 ± 9.9	+8.66 ± 8.21^b^	+0.15 ± 12.4	0.2170	−0.6 ± 11.4	+10.9 ± 15.1^b^	−0.4 ± 12.1	0.001	

Changes expressed as mg/dL. ^a^aintergroup comparisons. ^b^bintragroup significant increase.

**Conclusion: **These data suggest worsening lipid profiles after switching to TAF, mainly driven by TDF withdrawal. These results should be taken with caution due to small numbers and concomitant TAF and cobicistat use.

## P189

### Prevalence and severity of nonalcoholic fatty liver disease by transient elastography with controlled attenuation parameter: risk factors in unselected HIV mono‐infected population

**G Mazzola^1^, A Cervo^1^, C Gioé^1^, M Trizzino^1^, P Colletti^1^, D Mililli^1^, W Mazzucco^2^, S Mazzola^2^, G Sebastiani^3^, S Petta^4^ and A Cascio^1^**

^1^Infectious Disease Unit, University Hospital P. Giaccone of Palermo, Palermo, Italy. ^2^Clinical Epidemiology and Cancer Registry Unit, University Hospital P. Giaccone of Palermo, Palermo, Italy. ^3^Division of Gastroenterology and Hepatology, McGill University Health Center, Montreal, Canada. ^4^Gastroenterology and Hepatology Unit, University Hospital P. Giaccone of Palermo, Palermo, Italy

**Background: **Nonalcoholic fatty liver disease (NAFLD) is one of the most common causes of liver injury in Western countries. The risk to develop NAFLD is higher for HIV‐infected patients. In non‐HIV patients, prevalence of and the risk factors related to NAFLD are well documented, together with complications. By contrast, there are limited data on NAFLD in the HIV‐infected population [1].

**Materials and methods: **Between January and June 2018, 643 HIV‐infected patients in active follow‐up underwent transient elastography (TE) examination with controlled attenuation parameter (CAP). We prospectively investigated prevalence and predictors of NAFLD and liver fibrosis by TE and CAP in unselected HIV‐infected adults without significant alcohol intake (<20 g/d), viral hepatitis co‐infection and other causes of liver disease. We excluded from the sample all failed TE examination and unreliable measurements. NAFLD and severe NAFLD were defined as CAP at least 248 dB/m and 285 dB/m, respectively. Significant liver fibrosis and cirrhosis were defined as TE measurement at least 7.1 and 13 kPa, respectively [2,3]. Predictors of NAFLD and significant liver fibrosis in patients with steatosis were determined using logistic regression analysis, including covariates that were statistically significant in the univariable analysis. Statistical analyses were performed using IDE RStudio (v.0.98.945).

**Results: **A total of 412 consecutive HIV mono‐infected patients (mean age 47 years, 72% men, mean CD4^+^ cell count 700 cells/μL, 98% on antiretrovirals) were included. Prevalence of NAFLD and severe NAFLD in the cohort is 42.7% and 25%, respectively, while prevalence of significant liver fibrosis and cirrhosis was 14.3% and 2%, respectively. Restricting the sample to steatosic population, prevalence of fibrosis is 9.7%.  After adjustment, NAFLD is associated with BMI, sex, elevated ALT, triglycerides and previous exposure to old NRTI (AZT, ddI, d4T). As expected, BMI is a predictor of significant liver fibrosis in steatosic patients. Conversely, previous use of new protease (ATV, DRV) inhibitors seems to protect against liver fibrosis in patients with NAFLD.

**Conclusion: **In our cohort of HIV mono‐infected patients, NAFLD is frequently observed and a significant proportion of patients has fibrosis. Metabolic conditions and elevated ALT are main predictors. The protective value of new PI versus evolution in fibrosis is interesting although controversial, therefore further investigation is required. Diagnostic assessment with TE examination allows early recognition of NAFLD in HIV population, and consequently, lifestyle modifications to prevent complications and improve liver damage.

**References**

[1] Tafesh ZH, Verna EC. Managing nonalcoholic fatty liver disease in patients living with HIV. Curr Opin Infect Dis. 2017;30:12‐20.

[2] Vuille‐Lessard É, Lebouché B, Lennox L, Routy JP, Costiniuk CT, Pexos C, et al. Nonalcoholic fatty liver disease diagnosed by transient elastography with controlled attenuation parameter in unselected HIV monoinfected patients. AIDS. 2016;30:2635‐43.

[3] Petta S, Di Marco V, Pipitone RM, Grimaudo S, Buscemi C, Craxì A, et al. Prevalence and severity of nonalcoholic fatty liver disease by transient elastography: genetic and metabolic risk factors in a general population. Liver Int. 2018 Mar 25. http://https://doi.org/10.1111/liv.13743. [Epub ahead of print].

## P190

### Waist‐to‐hip ratio is a predictive marker of hepatic steatosis in people living with HIV

**J Mok^1^, L Goff^2^ and B Peters^3^, A Duncan^4^**

^1^School of Medical Education, King's College London, London, UK. ^2^Department of Nutritional Sciences, King's College London, London, UK. ^3^Department of Infectious Diseases, King's College London, London, UK. ^4^Harrison Wing, Guy's and St Thomas’ NHS Foundation Trust, London, UK

**Background: **Hepatic steatosis is highly prevalent in PLWHIV, with aetiology postulated to include viral factors such as hepatitis C, antiretrovirals and metabolic derangement. To date no clinical screening tool to detect hepatic steatosis has been developed. We aimed to investigate hepatic steatosis prevalence in our cohort of PLWHIV and whether any routinely collected clinical data can be used to predict this condition.

**Materials and methods: **Demographic, anthropometric and clinical data were collected from a sample of PLWHIV structured to represent the demographic of three South London clinics. A diagnosis of hepatic steatosis was established by Fibroscan or liver biopsy. Waist and waist:hip ratios were measured using non‐stretch tapes. Using World Health Organization criteria, central obesity was defined as: a waist size greater than 90 cm for male South/East Asians and Central/South Americans, 94 cm for all other males and 80 cm for all women; and a waist:hip ratio >0.9 for males and >0.85 for females. Univariate analysis and binary logistic regression estimated the contribution of a range of factors to hepatic steatosis risk. Receiver operator characteristic (ROC) curves were used to estimate the sensitivity and specificity of using clinical measures to identify hepatic steatosis.

**Results: **Of 338 patients sampled, 71 (21%) had a confirmed diagnosis of hepatic steatosis, with age, non‐Caucasian ethnicity, dysglycaemia and body mass index category all significantly associated with risk (*p *< 0.05 for all). Waist and waist:hip ratios signifying central obesity were significantly associated with hepatic steatosis (*p *< 0.001). ROC curve analysis of waist:hip ratio against hepatic steatosis calculated an area under the curve (AUC) value of 0.788 (95% CI 0.722 to 0.855, *p *< 0.001), with a ratio of >1.0 for males and >0.95 for females identifying hepatic steatosis with a sensitivity of 0.761 and specificity of 0.723. ROC curve analysis for waist calculated an AUC of 0.738 (95% CI 0.662 to 0.813, *p *< 0.001), with circumferences >101 cm and 105 cm for men of South or East Asian/Central or South American and other ethnicities respectively, and >91 cm for women identifying hepatic steatosis with a sensitivity of 0.800 and a specificity of 0.526.

**Conclusion: **Waist:hip ratio can identify hepatic steatosis risk in PLWHIV. Waist circumference is less specific and sensitive than waist:hip ratio but still has validity. We recognise that in clinical practice measuring waist alone may be pragmatic, but recommend using the waist:hip ratio wherever possible to identify hepatic steatosis risk, for example in research.

## P191

### Dysglycaemia is prevalent in HIV patients over 40 and may be detected using routine screening for cardiovascular risk

**J Mok^1^, L Goff^2^ and A Duncan^3^**

^1^School of Medical Education, King's College London, London, UK. ^2^Department of Nutritional Sciences, King's College London, London, UK. ^3^Harrison Wing, Guy's and St Thomas’ NHS Foundation Trust, London, UK

**Background: **Current UK guidelines recommend that all PLWHIV over the age of 40 are screened annually for metabolic comorbidities including glycated haemoglobin (HbA1c) for diabetes risk [1]. However, it is unknown whether these guidelines are best suited for all cohorts with a diversity of age and ethnicity. Secondly, access to HbA1c screening may not be universal or may not be routinely measured in clinical practice. We aimed to investigate the prevalence of pre‐diabetes and type 2 diabetes (T2DM) in PLWHIV, and to assess whether the cardiovascular risk tool QRISK2 can identify PLWHIV at risk of diabetes.

**Materials and methods: **A cohort of HIV‐positive adults was purposively sampled in order to represent the demographic of three large South London clinics. Baseline demographic, anthropometric and routine clinical data were gathered and analysed. Glycaemic status was established using World Health Organization criteria for normoglycaemia, pre‐diabetes and T2DM (<6.0, 6.0 to 6.9 and ≥7.0 mmol/L respectively) or by previous diagnosis. QRISK2 was used to calculate 10‐year cardiovascular risk. Data were analysed using SPSS Statistics v.23. Continuous variables were checked for normality, and chi‐squared and ANOVA tests used to estimate the strength of relationships. The sensitivity and specificity of QRISK2 scores for predicting pre‐diabetes was estimated using a receiver operator characteristic (ROC) curve.

**Results: **In this ethnically diverse (49.7% Caucasian) cohort (n = 338) with a median age of 49 (interquartile range 42 to 57) dysglycaemia was highly prevalent, with pre‐diabetes and T2DM diagnosed in 17% and 15% of participants respectively. Age over 40 years was significantly associated with dysglycaemia (*p *< 0.001) but ethnicity was not (*p* = 0.751). ROC curve analysis for those with pre‐diabetes estimated an area under the curve of 0.653 (95% CI 0.582 to 0.725; *p *< 0.001), with a 10‐year CVD risk of ≥4% suggesting a sensitivity of 72% and a specificity of 51% for increased diabetes risk.

**Conclusion: **Our findings provide extra evidence that annual screening for diabetes risk should be routine in PLWHIV over the age of 40 in accordance with the current BHIVA guidelines. For individuals where in error glycated haemoglobin has not been measured, a 10‐year cardiovascular risk of 4% or more estimated by the QRISK2 tool may be used as a surrogate marker to detect patients with undiagnosed pre‐diabetes or type 2 diabetes and warrant more urgent further investigations.

**Reference: **[1] British HIV Association (BHIVA). BHIVA guidelines for the routine investigation and monitoring of adult HIV‐1‐positive individuals [Internet]. 2016 [cited 2018 Jul 4]. Available from: http://www.bhiva.org/monitoring‐guidelines.aspx.

## P192

### Current or historic HIV‐related lipodystrophy largely associated with protease inhibitor exposure is a predictor of future diabetes risk

**J Mok^1^, L Goff^2^, B Peters^3^ and A Duncan^4^**

^1^School of Medical Education, King's College London, London, UK. ^2^Department of Nutritional Sciences, King's College London, London, UK. ^3^Department of Infectious Diseases, King's College London, London, UK. ^4^Harrison Wing, Guy's and St Thomas’ NHS Foundation Trust, London, UK

**Background: **HIV infection and its management have been implicated in the development of metabolic comorbidities including increased risk for type 2 diabetes. In vitro studies suggest a role for antiretroviral therapy in mediating lipodystrophy through mitochondrial toxicity and pro‐inflammatory mechanisms, potentially exacerbating metabolic comorbidity risk [1]. We aimed to investigate the relationship between lipodystrophy and dysglycaemia in an urban HIV cohort and also investigate any associated clinical parameters.

**Materials and methods: **Clinical parameters, demographics and anthropometric data were collected from a cohort of PLWHIV sampled to be statistically representative of patients attending three South London clinics. Current and historic duration of exposure to individual antiretrovirals, statins and corticosteroids was recorded. Historic or current lipodystrophy was recorded as one of three categories clinically assessed by a specialist metabolic HIV consultant physician: lipoatrophy, lipohypertrophy and mixed lipodystrophy. Glycaemic status was defined as either normal or dysglycaemia using glucose measured after a 10‐hour fast (* *<6.0 and ≥6.0 mmol/L respectively). Univariate statistical analysis and binary logistic regression were used to estimate risk factors for lipodystrophy, and their relative contributions to dysglycaemia.

**Results: **Past or current lipodystrophy was present in 22% (n = 73) of 310 patients, with a significant correlation between lipodystrophy and dysglycaemia (*p* = 0.016). Of those with lipodystrophy, 8% were classified with lipohypertrophy, 40% with lipoatrophy and 52% with mixed. For all patients with past or current lipodystrophy the odds ratio of developing dysglycaemia was 2.05 (95% CI 1.20 to 3.49, *p* = 0.008). Univariate analysis suggests that age, corticosteroid and statin use are significantly associated with lipodystrophy (*p *< 0.001, *p* = 0.042 and *p* = 0.003 respectively) but nucleoside reverse transcriptase inhibitors, non‐nucleoside reverse transcriptase inhibitors and body mass index were not. Binary logistic regression suggests that current or historic protease inhibitor use remains significantly associated with current or historic lipodystrophy when controlling for age, corticosteroid and statin use (OR 4.70, 95% CI 2.39 to 9.25, *p *< 0.001) but duration of exposure to any individual protease inhibitor was not.

**Conclusion: **Lipodystrophy is significantly correlated with future diabetes risk in PLWHIV. Our findings suggest current or historic exposure to protease inhibitors is strongly implicated with lipodystrophy even after adjusting for covariants, providing additional evidence for previous observations from other studies. In clinical practice we suggest that patients with current or historic lipodystrophy should be screened for diabetes.

**Reference: **[1] Mallewa JE, Wilkins E, Vilar J, Mallewa M, Doran D, Back D, et al. HIV‐associated lipodystrophy: a review of underlying mechanisms and therapeutic options. J Antimicrob Chemother. 2008;62:648‐60.

## P193

### The prevalence of diabetes mellitus type 2 in HIV‐1 infected patients and antiretroviral treatment utilisation in Ukraine: the results of observational multicentre cross‐sectional retrospective study

**S Antonyak^1^, I Matkovskyi^2^, O Yurchenko^3^, S Antoniak^1^, T Adamczewski^4^ and A Koycheva^5^**

^1^HIV/HCV Department, Clinic of the Gromashevsky Institute of Epidemiology and Infectious Diseases, Kiev, Ukraine. ^2^Vinnytsya Regional Clinical AIDS Center, Vinnytsya, Ukraine. ^3^City Center for HIV/AIDS Prevention and Control, Municipal Clinical Hospital No 5, Kiev, Ukraine. ^4^Medical Affairs, MSD, GMA Poland, Warsaw, Poland. ^5^Medical Affairs, MSD, GMA Ukraine, Kiev, Ukraine

**Background: **Based on published data 4.3% to 12.6% of patients with HIV have diabetes mellitus type 2 (T2DM) [1], which is the second most prevalent comorbidity in PLWHIV [2]. Patients with T2DM belong to a high cardiovascular risk (CVR) group [3,4]. ARV regimens need to be carefully tailored in HIV‐infected patients with T2DM and high CVR due to potential drug‐drug interactions (DDI), tolerability and long‐term adverse effects. For instance, PI‐ and EFV‐based regimens may negatively affect lipids profile [5,6], LPV may facilitate the development of insulin resistance [7] and dolutegravir has DDI with metformin [8], which is the most commonly used treatment in T2DM patients. The aim of the study was to assess the prevalence of T2DM in PLWHIV in Ukraine and the patterns of ART regimens utilisation in this patient group.

**Materials and methods: **Retrospective, observational, multicentre cross‐sectional study. Two thousand four hundred patients with HIV‐1 infection, who were under care in three large urban HIV centres in Ukraine in 2017, were randomly enrolled in the study. Medical records were used as the primary source documents.

**Results: **The prevalence of T2DM among PLWHIV was 4.75% (114 out of 2400). 87.72% (n = 100) patients with HIV and T2DM were not previously diagnosed with T2DM, and 88.60% (n = 101) did not receive hypoglycaemic drugs. The most commonly prescribed hypoglycaemic drug was metformin (seven out of 13). Patients with HIV‐1 infection and T2DM were generally older than patients with HIV‐1 without T2DM, 43.84 ± 9.86 years and 39.08 ± 8.07 respectively (*p *<* *0.0001). BMI among patients with T2DM was higher than among patients without T2DM, 24.3 and 23.3 respectively (*p *=* *0.009). The majority of patients with T2DM (60%) received EFV‐containing regimens, 24.55% LPV/r and 8.18% DTG.

**Conclusions: **The prevalence of T2DM among PLWHIV in Ukraine was comparable to that described in the literature. Almost 90% of identified T2DM patients were not under T2DM‐related medical care including treatment. Based on the results of this study we may conclude that diagnosis and management of patients with HIV and T2DM must be improved, including considerations about the optimal ARV regimens and T2DM treatment.

**References**

[1] Vance DE, Mugavero M, Willig J, Raper JL, Saag MS. Aging with HIV: a cross‐sectional study of comorbidity prevalence and clinical characteristics across decades of life. J Assoc Nurses AIDS Care. 2011;22:17‐25.

[2] Guaraldi G, Zona S, Brothers TD, Carli F, Stentarelli C, Dolci G, et al. Aging with HIV vs. HIV seroconversion at older age: a diverse population with distinct comorbidity profiles. PLoS One. 2015;10:e0118531.

[3] Piepoli MF, Hoes AW, Agewall S, Albus C, Brotons C, Catapano AL, et al. 2016 European guidelines on cardiovascular disease prevention in clinical practice. Eur Heart J. 2016;37:2315‐81.

[4] Taylor KS, Heneghan CJ, Farmer AJ, Fuller AM, Adler AI, Aronson JK, et al. All‐cause and cardiovascular mortality in middle‐aged people with type 2 diabetes compared with people without diabetes in a large U.K. primary care database. Diabetes Care. 2013;36:2366‐71.

[5] Rockstroh JH, DeJesus E, Lennox JL, Yazdanpanah Y, Saag MS, Wan H, et al. Durable efficacy and safety of raltegravir versus efavirenz when combined with tenofovir/emtricitabine in treatment‐naive HIV‐1‐infected patients: final 5‐year results from STARTMRK. J Acquir Immune Defic Syndr. 2013;63:77‐85.

[6] Lennox JL, et al. Efficacy and tolerability of 3 nonnucleoside reverse transcriptase inhibitor‐sparing antiretroviral regimens for treatment‐naive volunteers infected with HIV‐1: a randomized, controlled equivalence trial. Ann Intern Med. 2014;161:461‐71.

[7] Djedaini M, et al. Lopinavir co‐induces insulin resistance and ER stress in human adipocytes. Biochem Biophys Res Commun. 2009;386:96‐100.

[8] Zong J, et al. The effect of dolutegravir on the pharmacokinetics of metformin in healthy subjects. J Int AIDS Soc. 2014;17:19584.

## P194

### Plasma NRTI exposure and associations with serum alanine aminotransferase in people living with HIV

**X Wang^1^, M Boffito^2^, L Dickinson^3^, E Bagkeris^4^, S Khoo^3^, F Post^5^, J Vera^6^, I Williams^4^, D Babalis^7^, J Anderson^8^, P Mallon^9^, M McClure^1^ and A Winston^1^, C Sabin^4^**

^1^Department of Medicine, Imperial College London, London, UK. ^2^St Stephen's Centre, Chelsea and Westminster Hospital, London, UK. ^3^Institute of Translational Medicine, University of Liverpool, Liverpool, UK. ^4^Infection & Population Health, University College London, London, UK. ^5^School of Immunology & Microbial Sciences, Kings College London, London, UK. ^6^Wellcome Trust Centre for Global Health, Brighton and Sussex Medical School, Falmer, UK. ^7^School of Public Health, Imperial College London, London, UK. ^8^Centre for the Study of Sexual Health and HIV, Homerton University Hospital NHS Foundation Trust, London, UK. ^9^School of Medicine, University College Dublin, Dublin, UK

**Background: **NRTIs can induce hepatocyte damage through reduced cell proliferation, mitochondrial toxicity and mitochondrial DNA (mtDNA) loss. Whilst abnormal liver function tests are observed in PLWHIV on ART, correlations of NRTI plasma exposure with markers of liver function are ill‐defined. We investigated the associations of tenofovir (TFV), emtricitabine (FTC), abacavir (ABC) and lamivudine (3TC) pharmacokinetics (PK) with alanine transaminase (ALT) as part of a large cohort study of PLWHIV.

**Methods: **Plasma NRTI concentrations were measured by ultra‐high performance liquid chromatography (one PK sample/participant) in PLWHIV receiving tenofovir disoproxil fumarate (TDF), ABC, FTC or 3TC. Population PK models were developed to predict PK parameters that included area under the curve (AUC24 h), maximum concentration (Cmax), trough concentration (C24 h) and apparent oral clearance (CL/F). Linear regression analysis determined the association between ALT and PK parameters after adjustment for age, gender, ethnicity, current use of boosted PIs, efavirenz or nevirapine, hepatitis B or C virus co‐infection, current use of alcohol, recreational drugs, lipid lowering drugs and body mass index (BMI). 

**Results: **Of the 488, 452, 92 and 122 participants with PK samples for measurement of TFV, FTC, ABC and 3TC, respectively, 14.3%, 13.3%, 14.1% and 15.5% had hepatitis B virus co‐infection. Most were white (90.2%) and male (87.6%) with a median age of 52 (range 23 to 82) years. Median (range) ALT was 30 (6 to 99), 30 (6 to 99), 27 (8 to 89) and 27 (7 to 89) U/L, respectively. In univariate analyses, ALT values inversely correlated with TFV AUC24 h (*p *<* *0.001), Cmax (*p *<* *0.001) and C24 h (*p *=* *0.003), while for CL/F there was a positive correlation (*p *<* *0.001). These associations were substantially attenuated after adjustment for confounders (post‐hoc analysis suggested that adjustment for BMI explained most of the attenuation) (Table 1). A weaker association between FTC PK parameters and ALT could be explained by co‐administration of TFV in the regimen (associations with TFV PK parameters were similar, regardless of whether FTC was or was not included in the regimen). No associations were observed between ALT and either ABC or 3TC PK parameters.

**Table:** Abstract P194 – Table 1. Univariable and multivariable association of ALT with TFV PK parameters

		Univariable models		Multivariable model 1^a^		Multivariable model 2^b^	
	N	β‐coef. (95% CI)	*p*‐value	β‐coef. (95% CI)	*p*‐value	β‐coef. (95% CI)	*p*‐value
AUC0‐24 (mg.h/L)			<0.001		<0.001		0.09
≤2.355	98	Ref.	‐	Ref.	‐	Ref.
2.356 to 2.612	98	−3.93 (−8.55, 0.69)		−5.06 (−9.64, −0.48)		−2.66 (−7.31, 1.99)
2.613 to 2.928	97	−7.22 (−11.85, −2.58)		−7.84 (−12.47, −3.22)		−4.35 (−9.19, 0.49)
2.929 to 3.410	98	−7.97 (−12.59, −3.35)		−9.51 (−14.29, −4.73)		−4.58 (−9.83, 0.67)
≥3.411	97	−9.23 (−13.86, −4.59)		−9.94 (−14.82, −5.05)		−4.71 (−10.12, 0.70)
Cmax (mg/L)			<0.001		<0.001		0.54
≤0.219	100	Ref.	‐	Ref.	‐	Ref.	‐
0.220 to 0.241	99	−2.00 (−6.56, 2.57)		−2.61 (−7.16, 1.94)		0.47 (−4.27, 5.21)
0.242 to 0.263	98	−6.50 (−11.08, −1.92)		−7.13 (−11.66, −2.59)		−2.18 (−7.29, 2.93)
0.264 to 0.289	95	−9.13 (−13.74, −4.51)		−8.74 (−13.36, −4.11)		−2.49 (−8.01, 3.04)
≥0.290	96	−8.77 (−13.37, −4.16)		−8.46 (−13.27, −3.64)		−0.65 (−6.79, 5.48)
Trough (mg/L)	0.003	0.003	0.18
≤0.042	101	Ref.	‐	Ref.	‐	Ref.	‐
0.043 to 0.049	95	−3.21 (−7.88, 1.46)	−3.62 (−8.33, 1.09)	−2.41 (−7.02, 2.21)
0.050 to 0.057	98	−5.54 (−10.17, −0.91)	−5.99 (−10.70, −1.28)	−3.40 (−8.10, 1.29)
0.058 to 0.072	99	−5.94 (−10.56, −1.32)	−6.89 (−11.86, −1.93)	−3.73 (−8.72, 1.25)
≥0.073	95	−6.50 (−11.17, −1.83)	−7.05 (−12.15, −1.95)	−3.47 (−8.63, 1.69)
Clearance (L/h)	<0.001	<0.001	0.006
≤40.523	98	Ref.	‐	Ref.	‐	Ref.	‐
40.524 to 46.973	98	0.96 (−3.62, 5.53)	0.76 (−3.83, 5.35)	−0.18 (−4.78, 4.42)
46.974 to 52.448	97	3.61 (−0.97, 8.20)	4.15 (−0.53, 8.83)	1.94 (−2.85, 6.74)
52.449 to 58.096	98	7.14 (2.57, 11.72)	7.34 (2.54, 12.13)	4.38 (−0.66, 9.41)
≥58.097	97	11.02 (6.43, 15.60)	11.92 (7.04, 16.80)	6.78 (1.15, 12.41)

^a^aadjusted for age at baseline, gender, ethnicity, use of boosted PIs, efavirenz or nevirapine as part of current regimen, HCV, HBV, current alcohol use, recreational drugs in past six months and receipt of lipid lowering drugs. ^b^additional post hoc including further adjustment for BMI.

**Conclusions: **We have observed a correlation between higher TFV plasma exposure and lower ALT concentrations, but no association between exposure of the other NRTIs and ALT concentration. These observations were strongly attenuated by BMI.

## P195

### Lipid changes after switch from tenofovir disoproxil fumarate to tenofovir alafenamide: a longitudinal cohort study

**F Berger^1^, A Milinkovic^2^, A Arenas‐Pinto^3^ and S Mauss^1^**

^1^HIV and Hepatogastroenterology, Center for HIV and Hepatogastroenterology, Düsseldorf, Germany. ^2^Department of HIV Medicine, Chelsea and Westminster Hospital, London, UK. ^3^Institute for Global Health, University College London, London, UK

**Introduction: **Switching from tenofovir disoproxil fumarate (TDF) to tenofovir alafenamide (TAF) has been recommended as strategy to reduce the risk of renal and bone toxicities. However, its effect on lipid profile has not been a focus of interest.

**Methods: **This analysis consists of a retrospective data collection on effectively suppressed HIV‐positive patients who were switched from TDF‐ to TAF‐based ART due to medical reasons (bone, kidney disease) or as a result of optimisation of therapy, in a single site (Center for HIV and Hepatogastroenterology, Düsseldorf). All components of ART for all patients were maintained with the single substitution of TDF to TAF. Only patients on stable lipid‐lowering therapy were included. Lipid profile was measured before switch and at 12‐week intervals after initiation of TAF.

**Results: **A total of 385 virologically suppressed patients on stable TDF‐containing regimens were included. Median age was 50 (IQR 42 to 56) years, 90% were male, 93% Caucasian, with a median BMI of 23.52 kg/m^2^ (IQR 21.26 to 25.61) at switching. Statin use was reported in 11% of patients and diabetes in 5%. At switching, mean triglycerides were 126 mg/dL (IQR 87 to 191) and triglycerides >200 mg/dL were reported in 21% of patients, mean total cholesterol (TC) was 186 mg/dL (IQR 162 to 210) and 7% of patients had TC >240 mg/dL, mean LDL‐cholesterol  was 71 mg/dL (IQR 95 to 139). At 12 and 24 weeks, mean triglycerides had increased by +25.75 mg/dL (SD 100.54) and +13.09 mg/dL (SD 44.26), and TC had increased by +20.33 mg/dL (SD 26.38) and +13.09 mg/dL (SD 44.26) after switch to TAF, respectively, and differences in mean changes were significant (*p *<* *0.0001) for both parameters, mean ratio of TC/HDL‐cholesterol showed a significant decrease three months after switching −2.31 (SD 13.35) (*p *<* *0.01). Regardless of baseline TC, switching to TAF increases mean TC (*p *<* *0.0001), the odds of having TC >240 mg/dL after switching was associated with older age (increased by 2% per year; *p *=* *0.027), BMI >25 kg/m^2^ (*p *=* *0.020) and elevated baseline LDL‐cholesterol (*p *<* *0.001). In the multivariable model, age >50 and BMI >25 kg/m^2^ remained independently associated with TC >240 mg/dL (OR 1.58 and 2.08 respectively).

**Conclusions: **Results of our study show that patients with pre‐existing higher CV risk are more likely to show increases in lipids after switching from TDF which may worsen the cardiovascular risk profile. In these patients risk and benefit of switching TDF to TAF should be carefully accessed, taking lipids in consideration.

## P196

### Correlation between PAI‐1, leptin and ferritin with HOMA in HIV/AIDS patients

**G Dragovic^1^, M Sumarac Dumanovic^2^, K Al Musalhi^3^, I Soldatovic^4^, B Dimitrijevic^1^, D Jevtovic^5^ and D Nair^3^**

^1^Department of Clinical Pharmacology, University of Belgrade, School of Medicine, Belgrade, Serbia. ^2^Endocrinology, Diabetes, Metabolism Disease, University of Belgrade, School of Medicine, Belgrade, Serbia. ^3^Department of Clinical Biochemistry, Royal Free Hospital and University College London, London, UK. ^4^Department of Biomedical Statistics, University of Belgrade, School of Medicine, Belgrade, Serbia. ^5^HIV/AIDS Center/Infective/Tropical Disease Clinic, University of Belgrade, School of Medicine, Belgrade, Serbia

**Background: **Data about correlation of interleukins (IL‐1α, IL‐1β, IFNγ, IL‐2, IL‐4, IL‐6, IL‐8, IL‐10), adipocytokines (leptin, adiponectin, monocyte chemoattractant protein‐1 [MCP‐1], resistin, plasminogen activator inhibitor‐1 [PAI‐1], tumour necrosis factor alpha [TNFα]), ferritin, C reactive protein (CRP) and vascular endothelial growth factor (VEGF)) with homeostasis model assessment (HOMA) in HIV/AIDS patients are still limited. Therefore, the aim of this study was to evaluate the possible correlations of serum interleukins (IL‐1α, IL‐1β, IFNγ, IL‐2, IL‐4, IL‐6, IL‐8, IL‐10) levels, adipocytokines (leptin, adiponectin, MCP‐1, resistin, PAI‐1, TNF α), ferritin, CRP and VEGF with HOMA in HIV/AIDS patients treated with combined ART (cART).

**Materials and methods: **This cross‐sectional study included 64 HIV/AIDS patients, all Caucasians, receiving cART at the HIV/AIDS Centre. PAI‐1, leptin, ferritin and insulin levels were measured using the Metabolic Syndrome Array I (Randox Laboratories Ltd., London, UK), while adiponectin and resistin levels were measured using Metabolic Syndrome Array II (Randox Laboratories Ltd., London, UK), interleukins (IL‐1α, IL‐1β, IFNγ, IL‐2, IL‐4, IL‐6, IL‐8, IL‐10), MCP‐1, TNF‐α as well as VEGF was measured using Cytokine Array I (Randox Laboratories Ltd., London, UK). Insulin resistance was determined using the homeostasis model assessment index (HOMA). All biochemical analyses were performed at the Department of Clinical Biochemistry, Royal Free Hospital and University College London, UK. Multicollinearity of independent variables in multivariate model was analysed using variance inflation factor.

**Results: **Correlation analysis revealed significant correlations between HOMA and waist circumference, body mass index, patients’ age, number of cART combinations and triglycerides (*p *=* *0.001, *p *=* *0.001, *p *=* *0.050, *p *=* *0.044, *p *=* *0.002, respectively). HOMA negatively correlated with levels of high density lipoprotein (Rho=−0.282, *p *=* *0.025). PAI‐1 (Rho = 0.334, *p *=* *0.007) and leptin (Rho = 0.492, *p *=* *0.001) together with ferritin (Rho = 0.396, *p *=* *0.001) levels positively and significantly correlated with HOMA. Levels of IL‐1α, IL‐1β , IFNγ, IL‐2, IL‐4, IL‐6, IL‐8, IL‐10, adiponectin, MCP‐1, resistin, TNFα, CRP and VEGF did not significantly correlate with HOMA. Further, multiple logistic regression showed that there is a statistically significant correlation between PAI, leptin and ferritin with HOMA levels (*p *=* *0.042, *p *<* *0.001, *p *=* *0.009, respectively).

**Conclusions: **In accordance with our knowledge we showed for the first time significant correlation between PAI‐1, leptin and ferritin, independently of each other with HOMA, in HIV/AIDS patients on cART.

## P197

### Evaluation of the dual X‐ray absorptiometry to predict metabolic syndrome in HIV‐infected patients

**M Fontecha, M Monsalvo, M Vivancos and A Moreno and J Casado**

Infectious Diseases, Hospital Universitario Ramón y Cajal, Madrid, Spain

**Background: **There are no studies in HIV patients that correlate fat mass determinations as assessed by dual X‐ray absorptiometry (DXA) with the development of the metabolic syndrome (MS), neither its relative value in comparison with other anthropometric measurements.

**Material and methods**

Prospective study of 276 patients included and followed in a study about lipodystrophy and MS (NCT02614027). Body composition was evaluated with DXA scans by using an Hologic QDR‐4500 model. At the same time, patients underwent measurements of weight, height, BMI, waist circumference and waist‐hip ratio. MS was diagnosed attending to the criteria established by Adult Treatment Panel III (ATP‐III).

**Results: **Overall, 111 patients (45%) met the diagnostic criteria of MS during the study. Mean age was 45.1 years (20 to 80) and 80% were male. Median time since DXA to evaluation of MS was 41 months. A final diagnosis of MS was correlated with higher body mass index (BMI, 25.9 vs. 23.6 kg/m^2^), higher trunk fat mass (%; 32.14% vs. 27%, *p *<* *0.01), lower fat mass (%) in arms (21.7% vs. 31.6%, *p *<* *0.01), and there was no relationship between MS and legs fat mass. The fat mass ratio (FMR, % trunk/limbs fat mass) was also significantly higher (1.69 vs. 1.27). In addition, patients with MS presented higher waist circumference (97.55 vs. 86.7, *p *<* *0.01), as well as a higher waist‐hip ratio (0.97 vs. 0.9, *p *<* *0.01). There was a significant correlation between waist circumference and DXA trunk fat mass (*r* = 0.81, *p *<* *0.01). In a ROC curve analysis in males, waist circumference (AUC 0.8), BMI (AUC 0.74), trunk fat mass (AUC 0.74), FMR (AUC 0.77) and the percentage of trunk fat mass (AUC 0.7) showed adequate usefulness for MS diagnosis, better than that observed with legs fat mass, or with the waist‐hip ratio. A value of FMR of 2 showed a positive predictive value (PPV) of 70% to diagnosis MS, whereas a cutoff value of 102 cm for the waist circumference (19% of cases) denotes a PPV of 85%. However, a higher negative predictive value (NPV) was found for the percentage of trunk fat mass in patients without abdominal obesity (NPV 95% for abdominal fat ≤18%, NPV 85% for waist circumference).

**Conclusion: **DXA fat determinations could be useful in the prediction of MS in HIV‐positive patients, by offering additional data to those obtained through anthropometric measurements.

## P198

### How well do we manage type 2 diabetes and abnormal glucose metabolism in HIV? A service evaluation

**H Smith^1^, P Hine^1^, M Beadsworth^1^, P Weston^2^ and M Chaponda^1^**

^1^Tropical and Infectious Diseases, Royal Liverpool University Hospital, Liverpool, UK. ^2^Diabetic Medicine, Royal Liverpool University Hospital, Liverpool, UK

**Background: ** HIV and antiretrovirals have been suggested as risk for type 2 diabetes mellitus (T2DM). Meanwhile, if T2DM is not well managed, the gains in healthy life expectancy offered by antiretrovirals may not be realised.

**Materials and methods: **We analysed cross‐sectional data from a teaching hospital in the UK in 2018. We collected data on the following outcome measures: HbA1c < target; latest blood pressure <140/90 mmHg; not smoking; viral suppression. We collected data on process measures including: communication with primary care; monitoring and recording of results; appropriate prescribing.

**Results: **Of 1123 patients, 53 patients had a recorded diagnosis of T2DM, and eight had recorded non‐diabetic hyperglycaemia (age‐adjusted prevalence 3.4%). For outcome measures: 18 patients (30%) achieved the HbA1c target; of 35 patients (69%) with a recorded diagnosis of hypertension, 24 (69%) achieved the blood pressure target; 48 (80%) were non‐smokers. For process measures: 58 patients (97%) had recorded communication with primary care; five (8.3%) had unrecorded HbA1c data; 60 (100%) were prescribed antiretrovirals, and only one patient had a detectable viral load. Diabetes management strategies were: diet control (15 patients, 20%); biguanides (30 patients, 50%); sulfonylureas (gliclazide) (10 patients, 17%); gliptins (five patients, 8%); insulin regimens (13 patients, 22%). We identified the suboptimal prescribing: eight patients (13%) received abacavir which may confer higher cardiovascular risk; four of 19 (21%) of patients with renal impairment received tenofovir‐disproxil or atazanavir; one patient (1.7%) received co‐prescription of dolutegravir and 1 g daily metformin.

**Conclusions: **Prevalence of T2DM and non‐diabetic hyperglycaemia was lower in our cohort than UK national average (6%), indicating possible under‐diagnosis. HIV disease was well controlled in all but one patient. However, we identified suboptimal glycaemic and hypertensive control and suboptimal prescribing of ART in high‐risk groups. We need to better manage diabetes in HIV to reap the benefits of gains in healthy life expectancy provided by antiretrovirals. This might be achieved through clinician and patient education, or integration of HIV and diabetes services.

## P199

### Metabolic disorders in HIV‐infected patients receiving antiretroviral therapy

**V Kanestri and A Kravchenko**

Federal AIDS Center, Central Scientific Research Institute of Epidemiology of Rospotrebnadzor, Moscow, Russian Federation

**Background: **Evaluation of lipid and carbohydrate metabolism in HIV‐infected patients depending on ART and its duration.

**Materials and methods: **Analysis of lipid and carbohydrate metabolism in 229 adult HIV‐infected patients (44 patients without ART, 29 patients receiving ART for less than one year and 156 patients receiving ART during period of three to nine years) was conducted. The following changes in body composition and laboratory parameters in fasted state were assessed: total cholesterol (TC), triglycerides (TG), high‐density lipoprotein (HDL), low‐density lipoprotein (LDL), very low‐density lipoprotein (VLDL), apolipoprotein B (Apo B), atherogenic index of plasma (AIP) levels, as well as plasma levels of glucose, insulin and glycated haemoglobin A1c (HbA1c).

**Results: **During the first year, ART exacerbates lipid disorders caused by HIV, particularly affecting TG and HDL levels. During the treatment, partial adaptation of patients to antiretroviral drugs contributing to improvement of these parameters was observed. In addition to that, elevation in TC, LDL, VLDL levels can lead to AIP increase. Increased levels of TG, VLDL, AIP, and decreased levels of HDL are observed significantly more frequently in men in comparison with women. Administration of efavirenz (EFV) contributed to reduction of HDL levels and to increase in AIP, and the use of lopinavir/ritonavir (LPV/r) caused elevation of TG and VLDL levels. Smoking influences, albeit to a lesser extent, on increase in VLDL, Apo B concentrations, AIP, as well as on decrease in HDL concentrations. When using ART, the disorders of carbohydrate metabolism were substantially less frequent (8 to 10%), and in combination with other risk factors led to development of diabetes mellitus in 1.6% of patients. Long‐term ART, male gender and smoking increase significantly the risk of lipid disorders (Table 1).

**Table:** Abstract P199 – Table 1. Influence of risk factors on lipid metabolism in patients with HIV infection (OR)

Lipid metabolism parameters (OR [95% CI]) / risk factors	ART >three years	EFV	LPV/r	Male gender	Smoking
Increase in TC level	7.39 (6.47 to 8.31)	No influence	No influence	No influence	No influence
Increase in TG level	7.24 (6.02 to 8.46)	No influence	3.8 (3.03 to 4.57)	2.4 (1.33 to 3.47)	No influence
Decrease in HDL level	No influence	2.01 (0.88 to 3.14)	No influence	1.55 (0.11 to 2.99)	1.56 (0.85 to 2.27)
Increase in LDL level	1.88 (1.18 to 2.58)	No influence	No influence	No influence	No influence
Increase in VLDL level	3.26 (1.16 to 5.36)	No influence	3.53 (2.12 to 4.94)	5.51 (3.41 to 7.61)	3.46 (2.26 to 4.66)
Increase in AIP	1.57 (0.9 to 2.24)	2.26 (1.41 to 3.11)	No influence	1.84 (1 to 2.68)	1.67 (0.97 to 2.37)
Increase in Apo B level	No influence	No influence	No influence	No influence	6.64 (4.47 to 8.81)

**Conclusions: **It is necessary to choose ART scheme with the least impact on lipid levels, and to advise the patients to give up smoking. In case of significant abnormalities in the course of therapy, the regime of ART can be corrected and/or lipid‐lowering medicinal products can be administered. Routine screening of the patients is important for timely diagnostics of impaired glucose tolerance, diabetes mellitus, insulin resistance, and for conduction of corrective measures.

**References**

[1] Calza L, Manfredi R, Chiodo F. Hyperlipidemia in patients with HIV‐1 infection receiving highly active antiretroviral therapy: epidemiology, pathogenesis, clinical course and management. Int J Antimicrob Agents. 2003;22:89‐99.

[2] Dube M, Fenton M. Lipid abnormalities. Clin Infect Dis. 2003;36 Suppl 2:79‐83.

[3] Gelato M. Insulin and carbohydrate dysregulation. Clin Infect Dis. 2003;36 Suppl 2:91‐5.

[4] Data Collection on Adverse Events of Anti‐HIV drugs (D:A:D) Study Group. Factors associated with specific causes of death amongst HIV‐positive individuals in the D:A:D study. AIDS. 2010;24:1537‐48.

## COMORBIDITIES AND COMPLICATIONS OF DISEASE AND/OR TREATMENT: NEUROLOGICAL

## P200

### Factors associated with HIV‐associated neurocognitive disorder in an unselected cohort in East South London: the HAND study

**S Rackstraw^1^, O Davies^2^, A Thiyagarajan^1^, A Sharp^2^, N Patel^2^, R Szydlo^3^ and R Kulasegaram^2^**

^1^HIV, Barts Health NHS Trust, London, UK. ^2^HIV, Guy's & St Thomas’ Hospitals NHS Trust, London, UK. ^3^Statistics, Imperial College London, London, UK

**Background: **UK studies have shown low levels of neurocognitive impairment (NCI) in selected cohorts. Several clinical factors including ART CNS penetration effectiveness (CPE) score have been linked with HIV‐associated neurocognitive disorder (HAND). In the first study of HAND in an unselected cohort in London, we aimed to: (1) determine the extent of NCI in this cohort; (2) establish correlation with HIV‐related factors and medical comorbidities.

**Methods: **Seven hundred and eighty‐six HIV+ participants aged >18 were prospectively recruited from four HIV clinics in East and South London. Medical and ART history and mental state assessment were completed. Computerised assessment of neurocognitive function was performed using Cogstate tests. Participants had NCI if they were >1 standard deviation (SD) outside of the population mean in two or more cognitive domains.

**Results: **The median age was 46. Sixty‐five percent were Caucasian. Eighty‐one percent had HIV VL <100. Median CD4 count was 566 cells/µL. Of the 710 who completed the computerised Cogstate tests, 84% were men. 37.2% had NCI. Table 1 demonstrates the factors found to be independently associated with HAND on multivariate analysis when adjusted for ART drug regimen.

**Table:** Abstract P200 – Table 1. Factors independently associated with HAND

Variable	n	Odds ratio	*p* value
Anxiety	Normal	354	1.00	
	Borderline	143	1.41	0.15
	Abnormal	193	2.47	0.0001
Summarised IHDS	Normal	553	1.00	
	Abnormal	137	3.90	0.0001
Race	Black African	129	1.00	
	Black Caribbean	32	0.88	0.78
	Caucasian	475	0.22	0.0001
	Other	22	0.31	0.041
Education	College/university	458	1.00	
	Other	232	1.73	0.004
Transmission route	MSM	446	1.00	
	Other	244	1.96	0.003
CPE score	0 to 5	42	1.00	
	6 to 8	530	1.35	0.47
	>8	35	3.22	0.037

**Conclusion: **NCI was observed in 37.2% of this cohort. Anxiety, abnormal IHDS, being black African, below college level education, non‐MSM transmission route and CPE score >8 were associated with HAND. The CPE score effect is at variance with that observed in other studies. This could be due to intensification of ART in those with prior NCI or even a degree of drug toxicity. More research is needed on the effects of ART on HAND.

## P201

### Efficacy of a computerised cognitive rehabilitation training in improving HIV‐associated neurocognitive disorders

**F Bai^1^, M Allegrini^1^, C Falcinella^1^, I Strada^2^, L Borghi^2^, E Merlini^1^, A d'Arminio Monforte^1^ and G Marchetti^1^**

^1^Dept Health Sciences/Clinic of Infectious Diseases, San Paolo Hospital, University of Milan, Milan, Italy. ^2^Dept Health Sciences/Unit of Clinical Psychology, San Paolo Hospital, University of Milan, Milan, Italy

**Background: **We aimed to investigate the efficacy of a new computer‐based cognitive rehabilitation protocol (restorative approach) in improving the cognitive performance of patients affected by HIV‐associated neurocognitive disorders (HAND).

**Materials and methods:** ****Pilot unblinded randomised controlled trial (parallel allocation 1:1) enrolling HIV‐infected patients on combination antiretroviral treatment (cART) in follow‐up at the San Paolo Infectious Diseases (SPID) Cohort, Milan, Italy. At screening, patients underwent a neuropsychological battery (11 tests, seven cognitive domains plus assessment of mental health and quality of life by Beck Anxiety Inventory, Beck Depression Inventory and Medical Outcome Study HIV Health Survey) to diagnose HAND (Frascati criteria). Patients diagnosed with HAND and meeting the eligibility criteria (exclusion criteria: AIDS‐defining illnesses, not adequately managed depression, neurological/psychiatric comorbidities, active alcohol/substance abuse, cirrhosis/severe comorbidities requiring hospitalisation, not comprehension of Italian language, detectable HIV‐RNA for cART‐treated patients) were randomly allocated to continue cART (control group) or to the cognitive rehabilitation training in association with cART (intervention group: ERICA software, 12 weekly sessions of nine computer‐based exercises lasting one hour under the supervision of a physician aimed at improving five cognitive domains). After completion of the protocol (t12), the intervention group was reassessed by the same neuropsychological battery; both control and intervention groups were also planned to be tested at Week 24. In the intervention group, repeated measures ANOVA was used to compare pre‐ and post‐rehabilitation mean T scores on each cognitive domain.

**Results: **We screened 55 patients; 28 patients with HAND were randomised (14 intervention group, 14 control group). The two groups did not differ at randomisation. The raw scores obtained at the neuropsychological tests are corrected for age, educational level and gender and converted to normative T scores. In the intervention group, four patients declined and three patients were lost to follow‐up; seven patients completed the cognitive rehabilitation programme. At t12, the proportion of HAND has declined with no cognitive impairment in 2/7 (28%) patients. The mean T scores in two cognitive domains (attention/working memory and abstraction/executive functions), and five neuropsychological tests (Digit Span Test‐Backward, Rey Auditory Verbal Learning Test‐Delayed Recall, Rey‐Osterrieth Complex Figure Test‐Delayed Recall, Stroop Color and Word Test‐Errors and Phonemic Fluency Task), significantly improved from baseline to t12.

**Abstract P201 – Figure 1.** Mean T scores for each cognitive domain at t0 (screening) and t12 (after 12 weeks of rehabilitation training) in the intervention group.

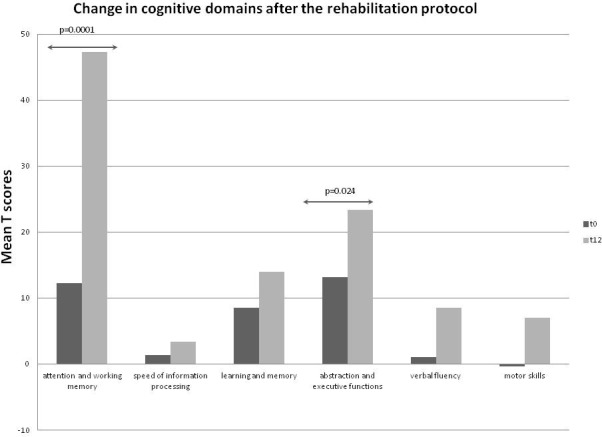

**Conclusions: **While not recovering HAND, 12 weeks of this new computer‐based rehabilitation protocol was effective in improving specific cognitive domains.

## P202

### The effect of drug use on specific neuropsychological domains among patients enrolled in the Neurocognitive Assessment in the Metabolic and Aging COhort (NAMACO) study

**K Darling^1^, I Locatelli^2^, N Benghalem^1^, I Nadin^3^, P Brugger^4^, K Gutbrod^5^, S Frueh^6^, S Rossi^7^, U Kunze^8^, T Lecompte^9^, C Hauser^10^, B Hasse^11^, H Kovari^11^, P Tarr^12^, M Stoeckle^13^, C Di Benedetto^14^, P Schmid^15^, R Du Pasquier^16^ and M Cavassini^1^**

^1^Infectious Diseases Service, Lausanne University Hospital, Lausanne, Switzerland. ^2^Division of Biostatistics and Quantitative Methods, Lausanne University Hospital, Lausanne, Switzerland. ^3^Department of Clinical Neurosciences, Lausanne University Hospital, Lausanne, Switzerland. ^4^Department of Neurology, University Hospital Zürich, Zürich, Switzerland. ^5^Department of Neurology, Inselspital Bern, Bern, Switzerland. ^6^Department of Neurology, Kantonsspital St. Gallen, St. Gallen, Switzerland. ^7^Unità di Neuropsicologia e Logopedia, Ospedale Civico Lugano, Lugano, Switzerland. ^8^Memory Clinic, Felix Platter Hospital, Basel, Switzerland. ^9^Infectious Diseases Division, Hôpitaux Universitaires de Genève, Geneva, Switzerland. ^10^Department of Infectious Diseases, Bern University Hospital, Bern, Switzerland. ^11^Department of Infectious Diseases, Universitätsspital Zurich, Zurich, Switzerland. ^12^University Department of Medicine, Kantonsspital Bruderholz, Bruderholz, Switzerland. ^13^Infectious Diseases, University Hospital Basel, Basel, Switzerland. ^14^Infectious Diseases, Ospedale Regionale di Lugano, Lugano, Switzerland. ^15^Infectious Diseases, Kantonsspital St. Gallen, St. Gallen, Switzerland. ^16^Department of Clinical Neurosciences, Lausanne University Hospital, Lausanne, Switzerland

**Background: **HIV‐associated neurocognitive impairment (NCI) is diagnosed based on neuropsychological (NP) testing and functional assessment, after excluding non‐HIV‐associated confounders. The effect of active or previous drug use on the development of NCI among people living with HIV has been incompletely characterised. The aim of this study was to examine the influence of drugs on NP tests in a large cohort of HIV‐positive patients with well‐controlled infection.

**Methods: **We analysed patients enrolled in the Neurocognitive Assessment in the Metabolic and Aging COhort (NAMACO) study, an ongoing, prospective, longitudinal, multicentre and multilingual study within the Swiss HIV Cohort Study (SHCS). NAMACO patients had to be aged ≥45 years old and speak a national language (French, German, Italian) to participate, and all underwent baseline NP tests. Drug use data, which are collected twice yearly at clinic visits, were obtained from the SHCS database. We defined binary (presence/absence of impairment) and continuous (mean z‐score) outcomes for NCI and five specific NP domains: motor skills, speed of information processing, executive function, attention/working memory and verbal learning/memory. The effects of previous injecting drug use (IDU) (heroin) and current non‐IDU (cocaine, cannabis) were examined with uni‐ and multivariable logistic and linear regression models adjusted for age, sex, ethnicity, education level and alcohol consumption.

**Results: **Among 981 included patients, mean age was 54.5 years (SD 7.5); 782 patients (79.7%) were male, 899 (91.6%) were Caucasian and 942 (96.2%) had undetectable viral loads (<50 copies/mL). Previous IDU was reported in 137 patients (14%) and current non‐IDU in 108 patients (11%). Five patients reported current IDU (heroin +/‐ cocaine) and were excluded from further analysis. Previous IDU did not affect NCI overall but was associated with effects on specific NP domains: a protective effect on attention/working memory (significant for binary outcomes: adjusted OR [aOR] 0.63, 95% CI 0.40 to 0.97, *p *=* *0.04, but not for continuous outcomes: mean z‐score difference 0.10, 95% CI −0.05 to 0.24), and a negative effect on speed of information processing (significant for continuous outcomes: mean z‐score difference −0.19, 95% CI −0.35 to −0.02, *p *=* *0.02, but not for binary outcomes: aOR 1.13 95% CI 0.73 to 1.73). We observed no effect of current non‐IDU on NCI overall or on specific NP domains.

**Conclusions: **In this population of HIV‐positive persons well controlled on antiretroviral therapy, we observe no effect of previous IDU or current non‐IDU on NCI overall but an effect of previous IDU on specific NP domains.

## P203

### A real‐life analysis of dolutegravir adverse effects in a cohort of naïve and experienced HIV‐infected patients accessing the Infectious Diseases Clinic of Modena

**M Digaetano^1^, C Monari^2^, C Rogati^1^, M Menozzi^1^, A Santoro^1^, A Bonazza^1^, F Carli^1^, V Borghi^1^ and C Mussini^1^**

^1^Clinica di Malattie Infettive, Policlinico di Modena, Modena, Italy. ^2^Department of Mental Health and Public Medicine, University of Campania L. Vanvitelli, Napoli, Italy

**Background: **Increasing interest in dolutegravir (DTG) is reported, thanks to its high potency and efficacy, high barrier‐to‐resistance development, low risk of drug‐to‐drug interactions. Nevertheless, several authors arise concerns around DTG tolerance and safety profile in real‐life settings, due to the onset of neuropsychiatric adverse effects (NAEs), i.e. sleep disturbances, headache, nervousness, poor concentration and unexplained pain or paraesthesia. Aim of this study was to analyse the most frequent AEs leading to DTG discontinuation in our cohort.

**Material and methods**

****We performed a retrospective analysis of a cohort of 632 HIV‐infected patients who started DTG between January 2015 and March 2018. Data were obtained from the electronic outpatients chart of our clinic. We focused on the main AEs responsible for DTG withdrawal.

**Results: **Forty‐four out of 632 patients (7%) discontinued DTG because of AEs. Among them 26 were males (59%), the average age was 49.1 years. Nine patients (12.7%) were naïve to ART and seven of them started a TDF/TAF backbone. Experienced patients discontinued dual therapy (mostly with 3TC) in 15 cases and triple ART in 20 cases (12 with an ABC and eight with a TAF/TDF backbone). Most patients who discontinued DTG complained NAEs (32 subjects, 5% of the total DTG cohort). Other reported AEs are listed in Table 1. Among people suffering of NAEs (see Table 2), five patients were naïve to ART (two ABC backbone) while 27 were experienced. In the latter group, 13 subjects interrupted dual therapy and 14 a triple ART (seven ABC and seven TDF/TAF). Moreover, 30 out of patients who experienced NAEs (93.7%) did not report them after ART switch.

**Table:** Abstract P203 – Table 2. DTG discontinuation due to NAEs

	n	%
Discontinuations due to neuropsychiatric AEs	32	5
Naive	5	7
ABC backbone/n tot ABC	2	0.9
TDF/TAF backbone/n tot tenofovir	3	2.6
Experienced	27	4.8
Dual	13	
Triple	14	
ABC backbone/n tot ABC	7	3
TDF/TAF backbone/n tot tenofovir	7	6.1
Previous neuropsychiatric disorders or psychiatric drugs	8	25
Resolution after ART modification	30	93.7

**Conclusions:** ****This study shows a rate of DTG interruption due to AEs (7%) similar to the literature reported ones (ranging from 5.4 to 13.7%). We also obtain similar results in appearance of NAEs (5 vs. 5.4%). In our cohort, naïve rate of DTG suspension due to AEs is 12.7%, lower to a literature rate ranging from 16.8 to 17.6%. Comparing the cases of suspension between naive and experienced, we note, as reported in literature, a higher rate of discontinuation of the therapy among the first ones (12.7% vs. 6.2%). Regarding accompanying drugs, a higher rate of suspensions was observed in the cohort taking TDF/TAF versus ABC backbone (13% vs. 6%). This data, not in line with the literature, will have to be verified with further analysis and wider populations.

## COMORBIDITIES AND COMPLICATIONS OF DISEASE AND/OR TREATMENT: RENAL

## P204

### Real‐world experience using tenofovir alafenamide fumarate in Glasgow, UK

**K Brown^1^, G Sutherland^2^, R O'Hara^1^ and D Bell^3^**

^1^Pharmacy, NHS Greater Glasgow and Clyde, Glasgow, UK. ^2^Medicine, University of Glasgow, Glasgow, UK. ^3^Infectious Diseases, NHS Greater Glasgow and Clyde, Glasgow, UK

**Background: **Tenofovir alafenamide fumarate (TAF) and tenofovir disoproxil fumarate (TDF) are pro‐drugs of tenofovir, and used in the treatment of HIV infection. In clinical trials TAF demonstrated improvement in laboratory markers of renal and bone safety compared to TDF. Since 2015, European marketing authorisation has been granted for four TAF‐containing products for HIV: Genvoya, Descovy, Odefsey and Symtuza. Generic versions of TDF are available in the UK, alone and in combination with other ARVs. These are cheaper than TAF‐containing products. In 2017 the Scottish HIV Clinical Leads published cost‐sensitive prescribing recommendations for first‐line ARVs taking account the costs of medication at that time [1]. These recommended a TAF‐containing regimen for patients with established or significant risk factors for bone or renal problems and who were unsuitable for abacavir.

**Materials and methods: **We identified all patients who had started a TAF‐containing regimen since 2015 to May 2018 from our clinic database. Clinical records were used to determine the reason for the choice of treatment, whether this was a first treatment or a switch, clinical parameters including comorbidities and to compare laboratory renal parameters before and after switch.

**Results: **One thousand seven hundred and twenty‐one patients were on ARVs in May 2018, and of these, 272 (16%) were on a TAF‐containing regimen: 56% took Genvoya, 30% Descovy, 13% Odefsey and 1% were on Symtuza. Two hundred and fifty‐four (93%) were treatment switches and 18 (7%) were on their first ARV treatment. For 254 switch patients, 192 had switched from TDF and 62 patients from non‐TDF containing regimens. The reasons documented for these switches to a TAF‐containing regimen were: 48%, established renal dysfunction or fragility fracture / osteoporosis; 12%, as a cost saving (the price of Stribild is greater than Genvoya); 11%, to reduce pill burden; 8%, risk factors for renal dysfunction present but no current renal abnormalities; 8%, side effects of current medication; 12%, no reason given / other.

**Conclusions: **Sixteen percent of patients in the Glasgow HIV cohort are now prescribed a TAF‐containing regimen; 93% were switched onto TAF from an alternative treatment, mostly from TDF. For 45% of patients receiving TAF, we found no evidence of current renal or bone problems, or clear risk factors for future renal or bone disease, treatment criteria suggested by a national prescribing guideline. Given the wide availability of generic alternatives, further cost savings could be made after clinical review and patient discussion.

**Reference: **[1] Scottish Health Protection Network HIV Clinical Leads. Guidance for cost‐sensitive HIV therapy prescribing in NHS Scotland 2017 [Internet]. 2017. Available from: http://www.hivscotland.com/downloads/1495095774‐Scotland%20HIV%20cost‐sensitive%20prescribing%20FINAL.pdf.

## P205

### Incidence rate of substitutions due to tenofovir disoproxil fumarate‐related bone and renal toxicity among adults attending the Infectious Diseases Institute Kampala

**J Akirana, E Laker, R Muyise, N Owarwo, I Lwanga, F Mubiru, B Castelnuovo, A Kiragga and M Lamorde**

HIV Prevention, Care and Treatment Department, Infectious Diseases Institute, Kampala, Uganda

**Background: **Lifelong antiretroviral therapy may lead to toxicities that increase patient morbidity and may prove costly to the health system. Drug substitutions are a recognised strategy for managing antiretroviral toxicities. The World Health Organization (WHO) recommends tenofovir disoproxil fumarate (TDF)‐based regimens as preferred first‐line regimens but TDF is associated with bone and renal toxicity and data are scarce on these toxicities in sub‐Saharan African countries [1,2]. We report the incidence rate and factors associated with TDF‐based regimen substitutions due to toxicities among adults attending an HIV clinic in Uganda.

**Materials and methods: **A retrospective case‐control analysis was conducted at the Infectious Diseases Clinic in Uganda between 1 January 2005 and 31 December 2017. Adults who had had a substitution to their TDF‐based regimen due to TDF bone and renal toxicity were included as cases. Controls were randomly selected (1:3) and frequency matched to case patients by age, gender and duration on TDF‐based regimen. We determined the incidence rate and summarised patient demographics using medians, frequencies and percentages.  Patient characteristics were compared using chi‐square tests and Wilcoxon signed rank tests. Logistic regression was performed to explore associations between baseline covariates and the odds of substitution of TDF due to bone and renal toxicities.

**Results: **From 24 to 60 months of TDF exposure, incidence rate was <4 per 10,000 per‐person‐years (ppy) but this increased to >9 per 10,000 ppy after 72 months of exposure. Compared to the controls, in univariate analyses, cases were more likely to have advanced disease (WHO stage III to IV) and have lower CD4 counts but other baseline characteristics were similar (Table 1). In multivariate analyses, patients at WHO stage III and IV at ART initiation had greater odds of switching from TDF‐based regimen due to toxicity compared to those who were in WHO stage I and II (OR 2.12, 95% CI 1.25 to 3.55, *p *=* *0.004). Higher CD4 at ART start was protective of switching (OR 0.94, 95% CI 0.89 to 0.98, *p *=* *0.017). However, we did not find any association between odds of substitution and other baseline characteristics (Table 1).

**Table:** Abstract P205 – Table 1. Patient characteristics at baseline

Characteristics	Cases (n = 113)	Controls (n = 339)	*p* value
Male sex, %	42.48	44.31	0.736
Age, median (IQR)	42.1 (35.00 to 51.20)	41.50 (34.8 to 49.10)	0.57
BMI, %			0.584
Underweight (<18.5)	20.19	18.95	
Normal (18.5 to 24.9)	60.58	56.86	
Overweight (>25)	19.23	24.18	
WHO stage, %			<0.0005
I to II	42.48	61.85	
III to IV	57.52	38.15	
CD4 count, median (range)	166 (41 to 352)	238 (87 to 462)	0.005
ART duration before exposure to TDF	0.5 (0 to 36.60)	0 (0 to 34.30)	0.22
ART duration on TDF	43.15 (10.82 to 96.69)	51.51 (17.71 to 84.66)	0.20
History of TB, %	10.62	7.38	0.22
Hypertension, %	6.19	11.69	0.10
Diabetes, %	1.77	2.46	0.67
NSAIDS use, %	46.02	46.46	0.94
History of renal disease, %	0.00	0.62	0.400

**Conclusion: **Incidence of toxicities increased more than two‐fold after five years of TDF exposure. Furthermore, patients with advanced disease had greater odds of a switch for toxicity. Clinicians should remain vigilant for TDF toxicity in otherwise stable patients on TDF‐based regimens and among patients with advanced disease.

**References**

[1] Dadi TL, Kefale AT, Mega TA, Kedir MS, Addo HA, Biru TT. Efficacy and tolerability of tenofovir disoproxil fumarate based regimen as compared to zidovudine based regimens: a systematic review and meta‐analysis. AIDS Res Treat. 2017;2017:5792925.

[2] World Health Organization. Consolidated guidelines on the use of antiretroviral drugs for treating and preventing HIV infection: recommendations for a public health approach. 2nd ed. Geneva: World Health Organization; 2016.

## P206

### Real‐world effects of treatment with emtricitabine/tenofovir alafenamide‐ versus emtricitabine/tenofovir disoproxil fumarate‐based regimens in people living with HIV in a clinical cohort in Germany

**A Rieke^1^, H Jessen^2^, R Pauli^3^, M Waizmann^4^, T Heuchel^5^, N Postel^6^, C Lymperopoulou^7^, I Faghmous^8^, H Diaz‐Cuervo^9^, H Ramroth^8^ and H Stellbrink^10^**

^1^Gemeinschaftsklinikum Mittelrhein, Kemperhof, Germany. ^2^Gemeinschaftspraxis, Jessen, Berlin, Germany. ^3^Gemeinschaftspraxis, Becker/Pauli, Munich, Germany. ^4^Praxis, Waizmann, Leipzig, Germany. ^5^Praxis, Heuchel, Chemnitz, Germany. ^6^Prinzmed, Private Praxis, Munich, Germany. ^7^School of Public Health, Imperial College London, London, UK. ^8^Epidemiology, Gilead Sciences, London, UK. ^9^Global Health Economics and Outcomes Research, Gilead Sciences, London, UK. ^10^ICH Infectious Diseases Center, Hamburg, Germany

**Background: **Treatment with tenofovir disoproxil fumarate (TDF) may be associated with impairment of renal function in HIV‐1 infected patients and may cause proximal renal tubular abnormalities and/or decrease of estimated glomerular filtration rate (eGFR). A cohort study was initiated to evaluate the effectiveness and safety of emtricitabine/tenofovir alafenamide (F/TAF)‐based regimens in HIV‐1 infected patients since 2016. The present analysis evaluated the effects of elvitegravir/cobicistat/F/TAF (E/C/F/TAF) on renal function among the patients switched from elvitegravir/cobicistat/emtricitabine/TDF (E/C/F/TDF).

**Materials and methods: **TAFNES is an ongoing 24‐month prospective, observational clinical cohort of HIV patients initiating treatment with F/TAF‐based regimens in routine care in Germany. Patients switched from E/C/F/TDF (with a minimum usage of 30 days) to E/C/F/TAF and with recorded baseline and 12‐month renal parameters in an interim data cut dated 1 June 2018 were included in the present analysis. Selection of E/C/F/TAF was based on clinician discretion and was in accordance with SMPC guidance. eGFR was calculated using CKD‐EPI equation. Change over time and evolution of renal function stage was evaluated by Wilcoxon signed‐rank test.

**Results: **Of the 87 patients in the cohort who switched from E/C/F/TDF to E/C/F/TAF, 66 (75.9%) had registered renal parameters at the moment of switch and after 12 months and thus were included in this analysis; 93.9% were male and median age was 41 (IQR 34 to 53). 31.8% were ≥50 years. Median previous E/C/F/TDF usage duration was 17.1 months (IQR 6.5 to 32.3). Median eGFR at switch was 86.1 mL/min/1.73 m^2^ (95% CI 77.0 to 95.2) and increased to 95.3 mL/min/1.73 m^2^ at Month 12 (*p *=* *0.034; 95% CI 85.7 to 104.9), accounting for a median 10.1% increase (Figure 1). The percentage of patients with eGFR ≥90 mL/min/1.73 m^2^ increased from 42.4% at switch to 63.6% at Month 12 whereas percentage of those with 60 to 90 mL/min/1.73 m^2^ decreased from 48.5% to 33.3% (increase from 90.9% to 97% in patients with eGFR ≥60 mL/min/1.73 m^2^) and percentage of those with 30 to 60 mL/min/1.73 m^2^ decreased from 9.1% to 3.0%. No patients with eGFR <30 mL/min/1.73 m^2^ were observed at either point (Figure 2).

**Abstract P206 – Figure 1.** Estimated glomerular filtration rate (mL/min/1.73 m^2^) at switch and Month 12.

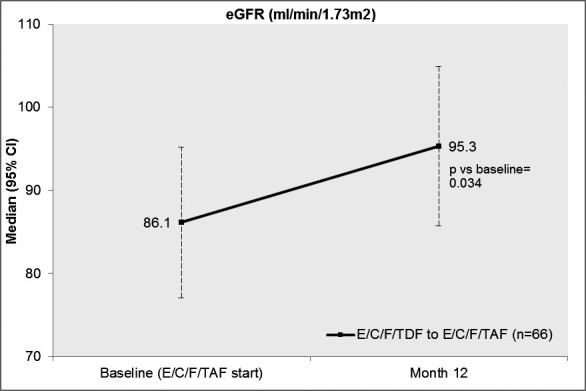

**Abstract P206 – Figure 2.** Change in distribution of estimated glomerular filtration categories from switch to Month 12.

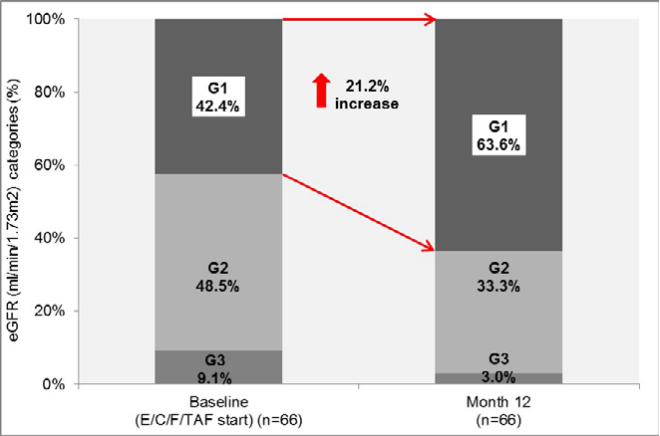

Estimated glomerular filtration rate categories: G1: Normal or high, eGFR ≥90; G2: Mildly decreased, 60 ≤  eGFR < 90; G3: Mildly to moderately or moderately to severely decreased, 30 ≤  eGFR < 60.

**Conclusions: **This clinical cohort demonstrated an improvement in renal parameters in patients switching from E/C/F/TDF to E/C/F/TAF, with a 21.2% increase in patients with normal renal function (eGFR ≥90) from switch to Month 12.

## P207

### Reclassification of severe renal failure in people living with HIV using modified KDIGO classification

**G Manmathan^1^, R Ramphul^2^, M Johnson^1^, R Rakhit^3^ and F Burns^1^**

^1^HIV Medicine, Royal Free London, London, UK. ^2^Renal Medicine, Croydon University Hospital, London, UK. ^3^Cardiology, Royal Free London, London, UK

**Background: **Chronic kidney disease (CKD) has emerged as an important health concern for PLWHIV. Preventing long‐term kidney toxicity from an antiretroviral therapy is therefore critical [1]. Clinicians can use several monitoring tools, including the Kidney Disease Improving Global Outcomes (KDIGO) risk score and routine measurements of estimated glomerular filtration (eGFR) and proteinuria, to identify high‐risk individuals who may require an intervention [2].

**Materials and methods: **During a 1‐week period of PLWHIV attending the HIV clinic at the Royal Free Hospital we analysed the results of 104 people. Ninety‐four patients had a PCR performed. We then re‐categorised them according to the modified KDIGO 2012 classification, replacing albumin creatinine ratio (ACR) with protein creatinine ratio (PCR) (Grading of proteinuria. adapted from Table 7. KDIGO 2012 guidelines). We also aimed to see if there was a correlation between high blood pressure and proteinuria.

**Results: **We had 25 (26.5%) females and 69 (73.5%) males with an average age of 49.5 years. When grading severity of proteinuria we had 68 (72.3%) patients with normal function, 21 (22.3%) with mild and five (5.3%) with severe. Comparing proteinuria and high blood pressure (>140/90 mmHg) there was not significant difference between the groups (Table 1.)

**Table:** Abstract P207 – Table 1. Distribution on PCR and high blood pressure

PCR	mg/mmol	N (%)	High BP	%
Normal	<15	68 (72.3)	32	47.8
Mild	15 to 50	21 (22.4)	7	33.3
Severe	>50	5 (5.3)	1	20.0
	Total	94	40	

So using the modified KDIGO classification (Table 2) we had a prevalance rate of low 69.1%, moderate 18.1%, high 11.7%, very high 1.1%. Compared to a Japanese cohort we have higher attrition rate of CKD in our HIV population [3]. They analysed 1447 PLWHIV (97% male; 3% female), with an average age of 44.4 years, and had prevalance rates of low 85.9%, moderate 11.0%, high 2.1% and very high 1.0%. So although the groups demographics are slightly different we still believe there are further multifactorial causes for the higher prevalence of more severe renal disease, a reason for the higher prevalence of CKD between the populations is likely due to the eGFR not accounting for Afro‐Caribbean ethnicity.

**Table:** Abstract P207 – Table 2. Distribution of PLWHIV determined by the modified KDIGO 12 classification

CKD grading	eGFR (mL/min)	P1 (%)	P2 (%)	P3 (%)	Total (%)
G1	>90	28.7	9.6	1.1	39.4
G2	60 to 90	40.4	7.4	4.3	52.1
G3a	45 to 59	1.1	5.3	0.0	6.4
G3b	30 to 44	1.1	0.0	1.1	2.1
G4	15 to 29	0.0	0.0	0.0	0.0
G5	<15	0.0	0.0	0.0	0.0
Total		71.3	22.3	6.4	

**Conclusions: **In a subgroup analysis the presence of proteinuria did not correlate with high blood pressure. Using this method we were able to reclassify those who were very high risk who would require intervention and reduce this number; however, there were more PLWHIV at high and moderate risk who would require closer monitoring. Compared to a Japanese cohort we have higher attrition rate of CKD in our HIV population [3]. We would propose to analyse our patients using a large number and account for ethnicity and re‐compare our data.

**References**

[1] Achhra AC, Nugent M, Mocroft A, Ryom L, Wyatt CM. Chronic kidney disease and antiretroviral therapy in HIV‐positive individuals: recent developments. Curr HIV/AIDS Rep. 2016;13:149‐57.

[2] Kidney Disease Improving Global Outcomes. KDIGO 2012 clinical practice guideline for the evaluation and management of chronic kidney disease. Kidney Int Suppl. 2013;3:1‐150.

[3] Yanagisawa N, Muramatsu T, Yamamoto Y, Tsuchiya K, Nitta K, Ajisawa A, et al. Classification of human immunodeficiency virus‐infected patients with chronic kidney disease using a combination of proteinuria and estimated glomerular filtration rate. Clin Exp Nephrol. 2014;18:600‐5.

## P208

### Evolution of renal function in patients starting simultaneous therapy with DRV/c and DTG

**F Roque Rojas, S de la Fuente Moral, A Díaz de Santiago, C Lavilla Salgado, A Muñoz Serrano and A Ángel‐Moreno Maroto**

Servicio de Medicina Interna, Hospital Universitario Puerta de Hierro Majadahonda, Majadahonda, Spain

**Introduction and objective**

Double therapy with DRV/c and DTG is an attractive option of ART in some situations, due to its high genetic barrier, its simplicity and the absence of nucleoside analogue RTIs. Patients on ART become progressively older and thus present comorbidities more frequently. Many of them suffer some degree of renal insufficiency or risk factors for its development. DTG and DRV/c inhibit creatinine tubular secretion at several sites, which results in increased serum values and the subsequent decrease in estimated glomerular filtration rate (eGFR) as calculated by traditional methods. It remains unknown if this is an additive effect. The goal of this study was to evaluate changes in renal function in patients starting simultaneous therapy with DRV/c and DTG.

**Method: **We designed a retrospective study including all patients being treated with DRV/c‐DTG. Other drugs are allowed to be included in the regimen. Serum creatinine and eGFR (via CKD‐EPI) before initiating the drug combination are compared with values at the first follow‐up and at 24 weeks after initiation. The ART regimen immediately prior to the change is also registered, as well as the presence or absence of TDF. 

**Results: **Thirty‐six patients (mean age 49 years, 61% male, 50% former IV drug users, 25% MSM, 19% heterosexuals) were included, with a mean time of life with HIV of 19.7 years and a mean time on ART of 13.5 years. Sixty‐four percent of patients started DTG and DRV/c as a simplification of more complex regimens or in order to avoid toxicity, and 26% started them following virological failure or low‐level viraemia. An increase of 0.06 mg/dL (6.45%) in creatinine serum levels is observed after initiating DRV/c‐DTG (*p *=* *0.03). Parallel to this, a decrease of 4.05 mL/min/1.73 m² in eGFR is seen, but it does not prove to be statistically significant (*p *=* *0.06). Changes in renal function were not significant in later determinations, and they were similar to the ones observed in two previous series of ours: one including patients initiating DRV/c as a switch from DRV/r and another one including naïve patients who start therapy with DTG.

**Conclusion: **Patients receiving combined treatment with DRV/c and DTG show an initial increase in serum creatinine levels, with a subsequent decrease in estimated glomerular filtration rate. None of them prove to be progressive, and they are similar to the effects previously described separately with DRV/c and DTG.

## P209

### Changes in proximal tubular function after early discontinuation of tenofovir disoproxil fumarate (TDF) in HIV patients with TDF‐induced renal dysfunction

**P Payoong^1^, A Leelahavanichkul^2^, O Putcharoen^1^**

^1^Department of Medicine, Division of Infectious Diseases, Chulalongkorn University, Faculty of Medicine, Bangkok, Thailand. ^2^Department of Microbiology, Division of Immunology, Chulalongkorn University, Faculty of Medicine, Bangkok, Thailand

**Background: **Tenofovir (TDF) is the first‐line NRTI for treatment of HIV‐1 infection in Thailand. TDF causes a decline in renal function and proximal tubular dysfunction. The Thai guideline recommends stopping TDF in patients with eGFR decline greater than 25% from the baseline. Early discontinuation of TDF might preserve long‐term renal function.

**Materials and methods: **A prospective, controlled study was carried out in HIV‐1 infected patients at King Chulalongkorn Memorial Hospital, Bangkok, Thailand from 1 September 2017 to 31 March 2018. Patients with TDF‐induced nephropathy were switched the regimen to either to abacavir, lamivudine and efavirenz (ABC + 3TC + EFV) or lopinavir/ritonavir plus lamivudine (LPV/r + 3TC). Early discontinuation was applied in patients with proximal renal tubular dysfunction (PRTD) that was defined as the presence of ≥2 abnormalities in renal proximal tubular function; phosphaturia, non‐diabetic glycosuria, hyperuricosuria, proteinuria and/or increased urinary beta‐2 microglobulin or a decline in eGFR of 10 to 25% from baseline. The other group was made up of patients that continued TDF until eGFR declined to greater than 25% (standard treatment arm). The changes in proximal tubular function between these two arms were compared.

**Results: **A total of 26 patients were included in this study. Fifteen patients were in the early discontinuation arm and 11 patients were in the standard treatment arm (four patients were in the standard discontinuation and seven patients were in the continuation group). Thirteen patients were switched to ABC + 3TC + EFV and six patients were switched to LPV/r + 3TC. The median durations of TDF treatment were 73.46 (±23.14) months in the early discontinuation arm and 85.27 (±37.01) months in the standard treatment arm. At 12 weeks after TDF discontinuation, the percentage of change (% change) in rate of fractional excretion (FE) of phosphate was improved 31.3 (IQR −4.4 to 65.3) in the early discontinuation arm but worsening −17.4 (IQR 39 to 34.7) in the standard treatment arm (*p *=* *0.052). The changes in FE of uric, urine protein to creatinine ratio and urine β2 microglobulin were also improved at 12 weeks after TDF withdrawal. The eGFR was stable (% change 0 [IQR −8.8 to 6]) in the early discontinuation arm at 12 weeks after TDF withdrawal but was worsening −1.8 (IQR −2.7 to 9; *p *=* *0.678) in the standard treatment arm.

**Conclusions: **Early detection in TDF‐induced nephropathy leads to early withdrawal and may result in a better outcome of renal function.

## COMORBIDITIES AND COMPLICATIONS OF DISEASE AND/OR TREATMENT: OTHER

## P210

### Haematological manifestations in virologically‐suppressed people living with HIV

**D Akdag^1^, A Dehlbæk Knudsen^1^, R Faber Thudium^1^, D Kirkegaard‐Klitbo^2^, P De Nully Brown^3^, S Afzal^4^, BG Nordestgaard^5^, J Lundgren^6^ and S Dam Nielsen^1^**

^1^Department of Infectious Diseases 8632, Rigshospitalet, Copenhagen, Denmark. ^2^Department of Infectious Diseases, Hvidovre Hospital, Copenhagen, Denmark. ^3^Department of Hematology, Rigshospitalet, Copenhagen, Denmark. ^4^Department of Clinical Biochemistry, Herlev and Gentofte Hospital, Copenhagen, Denmark. ^5^Faculty of Health and Medical Sciences, University of Copenhagen, Copenhagen, Denmark. ^6^Department of Infectious Diseases 8632, CHIP & Rigshospitalet, Copenhagen, Denmark

**Background: **Data on prevalence, incidence and severity of cytopenia, more specifically anaemia, neutropenia, lymphocytopenia and thrombocytopenia in PLWHIV are inconclusive. We aimed to determine the prevalence of the abovementioned cytopenias in well‐treated PLWHIV compared to uninfected controls. Furthermore, we determined if HIV is an independent risk factor for the four dependent outcomes of interest.

**Materials and methods: **PLWHIV without detectable viral replication or chronic hepatitis infection were recruited from the Copenhagen Comorbidity in HIV Infection (COCOMO) Study. Age‐ and sex‐matched uninfected controls were recruited from the Copenhagen General Population Study. Demographic data were collected from uniform questionnaires. Venous blood samples were collected and analysed using the same laboratory and equipment in the two populations. The four outcomes were defined, according to the Common Terminology Criteria for Adverse Events (CTCAE) v.5.0. Logistic regression analyses were performed to determine the association between HIV infection and the four dependent outcomes of interest, after adjusting for age, sex, ethnicity, smoking status and alcohol intake.

**Results: **Seven hundred and ninety‐six PLWHIV and 2388 uninfected controls were included in the study. All PLWHIV had undetectable viral replication. The majority of PLWHIV and controls were male (84.1% and 85.2%, respectively) of Scandinavian descent (73.3% and 64.9%, respectively). Median age was 50.2 (43.2 to 57.8) for PLWHIV and 50.5 (43.4 to 58.5) for controls. Most PLWHIV (99.2%) were on cART, and the median time since diagnosis was 13.7 years. PLWHIV had a higher prevalence of anaemia (6.9% vs. 3.4%, *p *≤* *0.0001), neutropenia (1.3% vs. 0.2%, *p *≤* *0.0002) and thrombocytopenia (5.5% vs. 2.7%, *p *=* *0.0001) compared to uninfected controls. There was no difference in prevalence of lymphocytopenia between PLWHIV and controls (2.4% vs. 1.6%, *p *=* *0.1687). In adjusted multivariable logistic regression analyses, HIV was independently associated with the prevalence of anaemia (adjusted odds ratio (aOR) 2.0 [95% CI 1.4 to 3.0]), neutropenia (aOR 6.3 [95% CI 2.0 to 19.6]) and thrombocytopenia (aOR 2.7 [95% CI 1.8 to 4.2]) (Table 1). No association was found between HIV and lymphocytopenia.

**Table:** Abstract P210 – Table 1. Multivariate analyses of risk factors associated with anaemia, neutropenia, lymphocytopenia and thrombocytopenia

	Anaemia		Neutropenia		Lymphocytopenia		Thrombocytopenia	
aOR [95% CI]	*p* value	aOR [95% CI]	*p* value	aOR [95% CI]	*p* value	aOR [95% CI]	*p* value
HIV, yes vs. no	2.0 [1.4 to 3.0]	0.0003	6.3 [2.0 to 19.6]	0.0014	1.6 [0.9 to 2.9]	0.1270	2.7 [1.8 to 4.2]	<0.0001
Age, per decade	1.5 [1.2 to 1.7]	<0.0001	0.8 [0.5 to 1.3]	0.3397	1.6 [1.3 to 2.1]	<0.0001	1.4 [1.2 to 1.7]	0.0003
Sex, male vs. female	1.2 [0.7 to 2.0]	0.5658	1.3 [0.3 to 6.1]	0.7623	0.9 [0.4 to 1.9]	0.7192	1.2 [0.7 to 2.3]	0.4983
Smoking	0.4894	0.9286	0.1465	0.0305
Never	Ref.	Ref.	Ref.	Ref.
Current	0.8 [0.4 to 1.3]	1.2 [0.3 to 4.5]	0.4 [0.1 to 1.0]	0.5 [0.3 to 0.9]
Former	1.1 [0.7 to 1.6]	0.9 [0.2 to 3.3]	1.0 [0.6 to 1.7]	0.6 [0.4 to 1.0]
Alcohol, units per week	1.0 [1.0 to 1.0]	0.0584	1.0 [1.0 to 1.1]	0.9859	1.0 [1.0 to 1.0]	0.0724	1.0 [1.0 to 1.0]	0.2752

**Conclusion: **Even in PLWHIV with successful viral suppression and absence of chronic hepatitis infection HIV infection is independently associated with higher prevalence of anaemia, neutropenia and thrombocytopenia. Although relatively rare, cytopenias in PLWHIV remain prevalent and require ongoing attention and monitoring.

## P211

### Polypharmacy and drug‐drug interactions in HIV‐infected subjects in the region of Madrid (Spain): a population‐based study

**B López‐Centeno^1^, C Badenes‐Olmedo^2^, Á Mataix‐Sanjuan^1^, K McAllister^3^, J Bellón‐Cano^4^, P Balsalobre^4^, J Benedí^5^, S Khoo^3^, M Calvo‐Alcántara^1^ and J Berenguer^4^**

^1^Servicio Madrileño de Salud, Subdirección General de Farmacia y Productos Sanitarios, Madrid, Spain. ^2^Escuela Técnica Superior de Ingeniería Informática, Ontology Engineering Group, Universidad Politécnica de Madrid, Madrid, Spain. ^3^Department of Pharmacology, University of Liverpool, Liverpool, UK. ^4^Instituto de Investigacion Sanitaria, Hospital General Universitario Gregorio Marañón, Madrid, Spain. ^5^Departamento de Farmacología, Facultad de Farmacia, Universidad Complutense de Madrid, Madrid, Spain

**Background: **We analysed polypharmacy and drug‐drug interactions (DDIs) in HIV‐infected subjects (HIV + S) who received ARVs in the region of Madrid.

**Materials and methods: **We analysed the computerised dispensation registry of community and hospital pharmacies from the Madrid Regional Health Service (SERMAS) between 1 January and 30 June 2017. Co‐meds were classified according to the ATC classification system. Polypharmacy was considered as the intake of ≥5 non‐antiretroviral medications (co‐meds). DDIs between ARVs and co‐meds were screened using a customised application programming interface connecting the SERMAS database and the University of Liverpool (UoL) drug interactions database. DDIs were classified according to UoL criteria and summarised with a traffic light method.

**Results: **During the study period, 6,636,451 different individuals (22,945 HIV + S) received medications in the region of Madrid. ART was predominantly PI‐based in paediatric patients (47.3%), and INSTI‐based in adults (57.5%). Overall, 16,402 HIV + S (71.5%) were taking at least one co‐med. The most frequently dispensed co‐meds among HIV + S were nervous system drugs (61.5%), gastrointestinal drugs (52.9%) and anti‐infectives for systemic use (41.0%). Polypharmacy was observed in 7557 HIV + S (32.9%), and 1,465,552 non–HIV + S (22.2%); *p *<* *0.001. Polypharmacy was statistically significantly higher among HIV + S than among non–HIV + S across all age strata except for those aged ≥75 years. The prevalence of the different categories of DDIs among HIV + S according to UoL criteria were: red‐flag 729 (3.2%), orange‐flag 4193 (18.3%), yellow‐flag 2363 (10.3%), green‐flag 11,811 (51.5%) and grey‐flag 26 (0.1%). Main red‐flag interactions among HIV + S are shown in Table 1.

**Table:** Abstract P211 – Table 1. Main red‐flag interactions among HIV‐infected subjects (n = 729)

ARVs class	ARVs	Co‐meds (ATC code)	n (%)
Boosted PIs (RTV or COBI)	bATV, bDRV, LPV/r	Corticosteroids (R01AD, R03BA, R03AK, H02AB)	332 (45.5)
Boosted PIs (RTV or COBI)	bATV, bDRV, LPV/r	Statins (C10AA)	72 (9.9)
Boosted PIs (RTV or COBI)	bATV, bDRV, LPV/r	Antipsychotic drugs (N05AH)	10 (1.4)
Boosted INSTI	FTC/TDF/EVG/COBI	Corticosteroids (R01AD, R03BA, R03AK, H02AB)	25 (3.4)
nnRTIs	EFV, NVP	Imidazole and triazole derivatives (D01AC, J02AC)	61 (8.4)
nnRTIs	RPV	Proton pump inhibitors (A02BC)	9 (1.2)
All classes	All ARVs	Other co‐meds	220 (30.2)

**Conclusions: **Polypharmacy was significantly more frequent among HIV + S than among non–HIV + S across all age strata, except for individuals aged ≥75 years. Nervous system drugs followed by gastrointestinal drugs and anti‐infectives were the most frequently prescribed co‐meds among HIV + S. The prevalence of red‐flag DDIs was 3.2%. The most frequent involved medications in red‐flag DDIs were boosted PIs and boosted INSTI among ARVs, and corticosteroids, statins and imidazole and triazole derivatives among co‐meds.

## P212

### Risky alcohol consumption and associated health behaviour among HIV‐positive and HIV‐negative patients in a UK sexual health and HIV clinic: the HAZAL study

**E Suonpera^1^, R Matthews^1^, A Milinkovic^2^ and A Arenas‐Pinto^1^**

^1^UCL Institute for Global Health, University College London, London, UK. ^2^Chelsea & Westminster Hospital, Chelsea & Westminster Hospital NHS Foundation Trust, London, UK

**Background: **Alcohol misuse has been associated with negative consequences in HIV‐positive patients. However, data on real prevalence of risky alcohol consumption in British HIV‐infected population are limited. We estimated the prevalence of risky alcohol consumption among HIV‐positive and comparable HIV‐negative patients attending our sexual health and HIV clinic.

**Materials and methods: **Two hundred and ninety‐six patients (227 HIV positive and 69 HIV negative) completed a self‐administrated cross‐sectional survey comprised of the following validated standardised instruments: the Alcohol Use Disorders Identification Test (AUDIT), the Patient Health Questionnaire‐9 (PHQ‐9), the Drug Use Disorders Identification Test (DUDIT) and the Centre for Adherence Support Evaluation (CASE) Adherence Index. Socio‐demographic, health and sexual behaviour data were collected. We assessed the prevalence of risky alcohol consumption (AUDIT score ≥8) among the groups of patients and evaluated the effects of socio‐demographic, health and sexual behaviour factors on the risky alcohol consumption using logistic regression. All analyses accounted for other variables associated with risky alcohol consumption in univariate analyses (≤0.10).

**Results: **The HIV‐positive and HIV‐negative patients were predominantly men (92% and 94%, respectively) of white ethnicity (76% and 67%, respectively) with a median age (IQR) of 46 (39 to 53) and 40 (33 to 47), respectively. Twenty‐five percent of HIV‐positive and 36% of HIV‐negative patients reported risky alcohol consumption. Depressive symptoms (PHQ‐9 score ≥10), harmful drug use (DUDIT score men ≥6; women ≥2) and smoking were reported in 10% and 13%, 25% and 29% and 19% and 17% respectively among HIV‐positive and HIV‐negative patients. Among HIV‐positive and HIV‐negative patients 44% and 82% reported ≥3 sexual partners, 45% and 86% unprotected sex, 15% and 30% STD diagnoses and 23% and 45% chemsex participation respectively in three months preceding the survey. Majority (88%) of HIV‐positive patients adhered well to ART (CASE score >10). Presence of depressive symptoms (*p *<* *0.001), smoking (*p *=* *0.04), harmful drug use (*p *<* *0.001), chemsex participation (*p *<* *0.001) and poor adherence to ART (*p *=* *0.01) were associated with risky alcohol consumption among HIV‐positive patients in the univariate analyses, but only depressive symptoms (*p *=* *0.03) and harmful drug use (*p *=* *0.007) remained significant in multivariable analyses. Among the HIV‐negative patients presence of depressive symptoms and harmful drug use had borderline associations with risky alcohol consumption (*p *=* *0.05 and 0.09 respectively) in univariate analyses, but in multivariable analyses these associations diminished (Table 1, 2).

**Conclusions: **Risky alcohol consumption was observed in a quarter of our HIV‐positive participants and was associated with increased depressive disorders and harmful drug use. Among a sample of our HIV‐negative patients these associations were not present.

**Table:** Abstract P212 – Table 1. Associations between risky alcohol consumption (AUDIT score ≥8) and other health and sexual behaviour variables among HIV‐positive patients

Proportion of patients reporting risky alcohol consumption (n = 57/227 (25.11%))	OR (95% CI)	*p* value	aOR (95% CI)	*p* value
Depressive symptoms (PHQ‐9)					
None/mild	39/195 (20.00)	1.00	1.00
Moderate/severe	14/24 (58.33)	5.6 (2.23 to 14.09)	<0.001	3.13 (1.12 to 8.77)	0.03
Smoking status					
Never/ex‐smoker	41/183 (22.40)	1.00	1.00
Smoker	16/42 (38.10)	2.13 (1.04 to 4.39)	0.04	1.36 (0.57 to 3.34)	0.48
Drug‐related problems (DUDIT)					
No	25/148 (16.89)	1.00	1.00
Yes	28/57 (49.12)	4.75 (2.32 to 9.72)	<0.001	3.60 (1.42 to 9.14)	0.007
STD diagnosis					
No	42/185 (22.70)	1.00	1.00
Yes	12/33 (36.36)	1.95 (0.88 to 4.31)	0.09	1.43 (0.55 to 3.73)	0.46
Chemsex					
No	32/167 (19.16)	1.00	1.00
Yes	23/53 (43.40)	3.23 (1.63 to 6.43)	<0.001	1.22 (0.46 to 3.21)	0.69
Adherence to ART					
Poor	12/27 (44.44)	2.82 (1.21 to 6.58)	0.01	1.54 (0.52 to 4.57)	0.44
Good	42/190 (22.11)	1.00	1.00

**Table:** Abstract P212 – Table 2. Associations between risky alcohol consumption (AUDIT score ≥8) and other health and sexual behaviour variables among HIV‐negative patients

Proportion of patients reporting risky alcohol consumption (n = 25/69 (36.23%))	OR (95% CI)	*p* value	aOR (95% CI)	*p* value
Depressive symptoms (PHQ‐9)					
None/mild	19/60 (31.67)	1.00	1.00
Moderate/severe	6/9 (66.67)	4.32 (0.92 to 20.27)	0.05	3.99 (0.85 to 18.63)	0.08
Drug‐related problems (DUDIT)					
No	12/43 (27.91)	1.00	1.00
Yes	10/20 (50.00)	2.58 (0.83 to 8.05)	0.09	2.29 (0.60 to 8.76)	0.22
Chemsex					
No	12/37 (32.43)	1.00	1.00
Yes	12/31 (38.71)	1.32 (0.48 to 3.60)	0.59	0.91 (0.25 to 3.26)	0.88

## P213

### Health status and quality of life in PLWHIV: results from the ICONA cohort

**A Cingolani^1^, J Romaine^2^, A Tavelli^3^, F Maggiolo^4^, E Girardi^5^, A Antinori^6^, A Cascio^7^, A Cattelan^8^, A De Luca^9^, M Murray^10^, A D'Arminio Monforte^11^ and C Bradley^12^, on behalf of Icona Foundation Study Group**

^1^Institute of Infectious Diseases, Catholic University, Roma, Italy. ^2^Health Psychology Research Unit, University of London, London, UK. ^3^ICONA Foundation, University of Milano, Milan, Italy. ^4^Infectious Diseases, Azienda Ospedaliera S. Giovanni XXIII, Bergamo, Italy. ^5^Epidemiology, National Institute for Infectious Diseases L. Spallanzani, Roma, Italy. ^6^HIV/AIDS, National Institute for Infectious Diseases L. Spallanzani, Roma, Italy. ^7^Infectious Diseases, AOU Policlinico P. Giaccone, Palermo, Italy. ^8^Infectious Diseases, Azienda Ospedaliera Padova, Padova, Italy. ^9^Infectious Diseases, University of Siena, Siena, Italy. ^10^ViiV Healthcare, London, UK. ^11^Infectious Diseases, University of Milano, Milano, Italy. ^12^Health Psychology Research Ltd, Royal Holloway University of London, London, UK.

**Background: **As HIV has become a long‐term condition, it is important to evaluate the impact of therapies on patient‐reported outcomes (PROs). Here we report analyses of associations between clinical/demographic variables and health status and quality of life (QoL) in PLWHIV, enrolled in ICONA.

**Materials and methods: **The HIV‐Dependent QoL (HIVDQoL) and EQ‐5D‐3L health status tool were administered consecutively to two groups of ICONA patients: newly diagnosed, pre‐treatment patients and those with >six months of cART, from March 2017 to March 2018. The analyses focused on the HIVDQoL overview item measuring generic QoL (3 = ’excellent’ to −3 = ’extremely bad’) and the EQ‐5D visual analogue score (EQ‐VAS) measuring self‐rated health (100 = ’best imaginable health state’ to 0 = ‘worst’). Analyses included non‐parametric tests of difference and correlational analyses.

**Results: **One hundred and thirty‐five patients were included (122 men; 13 women), mean age 43 (SD 12.25). One hundred and seven patients were on cART (NNSTI, N = 66; NNRTI, N = 23; PI, N = 15/r‐based regimen). Mode of transmission included: MSM (N = 76), heterosexual (N = 40) and IDU (N = 10). Mean CD4+ was 655/mm^3^ (SD 316) for those on cART and 429/mm^3^ (SD 259) for patients’ pre‐treatment. Mean self‐reported health (EQ‐VAS) was 79 (SD 14.57) for cART‐treated and 78 (SD 18.73) for those pre‐treatment. Generic QoL (HIVDQoL item (1) mean was 1.21 (SD 1.19) (>‘good’ QoL) for cART‐treated and 0.48 (SD 1.74) (midway between ‘neither good nor bad’ and ‘good’) for those pre‐treatment. EQ‐VAS health scores were found to differ by mode of transmission, with MSM reporting better health than IDU (*p *=* *0.022) and those reporting heterosexual transmission (*p *=* *0.043). However, there was no difference in QoL by mode of transmission. Treatment with cART was associated with better QoL than pre‐treatment status (*p *=* *0.049), without differences in health ratings. QoL, but not health status, was significantly worse for patients with CD4 count of <200 than those with CD4 count of 200 to 499 (*p *=* *0.035) or CD4 count ≥500 (*p *=* *0.037). Correlational analyses for cART‐treated patients showed age was negatively related to both QoL (−0.312, *p *=* *0.001) and health (−0.357, *p *<* *0.001).

**Conclusion: **Although both generic QoL and health status were worse in older (vs. younger) PLWHIV, the two outcomes showed different patterns according to clinical variables, with cART‐treated patients reporting better QoL but no difference in perceived health compared with pre‐treatment patients. QoL, but not perceived health, was also better in patients with CD4 counts >200. Perceived health, but not QoL, differed with mode of HIV transmission. QoL is not simply a reflection of health status and it is important to measure both outcomes.

## P214

### Syphilis on the rise in HIV‐positive MSM in Germany

**J Wesselmann, C Boesecke, J Wasmuth, J Rockstroh and C Schwarze‐Zander**

Department of Internal Medicine I, University Hospital Bonn, Bonn, Germany

**Background: **STDs, such as syphilis, have been increasing in recent years among MSM, often HIV+ patients, due to more frequent condomless sex. Aim of our study was to evaluate incidence of syphilis infection, impact on immunological and laboratory markers and treatment response of serological markers in a German cohort.

**Methods: **This retrospective study included 859 HIV+ patients screened for syphilis infection (TPPA, VDRL) November 2015 to May 2017 in the HIV outpatient clinic at Bonn University Hospital. The impact of syphilis and its treatment on renal function markers (serum creatinine, GFR), liver enzymes (gamma‐GT, ALT, AST), inflammatory parameters and blood count (CRP, Hb, LDH) and immune response (leucocytes, CD4 count, CD8 count, CD4/CD8 ratio) was investigated three to six months before, at time of syphilis diagnosis, and three to six months after treatment. Serological response to syphilis treatment (VDRL, TPPA) was investigated every three months after treatment.

**Results: **In the study period 43/859 (5%) patients were newly diagnosed with syphilis. Of these 3/43 (7%) were re‐infected within the observation period. Compared to incidence of syphilis infection between 2000 and 2010 there was a 2.4‐fold increase in 2016. Past syphilis infection was detected in 28% (244/859). All patients with syphilis were male and 97% MSM, compared to the whole study population patients were younger (mean age 44 years vs. 49 years) and fewer had symptomatic HIV disease (77% CDC stage A vs. 57%). Only 37% developed symptoms of syphilis (47% exanthema, 20% chancres, 20% uveitis, 13% urethritis). At the three observed timepoints mean gamma‐GT increased from 49 U/L to 70 U/L (*p *=* *0.001) and decreased to 53 U/L, respectively, CRP increased from 2.1 to 7.4 mg/dL and decreased after treatment to 1.6 mg/dL (*p *=* *0.002) and the mean CD4 count dropped from 670/µL to 646/µL at time of syphilis diagnosis and increased significantly after treatment to 715/µL (mean, *p *=* *0.022). The relative CD4 cell count did not change during the observation period. Following syphilis treatment VDRL titer showed a slow decrease. After three to six months only 50% showed a ≥ 4‐fold decrease, which increased after nine to twelve months to 86%.

**Conclusion: **Syphilis co‐infection has dramatically increased in our HIV+ population, especially in younger, healthier MSM. Regular screening is extremely important in this group of HIV+ patients as more than half of syphilis cases miss symptoms of infection. Elevation of gamma‐GT and CRP and decrease of absolute CD4 cell count may be an indicator of syphilis infection. VDRL can show a slow decrease after treatment and requires monitoring.

## P215

### An assessment of how effectively health systems monitor HIV‐associated comorbidities, using current global and European frameworks

**K Safreed‐Harmon^1^, J Pericàs^2^, M Kall^3^, U Davidovich^4^, J del Amo^5^, J Lazarus^1^ and J Anderson^6^**

^1^Barcelona Institute of Global Health (ISGlobal), University of Barcelona, Barcelona, Spain. ^2^Infectious Disease Service, Hospital Clínic, University of Barcelona, Barcelona, Spain. ^3^National Infection Service, Public Health England, London, UK. ^4^Public Health Service of Amsterdam, Amsterdam, Netherlands. ^5^National Center for Epidemiology, Institute of Health Carlos III, Madrid, Spain. ^6^Jonathan Mann Clinic, Homerton University Hospital NHS Foundation Trust, London, UK

**Background: **Today we have the tools to deliver effective long‐term viral suppression of HIV. Data from continua of care show increasing proportions of PLWHIV progressing to viral suppression in countries at all income levels. Yet alongside this progress, there is a growing burden of non‐AIDS‐defining comorbidities and related health concerns for  PLWHIV. Decisions about which elements of service coverage and which health outcomes countries monitor have important implications for how health systems focus their HIV responses. This study examines whether existing monitoring frameworks sufficiently enable European countries to observe and understand the comorbidities that impact on the health and well‐being of PLWHIV.

**Materials and methods: **Drawing on recent literature, we identified 15 non‐AIDS‐defining comorbidity areas that contribute to poor health in PLWHIV globally: bacterial and viral infections (excluding bacterial STIs), bacterial STIs (chlamydia, gonorrhoea and syphilis), cardiovascular, digestive, drug toxicities, endocrine/metabolic, haematological, liver (including HBV and HCV), malignancies, malnutrition/wasting, neurological, parasitic infections (including malaria), renal, respiratory and psychiatric conditions. Three researchers independently assessed the extent to which each comorbidity area was monitored with regard to: (1) service access; and (2) disease burden in four monitoring frameworks: Global AIDS monitoring 2018 (UNAIDS); Modular framework handbook (The Global Fund); MER 2.0 indicator reference guide (PEPFAR); and the 2018 Dublin Declaration questionnaire (European Centre for Disease Prevention and Control). Researchers assigned grades of A when comorbidities were addressed comprehensively, B when comorbidities were addressed but not comprehensively and C when comorbidities were not addressed. Discrepancies were resolved through consultation. 

**Results: **Over half (8/15) of the comorbidities were not mentioned in any of the four monitoring frameworks (malignancies, parasitic infections, and digestive, endocrine/metabolic, haematological, neurological, renal and respiratory diseases/disorders). Across the four frameworks, there were more grades of A or B for access to services (11) than for comorbidity burden (four), and neither MER 2.0 nor the Dublin questionnaire included any indicators monitoring the comorbidity burden. The only item addressed comprehensively in Global AIDS Monitoring was comorbidity burden for drug toxicities. The only items addressed comprehensively in the Dublin questionnaire were access to services for bacterial STIs, liver diseases and psychiatric disorders.

**Conclusions: **We found major HIV monitoring frameworks fail to comprehensively address most non‐AIDS‐defining comorbidities, particularly chronic conditions associated with ageing. As the continuum of HIV care is reconceptualised to reflect long‐term health and well‐being, monitoring frameworks must be revised to include non‐AIDS‐defining comorbidities. This will encourage prevention, diagnosis and treatment of comorbidities consequently improving long‐term health outcomes and quality of life.

## P216

### Delayed but adequate serologic response to syphilis treatment in HIV‐positive adults

**M Ren^1^, L Szadkowski^2^, D Tan^3^, S Walmsley^4^**

^1^Medicine, University of Toronto, Toronto, Canada. ^2^Biostatistics Research Unit, University Health Network, Toronto, Canada. ^3^Infectious Diseases, St. Michael's Hospital, Toronto, Canada. ^4^Infectious Diseases, University Health Network, Toronto, Canada

**Background: **Adequate response to syphilis treatment is a four‐fold decrease in serum RPR at 6 (early syphilis) and 12 months (latent syphilis). We characterized the timeline of serologic response to syphilis treatment in HIV‐positive adults.

**Methods: **We conducted a chart review of 532 HIV‐positive adults with positive syphilis serology between 2000 and 2017. Inclusion criteria were: reactive pre‐treatment RPR titer; documentation of date and type of syphilis therapy; reversion to a non‐reactive RPR or at least six months or one year of follow‐up for early syphilis and late syphilis/neurosyphilis, respectively. The first eligible episode was included. Time to four‐fold decrease and non‐reactive RPR was calculated using Kaplan‐Meier estimates. Univariable proportional hazards models assessed associations between clinical covariates and time to four‐fold decrease and non‐reactive RPR.

**Results: **Two hundred and thirty‐one patients did not have a reactive pre‐treatment RPR titer. One hundred and eighty‐nine male patients (87% MSM) met inclusion criteria. Median (IQR) age was 42 (35 to 48), median (IQR) CD4 count was 443 (273 to 609) and 56% of patients had a suppressed viral load. Seventy‐five percent were on ARVs and 57% had a baseline RPR titer ≥1:64. Twelve percent were primary syphilis, 28% secondary, 12% early latent, 28% late latent and 19% neurosyphilis. Median (IQR) follow‐up was 2.55 (1.53 to 6.14) years. Seventy‐two percent received IM doses of benzathine penicillin G (27% 1 dose, 45% 2 to 3 doses), 21% received intravenous penicillin G and 5% doxycycline. One hundred and eighty‐four (97.4%) had at least a four‐fold decrease in RPR and 96 (52.2%) reverted to a non‐reactive RPR. The median (95% CI) time to four‐fold decrease and non‐reactive RPR was 0.37 (0.33 to 0.45) years and 2.72 (2.30 to 4.53) years, respectively. Patients with baseline titer ≥1:64 were more likely to achieve a four‐fold decrease (HR 1.36, *p *=* *0.05), but less likely to achieve a non‐reactive RPR (HR 0.41, *p *<* *0.0001). Patients with neurosyphilis or latent syphilis were less likely to achieve a four‐fold decrease (HR 0.67, *p *=* *0.048 and HR 0.61, *p *<* *0.01, respectively) or a non‐reactive RPR (HR 0.27, *p *<* *0.001 and HR 0.42, *p *<* *0.001, respectively) compared to patients with primary or secondary syphilis. Age, CD4 count and viral load were not associated with time to four‐fold decrease or non‐reactive RPR.

**Conclusions: **Serologic response to syphilis treatment in HIV‐positive adults is adequate. Four‐fold RPR decrease and seroreversion required an average of four months and 2.7 years, respectively. Patients with higher baseline titers were more likely to achieve a four‐fold decrease, but less likely to achieve a non‐reactive RPR.

## P217

### Clinical characteristics and outcome of infective endocarditis in non‐intravenous drug users (IDU) HIV‐infected patients and influence of HIV‐infection on prognosis: a prospective study of the International Collaboration on Endocarditis (ICE)

**M Hernández‐Meneses^1^, J Llopis^2^, V Chu^3^, J Ambrosioni^1^, D Wray^4^, N Fernández‐Hidalgo^5^, J Baddley^6^, A Moreno^1^, E Athan^7^ and J Miró^1^**

^1^Infectious Diseases Service, Hospital Clinic, Barcelona, Spain. ^2^Statistics Department, Hospital Clinic, Barcelona, Spain. ^3^Infectious Diseases Service, Duke Clinical Research Institute, Durham, NC, USA. ^4^Infectious Diseases Service, Medical University South Carolina, Charleston, SC, USA. ^5^Infectious Diseases Service, Hospital Vall´d Hebron, Barcelona, Spain. ^6^Infectious Diseases Service, University of Alabama, Birmingham, AL, USA. ^7^Infectious Diseases Service, Barwon Health, Geelong, Australia

**Introduction: **Few studies analysing clinical characteristics and prognosis of infective endocarditis (IE) in non‐IDU HIV‐infected patients were performed before or during the first years of HAART. This study aimed to describe the current clinical characteristics of IE in non‐IDU HIV‐infected patients, to compare them with the general population with IE and to analyse whether HIV infection influences prognosis.

**Material and methods**

Prospective multicentre study of 56 cases of definitive IE in HIV‐infected patients and 7367 of definitive IE in the general population without HIV infection. IDU cases were excluded. There were 64 centres in the 28 countries of the International Collaboration on Endocarditis cohort between 2000 to 2006 and 2008 to 2012. A propensity analysis was performed matching each HIV‐positive IE case with an HIV‐negative control with a similar age and the same gender, year of diagnosis, type of endocarditis, affected valve and causative organism.

**Results: **The clinical characteristics of IE in HIV‐infected patients and in the general population are depicted in Table 1. In comparison with the general population with IE, HIV‐infected patients with IE were younger (45 years vs. 70 years, *p *<* *0.001), predominantly male (84% vs. 67%, *p *=* *0.001), with higher rates of haemodialysis (21% vs. 9%, *p *=* *0.04) and previous IE (23% vs. 9%, *p *=* *0.01) and lower prevalence of diabetes (11% vs. 20%, *p *=* *0.02). HIV infection was acquired by sexual transmission. Median (IQR) CD4 (N = 9) was 70 cells/mm^3^ (20 to 336) and 80% of patients had undetectable plasma viral load (n = 6). The type of IE was similar in both groups as was the aetiology, except for S. gallolyticus, which was more frequent in HIV‐negative IE cases (2% vs. 7%, *p *=* *0.005). The rates of vegetations (69% vs. 81%) and mitral involvement (26% vs. 39%) were significantly lower in HIV‐positive patients. Cardiac surgery was less frequent in HIV‐infected patients (26% vs. 48%, *p *<* *0.001), but in‐hospital and one‐year mortality rates were similar in both groups. In the propensity analysis, complications (heart failure, stroke, systemic embolisms and persistent bacteraemia) were similar in patients and controls. However, cardiac surgery was significantly lower in HIV‐infected patients (26% vs. 52%, *p *=* *0.004) without significant differences in in‐hospital mortality (20% vs. 14%, *p *=* *0.45) and one‐year mortality (36% vs. 26%, *p *=* *0.26).

**Table:** Abstract P217 – Table 1. Clinical characteristics of IE in patients with and without HIV infection

HIV positive (n = 56)	HIV negative (n = 7367)	*p* value
Age	45 (35 to 55)	70 (45 to 73)	<0.001
Sex	47 (83.9%)	4945 (67.3%)	0.001
Comorbidities			
Diabetes	6 (10.7%)	1457 (20.1%)	0.02
Haemodialysis	10 (20.8%)	533 (8.8%)	0.04
Cancer	6 (10.7%)	809 (11.2%)	0.92
Previous IE	13 (23.2%)	655 (9%)	0.01
Symptoms for less than 30 days	45 (83.9%)	5349 (79.5%)	0.71
IE type			
Native	36 (66.7%)	4580 (64.3%)	0.71
Prosthetic	15 (27.8%)	1927 (27%)	0.31
Intracardiac device IE (PCM/ICD/CRT)	3 (5.6%)	621 (8.7%)	0.31
Aortic IE	26 (48%)	3564 (50%)	0.80
Mitral IE	20 (37%)	3294 (46.2%)	0.17
Microorganism			
Staphylococcus aureus	17 (31.5%)	1736 (25.6%)	0.35
Staphylococcus coagulase negatives	5 (9.3%)	810 (11.9%)	0.50
Viridans group streptococci	7 (13%)	1262 (18.6%)	0.22
Streptococcus gallolyticus (S. bovis)	1 (1.9%)	477 (7%)	0.005
Enterococcus sp.	4 (7.4%)	812 (12%)	0.21
HACEK	1 (1.9%)	98 (1.4%)	0.82
Negative culture	4 (7.4%)	509 (7.5%)	0.98
Echo and outcome data			
Echocardiographic vegetation	37 (68.5%)	5909 (81.3%)	0.04
Mitral valve vegetation	14 (25.5%)	2846 (39.3%)	0.02
Para‐valvular abscess	7 (12.5%)	1117 (15.8%)	0.45
CNS embolism	7 (12.5%)	1265 (17.5%)	0.26
Other systemic embolisms	10 (17.9%)	1621 (22.4%)	0.38
Heart failure	14 (25.5%)	3487 (47.7%)	0.30
Persistent bacteraemia (>seven days)	9 (16.4%)	644 (9.3%)	0.16
Cardiac surgery in‐hospital	14 (25.5%)	3487 (47.7%)	<0.001
In‐hospital mortality	11 (19.6%)	1357 (18.5%)	0.83
One‐year cardiac surgery	4 (12.5%)	267 (7.1%)	0.36
One‐year mortality	18 (36%)	1914 (32.2%)	0.58

CRT = cardiac resynchronisation therapy; ICD = automatic cardiac‐defibrillator; PCM = pacemaker.

**Conclusions: **Compared to the general population with IE, HIV‐infected IE patients are younger and predominantly male and have a different comorbidity pattern. Although HIV infection did not influence the prognosis, these patients had less access to cardiac surgery.

## P218

### Switching from tenofovir disoproxil fumarate to tenofovir alafenamide and hepatic safety: a new paradigm?

**N Squillace^1^, E Ricci^2^, B Menzaghi^3^, G Migliorino^1^, G De Socio^4^, S Passerini^5^, C Martinelli^6^, M Mameli^7^, P Maggi^8^, K Falasca^9^, L Cordier^5^, B Celesia^10^, E Salomoni^11^, A Di Biagio^12^, G Pellicano’^13^ and P Bonfanti^14^**

^1^Infectious Diseases Unit, ASST Monza, San Gerardo Hospital, University of Milano‐Bicocca, Monza, Italy. ^2^Fondazione IRCCS Ca’ Granda, Ospedale Maggiore Policlinico, Milano, Italy. ^3^Unit of Infectious Diseases, ASST della Valle Olona Busto Arsizio (VA), Busto Arsizio, Italy. ^4^Department of Internal Medicine 2, Infectious Diseases Unit Santa Maria della Misericordia General Hospital, Perugia, Italy. ^5^1st Department of Infectious Diseases, ASST Fatebenefratelli Sacco, Milano, Italy. ^6^SOD Malattie Infettive e Tropicali, AOU Careggi, Firenze, Italy. ^7^Department of Medical Surgery and Experimental Sciences, Infectious Diseases Unit, University of Sassari, Sassari, Italy. ^8^Infectious Disease Clinic, University of Bari, Bari, Italy. ^9^Department of Medicine/Scientific Aging, Clinic of Infectious Diseases, University G. D'Annunzio, Chieti, Italy. ^10^Unit of Infectious Diseases, University of Catania, ARNAS Garibaldi, Catania, Italy. ^11^Infectious Diseases Unit, Santa Maria Annunziata Hospital, Usl Centro, Firenze, Italy. ^12^Infectious Diseases, San Martino Hospital Genoa, Genoa, Italy. ^13^Unit of Infectious Diseases, Department of Human Pathology of Adult and Dev. Age, University of Messina, Messina, Italy. ^14^Unit of Infectious Diseases, A. Manzoni Hospital, Lecco, Italy

**Background: **A switch from tenofovir disoproxil fumarate (TDF) to tenofovir alafenamide (TAF) was associated with a better renal and bone profile. Some studies reported liver toxicity due to TDF [1,2]. We aimed at investigating the effect of switching TDF to TAF on lipid, renal and hepatic safety profile.

**Methods: **Consecutive HIV patients (pts) enrolled in Surveillance Cohort Long‐term Toxicity Antiretrovirals/Antivirals (SCOLTA) project switching from TDF/FTC/EVG/cOBI to TAF/FTC/EVG/COBI for any reasons were included. Changes from baseline (T0) to six‐month follow‐up (T1) were evaluated using paired *t*‐test if differences were normally distributed (blood lipids, estimated glomerular filtration rate [eGFR]), and using signed rank test if not (liver enzymes).

**Results: **One hundred and eighty‐eight pts switched from TDF/FTC/EVG/COBI to TAF/FTC/EVG/COBI, and 100 had at least one six‐month follow‐up. They were 79% male, 76% at CDC stage A–B, 97% with undetectable HIV viral load, 36% on second ART regimen. Mean age was 45.1 ± 10.6 years, body mass index 24.5 ± 3.3 kg/m^2^, total cholesterol (TC) 193 ± 37 mg/dL, HDL cholesterol (HDL) 54 ± 40 mg/dL, eGFR 88 ± 18 mL/min, median CD4 cell count 716 cells/µL (interquartile range [IQR] 513 to 914), ART duration 5.6 (IQR 2.0 to 12.2) years, AST 23 (IQR 19 to 30) IU/L and ALT 24 (IQR 18 to 33) IU/L. Sixteen pts were positive for HCV‐Ab and 10 pts for HbsAg. TDF/FTC/EVG/COBI duration before switch was 827 days (range 41 to 1610). At T1, we observed increased TC (+13 ± 26 mg/dL, *p *<* *0.001) and LDL cholesterol (LDL) (+8 ± 28 mg/dL, *p *<* *0.05) while HDL, triglycerides (TG) and TC/HDL ratio remained stable. At T1, both ALT (median ‐2, IQR −7 to 2 IU/L, *p *=* *0.009) and AST (median ‐1, IQR −2 to 5 IU/L, *p *=* *0.02) were significantly reduced. AST and ALT reduction remained significant in HCV‐/HbsAg‐ patients. Among 16 pts with ALT >40 IU/L, a significant proportion reduced this parameter (median change −23.5, −38.5 to −13.5, *p *<* *0.0001). In details 11 pts normalised ALT, four pts had a reduction without normalisation and one no variation. At T1, eGFR showed a slight increase (+2.6 ± 13.0 mL/min, *p *=* *0.05).

**Conclusions: **Switching from TDF/FTC/EVG/COBI to TAF/FTC/EVG/COBI in a real‐life setting was associated with an improvement in eGFR, with increased TC and LDL, and stable HDL, TC/HDL ratio and TG. A significant reduction of ALT and AST, especially in pts without HBV and/or HCV infection, was observed. Further studies are needed to confirm a better liver safety of TAF versus TDF.

**References**

[1] Kovari H, Sabin CA, Ledergerber B, Ryom L, Reiss P, Law M, et al. Antiretroviral drugs and risk of chronic alanine aminotransferase elevation in human immunodeficiency virus (HIV)‐monoinfected persons: the Data Collection on Adverse Events of Anti‐HIV Drugs study. Open Forum Infect Dis. 2016;3:ofw009.

[2] Mandala J, Nanda K, Wang M, De Baetselier I, Deese J, Lombaard J, et al. Liver and renal safety of tenofovir disoproxil fumarate in combination with emtricitabine among African women in a pre‐exposure prophylaxis trial. BMC Pharmacol Toxicol. 2014; 15:77.

## P219

### E‐vaccine registry: systematic vaccine registry improves immunisation coverage in HIV patients

**N Enriquez^1^, V Pecoul^1^, M Hartley^1^, C Siegrist^2^ and A Calmy^1^**

^1^Infectious Diseases, University Hospitals of Geneva, Geneva, Switzerland. ^2^Center for Vaccinology, University Hospitals of Geneva and Faculty of Medicine, Geneva, Switzerland

**Background: **Infections represent a significant threat for HIV patients, with higher attack rates and an increased risk for severe and complicated illness, especially when CD4 counts fall below 200 cells/mm^3^. Despite existing recommendations, HIV patients may not be immune to vaccine‐preventable diseases due to immunisation schedules not being systematically reviewed during routine consultations, as well as concerns over vaccinal side effects and efficacy in this immunocompromised population. The aim of this study is to evaluate the impact of an electronic vaccine record on the immunisation status of HIV patients in Geneva.

**Materials and methods: **In this controlled before‐and‐after cohort study, a total of 328 HIV patients were enrolled between 1 May 2016 and 10 April 2018 at the Infectious Diseases Division of the University Hospitals of Geneva. A vaccinology consultation was offered systematically to all adult patients. After oral consent, vaccine history was taken and immunisation status documented in a national electronic immunisation registry (www.myvaccines.ch), which was accessible by the collaborators of the HIV Division, the patients themselves and their general practitioners. Vaccine status was assessed by an expert clinical decision support system (CDSS) embedded in the registry and based on age, gender, risk factors, registered vaccines and serologies. Incomplete immunisation was defined by the CDSS according to national guidelines. The catch‐up immunisation plan generated by the CDSS was then implemented during follow‐up visits.

**Results: **Of the 328 patients, 152 (46%) were enrolled in 2018. [EN4] The cohort had a median age of 49 years (range 13 to 85) and a male‐to‐female gender ratio of 2.22. CD4 counts were >200 cells/mm^3^ in 296/323 (90%). At enrolment, past HBV infection was documented in 106 (32%) patients, 101 (31%) had been immunised and had documented anti‐HBS antibodies >100 IU/L [AH6] and 121 (37%) were susceptible or had unknown or incomplete HBV immunity. Patients up to date for “basic vaccines” (immunisations/immunity) were: HBV 206 (63%); HAV 189 (58%); varicella 184 (56%); measles 48 (15%); tetanus 8 (2%); and “complementary vaccines”: pneumococcus 0 (0%). By the first interim analysis (1 June 2018), immunisations/immunity rates had increased for all vaccine‐preventable diseases such as tetanus 141, 43% (difference 45.5%); pneumococcus 87, 26.5% (difference 26.5% [EN9]); measles 97, 29.6% (difference 15%); HAV 206, 63% (difference 5%); varicella 187, 57% (difference 0.9%); HBV 207, 63% (difference 0.3%) (Table 1).

**Conclusions: **Despite regular visits, HIV patients in our expert care centre were poorly immunised / protected against vaccine‐preventable diseases. The implementation of a systematic evaluation, supported by an expert CDSS generating ready‐to‐use catchup plans, demonstrated that this did not reflect vaccine refusal but failure to identify missing immunisations during routine practice.

**Table:** Abstract P219 – Table 1. Up‐to‐date immunisation/immunity against vaccine preventable disease in HIV patients

Up‐to date immunisation	At enrolment n = 328		On June 1^st^ 2018 n = 328		Difference
		n	% (95% CI^a^)	% (95% CI^a^)	% (95% CI^b^)
Tetanus	8	2.4% (1.1 to 4.7)	141	43.0% (37.6 to 48.5)	40.5% (35.2 to 46.1)
Hepatitis A	189	57.6% (52.1 to 63.0)	206	62.8% (57.3 to 68.1)	5.2% (3.0 to 8.2)
Hepatitis B	206	62.8% (57.3 to 68.1)	207	63.1% (57.6 to 68.3)	0.3% (0.0 to 1.7)
Measles	48	14.6% (11.0 to 18.9)	97	29.6% (24.7 to 34.8)	14.9% (11.3 to 19.3)
Varicella	184	56.1% (50.5 to 61.5)	187	57.0% (51.5 to 62.4)	0.9% (0.2 to 2.6)
Pneumococcus	0	0.0% (0.0 to 1.1)	87	26.5% (21.8 to 31.7)	26.5% (21.8 to 31.7)

^a^95% confidence intervals obtained with Clopper‐Pearson's method. ^b^difference in number of patients divided by 328 (95% confidence intervals obtained with Clopper‐Pearson's exact method).

## P220

### Comparison of early serological response of early syphilis to treatment with a single‐dose benzathine penicillin G between HIV‐positive and HIV‐negative patients: a cohort study

**C Yang^1^, W Liu^2^, L Chang^2^, C Wu^2^, Y Su^2^ and C Hung^2^**

^1^Department of Internal Medicine, Far Eastern Memorial Hospital, New Taipei City, Taiwan. ^2^Department of Internal Medicine, National Taiwan University Hospital, Taipei, Taiwan

**Background: **Serological response of early syphilis to treatment has been reportedly poorer in HIV‐positive patients compared with HIV‐negative patients; however, the interpretation of the published data is limited by the differences in study design, subjects with different stages of syphilis included, definition used for serological response, treatment administered and follow‐up frequency and duration. We aimed to compare the early serological response to benzathine penicillin G (BPG) during the monthly follow‐up for 3 consecutive months between HIV‐positive and HIV‐negative patients with early syphilis.

**Materials and methods: **Since January 2015, adult patients aged 20 years or older who presented with early syphilis (primary, secondary and early latent syphilis) with baseline rapid plasma regain (RPR) titers of 4 or greater were included in this prospective observational study after the patients received a single dose of BPG for early syphilis according to the STD Treatment Guidelines 2015 of US CDC. RPR titers were determined at baseline and thereafter every 4 weeks for 12 weeks, followed by every 12 weeks. Serological response was defined as decline of RPR titer by four‐fold or greater at each time point compared with baseline. Serological failure was defined as an increase of RPR titer by four‐fold or greater during follow‐up after ever achieving a decline of the titer.

**Results: **Between January 2015 and March 2018, 151 HIV‐positive and 48 HIV‐negative patients were included; all were men who have sex with men. Compared with HIV‐positive patients, HIV‐uninfected patients had more cases of secondary syphilis (52.5% vs. 32.9%, *p *=* *0.027) and fewer early latent syphilis (40.0% vs. 58.9%, *p *=* *0.048) and prior syphilis (20.0% vs. 67.8%, *p *<* *0.001). HIV‐negative patients achieved faster serological response than HIV‐positive patients: 63.0% versus 37.9% (*p *=* *0.011), 95.0% versus 72.0% (*p *=* *0.004), 95.2% versus 81.8% (*p *=* *0.061) and 96.7% versus 83.3% (*p *=* *0.047) at Week 4, Week 8, Week 12 and Week 24, respectively, in the per‐protocol analysis. In multivariate analysis to examine the factors associated with 12‐week serological response, we found that the response was statistically significantly associated with early latent syphilis (adjusted odds ratio [aOR] 0.25, 95% CI 0.07 to 0.92) and per 1‐log2 increase of RPR titer at baseline (aOR 1.01, 95% CI 1.00 to 1.01).

**Conclusions: **HIV‐negative patients had better early serological response of early syphilis to BPG than HIV‐positive patients during the first 12 weeks of follow‐up. Early latent syphilis was associated with a poorer response while a higher RPR titer was associated with a better response to BPG.

## P221

### Concurrent transmission of HCV and bacterial STI in HIV‐infected MSM

**N Wong^1^, B Wong^2^, D Chan^1^ and S Lee^1^**

^1^Stanley Ho Centre for Emerging Infectious Diseases, The Chinese University of Hong Kong, Hong Kong, China. ^2^Department of Health, Integrated Treatment Centre, Hong Kong, China

**Background: **Worldwide, sexual transmission of HCV has become widely reported in MSM, especially among those HIV infected. Its association with other bacterial sexually transmitted infections (STI) has not been well characterised.  

**Methods: **Clinical data and blood samples of HIV patients diagnosed with acute HCV infection were collected from a major HIV specialist clinic in Hong Kong. HCV genotyping was performed on HCV RNA+ samples. Concurrent STI was defined as the diagnosis of syphilis, gonorrhoea and/or chlamydia within one year of HCV diagnosis. HIV/HCV co‐infected patients with and without concurrent STI were compared in logistic regression models and Mann‐Whitney U in SPSS Statistics.

**Results: **Between 2004 and 2017, a total of 79 HIV patients were diagnosed with sexually acquired acute HCV infection. All of them were male, 75 (95%) being Chinese, and all except one were MSM. Among 44 cases with known HIV subtype, 31 (70%) were subtype B, 12 (27%) subtype CRF_01AE and one (2%) CRF_07BC. Around 70% (44/60) were infected with HCV genotype 3a, followed by 1a (18%) and 6a (5%). However, only 17 out of 27 HIV subtype B cases were in HCV genotype 3a. Thirty percent of the HCV patients gave a history of recreational use of drug for sex (chem‐fun). Overall 58 (74%) had concurrent STI: 53 (90%) with syphilis only, one (2%) with chlamydia only and five (8%) with multiple STI. HIV + MSM with concurrent STI were more likely to be diagnosed with HIV in the preceding five years (median year of diagnosis: 2013), and had HAART initiated (median year of 2013 vs. 2012) shortly afterwards. HIV + MSM with concurrent STI were more likely to be infected with HCV genotype 3a (80% vs. 53%), the latter forming a monophyletic cluster that has continued to grow. However, HIV subtype, age, year of first HCV positive, time interval between HIV and HCV diagnoses and the history of chem‐fun were not significantly different between the STI+ and STI‐ MSM.

**Conclusion: **In Hong Kong, HCV and HIV were separately transmitted through sex in co‐infected patients who contracted HIV infection largely within the last five years. Parallel emergence of STI confirmed the extensive practice of condomless sex in the HIV+ MSM community. Chem‐fun was one of the most important associated factors, which explained the clustering of HCV genotype 3a and the concurrent transmission of HCV and syphilis.

## P222

### Non‐AIDS bacterial infections are the main cause of hospital admissions in HIV‐infected patients over 2010‐2017: data from the San Paolo Infectious Diseases (SPID) cohort

**F Bai^1^, E Suardi^1^, F Parolo^1^, A Tavelli^2^, F Crippa^1^, T Bini^1^, G Marchetti^1^ and A d'Arminio Monforte^1^**

^1^Clinic of Infectious Diseases, San Paolo Hospital, University of Milan, Department of Health Sciences, Milan, Italy. ^2^ICONA Foundation, Department of Health Sciences, Milan, Italy

**Background: **We aimed to evaluate the prevalence and the clinical and viro‐immunological correlates of hospital admissions for non‐AIDS bacterial infections (INF) in HIV‐positive patients over 2010 to 2017 at the Clinic of Infectious Diseases, S. Paolo Hospital, Milan, Italy (SPID cohort).

**Material and methods**

Retrospective study including hospital admissions for any reason among HIV‐positive and HIV‐negative patients at our centre (1 January 2010 to 31 December 2017). The main cause of hospitalisation in HIV‐positive patients was grouped in: non‐AIDS defining bacterial infections (INF); AIDS‐defining illnesses (ADI); liver/gastrointestinal diseases (GI); non‐AIDS cancers; and other (cardiovascular‐renal‐genitourinary‐pulmonary‐psychiatric‐other). Each hospitalisation was placed into a single mutually exclusive category. Yearly prevalence of hospital admission in HIV‐positive and HIV‐negative patients was calculated. Chi‐square and Mann‐Whitney/Kruskal‐Wallis test were used.

**Results: **Three thousand four hundred and eighty‐eight hospitalisations were recorded at our centre over 2010 to 2017: 1056 (30%) in HIV‐positive patients (191, 18% patients at first HIV diagnosis; 786, 75% combination antiretroviral therapy (cART)‐treated patients; 73, 7% unknown therapeutic history). There was a reduced proportion of hospitalisations in HIV‐positive versus HIV‐negative patients in recent years (from 148/427, 35% in 2010 to 110/410, 27% in 2017 in HIV‐positive patients; from 279/427, 65% in 2010 to 300/410, 73% in HIV‐negative patients; *p *<* *0.0001), with a reduction in cART‐treated patients (from 120/427, 29% in 2010 to 92/410, 22% in 2017; antiretroviral‐naïve patients: from 28/427, 6% in 2010 to 18/410, 5% in 2017; *p *<* *0.0001). Three hundred and eighty‐four (36%) hospitalisations were for INF, 223 (21%) for ADI, 219 (21%) for GI, 39 (4%) for non‐AIDS cancers and 191 (18%) for other. The most frequent cause of admission were INF (304/786, 39%), followed by GI (196/786, 25%) among cART‐treated patients and ADI among cART‐naïve patients (89/191, 47%) (*p *<* *0.0001). In cART‐treated patients, from 2010 to 2013 to 2014 to 2017 admissions for INF remained stable (from 38% to 40%), non AIDS‐cancers doubled (from 3.3% to 6.2%) and ADI decreased (from 17% to 13%) (*p *=* *0.004). HIV‐positive patients admitted for INF were more commonly older, cART‐treated with undetectable HIV‐RNA and higher current/nadir CD4+ count and presented shorter length of hospital stay, compared to patients admitted for ADI. INF were represented mainly by pneumonia (206, 54%) and sepsis (71, 18%) (gastrointestinal: 30, 8%; genito‐urinary: 31, 8%; skin‐soft tissue infections: 27, 7%; other: 19, 5%). Data are presented as absolute numbers, percentages for categorical variables and median, interquartile range (IQR) for quantitative parameters. CD4+ count and HIV‐RNA were recorded at the hospital admission. Detectable HIV‐RNA was considered >40 copies/mL.

**Conclusions: **Hospitalisation among cART‐treated HIV‐positive patients declined in the recent years; INF, and in particular pneumonias, still represent the main cause of hospital admission in virally suppressed cART‐treated patients. With the ageing of HIV population, hospitalisations for non‐AIDS cancers doubled over 2010 to 2017.

## P223

### Use of recreational drugs and sexually transmitted infection (STI) diagnosis among patients attending a STI/HIV reference clinic in Rome

**A Latini^1^, M Colafigli^1^, M Frasca^1^, L Alei^1^, M Giuliani^1^, A Cristaudo^1^ and M Zaccarelli^2^**

^1^STI/HIV Unit, San Gallicano Dermatologic Institute, IRCCS, Rome, Italy. ^2^Clinical Department, National Institute for Infectious Diseases Lazzaro Spallanzani IRCCS, Rome, Italy

**Background: **To assess frequency and association with recent STI and viral hepatitis (VH) diagnosis with reported recreational drug (RD) use among clients of a reference centre in Rome.

**Methods: **Patients attended the centre for HIV treatment or STI visit between January and May 2018. Patients, after signing informed consent, self‐compiled a questionnaire concerning RD and sexual behaviours. Data about drugs used during sex were collected: crystal meth, GHB and mephedrone (3 chems) and in addition with MDMA, cocaine, ketamine, erectile dysfunction agents (EDA), poppers and steroids (9 chems). Association with STI or VH diagnosis in the past six months, HIV status, demographic and behavioural data was assessed.

**Results: **Overall, 401 patients were included (203 attending the centre for STI and 198 for HIV treatment), of them 235 (59%) were MSM, 113 (28%) hetero males (HM) and 53 (13%) females. The proportion of HIV patients was higher among MSM (76% vs. 10% among other groups). Although cannabinoids were the most widely used, cocaine, poppers, EDA, crack and mephedrone use are significantly more frequently reported by MSM, who reported more often poly‐drugs use. Among MSM, recent STI or VH diagnosis in the past six months were significantly associated with RD use, overall and for syphilis, hepatitis A and urethritis (Figure 1). Patients who reported any of 3 chems use also reported poly‐RD use (mean number of RD reported: 6.3 ± 3.0 vs. 0.8 ± 1.2 among patients who did not report use of any 3 chems). Condomless sex with non‐steady partners was significantly more frequently reported (*p *<* *0.05) by HM and females (72% and 73% respectively) versus MSM (41%); by HIV patients (65% vs. 41% by HIV neg) and by patients with STI diagnosis in the past six months (63% vs. 42% reported by patients without STI). At adjusted logistic regression, use of any of 9 chems among MSM was significantly associated with younger age, group sex and STI diagnosis, and marginally associated with sex with online partner, condomless sex was not found associated (Table 1).

**Abstract P223 – Figure 1.** Proportions of STI in the past six months among MSM by use of 9 chems (amphetamines, poppers, GHB, cocaine, ketamine, crystal, crack, EDA, steroids).

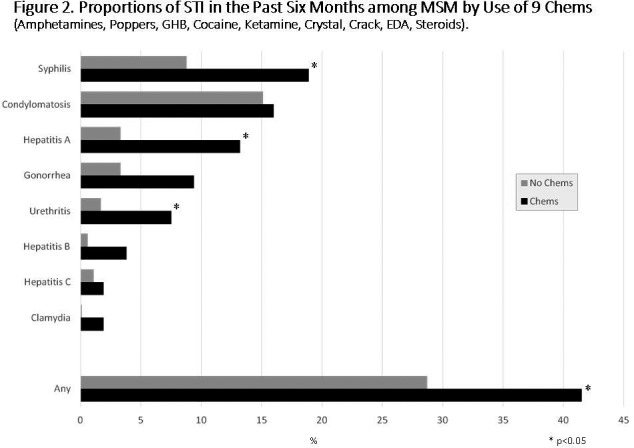

**Table:** Abstract P223 – Table 1. Adjusted logistic regression for association with use of 9 chems

	OR (95% CI)	*p* value
Hetero males	1
MSM	1.62 (0.76 to 3.45)	0.210
Females	0.92 (0.38 to 2.24)	0.862
Age (by 10 years)	0.70 (0.56 to 0.88)	0.002
No steady job	1.40 (0.69 to 2.81)	0.353
High school education	0.78 (0.46 to 1.31)	0.341
Condomless sex	0.89 (0.75 to 1.06)	0.209
Online partner	1.73 (0.99 to 3.01)	0.053
Group sex	4.06 (1.96 to 8.39)	<0.001
HIV pos	0.94 (0.48 to 1.83)	0.845
STI in the past six months	1.77 (1.02 to 3.00)	0.042

**Conclusions: **Our data suggest that recreational drug use is an increasing behaviour in Italy among MSM. The use of classic chems (crystal, mephedrone, GHB) is still limited and cocaine, poppers and erectile dysfunction agents are preferred. However, recreational drug use appears associated with higher risk sexual behaviour and generally higher risk of STI and viral hepatitis diagnosis.

## P224

### Co‐infection with syphilis in new diagnosis of HIV infection during the period 2010 to 2017: a single centre cohort

**A Bianchi, G Cuomo, C Puzzolante, A Raimondi, C Mussini and V Borghi**

Infectious Diseases, Policlinico di Modena, Modena, Italy

**Background: **An increasing number of syphilis diagnoses were reported in recent years, concurrently, in Italy, the number of new cases of HIV infection remained stable; also if raising in MSM. In this retrospective study, we evaluated the presence of syphilis in new diagnosis of HIV infection during the period 2010 to 2017 in the Modena HIV Cohort. At once, new infections with syphilis after HIV diagnosis were considered in this population.

**Material and methods**

All new diagnoses of HIV infection reported to the HIV Surveillance System of Modena during the period 2010 to 2017 were enrolled. Diagnoses of syphilis were performed using the combined Rapid Plasma Reagin‐Treponema Pallidum Haemagglutination Assay (RPR‐TPHA) test.

**Results: **During the study period 479 new diagnoses of HIV infection were reported; 355 (39.9%) were males, 187 (39%) were foreign born of Italy and 176 (36.7%) were MSM as described in Table 1. Median age was 38.0 years old (IQR 30 to 47). No statistical differences in the number of new HIV diagnoses was observed during the study period (*p* for trend = 0.598). In 45 (9.4%) patients a concomitant diagnosis of syphilis was reported; mainly in males (97.8%), Italians (84.4%) and more frequent in MSM (73.3%) despite in heterosexual (HC) (26.7%) (*p *<* *0.001). A four‐year period was used to evaluate the rate of co‐infection: 7.3% in 2010 to 2013 and 11.9% in 2014 to 2017 (*p *=* *0.089), without statistically significant increase. The only determinants to be co‐infected with HIV and syphilis in multivariate analysis were the risk of transmission (MSM vs. HC: OR 3.02 [95% CI 1.38 to 6.61], *p *=* *0.006) and gender (male vs. female: OR 9.03 [95% CI 1.12 to 72.48], *p *=* *0.038); age (OR 1.01 [95% CI 0.98 to 1.04], *p *=* *0.577) and immigrant (OR 0.71 [95% CI 0.28 to 1.81], *p *=* *0.474) were not significant. Twenty‐one out of 479 (4.4%) patients experienced a new episode of syphilis during follow‐up; in 16 people a new diagnosis was reported, in five (11.1%) with experienced syphilis infection. All of these ones were males, 16/21 (76.2%) Italians and in 76.2% of cases in MSM.

**Table:** Abstract P224 – Table 1. Characteristics of people involved in the study

Characteristic	HIV infection, N (%)	Syphilis co‐infection, N (%)
Total	479	45
Male	355 (69.9)	44 (97.8)
Female	144 (30.1)	1 (2.2)
Age	38.0 (30.0 to 47.0)	41.0 (28.0 to 54.0)
Italian	292 (61.0)	38 (84.4)
Foreign born	187 (39.0)	7 (15.6)
MSM	176 (36.7)	33 (73.3)
Heterosexual	303 (63.3)	12 (26.7)

**Conclusion: **The study shows that the number of diagnoses of HIV infection has remained stable in the last years, and a reasonable proportion of diagnosis of co‐infection with syphilis has not changed, particularly it occurred about in one‐fifth of MSM. These conditions show a low perception of risk transmission of both infections in MSM. Additionally, HIV‐infected patients seem not to reduce their sexual risk behaviour as shown in syphilis re‐infection.

## P225

### Severe bacterial infections in HIV‐infected injecting drug users from a Romanian health care facility

**I Ianache^1^, I Popa^1^, R Horghidan^1^, E Ceausu^2^ and C Oprea^2^**

^1^Infectious Diseases, Victor Babes Hospital, Bucharest, Romania. ^2^Infectious Diseases, Carol Davila University of General Medicine and Pharmacy, Victor Babes Hospital, Bucharest, Romania

**Background: **During last decade, Romania faced an alarming increase in the number of HIV‐infected injecting drug users (IDUs), often diagnosed with severe bacterial infections, with an important economic impact. The aim of our study was to assess the prevalence of bacterial infections in HIV‐infected IDUs, admitted at a tertiary health care facility and to describe their socio‐economic, clinical and immunological status.

**Methods: **Prospective study on HIV‐infected IDUs, diagnosed with bacterial infections at Victor Babes Hospital, Bucharest, between January 2016 and December 2016. Statistical analysis was performed using SPSS Statistics v.20.

**Results: **Out of 579 IDUs in active care, 149 (25.7%) were diagnosed with 247 bacterial infections (prevalence 42.6%). A total of 60 (40.2%) were diagnosed with multiple infections. HIV and bacterial infections were diagnosed simultaneously in 31 (20.8%) patients and 54 (36.2%) IDUs had recurrent bacterial infections. The majority, 126 (84.5%), were male IDUs, with median age of 30 years (IQR 25 to 34), who injected most often 71.8% a combination of heroin and newer psychostimulant drugs, for a median time of 10 years (IQR 25 to 30). Median CD4 cell count/µL and median HIV viral load (log10 copies/mL) at diagnosis of bacterial infection were 149 (IQR 37 to 377) and 4.7 (IQR 3.5 to 5.5), respectively. Almost all patients, 147 (98.6%), were co‐infected with HCV, HBsAg being positive in 17 (11.4%). The most common bacterial infections were pulmonary tract infections 91 (36.8%), tuberculosis (TB) 65 (26.3%), skin/soft tissues infections (SSTIs) 35 (14.1%), sepsis and right‐sided endocarditis 8 (3.2%) and others 56 (22.6%). The most frequent isolated pathogens were *Mycobacterium tuberculosis* (44 cases) and methicillin‐resistant *Staphylococcus aureus* (MRSA) (19). MRSA was responsible for all three cases diagnosed with endocarditis and one with sepsis, while *Klebsiella pneumoniae* was isolated in two patients with sepsis. Drug‐resistant TB was identified in 11 IDUs, five multi‐drug resistant and six with extensive‐drug resistance. There was no statistically significant difference between the types of bacterial infections in relation to the immunological status at diagnosis (Table 1). SSTIs were more often recurrent (*p *=* *0.001). The overall mortality rate was 13.7%, being higher in patients with drug‐resistant TB (*p *<* *0.0001).

**Table:** Abstract P225 – Table 1. Laboratory characteristics and outcomes in HIV‐infected IDUs diagnosed with severe bacterial infections

	Total bacterial infections (n = 247)	Pulmonary tract infections (n = 91)	Tuberculosis (n = 65)	SSTIs (n = 35)	Other infections^a^ (n = 56)	*p* value
Immuno‐virological status at HIV diagnosis						
Median CD4 (cells/µL), median (IQR)	265 (42 to 525)	318 (72 to 552)	79 (25 to 448)	343 (139 to 484)	316 (40 to 498)	0.78
Median HIV viral load (log10 copies/mL), median (IQR)	5.2 (4.6 to 5.7)	5.1 (4.5 to 5.5)	5.3 (4.6 to 5.7)	5.4 (5.0 to 5.7)	5.3 (4.6 to 5.6)	0.53
Immuno‐virological status at diagnosis of bacterial infection						
Median CD4 (cells/µL), median (IQR)	149 (77 to 377)	180 (43 to 407)	49 (16 to 202)	90 (19 to 401)	94 (35 to 335)	0.07
Median HIV viral load (log10 copies/mL), median (IQR)	4.7 (3.5 to 5.5)	4.6 (3.5 to 5.2)	5.2 (4.3 to 5.7)	4.8 (4.4 to 5.4)	5.2 (4.3 to 5.8)	0.46
Median nadir CD4 (cells/µL), median (IQR)	77 (21 to 267)	146 (34 to 335)	33 (12 to 82)	73 (21 to 168)	86 (22 to 153)	0.26
Mortality rate, n (%)	34 (13.7)	7 (7.6)	22 (33.8)	3 (8.5)	5 (8.9)	<0.0001
Median survival (months), median (IQR)	16.9 (13.4 to 21.5)	17.5 (14.0 to 22.1)	16.2 (6.7 to 21.2)	18.0 (15.0 to 21.8)	16.6 (3.1 to 20.4)	<0.0001

^a^including sepsis and endocarditis.

**Conclusion: **The prevalence of bacterial infections in HIV‐positive IDUs was high and associated with severe immunosuppression. Pulmonary tract infections, TB and SSTIs were the most frequent. IDUs diagnosed with severe pulmonary infections, drug‐resistant TB and sepsis had poor outcome, with high mortality rates.

## P226

### Use of dolutegravir in women of childbearing potential: a local response to preliminary data suggesting higher incidence of neural tube defects in women conceiving on dolutegravir‐based regimens

**G Haidari, P Farrugia, C Williams, A Mukela, D Chilton and A Grant, R Simons**

HIV, Guy's & St Thomas’ NHS Foundation Trust, London, UK

**Background: **Recent preliminary data from a birth surveillance study in Botswana has reported a 0.9% incidence of neural tube defects (NTDs) amongst infants born to mothers on dolutegravir (DTG)‐based ART compared to a 0.1% incidence in those on alternative regimens. Based on this early data BHIVA (plus other agencies) recommend a conception and contraception review of all women under 50 years on DTG.

**Materials and methods: **Women on DTG‐based ART under 50 years were identified by our pharmacy team. For each patient clinic notes were reviewed with regards to pregnancy status, contraception and conception plans. A clinical alert was placed on all electronic patient records with a clear plan for clinicians to offer contraception or a switch in ART if appropriate. For pregnant patients, those planning pregnancy or those not wishing to receive letters, patients were contacted directly for discussion. A letter was drafted by the antenatal team outlining the preliminary data and inviting women to clinic for further discussion.

**Results: **One hundred and fifteen women were identified with a median age of 38 years. Of these 20 women (17%) were on effective contraception (pill/patch/depo/coil), with two patients having had tubal ligation and four hysterectomies. There were six post‐menopausal woman in this cohort. Sixty‐nine women (60%) were documented not to be using contraception. There was no documentation for four patients. Of note six patients in this cohort had already conceived and delivered on DTG with no reported foetal abnormalities that we are aware of. Two women were already pregnant on DTG and in the second trimester. Eighteen women (16%) were actively recalled by phone either due to clear documentation of an imminent pregnancy or documentation they did not wish to receive letters from our department. All other women received the letter outlined above.

**Conclusions: **Our clinic was able to identify all women of childbearing potential on DTG‐based therapy in a timely manner, in line with current BHIVA recommendations. As this is new data, recall of our cohort is an ongoing process and in due course we hope to offer the majority either effective contraception or a switch in ART if appropriate. This work highlights the need for contraceptive history taking in women of childbearing potential and will be submitted as a quality improvement project, enabling us to better address the contraceptive needs in this population.

## P227

### Assessment of monocyte activation and systemic inflammation markers in HIV‐positive opioid users

**A Kholodnaya^1^, K So‐Armah^2^, D Cheng^3^, N Gnatienko^4^, G Patts^3^, J Samet^5^, M Freiberg^6^ and D Lioznov^7^**

^1^Infectious Diseases and Epidemiology, Saint Academician I.P. Pavlov First St. Petersburg State Medical University, St Petersburg, Russian Federation. ^2^Clinical Addiction Research and Education Unit, Boston University School of Medicine, Boston, MA, USA. ^3^Biostatistics & Epidemiology Data Analytics Centre, Boston University School of Public Health, Boston, MA, USA. ^4^Boston Medical Center, Boston, MA, USA. ^5^Clinical Addiction Research and Education Unit, Boston University School of Public Health, Boston, MA, USA. ^6^Cardiovascular Medicine Division, Vanderbilt University Medical Center, Nashville, TN, USA. ^7^Department of Infectious Diseases and Epidemiology, Academician I.P. Pavlov First St. Petersburg State Medical University, St Petersburg, Russian Federation

**Background: **With increased life expectancy for treated HIV‐positive patients non‐AIDS events and substance use have become especially relevant sources of morbidity and mortality. Chronic inflammation is thought to be a driver of non‐AIDS events. We hypothesise that opioid use increases intestinal permeability and bacterial translocation from the gut into blood and leads to increased systemic inflammation and increased morbidity and mortality. Our primary objective was to investigate the association between opioid use and soluble CD14 (sCD14), a biomarker of monocyte activation that indicates the presence of components of gram‐negative bacteria in blood. We also assessed the association of opioid use with inflammation (interleukin‐6 [IL‐6]) and altered coagulation (D‐dimer).

**Methods: **We analysed data from the Russia ARCH study – an observational cohort of 351 HIV‐positive antiretroviral therapy‐naive individuals followed over two years. Plasma levels of sCD14 (primary outcome), IL‐6 and D‐dimer (secondary outcomes), HIV viral load and CD4 count were evaluated at baseline, 12 and 24 months. Data on opioid use (the main independent variable) were collected through self‐report. Participants were categorised into three groups according to their history of opioid use: (1) current opioid use – last opioid use within past 30 days (n = 121); (2) prior opioid use – no use in past 30 days (n = 186); 3) never opioid use (n = 44). Linear mixed effects models (adjusting for age, gender, body mass index, HIV viral load, years since first HIV‐positive test, alcohol consumption, diarrhoea and use of NSAIDs) were used to evaluate the associations between opioid use and the biomarkers. Variables representing concomitant liver disease (i.e. liver fibrosis, hepatitis C, hepatitis B) were explored as potential effect modifiers.

**Results: **In adjusted models, compared to never opioids users, sCD14 levels were significantly higher among current users (adjusted mean difference 259.5 ng/mL [95% CI 77.0 to 441.9], *p *=* *0.008) but not prior users (adjusted mean difference 116.7 [−54.4 to 287.7], *p *=* *0.170). IL‐6 and D‐dimer were higher among those with current and prior opioid use (Table 1).

**Table:** Abstract P227 – Table 1. Association between opioid use category and plasma concentrations of sCD14, IL6 and D‐dimer. Linear mixed effects model

	sCD14 adjusted mean difference (95% CI)	IL‐6 adjusted ratio of means^a^ (95% CI)	D‐dimer adjusted ratio of means^a^ (95% CI)
Current opioid use (within past 30 days)	259.5 (77.0 to 441.9) *p *=* *0.008	2.130 (1.576 to 2.879) *p *<* *0.001	2.062 (1.519 to 2.799) *p *<* *0.001
Prior opioid use	116.7 (−54.4 to 287.7) *p *=* *0.170	1.333 (1.005 to 1.767) *p *=* *0.046	1.682 (1.257 to 2.250) *p *=* *0.001
Never opioid use	Reference group	Reference group	Reference group

We did not detect interaction effects from liver disease. ^a^represents the ratio of means after back transformation from natural log scale.

**Conclusions: **Opioid use in HIV‐positive participants is associated with an increase in monocyte activation and higher inflammatory response. The underlying mechanism for this association is not known. However, it is possible that opioid use may lead to intestinal permeability and chronic systemic inflammation.

## P228

### Impact of CMV on liver progression in HIV/HCV/CMV co‐infected patients in a large cohort of HIV‐infected patients

**S Vita^1^, P Lorenzini^2^, M Lichtner^3^, M Giulia^4^, C Mastroianni^5^, A Bandera^6^, A Di Biagio^7^, C Pinnetti^8^, A Calcagno^9^, A Castagna^10^, A Antinori^11^ and A d'Arminio Monforte^12^, on behalf of Icona Foundation Study Group**

^1^Public Health and Infectious Disease, SM Goretti Hospital, Rome, Italy. ^2^Clinical Department, National Institute of Infectious Diseases Lazzaro Spallanzani, Rome, Italy. ^3^Public Health and Infectious Disease, Sapienza University of Rome, Polo Pontino, Rome, Italy. ^4^Department of Health Sciences/Clinic of Infectious Diseases, University of Milan, Rome, Italy. ^5^Public Health and Infectious Disease, Sapienza University of Rome, Rome, Italy. ^6^Azienda Socio Sanitaria Territoriale di Monza, San Gerardo Hospital, University of Milano‐Bicocca, Rome, Italy. ^7^Clinic of Infectious Diseases, Ospedale Policlinico San Martino, Genova, Italy. ^8^Division of Infectious Diseases, National Institute of Infectious Diseases Lazzaro Spallanzani, Rome, Italy. ^9^Department of Medical Sciences, University of Turin, Amedeo di Savoia Hospital, Turin, Italy. ^10^Infectious Diseases Department, IRCCS San Raffaele, Milan, Italy. ^11^HIV/AIDS Department, National Institute of Infectious Diseases Lazzaro Spallanzani, Rome, Italy. ^12^Department of Health Sciences, ASST Santi Paolo e Carlo, S. Paolo Hospital, University of Milan, Milan, Italy

**Background: **CMV seropositivity has been linked with severe non‐AIDS events/deaths and subclinical carotid artery disease in HIV‐infected individuals [1,2]. CMV/HCV/HIV co‐infected women have been shown to display higher CMV IgG levels versus HIV mono‐infected [3]; likewise HIV/HCV/CMV co‐infected patients have higher HCV viral load, suggesting a persistent interaction between viruses. We evaluated prevalence and impact of CMV‐Ab on liver progression and AIDS events in HIV/HCV/CMV co‐infected subjects in the ICONA cohort.

**Materials and methods:** ****We included patients from ICONA with ≥1 CMV‐IgG and HCV‐Ab available, at least one follow‐up visit and no end stage liver disease (ESLD) at baseline. Three different endpoints: (1) first of two consecutive Fib‐4 ≥ 3.25, 2) ESLD, 3) AIDS‐defining event/death. Subjects were followed from first CMV available test (baseline) to first event/last observation/first negative HCV‐RNA. Three separate Cox regression models were used.

**Results: **Eight thousand and ninety‐eight patients had known baseline CMV IgG serostatus, of these 1994 (25%) were HCV‐Ab positive and included in the analysis; 82% (1633) were HIV/CMV/HCV co‐infected, while 18% (361) were HIV+/CMV‐/HCV+. Two populations differed at baseline only for CD8 count (median 823 in CMV‐ vs. 903 in CMV+, *p *=* *0.007) and nadir CD4 (<200 70.4% in CMV‐ vs. 76.4% in CMV+, *p *=* *0.007). No differences were observed for liver‐related parameters (AST, ALT, Fib‐4, APRI). When considering Fib‐4 index ≥3.25 endpoint on 1490 subjects with baseline Fib‐4 < 3.25, ESLD events were recorded on 13,741 PYFU (IR 0.5 per 100 PYFU [95% CI 0.4 to 0.7]). No differences were found according to CMV infection (Figure 1). Protective factors for Fib‐4 progression were female sex and homosexual as mode of HIV transmission versus IVDU, while older age and higher Fib‐4 index at baseline were associated with higher risk of the outcome. AIDS‐defining events or AIDS‐related death occurred in 207 patients (180 and 27 respectively) over 12,973 PYFU (IR 1.6 per 100 PYFU [95% CI 1.4 to 1.8]). Patients with CMV infection presented not significant higher risk respect those without infection. Females, CDC stage C and baseline Fib‐4 > 3.25 were associated with higher risk of the outcome, while reduced risk was associated to baseline CD4 > 500 cells/mm^3^ and nadir CD4 > 200 cells/mm^3^.

**Conclusion: **In our study population of HCV/HIV co‐infected subjects, CMV IgG positivity does not seem to be associated with a higher risk of liver and AIDS progression. The current use of DAA could allow us to evaluate the impact of HCV eradication in this specific population.

**Abstract P228 – Figure 1.** KM analysis for the three endpoints: Fib‐4 index ≥3.25, end stage liver disease, AIDS events/death.

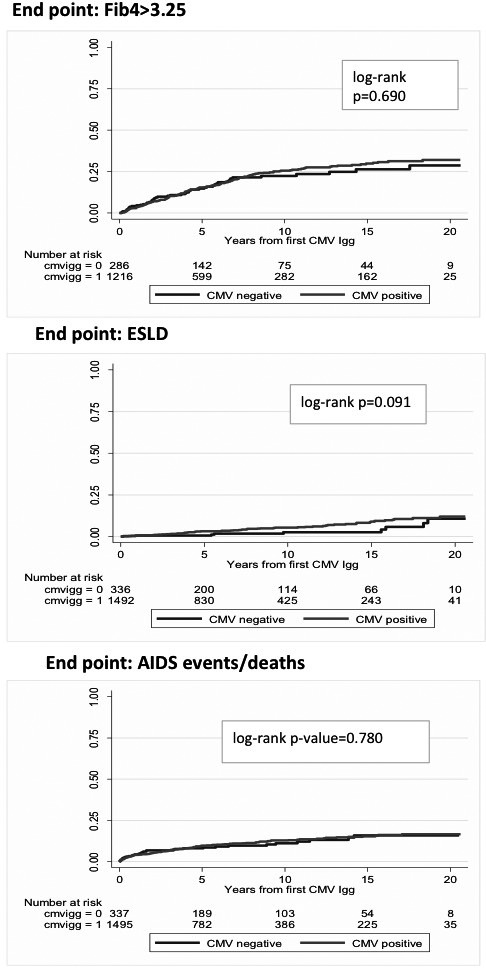

**References**

[1] Lichtner M, Cicconi P, Vita S, Cozzi‐Lepri A, Galli M, Lo Caputo S, et al. for the Icona Foundation Study. Cytomegalovirus coinfection is associated with an increased risk of severe non‐AIDS‐defining events in a large cohort of HIV‐infected patients. J Infect Dis. 2015;211:178‐86.

[2] Hsue PY, Hunt PW, Sinclair E, Bredt B, Franklin A, Killian M, et al. Increased carotid intima‐media thickness in HIV patients is associated with increased cytomegalovirus‐specific T‐cell responses. AIDS 2006;20:2275‐83.

[3] Kuniholm MH, Parrinello CM, Anastos K, Augenbraun M, Plankey M, Nowicki M, et al. Hepatitis C viremia is associated with cytomegalovirus IgG antibody levels in HIV‐infected women. PLoS One. 2013;8:e61973.

## P229

### The importance of serological testing and risk factors of measles, mumps, rubella and VZV among HIV‐infected adults in Istanbul, Turkey during measles outbreak in Europe

**O Altuntas Aydin^1^, S Senoglu^1^, H Kumbasar Karaosmanoglu^1^, Z Yesilbag^1^ and A Aydin^2^**

^1^Infectious Diseases and Clinical Microbiology, University of Health Sciences, Bakirkoy Dr Sadİ Konuk Training and Research Hospital, Istanbul, Turkey. ^2^Neurosurgery, University of Health Sciences, FTR Training and Research Hospital, Istanbul, Turkey

**Background: **Measles, mumps, rubella (MMR) and varicella zoster virus (VZV) infection can cause serious diseases and complications in HIV/AIDS patients. Due to successful vaccination programmes measles has become neglected in Turkey. Even though welcoming more than 3 million immigrants from Syria in the last seven years, measles outbreak has not been encountered in our country. However, recent outbreaks of measles are ongoing in Europe, especially in Italy, France, Romania and Turkey's neighbourhood Greece [1]. The objective of this study was to determine of MMR and VZV seronegativity and the risk factors associated being seronegative in HIV‐infected adults in Istanbul, Turkey.

**Materials and methods: **All HIV‐infected patients in our cohort who had MMR and VZV serological tests performed between January 2016 and May 2018 were retrospectively identified. Sera were tested for MMR and VZV IgG using commercial immunoassays. Age, gender, CD4 cell counts, MMR and VZV IgG serological results were evaluated from the records. Statistical analyses were performed by using SPSS Statistics v.21.0. Categorical variables were compared using chi‐square test and Fisher's exact test.

**Results: **MMR and VZV IgG serologies were available in 415 of 712 patients in active follow‐up (58.2%). Out of 415 naive patients 88.6% were men and median age was 35.6 years (range 18 to 67). Seronegativity was found in 3.9% for measles, 4.8% for rubella, 8.2% for mumps and 3.4% for VZV. Among 18.7% measles, 23.5% mumps, 35% rubella and 7.1% varicella IgG seronegative patients CD4 cell count was less than 200/mm^3^. Being born after 1983 is strongly associated with seronegativity against measles (*p *=* *0.00) and a nadir CD4 cell count below 200/mm^3^ is independently associated with rubella seronegativity (*p *=* *0.013).

**Conclusions: **There is high need for MMR vaccination in HIV‐infected patients in Turkey born in 1983 or later. Measles continues to spread across Europe as the vaccination coverage in many European countries is suboptimal. Systematic measles antibody screening and vaccination should be performed in HIV‐infected individuals to prevent serious disease and complications in the era of vaccination.

**Reference: **[1] European Centre for Disease Prevention and Control (ECDC). Monthly measles and rubella monitoring report, April 2018. Stockholm: ECDC; 2018.

## P230

### Prescription patterns of comedication and potential for drug‐drug interactions with ART in HIV patients in a retrospective claims database in Germany: implications for adequate HIV treatment selection

**S Lopes^1^, K O'Day^2^, J Van Stiphout^3^, Y Punekar^1^, M Radford^4^ and J Haas^5^**

^1^Global Health Outcomes, Viiv Healthcare, London, UK. ^2^Global Health Economics, Xcenda LLC, Palm Harbor, FL, USA. ^3^Global HEOR and Market Access, Xcenda Switzerland GmbH, Bern, Switzerland. ^4^Health Outcomes Europe, Viiv Healthcare, London, UK. ^5^Real World Evidence, Xcenda GmbH, Hannover, Germany

**Background: **ART has increased life expectancy of PLWHIV, with consequent increase in the prevalence of comorbidities, and polypharmacy. This in turn increases the risk of drug‐drug interactions (DDIs) and contraindications (CIs) with ART, which are amongst the main reasons for changing ART. Different first‐line recommended ART regimens have different DDI and CI profiles. The aim of this study was to estimate the rate of potential DDIs and CIs of real‐world prescribed non‐ART comedication, with first‐line recommended ART in PLWHIV in Germany.

**Materials and methods:** ****A retrospective, cross‐sectional cohort study design was used to collect non‐ART comedication prescription data from a representative sample of German health insurance claims from the InGef research database, in 2016. Adult patients with an ICD‐10 diagnosis code for HIV and who were prescribed any ART during 2016 were included in the analysis. Patients were stratified by gender, age, comorbidities and time on ART. Prescribed comedications were used to estimate the rate of potential for DDIs and CIs for each recommended first‐line ART per patient for the year of 2016, based on criteria from an established DDI reference database (www.hiv‐druginteractions.org).

**Results: **Two thousand six hundred and eighty patients were included in the study, 86% were male, with mean age of 45.6 years, 34% were aged 50 years or over, and 29% had been on ART for at least five years. A total of 8025 prescribed comedications were counted in 2016. Overall, approximately one out of seven patients could potentially have a DDI/CI when prescribed raltegravir + emtricitabine/tenofovir alafenamide (FTC/TAF), one in six patients could potentially have a DDI/CI when prescribed with dolutegravir (DTG) /abacavir/lamivudine (ABC/3TC) or DTG + FTC/TAF; and approximately one in five patients could potentially have a DDI/CI when prescribed bictegravir/FTC/TAF. Boosted regimens presented the highest potential for DDIs/CIs (Table 1).

**Table:** Abstract P230 – Table 1. Rate of potential drug‐drug interaction or contraindication with each first‐line recommended ART regimen per patient per year

Regimens	Overall population (n = 2680)
Dolutegravir/abacavir/lamivudine	0.159
Elvitegravir/cobicistat/emtricitabine/tenofovir alafenamide	0.851
Bictegravir/emtricitabine/tenofovir alafenamide	0.218
Dolutegravir+emtricitabine/tenofovir alafenamide	0.174
Darunavir/cobicistat/emtricitabine/tenofovir alafenamide	1.014
Raltegravir+emtricitabine/tenofovir alafenamide	0.150
Rilpivirine/emtricitabine/tenofovir alafenamide	0.422

**Conclusions: **Results indicate that comedication with potential DDIs/CIs with first‐line recommended ART are frequently prescribed among real‐world HIV patients in Germany. The potential risk of DDIs and CIs varies greatly by ART regimen. As patients grow older, understanding each ART regimen's DDI profile, alongside the comorbidities and comedications of the HIV population, can help inform treatment decisions, based on potential risks and benefits of available recommended ART and may avoid future complications.

## P231

### Syphilis and HIV: characteristics of the co‐infection in patients newly diagnosed with HIV

**I Abreu, P Palma, L Graça, R Filipe, R Ruas, E Branco, C Caldas, C Piñeiro, J Soares, M Tavares, R Serrão and A Sarmento**

Infectious Diseases, Centro Hospitalar São João, Porto, Portugal

**Background: **The prevalence of syphilis continues to rise in Europe, particularly in groups with high risk sexual behaviour, like MSM and sex workers [1,2]. It is also increased in people living with HIV. Furthermore, in these patients, a case of untreated syphilis can have direct implications on the course of this disease, leading to increase in plasma HIV viral load and decrease of T CD4+ lymphocytes [3]. Our objective with this study was to identify every case of syphilis in newly diagnosed HIV patients followed in our outpatient clinic in Centro Hospitalar São João, while trying to establish possible risk factors for the acquisiton of syphilis.

**Materials and methods: **Retrospective observational study. We reviewed all patients that were newly diagnosed with HIV in our patient clinic between January 2015 and December 2017. In our clinic, we use the Treponema pallidum particle agglutination assay (TPPA) for the initial screening of syphilis. If positive, we do the Venereal Disease Research Laboratory (VDRL) test. For discordant results, we confirm the diagnosis with the fluorescent treponemal antibody absorption test (FTA‐ABS).

**Results: **We identified 217 patients: 168 (77.4%) were men; the median age was 39 years. The mode of HIV exposure was heterosexual in 108 cases (49.8%); MSM in 97 (44.7%); intravenous drug use in five cases (2.3%) and unknown in seven cases (3.2%). There were 59 cases (27.2%) of syphilis: 22 (37.3%) were past treated infections and 37 (62.7%) were unknown and untreated infections (54.1% late latent syphilis; 13.5% early latent syphilis; 18.9% primary syphilis, 0.08% neurosyphilis; 0.03% retinitis; and 0.03% secondary syphilis). When we compared patients with syphilis and patients without, we found a statistically significant association between being an MSM and being diagnosed with syphilis (*p *=* *0.04). The percentage of cases diagnosed per year remained stable during the period of time we studied: 30% in 2015, 20.2% in 2016 and 32.8% in 2017.

**Conclusion: **The overall prevalence of syphilis in our group of HIV patients was of 27.2%, with a frequency that has remained stable through the last three years. The association between this diagnosis and MSM reflects the need to develop better campaigns on high risk groups for the adoption of safer sexual practices and periodic screening of sexually transmitted diseases. Further studies in our clinic intend to see which of these patients got re‐infected with syphilis and if we can use the syphilis screening as a marker for risk of other sexually transmitted diseases.

**References**

[1] European Centre for Disease Prevention and Control (ECDC). Syphilis. In: ECDC. Annual epidemiological report for 2015. Stockholm: ECDC; 2017.

[2] Mulhall BP, Wright ST, De La Mata N, Allen D, Brown K, Dickson B, et al. Risk factors associated with incident sexually transmitted infections in HIV‐positive patients in the Australian HIV observational database: a prospective cohort study. HIV Med. 2016;17:623‐30.

[3] Kalichman SC, Pellowski J, Turner C. Prevalence of sexually transmitted co‐infections in people living with HIV/AIDS: systematic review with implications for using HIV treatments for prevention. Sex Transm Infect. 2011;87:183‐90.

## P233

### How are HIV patients dying?

**F Duarte, J Laranjinha, R Correia de Abreu and I Neves**

Infectious Diseases, Hospital Pedro Hispano, Matosinhos, Portugal

**Background: **The introduction of HAART remains a hallmark in increasing the average life expectancy and the quality of life in HIV‐infected patients. However, diagnostic delay still is a reality with a high impact on mortality.

**Objectives: **To assess the causes of mortality in HIV‐infected patients with a follow‐up at a 330‐bed tertiary hospital in the last 20 years, after the introduction of HAART.

**Methods: **Two hundred and three deaths were extracted from a hospital database of 1230 HIV‐infected patients followed at the infectious diseases department, between 1997 and 2017. Out of these 203 patients, 113 were studied based on the attending physician (by the updated registration probability) and 11 were excluded (cause of death not registered). Epidemiological, immunological, virological and therapeutic aspects were evaluated at the time of diagnosis and of death.

**Results: **One hundred and two HIV‐infected patients (77 males) were evaluated. At diagnosis, with an average of 38 ± 13 years old, intravenous drug use being the major risk of transmission (62%), only one case of HIV type 2, 79% with the diagnosis of a concomitant AIDS‐defining illness and 62% were late presenters (CD4+ T lymphocyte count <200/mL). At the time of death, they were on average 45 ± 11 years old, 45 patients died due to an AIDS‐defining illness with an average CD4+ T lymphocyte count of 199/mL (70% in C3 CDC stage), 12 of those cases being lymphomas. Non‐AIDS‐defining illnesses were the cause of death of 57 patients, 26 (46%) dying of infectious diseases, 16 (28%) of neoplasms, five (9%) of cardiovascular events, 10 (18%) of violent deaths. In this group, the CD4+ T lymphocyte average count was 354/mL. The duration of antiretroviral therapy was less than 48 weeks in 27% of the overall sample. In late presenters, 52% of deaths were attributable to AIDS‐defining illnesses (21% died in the first year), with an average infection time of 6 ± 5 years.

**Conclusion: **Our study shows that the longer life expectancy in HIV‐infected patients leads to an increase in mortality related to non‐AIDS‐defining illnesses, such as oncological and cardiovascular diseases. These findings are in line with previous studies and raise awareness for the need of better cancer screening protocols and more efficacious management of vascular risk factors. Despite upcoming better treatment options, in our sample 62% of patients were late presenters, in which AIDS‐defining illness was a very prevalent cause of death (89%). Therefore, more effective HIV screening measures are also lacking, such as optimising patient adherence in healthcare system.

## P234

### Inflammatory bowel diseases: a hidden comorbidity in people living with HIV?

**V Guardigni, E Scaioli, S Coladonato, G Fornaro, G Piazza, L Badia, A Belluzzi, S Vitale, M Salice, F Rizzello, P Gionchetti, L Calza and G Verucchi**

Department of Medical and Surgical Sciences, University of Bologna, Bologna, Italy

**Background: **The interplay between HIV infection and inflammatory bowel diseases (IBDs) is not clear, although events like cell‐mediated immune responses and microbial translocation occur in both conditions. Prevalence of IBDs in HIV population is unknown. It has been suggested that HIV‐related immunosuppression may reduce IBD activity, but data on HIV patients who have been diagnosed with IBDs are still lacking. The aim of this study was to determine the prevalence of IBDs in our HIV population and describe the characteristics of HIV+ patients with IBDs.

**Materials and methods: **We conducted a retrospective study, including all the HIV+ patients on ART with a definitive diagnosis (histologically confirmed) of ulcerative colitis (UC) or Crohn's disease (CD), at our HIV clinic. Pharmacy records for IBD drugs were examined for all HIV+ men and women in the last 10 years to individuate eligible individuals.

**Results: **We identified 17 HIV+ men with IBDs (0.72% of the overall HIV population on ART at our clinic), aged 44 years (IQR 44 to 49.5), with undetectable HIV‐RNA and good immune response (median CD4+ T cells 826/mm^3^). Seventy‐seven percent were MSM. Median CD4+ T cells nadir was 369/mm^3^, and only four individuals had CDC class C disease. In most cases (82%), IBD identification followed (for years) HIV diagnosis. All the included patients had UC (47% proctitis), no CD was observed. Median age at RCU diagnosis was 37 years. Four patients had an mild active disease (partial Mayo score 2) and six were not taking drugs for UC, at the time of this study. Three patients had undergone surgery for UC. Median duration of follow‐up since IBD diagnosis was 7.5 years. Forty‐one percent had had at least a UC relapse over time (Table 1). Median relapse rate was 0.041/year of follow‐up.  No HIV or ART‐related factor was associated to UC extent or relapse rate.

**Table:** Abstract P234 – Table 1. Characteristics of HIV population with IBD diagnosis, at the time of the study

Characteristics of population	N 17
Male sex, n (%)	17 (100%)
Age, years (median IQR)	44 (37 to 49.5)
MSM, n (%)	13 (77%)
CDC class C (%)	4 (24%)
CD4‐T cells nadir (/mm^3^)	396 (209 to 586)
Years since HIV diagnosis, median (IQR)	8 (5.5 to 18)
CD4‐T cells (/mm^3^)	826 (589 to 973)
HIV <50 copies/mL, n (%)	17 (100%)
PI‐containing ART regimen, n (%)	7 (41.2%)
HCV Ab positive, n (%)	8 (47%)
Years since IBD diagnosis, median (IQR)	7.5 (2.25 to 10.5)
Ulcerative colitis, n (%)	17 (100%)
UC extent	
Proctitis, n (%)	8 (47%)
Distal colitis, n (%)	1 (6%)
Pancolitis, n (%)	7 (41%)
N/A	1 (6%)
Pharmacological treatment for IBD	
No treatment, n (%)	6 (35%)
Steroids, n (%)	1 (6%)
Mesalazine, n (%)	8 (47%)
Mesalazine plus steroids, n (%)	2 (12%)
Immunosuppressive drugs, n (%)	0

**Conclusions: **Prevalence of IBDs in our HIV population was low. Males and MSM might be at higher risk for UC. Diagnosis of IBDs might be often missed or delayed in people living with HIV (PLWHIV) since intestinal symptoms are usually attributed to HIV itself, ART side effects or opportunisms. Since IBD misdiagnosis may represent a risk, new diagnostic tools and algorithms as well as deeper insight in therapeutic options for IBDs are needed in PLWHIV. Main limitation of this study is its retrospective nature.

## P235

### Benefit of an annual screening of comorbidities for people living with HIV in a day‐care hospital

**L Bidouze^1^, M Michaud^2^, F Catros^2^, S Ancellin^2^, A Bicart See^2^, D Garipuy^2^, C Fourcade^2^, E Bonnet^2^, M Obadia^2^, E Pouchelon^3^, S Fritsch^4^, F Martini^2^, V Violton^2^, T Charasson^5^, L Gautié^6^ and F Gaches^2^**

^1^General Practice Department, Toulouse University, Toulouse, France. ^2^Department of HIV and Viral Hepatitis, Joseph Ducuing Hospital, Toulouse, France. ^3^Department of Cardiology, Joseph Ducuing Hospital, Toulouse, France. ^4^Department of General and Visceral Surgery, Joseph Ducuing Hospital, Toulouse, France. ^5^Department of Obstetrics and Gynecology, Joseph Ducuing Hospital, Toulouse, France. ^6^Department of Pharmacy, Joseph Ducuing Hospital, Toulouse, France

**Background: **French Guidelines recommends that people living with HIV should be tested annually for comorbidities and re‐evaluation of treatment in a day hospitalisation course.

**Materials and methods: **We conducted a retrospective, descriptive and monocentric study, whose main objective was to detect the HIV complications, during the annual day‐care hospitalisation. Data were collected using a standardised case report form (CRF).

**Results: **Data of 128 patients have been analysed over one year. Sex ratio was 0.67, mean age 49.1 years old. Patients were at the AIDS stage in 17% and 36% had a CD4 nadir <200/mm^3^. Hepatitis C co‐infection was noted for 15% of patients. Mean CD4 was 668/mm^3^. One hundred and twenty‐six patients (98%) received antiretroviral therapy, triple therapy in 88%, bitherapy in 8% (10 patients) and a protease inhibitor monotherapy in 4% (five patients). Global acceptance of the treatment based on a scale of 1 to 10, had a mean of 9.3. Vaccination coverage was incomplete in 68% (46% for *diphtheria‐tetanus‐pertussis,* 25% for *influenza*, 19% for *pneumococcus*). Regarding the level of cardiovascular Framingham risk score, it was very low for 23% of patients, low for 22%, intermediate for 22%, high for 27% and very high for 6%. In addition, 16% of patients met criteria for metabolic syndrome. A complementary cardiological assessment was therefore indicated in 46% of cases, including a stress test in 19%. Seven percent of patients who had a therapeutic indication for a statin did not receive it. Regarding renal function, 37% of patients had a CKD‐EPI clearance <90 mL/min and 5% had a clearance <60 mL/min. Proteinuria/creatininuria was >300 mg/g in 34% of cases. Forty‐eight patients had a proctological consultation and an abnormality was detected during this examination in 37.5% of them. One prostate cancer was diagnosed. A gynecological consultation was performed in 27 women (62%) and a genital or breast complication was detected in 15% of them. A faecal occult blood test was performed in 13 patients, and positive for one. Regarding the antiretroviral treatment, a therapeutic modification was proposed in 20% of cases (complication, side effect, prevention of toxicity, etc).

**Conclusions: **Management with an annual summary report is recommended for the follow‐up of people living with HIV in France. Its realisation during a day‐care hospitalisation and the use of a standardised CRF allows to detect number of comorbidities. We showed that even in a medically followed population, vaccination coverage is insufficient, cardiovascular risk under‐evaluated and proctologic screening poorly performed.

## P236

### The coverage of influenza and pneumococcal vaccination among HIV‐infected patients in Denmark: a cross‐sectional survey

**L Larsen^1^, M Nguyen^2^ and I Johansen^1^**

^1^Department of Infectious Diseases, Odense University Hospital, University of Southern Denmark, Odense, Denmark. ^2^Department of Infectious Diseases, Odense University Hospital, Odense, Denmark

**Background: **Annual influenza vaccination is recommended for all HIV‐infected persons and so is pneumococcal vaccination at least once [1]. This is due to higher incidence rates for both infections among HIV‐infected persons [2,3] compared to HIV‐negative controls even in the later ART era. To clarify the use of and attitude towards these recommended vaccines among HIV‐infected persons in a high income country a questionnaire survey was performed.

**Materials and methods: **In a single HIV outpatient clinic at a tertiary hospital adult HIV‐infected persons were invited to participate in the survey during their regular visit in Spring 2017. The questionnaire consisted of four parts: (1) Demography, educational level and household annual income. (2) Influenza vaccination for season 2016/17 and previous/next year and reasons for not being vaccinated. (3) Pneumococcal vaccine uptake ever and reasons for not being vaccinated. (4) Source of information for the patient regarding vaccination. To identify predictors for the 2016/17 influenza vaccine we used Fisher's exact test and student *t*‐test. *p *<* *0.05 was considered significant.

**Results: **Preliminary data are presented here and the final results will be available for presentation. Pneumococcal vaccination had been offered to 13 (6%) of the 203 participants and nine (70%) had accepted. Of the 203 participants, 31% had received the influenza vaccine in the latest influenza season. Older age and any prior influenza vaccination were significantly associated with a positive influenza vaccination status (*p *<* *0.001). Not receiving information about vaccination was significantly associated with a negative influenza vaccination status (*p *<* *0.001). In the group not vaccinated in the latest influenza season, 25% were not aware that they have a medical condition with elevated risk of complications if they contracted influenza. Frequent reasons for refusing any of the vaccines were “perception of own excellent health and can´t see the need for or effect of vaccination” (39%) and lack of information (25%). Of all participants 29% stated that they had never received advice about vaccination from their doctor.

**Conclusions: **The vaccination coverage against influenza and Streptococcus pneumoniae is very low in this HIV‐infected population. The important reasons for this are lack of information and the patients´ conception of own excellent health. Increased awareness of vaccination among HIV patients is needed. The significant impacts on influenza vaccination behaviour were older age, prior vaccination.

**References**

[1] Ryom L, Boesecke C, Bracchi M, Ambrosioni J, Pozniak A, Arribas J, et al. Highlights of the 2017 European AIDS Clinical Society (EACS) Guidelines for the treatment of adult HIV‐positive persons version 9.0. HIV Med. 2018;19:309‐15. 

[2] van Aalst M, Lotsch F, Spijker R, van der Meer JTM, Langendam MW, Goorhuis A, et al. Incidence of invasive pneumococcal disease in immunocompromised patients: a systematic review and meta‐analysis. Travel Med Infect Dis. 2018;24:89‐100.

[3] Peters PJ, Skarbinski J, Louie JK, Jain S; New York City Department of Health Swine Flu Investigation Team, Roland M, et al. HIV‐infected hospitalized patients with 2009 pandemic influenza A (pH1N1)‐‐United States, spring and summer 2009. Clin Infect Dis. 2011;52 Suppl 1:S183‐8.

## P237

### The mental health of people living with HIV in the Asia‐Pacific: a literature review

**E O'Doherty^1^ and S Kerr^2^**

^1^Medical School, University of Aberdeen, Glasgow, UK. ^2^Biostatistics, HIV‐NAT, Bangkok, Thailand

**Background: **The mental health consequences of HIV and AIDS have been well researched across the globe [1]. Poorly managed mental health has been shown to negatively affect the disease progression of HIV and also worsen physical symptoms [2]. The Asia‐Pacific region has the second‐highest prevalence of HIV infection in the world and many of the nations are regarded as low‐to‐medium income (LTMIC) [3]. Due to cultural stigmatisation and discrimination, as well as poverty and low education, it is believed that common mental disorders are prevalent in large numbers across the region. However, due to poor mental health infrastructure, the most vulnerable key populations’ mental health requirements are not being met.

**Materials and methods:** ****Without the knowledge of how prevalent common mental disorders such as depression and anxiety are across the Asia‐Pacific region, this review was designed to study the current literature on the topic. Based in Bangkok and working under the guidance of HIV‐NAT, PsychINFO and MedLine databases were used along with a manual search. We reviewed 34 articles which studied the rates of common mental disorders within HIV populations.

**Results: **The review demonstrated that common mental disorders such as depressive and anxiety disorders are prevalent at high rates in HIV populations across the Asia‐Pacific region. Other correlates of mental health including suicidal ideation, alcohol and substance misuse were also found to be high. Key factors such as poor social support and stigmatisation were found to strongly impact upon the mental health of people living with HIV.

**Conclusions:** ****Whilst from this review it is clear that common mental disorders are prevalent in large number in PLWHIV, gaps remain between the acknowledgement of psychosocial factors as important to the daily lives of PLWHIV and the application of adequate resources to provide quality mental health support in LTMIC. Furthermore, studies included in this review described the detrimental effect mental health can have on physical disease progression. Recent literature has demonstrated that psychological intervention strategies can be extremely effective in improving mental health of those infected [4]. Mental health infrastructure must be improved in LTMIC to meet the requirements of people with HIV and reduce the symptoms of mental illness. Further research should recognise the urgency to implement an effective strategy to tackle these mental disorders by studying psychosocial interventions best suited to this region.

**References**

[1] Niu L, Luo D, Liu Y, Silenzio VM, Xiao S. The mental health of people lving with HIV in China, 1998–2014: a systematic review. PLoS One. 2016;11:e0153489.

[2] Churcher S. Stigma related to HIV and AIDS as a barrier to accessing health care in Thailand: a review of recent literature. WHO South East Asia J Public Health. 2013;2:12‐22.

[3] Avert. HIV and AIDS in Asia and the Pacific regional overview [Internet]. 2016 [cited 2018 Jan 26]. Available from: http://https://www.avert.org/professionals/hiv‐around‐ world/asia‐pacific/overview.

[4] Sherr L, Clucas C, Harding R, Sibley E, Catalan J. HIV and depression‐‐a systematic review of interventions. Psychol Health Med. 2011;16:493‐527.

## P238

### The risk of upper respiratory tract bacterial infections among HIV‐positive patients is higher for young MSM with detectable HIV RNA

**A Skrzat‐Klapaczyńska^1^, M Paciorek^1^, E Firląg‐Burkacka^2^, J Kowalska^1^ and A Horban^1^**

^1^Department of Adults’ Infectious Diseases, Medical University of Warsaw, Warsaw, Poland. ^2^HIV Out‐Patient Clinic, Hospital for Infectious Diseases, Warsaw, Poland

**Background: **The risk and characteristics of upper respiratory tract (URT) bacterial infections (URT‐BI) among HIV+ patients is understudied. Therefore, we analysed factors associated with its occurrence and the spectrum of pathogens among patients routinely followed at the HIV Out‐Patient Clinic in Warsaw.

**Methods: **All symptomatic HIV+ patients with available URT swab culture were included into analyses. Patients were followed from the day of registration in the clinic until first positive URT swab culture or last clinical visit. Cox proportional hazard models were used to identify factors associated with positive URT swab culture (those with *p *<* *0.1 in univariate included into multivariable).

**Results: **In total 474 patients were included into the analyses, 166 with positive URT swab. In general 416 (87.8%) man, 342 (72.1%) infected through MSM contact, 253 (53.4%) on antiretroviral therapy. Median follow‐up time was 3.4 (1.3 to 5.7) years, age 35.2 (30.6 to 42.6) years and CD4+ count 528 (400 to 685) cells/µL. The most common pathogens were *S. aureus* (40.4%) and *S. pyogenes* (13.9%). Patients with URT‐BI were more likely to be MSM (68.5% vs. 78.9%; *p *<* *0.016), have detectable viral load (20.9% vs. 12.0%; *p *<* *0.0001) and CD4+ cell count <500 cells/µL (55.2% vs. 39.0%; *p *=* *0.003). In multivariate survival analyses detectable viral load (HR 3.13, 95% Cl 2.34 to 4.19) and MSM (1.63, 1.09 to 2.42) were increasing, but older age (0.63, 0.58 to 0.69, per five years older) and higher CD4+ count (0.90, 0.85 to 0.95, per 100 cells/µL) decreasing the risk of URT‐BI (Table 1).

**Table:** Abstract P238 – Table 1. Univariate and multivariate logistic regression models for positive upper respiratory tract swab culture

	Univariate	Univariate	Multivariate^a^	Multivariate^a^
	HR (95% CI)	*p* value	HR (95% CI)	*p* value
Variable				
Age in years (per five years)	0.90 (0.88 to 0.92)	<0.0001	0.91 (0.89 to 0.93)	<0.0001
Last CD4+ cells/μL (per 100)	0.99 (0.98 to 0.99)	<0.0001	0.99 (0.99 to 1.00)	0.0005
Last CD8+ cells/μL (per 100)	1.00 (1.00 to 1.00)	<0.0001	1.00 (1.00 to 1.00)	0.12
HIV RNA >50 copies/mL	6.36 (4.64 to 8.73)	<0.0001	3.13 (2.34 to 4.19)	<0.0001
MSM vs. hetero	2.10 (1.28 to 3.45)	0.003	1.63 (1.09 to 2.42)	0.01
IDU vs. hetero	1.76 (0.85 to 3.66)	0.12	0.90 (0.57 to 1.43)	0.66
Unknown vs. hetero	3.23 (1.20 to 8.72)	0.02	2.27 (0.99 to 5.20)	0.005
Male gender	1.83 (1.03 to 3.22)	0.03	‐	‐
On antiretroviral treatment	0.90 (0.66 to 1.22)	0.50	‐	‐

^a^adjusted for all variables significant (*p *<* *0.1) in univariable analyses.

**Result: **URT BI are common among HIV+ patients with high CD4+ count. Similarly to general population most common pathogens are *S. aureus* and *S. pyogenes*. Risk factors identified in multivariate survival analysis indicate that younger MSM patients with detectable HIV viral load are at highest risk. Thus in clinical practice this group of patients requires special attention.

**Conclusions: **Our study suggests that in HIV/HCV co‐infected patients, HCV eradication after DAA‐based therapy is associated with an increase in the 10‐year Framingham cardiovascular risk.

## VIRAL HEPATITIS

## P239

### Early seroreversion after two doses of hepatitis A vaccination among HIV‐positive patients who had achieved serological response: incidence and associated factors

**S Huang^1^, C Huang^2^, T Chen^3^, C Yang^4^, S Lin^5^, Y Lee^6^, N Wang^7^, Y Lee^8^, K Lin^9^, C Liu^6^, P Lu^2^ and C Hung^10^**

^1^Department of Internal Medicine, National Taiwan University Hospital, Hsin‐Chu Branch, Hsin‐Chu, Taiwan. ^2^Department of Internal Medicine, Kaohsiung Medical University Hospital, Kaohsiung, Taiwan. ^3^Department of Internal Medicine, Kaohsiung Municipal Ta‐Tung Hospital, Kaohsiung, Taiwan. ^4^Department of Internal Medicine, Far Eastern Memorial Hospital, New Taipei City, Taiwan. ^5^Department of Internal Medicine, Taichung Veterans General Hospital, Taichung, Taiwan. ^6^Department of Internal Medicine, Changhua Christian Hospital, Changhua, Taiwan. ^7^Department of Internal Medicine, Tri‐Service General Hospital, Taipei, Taiwan. ^8^Department of Internal Medicine, Chung Shan Medical University Hospital, Taichung, Taiwan. ^9^Department of Medicine, National Taiwan University Hospital, Jin‐Shan Branch, New Taipei City, Taiwan. ^10^Department of Internal Medicine, National Taiwan University Hospital, Taipei, Taiwan

**Background: **Recent outbreaks of hepatitis A virus (HAV) infection among MSM, injecting drug users and homeless people in European countries, US and Taiwan have raised public concerns and calls for vaccination campaign among at‐risk population. Serological responses (seroresponse) and durability of HAV vaccination among HIV‐positive patients were impaired compared to those among HIV‐negative individuals. The incidence of and associated factors with early seroreversion among HIV‐positive patients who had achieved seroresponses after two doses of HAV vaccination remains unclear during an outbreak setting.

**Materials and methods: **This multicentre study was conducted in three medical centres in Taiwan, where an unprecedented HAV outbreak took place between 2015 and 2017. HIV‐positive adults achieving seroresponses after two doses of HAV vaccination administered 6 months apart were prospectively followed for anti‐HAV IgG. A 1:4 case‐control study was conducted to identify factors associated with seroreversion. Case patients were those testing negative for anti‐HAV IgG 12 months after HAV vaccine‐induced seroconversion (seroreverters) and controls were those with similar follow‐up durations who were able to maintain antibody responses.

**Results: **Between 2015 and 2017, 621 HIV‐positive patients were included who had documented seroconversion within 12 months after HAV vaccination. At the time of HAV vaccination, 96.6% of the included patients were receiving antiretroviral therapy, about 90% had plasma HIV RNA load <50 copies/mL and CD4 lymphocyte count >350 cells/μL. After a median follow‐up of 623 days (range 523 to 705), 23 (3.7%) seroreverted. In the case‐control study, peak anti‐HAV IgG titer and positive anti‐HAV response at six months were inversely associated with early seroreversion with adjusted odds ratio (aOR) of 0.13 (95% CI 0.01 to 0.41) and 0.02 (95% CI 0.001 to 0.19), respectively, while seroreversion was more likely to occur in patients with older age (aOR 1.50, 95% CI 1.13 to 2.64) or with HIV viraemia at 12 months (aOR 13.66, 95% CI 1.40 to 315.28) in multivariable analyses.

**Conclusions: **During an outbreak setting, early seroconversion occurred in 3.7% of HIV‐positive patients after successful HAV vaccination after 20 months of follow‐up. Lower and delayed seroresponses to HAV vaccination, older age and failure to maintain HIV viral suppression at 12 months were associated with early seroreversion.

## P240

### Current chronic hepatitis C treatment in HIV co‐infection in Portugal: a cohort of 2133 patients presented by GEPCOI (Portuguese Coinfection Study Group)

**A Miranda^1^, J Mendez^2^, R Serrão^3^, F Vale^4^, M Manata^5^, S Pinto^6^, A Gomes^7^, M Prata^8^, P Pacheco^9^, R Pazos^10^, R Pereira^11^, A Martins^12^, I Germano^13^, S Rocha^14^, A Reis^15^ and R Sarmento e Castro^2^**

^1^Serviço de Infecciologia e Medicina Tropical, Hospital de Egas Moniz, Centro Hospitalar de Lisboa Ocidental, Lisbon, Portugal. ^2^Serviço de Doenças Infecciosas, Centro Hospitalar do Porto, Porto, Portugal. ^3^Serviço de Doenças Infecciosas, Centro Hospitalar de São João, Porto, Portugal. ^4^Serviço de Doenças Infecciosas, Centro Hospitalar de Setúbal, Setúbal, Portugal. ^5^Serviço de Doenças Infecciosas, Hospital de Curry Cabral, Centro Hospitalar de Lisboa Central, Lisbon, Portugal. ^6^Serviço de Doenças Infecciosas, Centro Hospitalar de Gaia/Espinho, Gaia, Portugal. ^7^Serviço de Doenças Infecciosas, Hospital Garcia de Orta, Almada, Portugal. ^8^Serviço de Doenças Infecciosas, Centro Hospitalar e Universitário de Coimbra, Coimbra, Portugal. ^9^Serviço de Doenças Infecciosas, Hospital Fernando da Fonseca, Amadora, Portugal. ^10^Serviço de Medicina, Centro Hospitalar Universitário do Algarve, Hospital de Portimão, Portimão, Portugal. ^11^Serviço de Doenças Infecciosas, Centro Hospitalar Universitário do Algarve, Hospital de Faro, Faro, Portugal. ^12^Serviço de Doenças Infecciosas, Centro Hospitalar do Baixo Vouga, Aveiro, Portugal. ^13^Serviço de Medicina, Hospital de São José, Centro Hospitalar de Lisboa Central, Lisbon, Portugal. ^14^Serviço de Doenças Infecciosas, Unidade Local Saúde Alto Minho, Viana do Castelo, Portugal. ^15^Serviço de Doenças Infecciosas, Hospital dos Marmeleiros, Funchal, Portugal

**Background: **Direct acting antiviral drugs (DAA) changed the paradigm of hepatitis C therapy, significantly improving treatment response rates, patient life expectancy and quality of life. In Portugal, DAA therapy has been sequentially reimbursed since 2015 and generalised use of interferon‐free regimens became current practice. HCV/HIV co‐infection is a priority to engage HCV treatment due to faster disease progression. Real‐world data regarding efficacy and safety of DAA treatment in HIV/HCV co‐infected patients remain scarce.

**Material and methods**

Multicentre, retrospective, observational study of a real‐life clinical cohort (15 Portuguese hospital centres) of HCV/HIV infected patients, HCV treated with DAA‐based regimens, who completed treatment and response evaluation (12/24 weeks after treatment). Demographic, epidemiological, clinical, virological and treatment response data were analysed. Hepatic fibrosis was assessed by non‐invasive methods. Statistical analysis was performed by Microsoft Excel and SPSS Statistics v.24.0.

**Results: **A total of 2133 patients were included. Demographic characterisation revealed male predominance (83%), mean age of 46 years and 96% Portuguese origin. HCV was acquired by intravenous drug use in 91% and 69% of patients were naïve. Genotype prevalence was 67.8% G1, 1.2% G2, 15.3% G3 and 15.7% G4. Hepatic fibrosis evidenced METAVIR score ≤F2 in 59.2%, 18.7% F3 and 22.1% F4. Mean baseline TCD4 was 619 cells/mm^3^ and 96% of patients on antiretroviral therapy were virologically suppressed. Most prescribed DAA regimens were SOF/LDV (84%) and SOF+RBV (9.6%). Global sustained virological response (SVR) achieved was 95%. SVR by genotype was 96% G1, 76% G2, 91% G3 and 96% G4. SVR by fibrosis stage was 97% F1, 95% F2, 94% F3 and 92% F4. SVR by DAA regimen was SOF/LDV 96% (n = 1784), SOF+SMV 100% (n = 2), SOF+RBV 87% (n = 204), SOF+DCV 91% (n = 54), GZR/EBR 93% (n = 15), 3D 95% (n = 43), SOF/VEL 86% (n = 7) and SOF+PR 100% (n = 24). Linear regression analysis showed lower response rates in the treatment of genotypes 2/3 (*p* < 0.05), those treated with SOF/RBV (*p* < 0.05) and with cirrhosis (*p* < 0.05). Logistic regression confirmed cirrhosis as a negative predictor of response. During the study period, 0.9% patients deceased and 1.6% were lost to follow‐up.

**Conclusion: **This real‐life Portuguese experience shows a high SVR and retention in care in a large HCV/HIV co‐infected cohort. This study corroborates early treatment in HIV‐HCV as response rates decrease by fibrosis stage. The current health policy will promote a wider and individualised treatment, aiming the near elimination of HCV in this high risk population.

## P241

### Hepatitis C virus re‐infection after viral clearance of HCV among HIV‐positive patients with recent HCV infection in Taiwan

**M Huang^1^, W Liu^1^, L Su^1^, C Wu^1^, P Wu^2^, S Yang^2^, J Zhang^2^, H Chang^2^, S Chang^3^, C Liu^1^, H Sun^1^, C Hung^1^ and S Chang^1^**

^1^Internal Medicine, National Taiwan University Hospital, Taipei, Taiwan. ^2^Center of Infection Control, National Taiwan University Hospital, Taipei, Taiwan. ^3^Laboratory Medicine, National Taiwan University Hospital, Taipei, Taiwan

**Background: **A high rate of HCV re‐infection after viral clearance among HIV‐positive MSM has been well described in Europe. However, whether the high rate of HCV re‐infection also occurs in the Asia‐Pacific region remains unknown. Given the observation that patients with recent HCV infection had higher incidence of HCV re‐infection than those with chronic infection and the concerns about onward HCV transmission if left undiagnosed, this study aimed to assess the incidence rate of HCV re‐infection after HCV viral clearance, to identify the factors associated with HCV re‐infection and to examine different testing strategies for timely diagnosis of HCV re‐infection among HIV‐positive Taiwanese patients with recent HCV infection.

**Materials and methods: **Among 3083 HIV‐positive patients with negative baseline anti‐HCV antibody seeking medical care at the National Taiwan University Hospital between January 2011 and December 2015, data of those with recent HCV infection were retrospectively collected and analysed, and patients were followed until death, loss to follow‐up or 31 December 2017. HCV re‐infection was defined as recurrence of HCV viraemia after having achieved sustained virological response (SVR) with anti‐HCV treatment or after spontaneous clearance. 

**Results: **In the 5‐year study period, 130 (114 MSM) out of 3083 HIV‐positive patients who receive a diagnosis of recent HCV infection with an incidence rate of 13.73 per 1000 person‐years of follow‐up (PYFU) were included. Among the 65 patients who received anti‐HCV treatment, 58 (89.2%) achieved SVR. Of the remaining 65 patients without anti‐HCV treatment, chronic infection developed in 54 (83.1%) and spontaneous clearance in 11 (16.9%). Among the 69 patients who cleared their primary HCV infection, 12 acquired HCV re‐infection, resulting in an overall incidence rate of 7.75 per 100 PYFU. Compared with patients without HCV re‐infection, those with re‐infection were more likely to have syphilis (91.7% vs. 52.6%, *p* = 0.02) and to acquire syphilis more often (median episode, 2 vs. 1, *p* = 0.003). Nevertheless, follow‐up HCV RNA measurements detected all cases of HCV re‐infection, HCV testing performed only following a serological diagnosis of syphilis and elevated aminotransferases only detected 42.9% and 71.4% of the cases, respectively.

**Conclusions: **Similar to the findings in Europe, we observed a high incidence rate of HCV re‐infection among HIV‐positive Taiwanese patients with recent HCV infection, which was significantly associated with syphilis. To identify recent HCV re‐infection, annual HCV RNA testing should be instituted instead of testing driven by symptoms, syphilis or elevated aminotransferases.

## P242

### How to identify HIV‐positive men who have sex with men at risk for HCV re‐infection: is a screening question about condom use sensitive enough?

**P Künzler‐Heule^1^, S Engberg^2^, M Battegay^3^, K Fierz^4^, A Schmidt^5^, B Hampel^6^, M Stöckle^3^, C Béguelin^7^, D Braun^6^, J Fehr^6^ and D Nicca^1^**

^1^Institute of Nursing Science, University of Basel, Basel, Switzerland. ^2^School of Nursing, University of Pittsburgh, Pittsburgh, PA, USA. ^3^Department of Infectious Disease, University Hospital Basel, Basel, Switzerland. ^4^Nursing, Zurich University of Applied Sciences, Winterthur, Switzerland. ^5^Division of Infectious Diseases, Cantonal Hospital St. Gallen, St. Gallen, Switzerland. ^6^Division of Infectious Diseases, University Hospital Zurich, Zurich, Switzerland. ^7^Department of Infectious Diseases, University Hospital and University of Bern, Bern, Switzerland

**Background: **HIV‐positive men who have sex with men are at risk of HCV re‐infection after successful treatment and are therefore a major obstacle in the elimination of HCV. In the Swiss HCVree trial, HIV/HCV co‐infected MSM received HCV treatment and those reporting inconsistent condom use for anal intercourse with non‐steady partners were additionally invited to participate in a behavioural intervention tailored to prevent re‐infection. The accuracy of using this criterion to identify men at risk for HCV re‐infection remains unclear as other sexual and drug‐using behaviours are described as risk factors for HCV acquisition (e.g. sharing of injection equipment, sexualised drug use). The aims of this study were to (1) describe other HCV‐related risk behaviours in HIV/HCV co‐infected MSM and (2) examine the sensitivity of the condom use question in identifying men who engaged in these risk behaviours.

**Materials and methods: **Participants were divided into two groups, those who reported not having anal intercourse with non‐steady partners or consistently using condoms versus those with anal intercourse reporting inconsistent condom use. Self‐report data about engaging in other potential HCV transmission risk behaviours during the prior six months were collected at baseline: fisting with/without gloves, unsafe sharing of sex toys with non‐steady partners, use of specific substances (name, yes/no). If the answer to substance use was yes, the participant was asked about sexualised drug use (yes/no) and the method of use.

**Results: **Of 122 Swiss HCVree participants, 118 (97%) disclosed their sexual and drug‐use behaviours. Inconsistent condom use was reported by 72 (61%). This group was also significantly more likely to be engaged in other risky sexual behaviour (Table 1). The sensitivity of the inconsistent condom use question regarding detecting other at‐risk behaviours was: (1) 100% for not using gloves when fisting, (2) 88.2% for injecting drugs, (3) 84.6% for any substance use, (4) 84.1% for sexualised drug use and (5) 66.7% for sharing sex toys.

**Table:** Abstract P242 – Table 1. Odds of reporting other risk behaviours if reported inconsistent condom use with non‐steady partners

Characteristic	Consistent condom use group^a^ (N = 46) n (%)	Inconsistent condom use group (N = 72) n (%)	Odds ratio (95% CI)	*p* value
Substance use yes	8 (17)	44 (61)	7.46 (3.03 to 18.32)	<0.001^b^
Sexualised substance use yes	7 (15)	37 (51)	5.89 (2.33 to 14.90)	<0.001^b^
Injecting drugs	2 (4)	15 (21)	5.79 (1.26 to 22.66)	0.01^c^
Not using gloves for fisting	0	14 (19)	11.95 (1.52 to 93.77)	0.005^c^
Sharing sex toys	7 (15)	18 (25)	1.86 (0.71 to 4.88)	0.25^c^

^a^including 27 men non‐engaging in anal intercourse with non‐steady partner. ^b^chi‐square test. ^c^Fisher's exact test.

**Conclusions: **Our findings show that HIV/HCV co‐infected MSM engage in multiple sexual and drug‐use behaviours commonly associated with HCV transmission. While using a question about inconsistent condom use showed good sensitivity in relation to identify individuals engaging in other HCV transmission behaviours, we missed men engaging in behaviours that probably carry even larger attributable risk (e.g. injecting drugs, engaging in sexualised drug use activities). Therefore, future behavioural intervention programmes should consider additional inclusion criteria or including all HIV/HCV co‐infected MSM.

## P243

### Comparative prevalence of hepatic steatosis and other metabolic conditions in treated hepatitis C cohorts, with or without HIV co‐infection, in a London hospital

**M Lee^1^, J Mok^1^, J Susanne^2^, L Church^1^, C Mohanadass^1^, S Vaidya^1^, T Wong^2^, R Kulasegaram^1^, B Peters^1^ and A Duncan^1^**

^1^Harrison Wing Department, Guy's and St Thomas Hospital NHS Foundation Trust, London, UK. ^2^Gastroenterology Department, Guy's and St Thomas Hospital NHS Foundation Trust, London, UK

**Background: **HIV and HCV infections are associated with the metabolic syndrome, even after HCV clearance [1,2]. We aim to describe the differences in the metabolic phenotype of people with treated hepatitis C infections, with and without HIV co‐infection, compared to a HCV‐negative cohort of people living with HIV.

**Materials and methods: **Three cohorts were identified attending a tertiary London hospital: (1) A representative HIV‐positive cohort without HCV infection, matched for age, gender and ethnicity to the patient population from September 2014. (2) All patients with HIV and hepatitis C co‐infection referred for direct‐acting antivirals (DAA) between October 2015 and June 2017. (3) All patients with hepatitis C referred for DAA between September 2015 and June 2017. Routine clinic data were collected retrospectively until 31 May 2018, including demographics, medical and treatment history, biomarkers of disease, biochemistry results and lifestyle factors. Univariate analysis was performed using chi‐squared or Fisher's exact test when appropriate for categorical data, and ANOVA or Kruskal‐Wallis test for continuous data. Multivariate analysis was performed using binary logistic regression.

**Results: **Cohorts 1, 2 and 3 included 279, 78 and 176 patients respectively. Table 1 summarises univariate analysis results. Cohort 2 were younger and more predominantly male compared to other cohorts; cohorts 2 and 3 were predominantly white compared to cohort 1. Both HIV cohorts had similar mean CD4 and undetectable HIV viral loads regardless of HCV status. Both HCV cohorts had similar rates of HCV sustained viral response at 48 weeks; however, median pre‐treatment Fibroscan scores were higher in cohort 3 than 2 (7.05 vs. 5.65 kPa, *p* = 0.002). We were unable to analyse duration of HCV infection due to missing dates of diagnosis. Prevalence of cardiovascular or cerebrovascular events (CCVE), hypertension, type 1 or 2 diabetes, current smoking, but not hepatic steatosis or chronic renal impairment, differed significantly between cohorts. Adjusting for age, gender, ethnicity, smoking and diabetes, cohort 3 (OR 1.88, 95% CI 1.00 to 3.55) and cohort 2 (OR 2.62, 95% CI 1.28 to 5.36) were more likely to have hepatic steatosis than cohort 1 (*p* = 0.016). Differences in CCVE, diabetes or hypertension prevalence were not significant following logistic regression.

**Table:** Abstract P243 – Table 1. Summary of univariate analysis

	Cohort 1: HIV		Cohort 2: HIV/HCV		Cohort 3: HCV	
	n = 279	% / SD	n = 78	% / SD	n = 176	% / SD	*p* value
Mean age	52.7	11.2 (SD)	45.6	9.6 (SD)	54.6	12.8 (SD)	<0.001
Male gender	200	71.7	73	93.6	109	61.9	<0.001
Ethnicity	<0.001
Black	116	41.6	7	9.2	27	15.5
White	132	47.3	64	84.2	130	74.7
Other	31	11.1	5	6.6	17	9.8
Not documented	0	‐	2	‐	2	‐
Mean current CD4 count (cells/uL)	649	278 (SD)	694	248 (SD)	‐	‐	0.197
Patients with undetectable HIV viral loads (<50 copies/mL)	255	91.4	76	97.4	‐	‐	0.070
Patients with HCV sustained viral response at Week 48	‐	‐	78	100	171	97.2	0.134
Cardiovascular or cerebrovascular events	28	10.0	1	1.3	6	3.4	0.003
Hypertension	87	31.2	8	10.4	40	22.9	0.001
Type 1 or 2 diabetes	48	17.2	3	3.8	19	10.9	0.005
Current smoking status	40	15.9	24	34.3	48	45.3	<0.001
Evidence of hepatic steatosis	44	15.8	18	23.1	32	18.2	0.318
Chronic kidney disease stage 3 or worse	28	10.0	9	11.5	9	5.1	0.117

**Conclusions: **Patients with treated HCV with or without HIV co‐infection differed from those with HIV mono‐infection in their demographics and metabolic phenotype. Continued monitoring of hepatic steatosis following treatment for HCV infection is necessary for hepatic complications, and the presence of HIV co‐infection increases this risk.

**References**

[1] Adinolfi LE, Nevola R, Guerrera B, D'Alterio G, Marrone A, Giordano M, et al. Hepatitis C virus clearance by direct‐acting antiviral treatments and impact on insulin resistance in chronic hepatitis C patients. J Gastroenterol Hepatol. 2018;33:1379‐82.

[2] Chang ML. Metabolic alterations and hepatitis C: from bench to bedside. World J Gastroenterol. 2016;22:1461‐76.

## P244

### HIV/hepatitis C co‐infection in key populations from Romania: HepCare Europe ‐ a model of management and service delivery

**C Oprea^1^, I Ianache^2^, A Kosa^2^, I Popa^2^, S Florescu^1^, S Lazar^1^ and P Calistru^1^**

^1^Carol Davila University of General Medicine and Pharmacy, Infectious Diseases, Victor Babes Hospital, Bucharest, Romania. ^2^Infectious Diseases, Victor Babes Hospital, Bucharest, Romania

**Background: **In the last decade, the prevalence of HCV increased dramatically among Romanian injecting drug users (IDUs). In this context, Bucharest was included in HepCare Europe project, co‐funded by the European Commission, whose aim is to improve access to HCV testing, diagnosis and treatment among key risk populations, through outreach to the community and integration of primary and secondary care services.

**Methods: **Prospective study on HIV‐infected patients who tested positive for HCV antibodies during the enrolment in HepCare project at Victor Babes clinical site, Bucharest, between April 2016 and April 2018. Five work packages were developed to enrich management of HCV in hard‐to‐reach populations: HepCheck (HCV screening with rapid oral tests ‐ Oraquick), HepLink (linkage to care), HepEd (inter‐professional education), HepFriend (peer support programmes) and HepCost (estimation of the cost‐efficiency of the model and evaluation of the socio‐economic impact).

**Results: **Out of 510 screened subjects, 213 (41.7%) had positive HCV antibody test, with the majority 92.4% (197/213) being IDUs. The percent of HCV positivity was higher in subjects from drug support and opioid substitution centres 95.0% (58/61), compared to patients from prisons 37.2% (57/153), night shelters 9.8% (19/193) and other health care facilities 76.6% (79/103), *p* < 0.0001. A total of 120 subjects (56.3%) were already linked to care, out of which 30 (30.0%) were diagnosed with high fibrosis score (METAVIR >9 kPa) by Fibroscan. Fifty‐eight (48.3%) IDUs were co‐infected with HIV (Table 1). Treatment with DAAs was initiated only in five co‐infected patients, four achieved sustained virological response, while one patient with genotype 3 was a non‐responder. Two educational HCV Masterclasses, organised within the HepEd work package, were attended by 150 health care practitioners (HCPs) and printed educational materials for patients, peers and social workers were distributed. Three peers were trained for awareness and linkage to testing and patients from key populations were addressed in nine peer support sessions (HepFriend).

**Table:** Abstract P244 – Table 1. Characteristics in HCV/HIV co‐infected patients from key populations with rapid HCV positive antibody test at screening

Characteristics	HIV‐positive patients (n = 58)
CD4 cell count/µL	median (IQR)	510 (327 to 659)
HIV viral load (log10 copies/mL)	median (IQR)	2.68 (1.27 to 4.57)
cART^a^	n (%)	22 (37.9)
HIV‐RNA undetectable	n (%)	15/22^b^ (68.1)
METAVIR F score ≥9 kPa (Fibroscan evaluation)	n (%)	13 (22.4)
HCV viral load (log10 IU/mL)	median (IQR)	6.27 (5.66 to 6.59)
HCV‐RNA undetectable	n (%)	6 (10.3)
ALT level (IU/L)	median (IQR)	66 (44 to 111)
HBsAg positive	n (%)	7 (12.0)

^a^combined antiretroviral treatment. ^b^22 IDUs on cART.

**Conclusions: **HIV/HCV co‐infection among subjects from key populations was high, especially among IDUs from drug support centres. Socio‐economic and structural barriers limited the treatment with DAAs in this group. There is an urgent need to improve the HIV/HCV screening and linkage to care for patients from hard‐to‐reach populations, to actively involve HCPs in HIV/HCV management and to find the best modalities to overcome the barriers to treatment.

## P245

### Increased total and LDL cholesterol plasma levels upon direct antiviral agents (DAAs) driven HCV eradication

**G Taliani^1^, G Marchetti^2^, A Cingolani^3^, M Lichtner^4^, S Cicalini^5^, E Quiros Roldan^6^, M Ursitti^7^, E Girardi^8^, A Antinori^5^, M Puoti^9^, A d'Arminio Monforte^2^ and A Cozzi‐Lepri^10^, on behalf of the Icona and Hepalcona Foundation Study Group**

^1^Department of Clinical Medicine, Sapienza University of Rome, Rome, Italy. ^2^Clinic of Infectious and Tropical Diseases, ASST Santi Paolo e Carlo, University of Milan, Milan, Italy. ^3^Institute of Clinical Infectious Diseases, Policlinic A. Gemelli, Catholic University of the Sacred Heart, Rome, Italy. ^4^Unit of Infectious Diseases, SM Goretti Hospital, Sapienza University of Rome, Latina, Italy. ^5^HIV/AIDS Unit, INMI L. Spallanzani IRCCS, Rome, Italy. ^6^Department of Clinical and Experimental Sciences, ASST Spedali Civili, University of Brescia, Brescia, Italy. ^7^Department of Infectious Diseases, S. Maria Nuova IRCCS Hospital, Reggio‐Emilia, Italy. ^8^Department of Epidemiology, INMI L. Spallanzani IRCCS, Rome, Italy. ^9^Division of Infectious Diseases, ASST Grande Ospedale Metropolitano Niguarda, Milan, Italy. ^10^Institute for Global Health, University College London, London, UK.

**Background: **HCV has complex interactions with human lipid metabolism leading to downregulation of cholesterol levels. Treatment with DAAs was proven to induce a sharp and significant increase in total and low‐density lipoprotein cholesterol (LDL) persisting after the end of treatment (EOT). It has been suggested that the increased risk of cardiovascular disease (CVD) associated with ritonavir‐boosted darunavir (DRV/r) use may be independent from dyslipidaemia [1]. The aim of this analysis was to examine cholesterol changes in HIV‐HCV co‐infected patients after HCV clearance and according to DRV/r, ATV/r or RAL exposure during DAA.

**Materials and methods: **The analysis includes data of HIV/HCV co‐infected patients in the Icona and HepaIcona cohorts for whom pairs of biomarkers were available. The first pair includes the two most recent values in a window [‐12;0] months of the date of DAA initiation. The second pair uses the latest in the window [+4;+12] months of the date EOT. Mean values at each time point were calculated as well as the difference among pairs. Univariable paired t‐test were conducted to test whether the variations were significantly different from zero. An ANCOVA analysis was used to test whether there was an effect of DRV/r, ATV/r and RAL use.

**Results: **We included 465 patients on ART, who achieved SVR; 22% on DRV/r, 20% on ATV/r and 24% on RAL. Patients’ characteristics: median age 52 (50 to 55) years; 26% female; median BMI 24 (21 to 26) kg/m^2^; median CD4 584 (360 to 828) cells/mm^3^; HCV genotype 1a (35%), 3a (18%), 4 (13%). Total and LDL‐cholesterol along with platelet count, which prior to DAA tended to be stable or decrease, significantly increased after HCV clearance whereas high‐density lipoprotein (HDL) cholesterol remained unchanged. These changes, which occur in a short time‐lapse, potentially contribute to an increase in CVD risk through shared or separate pathways (Figure 1). Moreover, in patients exposed to DRV/r a significant increase of total cholesterol was observed over T1–T2 compared to unexposed ones (Δ+10.7 mg/dL; *p* = 0.01); no evidence for a difference was found for ATV/r (Δ+2.1 mg/dL; *p* = 0.69) or RAL (Δ‐6.9 mg/dL; *p* = 0.08) treated patients.

**Abstract P245 – Figure 1**. DAG for the model exploring the causal link between HCV‐RNA eradication and risk of CVD.

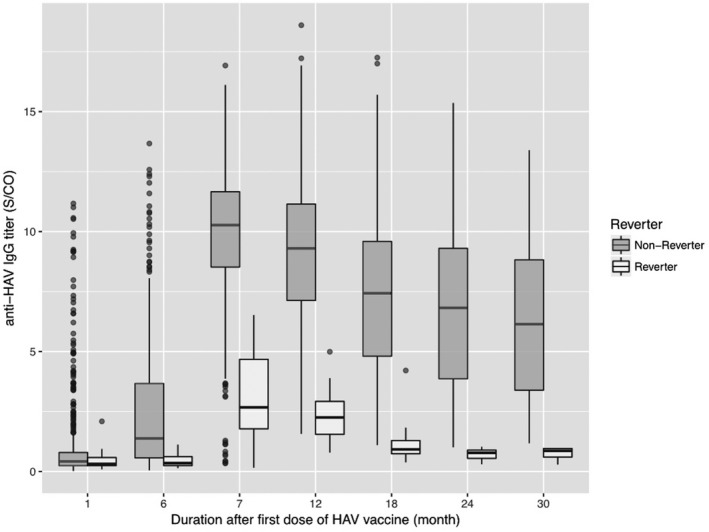

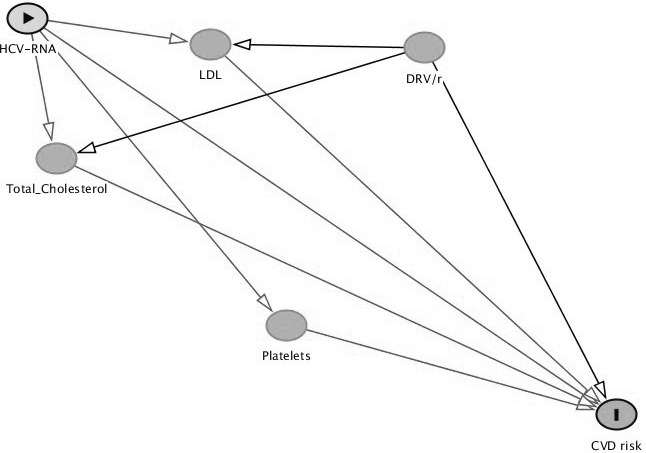

**Conclusions: **A complex and rapid change of risk factors for CVD risk seems to occur in HIV‐HCV co‐infected patients after HCV eradication with DAA, including increase of total and LDL‐cholesterol and platelets. Exposure to DRV/r independently contributed to the increase of total cholesterol. It is increasingly important to fit the individuals’ risk profiles before HCV treatment.

**Reference: **[1] Ryom L, Lundgren JD, El‐Sadr W, Reiss P, Kirk O, Law M, et al. Cardiovascular disease and use of contemporary protease inhibitors: the D:A:D international prospective multicohort study. Lancet HIV. 2018;5:e291‐300.

## P246

### Molecular determining of HIV‐1 with the presence of hepatitis B virus and hepatitis C virus co‐infections

**M Sayan^1^, M Ozguler^2^, F Sarigul Yildirim^3^, T Yidirmak^4^, A Gündüz^5^, B Dokuzoğuz^6^, M Çelen^7^, D İnan^8^, Y Heper^9^, G Munis Ersoz^10^, I Karaoglan^11^, N Ceran^12^, A Deveci^13^, S Ozturk^14^, S Sayin Kutlu^15^, H Ozkan Ozdemir^16^, A Akbulut^17^, S Yazici^18^, A Sener^19^, A Cagatay^20^ and S Unal^21^**

^1^Clinical Laboratory and DESAM, Kocaeli University (Turkey) and Near East University (Northern Cyprus), Kocaeli and Nicosia, Turkey. ^2^Infectious Diseases, Elazıg Education and Research Hospital, Elazıg, Turkey. ^3^Infectious Diseases, Antalya Education and Research Hospital, Antalya, Turkey. ^4^Infectious Diseases, Okmeydani Education and Research Hospital, Istanbul, Turkey. ^5^Infectious Diseases, Sisli Etfal Education and Research Hospital, Istanbul, Turkey. ^6^Infectious Diseases, Ankara Numune Education and Research Hospital, Ankara, Turkey. ^7^Infectious Diseases, Dicle University, Faculty of Medicine, Diyarbakir, Turkey. ^8^Faculty of Medicine, Infectious Diseases, Akdeniz University, Antalya, Turkey. ^9^Infectious Diseases, Uludag University, Faculty of Medicine, Bursa, Turkey. ^10^Infectious Diseases, Mersin University, Faculty of Medicine, Mersin, Turkey. ^11^Infectious Diseases, Gaziantep University, Faculty of Medicine, Gaziantep, Turkey. ^12^Infectious Diseases, Haydarpasa Numune Education and Research Hospital, Istanbul, Turkey. ^13^Infectious Diseases, Ondokuz Mayıs University, Faculty of Medicine, Samsun, Turkey. ^14^Infectious Diseases, Fatih Sultan Mehmet Education and Research Hospital, Istanbul, Turkey. ^15^Infectious Diseases, Pamukkale University, Faculty of Medicine, Denizli, Turkey. ^16^Infectious Diseases, Bozyaka Education and Research Hospital, Izmir, Turkey. ^17^Infectious Diseases, Firat University, Faculty of Medicine, Elazig, Turkey. ^18^Infectious Diseases, Medeniyet University, Goztepe Education and Research Hospital, Istanbul, Turkey. ^19^Infectious Diseases, Onsekiz Mart University, Faculty of Medicine, Canakkale, Turkey. ^20^Infectious Diseases, Istanbul University, Faculty of Medicine, Istanbul, Turkey. ^21^Infectious Diseases, Hacettepe University, Faculty of Medicine, Ankara, Turkey

**Background: **Because of the similar modes of transmission (include unsterile medical injection, blood transfusion, sexual intercourse and injecting drug use), co‐infection with viral hepatitis and HIV is increasingly seen as a major public health problem. In addition to that, co‐infection with HIV and HBV or HCV infection increases the urgency of starting ART [1]. On the other hand, increasing the demographic mass movements, may be changed the virus transmitted trends and may have a potential effect for HIV and co‐infection surveillance in the future. In this study, we aimed to determine the molecular characteristics of HIV‐1 in the presence of HBV and HCV co‐infections in Turkey.

**Material and methods**

The present study was conducted between March 2010 and March 2017. HIV‐1 RNA was detected and quantified by a commercial real‐time PCR assays. Subtyping and genotypic resistance analysis were performed by population sequencing of the viral protease and reverse transcriptase regions of HIV‐1 pol gene. Drug resistance mutations were defined according to the Surveillance Drug Resistance Mutation list as recommended by the World Health Organization [2].

**Results: **We detected totally 3896 HIV‐1 positive patients whose molecular laboratory tests were completed in Turkey. The viral hepatitis co‐infections were detected in 4.3% (n = 170) of all HIV‐1 infected patients in this study. HBV and HCV co‐infections were observed as 3.2% and 0.5% in HIV‐1 positive patients, respectively. Major HIV‐1 subtypes were detected as a group M, subtype B (67.5%). We observed 13.5% drug resistance mutation motifs in HIV‐1 genomes which included this study. NRTI, NNRT and PI resistance mutations have been investigated in a detailed manner and the mutation rates were determined 9.4%, 5.3% and 1.8%, respectively.

**Conclusions: **HBV and HCV co‐infections can be seen more frequently in HIV‐positive patients because of similar transmission routes. However, the ART drug resistance mutation pattern are observed similar. The molecular characterisation of HIV‐1 genome for ART resistance is not different from non‐co‐infected. In the light of increasing demographic mass movements and their potential to affect infection transmission trends, patients with HIV‐1 and their viral hepatitis co‐infections should be recommended an carefully surveillance.

**References**

[1] A Working Group of the Office of AIDS Research Advisory Council (OARAC) [Internet]. Guidelines for the use of antiretroviral agents in adults and adolescents living with HIV [cited 2018 Jan 1]. Available from: http://https://aidsinfo.nih.gov/guidelines.

[2] Bennett DE, Camacho RJ, Otelea D, Kuritzkes DR, Fleury H, Kiuchi M, et al. Drug resistance mutations for surveillance of transmitted HIV‐1 drug‐resistance: 2009 update. PLoS One. 2009;4:e4724.

## P247

### Estimation of HCV infection prevalence in a Basic Health Area of Madrid (Spain)

**J Martínez‐Sanz^1^, P Pérez Elías^2^, M Herrero Delgado^3^, R Barea^4^, Y de la Fuente Cortés^5^, M Vivancos Gallego^1^, A Moreno Zamora^1^, S Ares Blanco^3^, L Polo Benito^6^, A Mesa^7^, C Labrador Manzanares^8^, P González Huerga^9^, C Chamorro Escobar^5^, A Cano Espín^2^, A Fernández Rivera^2^, C Santos Álvarez^2^, M Rodríguez^10^, B Romero^10^ and M Pérez‐Elías^1^**

^1^Infectious Diseases, University Hospital Ramón y Cajal, Madrid, Spain. ^2^Primary Care, C.S. García Noblejas, Madrid, Spain. ^3^Primary Care, C.S. Mar Báltico, Madrid, Spain. ^4^Primary Care, C.S. Panamá, Madrid, Spain. ^5^Primary Care, C.S. Aquitania, Madrid, Spain. ^6^Hospital General Universitario Gregorio Marañón, Madrid, Spain. ^7^Primary Care, C.S. Avenida de Aragón, Madrid, Spain. ^8^DUE, Hospital de la Princesa, Madrid, Spain. ^9^SUMMA, Madrid, Spain. ^10^Microbiology, University Hospital Ramón y Cajal, Madrid, Spain

**Background: **In Spain, epidemiological data on HCV infection are scarce. Our objective is to estimate the prevalence of HCV infection in the Basic Health Area of the Ramón y Cajal Hospital (BHA‐RYC), and distinguish between new HCV diagnoses (NHCVD) and previously diagnosed infections, linked or not.

**Methods: **Sub‐analysis of the DRIVE03 study, which was carried out in four health centres of the BHA‐RYC, where patients without HCV aged between 18 and 70 years were prospectively included. After completing an HCV risk exposure and indicator conditions questionnaire, screening was performed using rapid tests in those with a positive questionnaire and all those over 50 years of age. We assessed the number of tests performed, the NHCVD and the number of patients with previous diagnosis but not conscious or not linked to care. The NHCVD rates (NHCVD/test performed) were also estimated.

**Results: **Seven thousand nine hundred and ninety‐one individuals were included, and a total of 4718 tests were performed (61.4% with a positive questionnaire, 38.6% for being >50 years), with a positive result in 56 (1.19%). The 65% were women and the median age was 51 years (IQR 38 to 58). Among total positive results, four (7.1%) were false positive, 15 (26.8%) were individuals with known HCV infection already treated and cured, 27 (48.2%) had at least one laboratory determination positive for HCV but they were not aware or linked to care, and 10 (17.9%) were NHCVD. Table 1 shows the prevalence by groups.

**Table:** Abstract P247 – Table 1. Estimation of prevalence and diagnostic rate of HCV by groups

	Total population (n = 7991)	Anti HCV+					
Global prevalence (n = 52)		Previous diagnosis not treated prevalence (n = 27)		New HCV diagnosis prevalence (n = 10)	
	N (%)	N	Rate per 1000 (95% CI)	N	Rate per 1000 (95% CI)	N	Rate per 1000 (95% CI)
Sex							
Male	2729 (34)	30	10.99 (7.06 to 14.93)	12	4.40 (1.91 to 6.89)	5	1.83 (0.23 to 3.44)
Female	5262 (66)	22	4.18 (2.43 to 5.93)	15	2.85 (1.41 to 4.29)	5	0.95 (0.12 to 1.78)
Age, years							
18 to 30	1652 (21)	1	0.61 (0 to 1.79)	0	‐	0	‐
31 to 40	1814 (23)	4	2.21 (0.04 to 4.37)	2	1.10 (0 to 2.63)	1	0.55 (0 to 1.63)
41 to 50	1967 (25)	15	7.63 (3.77 to 11.5)	9	4.58 (1.59 to 7.56)	3	1.53 (0 to 3.25)
51 to 60	1729 (21)	29	16.8 (10.7 to 22.9)	16	9.25 (4.72 to 13.79)	6	3.47 (0.69 to 6.25)
>60	829 (10)	3	3.62 (0 to 7.71)	0	‐	0	‐
Country							
Spain	6032 (78)	46	7.63 (5.42 to 9.83)	24	3.98 (2.39 to 5.57)	8	1.33 (0.41 to 2.25)
Eastern Europe	181 (2)	4	5.52 (0 to 16.35)	2	11.05 (0 to 26.36)	2	11.05 (0 to 26.36)
Latin America	1228 (15)	1	0.81 (0 to 2.41)	1	0.81 (0 to 2.41)	0	‐
Africa	86 (1)	1	11.63 (0 to 34.42)	0	‐	0	‐
Education level							
Primary	2086 (26)	26	12.46 (7.67 to 17.26)	15	7.19 (3.55 to 10.83)	4	1.92 (0.04 to 3.80)
Secondary	3243 (41)	21	6.48 (3.71 to 9.25)	10	3.08 (1.17 to 4.99)	4	1.23 (0.02 to 2.44)
Higher	2662 (33)	5	1.88 (0.23 to 3.52)	2	0.75 (0 to 1.79)	2	0.75 (0 to 1.79)
Total	7991 (100)	52	6.51 (4.74 to 8.28)	27	3.38 (2.10 to 4.65)	10	1.25 (0.48 to 2.03)

**Conclusion: **In early era of direct‐acting antivirals for the treatment of HCV infection, the prevalence of both new HCV diagnosis and patients not aware or not linked to care remains high. The population with the highest rates were males, 51 to 60 years old, from Spain or sub‐Saharan Africa and with a low level of education.

## P248

### Challenges in the micro‐elimination of HCV in HIV co‐infected individuals

**D Basoulis^1^, M Papadopoulou^1^, E Cholongitas^1^, G Kalamitsis^2^, G Daikos^1^ and M Psichogiou^1^**

^1^1st Internal Medicine Department, Athens Laiko General Hospital, Athens, Greece^2^Hellenic Liver Patient Association ‘Prometheus’, Athens, Greece

**Background: **In 2011 an epidemic outbreak of HIV infection amongst intravenous drug users (IDU) took place in Athens, Greece, increasing the burden of HIV‐HCV co‐infection and overcrowding the infectious diseases clinics [1]. Recent national policy changes have permitted the enrolment of HIV‐infected patients into HCV treatment programmes with direct acting antivirals (DAA) regardless of fibrosis status, allowing for micro‐elimination attempts to flourish [2,3].

**Materials and methods: **To facilitate this endeavour, we dedicated one of our clinic days solely to HCV treatment and made incremental steps to access the full range of health care services to individuals’ needs. Elastography was offered by a community organisation (Prometheus) at our practice. We systematically sought and arranged appointments and made sure that our patients adhere to treatment by frequently contacting them for encouragement and for rescheduling appointments if missed.

**Results: **One hundred and ninety‐six patients with co‐infection were referred to our centre in a median time of 1.1 months (interquartile range [IQR] 0.00 to 3.4) after diagnosis. 94.4% (185/196) were IDUs. One hundred and sixty (81.6%) were male, predominantly of Greek descent (86.2%), with a mean age of 34.3 ± 8.27 years. Twenty‐seven (13.8%) attended only one visit. One hundred and sixty‐nine patients were successfully linked (>1 visit) and 159 (94.1%) started HAART. Thirty (18.9%) discontinued, and four (2.5%) were transferred to a different clinic. One hundred and twenty‐five (63.7%) of 196 patients were retained in our care with a median follow‐up of 48.4 months (IQR 21.9 to 59.6) and were all receiving HAART. In 108 (93.9%), HCV‐RNA was evaluated. Of these, nine (8.3%) had an undetectable viral load. Median HCV‐RNA load was 6.18 log10 IU/mL (IQR 5.71 to 6.61). Liver stiffness was measured in 59 patients (47.2%) with a median of 6.1 kPa (IQR 5.2 to 8.6, range 10.6). Ninety‐nine (91.3%) of 108 patients were tested for genotype. Our population was distributed as follows: 1a, 40.5%; 3, 38.2%; 4, 19.1%; mixed genotype 1 and 3, 2.2%. 89.9% (89/99) were treated for HCV infection. Five were treated with Peg‐interferon/ribavirin successfully, while 84/89 (94.4%) received DAA treatment. 47.6% (40/84) have already completed the antiviral scheme, two discontinued due to compliance issues and 42 are still undergoing treatment. Eleven have achieved successful SVR, while it is pending for the rest.

**Conclusions: **Micro‐elimination is achievable only in patients retained to care. A large population of co‐infected IDUs is not retained to care and micro‐elimination seems challenging. Novel models of care are needed to link and retain key populations reducing barriers and to allow the therapeutic advances to deliver a public health benefit.

**References**

[1] Nikolopoulos G, Tsiara C, Paraskeva D, Sypsa V, Psichogiou M, Paraskevis D, et al. Rapid decline in HIV incidence among persons who inject drugs during a fast‐track combination prevention program after an HIV outbreak in Athens. J Infect Dis. 2017;215:1496‐505.

[2] Marshall AD, Pawlotsky JM, Lazarus JV, Aghemo A, Dore GJ, Grebely J. The removal of DAA restrictions in Europe ‐ one step closer to eliminating HCV as a major public health threat. J Hepatol. 2018 Jun 28;https://doi.org/10.1016/j.jhep.2018.06.016. [Epub ahead of print].

[3] Lazarus JV, Wiktor S, Colombo M, Thursz M; EASL International Liver Foundation. Micro‐elimination ‐ a path to global elimination of hepatitis C. J Hepatol. 2017;67:665‐6.

## P249

### Increase in 10‐year Framingham cardiovascular risk following HCV eradication with DAA‐based therapy in HIV/HCV co‐infected patients

**T Aldamiz‐Echevarría^1^, F Tejerina^1^, L Perez^1^, C Diez^1^, P Miralles^1^, J Lopez^1^, F Parras^1^ and J Bellon^2^**

^1^Infectious Diseases, Hospital Gregorio Marañón, Madrid, Spain. ^2^Bioestadística, Instituto de Investigación Biomédica Gregorio Marañón, Madrid, Spain

**Background: **Clearance of HCV viral load can lead to a rise in cholesterol rate unmasking a hyperlipaemia condition previously blocked by the virus; but its real impact on cardiovascular risk profile is unknown. Our objective is to analyse a potential worsening in cardiovascular risk.

**Material and methods**

We performed an observational retrospective study nested in a prospective setting in all consecutive HIV‐HCV infected patients who were treated with direct antiviral agents (DAA) and achieved HCV clearance in Gregorio Marañón Hospital Madrid, from November 2014 until June 2017. All recruited patients had HIV and HCV infection, both confirmed with serology and PCR in our laboratory. HCV eradication was defined as getting sustained viral response (SVR) at Week 12 after treatment. Those patients who were on hypolipaemiant therapy before HCV treatment were excluded, even if it was removed the time on DAA therapy. Framingham score was calculated for all the included patients at baseline, and 12 weeks after treatment. Lipids and cardiovascular risk were analysed as continuous and ordinal variables. Multivariable logistic regression was used to test the association of variables with cardiovascular risk progression.

**Results: **Two hundred and ninety‐seven HIV/HCV co‐infected, not previously hypercholesterolaemic, were treated with DAA for HCV infection in Gregorio Marañón Hospital from November 2014 till June 2017 achieving SVR12. Framingham score was performed before DAA and 12 weeks after HCV treatment in 190 of them. One hundred and fifty‐one (79.5%) were men. Median age was 51.2 years (SD 5.7). Seventy‐seven (47%) were stage C for CDC score. Except for four, all included patients were on ARV treatment at the moment DAA was started. Ninety (47.9%) required a change in its ARV regimen to make it compatible to HCV treatment. One hundred and seventy‐four (93%) were former or active smokers. Thirteen (6.8%) had diabetes mellitus. Sixty‐nine (37.3%) had cirrhosis and 10 (5.4%) decompensated cirrhosis. Eighty‐three (43.6) received a PI‐containing regimen for HCV treatment. Twenty‐five of them already had IP in their ARV therapy. Median cholesterol levels were 160 mg/dL (SD 34.6) and 181.9 mg/dL (SD 97), basal and Week 12 post‐treatment. Median Framingham score was 13.9 (SD 9.2) and 16.2 (SD 10.2). Forty‐four patients (23.15%) increased their Framingham score. Logistic regression showed no association with age, sex, logHCV viral load, F3/F4 fibrosis, body mass index, treatment with a PI‐containing regimen for HCV.

**Conclusions: **Our study suggests that in HIV/HCV co‐infected patients, HCV eradication after DAA‐based therapy is associated with an increase in the 10‐year Framingham cardiovascular risk.

## P250

### HBcAb positivity is an independent risk factor for HIV viral blips in HIV‐HBV co‐infected patients on antiretroviral therapy

**V Malagnino^1^, C Cerva^1^, G Maffongelli^2^, E Teti^2^, L Foroghi Biland^1^, N Cesta^1^, M De Masi^1^, M Andreoni^1^ and L Sarmati^1^**

^1^Clinic of Infectious Diseases, Policlinico Tor Vergata of Rome, Rome, Italy^2^Department of System Medicine, Tor Vergata University, Rome, Italy

**Background: **Co‐infection with HBV and HIV is common; however, there are few data on the influence of HBV on HIV viral replication control and infection progression in the course of ART in HIV/HBV co‐infected patients.

**Materials and methods: **A retrospective analysis of HIV‐positive patients, enrolled from 2007 to June 2018 at the Tor Vergata Infection Unit, was conducted grouping patients for HBV status and recording baseline viro‐immunological features, history of virological failure, efficacy of ART to achieving viral undetectability and HIV viral blip detection (intermittent episodes of detectable viraemia between 50 and 100 copies/mL).

**Results: **Two hundred and thirty‐one patients were included, among them 10 (4.3%) were HBsAg+, 85 (36.8%) anti‐HBc+/anti‐HBs+/‐ and 136 (58.9%) HBV negative. At baseline, anti‐HBc‐positive patients were older (48 years, IQR 39 to 55, *p* = 0.0001), they had lower CD4+ cell count and CD4+ nadir (188 cells/mm^3^, IQR 78 to 334, *p* = 0.02 and 176 cells/mm^3^, IQR 52 to 284, *p* = 0.001, respectively). Compared to HBV‐negative patients, a significantly higher number of AIDS and non‐AIDS events were documented in the group of anti‐HBc‐positive subjects (41.1% vs. 19.1%, *p* = 0.002 and 56.5% vs. 28.7%, *p* = <0.0001, respectively) who, after ART initiation, reached viral undetectability in significantly longer time (six vs. four months, *p* = 0.0001). HIV viral blips were more frequent in anti‐HBc‐positive patients compared to HBV‐negative subjects (74.7% vs. 22.1%, *p* = <0.0001) (Table 1). The multivariate analysis confirm that anti‐HBc‐positive status was an independent risk factor of appearance of HIV viral blips (OR 6.01, 95% CI 3.04 to 11.9, *p* < 0.0001), while CD4+ nadir >350 cells/mm^3^ and achieving HIV viral suppression within six months of ART had a protective role from HIV viral blip appearance during ART (OR 0.17, 95% CI 0.05 to 0.57, *p* = 0.005 and OR 0.26, 95% CI 0.12 to 0.56, *p* = 0.001, respectively).

**Conclusion: **Anti‐HBc‐positive status, regardless of anti‐HBs status, CD4‐nadir <350 mm^3^ and achieving HIV viral suppression over 6 months of ART are all independent risk factors for subsequent appearance of HIV viral blips in our cohort of patients. Further studies are needed to assess how HBV replication could contribute to reduce HIV replication control [1,2].

**Table:** Abstract P250 – Table 1. Comparison between HBV‐serology population

Univariable		Multivariable	
	aOR (95% CI)	*p* value	aOR (95%)	*p* value
Age >40 years	1.67 (0.98 to 2.85)	0.057	0.84 (0.43 to 1.65)	0.62
Person who injected drugs	1.91 (0.89 to 4.13)	0.09	1.37 (0.52 to 3.58)	0.51
HIV‐RNA >10^6 copies/mL	2.48 (1.45 to 4.25)	0.001	1.52 (0.76 to 3.05)	0.23
CD4 + at baseline >350/mm^3^	0.35 (0.19 to 0.64)	0.001	1.85 (0.65 to 5.24)	0.24
CD4 + nadir >350/mm^3^	0.17 (0.08 to 0.37)	<0.0001	0.17 (0.05 to 0.57)	0.005
Undetectability within 6 months	0.17 (0.08 to 0.33)	<0.0001	0.26 (0.12 to 0.56)	0.001
Occult hepatitis B virus^a^	8.1 (4.3 to 14.9)	<0.0001	6.01 (3.04 to 11.9)	<0.0001

^a^know as HBV serology HBsAg‐/antiHBc+/anti, regardless of antiHBs status.

**References**

[1] Morsica G, Ancarani F, Bagaglio S, Maracci M, Cicconi P, Cozzi Lepri A, et al. Occult hepatitis B virus infection in a cohort of HIV‐positive patients: correlation with hepatitis C virus coinfection, virological and immunological features. Infection. 2009;37:445‐9.

[2] Makvandi M. Update on occult hepatitis B virus infection. World J Gastroenterol. 2016;22:8720‐34.

## P251

### Risk of occult HBV infection reactivation in patients receiving ABC/3TC/DTG or FTC/TAF/EVG/c

**I Morrás^1^, A Gutiérrez^1^, P Mills^1^, V Moreno‐Torres^1^, S De La Fuente^1^, C Folguera^2^, A Ángel‐Moreno^1^ and A Díaz‐de Santiago^1^**

^1^Internal Medicine, Hospital Universitario Puerta de Hierro Majadahonda, Madrid, Spain^2^Pharmacy, Hospital Universitario Puerta de Hierro Majadahonda, Madrid, Spain

**Background: **Occult HBV infection (isolated anti‐HBcore) is present in up to 30% of HIV‐infected patients, higher even in IDU. The aim of our study is to evaluate proportion of HIV‐infected patients with previous contact with HBV which start ABC/3TC/DTG versus FTC/TAF/EVG/c or FTC/TDF/EVG/C for any reason in Puerta de Hierro Majadahonda University Hospital, Madrid (tertiary Public Health System centre).

**Materials and methods: **Observational and retrospective study where patients with occult HBV infection (negative HBsAg, positive anti‐HBc, negative anti‐HBs) were included. We compare HBV reactivation between 2014 and 2018 in two groups: persons which initiate ABC/3TC/DTG versus FTC/TAF/EVG/c or FTC/TDF/EVG/c (naive, simplification, toxicity, drug‐drug interactions, virological rescue). We excluded those with positive HBsAg, positive HBV DNA or are under treatment with other anti‐HBV drugs (entecavir, adefovir, interferon). Clinical and epidemiological baseline charactersitics, hepatitis serology, HIV plasma load, TCD4 cell count and HBV DNA (baseline and after study treatment was started) were collected from electronical database. Main outcome was HBsAg conversion or positive HBV plasma DNA.

**Results: **From 464 patients which start ART with ABC/3TC/DTG (n = 329), FTC/TAF/EVG/c (n = 100) or FTC/TDF/EVG/c (n = 35), 53 met inclusion criteria: one patient (1.9%) started FTC/TDF/EVG/c, 12 (22.7%) FTC/TAF/EVG/c and 40 (75.4%) ABC/3TC/DTG. Ninety percent were male, with a median age of 52 years (ICR 49 to 55), 95% Spanish, 58% IDU, 21% MSM, AIDS 31%. Seventy‐five percent showed HCV co‐infection (of which 45% showed positive blood RNA). Median time since first ART regimen was 16 years (ICR 9 to 19). Reasons for initiating study regimen were as followed: simplification 45%, toxicity 25%, drug‐drug interactions 21%, naive 3.8%. Fifty‐three percent were previously taking ART based in TDF and 47% based in ABC. Baseline viral load mean was 1.67 log (SD 1.07); 86% showed HIV load <1.3 log. Baseline TCD4 cell count was high (median 516, ICR 361 to 713), with percentage of 30 (ICR 22 to 34) and CD4/CD8 ratio of 0.58 (ICR 0.35 to 0.86). Median time of follow‐up in the study was 58 months (ICR 49 to 67), and we did not detect any HBV infection reactivation in any study arm. Five patients (9.4%) were under other immunosuppressant drugs before initiating study period, without differences between two groups. Only 9.4% modified ART study regimen along five years.

**Conclusions: **We did not observe reactivation of HBV infection in HIV‐infected patients treated with ABC/3TC/DTG, FTC/TAF/EVG/c or FTC/TDF/EVG/c for any reason along five years.

## P252

### Burden of HIV/hepatitis C co‐infection in an inner‐city sexual health clinic

**A Tomkins^1^ and V Lee^2^**

^1^The Northern Contraception, Sexual Health and HIV Service, Manchester University Foundation Trust, Salford, UK^2^The Northern Contraception, Sexual Health and HIV Service, Manchester University NHS Foundation Trust, Manchester, UK

**Background: **Despite major advances in the treatment of HCV infection in recent years, it remains a major public health concern, with 160,000 estimated to be living with chronic HCV in England. Whilst intravenous drug use (IDU) remains the greatest risk factor for acquisition, sexual transmission in MSM, particularly HIV co‐infected individuals, is increasingly reported, with an estimated prevalence of 9.9% [1]. HIV/HCV co‐infection leads to: increased HCV viral loads, more rapid liver fibrosis rates and increased risk of hepatocellular carcinoma [2]. Our inner‐city sexual health department has been providing a joint HIV/hepatology clinic to ensure appropriate management. Directly acting antivirals (DAAs) were available since 2013, however have been prioritised for individuals with the greatest need.

**Materials and methods: **A retrospective case‐note review was conducted on all new HIV/HCV co‐infected patients attending from 2007 to 2018. Demographics, risk factors for HCV acquisition, initial treatment offered, re‐infection rates and retreatments were recorded. Comparison is made pre‐ and post‐DAA availability.

**Results: **A total of 174 HIV/HCV co‐infected patients accessed our service from 2007 to April 2018, of whom 97% were male. Acute HCV was diagnosed in 70% of total patients. Reported risk factors for HCV acquisition included: being MSM (88%), a history of IDU (41%), history of being MSM and reporting IDU (30%), contact with blood/blood products (3%). There were marked increases in patients reporting being both MSM and IDU from 2013 onwards, which coincided with the reporting of chemsex regionally. Of those received pegylated interferon and ribavirin as first‐line therapy, 5% discontinued due to side effects. All virological non‐responders (16; 9.2%) to therapy were in those treated with pegylated interferon and ribavirin. All individuals receiving DAAs achieved a sustained virological response (SVR). Of the sixteen re‐infections, rates were highest amongst MSM (8; 57%) and MSM reporting IDU (4; 28.6%). Re‐infection rates were highest post‐DAA availability (11; 10.5%).

**Conclusions: **Our findings demonstrate increasing rates of HCV in MSM and those reporting IDU. As improved DAA availability occurred, fewer treatment discontinuations and increased SVRs have been witnessed. Despite these findings, we identify persistently high rates of HCV re‐infection, thus highlighting the importance of continued education, needle exchange programmes, safer sex advice, regular testing and access to retreatment.

**References**

[1] Martin NK, Thornton A, Hickman M, Sabin C, Nelson M, Cooke GS, et al. Can hepatitis C virus (HCV) direct‐acting antiviral treatment as prevention reverse the HCV epidemic among men who have sex with men in the United Kingdom? Epidemiological and modeling insights. Clin Infect Dis. 2016;62:1072‐80.

[2] Wilkins E, Nelson M, Agarwal K, Awoyemi D, Barnes E, Bhagani S, et al. British HIV Association guidelines for the management of hepatitis viruses in adults infected with HIV 2013. HIV Med. 2013;14 Suppl 4:1‐71.

## P253

### Clinical and virological characteristics of HIV‐1 positive patients with delta hepatitis

**G Morsica^1^, L Peano^2^, S Bagaglio^1^, A Poli^1^, E Messina^1^, R Vercesi^1^, H Hasson^1^ and C Uberti‐Foppa^3^**

^1^Infectious Diseases Department, San Raffaele, Scientific Institute, Milan, Italy. ^2^Mother‐Child Department, Valley Regional Hospital, Aosta, Italy. ^3^Infectious Diseases Department, University Vita Salute, Milan, Italy

**Background: **HDV infection is more frequent in HBsAg+ HIV‐infected patients, than in the counterpart of HIV‐negative patients. HDV‐infected patients have more frequently a positive serology for HCV compared to HDV‐uninfected ones. However, data on clinical and virological characteristics of multiple (HBV/HDV/HCV) infections are limited.

**Methods: **This retrospective observational study examined demographic, clinical, therapeutic information and laboratory data retrieved from the database of the Division of Infectious Diseases of the San Raffaele Hospital (CSLHIV cohort), Milan, Italy. Data were collected for each HIV‐1 infected patient at last visit available in 2017. The CSLHIV cohort was approved by the ethics committee of the San Raffaele Hospital. Results for continuous variables were reported as median (interquartile range [IQR]). Characteristics of HIV‐1 positive patients were compared using the Pearson's chi‐square or Fisher's exact test for categorical variables and the Mann‐Whitney test for continuous variables. Potential determinants for HDV positivity were examined by applying multivariate regression model. All statistical tests were two‐sided at the 5% level (*p* ≤ 0.05).

**Results: **Among 78 HBsAg+ HIV‐1 infected patients tested for anti‐HDV, 59 were anti‐HDV negative (HDV‐, 75.6%) and 19 were anti‐HDV positive (HDV+, 24.4%). Clinical characteristics of HDV+ patients and HDV‐ patients are depicted in Table 1. Multivariate analysis showed that years of ART (OR 1.22, CI 1.04 to 1.43, *p* = 0.014) was associated with anti‐HDV positivity, while sexual exposure (OR 0.08, CI 0.01 to 0.44, *p* = 0.004) was inversely associated with anti‐HDV positivity.

**Table:** Abstract P253 – Table 1. Characteristics of HIV‐1/HBsAg positive patients with or without HDV

	Overall	HDV + No pts=19	HDV ‐ No pts=59	*p*
Gender males/females	71/7	17/2	54/5	1.000
Age	51.0 (48.0 to 55.3)	53.0 (50.0 to 55.0)	51.0 (46.0 to 56.0)	0.151
Risk factor for HIV‐1 IVDU/sexual/unknown	17/39/22	11/4/4	6/35/18	0.001
Years of HIV infection	21.2 (14.4 to 27.8)	25.5 (23.0 to 30.5)	18.5 (11.5 to 25.7)	0.002
Years of ART	17.0 (9.1 to 20.7)	20.6 (16.9 to 23.7)	14.7 (7.3 to 19.4)	0.001
CD4 T cells number/mm^3^	698 (426 to 857)	535 (245 to 854)	750 (482 to 867)	0.151
ALT^a^ IU/L	34 (26 to 56)	51 (28 to 88)	32 (22 to 46)	0.021
Transient elastography KpA	7.2 (4.6 to 11.7)	9.6 (7.2 to 14.1)	5.9 (3.8 to 8.3)	0.001
Fibrosis degree F0‐F2 versus F3‐F4 (N 58)	41/17	No pts=18 9/9	No pts=40 32/8	0.020
HBeAg‐positive/negative (N72)	25/47	1/16	24/31	0.004
HIV‐RNA^b^ positive/negative	7/71	3/16	4/55	0.352
HIV‐RNA load, copies/mL	164 (60 to 5569)	352 (‐)^c^	112 (50 to 4218)	0.400
HBV‐DNA positive/negative (N 74)	19/55	No pts=17 4/13	No pts=57 15/42	1.000
HBV‐DNA load IU/mL	10 (10 to 15)	10 (10 to 42)	10 (10 to 19)	0.754
Anti‐HCV pos/neg	26/52	13/6	13/46	0.001
HCV‐RNA positive/negative	8/18	1/12	7/6	0.030
HCV‐RNA load IU/mL	114527 (401 to 446430)	173575 (‐)^d^	55478 (362 to 1830824)	1.000
FTC or 3TC/TDF+FTC^e^	8/69	8/10	0/59	0.001

IVDU = intravenous drug users; pos = positive; neg = negative; ALT = alanine aminotransferase; FTC = emtricitabine; 3TC = lamivudine; TDF = tenofovir. Results are expressed as median (IQR). *p* values according to Mann‐Whitney test or Chi‐square/Fisher's exact test, as appropriate. ^a^ALT (normal value <35 IU/L). ^b^HIV‐RNA positive >50 copies/mL. ^c^IQR was not calculated because only 3 patients had quantifiable HIV load among anti‐HDV+ patients. ^d^IQR was not calculated because only 1 HDV+ patient had a quantifiable HCV load. ^e^one patient did not receive any treatment.

**Conclusions: **HDV+ patients showed a different clinical profile respect to HDV‐ patients, being more frequently infected by HCV and exhibiting longer duration of HIV infection; additionally, HDV+ had more severe liver disease and higher necroinflammatory activity respect to HDV‐. Concerning virological interference, a suppressive effect of HDV on HCV replication was shown. Longer duration of ART and IVDU were independently associated with HDV positivity, likely reflecting an increased risk of exposure to HDV. In conclusion our data underline the need for screening and monitoring a population at high risk, as well as development of newer treatment option for HDV.

## P254

### Use of urinary albumin as a marker of renal damage in patients with chronic hepatitis C treated with direct antiviral drugs

**B Granozzi^1^, L Badia^2^, V Viotti^2^, V Guardigni^2^, P Viale^2^ and G Verucchi^2^**

^1^Department of Clinical and Experimental Medicine, S. Orsola, Bologna, Italy^2^Department of Clinical and Experimental Medicine, Alma Mater Studiorum, University of Bologna, Bologna, Italy

**Background: **Direct‐acting antiviral medications (DAAs) have revolutionised care for chronic hepatitis C virus infection. Anyway, data on kidney safety of these drugs are still scarce [1,2]. Aim of this study was to evaluate urinary albumin modifications and subclinical glomerular damage during and after treatment with DAAs.

**Material and methods**

We retrospectively evaluated patients treated with DAAs between February 2016 and November 2017 with baseline creatinine clearance >60 mL/min/1.73 m^2^ and who achieved SVR. Urinary albumin concentration and serum creatinine were measured at baseline and at the end of 12 weeks of follow‐up.

**Results: **Eighty‐seven patients with HCV infection (55.2% HCV/HIV co‐infected) were included in this study. Hypertension was the highest represented comorbidity (29.9%), followed by diabetes (10.3%). There were no significant changes in serum creatinine concentration and eGFR during therapy and at the end of follow‐up. In 40 patients (46.0%) an increase in urinary albumin value was observed; the average increase was 36.9 mg/L (range 1 to 775). Univariate analysis revealed no significant associations between an increased urine albumin value in the follow‐up and the baseline characteristics of the population (BMI, HIV infection, cirrhosis, diabetes, hypertension, antiretroviral therapies with tenofovir). Quantitative analysis of urinary albumin concentration variation showed a statistically significant correlation, at the univariate analysis, with baseline urinary albumin values (*p* < 0.001) and hypertension. Patients with higher baseline values of urinary albumin and those with hypertension showed a greater increase in these values. At the multivariate analysis the association with basal urinary albumin values (*p* < 0.001) was confirmed and the association with diabetes mellitus acquired statistical significance (*p* < 0.01), showing a greater decrease in urinary albumin values in patients with diabetes.

**Conclusion: **Urinary albumin values deteriorate in absolute terms in 46% of patients; this increase appears quantitatively related to the baseline values, while correlation with other clinical features and/or risk factors for renal disease do not emerge; therefore a risk of direct damage caused by DAAs at the glomerular level cannot be excluded. The presence of diabetes mellitus is directly associated with a greater decrease in urinary albumin values; this result can probably be explained by the improvement of glycaemic control, already revealed in some studies [3], in diabetic patients treated with regimens containing DAAs. Clinicians should be aware of possible subclinical glomerular damage during treatment with DAAs and routine monitoring of glomerular markers should be considered.

**References**

[1] Strazzulla A, Coppolino G, Barreca GS, Gentile I, Rivoli L, Postorino MC, et al. Evolution of glomerular filtration rates and neutrophil gelatinase‐ associated lipocalin during treatment with direct acting antivirals. Clin Mol Hepatol. 2018;24:151‐62.

[2] Dell’ Acqua R, de Vita G, Procopio A, Milella M, Saracino A, Angarano G. Renal function changes in HCV‐infected patients with chronic kidney disease during and after treatment with direct antiviral agents. Dig Liver Dis. 2017;49:1166‐9.

[3] Holzscheiter L, Beck C, Rutz S, Manuilova E, Domke I, Guder WG, et al. NGAL, L FABP, and KIM‐1 in comparison to established markers of renal dysfunction. Clin Chem Lab Med. 204;52:537‐46.

## P255

### Measles outbreak: are our patients at risk? An audit of viral screening 2018

**I Cormack**

HIV Department Heath Clinic, Croydon University Hospital, Croydon, UK

**Background: **In 2018 up to 17 June there have been 23 suspected cases of measles that have been reported to Public Health England (PHE) in Croydon. BHIVA guidelines recommend that HIV‐positive adults be screened for measles IgG regardless of a history of childhood vaccination and that measles seronegative patients with CD4 cell counts >200 are vaccinated with MMR. BHIVA also recommends vaccination against varicella zoster (VZV) if found to be VZVIg negative.

**Methods: **All electronic patient records (EPR) and blood test results were checked on HIV‐positive patients attending their consultant over a 6‐month period in 2018 to evaluate screening for measles, VZV, hepatitis A and B.

**Results: **Three hundred and twenty‐four HIV‐positive patients attended over a 6‐month period. One hundred and forty‐eight of 160 (92.5%) men and 152/164 (93%) women were screened for measles IgG and VZVIgG. Eight (5%) men and eight (5%) women were found to be measles IgG‐ve and eligible for vaccination. Sixteen of 16 (100%) GP letters had been completed requesting measles vaccination. Twenty‐four (7%) patients had not been screened yet and blood requests were added to their next clinic visit. Twenty‐two of 300 (7%) patients were found to be VZVIgG‐ve even with some having a documented history of childhood infection. Seventeen of 22 (77%) had a documented GP letter advising VZV vaccination. Four have not had GP letters sent yet and one has not given correct GP details so we are unable to contact. Twenty‐six MSM were found to be HepAIgG‐ve 24/26 (92%) had been offered and given hepatitis A vaccines. One patient defaulted care and one transferred care. Letters advising need for hepatitis A vaccination had been sent to their GP or next centre of care. Thirty‐two of 160 (20%) men and 45/164 (27%) women were found to be hepatitis B naive with HepBsAb <100. Two men and five women have so far declined hepatitis B vaccination. Two men and four women have defaulted care while the rest have had vaccination prescribed for their next clinic visit. Reasons for declining hepatitis B vaccination included perceived low risk and previous non‐response to vaccine course.

**Conclusion: **Five percent HIV‐positive patients in our cohort were measlesIgG‐ve and 7% were VZVIgG‐ve so will benefit from vaccination. Hepatitis A vaccination rate for HepAIgG‐ve MSM was high (92%). A significant number of patients were found to have inadequate protection from hepatitis B and will benefit from the hepatitis B vaccination prescribed as a result of this audit. I intend to re‐audit this in the next six months.

## P256

### Treatment of acute HCV infection with direct acting antivirals (DAA) in HIV patients

**C Gómez‐Ayerbe^1^, R Palacios^1^, F Téllez^2^, C Sayago^3^, M Ríos^4^, A Martín‐Aspas^5^, A Camacho^6^, L Muñoz^7^ and J Santos^1^**

^1^Infectious Diseases, Hospital Clínico Virgen de la Victoria/IBIMA, Málaga, Spain. ^2^Infectious Diseases, Hospital Universitario de Puerto Real, Cádiz, Spain. ^3^Infectious Diseases, Hospital Universitario Virgen de Valme, Sevilla, Spain. ^4^Infectious Diseases, Hospital Universitario Virgen de Macarena, Sevilla, Spain. ^5^Infectious Diseases, Hospital Universitario Puerta del Mar, Cádiz, Spain. ^6^Infectious Diseases, Hospital Universitario Reina Sofia, Córdoba, Spain. ^7^Infectious Diseases, Hospital Universitario San Cecilio, Granada, Spain

**Background and objective**

Acute HCV infection is often asymptomatic and may be undetected unless periodic screening is performed. Early treatment (in acute phase) achieves sustained virological response (SVR) in a high proportion of cases even with short regimens and drastically reduces the infective time of subjects. The aim of this study is to describe cases of acute HCV infection treated with DAA in seven Andalusian (Spain) hospitals.

**Patients and methods**

Retrospective, multicentric study of HIV‐infected patients treated with DAA during HCV acute infection (in the first six months after diagnosis), from November 2015 to December 2017. Epidemiological, clinical, analytical, therapeutic and evolutionary variables were analysed.

**Results: **Eighteen episodes of acute HCV infection in 17 patients (three re‐infection cases) were included; all were MSM, with a mean age of 41.5 (±8) years and a median HIV infection time of 36.9 months (IQR 23.5 to 76.6). All were on ART with undetectable viral load except two cases (65 and 68 copies/mL) and median CD4 cell count was 762 cells/mm^3^ (IQR 579 to 959). Eleven patients (64.7%) had previous or coincidental episodes of other sexually transmitted infections (STIs): 10 syphilis, two venereum lymphogranuloma, one chlamydia and two gonococcus. Acute HCV infection was asymptomatic in 14 (77.7%) cases. Distribution of genotypes was 50% for G1a and G4, respectively. The median time to HCV treatment was 4.2 (1.6 to 5.5) months and the median baseline HCV‐RNA was 5.68 log (IQR 5.21 to 6.29). Treatment with SOF‐LDV was prescribed in 15 episodes (8 weeks in eight patients and 12 weeks in seven), GRZ/ELB for 12 weeks was prescribed in two patients and one patient received 2D + RBV. All patients presented SVR except the one treated with 2D + RBV (94.4%). There was no discontinuation due to adverse effects.

**Conclusions: **All episodes of acute HCV infection were in MSM with good immuno‐virological situation. More than half of the subjects presented a history of other STIs. HCV infection was asymptomatic in a high percentage of cases, forcing systematic screening in certain populations. Treatment of acute HCV infection with DAA was effective and safe.

## P257

### Improving the care cascade of hepatitis C management among HIV‐HCV co‐infected persons by facilitating access to direct acting agents (DAAs): a real‐life, single centre experience

**K Protopapas, P Kazakou, K Thomas, D Kavatha, A Chounta, G Zampetas, C Oikonomoulou, C Moschopoulos, A Papadopoulos and A Antoniadou**

4th Department of Internal Medicine, Attikon University Hospital, Athens, Greece

**Background: **Novel DAAs offer improved tolerability and sustained virological responses (SVR) over prior interferon‐based therapies for HCV and a unique opportunity for cure and improved prognosis for co‐infected with HCV and HIV patients. In Greece up to the end of 2017 access to DAAs by reimbursement was limited only for patients with chronic hepatitis C and advanced fibrosis and the majority of co‐infected patients (of whom most are drug users [IVDUs]) lacked the chance to be treated. This changed in 2018 and all co‐infected patients have free access to DAAs. This is a retrospective cohort study of the impact of this new strategy in the care cascade of hepatits C in co‐infected patients from a single centre in Athens.

**Material and methods**

All persons diagnosed with HIV and HCV infection in an HIV unit in Athens were recorded and demographic characteristics, HIV infection parameters and hepatitis C management were evaluated before and after free access to DAAs (in September 2017 and June 2018).

**Results: **Among 1167 persons with HIV infection 142 (12%) were diagnosed with co‐infection. The incidence over time of the diagnosis of the co‐infection followed the epidemic pattern of HIV infection among IVDUs in Athens (67% of new cases between 2011 and 2013). Mean age of co‐infected persons was 36 years, 82% were male and 80% were IVDUs with 63% engaged in active substance use. It is a difficult‐to‐manage patient group with 91.5% without insurance or supported by welfare, 79% unemployed and 49% with late presentation. Mean CD4 count at diagnosis was 456 and mean HIV‐RNA was 5 log. Sixteen patients died (11%) mostly because of drug use, 36 (25%) were lost to follow‐up and 90 (63.3%) were retained to care, all receiving antiretroviral treatment with 85% viral suppression. The availability of DAAs through electronic application and rapid approval increased significantly (*p* < 0.001) the measurement of HIV‐RNA, genotyping, fibrosis staging and the administration of DAAs (from one patient until the end of 2017 to 21 the first six months of 2018 [SVR 95%]). Patients cured from hepatitis C increased from nine to 27 in 2018 (from 10% to 30%, *p* < 0.001). No re‐infections have been noted. It is expected all patients to be treated in a year.

**Conclusion: **Facilitating access to DAAs is a successful strategy for the elimination of hepatitis C even in a difficult‐to‐manage patient group as the co‐infected HIV‐HCV IVDUs.

## CLINICAL PHARMACOLOGY

## P258

### Using mechanistic physiologically‐based pharmacokinetic models to assess prenatal drug exposure: thalidomide versus efavirenz as case studies

**S Atoyebi^1^, A Olagunju^1^, R Rajoli^2^, E Adejuyigbe^3^, A Owen^2^, O Bolaji^1^ and M Siccardi^2^**

^1^Department of Pharmaceutical Chemistry, Obafemi Awolowo University, Ile Ife, Nigeria^2^Department of Molecular and Clinical Pharmacology, University of Liverpool, Liverpool, UK^3^Department of Paediatrics and Child Health, Obafemi Awolowo University, Ile Ife, Nigeria

**Background: **Physiologically‐based pharmacokinetic (PBPK) modelling approach was used to assess prenatal drug exposure using efavirenz and thalidomide.

**Method: **Maternofoetal PBPK models integrating multi‐compartmental maternal and foetal units were developed using Simbiology^®^. Processes governing drug disposition were described using differential equations with key system and drug‐specific parameters. Transplacental drug transfer was modelled as bidirectional passive diffusion. Predicted pharmacokinetic parameters were validated against clinically observed data with two‐fold difference acceptance threshold. Key indices of foetal exposure were computed.

**Results: **Model predictions for key pharmacokinetic parameters during pregnancy were generally within two‐fold difference of clinically observed figures. Foetal exposure to efavirenz was lower during the second trimester than the third with median (range) foetal‐to‐maternal plasma ratio of 0.62 (0.12 to 1.01) versus 1.24 (1.02 to 1.41), and AUC0–24 of 29.9 mg.h/L (6.12 to 196) versus 49.1 mg.h/L (23.0–192) at 600 mg maternal dose (Table 1). Foetal exposure to thalidomide was higher than efavirenz (Figure 1), with second and third trimesters foetal‐to‐maternal plasma ratios of 2.36 (1.94 to 3.74) and 2.46 (2.03 to 3.60) respectively at 200 mg (Table 2).

**Conclusion: **Model predictions indicated significantly higher foetal exposure to thalidomide, a known teratogen, with foetal plasma concentrations above 200% of maternal plasma concentrations compared with <150% for efavirenz which has been used extensively during pregnancy. Further qualification of this approach as a tool in evaluating drug safety during pregnancy is needed.

**Abstract P258 – Figure 1.** Predicted time profile of cord‐to‐maternal blood concentration (CM) and foetal‐to‐maternal blood concentration (FM) ratios across the dosing interval with maternal dose of 600 mg efavirenz and 200 mg thalidomide during pregnancy. Data presented as mean (SD).

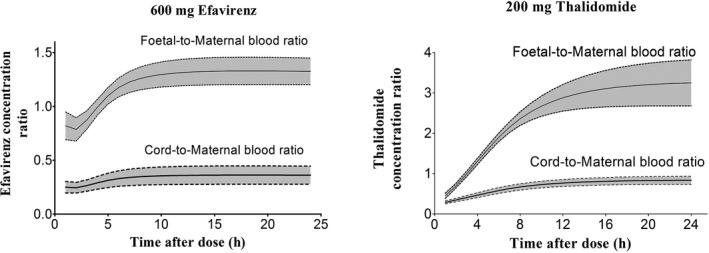

**Table:** Abstract P258 – Table 1. Predicted indices of foetal exposure to 600 mg efavirenz in the foetal plasma and umbilical cord during pregnancy. Data presented as median (range)

Pharmacokinetic parameter	Second trimester n = 100	Third trimester n = 100
Foetal plasma		
Efavirenz concentration (mg/L)	1.28 (0.264 to 8.50)	2.11 (0.984 to 8.35)
AUC (mg.h/L)	29.9 (6.12 to 196)	49.1 (23.0 to 193)
F:M ratio	0.62 (0.12 to 1.01)	1.24 (1.02 to 1.41)
Umbilical vein		
Efavirenz concentration (mg/L)	0.192 (0.019 to 1.50)	0.540 (0.242 to 2.03)
AUC (mg.h/L)	4.47 (0.44 to 34.6)	12.6 (5.65 to 46.8)
C:M ratio	0.08 (0.01 to 0.19)	0.34 (0.21 to 0.47)

**Table:** Abstract P258 – Table 2. Predicted indices of foetal exposure to 200 mg thalidomide in the foetal plasma and umbilical cord during pregnancy. Data presented as median (range)

Pharmacokinetic parameter	Second trimester n = 100	Third trimester n = 100
Foetal plasma		
Thalidomide concentration (mg/L)	0.909 (0.023 to 4.51)	0.859 (0.030 to 3.87)
AUC (mg.h/L)	26.7 (17.6 to 43.6)	26.2 (17.9 to 37.3)
F:M ratio	2.36 (1.94 to 3.74)	2.46 (2.03 to 3.60)
Umbilical vein		
Thalidomide concentration (mg/L)	0.261 (0.005 to 1.75)	0.248 (0.006 to 1.21)
AUC (mg.h/L)	8.29 (4.95 to 15.5)	8.05 (4.78 to 13.8)
C:M ratio	0.69 (0.44 to 0.90)	0.69 (0.53 to 0.90)

## P259

### Evaluation of the concentrations of psychotropic drugs in HIV‐infected versus HIV‐negative patients: potential implications for clinical practice

**D Cattaneo^1^, S Baldelli^1^, C Resnati^2^, A Giacomelli^2^, P Meraviglia^2^, D Minisci^2^, N Astuti^2^, A Ridolfo^2^, G De Socio^3^, E Clementi^1^, M Galli^2^ and C Gervasoni^2^**

^1^Laboratory Medicine, Luigi Sacco University Hospital, Milano, Italy. ^2^Infectious Diseases, Luigi Sacco University Hospital, Milano, Italy. ^3^Infectious Diseases, Azienda Ospedaliero‐Universitaria di Perugia, Perugia, Italy

**Background: **The management of psychiatric illness in HIV‐infected patients is clinically challenging because of the risk of potential drug‐drug interactions. This may result in the selection of inadequate psychotropic drug doses, eventually causing suboptimal drug exposure. Here, we aimed to measure the antidepressant and/or antipsychotic drug concentrations in HIV‐infected patients during routine outpatient visits.

**Materials and methods: **Six hundred HIV‐infected patients were screened during the first 15 months after the introduction of our outpatient polytherapy management service in a search for subjects treated with psychotropic drugs for at least three months. The self‐reported data concerning adherence to therapies were compared with data provided by our Pharmacy Department. The distribution of psychotropic drug concentrations in HIV‐infected patients was compared with that observed in a control group of HIV‐negative patients monitored over the same period.

**Results: **The search identified 82 HIV‐infected patients concomitantly receiving antiretroviral and psychotropic drug treatment. They were given psychotropic drugs for the treatment of major depression (nine patients), dysthymic disorders (34), schizophrenia spectrum disorders (seven), bipolar disorders (30) or panic attacks (two). Trough concentrations of all antiretrovirals fell within or close to the therapeutic ranges used in our laboratory, and patients showed optimal compliance to therapies. As shown in Table 1, a total of 55% of the samples taken from the HIV‐infected patients had trough psychotropic levels below the minimum effective drug concentrations. Conversely, only 26% of the trough psychotropic levels measured in the 453 HIV‐negative patients were below the minimum effective drug concentration. Interestingly, 61% of the patients concomitantly treated with cobicistat or ritonavir‐based regimens, 44% of those receiving NNRTIs and 54% of those treated with INIs had sub‐therapeutic trough plasma psychotropic concentrations (*p* < 0.05: NNRTIs vs. the other groups), and respectively 5%, 16% and 2% had had trough plasma psychotropic concentrations above the upper threshold of the therapeutic range (*p* < 0.05: INIs vs. the other groups).

**Table:** **Abstract P259 –** Table 1. Distribution of psychotropic drug concentrations in the 82 HIV‐infected patients of the GAP cohort and the 423 HIV‐negative controls receiving maintenance antidepressant and/or antipsychotic therapy

Psychotropic drug	HIV‐positive patients (n)	Daily drug doses	Trough levels (ng/mL)	Reference ranges (ng/mL)	Sub‐therapeutic samples (%)	HIV‐negative patients (n)	Trough levels (ng/mL)	Sub‐therapeutic samples (%)
Citalopram	15	10 to 20 mg	65 ± 67	50 to 110	60%^a^	50	73 ± 58	34%
Duloxetine	8	60 to 90 mg	32 ± 35	30 to 120	63%	19	68 ± 41	32%
Fluoxetine	5	20 to 40 mg	204 ± 190	120 to 500	50%	14	250 ± 160	21%
Paroxetine	13	20 to 40 mg	22 ± 20	20 to 65	54%	21	150 ± 116	33%
Sertraline	10	50 to 200 mg	20 ± 12	10 to 150	20%^a^	85	47 ± 43	6%
Venlafaxine	4	75 to 150 mg	223 ± 52	100 to 400	0%	44	288 ± 239	23%
Haloperidol	7	2 to 5 mg	1.4 ± 0.5	1 to 10	57%^a^	41	4.1 ± 2.6	5%
Olanzapine	8	2.5 to 20 mg	16 ± 16	20 to 80	88%^a^	37	47 ± 66	46%
Quetiapine	12	25 to 200 mg	266 ± 225	100 to 500	46%	112	211 ± 51	31%

a*p* < 0.05 versus HIV‐negative controls.

**Conclusions: **Our study showed that in real‐life setting, psychotropic drug concentrations are likely to be associated with a higher rate of sub‐therapeutic concentrations compared with HIV‐negative patients. Such results were not related to patients’ compliance or to drug‐drug interactions. Therapeutic drug monitoring also of antidepressants or antipsychotics can significantly contribute to the optimal management of such patients by allowing the rational selection of the best psychotropic drug dose in individual cases.

## P260

### Pharmacokinetics (PK) of bictegravir (BIC) in combination with polyvalent cation containing (PVCC) antacids and supplements

**A Mathias^1^, J Lutz^1^, S West^2^, D Xiao^1^, S Chuck^3^, H Martin^4^, E Quirk^4^ and B Kearney^1^**

^1^Clinical Pharmacology, Gilead Sciences, Foster City, CA, USA. ^2^Biostatistics, Gilead Sciences, Foster City, CA, USA. ^3^Medical Affairs, Gilead Sciences, Foster City, CA, USA. ^4^Clinical Research, Gilead Sciences, Foster City, CA, USA

**Background: **BIC is a potent, unboosted integrase strand transfer inhibitor (INSTI) with a high barrier to resistance in the HIV single‐tablet regimen BIC/emtricitabine/tenofovir alafenamide (B/F/TAF). BIC has a wide therapeutic‐efficacy range and high mean (%CV) inhibitory quotient (IQ) of 16.1 (35.2%); where IQ is the trough plasma concentration over the protein adjusted concentration that results in 95% inhibition of wild‐type HIV‐1 naïve virus (paIC95). Absorption of INSTIs, including BIC, can be lowered via chelation by the high concentrations of di/trivalent cations contained in certain antacids or supplements. The effect of polyvalent cations on BIC exposures administered under various dosing conditions (i.e. fasted vs. fed and simultaneous vs. staggered administration) was evaluated in healthy volunteers.

**Materials and methods: **B/F/TAF 50/200/25 mg was administered to healthy volunteers (N = 14/cohort) simultaneously under fasted or fed conditions, with/without maximum strength antacid (aluminum hydroxide 1600 mg, magnesium hydroxide 1600 mg, simethicone 160 mg), calcium carbonate (1200 mg) or ferrous fumarate (324 mg). Additionally, the effect of staggering B/F/TAF ±2 hours from maximum strength antacid under fasted conditions was evaluated in healthy volunteers (N = 14). BIC area under the plasma concentration versus time curves from time zero to infinity (AUC) for each treatment were determined and compared to B/F/TAF administered alone fasted (control). Geometric least‐squares mean (GLSM) ratios of treatment versus control and 90% confidence intervals (CI) were calculated.

**Results: **B/F/TAF co‐administered simultaneously with certain cations (antacid, ferrous fumarate) under fasted conditions substantially reduced (63 to 79%) BIC exposures (Table 1). Under fed conditions, BIC exposures were reduced modestly (47%) with antacid and were unaffected by ferrous fumarate or calcium carbonate supplements. When B/F/TAF was administered in the fasted state 2 hours after antacid, mean BIC exposures were reduced 52% but remain projected to be substantially above paIC95 (IQ = 7.7) and the lower IQ values associated with efficacy in the B/F/TAF registrational trials (lowest observed IQ = 4.7). BIC exposures were not affected by B/F/TAF fasted administration 2 hours before antacid. All study treatments were well tolerated.

**Conclusions: **The high IQ values and associated efficacy of BIC in the B/F/TAF registrational trials supports its flexible use in patients using PVCC antacids/supplements through co‐administration either fed or fasted when staggered ±2 hours.

**Table:** Abstract P260 – Table 1. Bictegravir AUC % geometric least squares mean (% GLSM) ratio of test treatment (antacid/supplement, staggered administration and/or food),  as compared to B/F/TAF alone under fasted conditions (reference)

B/F/TAF Dosing	Diet	Cation	BIC AUC GLSM ratio (90% CI)	Projected mean IQ with indicated diet/cation regimen^a^
Simultaneous	Fasted	Antacid	0.21 (0.18 to 0.26)	3.4
		Fe supplement	0.37 (0.33 to 0.42)	6.0
		Ca supplement	0.67 (0.57 to 0.78)	10.8
Simultaneous	Fed	Antacid	0.53 (0.44 to 0.64)	8.5
		Fe supplement	0.84 (0.74 to 0.95)	13.5
		Ca supplement	1.03 (0.89 to 1.20)	16.6
2 hours before	Fasted	Antacid	0.87 (0.81 to 0.93)	14.0
2 hours after			0.48 (0.38 to 0.59)	7.7

^a^calculated via application of the BIC AUC GLSM ratio to the mean BIC IQ from the B/F/TAF registrational trials (IQ = 16.1).

## P261

### Impact of mild, moderate and severe renal impairment and hemodialysis on temsavir pharmacokinetics following oral administration of fostemsavir, an attachment inhibitor for heavily treatment‐experienced HIV‐1 infected patients

**K Moore^1^, M Magee^2^, M Gunshenan^3^, H Sevinsky^4^, C Llamoso^5^ and P Ackerman^5^**

^1^Clinical Pharmacology, ViiV Healthcare, Research Triangle Park, NC, USA. ^2^Clinical Pharmacology, GlaxoSmithKline, Upper Providence, PA, USA. ^3^Statistics and Programming, GlaxoSmithKline, Upper Providence, PA, USA. ^4^Clinical Pharmacology, Arbutus, Warminster, PA, USA. ^5^Clinical Development, ViiV Healthcare, Branford, CT, USA

**Background: **Fostemsavir (FTR) is a prodrug of temsavir (TMR), a first‐in‐class attachment inhibitor that binds directly to HIV‐1 gp120, preventing initial viral attachment and entry into host CD4+ T cells. Phase III results in heavily treatment‐experienced (HTE) HIV‐1 infected patients at Week 24 was 54% receiving FTR + optimized background therapy achieved HIV‐1 RNA <40 copies/mL; 71% and 77% achieved HIV‐1 RNA <200 copies/mL and <400 copies/mL, respectively. TMR is primarily eliminated via biotransformation and excreted as metabolites in the urine (44 to 51%) and feces (33%) with <2% as unchanged TMR; the impact of RI, a comorbidity in HTE patients, on TMR PK is important to assess.

**Materials and methods: **Study 206217 (NCT02674581) was an open‐label, parallel‐group study in HIV‐seronegative adults with varying degrees of renal function with estimated glomerular filtration rate (eGFR) determined by Modification of Diet in Renal Disease formula (normal ≥90, mild 60 to 89, moderate 30 to 59 and severe), RI (≤29 mL/min/1.73 m^2^, not on hemodialysis [HD]), and end‐stage renal dysfunction (≤29 mL/min/1.73 m^2^ on HD per protocol). Subjects (excluding HD) received a single oral dose of FTR 600 mg extended‐release (ER) tablet with a standard meal on Day 1. Those on HD received FTR after HD session during Period 1 separated by up to seven days and then 4 hours prior to HD session during Period 2. Serial blood samples were collected up to 96 hours post‐dose. Total and unbound TMR PK were derived by noncompartmental methods. Linear regression analysis of pooled data (excluding HD) was used to estimate the effect of RI on TMR PK. Categorical analysis was also assessed including the effect of HD on TMR PK between periods.

**Results: **Thirty subjects were dosed (6/group); 29 completed the study. eGFR‐based regression analysis showed ≤2% change in total and 15% change in unbound TMR AUC (INF) or Cmax in subjects with RI, respectively (Figure 1). Protein binding tended to decrease with increasing RI (mean unbound fraction: 11.8% normal, 12.3% mild, 13% moderate, 19% severe, 16% on HD). Categorical analysis showed a 43% decrease in TMR renal clearance in subjects with severe RI. HD was associated with an 11% reduction in total TMR AUC (INF) and 46% increase in Cmax. No new safety signals associated with FTR were observed.

**Abstract P261 – Figure 1.** Effect of renal impairment on total and unbound TMR PK parameters based on eGFR regression analysis.

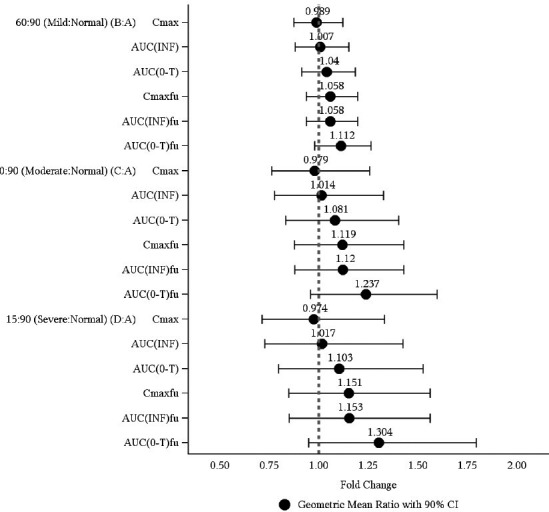

**Conclusion: **In HTE patients, FTR can be administered without dose adjustment in patients with renal impairment, including those on hemodialysis.

## P262

### No clinically relevant effect of subject demographic or disease covariates on the exposures of bictegravir and tenofovir alafenamide following administration of a B/F/TAF fixed dose combination to HIV‐1 infected subjects

**J Lutz^1^, B Kirby^1^, Y Shao^2^, Y Gao^3^, E Quirk^4^ and A Mathias^1^**

^1^Clinical Pharmacology, Gilead Sciences, Foster City, CA, USA. ^2^Biostatistics, Gilead Sciences, Foster City, CA, USA. ^3^Consulting Services, Certara, Menlo Park, CA, USA. ^4^Clinical Research, Gilead Sciences, Foster City, CA, USA

**Background: **Bictegravir (BIC, B) is a potent unboosted HIV integrase strand transfer inhibitor with a high barrier to resistance and low potential for drug interactions. When coformulated with the nucleoside analog reverse transcriptase inhibitors, emtricitabine (F) and tenofovir alafenamide (TAF), BIC demonstrated high efficacy with no resistance development in Phase III clinical trials. A population‐based pharmacokinetic (PK) model was developed to understand the clinical covariates of the PK of BIC and TAF in HIV‐1 infected subjects when administered once daily as B/F/TAF 50/200/25 mg, a fixed dose combination currently under regulatory review for the treatment of HIV‐1 infection.

**Materials and methods: **Population PK models for BIC and TAF were developed using pooled intensive and sparse plasma concentration data (8752 and 4201 observations, respectively) from 18 Phase I and III studies in heathy volunteers and subjects with HIV‐1 infection (BIC N = 1193 and TAF N = 486). A nonlinear mixed effects modeling approach using a first‐order conditional estimation with interaction (FOCE‐I) method in NONMEM v.7.3 was employed. The effect of demographic and disease covariates, including age, sex, body weight (BW), body mass index, race, ethnicity, glomerular filtration rate, fasting versus fed administration status, infection status (HIV+ vs. HIV‐), antiretroviral treatment status (naïve vs. virally suppressed), HBV and HCV co‐infection status, baseline HIV‐1 RNA, baseline CD4 count, concomitant H2‐receptor antagonist (H2RA) administration and concomitant proton pump inhibitor (PPI) administration on the PK of BIC and TAF were evaluated.

**Results: **The final population PK models appropriately describe BIC and TAF plasma concentration data. Only infection status, BW and concomitant PPI administration were statistically significant covariates on BIC PK (*p* < 0.01). Only infection status and sex were statistically significant covariates on TAF PK (*p* < 0.01). No clinically relevant patient differences in BIC or TAF PK were observed with respect to the above covariates. The mean BIC AUCtau for the highest (N = 297) and lowest (N = 300) BW quartiles differed by <30% (90 and 116 ug*hr/mL, respectively) and mean BIC Cmax between subjects with (N = 109) or without (N = 1084) concomitant PPI administration differed by <10% (5.6 and 6.2 ug/mL, respectively). The mean TAF AUCtau between males (N = 439) and females (N = 47) differed by <15% (140 and 160 ng*hr/mL, respectively).

**Conclusions: **All demographic and disease covariates were determined to have no clinically relevant impact on BIC or TAF exposure in HIV‐infected subjects. No dose adjustment of BIC or TAF due to the evaluated subject demographics or disease severity is necessary.

## P263

### Using Climate‐HIV to describe non‐antiretroviral use and potential DDIs for people living with HIV within a UK cohort

**C Okoli^1^, S Khoo^2^, A Schwenk^3^, M Radford^1^, M Myland^4^, S Taylor^5^, A Darley^6^, J Barnes^5^, A Fox^6^, F Grimson^4^, I Reeves^7^, S Munshi^7^, A Croucher^7^, N Boxall^4^, A Paice^1^, J van Wyk^1^ and P Benn^1^**

^1^Medical, ViiV Healthcare, Middlesex, UK. ^2^Pharmacology, University of Liverpool, Liverpool, UK. ^3^Alexander Pringle Centre, North Middlesex University Hospital NHS Trust, London, UK. ^4^Real World Insights, IQVIA, UK & Ireland, London, UK. ^5^Birmingham Heartlands HIV Service, Birmingham Heartlands Hospital, University Hospitals Birmingham NHS Foundation Trust, Birmingham, UK. ^6^Nottingham University Hospitals NHS Trust, Nottingham, UK. ^7^Homerton University Hospital NHS Foundation Trust, London, UK

**Background: **Polypharmacy is associated with ageing, increased hospitalisations and increased drug‐drug interactions (DDIs) in the general population. Healthcare professionals (HCPs) involved in the care of PLWHIV need to be aware of the potential for DDIs with ARVs especially in an ageing population. Effective collaboration and communication between HCPs and patients is required to avoid or manage DDIs. Climate‐HIV is an electronic patient record system supporting the management of PLWHIV in specialised services. This study aims to describe the use of non‐ARVs among a cohort of PLWHIV and the number of potential and actual DDIs.

**Methods: **This cross‐sectional study included PLWHIV who were ≥18 years and had an ARV record current in February 2018, from those attending four HIV units using Climate‐HIV. Data regarding demographics, ARV regimen, comorbidities and comedication were collected. The most common ARV regimens and non‐ARVs were reviewed for DDIs and categorised as red, amber, yellow and green in accordance to the significance of interaction as per the Liverpool website. Additionally, non‐ARVs recommended in the NICE guidelines with respect to observed comorbidities were reviewed for DDI potential.

**Results: **Four thousand six hundred and thirty patients were included in this analysis, 2021 (44%) were female and 1898 (41%) were aged over 50 years. Two thousand and sixty‐four (45%) and 1582 (34%) were of black‐African and white ethnicity respectively. Patients <50 years old had recorded their (median) third (IQR 2 to 4) regimen at time of analysis, while those ≥50 years recorded their fourth (IQR 2 to 6) ARV regimen. Commonly reported comorbidities had a greater prevalence in the ≥50 years group. Two hundred and eighteen different non‐ARVs were recorded with 3010 (65%) of patients recorded ≥1 non‐ARV, and 787 (17%) ≥5. The median (IQR) number of non‐ARVs were 1 (0 to 2) and 2 (1 to 5) for younger and older groups, respectively. Seventy‐nine percent of the non‐ARVs were sourced outside the HIV clinic. Clear differences were observed between regimens, with cobicistat/ritonavir‐based regimens having more red and amber DDI categories when compared to raltegravir or dolutegravir‐based regimens which were mostly green. Amongst non‐ARVs specific to comorbidities observed, red DDIs were most associated with drugs used for hepatitis C, amber for mental health illnesses.

**Conclusion: **We report the frequency of polypharmacy and DDIs in a large, real‐life HIV cohort. Significant DDIs were identified with many ritonavir/cobicistat‐based regimens. Increased comorbidities and non‐ARVs were observed in older patients. Some first‐line ARV regimens differ in risk of DDIs, which could inform prescribing for PLWHIV.

**Abstract P263 – Figure 1.** Illustrates the mean number of concurrent non‐ARVs over the different age groups.

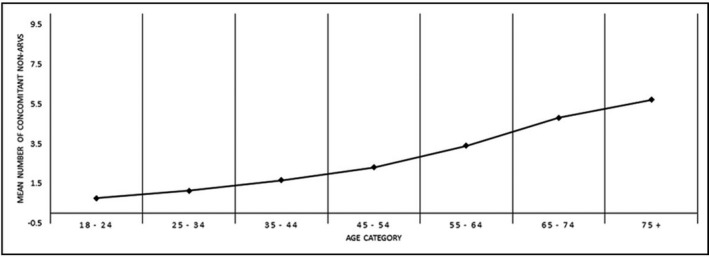

## P264

### Lack of clinically relevant drug interactions between bictegravir/emtricitabine/tenofovir alafenamide and ledipasvir/sofosbuvir or sofosbuvir/velpatasvir/voxilaprevir

**K Garrison^1^, R Humeniuk^1^, S West^2^, L Wei^2^, J Ling^1^, H Graham^3^, H Martin^3^, L Stamm^3^ and A Mathias^1^**

^1^Clinical Pharmacology, Gilead Sciences, Foster City, CA, USA. ^2^Biostatistics, Gilead Sciences, Foster City, CA, USA. ^3^Clinical Research, Gilead Sciences, Foster City, CA, USA

**Background: **Concomitant use of HCV direct‐acting antivirals (DAAs) in HCV/HIV co‐infected patients may be complicated by pharmacokinetic (PK) drug‐drug interactions (DDIs) with HIV antiretrovirals. Ledipasvir/sofosbuvir (LDV/SOF; Harvoni^®^) and sofosbuvir/velpatasvir/voxilaprevir (SOF/VEL/VOX, Vosevi^®^) are once‐daily fixed‐dose DAA combination regimens approved for 12 or 24 weeks treatment of chronic HCV infection. Bictegravir/emtricitabine/tenofovir alafenamide (B/F/TAF; Biktarvy^®^) is an approved once‐daily single tablet regimen for treatment of HIV that combines the unboosted HIV integrase strand transfer inhibitor bictegravir (BIC; B) and the nucleoside reverse transcriptase inhibitor backbone F/TAF. Phase I studies were conducted in healthy volunteers to assess these DDIs.

**Materials and methods: **The LDV/SOF + B/F/TAF DDI was assessed in a fixed‐sequence, three‐period crossover study. Subjects received B/F/TAF (75/200/25 mg) or LDV/SOF (90/400 mg) alone or together, each for 10 days. The SOF/VEL/VOX + B/F/TAF DDI was assessed in a randomized, six‐sequence, three‐period crossover study. Subjects received B/F/TAF (50/200/25 mg) or SOF/VEL/VOX (400/100/100 mg) + VOX (100 mg) alone or together, each for 10 days. The additional 100 mg of VOX was administered to approximate VOX exposures observed in HCV‐infected patients. Intensive PK samples were collected over 24 hours on the last day of each treatment period and analyzed for BIC, emtricitabine (FTC), TAF, tenofovir (TFV), SOF, GS‐331007 (primary circulating metabolite of SOF), LDV, VEL and VOX plasma concentrations. Geometric least‐squares means (GLSM) ratios and associated 90% CIs (combination vs. alone) for the PK parameters AUCtau, Cmax and Ctau (as applicable) were estimated and compared against lack of PK alteration boundaries of 70% to 143%. Safety assessments were conducted throughout the study.

**Results: **Sixty subjects enrolled and completed the studies (N = 30/study). All adverse events (AEs) were Grade 1 or 2. The only common AE observed was headache (10%, 6/60 subjects). The PK of BIC, FTC, SOF, GS‐331007, LDV, VEL and VOX was unaltered with co‐administration. Modest increases (<2‐fold) in TAF (with SOF/VEL/VOX) and/or TFV exposures (with LDV/SOF and SOF/VEL/VOX) were observed. These increases are not considered clinically relevant and are likely due to increased absorption of TAF resulting from LDV, VEL and VOX inhibition of intestinal P‐gp and/or BCRP transporters, for which TAF is a substrate.

**Conclusions: **Study treatments were generally well tolerated. There were no clinically relevant changes in the PK of any components of B/F/TAF, LDV/SOF or SOF/VEL/VOX. Based on these results B/F/TAF may be co‐administered with SOF, LDV/SOF, SOF/VEL or SOF/VEL/VOX.

**Table:** Abstract P264 – Table 1. PK parameters of HIV ARVs and HCV DAAs when co‐administered

B/F/TAF+LDV/SOF	BIC	FTC	TAF^a^	TFV	SOF	GS‐331007	LDV
AUCtau	↔	↔	↔	67%↑	↔	↔	↔
Cmax	↔	↔	↔	43%↑	↔	↔	↔
Ctau	↔	↔	NR	81%↑	NR	↔	↔
B/F/TAF+SOF/VEL/VOX	BIC	FTC	TAF^a^	TFV	SOF	GS‐331007	VEL	VOX
AUCtau	↔	↔	58%↑	67%↑	↔	↔	↔	↔
Cmax	↔	↔	28%↑	51%↑	↔	↔	↔	↔
Ctau	↔	↔	NR	74%↑	NR	↔	↔	↔

Note: The % change is presented in the table based on calculated %GLSM ratios; 90% CI of the GLSM ratios were within (), extended above (↑) the predefined lack of PK alteration boundaries of 70% to 143%. ^a^AUClast presented for TAF. NR = not reported.

## P265

### Assessment of anti‐epileptic drug concentrations in HIV‐infected patients: a real‐life study

**D Cattaneo^1^, S Baldelli^1^, D Minisci^2^, A Giacomelli^2^, P Meraviglia^2^, N Astuti^2^, M Fusi^1^, V Cozzi^1^, E Clementi^1^, M Galli^2^ and C Gervasoni^2^**

^1^Laboratory Medicine, Luigi Sacco University Hospital, Milano, Italy. ^2^Infectious Diseases, Luigi Sacco University Hospital, Milano, Italy

**Background: **The management of epilepsy in HIV‐infected patients is clinically challenging because of the risk of potential drug‐drug interactions, and further complicated by the lack of data from real‐life settings. This may result in the selection of inadequate anti‐epileptic drug doses, eventually causing suboptimal drug exposure. Here, we aimed to characterise the anti‐epileptic drug concentrations in HIV‐infected patients during routine outpatient visits.

**Materials and methods: **Our clinical databases were investigated in a search for HIV‐infected patients treated with anti‐epileptic drugs for at least 1 month and with at least one assessment of therapeutic drug monitoring (TDM) of trough concentrations available. Such concentrations were categorised as below, within or above each anti‐epileptic target proposed by the AGNP Consensus Guidelines for TDM in Neuropsychopharmacology, update 2017. Main demographic and clinical characteristics of each subject at the time of TDM were also collected.

**Results: **The search identified 260 HIV‐infected patients concomitantly receiving antiretroviral and anti‐epileptic drugs, mainly levetiracetam (44%), valproate (24%), phenobarbital (16%), carbamazepine (7%) and phenytoin (3%). The remaining (6%) were treated with the more recent drugs (topiramate, oxcarbazepine and lamotrigine). Overall, 63% and 37% (31% below and 6% above) of the anti‐epileptic TDM determinations resulted within or out the AGNP therapeutic ranges, respectively (Table 1). Duration of therapy (*p* = 0.008) and type of anti‐epileptic drug resulted significantly associated with the risk of out‐of‐range anti‐epileptic drug concentrations by logistic regression analysis. Indeed, valproate was the anti‐epileptic drug associated with the highest risk of subtherapeutic drug concentrations in HIV‐positive patients (but not in HIV‐negative subjects), with 57%, 0% and 43% of TDM determinations below, above or within the therapeutic range (set at 50 to 100 mg/L). HIV‐infected patients with subtherapeutic valproate concentrations had a trend for lower frequency of boosted‐based (36% vs. 52%) and/or NNRTI‐based (25% vs. 48%) antiretroviral regimens compared with those falling within the AGNP therapeutic windows. Among the clinical covariates, serum creatinine levels and drug daily dose resulted significantly associated with the risk of subtherapeutic valproate concentrations by multivariate logistic regression analysis.

**Table:** **Abstract P265 –** Table 1. Demographic/clinical characteristics of the HIV‐infected patients concomitantly treated with anti‐epileptic drugs categorised according to the therapeutic ranges proposed by the AGNP Consensus Guidelines for TDM in Neuropsychopharmacology, update 2017

Patients’ characteristics	Anti‐epileptic drug concentrations in range	Anti‐epileptic drug concentrations out of range
Age, years	50 ± 8	48 ± 9
Gender, % women	27.6	19.8
Levetiracetam, n (%)	73 (64)	41 (36)
Valproate, n (%)	27 (43)	36 (57)
Phenobarbital, n (%)	36 (88)	5 (12)
Carbamazepine, n (%)	16 (94)	1 (6)
Days of anti‐epileptic therapy	1988 ± 2064	1195 ± 1668
Booster‐based antiretroviral therapy, %^a^	39	38
NNRTI‐based antiretroviral therapy, %^a^	21	19
Tenofovir‐based antiretroviral therapy, %^a^	36	40
INI‐based antiretroviral therapy, %	54	52
Body weight, kg	68 ± 11	74 ± 16
Serum creatinine, mg/dL	0.9 ± 0.2	0.8 ± 0.3
GGT, IU/L	69 ± 62	63 ± 50
ALT, IU/L	35 ± 51	48 ± 82
HCV/HBV co‐infection, %	39	28
CD4 cell count, cells/mm^3^	626 ± 364	577 ± 381
Viral load >37 copies/mL, %	10	20
No. of comedications	2.3 ± 2.0	2.1 ± 2.0

^a^the sum is >100% because patients received more than one antiretroviral. INI = dolutegravir or raltegravir.

**Conclusions: **Our study showed that, in real‐life setting, most of HIV‐infected patients had anti‐epileptic drug concentrations falling within the AGNP therapeutic targets. One important exception is represented by valproate, which was associated with a higher rate of subtherapeutic concentrations compared with other anti‐epileptic drugs. We hypothesise that the fear for potential drug‐drug interactions with some antiretrovirals might have resulted in the selection of inappropriate valproate doses.

## P266

### Pharmacokinetic analysis for darunavir in HIV‐1 infected patients on the cobicistat‐boosted darunavir regimen in an Italian observational, multicentre, prospective study (the TMC114FD1HTX4003 ‐ ST.O.RE. study)

**E Focà^1^, A Antinori^2^, D Ripamonti^3^, R Maserati^4^, S Rusconi^5^, G Rizzardini^6^, M Palma^7^, D Mancusi^7^, R Termini^7^ and A Uglietti^7^**

^1^Tropical and Infectious Diseases, Spedali Civili, Brescia, Italy. ^2^HIV/AIDS Department, National Institute for Infectious Diseases Lazzaro Spallanzani IRCCS, Rome, Italy. ^3^Division of Infectious Diseases, ASST Papa Giovanni XXIII, Bergamo, Italy. ^4^Department of Infectious Diseases, Foundation IRCCS San Matteo Hospital, Pavia, Italy. ^5^Infectious Diseases Unit, DIBIC Luigi Sacco, Milan, Italy. ^6^Division of Infectious Diseases, Luigi Sacco Hospital, Milan, Italy. ^7^Medical Affairs, Janssen‐Cilag SpA, Cologno Monzese, Italy

**Background: **The ST.O.RE. study is an observational, single arm, multicentre, prospective study aimed at collecting real‐life data regarding the effectiveness and safety of an ARV regimen based on cobicistat‐boosted darunavir (DRV/c) in HIV1‐positive patients. A secondary objective of the study was to describe steady‐state DRV Ctrough during observational period.

**Materials and methods: **Darunavir Ctrough values were recorded in the electronic case report form (eCRF) by clinical centres that collect blood samples to perform Ctrough analysis in their routine practice. In 14 patients we obtained two repeated DRV Ctrough sampling. All patients included in this analysis were virologically suppressed when switched from a ritonavir‐boosted protease inhibitor to the DRV/c‐based regimen and at the time of sampling. Comparisons between groups were performed using Mann‐Whitney test.

**Results: **We collected the DRV Ctrough values from blood samples of 56 patients. All of them were Caucasian; 39 were male; the mean (±SD) age was 47.6 (±9.5) years. Median (IQR) DRV Ctrough values were 2862.5 (2969.5) overall, 2634 (2322) ng/mL in males and 4221 (2881) ng/mL in females. Female DRV Ctrough values were statistically higher (*p* = 0.046). Fourteen patients had two repeated DRV Ctrough sampling. Patients characteristics are reported in Table 1. Data of Ctrough are shown in Figure 1. In first sampling all patients were virologically suppressed while in the second one, one patient had HIV‐RNA value of 81 copies/mL with Ctrough 1148 ng/mL. Among the 14 patients, six males and three females were taking concomitant medications (vitamin D, phosphate and antihypertensives). None of the recorded concomitant drugs is known to alter the pharmacokinetics of DRV/c so far. Considering 70 samplings (56 single and 14 repeated) no patients had values below the 55 ng/mL, the protein‐binding adjusted EC50 for wild‐type HIV; six reported values below the threshold of 550 ng/mL, the protein‐binding adjusted EC50 for PI‐resistant HIV: five males (100, 159, 296, 363 and 494 ng/mL) and one female (435 ng/mL). Only half of these 70 patients had a double sampling greater than 550 ng/mL. None showed adverse events at the time of samplings.

**Table:** Abstract P266 – Table 1. Patient characteristics with repeated DRV Ctrough

	Overall (N = 14)	Male (N = 11)	Female (N = 3)
Age (years), mean (SD)	47.3 (8.3)	46.0 (9)	52.0 (2.6)
BMI (kg/mg), mean (SD)	23.4 (7.32)	23.6 (3.5)	22.7 (6.2)
Race (White), N (%)	14 (100)	11 (100)	3 (100)
CD4 nadir (cell/mm^3^), mean (SD)	269.2 (231.4)	254.3 (262.8)	319.0 (75.9)
CDC category C, N (%)	4 (28.6)	4 (36.4)	0
HCV positive, N (%)	4 (27.3)	3 (27.3)	1 (33.3)
Ongoing and treated conditions, N (%)	10 (71.4)	7 (63.6)	3 (100)
% of TDF/FTC as backbone therapy	57.2	54.5	66.6
% of ABC/3TC as backbone therapy	21.4	100	0
% dual therapies	21.4	18.2	33.3

**Abstract P266 – Figure 1.** DRV Ctrough values: repeated samplings.

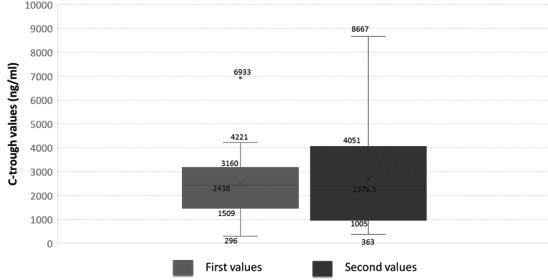

**Conclusions: **In clinical practice we observed a DRV threshold being effective in all patients as cobicistat allowed to obtain Ctrough values far above the protein‐binding adjusted EC50 for wild‐type HIV. The observed intra‐and interpatient variability can be due to the observational nature of this study where samplings might not have been collected at the real Ctrough.

## P267

### Association of tenofovir level and discontinuation due to impaired renal function

**H Yagura^1^, D Watanabe^2^, T Nakauchi^1^, K Tomishima^1^, Y Nishida^2^, M Yoshino^3^, K Yamazaki^1^, T Uehira^2^ and T Shirasaka^2^**

^1^Pharmacy, Osaka National Hospital, Osaka, Japan. ^2^AIDS Medical Center, Osaka National Hospital, Osaka, Japan. ^3^Pharmacy, Utano Hospital, Kyoto, Japan

**Background: **Tenofovir (TFV) preparations are nucleotide reverse transcriptase inhibitors with highly antiretroviral activity used as first‐line treatment in HIV‐1‐infected patients. Long term TFV treatment, however, is associated with a risk of renal impairment, especially cellular damage in renal tubules. Plasma trough concentrations of TFV are high in patients with renal impairment, but the mechanism by which high trough concentration of TFV following its long‐term administration affects renal function has not been clarified.

**Methods: **A regimen including TFV disoproxil fumarate (TDF) was administered to 136 HIV‐1‐infected patients aged ≥18 years treated at the National Hospital Organization Osaka National Hospital between January 2007 and December 2011. Plasma trough concentrations of TFV were measured 20–28 hours after its administration within 3 months after starting treatment. The association between trough concentrations of TFV and discontinuation of its administration due to renal function‐related adverse events within 288 weeks after the start of therapy was measured.

**Results: **The median age of the 136 patients (135 were male) was 39 years (range 18 to 68 years). TDF was discontinued due to renal function‐related adverse events in 34 patients (25%), and the median time to discontinuation was 967 days (range 183 to 1986 days). The median trough TFV concentration was significantly higher in discontinued than in continued patients (87.5 vs. 73.0 ng/mL; *p* = 0.0033). Construction of a receiver operating characteristic (ROC) curve showed that the cut‐off value of trough TFV concentration at TDF discontinuation was 98 ng/mL (area under the curve [AUC], 0.668; sensitivity, 0.471; specificity, 0.833). Trough TFV concentration was significantly correlated with TDF discontinuation due to renal function‐related adverse events (*p* = 0.0022). However, trough concentrations of TFV did not correlate with time to TDF discontinuation (*p* = 0.29).

**Conclusions: **Discontinuation of long‐term TDF administration due to renal function‐related adverse events is associated with high trough TFV concentrations in Japanese patients infected with HIV‐1. This implied the importance of early after administration TFV concentration measurement for the risk assessment of renal dysfunction in long‐term administration.

## COMMUNITY INITIATIVES

## P268

### Effectiveness of a pilot partner notification programme (PNP) for new HIV cases (NHIVC) in Málaga, Spain

**C Gómez‐Ayerbe^1^, C González‐Doménech^2^, I Viciana^3^, M Villalobos^1^, J Santos^1^ and R Palacios^1^**

^1^Infectious Diseases, Hospital Clínico Virgen de la Victoria, Málaga, Spain. ^2^Microbiología, Universidad de Granada/Red Investigación en SIDA, Granada, Spain. ^3^Microbiology, Hospital Clínico Virgen de la Victoria, Málaga, Spain

**Background: **HIV epidemic is not stabilised and almost half of Spanish NHIVC are still late diagnosis (<350 cells/mm^3^) [1]. The partner notification represents an excellent strategy to control it [2], enabling the detection of new cases and decreasing the hidden infections rate. Our objective is to determine the acceptability and effectiveness of a proactive PNP in NHIVC.

**Methods: **A longitudinal prospective pilot study, including NHIVC that attended our centre between October 2017 and May 2018, was performed. Notification to partners was made through the index case (IC) or by the physician (Ph). Acceptability was estimated as the number of IC accepting to participate and the effectiveness as the number of NHIVC detected from partners tested. Demographic and clinical data, as well as sexual behaviour in the last 12 months, were collected in both IC and their partners. A rapid HIV test and screening of other sexually transmitted infections (STIs) (syphilis, chlamydia, gonococcus, HCV, HAV, HBV) were done in all cases.

**Results: **Forty‐eight out of 59 NHIVC were offered to participate in PNP, accepting all of them; 87.5% were MSM, with a mean age of 35.3 (±9.6) years, 68.8% Spanish and 85.5% with secondary or university studies. Stable partner was referred by 18 (37.5%), 35 (72.9%) confirmed condom use always or almost always, 13 (27.1%) practiced chemsex and 20 (41.6%) reported other prior STIs. Thrty‐six (75%) had previous HIV‐negative serology, with a mean seroconversion time of 30.3 months; at the moment of HIV diagnosis, the median CD4 T‐lymphocytes count was 428 cells/mm^3^ (IQR 245 to 529), VL 4.68 log10 (IQR 4.37 to 5.30) and 20 (41.7%) presented another STI (45% asymptomatic). From 13 traceable contacts, 55 were located (33 by IC and 22 by Ph); 18 were already HIV‐positive patients and 20 (HIV unknown or negative serology) came to our unit to be evaluated, two NHIVC were diagnosed. The effectiveness was therefore 10%.

**Conclusions: **The acceptability of partner notification in our cohort was very high, but the effectiveness moderated. Almost half of the NHIVC included had previous STIs, although 25% had never been tested for HIV. The PNP expands the screening coverage, reaching a larger high‐risk population.

**References**

[1] Vigilancia Epidemiológica del VIH y SIDA en España. Actualización 30 Junio 2017. Plan Nacional sobre el SIDA ‐ S.G. de Promoción de la Salud y Epidemiología/ Centro Nacional de Epidemiología ISCIII. Madrid; Noviembre 2017.

[2] Montaner JS. Treatment as prevention: towards an AIDS‐free generation. Top Antivir Med. 2013;21:110‐4.

## P270

### Stakeholders’ experiences of HIV patient engagement within the I‐Score patient‐reported outcome study: benefits and challenges

**D Lessard^1^, K Engler^1^, S Vicente^2^, I Toupin^1^, J Cox^3^, N Kronfli^3^, J Routy^3^ and B Lebouché^4^**

^1^Centre for Outcomes Research and Evaluation, McGill University Health Centre, Montreal, Canada. ^2^Department of Mathematics and Statistics, University of Montreal, Montreal, Canada. ^3^Chronic Viral Illness Service, McGill University Health Centre, Montreal, Canada. ^4^Department of Family Medicine, McGill University, Montreal, Canada

**Background: **Patient engagement (PE) in research is recognized as a valuable approach to improve the quality, applicability and relevance of health research; it ultimately leads to accountable and accessible healthcare. PE implies, in part, partnerships and mentorship between multidisciplinary investigators and patients, recognizing the importance and complementarity of their respective perspectives. However, how different stakeholders experience these partnerships and how these experiences evolve is rarely documented. Our aim is to address this gap. PE is currently a strong facet of the I‐Score patient‐reported outcome (PRO) study. This study is aimed at developing a digital HIV‐specific PRO measure of antiretroviral therapy adherence barriers. Patients are involved in the study's activities through the I‐Score Consulting Team, a Montreal‐based (Canada) consulting committee formed in November 2015 and composed of a diverse group of 10 patients living with HIV. A PE agent‐investigator organizes and co‐leads committee meetings. The Team was originally established to provide regular feedback on the conduct of the I‐Score study and to collaborate in knowledge transfer activities.

**Materials and methods: **We present the experiences of PE of three distinct types of stakeholders who participated in the I‐Score study: (1) clinical investigators, (2) a patient‐investigator and (3) a PE agent‐investigator. Using a reflexive and deliberative exercise, stakeholders identified the challenges and benefits they encountered while implementing and pursuing PE in this context.

**Results: **(1) I‐Score *clinical investigators* experienced challenges of coordinating early PE with the study but found receiving regular patient input reassuring. Integrating stakeholder engagement as a new professional standard, clinical investigators saw unexpected research program expansion yet negative effects on study timelines. (2) The *patient‐investigator* described initial passivity during early PE due to the burden of accessing HIV care and treatment, followed by increasing confidence and involvement in PE. The latter contributed to redefining their HIV diagnosis as an opportunity to viably combine patient and academic expertise as an investigator. (3) The *PE agent‐investigator* faced insecurities associated with initiating PE in a clinical research setting as well as difficulties accessing PE mentorship and finding methods to evaluate and report on PE. They also discovered unanticipated professional and academic opportunities and demand for applying PE beyond the I‐Score study.

**Conclusions: **Through HIV PE, stakeholders affiliated with the I‐Score PRO study faced unanticipated personal and professional impacts, illustrating PE's potential to challenge and change existing research practices and experiences of living with HIV.

## P271

### HIV, attitudes, knowledge and practices associated with Chemsex: a first study of the medical team in Argentina

**S Unda, C Strasorier and E Villegas**

Health and Science, National University of La Rioja, La Rioja, Argentina

**Background: **Argentina maintains the highest rates of synthetic drugs use in the region while the number of HIV patients continues to increase. The sexualised drug use has become a new global phenomenon to facilitate sex (also known as Chemsex). At present, it has not been studied yet in the country, which justifies the importance of determine the role of the medical team in the HIV transmission chain. The aim of this work is to determine, through self‐prepared scales, the relationship between knowledge and attitudes about HIV and the behaviours associated with Chemsex in a sample of medical students.

**Methods: **The sample n = 248 students was calculated with simple random method, a confidence interval of 95%. Research instrument: anonymous, closed‐type, self‐filled survey consisting of two parts: HIV Knowledge and Attitudes Scale and Prevailing Risk Factors and Practices Associated with Chemsex Scale. Results are represented by different groups (homosexuals, heterosexuals, males and females). The statistical analysis of the validation and the results was carried out with SPSS Statistics v.23, post hoc Duncan, Z test with Bonferroni´s multiples comparisons and Pearson test were applied considering significant differences of *p* < 0.05.

**Results: **In the scales validation an α‐Cronbach of 0.87 was obtained. The correlation with the Pearson test was: Knowledge/Attitude = 0.085, Knowledge/Chemsex = ‐0.033. In the assessed factors, the knowledge and attitude scores were no statistically different between each group. The Chemsex score is significantly highest (*p* < 0.05) in homosexual and male groups. The sexually transmitted infections (STIs) are more prevalent in homosexual (29%) and female (21%) groups. The homosexual (82%) and male (51%) groups are the ones who had tested for HIV the most.

**Conclusions: **In this first study of Chemsex in Argentina, from our sample of medicine students we obtained that knowledge is not related to better attitudes towards HIV patients, neither to practices associated to Chemsex. Moreover, we found that there is a high prevalence of risk behaviours and practices associated to Chemsex, STIs and poor control of HIV. It is possible that the increasing number of HIV patients has a strong link to the magnitude of drug use. With no doubts the health system needs to focus in the medical team who plays a key role in the HIV transmission chain, so they can awareness community in a consciously way.

## P272

### Improvement of intrinsic capacity in older adults living with HIV through a health promotion resource

**A Caselgrandi^1^, A Malagoli^1^, J Milic^1^, E Spencer^2^, B Gallagher^2^, G Lui^3^, C Cheung^3^, M Mancini^1^, V Masi^1^, E Bardi^1^, M Corni^1^, M Menozzi^1^, S Zona^1^, F Carli^1^, C Mussini^1^ and G Guaraldi^1^**

^1^Modena HIV Metabolic Clinic, University of Modena and Reggio Emilia, Modena, Italy. ^2^Department of Clinical Research, Holdsworth House Medical Practice, Sydney, Australia. ^3^Faculty of Medicine, The Chinese University of Hong Kong, Hong Kong

**Background: **My Smart Age with HIV (MySAwH) is a multi‐centre prospective ongoing study designed to empower older adults living with HIV (OALWH) in achieving healthier lifestyles. It is based on collection of physical function data and patient‐related outcomes through dedicated smart phone app. The aim of MySAwH is to detect health changes assessed with frailty index (FI), generated by health professionals, and with health index (HI) evaluating patient's intrinsic capacity (IC), generated by themselves. IC consists of five domains, comprising all the physical and mental capacities that an individual can draw in old age. It is built on residual health and patient empowerment. The aim of this study is to describe variations of FI and HI across time, after 9 months of follow‐up.

**Material and methods**

This study includes OALWH aged >50 years undergoing stable ART from Italy, Australia and Hong Kong recruitment sites who completed 9 months of follow‐up. FI includes 37‐item health variables, each of them is scored from 0 to 1. FI >0.3 was used to identify most frail individuals. HI includes 12 variables from five IC domains: Locomotion, Cognition, Vitality, Sensory, Psychosocial. After obtaining HI scores, IC is calculated with a 0 to 1 range score; the lower is the score, the better is IC evaluation. Variables were collected through a fitness tracking wearable device (Garmin‐Vivofit 2) and through questionnaires provided via ecological momentary assessment (EMA) [1] using MySAwH App. HI was collected on a monthly basis, while the FI was assessed on the baseline and follow‐up visit. Statistical analyses were performed in R software.

**Results: **One hundred and fifty‐three OALWH are included in this analysis. Median age is 57 years. Twenty‐two (14.38%) patients are women. Mean CD4 is 686.99 (324.14 SD) and 141 (92.76%) patients had undetectable HIV viral load. Median FI at baseline is 0.23 (0.2–0.32 IC) and at nine months follow‐up is 0.26 (0.2 to 0.31 IC) with no significant p value 0.37. Median IC at baseline is 0.33 (0.25 to 0.4 IC) and at nine months follow‐up is 0.3 (0.2 to 0.36) with significant *p* value 0.05. IC domains prevalence was calculated every month, but no domain shows a significant change after nine months.

**Conclusion: **This study shows a continuous improvement in IC after nine months of follow‐up, but not decrease of FI. The presence of a health coach that provides information about HI variations and lifestyle promotion can stimulate patients to be personally empowered to change their health measured by IC.

**Reference: **[1] ClinicalTrials.gov. My Smart Age With HIV: Smartphone Self‐assessment of Frailty (MySAwH) [Internet]. Available from: http://https://clinicaltrials.gov/ct2/show/NCT02663856.

## P273

### Surveying Ontario nurses using the COM‐B framework shows a high level of readiness for nurse‐led PEP and PrEP

**M Clifford‐Rashotte^1^, J Lee^2^, N Fawcett^3^, B Fowler^4^, J Reinhart^5^ and D Tan^2^**

^1^Medicine, University of Toronto, Toronto, Canada^2^Division of Infectious Diseases, St. Michael's Hospital, Toronto, Canada^3^Sexual Health Clinics, Toronto Public Health, Toronto, Canada^4^Health Sexuality Program, Region of Peel Health Department, Peel, Canada. ^5^Sherbourne Health Centre, Toronto, Canada

**Background: **Effective HIV prevention in Ontario requires more widespread implementation of post‐ and pre‐exposure prophylaxis (PEP and PrEP), including through nurse‐led models of care. To plan for further scale up of nurse‐led PEP and PrEP, we assessed nurses’ readiness to deliver these interventions, using a behavioral change framework.

**Materials and methods: **We asked the managers of every sexual health clinic, HIV clinic and community health center in Ontario to distribute an online survey to nurses in their organizations between March and June 2018. Our primary objective was to determine the proportion of nurses who would support the implementation of nurse‐led PEP and PrEP in their workplace. We explored nurses’ readiness for these interventions using the COM‐B behavioral change framework [1], assessing “capabilities,” “opportunities” and “motivations” for providing PEP and PrEP. Data were analyzed using descriptive statistics, and a multivariable logistic regression model was constructed to identify variables associated with support for nurse‐led PEP and PrEP.

**Results: **Of 470 surveys distributed, 165 had responses for the primary outcome and were included in the analysis. Respondents had a median of 16 (25th percentile=9, 75th percentile=25) years of nursing experience and most worked in sexual health clinics (65.5%). The largest proportion of respondents was from Central Ontario (29.5%), though all regions were represented. 72.7% of respondents supported implementation of nurse‐led PEP and PrEP. More experienced nurses were less likely to support nurse‐led PEP and PrEP (aOR 0.55 per decade nursing, 95% CI 0.37 to 0.82). Nurses’ self‐reported knowledge of topics related to PEP/PrEP and comfort performing relevant clinical tasks were high, with the exception of creatinine interpretation, which only 30.8% of respondents felt comfortable with. Most respondents had initiated conversations with patients about PEP (70.3%) or PrEP (62.2%), but few worked in institutions which provide them (22.4% and 13.3%, respectively). The most commonly cited barriers to implementation were a lack of physician support (38.8% for PEP, 42.9% for PrEP), followed by a lack of knowledge among nurses (38.8% for PEP, 37.4% for PrEP). Finally, the most popular modalities for receiving potential further education about PEP and PrEP were online modules (86.5%), followed by in‐person workshops (71.2%).

**Conclusions: **Nurses at Ontario sexual health clinics, HIV clinics and community health centers exhibit a high level of support for nurse‐led PEP and PrEP, and are well positioned to provide these interventions. To increase their implementation, priorities should include increasing physician support and providing online and in‐person education for nurses, with an emphasis on renal monitoring.

**Reference: **[1] Michie S, van Stralen MM, West R. The behaviour change wheel: a new method for characterising and designing behaviour change interventions. Implement Sci. 2011;6:42.

## P274

### Abstract withdrawn

## MODELS OF CARE: COST EFFECTIVENESS

## P275

### Exploring the correlation between price and affordability across 50 countries: is pricing of dolutegravir equitable?

**J Sim^1^ and A Hill^2^**

^1^School of Public Health, Imperial College London, London, UK. ^2^Department of Translational Medicine, University of Liverpool, Liverpool, UK

**Background: **In the SINGLE trial, dolutegravir (DTG) showed fewer adverse events than efavirenz (EFV) as first‐line treatment, but no difference in virological suppression, quality of life or survival [1]. In switching studies (NEAT 022, SWORD, STRIIVING), DTG led to significantly higher rates of adverse events and no virological benefit [1]. Yet, in upper‐income countries such as UK, DTG currently costs £6068 per year compared to £108 for EFV. There are other low‐cost generic antiretrovirals which could be used as alternatives to DTG.

**Method: **Lowest prices of DTG and EFV in over 50 countries were collected from national price databases, reimbursement authorities and WHO Global Price Reporting Mechanism database. Median prices calculated for each income level group. We analysed the correlation between (1) DTG prices or (2) ratio of DTG to EFV (DTG/EFV) and gross domestic product (GDP) per capita (2016) published by the World Bank. Prices were recorded in US$ per person‐year (PPY).

**Results: **Annual prices of DTG ranged from $27 PPY in Georgia to $20,130 in the USA (Table 1). DTG prices in high‐income countries (HICs) and upper middle‐income countries (UMICs) excluded from VL arrangements are substantially higher compared to low‐ and lower middle‐income countries (LMICs): median price in HICs $9045 PPY versus $2682 (UMICs) and $60 (LMICs). The price in USA ($20,130) is almost twice of Denmark ($11,056), although their GDP per capita is comparable (USA: $57,638 vs. Denmark: $53,579). In UMICs, we did not observe a correlation between DTG price and GDP per capita: in Bulgaria, DTG costs $9656 PPY compared to $2682 in Argentina, even though her GDP/capita is much lower (GDP/capita for Bulgaria: $7469 vs. Argentina: $12,440). There was also no relationship between DTG/EFV price ratio and GDP per capita. However, DTG prices were found to be much higher than EFV in UMICs (median DTG/EFV ratio 6.9) compared HICs (ratio 4.9). In LMICs, DTG prices were close to EFV prices (ratio 1.8).

**Table:** Abstract P275 – Table 1. Highest and lowest prices of dolutegravir, by income groups

Income group	Country	Price of DTG per person‐year (US$)	GDP per capita in 2016 (US$)
High‐income countries	Highest price: USA	$20,130	$57,638
High‐income countries	Lowest price: Canada	$5267	$42,349
Upper middle‐income countries	Highest price: Bulgaria	$9656	$7469
Upper middle‐income countries	Lowest price: Iran	$36	$5219
Low and lower middle‐income countries	Highest price: India	$538	$1710
Low and lower middle‐income countries	Lowest price: Georgia	$27	$3866

**Conclusions: **Prices of DTG vary widely between countries, from <$60 to over $20,000 per person‐year. Overall, we did not observe any correlation between DTG price and GDP per capita within income groups. In HICs and UMICs, the price of DTG is 4.9 to 6.9 times higher than EFV, despite limited clinical benefits. The cost‐effectiveness of DTG versus EFV should be re‐evaluated now that low‐cost generic EFV has become widely available.

**Reference: **[1] Hill A, Mitchell N, Hughes S, Liew Z, Pozniak A. Meta‐analysis of dolutegravir for 7340 patients in 13 trials: effects of current HIV RNA suppression on efficacy and safety [poster abstract P16]. HIV Med. 2018;19 Suppl 2:s26.

## P276

### Improving attendance at HIV clinic: a quality improvement project using a text message reminder service and analysis of a demographic database to tailor interventions

**A Holt, J Van Aarsten, M Chaponda and H Winslow**

Infectious Disease, Royal Liverpool University Hospital, Liverpool, UK

**Background: **Nine percent of all hospital clinic appointments in the UK are missed at an estimated cost of 225 million pounds annually [1]. Data from the Royal Liverpool University Hospital (RLUH) shows significantly higher rates of 10 to 26% across different specialties, with HIV clinics amongst the worst attended. There is good evidence linking clinic attendance and outcomes for HIV patients [2]. The cornerstones of successful HIV care, medication supply and blood monitoring, are primarily provided through clinic attendance at the RLUH so supporting attendance is likely to improve individual care and reduce wasted clinic time.

**Materials and methods: **Attendance data for a single HIV clinic at RLUH were collected from April 2016 onwards, initially through paper returns and subsequently from the computerised appointment system. After establishing a baseline attendance rate, a series of interventions were tried, the first being an ‘opt out’ text message reminder service. Impact on attendance was shared with the whole clinical group to plan further interventions. Using the clinic database, demographic data were collected for all service users who did not attend (DNA) an appointment between the months of August 2017 and November 2017. These data included age, CD4 count, viral load, active psychiatry input, medication regimes and history of illicit drug use.

**Results: **On average 92 patients were seen each month. There were big variations in DNA rates from week to week 0% to 46%. Before any intervention, the median DNA rates were 26%; this reduced to 19% after text messages and 17% after other interventions. Demographic data identified two groups of non‐attendees: (1) Those who repeatedly DNA, missing two or more appointments over 4 months. These are vulnerable patients who generally are not virally suppressed and are likely to have active psychiatric problems; (2) A larger group who each missed a single appointment. On the whole, these patients remained virally suppressed and have similar characteristics to the clinic cohort as a whole.

**Conclusions: **(1) There has been a significant and sustained improvement in DNA rates from 26% to 17%, an absolute reduction of 35%. This has prevented an average of nine appointments per month from being wasted in a single clinic. The project has now been rolled out to include all HIV clinics at RLUH. (2) Further interventions including tailoring the current service are being planned to help the separate subgroups identified engage better with effective care. (3) Significant further improvement is needed and likely to be possible through quality improvement methodology.

**Abstract P276 – Figure 1.** Run chart showing percentage DNA rate at HIV clinic.

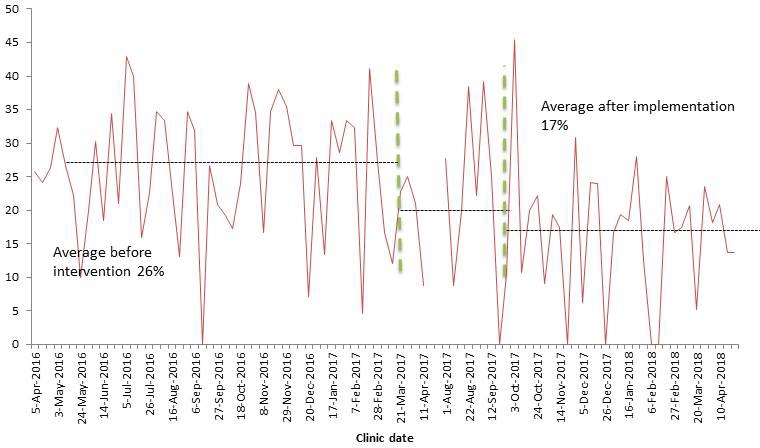

**References**

[1] Department Of Health And Social Care. A zero cost way to reduce missed hospital appointments [Internet]. [cited 2018 May 28]. Available from: http://https://www.gov.uk/government/publications/reducing‐missed‐hospital‐appointments‐using‐text‐messages/a‐zero‐cost‐way‐to‐reduce‐missed‐hospital‐appointment.

[2] Berg MB, Safren SA, Mimiaga MJ, Grasso C, Boswell S, Mayer KH. Non‐adherence to medical appointments is associated with increased plasma HIV RNA and decreased CD4 cell counts in a community‐based HIV primary care clinic. AIDS Care. 2005;17:902‐7.

## P277

### Abstract withdrawn

## P278

### Romania in HIV/AIDS numbers 1985 to 2017: cascade of care in HIV/AIDS infection

**M Mardarescu^1^, A Streinu‐Cercel^1^, S Petrea^2^, M Iancu^2^, D Vitelaru^2^, S Vintila^2^, D Otelea^3^, C Schiopu^2^ and A Mardarescu^2^**

^1^Immunodepression Department for Children & Adolescents, National Institute for Infectious Diseases Prof. Dr. Matei Bals, Bucharest, Romania. ^2^Compartment for M & E of HIV in Romania, National Institute for Infectious Diseases Prof. Dr. Matei Bals, Bucharest, Romania. ^3^Molecular Genetics Laboratory, National Institute for Infectious Diseases Prof. Dr. Matei Bals, Bucharest, Romania

**Background: **ECDC's annual report on the status of HIV epidemic in Europe, released in November 2017, states that during 2006 to 2016 the trend of diagnoses was stable, namely 6.8 and 6.9/100,000 in the earlier period of surveillance to 5.9/100,000 in 2016 [1]. In this context, besides interventions and financial investment in the National HIV/AIDS Programme, Romania has also adapted and implemented ECDC's monitoring tool: cascade of care.

**Objectives: **The objective was to identify the inconsistencies between the numbers of diagnosed and undiagnosed persons, access to public medical services, success and gaps in the specific national interventions using the continuum of care monitoring tool.

**Material and methods**

Each stage of the continuum was adapted and applied to the statistical data centralised annually in the National HIV/AIDS Database. Given our country's specific epidemic, the essential factors that help perform a correct evaluation of the HIV cascade of care in Romania are: the Romanian cohort infected in the late 1980s and early 1990s, with multiple ART schemes (33% more than three therapeutic schemes) and therapeutic fatigue; new HIV cases detected in young people in their fertile age with low CD4 count at the time of diagnoses (<350 cells/mm^3^) and who represent approximately 50% of the total number of cases; men who have sex with men (20.08% of the new cases in 2017), injecting drug users (13.18% from the total new cases diagnosed in 2017) and young mothers [2].

**Results: **Thus the numbers reflected by the continuum stages in Romania are: 15,009 people living with HIV/AIDS (83% from UNAIDS estimates of 16,000 to 18,000) registered in the national HIV/AIDS Database, of these 12,806 (85%) persons are in active records, of these 11,570 (92%) are under ART and finally from the latter 7386 (68%) are virally suppressed (<50 copies/mL) [3,4]. From the overall 15,009 patients in life, 5500 (37%) come from the Romanian cohort, non‐vertically infected.

**Abstract P278 – Figure 1.** Current HIV treatment cascade in Romania. Cascade of Care ‐ 31 December 2017.

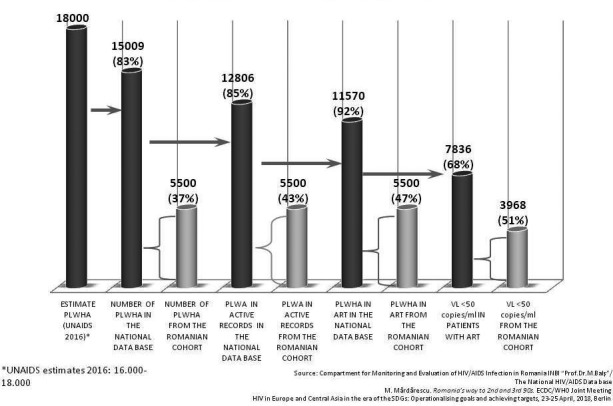

**Conclusions: **Given the desirable percentage of 74% virally suppressed of all people living with HIV [1], Romania's interventions should be adapted to current epidemic status focusing on: the correct management of long‐term survivors, pre‐exposure prophylaxis as means to prevent HIV transmission (which has the potential to reduce the risk of HIV to seronegative partners), pregnant women and perinatally children exposed, optimal therapeutic regimens for children, correct assessment of drug‐drug interactions, multidisciplinary teams and maybe the most important one: universal HIV testing for all vulnerable groups.

**References**

[1] European Centre for Disease Prevention and Control. Continuum of HIV care: monitoring implementation of the Dublin Declaration on Partnership to Fight HIV/AIDS in Europe and Central Asia: 2017 progress report [Internet]. 2017 [cited 2018 Jul 5]. Available from: http://https://ecdc.europa.eu/sites/portal/files/documents/Continuum‐of‐HIV‐care‐2017.pdf.

[2] European Centre for Disease Prevention and Control / World Health Organization. HIV/AIDS surveillance in Europe 2017: 2016 data [Internet]. 2017 [cited 2018 Jul 5]. Available from: http://https://ecdc.europa.eu/sites/portal/files/documents/20171127‐Annual_HIV_Report_Cover%2BInner.pdf.

[3] Compartment for Monitoring and Evaluation of HIV/AIDS Data in Romania. HIV/AIDS infection in Romania: update [Internet]. 2017 [cited 2018 Jul 5]. Available from: http://www.cnlas.ro/images/doc/01122017_rom.pdf.

[4] Mărdărescu M. Romania's way to 2nd and 3rd 90s. ECDC/WHO Joint Meeting: HIV in Europe and Central Asia in the Era of the SDGs: Operationalizing Goals and Achieving Targets; 2018 Apr 23‐25; Berlin, Germany.

## P279

### The HIV MDT: safe and cost effective?

**N Scott, A Torkington, B Dancso and L Johnson**

Infectious Diseases, North Manchester General Hospital, Manchester, UK

**Background: **Managing HIV‐infected individuals with ARVs can present complex challenges. The HIV multi‐disciplinary team (MDT) meeting provides a forum for discussion and an approval process for all decisions related to HIV treatment. This has been a commissioner's requirement since 2015 [1]. An early MDT review [2] demonstrated that the process was cost neutral. In the context of evolving prescribing practice we have analysed the current cost impact of the MDT and the health‐related outcomes of the approved treatments.

**Methods: **Demographic and pharmacy data were retrospectively reviewed via electronic systems and MDT records. Statistical analysis was performed.

**Results: **Records relating to 60 patients reviewed via MDT between January and March 2017 have been analysed to date. Eight patients were excluded (four treatment re‐starts post‐disengagement; four where no treatment change was made), resulting in analysis of 52 cases. Demographics: 66% male; mean age 46 years; 60% Caucasian, 27% Black African; 37% heterosexual, 33% MSM. The reasons for referral included tolerability (48%), adherence (22%), resistance/treatment failure (15%) and drug interactions (3%). Cost analysis demonstrated that HIV MDT decisions resulted in a net saving of £1158 over a 2.5‐month period. A monthly saving was made in 67% of cases (n = 35), with a total saving of £1550 per month achieved. In 17 cases regimen switch resulted in a total monthly cost increase of £1087. Higher cost regimen switches related to resistance/treatment failure (n = 6), co‐infection with hepatitis B (n = 2), increased cardiovascular risk (n = 3) or other side effects (n = 6). For the 15 cases, where treatment switch resulted in over £100 savings per month, the initial backbones were as follows: Truvada (n = 10), complex non‐standard regimens (n = 4) and PI monotherapy (n = 1). These were switched to either Kivexa (n = 6), TAF‐based regimes (n = 5) or different complex non‐standard regimes/PI monotherapy (n = 4). The MDT recommendations were followed in 98% cases. Of the patients with an initial viral load <40 copies/mL, two became detectable post‐switch. Both became undetectable on repeat testing. Thirty‐nine of the 51 cases who switched according to recommendations remain on the same regimen after 12 months. Of these 92% remain virologically suppressed.

**Conclusion: **This study demonstrates that the MDT remains a safe and effective forum for the discussion and approval of ARV switches compared to its introduction in 2015. Cost savings can be made, with necessary increased cost for complex patients being offset by the rationalisation of regimens for less complex individuals.

**References**

[1] British HIV Association. Standards of care for people living with HIV [Internet]. [cited 2018 Jul 6]. Available from: http://www.bhiva.org/documents/Standards‐of‐care/BHIVAStandardsA4.pdf.

[2] Dave F, Sayers I, Niazi F, Torkington A, Wilkins E, Ajdukiewicz K. Cost impact of an HIV MDT for managing anti‐retroviral switch. HIV Med. 2016;17 Suppl 1:12.

## P280

### Stable+: why do HIV clinicians review virally stable patients?

**A Findlay, P Hine and M Chaponda**

Tropical and Infectious Diseases, Royal Liverpool University Hospital, Liverpool, UK

**Background: **In 2016, the British HIV Association reduced the advised frequency of viral load monitoring for people with viral suppression from three to six monthly, to six monthly. This update sought to reduce unnecessary tests when most HIV‐positive patients are fit and well. We aimed to identify the reasons for more frequent clinical review in patients who were stable.

**Materials and methods: ** We identified patients attending HIV follow‐up between January 2017 and June 2018 in a UK hospital via the HIV and AIDS Reporting System (HARS) database. Inclusion criteria were: two or more undetectable viral loads recorded during follow‐up and more than one instance of medical follow‐up occurring at <24 week intervals. We excluded new patients and HARS complex categories.

**Results: ** Of 1223 patients in the cohort, we identified 246 patients (20%) who were established on ART with a consistently suppressed viral load, but reviewed more frequently than necessary for viral load monitoring alone. Of the 246 patients, 171 were male (69%), and median age was 46 years (range 18 to 77). The most common reasons for follow‐up intensity were medically related to HIV: therapy switch (27%) and low CD4. The next most common reasons were management of comorbidity often associated with, but not caused by, HIV. This included investigation of unresolved medical symptoms (15%), or because the patient had intercurrent follow‐up with another medical specialty (9%). Less common reasons related to wider holistic care, such as: concerns for patient's psychological wellbeing (6%); consultation challenges (5%); and concerns about potential adherence lapses (5%).

**Conclusions: ** HIV physicians offer increased follow‐up to virally stable patients for many reasons. Often these are not directly related to their HIV disease. HIV clinicians increasingly recognise this as a “stable+” category. In many cases managing the “plus” ensures the HIV remains stable. In those instances where direct HIV physician input is not required, cost‐effectiveness could be improved through involvement of allied health professionals, and better communication with colleagues in other specialities.

## MODELS OF CARE: EVALUATION OF ARV DELIVERY AND COVERAGE

## P281

### Modeling the future need for adult protease inhibitors (PIs) in generic accessible (GA) low‐ and middle‐income countries (LMICs) in the context of dolutegravir (DTG) roll‐out

**V Prabhu and S McGovern and Z Panos**

HIV Access Program, Clinton Health Access Initiative, Boston, MA, USA

**Background: **DTG‐based therapy is set to transform first‐line (1L) ART in GA LMICs. In particular, DTG's durability in terms of a high genetic barrier to resistance could lead to lower second‐line (2L) migration than in the past. Further, positive early results from the DAWNING study, donor interest and significantly lower costs may lead to DTG quickly replacing PIs in 2L. With these dynamics, it is important to understand what role PIs will have in the coming years.

**Materials and methods: **Starting in 2018, four patient segments who may eventually need PIs were modeled: a) those already on PIs in 2L in 2017; b) those newly failing a NNRTI in 1L; c) those newly failing DTG in 1L; and d) those newly failing DTG in 2L (after 1L NNRTI failure). CHAI's forecast for GA LMICs was used to establish the 1L NNRTI and DTG patient pools between 2018 and 2025. NNRTI patients were assumed to fail their therapy at historical rates. Assumptions were used for the timing and rate of adoption of DTG as the preferred therapy after 1L NNRTI failure, and proactive switching of stable 2L patients from PIs to DTG. Varied assumptions were made on median time to failure on DTG, with a distribution on either side of the median.

**Results: **In the short term, the number of individuals on PI‐based therapies is expected to decrease. The rate and extent of the decline will be dependent on how quickly existing 2L PI patients are proactively switched to DTG and the extent to which 1L NNRTI failures will go onto DTG for 2L treatment rather than using a PI. In the long term, the need for PIs will increase as those individuals failing DTG‐based 1L and 2L regimens will ultimately likely need a PI (Figure 1). The durability of DTG will be fundamental in determining when that eventual rise in PI need will occur (Table 1).

**Abstract P281 – Figure 1.** Illustrative representation of potential need for PIs over time.

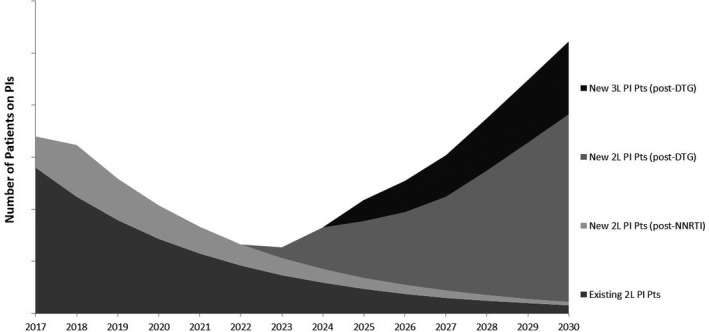

**Table:** **Abstract P281 –** Table 1. Potential scenarios based on 2L DTG adoption and DTG durability

Patients needing PIs (000s)	2018	2019	2020	2021	2022	2023	2024	2025
Scenario 1: 2L DTG adoption in 2020; 6 yr median DTG durability	990	1150	990	580	460	970	2390	4300
Scenario 2: 2L DTG adoption in 2019; 6 yr median DTG durability	990	920	520	250	410	980	2460	4460
Scenario 3: 2L DTG adoption in 2020; 7 yr median DTG durability	990	1150	990	530	240	360	890	2260
Scenario 4: 2L DTG adoption in 2019; 7 yr median DTG durability	990	920	520	200	180	360	910	2330
Scenario 5: 2L DTG adoption in 2020; 8 yr median DTG durability	990	1150	990	530	190	160	310	840
Scenario 6: 2L DTG adoption in 2019; 8 yr median DTG durability	990	920	520	200	130	140	310	860

**Conclusions: **These findings on the likely market dynamics around the demand for PIs, and timing of demand growth in particular, can help inform appropriate strategies for efforts to provide access in LMICs to optimal PI options (such as darunavir) in suitable formulations and at affordable prices.

## P282

### Practices and challenges for an HIV infection model of care: the Australian experience

**D Smith^1^, I Woolley^2^, D Russell^3^, F Bisshop^4^ and V Furner^1^**

^1^Albion Centre, South Eastern Sydney Local Hospital Network, Sydney, Australia. ^2^Monash Infectious Diseases, Monash University, Melbourne, Australia. ^3^College of Medicine and Dentistry, James Cook University, Cairns, Australia. ^4^Holdsworth House Medical Practice, Brisbane, Australia

**Background: **Unlike some other developed countries, PLWHIV in Australia can select how they access care. Patients can elect to be managed by ‘s100 HIV‐therapy prescribing’ general practitioners (s100 GPs), sexual health physicians (SHPs), hospital‐based physicians (HBPs) or a combination of these options. We explored the strengths and weaknesses of the Australian model of care, by investigating the practices and challenges in HIV infection management for each practitioner specialty group.

**Materials and methods: **We surveyed 26 s100 GPs, 24 SHPs and six HBPs currently involved in HIV infection management in Australia. The online quantitative survey was conducted between October and November 2017 by global research organisation, Kantar Health, and results analysed between November 2017 and January 2018. The 47 survey questions sought to identify current practices and challenges for HIV infection management under the Australian model of care, and differences in approaches between practitioner specialties. Survey questions focused on patient profile, testing triggers, treatment selection and initiation, alignment with management guidelines and unmet needs.

**Results: **Survey results suggested that practices and challenges are mostly similar across different practitioner specialties under the Australian model of care. However, treatment choices and approaches to prescribing HIV therapy were identified as key differences: more s100 GPs listed older therapies (such as nevirapine) as their preferred options in contrast with SHPs and HBPs. s100 GPs were also less likely to align prescribing approaches with current treatment recommendations in other ways, with 23% nominating zidovudine and 12% Atripla^®^ as top treatments for newly diagnosed patients with HIV, with no mentions of these therapies from SHPs. In further contrast with SHPs, s100 GPs reported lower overall satisfaction with current HIV treatment guidelines (83% of SHPs very satisfied vs. 46% of s100 GPs), and were less likely to change HIV therapies to simplify treatment regimens (88% of SHPs vs. 58% of s100 GPs, *p* = 0.019). Increasing mental health support also appears to be a critical issue in Australian HIV infection management, noted by 36% of all practitioners as a major unmet need.

**Conclusion: **The Australian model of care provides patients with choice as to their preferred management access point; however, greater support may be needed for some s100 GPs to ensure all patients are receiving treatment consistent with best practice guidelines. Further investigation with a much larger sample size of s100 GPs is warranted. A greater focus on improving mental health services for PLWHIV in Australia is also recommended.

## P283

### Progress with antiretroviral therapy in Russia

**A Pokrovskaya^1^, N Ladnaia^1^, Z Suvorova^1^, O Yurin^1^, L Dementieva^2^, K Emerole^3^ and V Pokrovsky^1^**

^1^Central Research Institute of Epidemiology of Rospotrebnadzor, Russian Federal AIDS Centre, Moscow, Russian Federation. ^2^HIV/AIDS Department, Russian Federal Service for Surveillance on Consumer Rights Protection and Human Wellbeing Rospotrebnadzor, Moscow, Russian Federation. ^3^Department of Infectious Diseases, RUDN University, Moscow, Russian Federation

**Background: **In Russia ART is provided free of charge to PLWHIV, who visit special AIDS centres. According current guidelines ART is eligible for all PLWHIV, but it is a priority for patients with CD4 counts <350 cells/mL. The aim of this study was to characterise the changes in the basic aspects of antiretroviral therapy in Russia.

**Methods: **We analysed the national HIV/AIDS monitoring forms of Rospotrebnadzor and the results of multicentre, open‐label study with the inclusion of a retrospective model “Portrait of the Patient” (7000 adult patients, who visited AIDS centres and signed an informed consent form in 27 regions of Russia in 2014).

**Results: **More than 500,000 new HIV cases were registered in Russia from 2011 to 2016 and reached 870,952 PLWHIV to the end 2016. More than 285,000 PLWHIV were on ART in 2016. Proportion of PLWHIV who received ART from the total diagnosed PLWHIV increased from 18% in 2011 to 33% in 2016. There was satisfactory adherence to ART so viral suppression was not less than 81% in treated patients during 2011–2014 by the results of study “Portrait of the Patient”. And 75% of PLWHIV on ART achieved HIV RNA <1000 copies/mL in 2016. The most commonly prescribed ART combination for naïve patients in 2016 was 2NRTI+NNRTI (74%). Only 4% of naïve patients took 2NRTI+INI. Discontinuation of therapy in 2016 was recorded in 8% of patients. This was due to death of the patient (54%), loss of patient monitoring (26%), refusal of treatment (15%), adverse events (6%). Fourteen percent of all patients on ART changed combination in 2016. Twenty‐four percent of patients who started treatment in 2016 switched ART in the same year. The reasons for ART switched were adverse events (43.3% in 2016, 56% in 2016), simplification (reducing the number of pills and multiplicity; 27% in 2014, 25% in 2016), treatment failure (3% in 2014, 10% in 2016). The most frequent adverse effects of the first ART regimens in study “Portrait of the Patient” were: gastrointestinal disorders (33.7%), CNS disorders (22.3%), anaemia/leukopenia (14.8%), rash/dermatitis/allergic reaction (10.15%).

**Conclusions: **During the six years we noted the positive trends in the involvement of PLWHIV in treatment despite the rapid increase of new HIV cases. ART combinations were effective and tolerable in a part of the patients, but the number of adverse effects were significant. Measures are needed to encourage ART initiation and utilise drugs with lower toxicity.

## P284

### Rapid initiation of ART following diagnosis of HIV among Medicaid beneficiaries: a real‐world evaluation

**C Benson^1^, B Emond^2^, H Romdhani^2^, K Dunn^3^, A Shohoudi^2^, N Tandon^4^ and P Lefebvre^2^**

^1^Infectious Diseases, LLC, Real World Value & Evidence, Janssen Scientific Affairs, Titusville, NJ, USA. ^2^HEOR, Analysis Group, Inc., Montreal, Canada. ^3^Infectious Diseases, Janssen Scientific Affairs, LLC, Boston, MA, USA. ^4^Health Economics and Outcomes Research, Janssen Scientific Affairs, LLC, New York, NY, USA

**Background: **Rapid initiation of ART may benefit patients with HIV and reduce transmissions. This study aims to assess real‐world time to ART initiation and describe treatment outcomes in Medicaid patients based on the timeliness of ART initiation.

**Methods: **A retrospective longitudinal analysis was conducted using multi‐state Medicaid data (January 1997–March 2017). Adults with HIV‐1 and ≥6 months of continuous eligibility pre‐HIV‐1 diagnosis, no ART pre‐HIV‐1 diagnosis, no HIV‐2 diagnosis at any time and initiating an ART (including one protease inhibitor [PI], or one integrase strand transfer inhibitor [INSTI], or one NNRTI, with ≥2 NRTIs within 14 days) in 2012 or later were included. Patients were classified into mutually exclusive groups based on timeliness of receipt of ART after HIV‐1 diagnosis. Patients were characterized based on demographics and clinical characteristics at the time of HIV diagnosis. During post‐diagnosis but prior to receiving ART, incidence of opportunistic infections and healthcare resource use were evaluated.

**Results: **Among eligible patients (n = 1022), mean age was 40.0 (SD 11.9, median 41, range 18.0 to 70.0) years, 451 (44.1%) were females and 582 (56.9%) were Black. Approximately 25% of the study population initiated ART within 28 days of HIV diagnosis (132 [12.9%] ≤14 days and 102 [10%] within 15 to 28 days), while 35% of patients initiated HIV therapy more than one year after diagnosis (Table 1). In total, 233 (22.8%), 337 (33.0%) and 452 (44.2%) patients initiated a PI‐, INSTI‐ or NNRTI‐based regimen, respectively. Darunavir (n = 94; 9.2%), elvitegravir (n = 148; 14.5%) and efavirenz (n = 283; 27.7%) were the most commonly used ART across classes, and most patients (n = 592; 57.9%) initiated on a single‐tablet regimen. The proportion of patients who had an emergency room visit between diagnosis and ART initiation increased from 6.1% for patients initiating ART within 14 days of HIV diagnosis to 53.5% for patients initiating therapy between 181 and 360 days of diagnosis (Table 1). Similarly, the proportion for inpatient admissions increased from 9.1% to 37.7% for these two cohorts, respectively.

**Table:** Abstract P284 – Table 1. Patients’ characteristics at HIV diagnosis and resource use measured between HIV diagnosis and ART initiation

	All patients	Time to ART initiation: ≤14 days	Time to ART initiation: 15 to 28 days	Time to ART initiation: 29 to 60 days	Time to ART initiation: 61 to 90 days	Time to ART initiation: 91 to 180 days	Time to ART initiation: 181 to 360 days	Time to ART initiation: >360 days
(N = 1022)	(N = 132)	(N = 102)	(N = 143)	(N = 51)	(N = 122)	(N = 114)	(N = 358)
Age at HIV diagnosis (years), mean ± SD [median]	40.0 ± 11.9 [40.9]	40.4 ± 11.7 [41.7]	40.2 ± 12.3 [41.4]	39.1 ± 13.7 [39.8]	41.1 ± 13.2 [41.5]	40.9 ± 11.9 [40.5]	40.2 ± 11.5 [40.8]	39.6 ± 11.0 [40.7]
Range (years)	(18.0 to 70.0)	(18.2 to 66.6)	(18.3 to 65.7)	(18.0 to 65.3)	(18.2 to 63.9)	(18.0 to 62.6)	(18.0 to 70.0)	(18.0 to 66.1)
Female, n (%)	451 (44.1%)	57 (43.2%)	51 (50.0%)	53 (37.1%)	22 (43.1%)	52 (42.6%)	49 (43.0%)	167 (46.6%)
Race, n (%)								
White	248 (24.3%)	35 (26.5%)	30 (29.4%)	39 (27.3%)	7 (13.7%)	23 (18.9%)	33 (28.9%)	81 (22.6%)
Black	582 (56.9%)	72 (54.5%)	47 (46.1%)	78 (54.5%)	35 (68.6%)	76 (62.3%)	54 (47.4%)	220 (61.5%)
Other	192 (18.8%)	25 (18.9%)	25 (24.5%)	26 (18.2%)	9 (17.6%)	23 (18.9%)	27 (23.7%)	57 (15.9%)
Patients who initiated a single‐tablet regimen, n (%)	592 (57.9%)	82 (62.1%)	60 (58.8%)	81 (56.6%)	26 (51.0%)	72 (59.0%)	65 (57.0%)	206 (57.5%)
Patients with ≥1 diagnosis of an opportunistic infection between diagnosis and ART initiation, n (%)	69 (6.8%)	6 (4.5%)	7 (6.9%)	7 (4.9%)	1 (2.0%)	9 (7.4%)	11 (9.6%)	28 (7.8%)
Patients with ≥1 emergency room between diagnosis and ART initiation, n (%)	438 (42.9%)	8 (6.1%)	11 (10.8%)	31 (21.7%)	12 (23.5%)	46 (37.7%)	61 (53.5%)	269 (75.1%)
Patients with ≥1 inpatient visit between diagnosis and ART initiation, n (%)	362 (35.4%)	12 (9.1%)	26 (25.5%)	39 (27.3%)	18 (35.3%)	36 (29.5%)	43 (37.7%)	188 (52.5%)

**Conclusions: **This study reveals that only 13% of Medicaid patients initiated ART within 14 days post‐diagnosis and that patients with early initiation of ART had better outcomes (fewer hospitalizations and OIs) between diagnosis and treatment initiation. Further research is warranted to assess the real‐world impact of rapid ART initiation on economic and clinical outcomes post‐initiation.

## P285

### Quality of life and experience of patients with HIV and other chronic diseases with Spanish health system: insights from the IEXPAC project

**M Galindo^1^, N Sanchez‐Vega^2^, M Cotarelo^2^, O Rincon^3^ and M Fuster^4^**

^1^Internal Medicine Service, Hospital Clínico de Valencia, Valencia, Spain. ^2^Medical Department, Merck Sharp & Dohme de España, Madrid, Spain. ^3^Medical Department, Merck Sharp & Dohme de España, Sevilla, Spain. ^4^Research Department, Sociedad Española Interdisciplinaria de SIDA (SEISIDA), Madrid, Spain

Improvements over the time in quality of care lead to a more positive experience for patients with chronic diseases. Careful measurement of patient's experience can provide meaningful data to further enhancements in quality of care, clinical effectiveness and patient's safety. The study describes the experience with health care system and health‐related quality of life (HRQoL) in Spanish patients with HIV (PHIV) infection and other chronic diseases. One thousand six hundred and eighteen patients participated in an observational cross‐sectional study. Surveys were handed to patients with four different chronic diseases with at least one comorbidity: PHIV, rheumatic diseases (RD), inflammatory bowel disease (IBD) or diabetes mellitus (DM). Patients filled anonymously the questionnaire at home and responded by pre‐paid mail. The experience with the health care system was measured through the validated IEXPAC scale (http://www.iemac.es/iexpac/). This scale contains 12 items with five responses from “always” to “never”, yields a score from 0 (worst) to 10 (best experience) and measures three dimensions: productive interactions, new relational model and patient self‐management. HRQoL and beliefs about medication were measured by EQ‐5D‐5L and BMQ questionnaires respectively. Two thousand four hundred and seventy‐four patients received the survey and 1618 were returned (65.4%): 467 corresponded to PHIV [mean age 51.5 ± 10.8 years, 27% women]. Responses to IEXPAC are displayed in Table 1. Mean IEXPAC score for PHIV was 6.6 ± 1.7. Patients declared a median of 8 visits to primary care or specialty clinics in the last year and 29% had visited an emergency room. In the last three years, 48% had been hospitalised. PHIV attended least frequently to primary care (76.20% vs. all 83.40%), declared a median of 3.89 visits to specialty clinics and reported higher % receiving once‐daily dosage (50.20% vs. all 31.80%). PHIV differ significantly in terms of: considering themselves well informed about their disease (82% vs. all 75%); their perception of need of medication, which is significantly the highest (22.20 ± 3.87); and being the least concerned about medication (13.32 ± 4.82). PHIV most often described no limitations in any of the five dimensions included in EQ‐5D‐5L; their scores on the visual analogue scale “Your health today” were the highest (73.3 ± 19.1) (all multiple comparison tests HIV infection vs other, *p* < 0.001). The IEXPAC questionnaire identified areas of improvement in chronic patients’ health care, especially those related with access to reliable information and services, interaction with other patients and continuity of health care after hospital discharge. PHIV scored the best, maybe consequence of a more personalised care and showed a better quality of life than patients with RD, IBD or DM.

**Table:** Abstract P285 – Table 1. Percentages of patients who responded “always” or “mostly” to the 12 IEXPAC items

Percentage (%) who responded	All patients	RD	IBD	HIV	DM	*p* value
They respect my lifestyle	81.5	76.5	75.1	89.6	81.9	<0.001
They are coordinated to offer good health care to me	69.3	60.6	69.1	76.8	73.3	<0.001
They help me to get information from the Internet	15.0	12.8	19.0	19.8	8.3	<0.001
Now I can take care of myself better	81.0	74.3	79.3	89.7	78.3	<0.001
They ask me and help me to follow my treatment plan	79.8	73.5	77.8	87.6	78.2	<0.001
We set goals for a healthy life and better control of my disease	70.1	63.4	62.6	74.7	76.1	<0.001
I can use Internet and my mobile phone to check my medical records	7.2	7.3	5.5	8.6	7.1	0.529
They make sure that I take medication correctly	76.0	72.4	73.7	83.5	72.9	<0.001
They worry about my wellbeing	84.3	79.1	80.4	91.5	83.8	<0.001
I have been informed on the health and social resources that can help me	41.3	33.8	32.3	52.6	42.3	<0.001
They encourage me to talk to other patients	14.9	10.3	15.7	20.4	12.0	<0.001
They care about me when I come home after being in the hospital (only if you have been admitted to hospital in last 3 years)	30.6	25.7	28.9	33.0	32.8	0.205
Global IEXPAC score, mean (SD)	6.0 (1.9)	5.5 (2.0)	5.9 (2.0)	6.6 (1.7)	5.9 (1.9)	<0.001

## P286

### Does AIDS mortality increase in Russia in spite of growing ART coverage?

**V Pokrovsky^1^, N Ladnaya^2^ and L Dementieva^3^**

^1^Central Research Institute of Epidemiology, RUDN University, Russian Federal AIDS Centre, Moscow, Russian Federation. ^2^Central Research Institute of Epidemiology, Russian Federal AIDS Centre, Moscow, Russian Federation. ^3^Department of Epidemiological Surveillance, Russian Federal Service for Surveillance on Consumer Rights Protection and Human Wellbeing, Moscow, Russian Federation

**Background: **Though a number of sources declare reductions in a number of deaths attributed to HIV/AIDS as a result of growing ART coverage, this opinion is based mainly on estimations. Official statistical data collected by responsible independent agencies may be controversial.

**Methods: **We combined databases on annual numbers of death among PLWHIV by the main cause collected by the Russian Federal State Statistics Service (Rosstat) and HIV/AIDS statistic data collected by Russian Federal Service for Surveillance on Consumer Rights Protection and Human Wellbeing (Rospotrebnadzor).

**Results: **HIV‐infection morbidity, mortality and coverage with ART are shown in Table 1.

**Table:** **Abstract P286 –** Table 1. Annual changes of HIV‐infection morbidity, mortality and coverage with ART

Year	2014	2015	2016	2017
Number of registered PLWHIV at the end of year	728,732	798,798	870,952	943,999
Number of PLWHIV deaths per year	24,416	27,564	30,550	31,898
Percent of PLWHIV died per year	3.2%	3.3%	3.4%	3.3%
Number reported HIV/AIDS‐related deaths per year	12,540	15,520	18,577	20,045
Percent HIV/AIDS‐related deaths of all registered PLWHIV	1.7%	1.9%	2.1%	2.1%
Percent HIV/AIDS‐related deaths among all PLWHIV died per year	51.4%	56.3%	60.8%	62.8%
Number of PLWHIV receiving any ART in a year	188,096	230,022	285,920	346,132
Percent of PLWHIV receiving ART per year	25.0%	27.8%	31.7%	35.5%

**Conclusions: **Though the total number and percentage of PLWHIV receiving ART has increased significantly, the percentage of annual deaths among PLWHIV has not changed and the total number of annual AIDS deaths keeps growing. Though the data collecting methodology of different agencies may be discussed, but the figures show that only ART coverage alone cannot lead to reduction in HIV/AIDS death rates. It shall also be accompanied by improvements in prevention of new HIV infections, early diagnostics and early ART start along with continuous support of adherence. Age of HIV acquiring and duration of life with HIV before and after ART start shall be taken in account for more correct interpretation.

## P287

### The impact of co‐payment ART cards on HIV biomarkers among persons with HIV followed at an ambulatory clinic: results from the McGill University Health Center, Montreal, Canada

**A Abulkhir^1^, B Lemire^1^, R Nitulescu^1^, K Engler^2^, C Pexos^1^, N Kronfli^1^, M Klein^1^, J Cox^3^ and B Lebouche^1^**

^1^Chronic Viral Illness Service, McGill University Health Centre, Montreal, Canada. ^2^Centre for Health Outcomes Research, McGill University Health Centre, Montreal, Canada. ^3^Department of Epidemiology and Occupational Health, McGill University Health Centre, Montreal, Canada

**Background: **Many Quebec residents must pay a portion of the costs of prescription drugs up to a maximum of 1000 CAD annually. Several pharmaceutical companies have created co‐payment programs to assist persons with HIV who have financial difficulties. Assistance is provided to either cover the monthly contribution for ART or to supply ART free of charge in the absence of insurance coverage. However, little is known regarding the impact of co‐payment cards on persons with HIV in Canada. The aim of this study was to (1) identify reasons for seeking co‐payment cards; (2) specify the pharmaceutical agents for which cards were received; and (3) assess the effect of the co‐payment programs on HIV biomarkers (CD4 and viral load).

**Methods: **Persons with HIV receiving care at the McGill University Health Centre (MUHC), Montreal, Quebec, who received co‐payment cards in 2017, were selected for this study. Subject‐level data were extracted from the MUHC database. Two observation periods were considered for each participant: six months prior to and after the receipt of a co‐payment card. To assess the short‐term effect of the co‐payment program on HIV biomarkers (i.e. CD4 cell count and log10 HIV RNA viral load [VL]), we used a mixed‐effects linear regression model with a random intercept for each patient and a binary indicator identifying the observation period (i.e. before vs after the co‐payment card was received). Secondary analyses were undertaken to document use of on‐site pharmacy services, again before and after co‐payment cards were received.

**Results: **Overall, 63 persons with HIV were included, 24 of whom were female. Reasons for seeking co‐payment cards include financial difficulties (46%), no health insurance coverage (43%) and unstable coverage (11%). Cards were mostly provided for FTC/TAF (20 cards), followed closely by EVG/COBI/FTC/TAF (16 cards). Cards were also provided for DTG/ABC/3TC (10 cards), FTC/RPV/TDF (six cards), DTG‐based regimen (five cards), and EVG/COBI/TDF/FTC (four cards). The 6‐month follow‐up period was associated with an increase of 15 CD4 cells (95% CI ‐42 to 71) and a decrease of 0.7 log10 copies of HIV RNA (95% CI ‐1.1 to ‐0.4). The number of patients with undetectable HIV RNA (≤50 copies) remained relatively stable over time as did the number of on‐site pharmacy visits.

**Conclusion: **The provision of co‐payment cards to facilitate access to ART was associated with beneficial short‐term (6‐month) effects on HIV biomarkers.

## VIROLOGY AND IMMUNOLOGY: BIOMARKERS

## P288

### HIV DNA undetectability during chronic HIV infection: frequency and predictive factors

**S Nozza^1^, L Galli^1^, N Gianotti^1^, N Galizzi^2^, A Poli^1^, P Cinque^1^, V Spagnuolo^2^, A Lazzarin^1^, G Tambussi^1^ and A Castagna^2^**

^1^Infectious Diseases, San Raffaele Scientific Institute, Milano, Italy. ^2^Universita’ Vita Salute, San Raffaele Scientific Institute, Milano, Italy

**Background: **HIV DNA is a marker of HIV reservoirs. Objectives of the study were to determine prevalence of undetectable HIV DNA and to identify factors associated with this in a cohort of HIV‐1 infected patients (pts) treated with ART and with undetectable viral load (VL).

**Materials and methods: **Cross‐sectional study on HIV‐1 infected pts followed at the Department of Infectious Diseases of San Raffaele Scientific Institute on stable ART, with availability of previous ART HIV RNA and with undetectable VL since ≥12 months. HIV DNA was amplified and quantified by real‐time PCR (ABI Prism 7900); limit of detectability is 100 copies/106 PBMC. Pts who were tested for HIV RNA, HIV DNA and immunological profile (CD4, CD8) at the same time were considered in the analysis. Results were described by median (IQR) or frequency (%). Logistic regression was used to identify predictive factors for HIV DNA <100 copies/106 PBMC.

**Results: **Four hundred and sixty‐eight pts considered in the analyses, 119 (25%) with HIV DNA <100 copies/106 PBMC. Pts’ characteristics at HIV DNA determination: age 37 (32 to 44) years, 18% female, 13% with previous AIDS‐defining event, HIV diagnosis since 11.2 (6.3 to 16.9) years, on ART since 7.9 (4.2 to 13.4) years, ART started within 16.2 (3.4 to 54.2) months since HIV diagnosis, undetectable VL since 3.9 (1.6 to 8.1) years, CD4 nadir 260 (165 to 345) cells/µL, CD4 718 (532 to 913) cells/µL, CD4/CD8 ratio 0.89 (0.59 to 1.24). Pre‐ART HIV RNA was 4.71 (4.13 to 5.14) log10 copies/mL, 22% currently treated with an integrase inhibitors regimen. At multivariate analysis, we found that pts with lower pre‐ART HIV RNA [adjusted odds ratio (AOR) per log10 copies/mL higher 1.48, 95% CI 1.12 to 1.98, *p* = 0.007], higher nadir CD4 [AOR per 50 cells/µL higher 0.91, 95% CI 0.83 to 0.99, *p* = 0.046] and a shorter time to ART start [AOR per 6 months higher 1.04, 95% CI 1.01 to 1.07, *p* = 0.019] were more likely to have HIV DNA <100 copies/106 PBMC, after adjustment for age, gender, calendar year of ART start, type of current ART regimen, time on undetectable VL since ART start, current CD4 and CD4/CD8 ratio. Prevalence of HIV DNA <100 copies/106 PBMC according to these factors is shown in Figure 1.

**Abstract P288 – Figure 1.** Prevalence of HIVDNA <100 copies/106 PBMC according to different factors.

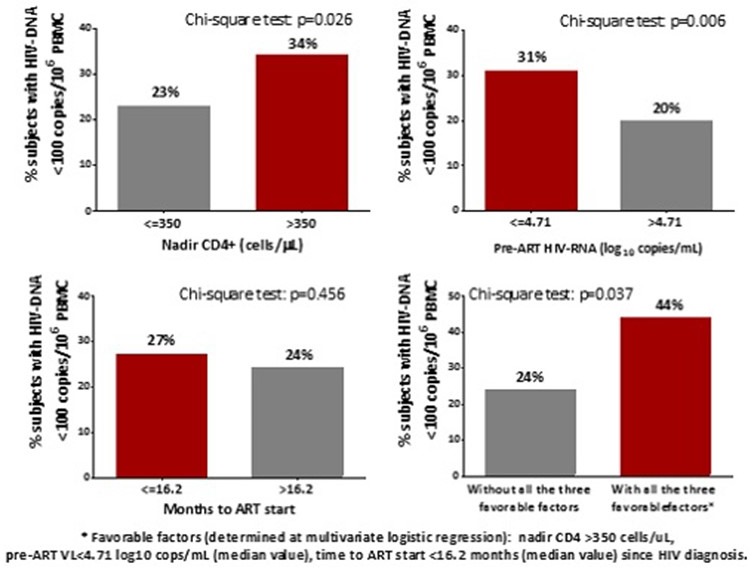

**Conclusions: **In our chronic HIV‐infected pts, undetectable from 4 years, frequency of undetectable viral load is 25%. Lower pre‐ART HIV RNA, shorter time between HIV diagnosis and starting ART and higher CD4 nadir were associated with undetectable HIV DNA. These findings may inform about selection of pts candidate to structured therapy interruption.

## P289

### Ability of a real‐time PCR to detect for the presence of HLA B*57:01 allele using DNA extracted from whole blood dried spots

**D Fofana^1^, V Calvez^2^, A Marcelin^2^ and A Maiga^3^**

^1^Virology, University of Bamako, Bamako, Mali. ^2^Virology, Sorbonne University Inserm U1136, Paris, France. ^3^Virology, Gabriel Toure University Hospital, Bamako, Mali

**Background: **Abacavir (ABC) is a NRTI recommended for the treatment of HIV‐1 infection. It is a potent medication but has a limiting toxicity of hypersensitivity reaction (HSR), in 2 to 8% of treated patients. HSR to ABC is significantly associated with carriage of the HLA‐B*57:01 allele and HLA‐B*57:01 testing is recommended to be performed prior to ABC initiation. Some real‐time PCR assays were developed for HLA‐B*57:01 testing but have not been validated using whole blood blotted. We assessed the ability of a real‐time PCR to detect for the presence of HLA B*57:01 allele using DNA extracted from whole blood spotted on blotters.

**Materials and methods: **A total of 200 genomic DNA samples were tested: 70 were HLA‐B*57:01 allele positive and 130 HLA‐B*57:01 allele negative. Whole blood belonged to HIV carriers, previously screened for HLA‐B*57:01, was spotted on blotters and dry blood spots and stored at room temperature for 2 weeks before use. Total DNA was extracted from 50 μL of whole blood spotted on blotters by using the EZ1 Advanced XL Qiagen instrument with the EZ1 DSP Virus Kit. DNA was measured spectrophotometrically and diluted to 10 ng/μL. Three primers target the HLA B*57:01 allele: HLA1 (5′‐GTCTCACATCATCCAGGT‐3′), HLA2 (5′‐ATCCTTGCCGTCGTAGGCGG‐3′) and HLA3 (5′‐ATCCTTGCCGTCGTAGGCAG‐3′) [1]. The reverse primers HLA2 and HLA3 target the HLA B*57:0101 and B*57:0102 alleles, respectively, both coupled with the invariant forward primer HLA1, yielding a PCR product of 96 base pairs in length.

**Results: **Our results show that our assay used from DNA extracted from dried blood spots was 100% sensitive, detecting all HLA‐B*57:01 allele‐positive patients and 100% specific, having no false‐positive results achieving a 100% specificity and 100% sensitivity on this control panel.

**Conclusion: **Dried blood spots are an alternative specimen type for HIV drug resistance genotyping and HIV‐1 diagnosis in patients in resource‐limited settings but can also be used to detect the presence of HLA B*57:01 allele.

**Reference: **[1] Martin et al., 2005.

## P290

### The effect of first‐choice antiretroviral agents on mesenchymal stem cell commitment

**S De Rose^1^, A Cazzaniga^2^, V Romeo^2^, J Maier^2^ and S Rusconi^1^**

^1^Infectious Diseases Unit, DIBIC Luigi Sacco, University of Milan, Milan, Italy. ^2^General Pathology Lab, DIBIC Luigi Sacco, University of Milan, Milan, Italy

**Background: **HAART involves the administration of two or more compounds, in order to both have a multi‐target effect against the virus and to reduce the risk of developing resistance. Unfortunately, such drugs may cause very serious side effects on the long period, including osteopenia and eventually osteoporosis. Since mesenchymal stem cells (MSCs) are the cellular precursors of osteoblasts [1], a link might exist between the administration of HAART and the effect on the osteoblast lineage. The aim of the study is to demonstrate if and in what extent first‐choice molecules such as the protease inhibitor darunavir (DRV) and the integrase inhibitor dolutegravir (DTG) may affect the MSCs commitment.

**Materials and methods: **Experiments were preliminarily performed on a human stromal cell line (L88) [2]. Both drugs were used at their IC90 concentrations, i.e. 2.43 ng/mL (DRV), 0.064 µg/mL (DTG). Toxicity was evaluated through the MTT test on four concentrations: 2xIC90, IC90, 1/2xIC90, 1/4xIC90. Subsequently, cells were incubated both in the presence and in the absence of bone differentiation medium containing 2 x 10 to 8 M 1a,25‐Dihydroxyvitamin D3, 10 mM b‐glycerolphosphate and 0.05 mM ascorbic acid, with or without each drug. At Day 2 and Day 4 the RNA was harvested, reverse transcription and qPCR were carried out and the expression of RUNX2, which is known to be the principal trigger of osteogenesis, was evaluated [3].

**Results: **The drugs did not appear to be toxic at any of the tested concentrations. To study gene expression, 2xIC90 concentration was used. qPCR showed that the osteogenic cocktail induced the expression of *RUNX2* compared to the control (Figures 1a–b). Conversely, the addition of DTG [2xIC90] to the osteogenic mixture seems to lead to an inhibition of *RUNX2* expression after 4 days (Figure 1b).

**Abstract P290 – Figure 1a.** Expression of RUNX2 after 2 days of treatment with darunavir and dolutegravir compared with controls.

**Abstract P290 – Figure 1b.** Expression of RUNX2 after 4 days of treatment with darunavir and dolutegravir compared with controls.

‐ = drug‐untreated condition; CTR = control; DIFF = differentiation medium. *p < 0.05; ** *p* < 0.01.

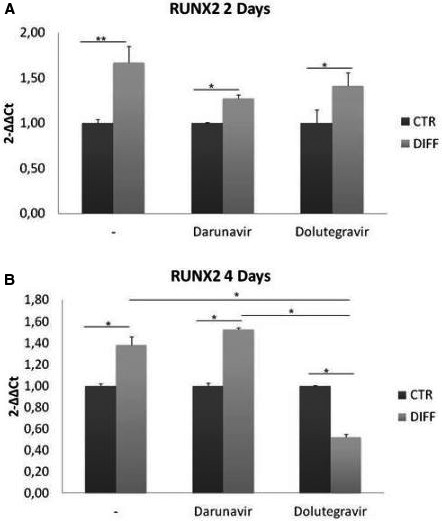

**Conclusions: **From our preliminary data, it is reasonable to speculate that DRV, after an apparent delay, does not affect the differentiation; instead DTG may be capable to block osteogenesis through the inhibition of *RUNX2* already after four days of treatment in vitro. In the future, it would be interesting to investigate the effect of these same drugs on other important genes involved in osteoblast differentiation such as us *COL1A1* and *OSTERIX,* osteclacin (*OSC*) and osteopontin (*OSP*) (late differentiation) [4]. Since MSCs are also adipocytes cellular progenitors [4] and HAART affects this cell lineage too, the effect  of HIV inhibitors on adipocyte differentiation will be investigated.

**References**

[1] Kern S, Eichler H, Stoeve J, Klüter H, Bieback K. Comparative analysis of mesenchymal stem cells from bone marrow, umbilical cord blood, or adipose tissue. Stem Cells. 2006;24:1294‐301.

[2] Thalmeier K, Meissner P, Moosmann S, Sagebiel S, Wiest I, Huss R. Mesenchymal differentiation and organ distribution of established human stromal cell lines in NOD/SCID mice. Acta Haematol. 2001;105:159‐65.

[3] Komori T. Runx2, an inducer of osteoblast and chondrocyte differentiation. Histochem Cell Biol. 2018;149:313‐23.

[4] Carr A. HIV protease inhibitor‐related lipodystrophy syndrome. Clin Infect Dis. 2000;30 Suppl 2:S135‐42.

## P291

### Longitudinal analysis of proviral HIV‐DNA

**E Heger^1^, S Peters^1^, M Onyuro^1^, T Kümmerle^2^, E Voigt^2^, V Di Cristanziano^1^, S Sierra^1^, L Huesgen^1^, H Kulartz^1^, R Kaiser^1^, C Wyen^2^ and E Knops^1^**

^1^Institute of Virology, University of Cologne, Cologne, Germany. ^2^Private Practice, Ebertplatz, Cologne, Germany

**Background: **HIV replication can be measured as viral load (VL) in plasma and is a suitable and approved marker for monitoring treatment success. Sustained plasma VL below the LOD is the goal in treatment strategy of HIV patients. In time of successful long‐term therapies and upcoming cure strategies there is need for a further marker to analyse influence of a certain drug combination on proviral reservoir and for long‐term monitoring of proviral reservoir. In order to analyse dynamics of proviral load (pVL) and evolution of proviruses in patients with undetectable plasma viral load we are examining longitudinal collected samples from HIV patients.

**Materials and methods: **Three consequent samples from each patient (n = 72) were collected during a period of 12 months and total proviral DNA from PBMCs was extracted. Proviral load (cop) and PBMC count were measured in a real‐time PCR to calculate pVL (log cop/Mio cells). Data were analysed with regard to plasma VL, CD4 cell counts, treatment combinations and also period of undetectable plasma VL. Patients were grouped according to their treatment regimen: without treatment (no, 4%), NRTI‐INI (44%), NRTI‐NNRTI (24%), NRTI‐PI (15%), other combinations [NNRTI‐PI, NNRTI‐INI‐PI, INI‐PI] (6%) and treatment change (7%). Sequence distances were analysed using Mega7.

**Results: **In our study mean pVL was 2.36 log10 total HIV‐1 DNA / Mio wbc (0.63 to 3.26). Mean pVL of measurements from three consequent time points was significantly higher in PI‐group compared to INI‐group (*p* = 0.025) and by trend higher compared to NNRTI group (*p* = 0.058). In order to include the CD4 cell count into the analysis of the pVL the quotient of pVL and CD4 cell count was determined. Here, mean of the quotient was significantly higher in PI‐group compared to INI‐ and NNRTI‐groups (*p* = 0.045 each). Evolution analysis of proviruses included protease and reverse transcriptase amplicons (1450 bp) of 56 patients. In 24 cases evolution (as distance from the first sample) could be observed. There was no significant difference in treatment composition between these two groups. Twenty percent of patients showed RT‐RAMs and 6% PI‐RAMs. In 50% of all patients APOBEC‐induced mutations could be found, where M184I and M230I were most prevalent mutations.

**Conclusions: **Based on observation that pVL in patients receiving standard treatment NRTI‐PI is higher compared to other therapy combinations, role of PI regimens in sustainment of proviral reservoir has to be determined. There is also need to examine in detail which factors contribute to evolution in proviral DNA in spite of suppressed plasma VL.

## P292

### HLA‐B*57:01 allele prevalence in Turkish HIV‐infected patients and the value of real‐time PCR allele testing compared with sequence specific primer technique

**D Inan^1^, M Sayan^2^, A Deveci^3^ and F Ucar^4^**

^1^Department of Infectious Diseases, Akdeniz University, Faculty of Medicine, Antalya, Turkey. ^2^Clinical Laboratory, PCR Unit and Res Center of Exp H Science, Kocaeli University, Faculty of Medicine and Near East University, Kocaeli (and Nicosia, Northern Cyprus), Turkey. ^3^Department of Infectious Diseases, Ondokuz Mayıs University, Faculty of Medicine, Samsun, Turkey. ^4^Department of Medical Biology and Genetics, Akdeniz University, Faculty of Medicine, Antalya, Turkey

**Background: **Abacavir (ABC) is a potent nucleoside analogue reverse transcriptase inhibitor that is used in combination with other antiretroviral agents to treat HIV‐infected patients. ABC‐containing single tablet regimen is eventually available in our country last two years. The HLA‐B*57:01 allele is associated with a higher risk of ABC‐associated hypersensitivity reaction. The HIV treatment guidelines endorse HLA‐B*57:01 screening in HIV‐positive patients prior to initiating ABC‐containing regimens [1]. The purpose of this study was to assess the prevalence of HLA‐B*57:01 allele in Turkish HIV‐1 positive patients from two different regions of Turkey. We also sought to find the performance of the real‐time PCR‐based test for screening HLA‐B*57:01 allele.

**Materials and methods: **Plasma samples from 487 HIV‐1 positive patients were screened for HLA‐B*57:01 allele using with sequence specific primer (SSP) technique (OlerupSSP, West Chester, PA, USA) method. Of 109 patients were also evaluated real‐time PCR method (GenVinSet, HLA‐B*57:01 Genotyping Kit, BDR, Spain).

**Results: **Of 260 HIV‐1 infected patients from Antalya, located in southern Turkey, three (1.1%) had the HLA‐B*57:01 allele; in contrast, in Samsun, representing northern Turkey, 11 of 227 patients (4.8%) had this allele. The difference was not statistically significant (*p* ≥ 0.05). Totally 109 plasma samples, tested by SSP were also analysed by real‐time PCR. Compared with SSP, real‐time PCR had 33% sensitivity and 94% specificity for the HLA‐B*57:01 allele. For real‐time PCR positive predictive value (PPV) and negative predictive value (NPV) were 14% and 98%, respectively. Two samples that found positive allele testing with SSP were negative with real‐time PCR.

**Conclusion: **The prevalence of HLA‐B*57:01 allele in Turkish HIV‐1 infected population was found to be between 1.1% and 4.8% which is lower than in Caucasians but higher than Asian populations [2–4]. However, real‐time based PCR allele test that was used in this study had lower sensitivity and PPV. Therefore, real‐time PCR positive results must be confirmed by SSP technique. More sensitive methods with high resolution such as SSP are recommended for accurate diagnosis.

**References**

[1] European AIDS Clinical Society. Guideline v.9.0 [Internet]. October 2017. Available from http://www.eacsociety.org.

[2] Orkin C, Sadiq ST, Rice L, Jackson F. Prospective epidemiological study of the prevalence of human leukocyte antigen (HLA)‐B*5701 in HIV‐1‐infected UK subjects. HIV Med. 2010;11:187‐92.

[3] Zhang H, Zhang T, Zhao H, Han N, Zhou H, He Y, et al. Low prevalence of human leukocyte antigen‐B*5701 in HIV‐1‐infected Chinese subjects: a prospective epidemiological investigation. AIDS Res Ther. 2015;12:28.

[4] Small CB, Margolis DA, Shaefer MS, Ross LL. HLA‐B*57:01 allele prevalence in HIVinfected North American subjects and the impact of allele testing on the incidence of abacavir‐associated hypersensitivity reaction in HLA‐B*57:01‐negative subjects. BMC Infect Dis. 2017;17:256.

## P293

### Multicentre evaluation of two next‐generation HIV‐1 quantitation assays, Aptima Quant Dx and Cobas 6800, in comparison to the RealTime HIV‐1 reference assay

**P Braun^1^, F Wiesmann^1^, G Naeth^1^, M Däumer^2^, R Ehret^3^, R Kaiser^4^, C Noah^5^, M Obermeier^3^, G Schalasta^6^, C Tiemann^7^, E Wolf^8^ and H Knechten^1^**

^1^HIV & Hepatitis Research Group, PZB Aachen, Aachen, Germany. ^2^Institute for Immunology and Genetics, Kaiserslautern, Germany. ^3^Medical Laboratory Berlin, Berlin, Germany. ^4^Institute of Virology, University of Cologne, Cologne, Germany. ^5^Laboratories, MVZ Labor Lademannbogen, Hamburg, Germany. ^6^Laboratory for Medical Diagnostics, Laboratory Enders, Stuttgart, Germany. ^7^Laboratory for Medical Diagnostics, Labor Krone, Bad Salzuflen, Germany. ^8^Virology, MUC Research, Munich, Germany

**Background: **A high accuracy and precision at the lower end of quantification is a crucial requirement of a modern HIV viral load (VL) assay since some clinically relevant thresholds are located at 50 and 200 copies/mL. In this study, we compared the performance of two new fully automated HIV‐1 VL assays, Aptima HIV‐1 Quant Dx and Cobas HIV‐1 (Cobas 6800), with the established RealTime m2000 assay.

**Materials and methods: **Assay precision and accuracy was evaluated in a retrospective evaluation out of excess plasma material from four HIV‐1+ individuals (subtypes B, C, AE and AG). Native plasma samples were diluted to nominal concentrations at 50 and 200 copies/mL (according to RealTime m2000). All dilutions were tested in triplicates in five independent runs over five days and in three labs per system. Assay concordance was determined using 1011 surplus clinical routine samples as well as selected retrospective longitudinal samples from seven patients on treatment.

**Results: **The three assays yielded highly concordant results for individual clinical samples (R2 > 0.98; average difference ≤0.2 log copies/mL) and retrospective longitudinal samples from patients on treatment. Aptima and RealTime showed similar high precision, meeting the 5σ‐criterion for the majority of samples across all labs and subtypes. Cobas was less precise, missing the 5σ‐criterion for the majority of samples. Aptima and RealTime results differed more noticeably from Cobas 6800 results, particularly for subtypes C and AE in clinical follow‐up samples (range 0.33 to 0.56 log). In precision analysis at 50 copies/mL and 200 copies/mL, mean, median and standard deviation turned out to be higher for Cobas 6800 as compared to Aptima and RealTime for all subtypes. Across all subtypes, coefficients of variation for absolute values at 50 copies/mL and 200 copies/mL ranged from 22% to 34% and 20% to 33% for Aptima, from 35% to 51% and 23% to 42% for Cobas and 26% to 37% and 21% to 35% for RealTime, respectively.

**Conclusions: **In this analysis, results from Cobas appeared less reliable near the clinically relevant cutoff and should be interpreted with more caution in this context. Aptima and RealTime differed more noticeably from Cobas 6800 results but showed a high agreement between their results at this low viraemic level. As an important criterion, this may ease the interpretation of Aptima results in comparison to previous RealTime results. In this context, low viraemic Cobas 6800 results after RealTime monitoring may require a closer view.

## VIROLOGY AND IMMUNOLOGY: RESISTANCE

## P294

### Week 48 resistance analyses of the once‐daily, single‐tablet regimen (STR) darunavir/cobicistat/emtricitabine/tenofovir alafenamide (D/C/F/TAF) in HIV‐1‐infected adults from the AMBER and EMERALD Phase III trials

**E Lathouwers^1^, E Wong^2^, K Brown^1^, B Baugh^3^, A Ghys^1^, J Jezorwski^3^, E Van Landuyt^1^, M Opsomer^1^ and S De Meyer^1^**

^1^Janssen, Pharmaceutica NV, Beerse, Belgium. ^2^Janssen, Scientific Affairs LLC, Titusville, NJ, USA. ^3^Janssen, Research & Development LLC, Raritan, NJ, USA

The once‐daily STr D/C/F/TAF 800/150/200/10 mg is approved in the EU, under regulatory review in the US and is being investigated in two international, randomised Phase III trials. D/C/F/TAF had non‐inferior efficacy versus D/C+F/TDF in HIV‐1‐infected, ART‐naïve adults (AMBER; NCT02431247) and versus bPI+F/TDF in ART‐experienced, virologically suppressed adults on a bPI+F/TDF regimen (EMERALD; NCT02269917). In EMERALD, patients with prior antiretroviral experience (58% had received ≥5 antiretrovirals) and prior VF (15%), including patients with emtricitabine and tenofovir resistance‐associated mutations (RAMs), were eligible for study participation. Only if historical genotypes were available, patients with darunavir RAMs were excluded. Week 48 AMBER and EMERALD resistance analyses are presented. Samples for genotyping/phenotyping were selected in patients with protocol‐defined VF (PDVF) in AMBER (virological non‐response, virological rebound and/or viraemic at final timepoint) or EMERALD (rebound) and with VL ≥400 copies/mL at failure or later timepoints. In AMBER, resistance testing was performed using GenoSure^®^MG at screening and PhenoSense^®^GT post‐baseline. Deep sequencing was performed post‐hoc using Illumina MiSeq on samples from one patient. In EMERALD, GenoSure^®^MG was used for resistance testing post‐baseline. In addition, HIV‐1 proviral DNA from baseline samples (VL <50 copies/mL) was analysed post‐hoc using GenoSure Archive^®^ to assess the prevalence of archived RAMs in patients with prior VF (N = 169), i.e. the most treatment‐experienced patients in this study. Through Week 48 across both studies, no darunavir, primary PI or tenofovir RAMs were observed in 1125 patients receiving D/C/F/TAF, or in 629 patients receiving boosted darunavir+F/TDF in the control groups (Table 1). In AMBER during the treatment phase, the NRTI RAM M184I/V, conferring phenotypic resistance to emtricitabine and lamivudine, was identified in one D/C/F/TAF patient; M184V was detected pre‐treatment by deep sequencing as a minority variant (9%). This patient also had transmitted NNRTI (efavirenz/nevirapine) resistance shown by the presence of K103N at screening, and discontinued the study due to noncompliance. In EMERALD patients with prior VF and geno‐archive data (N = 140), 14% had darunavir RAMs, 5% tenofovir RAMs and 38% emtricitabine RAMs, mainly at RT position M184 (Table 2). All these patients achieved VL <50 copies/mL at Week 48 or at last on‐treatment VL. D/C/F/TAF has a high genetic barrier to resistance; no darunavir, primary PI or tenofovir RAMs were observed through 48 weeks in AMBER and EMERALD. In only one PDVF (AMBER D/C/F/TAF group) M184I/V was observed. In EMERALD, baseline archived RAMs to darunavir, emtricitabine and tenofovir were observed in patients with prior VF and did not preclude virological response.

**Table:** Abstract P294 – Table 1. Post‐baseline resistance through Week 48 evaluated in patients with PDVF in the AMBER and EMERALD Phase III clinical studies

Patients with ≥1 RAM post‐baseline, n (%)						
					Reverse transcriptase	Protease
Study	ART	Patients, N	Patients with PDVF, n (%)	PDVF patients evaluated for resistance, n (%)	FTC/TFV	Primary PI/darunavir
**AMBER**	**D/C/F/TAF**	**362**	**8 (2.2)**	**7 (1.9)**	**M184I/V^a^; n = 1**	**0**
	D/C+F/TDF	363	6 (1.7)	2 (0.6)	0	0
	**D/C/F/TAF**	**763**	**19 (2.5)**	**1 (0.1)**	**0**	**0**
**EMERALD**	bPI^b^+F/TDF	378	8 (2.1)	3 (0.8)	0	0
**Total**	**D/C/F/TAF**	**1125**	**27 (2.4)**	**8 (0.7)**	**1 (0.1)**	**0**

^a^conferring phenotypic resistance to emtricitabine and lamivudine, but retaining sensitivity to abacavir, tenofovir, stavudine and zidovudine; this patient had transmitted efavirenz and nevirapine resistance shown by the presence of K103N at screening; M184V was detected by deep sequencing (9.4%) at screening. ^b^266 patients were on boosted darunavir (n = 202 darunavir/ritonavir; n = 64 darunavir/cobicistat) at screening. RAM = resistance‐associated mutation; ART = antiretroviral treatment; FTC = emtricitabine; TFV = tenofovir; bPI = boosted protease inhibitor; D/C/F/TAF = darunavir/cobicistat/emtricitabine/tenofovir alafenamide; F/TDF = emtricitabine/tenofovir disoproxil fumarate.

**Table:** **Abstract P294 –** Table 2. Prevalence of baseline RAMs^a^ in HIV‐1 proviral DNA for patients with previous virological failure in the EMERALD study

	D/C/F/TAF (N = 763)	bPI+F/TDF (N = 378)	Total
Patients with previous virologic failure, N	116	53	169
Patients with previous virologic failure and geno archive data at baseline, N	98	42	140
**≥1 darunavir RAMs, n (%)**	16^b^ (16.3)	4 (9.5)	20 (14.3)
I50I/V	7 (14.0)	1 (2.4)	8 (5.7)
I84I/V	4 (4.1)	1 (2.4)	5 (3.6)
L33L/F	0	1 (2.4)	1 (0.7)
L76L/V	1 (1.0)	0	1 (0.7)
T74T/P	0	1 (2.4)	1 (0.7)
V32V/I	5 (5.1)	0	5 (3.6)
**≥1 tenofovir RAMs, n (%)**	6 (6.1)	1 (2.4)	7 (5.0)
K65KR	4 (4.1)	0	4 (2.9)
K70K/D/E/N/Q	2 (2.0)	1 (2.4)	3 (2.1)
**≥1 emtricitabine RAMs, n (%)**	35 (35.7)	18 (42.9)	53 (37.9)
K65K/R	4 (4.1)	0	4 (2.9)
M184M/I/V	31 (31.6)	18 (42.9)	49 (35.0)

The denominator is patients with previous virologic failure and geno archive data at baseline. ^a^IAS‐USA 2017 mutation list; observed mutations were concatenated. ^b^in one patient (D/C/F/TAF arm) 2 DRV RAMs were observed (I84I/V and L76L/V).

## P295

### Clinical consequences of failing PI‐ and DTG‐based two drug combinations versus PI‐ and INI‐based triple therapies in HIV patients without previous virological failures

**V Calvez^1^, D Armenia^2^, C Charpentier^3^, M Santoro^2^, C Soulie^1^, M Wirden^1^, C Perno^4^, D Descamps^3^, F Ceccherini‐Silberstein^2^ and A Marcelin^1^**

^1^Virology, Sorbonne University Inserm U1136, Paris, France. ^2^Virology, Tor Vergata University, Rome, Italy. ^3^Virology, Sorbonne Paris Cité, Inserm IAME, UMR 1137, Paris, France. ^4^Virology, University of Milan, Milan, Italy

**Background: **Guidelines recommend exclusively triple therapies (TT) for initial treatment of HIV patients, whereas in the switch setting some two drug combinations (2DC) have recently been included in some European guidelines as alternative regimens for suppressed patients as maintenance strategies. Previous studies have shown that, when used in routine practice, 2DCs are associated with more resistance development in case of virological failures (VF) compared to most TT. The aim of this study was to compare the consequences of failures to integrase inhibitor (INI)‐based or protease inhibitor (PI)‐based TT versus 2DC in patients with no previous VF in their therapeutic histories.

**Materials and methods: **Genotypic resistance testing was performed at time of first VF in patients failing TT (PI or INI) or 2DC (PI and/or DTG‐containing regimen). Virological failure was defined as occurrence of two consecutive HIV plasma viral loads (VL) >50 copies/mL. One hundred patients failing INI‐based TT (RAL, EVG/cobi, DTG) and 100 failing PI‐based TT (DRV/r ATV/r (consecutive in the database)) were included, and all eligible patients failing DTG‐based 2DC (DTG+3TC, DTG+RPV) or PI‐based 2DC (PI + RAL, DTG or 3TC). Study period was 2015 to 2018 for genotypic testing.

**Results: **Twenty‐three patients who had experienced their first virological failure were included in the DTG‐based 2DC group and 32 in the PI‐based 2DC group. Median age was 39 years for TT, 41 for 2DC; median number of previous regimens was 2 for TT, 3 for 2DC; median zenith VL was 79,239 copies/mL for TT, 156,966 for 2DC, and median VL at time of failure was 3019 copies/mL for TT, 4313 for 2DC. Three percent of PI TT patients, 7.0% of INI TT patients (0.0% of DTG‐based TT), 21.7% of DTG 2DC patients and 37.5% of PI 2DC patients presented emergent resistance after failure. Table 1 shows the characteristics of the subsequent regimen after failure (% of PI use, % of multi‐tablet regimens) depending on the initial regimen group.

**Table:** **Abstract P295 –** Table 1. Emergent resistance and characteristics of subsequent regimen after failure

	2NRTIs+PI	2NRTIs+INI	DTG‐based 2DC	PI‐based 2DC
N	100	100	23	32
Emergent resistance, %	3.0	7.0	21.7	37.5
PI use, %	3.0	7.0	39.1	65.6
MTR use, %	4.0	7.0	52.2	65.6

**Conclusions: **Higher rates of drug resistance selection were observed in patients failing a DTG‐ or PI‐containing 2DC comparing to INI‐ or PI‐containing TT. Patients on 2DC are switched after failure to more complex regimens, containing PIs and higher number of tablets than patients failing TT.

## P296

### Frequent detection of drug resistance mutations by deep sequencing in patients with documented extensive resistance and long‐lasting viral suppression: the proviral DNA archive remains stable for decades

**C Hoffmann^1^, A Thielen^2^, E Wolf^3^, M Bickel^4^, A Stoehr^5^, P Braun^6^, H Knechten^6^, S Esser^7^, C Wyen^8^, I Krznaric^9^, M Müller^10^, J Brust^11^, J Wasmuth^12^, H Horst^13^, S Holm^14^ and M Däumer^2^**

^1^ICH Study Center, ICH Stadtmitte, Hamburg, Germany. ^2^Virology, Institute of Immunology and Genetics, Kaiserslautern, Germany. ^3^MUC Research, MVZ Karlsplatz, Munich, Germany. ^4^Infektiologikum, Frankfurt, Germany. ^5^IFI Institute, Hamburg, Germany. ^6^Praxiszentrum, Aachen, Germany. ^7^Dermatology, University of Essen, Essen, Germany. ^8^Praxis am Ebertplatz, Cologne, Germany. ^9^Medizinisches Infektiologiezentrum Berlin, Berlin, Germany. ^10^Gemeinschaftspraxis, Schwabstrasse, Stuttgart, Germany. ^11^Mannheimer Onkologie Praxis, Mannheim, Germany. ^12^Department of Medicine I, University of Bonn, Bonn, Germany. ^13^Department of Medicine II, University of Schleswig‐Holstein, Kiel, Germany. ^14^Praxis, Hannover, Germany

**Background: **Deep sequencing (DS) assays may represent a reproducible approach to analyse HIV‐1 mutant spectra, even at variant frequencies well below those routinely detectable by population (Sanger) sequencing. DS data from viral reservoirs (i.e. peripheral blood mononuclear cells [PBMCs]) in patients with documented multiple viral drug resistance mutations (DRMs) and with long‐standing viral suppression are scarce.

**Material and methods**

This nationwide study included patients with a history of multi‐drug resistance (MDR) in 13 German HIV centres. MDR was defined to comprise at least one major DRM in at least three classes of NRTIs, NNRTIs and PIs or INSTIs. In patients with viral suppression (<50 HIV RNA copies/mL), DS was performed from proviral DNA. The number of individually detected mutations by DS was compared with all reported historical DRMs.

**Results: **Of a total of 243 patients with MDR, 187 (168 males and 19 females) had achieved viral suppression with various ART regimens and were included in the analysis. Median age was 55.4 years, and median time since first positive HIV test was 25.2 years (range 6.8 to 34.3 years). Median time of viral suppression was 8.5 years (range 0 to 18.6 years). Using a Sanger‐like cut‐off of 15% and a cut‐off of 2%, DS detected 47.2% and 64.7% of all historically reported DRMs, respectively. Using a DS cut‐off of 2%, the duration of viral suppression was not associated with detection rates of all documented DRMs, of DRMs of different drug classes or of major class‐specific DRMs such as M184V, L90M and K103N. Even in the subgroups of patients with continuous viral suppression of more than 10 or 15 years, detection rates of different DRMs were not lower than in patients with shorter periods of viral suppression. The same results were found when patients with transient viraemia were excluded.

**Conclusions: **In this large cohort study of HIV‐infected patients with documented extensive resistance, DS of proviral DNA detected the majority of mutations that had emerged at previous virological failures, even after a decade or more of ongoing viral suppression. DS sensitivity appears to be not affected by the duration of viral suppression.

## P297

### Factors associated with virological response and resistance profile in virologically suppressed HIV‐1 infected patients switching to a dual therapy containing integrase inhibitors in clinical practice

**D Armenia^1^, C Gori^2^, F Forbici^2^, V Borghi^3^, W Gennari^4^, A Bertoli^1^, A Giannetti^5^, S Cicalini^5^, A Mondi^5^, M Colafigli^6^, M Lichtner^7^, M Andreoni^8^, C Mussini^3^, A Antinori^5^, F Ceccherini Silberstein^1^, C Perno^2^ and M Santoro^1^**

^1^Department of Experimental Medicine and Surgery, University of Rome Tor Vergata, Rome, Italy. ^2^Antiretroviral Therapy Monitoring Unit, INMI L. Spallanzani, IRCSS, Rome, Italy. ^3^Clinic of Infectious Diseases, Polyclinic of Modena, Modena, Italy. ^4^Microbiology Laboratory, Polyclinic of Modena, Modena, Italy. ^5^Division of Infectious Diseases, INMI L. Spallanzani, IRCSS, Rome, Italy. ^6^Infectious Dermatology and Allergology Unit, IFO S. Gallicano Institute, IRCCS, Rome, Italy. ^7^Department of Public Health & Infectious Diseases, La Sapienza University Polo Pontino, Latina, Italy. ^8^Infectious Disease Division, Polyclinic of Rome Tor Vergata, Rome, Italy

**Background: **We evaluated the virological response and the resistance profile in virologically suppressed patients switching for the first time to a dual therapy based on integrase inhibitors (INI).

**Materials and methods: **Survival analysis was used to assess probability and predictors of virological failure (VF, ≥2 consecutive viraemia >200 copies/mL after switch). Drug resistance was evaluated before (as cumulative plasma resistance) and after switch. Cumulative genotypic susceptibility score for companion drugs (cGSS) was also evaluated (HIVdb algorithm v.8.4).

**Results: **Overall, 248 cART‐treated patients virologically suppressed from a median time of 1.9 (IQR 0.6 to 4.5) years and starting an INI‐based dual therapy in 2015 (IQR 2011 to 2016) were analysed. At switch, patients experienced a median number of 5 (IQR 2 to 9) previous regimens. One hundred and sixty‐six of 248 patients switched to raltegravir, administered mainly with darunavir (80, 48.2%) or etravirine (21, 58.8%). Eighty‐two of 248 patients switched to dolutegravir, administered mainly with lamivudine (46, 56.1%) or darunavir (22, 26.8%). Overall, 64.5% had ≥1 previous major resistance mutation (NRTI, 56.9%; NNRTI, 44.8%; PI, 27.4%; INI, 2.9%). cGSS revealed that 20.2% of patients harboured a virus with intermediate/full resistance to the companion drugs adminstered with INI. The overall probability of VF at 36 months after switch was 8.3% (median [IQR] viraemia at VF: 10,458 [1829 to 51,596] copies/mL). By stratifying for INI, the probability of VF with dolutegravir was 5.2%, while with raltegravir was 9.5% (*p* = 0.292). The duration of virological suppression before switch was strongly associated with probability of VF after switch (<1 year, 19.4%; one to three years, 1.8%; three to five years, 2.2%; >5 years, 0%; *p* < 0.001). Patients showing intermediate/fully resistant cGSS had a higher probability of VF compared to those with a fully susceptible cGSS (18.3% vs. 5.2%, *p* = 0.006). Multivariable Cox‐regression confirmed that factors negatively associated with VF were a longer time of previous suppression (adjusted relative hazard [95% CI] per 1 year higher, 0.18 [0.05 to 0.59], *p* = 0.005) and a fully susceptible cGSS (vs an intermediate/resistant cGSS, 0.08 [0.15 to 0.393], *p* = 0.002). Among patients experiencing VF, 12 patients were tested for resistance. INI resistance was detected in seven (58.3%) patients (six under raltegravir, one under dolutegravir); four (33.3%) patients (all receiving raltegravir) accumulated further major resistance mutations to companion drugs (PI, 33.3%; NRTI, 8.3%) (Table 1).

**Table:** Abstract P297 – Table 1. Overview of patients harboring resistance after virological failure to a dual therapy based on integrase inhibitors

IDa	Previous ARVs experienced	Time under VS before switch (months)	Baseline cGSS for companion drug	Drugs received at switch	Viremia at GRT (copies/mL)	Time under INI‐based dual therapy (months)	Resistance mutations detected after VF				
	No of ARV classes INI	INI						INI MRMs	INI accessory	PI MRM	NRTI MRM	NNRTI MRM
357^a^	6	RAL	7	F/I	DTG, DRV/r	297560	11.3	**Y143YCHR**	**T97TA**	None	None	None
326	5	None	37	F/I	RAL, ATV/r	1786	7.1	**G140S, Q148H**	None	L90M	M184V	None
1321	5	RAL	1	F/I	RAL, SQV/r	3356	19.5	**Y143YCHR**	T97A	V32I, L33F, M46I, I47V, **I50V,** I54L	K70R, K219Q	None
3507	5	RAL	1	F/I	RAL, LPV/r	7590	34.2	**N155H**	None	M46L, **I54V,** V82A	M41L, **D67G,** L210W, T215Y, K219E	None
7583	5	RAL	24	F/I	RAL, NVP	4627	2.1	**N155H**	None	**M46L, I84V**	M184V, T215Y	K103N, Y188L
10930	6	None	44	S	RAL, ATV/r	2709	11.6	None	**G163R**	None	None	None
11067	4	None	16	S	RAL, FPV/r	1045	10.9	**Y143S**	None	**L76V**	None	None

Among 12 patients with an available GRT after virological failure, 7 showed resistance. In bold are indicated mutations selected after virological failure. ^a^the patient was previously heavily treated and developed the mutation N155H after raltegravir failure. /r = ritonavir‐boosted; ATV = atazanavir; cGSS = cumulative genotypic susceptibility score for companion drugs; DRV = daraunavir; DTG = dolutegravir; F/I = fully/intermediate resistant cGSS; GRT = genotypic resistance test; INI = integrase inhibitor; LPV = lopinavir; MRM = major resistance mutation; NRTI = nucleos(t)ide reverse transcriptase‐inhibitor; NNRTI = non‐NRTI; PI = protease inhibitor; RAL = raltegravir; S = susceptible cGSS; SQV = saquinavir; VF = virological failure; VS = virological suppression.

**Conclusions: **In pluri‐treated virologically suppressed patients, switching to a dual therapy including dolutegravir or raltegravir ensures a high rate of virological control. Being in stable virological suppression for at least one year, and having INI associated with a fully active companion drug, are factors linked to a greater rate of success.

## P298

### High prevalence of previously undocumented baseline M184V/I does not affect virologic outcome in virologically‐suppressed patients switching to bictegravir/emtricitabine/tenofovir alafenamide from a boosted protease inhibitor‐based regimen

**K Andreatta^1^, R Haubrich^2^, M Willkom^1^, R Martin^1^, S Chang^1^, R Acosta^1^, Y Liu^3^, H Graham^4^, E Quirk^4^ and K White^1^**

^1^Clinical Virology, Gilead Sciences, Foster City, CA, USA. ^2^Medical Affairs, Gilead Sciences, Foster City, CA, USA. ^3^Biostatistics, Gilead Sciences, Foster City, CA, USA. ^4^Clinical Research, Gilead Sciences, Foster City, CA, USA

**Background: **Pre‐existing resistance can affect the efficacy of switching antiretrovirals in virologically‐suppressed HIV‐infected individuals, but may be difficult to detect. M184V/I resistance substitutions commonly develop during virologic failure with emtricitabine (FTC)‐ or lamivudine (3TC)‐containing regimens, and may be archived in the HIV‐1 reservoir after virologic resuppression. Study 380‐1878 demonstrated non‐inferior efficacy of switching stably suppressed HIV‐1‐infected adults without known FTC/3TC resistance to bictegravir/emtricitabine/tenofovir alafenamide (B/F/TAF) versus continuing boosted protease inhibitor (PI)‐based regimens (92.1% B/F/TAF vs. 88.9% continued PI with HIV‐1 RNA <50 copies/mL at Week [W] 48). We retrospectively investigated pre‐existing M184V/I resistance substitutions and impact on virologic outcomes.

**Materials and methods: **HIV‐1 suppressed participants switched to B/F/TAF at baseline (N = 290) or maintained suppressive PI‐based therapy (N = 287). Participants with known historical resistance to study medications were excluded. Retrospective HIV‐1 proviral DNA genotyping of baseline whole blood samples was performed (GenoSure Archive^®^ assay, Monogram Biosciences). Participants with M184V/I detected post‐randomization were not discontinued provided HIV‐1 RNA suppression <50 copies/mL was maintained.

**Results: **Altogether, baseline genotypic data were available for 96% (277/290) of B/F/TAF‐treated participants. Historical genotypes were available at screening for 49% (141/290) and showed no evidence of pre‐existing M184V/I. Baseline proviral DNA genotypes were obtained for 89% (259/290) and detected archived M184V/I in 16% (42/259). In 88% (37/42) of these participants, only the M184V substitution was observed; in 7% (3/42), only M184I was observed; and in 5% (2/42) both M184V and M184I substitutions were observed. Through W48, transient viremia (one HIV‐1 RNA >50 copies/mL measurement; blip) was observed in 4.8% (2/42) with M184V/I versus 5.5% (12/217) without M184V/I. Among participants with M184V/I, 95% (40/42) were suppressed at their last study visit at the time of the W48 Snapshot analysis: 37 had HIV‐1 RNA <50 copies/mL at W48 and three were missing W48 data, but had HIV‐1 RNA <50 copies/mL at their last study visit. Two participants with pre‐existing M184V/I discontinued before W48 coincident with poor study drug adherence and the last available HIV‐1 RNA ≥50 copies/mL. No B/F/TAF‐treated participant developed new study drug resistance.

**Conclusions: **In Study 380‐1878, a high frequency of unidentified baseline M184V/I was detected among suppressed patients switching from PI‐based regimens. Pre‐existing M184V/I did not affect HIV‐1 RNA suppression or viral blipping in B/F/TAF‐treated patients. High rates of virologic suppression through 48 weeks and the absence of treatment‐emergent resistance indicate that B/F/TAF is a treatment option for suppressed patients, including those with evidence of pre‐existing M184V/I.

## P299

### Predicting two‐drug antiretroviral regimen efficacy by genotypic susceptibility score: results from a cohort study

**B Rossetti^1^, D Redi^2^, A Ciccullo^3^, F Lombardi^3^, S Paolucci^4^, L Bellazzai^5^, A Di Biagio^6^, G Penco^7^, L Lepore^8^, L Monno^9^, S Rusconi^10^, T Carli^11^, S Modica^2^, R Gagliardini^2^, M Zazzi^2^ and A De Luca^2^**

^1^Infectious Diseases Unit, Siena University Hospital, Siena, Italy. ^2^Department of Medical Biotechnologies, University of Siena, Siena, Italy. ^3^Institute of Clinical Infectious Diseases, Catholic University of Sacred Heart, Policlinico Gemelli, Roma, Italy. ^4^Virology Unit, S. Matteo Hospital, Pavia, Italy. ^5^Infectious Diseases Unit, S. Matteo Hospital, Pavia, Italy. ^6^Infectious Diseases Unit, IRCCS S. Martino‐IST, Genova, Italy. ^7^Infectious Diseases Unit, Ospedali Galliera, Genova, Italy. ^8^Virology Unit, Bari Hospital, Bari, Italy. ^9^Infectious Diseases Unit, Bari Hospital, Bari, Italy. ^10^Infectious and Tropical Diseases Unit, DIBIC L. Sacco Hospital, University of Milano, Milano, Italy. ^11^Infectious Diseases Unit, Grosseto Hospital, Grosseto, Italy

**Background: **HIV drug resistance has a deleterious effect on the virological outcome of ART. The aim of the study is to evaluate the ability of genotypic susceptibility score (GSS) to predict virological outcome following an ART switch to a two‐drug regimen in pretreated HIV‐1 infected patients.

**Material and methods**

From the ARCA database we selected HIV‐1 infected treatment‐experienced patients switching to two‐drug ART (2007 to 2017, time of switch=baseline), with pre‐baseline resistance genotype and at least one HIV‐1 RNA determination during follow‐up. Primary endopoint was virological failure (VF, defined as an HIV‐RNA VL >400 copies/mL). Survival analysis was used to investigate predictors of VF. The GSS predicted by the latest and the cumulative genotype (CGSS), was calculated using the Stanford HIVdb (v.8.5) with respect to the two‐drug regimen started. Pre‐baseline viraemia copy‐years (VCY) were calculated using the trapezoidal rule on the VL log10 scale using all the available VL.

**Results: **We included 2149 patients. Overall 68% were males, 31% MSM, 6% non‐Caucasians, with median age of 50 years (IQR 45 to 56), 15 years of HIV history (8 to 22). At baseline 71% of patients had HIV‐1 RNA <50 copies/mL and their last VL >50 copies/mL was three years before baseline (1 to 6). Median GSS was 2 (1 to 2), with GSS <2 in 41% patients and CGSS 2 (1–2), with CGSS <2 in 50%. The estimated probability of VF at 48 weeks was 5.1% (95% CI 4–6.2) among patients with GSS=2, 6.8% (5.1 to 8.5) among patients with GSS 1 to 1.99 and 26.3% (17.6 to 35) among those with GSS <1 (log rank *p* < 0.001). According to CGSS, the estimated probability of VF at 48 weeks was 4.5% (95% CI 3.4 to 5.6) among patients with HGSS = 2, 7.2% (5.7 to 8.7) among patients with HGSS 1–1.99 and 13% (9 to 17) among those with HGSS <1 (log rank p=ns). At multivariate analysis adjusting for ARV regimen, VCY, CD4 cells count at nadir, GSS, only higher baseline VL  (+1 log aHR 1.81, 95% CI 1.38 to 2.38, *p* < 0.001) resulted to be associated with higher risk of VF, while GSS showed a trend (Table 1).

**Table:** Abstract P299 – Table 1. Predictors of virological failure by Cox regression

	Univariate analysis		Multivariate analysis	
	*p* value	HR (CI 95%)	*p* value	aHR (CI 95%)
3TC + bPI (ref)				
INSTI + bPI	0.02	2.72 (1.44 to 5.14)	0.35	1.81 (0.53 to 6.23)
NRTI + INSTI	0.46	0.63 (0.18 to 2.18)	0.70	0.65 (0.07 to 6.00)
NNRTI + INSTI	<0.001	3.80 (0.18 to 2.18)	0.25	2.43 (0.54 to 11.00)
bPI + MVC	0.54	0.68 (0.19 to 2.35)	0.47	1.96 (0.32 to 11.96)
NNRTI + bPI	0.07	2.14 (0.94 to 4.9)	0.81	1.18 (0.30 to 4.69)
VCY 1° quartile (ref)				
VCY 2° quartile	0.25	1.88 (0.64 to 5.51)	0.44	1.96 (0.35 to 10.91)
VCY 3° quartile	0.33	1.72 (0.57 to 5.14)	0.62	1.53 (0.28 to 8.34)
VCY 4° quartile	0.28	3.11 (1.13 to 8.56)	0.20	2.81 (0.58 to 13.63)
Nadir CD4 cell count (per 100 cells/mm^3^ increase)	0.06	0.84 (0.70 to 1.00)	0.38	0.85 (0.59 to 1.22)
Baseline HIV‐1 RNA (per 1 log10 increase)	<0.001	1.95 (1.67 to 2.26)	<0.001	1.81 (1.38 to 2.38)
GSS <1 (ref)				
GSS 1 to 1.99	0.01	0.24 (0.10 to 0.58)	0.09	0.25 (0.05 to 1.22)
GSS=2	<0.001	0.20 (0.09 to 0.47)	0.19	0.39 (0.09 to 1.61)

**Conclusions: **Viral load at switch and the presence of less than one fully active drug strongly influence virological efficacy of two‐drug regimen switches.

## P300

### Prevalence of integrase inhibitor resistance mutation in patients with therapeutic failure

**I Viciana^1^, C Garcia‐Perez^1^, C Gonzalez‐Domenech^2^, C Gomez‐Ayerbe^2^, P Bardon^1^, R Palacios^3^, M Castaño^4^, A Del Arco^5^, F Tellez^6^, E Clavijo^1^ and J Santos^3^**

^1^Microbiology, Hospital Virgen de la Victoria, Malaga, Spain. ^2^FIMABIS, Hospital Virgen de la Victoria, Malaga, Spain. ^3^Infectious Disease, Hospital Virgen de la Victoria, Malaga, Spain. ^4^Infectious Disease, Hospital Regional, Malaga, Spain. ^5^Infectious Disease, Hospital Costa del Sol, Malaga, Spain. ^6^Infectious Disease, Hospital Puerto Real, Cadiz, Spain

**Objectives/background**

First‐generation integrase inhibitors (INI), raltegravir (RTG) and elvitegravir (EVG) have a low genetic barrier and broad cross‐resistance among them. Dolutegravir (DTG) has a higher genetic barrier and isolates resistant to RTG and EVG remains sensitive to it. The aim of this study was to identify the most frequent resistance mutation patterns selected at the virological failure (VF) with a regimen including INI, as well as the susceptibility to the drugs of this family.

**Methods: **We analysed the integrase resistance studies from patients with VF during April 2012–June 2018, performed in our hospital, a reference centre in southern Spain (Viroseq HIV‐1 Integrase System. Interpretation: Stanford algorithm v.7.1).

**Results: **A total of 236 patients in VF with a regimen with INI were included. The characteristics at failure were the following: 176 (74.6%) men with a median age of 49 years (IQR 16 to 72), median T‐lymphocyte CD4 count 363 cells/uL (IQR 6 to 1926) and viral load 3.34 log copies/mL (IQR 2.01 to 6.22). One hundred and ninety‐nine patients (84.3%) carried a subtype B virus. Sixty‐eight (28.8%) cases were in first failure, 151 (64%) two or multiple failures and 17 (7.2%) discontinued the treatment. One hundred and thirty‐eight (58.5%) regimens included RTG, 37 (15.7%) EVG and 61 (25.8%) DTG. **Failing with RTG**: 27 first failure (19.6%), 104 (75.4%) with ≥2 failures and seven (5.1%) treatment discontinuation. Forty‐three (31.2%) patients selected resistance mutations. Most frequent patterns: 140ACS+148H (seven), 138K+148H (four), 155H plus other mutations (15). Resistance to RTG, 29.7%; resistance to EVG, 27.5%; resistance to DTG, 9.4%. **Failing with EVG**: 21 (56.8%) first failure, 13 (35.1%) second failure or higher and three (8.1%) treatment interruption. Twelve (32.4%) patients selected resistance mutations. Most frequent patterns: 92Q+97A (four), 92Q plus other mutations (five). Resistance to RTG, 29.7%; resistance to EVG, 29.7%; resistance to DTG, 2.7%. **Failing with DTG**: first failure, 20 (32.8%), 34 (55.7%) second or higher failure and seven (11.5%) interrupted treatment. Nine (14.7%) patients selected resistance mutations. Most frequent patterns: 263K plus other mutations (three), 138K+148H (two), 155H (one). Resistance to RTG, 14.6%; resistance to EVG, 14.6%; resistance to DTG, 9.9%.

**Conclusions: **Over a quarter of patients in VF selected resistance mutations to integrase. The most frequently selected were 140ACS+148H, 92Q+97A and 263K plus other mutations for patients treated with RTG, EVG and RTG respectively. Almost three out of four patients presented resistance to RTG and EVG whereas resistance to DTG did not reach 10%. DTG continues maintaining activity against many of the isolates resistant to first‐generation INI.

## P301

### Gp120 substitutions at “hot” positions associated with resistance to fostemsavir in naïve HIV‐1 positive individuals

**L Lepore^1^, C Fabrizio^1^, E Milano^1^, N de Gennaro^1^, A Lagioia^1^, A Volpe^1^, L Scudeller^2^, A Saracino^1^, G Angarano^1^ and L Monno^1^**

^1^Clinic of Infectious Diseases, University of Bari, Bari, Italy. ^2^Clinical Epidemiology Unit, IRCCS San Matteo Foundation, Pavia, Italy

**Background: **Fostemsavir (FTR) is a novel entry inhibitor targeting the HIV‐1 gp120. In vitro activity was demonstrated regardless of viral tropism and subtype, except for the CRF01_AE. However, the high rate of HIV gp120 substitutions would jeopardise the efficacy of FTR. In the present study we investigated the presence of *env* substitutions at “hot” positions for resistance to FTR in patients with new HIV diagnosis according to HIV subtype and tropism (CRT).

**Material and methods**

Gp120 sequences from 409 subjects with new HIV diagnosis were retrospectively analysed. Clinical isolates were classified as either B and non‐B subtypes (REGA‐3 system); CRT was inferred with the g2p algorithm (FPR 10%) and duration of HIV infection estimated based on the proportion of ambiguous nucleotides in RT/PR (e.g. ≤0.2%: recent infection). The frequency of the following substitutions was evaluated: L116P/Q (325 sequences); A204D (370 seq); S375H/M/T (382 seq); M426L (282 seq); M434I (238 seq); M475I (112 seq), including other amino acid changes. For B strains, Entropy‐2 was used to compare variability at each amino acid position between R5 and non‐R5 viruses.

**Results: **Features of the study population are summarised in Table 1. Observed mutations were: S375T (13.0%); M426L (6.74%); M434I (2.94%); M475I (2.68%); S375H (1.57%)/M (0.79%) and L116P (0.31%). A statistically significant difference in R5 versus non‐R5 variants and B versus non B subtypes was found at positions 375 (p = 0.014 and p < 0.001, respectively) and 426 (p < 0.001) (Table 2). A post hoc analysis (significant at *p* < 0.025) revealed that significance for position 375 was steered by S375T (R5 vs non‐R5, *p* = 0.023 and B/non‐B, *p* < 0.0001) and showed a trend for position 426 (B/non‐B, *p* = 0.028). Variability of assessed *env* constant domains seemed to be more relevant in the non‐R5 virus population.

**Table:** **Abstract P301 –** Table 2. Frequency of gp120 mutations according to CRT and subtype

Substitution	R5	Non‐R5	*p* value	B	Non‐B	*p* value
L116 wt	261 (98.1)	58 (98.3)	0.99	208 (98.6)	111 (97.3)	0.58
L116P	1 (0.37)	0 (0.0)		1 (0.47)	0 (0.0)
L116 other	4 (1.5)	1 (1.7)		2 (0.93)	3 (2.7)
S375 wt	247 (80.4)	52 (69.3)	0.014	175 (72.0)	124 (89.2)	<0.001
S375 H/M/T	39 (12.7)	20 (26.7)		50 (20.6)	9 (6.5)
S375 other	21 (6.8)	3 (4.0)		18 (7.4)	6 (4.3)
M426 wt	171 (72.2)	26 (57.8)	0.10	104 (57.5)	93 (92.1)	<0.001
M426L	16 (6.8)	3 (6.7)		15 (8.3)	4 (4.0)
M426 other	50 (21.1)	16 (35.6)		62 (34.3)	4 (4.0)
M434 wt	185 (94.2)	40 (95.2)	0.79	149 (94.9)	76 (93.8)	0.73
M434I	6 (3.1)	1 (2.4)		5 (3.2)	2 (2.5)
M434 other	5 (2.6)	1 (2.4)		3 (1.9)	3 (3.7)
M475 wt	92 (95.8)	16 (100)	1.0	65 (98.5)	43 (93.5)	0.16
M475I	3 (3.1)	0 (0.0)		1 (1.5)	2 (4.3)
M475L	1 (1.0)	0 (0.0)		0 (0.0)	1 (2.2)

Results are presented as number (percentage) of strains. wt = wild type.

**Table:** Abstract P301 – Table 1. Baseline characteristics of the enrolled patients (N = 409)

Age, median (range)	35.5 years (IQR 27.8 to 44.8)
Male, N (%)	346 (84.6%)
Italians, N (%)	357 (87.3%)
Infection <1 year, N (%)	188 (46%)
Acute infection, N (%)	23 (5.6%)
Risk factor for HIV transmission ‐ sexual route, N (%)	368 (90.1%)
Risk factor for HIV transmission ‐ MSM, N (%)	213 (52%)
Baseline CD4 cell count, cell/mm^3^ (range)	364.0 (IQR 162.0 to 510.0)
Baseline log10 HIV‐RNA, median (range)	4.72 (IQR 4.07 to 5.32)
Coreceptor tropism R5, N (%)	331 (81%)
Non‐B strains, N (%)	149 (36.4%)
CRF01_AE, N (%)	4 (1%)

**Conclusions: **Gp120 substitutions that may prejudice susceptibility to FTR were detected in different subtypes and in both R5 and non‐R5 variants. Nevertheless, despite the great variability of gp120, the frequency of mutations was overall low. Furthermore, the predominant mutation among B subtypes was S375T, whose role in reducing FTR efficacy is much less substantial than L116P, S375H and M426L, which, conversely, were detected in a smaller proportion of subjects. Based on these premises, we believe that FTR can be considered a promising therapeutic option for a large target of patients. It would be worthwhile to investigate additional mutations with the potential to reduce FTR efficacy in a larger scale.

## P302

### Molecular epidemiology of HIV‐1 in South Russia and neighbouring regions

**M Schlösser^1^, V Kartashev^2^, V Mikkola^1^, A Shemsura^3^, D Kolpakov^2^, A Suladze^2^, T Tverdokhlebova^2^, S Saukhat^4^, R Kaiser^1^, E Heger^1^, E Knops^1^, M Böhm^1^, K Hutt^1^ and S Sierra^1^**

^1^Institut für Virologie, University of Cologne, Köln, Germany. ^2^Russian Southern Federal Center for HIV Control, Rostov‐na‐Donu, Russian Federation. ^3^Krasnodar Clinical Center for HIV Control, Krasnodar, Russian Federation. ^4^Rostov State Medical University, Rostov‐na‐Donu, Russian Federation

**Background: **In East Europe and Central Asia HIV‐1 infection rate has risen by 57% in 2000 to 2015, with currently 1.5 million HIV‐infected people, whereof 1.16 million live in Russia. Russia shows an HIV‐1 landscape with predominant subtype A1/A6 throughout the land and a subtype G cohort in the South. Resistance testing at baseline and after failure of ART is not routinely performed. Epidemiology and resistance data are still limited and not accessible through international databases.

**Materials and methods: **Three hundred and twenty‐eight patients from 11 regions were included, mainly from South Russia (65.2%) and from North Caucasus (9.1%). For 79 (24.1%) patients place of residence was unknown. Blood samples and epidemiological data were collected within the Rostov‐on‐Don/Cologne cooperation project (ethics approval #246), RT‐PCR for the protease‐reverse transcriptase (PRRT) and integrase (IN) was performed and amplicons were sequenced with the next‐generation sequencing Illumina MiSeq platform (cut‐off 10%). Subtyping and resistance‐associated mutations (RAMs) analysis were performed using the geno2pheno[resistance] and Stanford HIVdb PROGRAM tools (http://www.geno2pheno.org/; http://https://hivdb.stanford.edu/hivdb/by‐mutations/).

**Results: **Three hundred patients could be analysed. One hundred and twenty‐six (42%) were female, 147 (49%) male and 27 (9%) unknown. The most frequent transmission route was PWID (n = 97, 32.3%), followed by heterosexual (n = 81, 27%) and nosocomial (n = 72, 24%). Males were significantly more often infected through drug consume, whereas females via heterosexual contact. Most common subtype in all different transmission route groups (except nosocomial) was A1/A6 (71%), followed by G (22.6%, almost exclusively in nosocomial transmission). Subtype B was significantly associated to infections in MSM. Two hundred and forty‐eight PR sequences were obtained, where 228 (91.9%) displayed no major PI‐RAMs and 20 (8.1%) showed 1 to 5 major mutations. Most frequently found RAMs were M46I, I54V and L90M (Table 1). Two hundred and seventy‐three RT sequences were analysed: 147 (53.8%) displayed no NRTI‐RAMs and 126 (46.2%) samples showed 1 to 7 mutations, with A62V and M184V being the most highly prevalent. One hundred and ninety‐eight RT samples (72.5%) contained no NNRTI mutations and 75 (27.5%) showed 1 to 5 NNRTI‐RAMs; K103N, E138A and G190S were most commonly found.

**Conclusions: **HIV‐1 subtype distribution in Russia is different to West and Central Europe, with predominant subtype A1/A6 throughout the land and subtype G cohort in the South. In addition, the proportion of PR sequences displaying mayor PI‐RAMs in our study is similar to that described for West and Central Europe therapy‐experienced patients, while the prevalence of NRTI‐ and NNRTI‐RAMs is larger in Russia than in Western Europe. Efforts to increase the implementation of routine HIV‐1 resistance testing will improve therapy efficacy and Russian epidemiology knowledge.

**Table:** Abstract P302 – Table 1. Resistance‐associated mutations (RAMs) detected

Pls	N	%	NRTIs	N	%	NNRTIs	N	%
D30N	1	2.4	M41L	21	8.8	A98G	2	1.7
M46I	8	19.5	A62V	61	25.4	K101E	8	6.8
M46L	3	7.3	D67G	2	0.8	K101HQ	1	0.8
G48V	2	4.9	D67N	15	6.3	K103N	26	22.0
I50V	1	2.4	K65DEN	1	0.4	K103S	1	0.8
I50L	2	4.9	K65E	1	0.4	L100F	2	1.7
I54A	1	2.4	T69DN	1	0.4	V106A	1	0.8
I54V	5	12.2	T69G_SG	1	0.4	V108I	6	5.1
L76V	2	4.9	K70E	1	0.4	E138A	14	11.9
V82S	1	2.4	K70R	12	5.0	E138G	1	0.8
V82M	2	4.9	L210W	7	2.9	E138GQR	1	0.8
V82A	1	2.4	L74I	1	0.4	E138K	2	1.7
V82T	2	4.9	L74V	7	2.9	V179D	2	1.7
V82F	2	4.9	V75A	1	0.4	V179E	4	3.4
N88S	1	2.4	V75I	1	0.4	Y181C	10	8.5
L90M	7	17.1	V75M	1	0.4	Y181F	1	0.8
			Y115F	2	0.8	Y181I	1	0.8
			M184I	1	0.4	Y181V	1	0.8
			M184IV	1	0.4	Y188L	2	1.7
			M184V	56	23.3	Y188S	1	0.8
			T215C	1	0.4	G190A	4	3.4
			T215f	9	3.8	G190S	17	14.4
			T215I	1	0.4	H221Y	2	1.7
			T215L	2	0.8	P225H	4	3.4
			T215NSY	2	0.8	F227L	1	0.8
			T215S	1	0.4	M230L	1	0.8
			T215Y	16	6.7	K238T	1	0.8
			K219E	6	2.5	Y318F	1	0.8
			K219EQ	1	0.4			
			K219N	1	0.4			
			K219Q	6	2.5			

## P303

### Clinical impact of virological failure and resistance analysis definitions used in pivotal clinical trials of initial antiretroviral treatment: a systematic review

**H Álvarez^1^, M Yzusqui^2^ and J Llibre^3^**

^1^Infectious Diseases Unit, University Hospital of Ferrol, Internal Medicine Department, Ferrol, Spain. ^2^Internal Medicine Department, Hospital Nuestra Señora del Prado, Talavera de la Reina, Spain. ^3^Infectious Diseases, University Hospital Germans Trias i Pujol, Badalona, Spain

**Background: **There are no standardised criteria for defining confirmed virological failure (PDVF) nor the inclusion criteria for the resistance analysis population (RAP) in Phase III randomised clinical trials (RCTs) of initial ART. We assessed the clinical impact of mismatching between virological non‐response (HIV‐1 RNA ≥50 copies/mL), confirmed PDVF and RAP definition in studies with the newest first‐line ART, at 48 weeks.

**Methods: **Systematic review of all Phase III RCTs including preferred once‐daily ART (European AIDS guidelines) or recently approved by the Food and Drug Administration.

**Results: **We identified 16 treatment arms (14 RCTs) with 6175 participants treated with dolutegravir, bictegravir, elvitegravir/cobicistat, raltegravir, darunavir/cobicistat, rilpivirine or doravirine. Plasma HIV‐1 RNA thresholds for PDVF or RAP ranged from 40 to 50, 200, 400 and 500 copies/mL. This led to discrepancies between trials regarding the participants defined as virological non‐responders, PDVF or included in RAP. Overall, 85/296 (29%) patients with PDVF were not genotyped. We found a strong evidence of a linear correlation between the higher HIV‐1 RNA threshold for genotyping and increasing rates of participants with PDVF that were not eventually genotyped: <50 copies/mL 0/110 (0%), <200 copies/mL 13/32 (40.6%), <400 copies/mL 50/118 (42.4%) and <500 copies/mL 22/36 (61.1%), *p* < 0.001 (Cochran‐Armitage test). Only eight treatment arms genotyped all participants with PDVF. Most of the remaining eight arms genotyped roughly <50% of those with PDVF. No resistance was selected against the third drug or the backbone NRTIs in any participant in the studies with dolutegravir (n = 1955 participants), bictegravir (n = 634) or darunavir/cobicistat (n = 362). Elvitegravir/cobicistat, raltegravir and rilpivirine showed selection of HIV‐1 resistance against both the third drug and the NRTIs used in the backbone in approximately 50% of the participants with PDVF and genotypes successfully performed. Percentages of participants with drug resistance mutations selected at virological failure, and participants meeting PDVF criteria but with no genotype data available (not genotyped for HIV resistance or failed amplification) at 48 weeks in the RCTs analysed, are shown in Figure 1.

**Abstract P303 – Figure 1.** Percentages of participants with drug resistance mutations selected at virological failure, and participants meeting PDVF criteria but with no genotype data available (not genotyped for HIV resistance or failed amplification) at 48 weeks in the RCTs analysed.

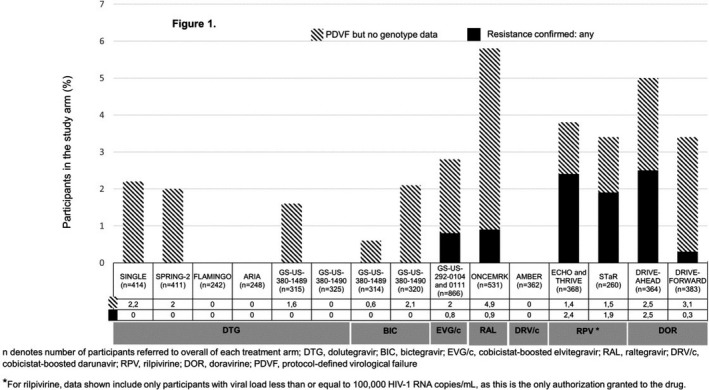

**Conclusions: **The absence of standardised definitions of VF and criteria for resistance testing in pivotal Phase III RCTs of first‐line ART leads to the possibility of underreporting of resistance mutations when genotypes are only performed at higher viral load cut‐offs. Stringent homogeneous criteria should be defined to ensure that all participants with PDVF (confirmed HIV RNA >50 copies/mL and the second >200 copies/mL) undergo genotyping.

## P304

### Impact of NRTI mutations on virological efficacy of antiretroviral regimens containing elvitegravir: an ARCA‐ECCO cohort study

**R Gagliardini^1^, S Modica^1^, D Redi^1^, E Giombini^2^, A Bezenchek^3^, D Di Carlo^4^, F Maggiolo^5^, F Lombardi^6^, A Borghetti^6^, A Callegaro^7^, M Gismondo^8^, M Colafigli^9^, G Sterrantino^10^, A Costantini^11^, S Ferrara^12^, S Rusconi^13^, S Di Giambenedetto^6^, M Zazzi^1^, A De Luca^1^, B Rossetti^14^ and N Gianotti^15^**

^1^Department of Medical Biotechnologies, University of Siena, Siena, Italy. ^2^National Institute for Infectious Diseases L. Spallanzani, Spallanzani, Rome, Italy. ^3^InformaPRO, Rome, Italy. ^4^Pediatric Clinical Research Center, University of Milan, Milan, Italy. ^5^Infectious Diseases Unit, Bergamo Hospital, Bergamo, Italy. ^6^Clinic of Infectious Diseases, Catholic University of Sacred Heart, Rome, Italy. ^7^Microbiology and Virology Unit, Bergamo Hospital, Bergamo, Italy. ^8^Microbiology Unit, Ospedale L. Sacco, Milan, Italy. ^9^STI/HIV Unit, IRCCS Rome, San Gallicano Dermatologic Institute, Rome, Italy. ^10^Division of Tropical and Infectious Diseases, Careggi Hospital, Florence, Italy. ^11^Clinical Immunology Unit, Università Politecnica delle Marche, Ancona, Italy. ^12^Clinic of Infectious Diseases, Azienda Ospedaliera‐Universitaria Ospedali Riuniti, Foggia, Italy. ^13^Infectious and Tropical Diseases Unit, DIBIC L. Sacco Hospital, Milano, Italy. ^14^Infectious Diseases Unit, AOU Senese, Siena, Italy. ^15^Infectious Diseases, IRCCS San Raffaele, Milano, Italy

**Background: **Integrase inhibitor‐based regimens are recommended by current guidelines as first‐choice ARV therapy. ARV drug resistance mutations remain a major cause of treatment failure. The aim of study was to evaluate the effect of mutations on virological efficacy of elvitegravir‐containing ARV regimens in naïve and treatment‐experienced HIV‐1 patients in a real‐life setting.

**Material and methods**

From the ARCA and ECCO databases we selected naïve and treatment‐experienced HIV‐1 infected patients starting tenofovir disoproxil fumarate or alafenamide/emtricitabine/elvitegravir/cobicistat (from June 2012 to December 2017), with baseline PR/RT resistance genotype and at least one HIV‐1 RNA during follow‐up. NRTI resistance mutations were defined as the detection of at least one mutation according to Stanford algorithm. Primary endpoint was virological failure (VF, defined as an HIV‐RNA VL >1000 copies/mL or two consecutive values of >50 copies/mL after Week 24 for treatment‐experienced with baseline VL >50 copies/mL and at any time for treatment‐experienced with baseline values of <50 copies/mL). Cox survival analysis was used to investigate predictors of VF.

**Results: **We included 282 patients: 16% naïve, 70% males, 29% heterosexuals, median age 47 years (IQR 37 to 53), 10 years of HIV (3 to 19), nine years of ART (4 to 17), nadir CD4+ 177 cells/µL (58 to 331), peak VL 147,840 copies/mL (38,779 to 500,000), 62% had VL <50 copies/mL, 16% presented at least one NRTI mutation, 16% at least one mutation among M184V/I or K65R or TAM (Table 1). During a median observation time of 8 months (4 to 17), 33 VF occurred (two in naïve patients, 31 in experienced). Among the experienced, the estimated probability of being free from VF at 12 months was 89% (95% CI 77 to 101) in patients with any NRTI major mutation versus 89% (84 to 94) among those without (log rank 0.478). Virological outcome stratified by NRTI mutation is shown in Figure 1. At multivariate analysis adjusting for any NRTI mutation and HCV serostatus, a longer duration of HIV infection (per one year longer aHR 1.05, 0.99 to 1.09, *p* = 0.063), higher peak VL (+ 1 log aHR 1.42, 0.97 to 2.08, *p* = 0.069) and VL >50 copies/mL (aHR 2.73, 0.95 to 7.81, *p* = 0.062) showed a trend with higher risk of VF. In the naïve group, 1/3 patients with any NRTI mutation had VF versus 1/43 without NRTI mutation (*p* < 0.001).

**Table:** Abstract P304 – Table 1. Prevalence of NRTI mutations at baseline

NRTI mutation, n (%)	Overall (n = 282)	Naïve (n = 46)	Experienced (n = 236)
M41L	1 (0.3%)	1 (2%)	0 (0%)
D67N	5 (1.8%)	2 (4%)	3 (1.3%)
K70R	9 (3%)	0 (0%)	9 (3.8%)
L210W	11 (4%)	2 (4%)	9 (3.8%)
T215Y/F	14 (5%)	0 (0%)	14 (6%)
K219Q/E	10 (4%)	0 (0%)	10 (4%)
L74V	2 (0.7%)	0 (0%)	2 (0.8%)
Y115F	3 (1%)	0 (0%)	0 (0%)
M184V/I	32 (11%)	1 (2%)	31 (13%)
K65R	1 (0.3%)	0 (0%)	1 (0.4%)
M184 + K65R	1 (0.3%)	0 (0%)	1 (0.4%)
Any TAM	28 (10%)	2 (4%)	26 (11%)
Any mutation except M184V/I	31 (11%)	2 (4%)	29 (12%)
At least one among M184V/I or K65R or TAM	45 (16%)	3 (6.5%)	42 (18%)
Any mutation	46 (16%)	3 (6.5%)	43 (18%)

**Abstract P304 – Figure 1.** Virological outcome stratified by baseline NRTI mutations for experienced patients.

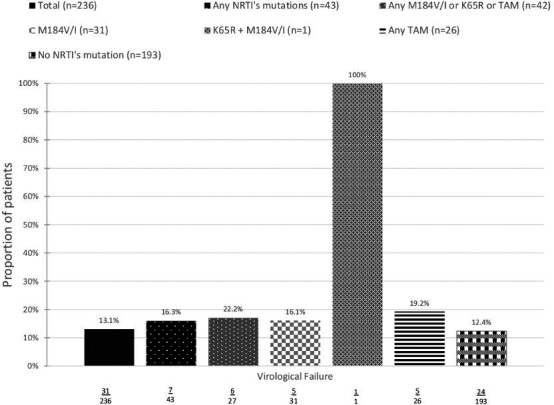

**Conclusions: **Elvitegravir‐containing ARV regimens resulted in a good rate of virological suppression, regardless of the presence of pre‐existing resistance mutations. In naïve patients it seems prudent to consider the presence of transmitted NRTI resistance.

## P305

### Resistance mutations to protease inhibitors in proviral DNA of HIV‐2 infected patients predict response to treatment

**F Martin^1^, A Martins^1^, F Maia^1^, C Rocha^1^, P Borrego^1^, F Antunes^2^, L Caldeira^2^, E Valadas^2^ and N Taveira^1^**

^1^Research Institute for Medicines (iMed.ULisboa), Faculty of Pharmacy, Universidade de Lisboa, Lisbon, Portugal. ^2^Serviço de Doenças Infecciosas, Faculdade de Medicina de Lisboa, Hospital de Santa Maria, Lisboa, Portugal

**Background: **Compared to HIV‐1, data on the diversity of the HIV‐2 protease (PR) gene and evolution of resistance to protease inhibitors (PIs) are limited. Herein, we make the characterisation of PR diversity and resistance to PIs in proviral DNA of HIV‐2 infected individuals using clonal sequencing.

**Materials and methods: **The PR gene was amplified from proviral DNA present in PBMCs from 27 HIV‐2 infected patients attending a central hospital in Lisbon, Portugal. Fifteen were on treatment and 12 were untreated. PR diversity was analysed by phylogenetic and entropy analysis. PI resistance mutations were identified using EU HIV‐2 internet tool HIV‐GRADE. The treatment outcomes and resistance mutations of all patients were analysed eight years after enrolment.

**Results: **In total, 187 clonal sequences were generated, 91 from PI‐treated patients and 96 from untreated patients. PR mutations associated with resistance to the most potent PIs, DRV, LPV, SQV, were detected in 42.8% treated patients. The most common resistance mutations in this subgroup of patients were L90M (n = 3, 21.4%) and I84V (n = 2, 14.2%). Other well‐known resistance mutations were I54M (n = 2, 14.2%) and I82F (n = 2, 14.2%). Importantly, we found major resistance mutations to PIs in 15.4% untreated individuals, indicating potential cases of transmitted drug resistance. Additionally, we found that 80% (4/5) of treated patients who presented at least one of these PI resistance mutations (I54M, I82F, L90M, I84V) experienced virological failure during the study period. After eight years of follow‐up, PR genetic diversity increased in two treated patients, with undetectable viral loads and high T CD4+ counts, indicating persistent viral replication during long‐term HAART, regardless of plasma viral load. Entropy analyses of PR identified three PI resistance associated positions (84, 90, 99) with significant higher entropy levels in treated group of patients compared with untreated group (0.51 vs. 0.063, 0.44 vs. 0.063 and 0.347 vs. 0.0, respectively). Furthermore, we found that 65% of the amino acid positions in PR that vary significantly between treated and untreated groups were located within some of the best‐characterised CTL epitopes described for HIV‐1, suggesting a potential interaction between PI treatment and CTL immune response in HIV‐2, similar to that described for HIV‐1.

**Conclusions: **Our results suggest that proviral DNA is a good alternative to genomic RNA for testing for drug resistance mutations in HIV‐2 infected patients and indicate that early resistance analysis of the viruses archived in PBMCs predict treatment response particularly at low or undetectable viral loads.

## P306

### Evaluation of the application of HIV‐1 genotypic drug resistance in treatment‐naïve patients in Taiwan where single‐tablet regimens are used as the first‐line regimens

**S Chang^1^, P Lin^1^, S Chang^1^, L Su^1^, Y Su^2^, W Liu^2^ and C Hung^2^**

^1^Clinical Laboratory Sciences/Medical Biotechnology, National Taiwan University, Taipei, Taiwan. ^2^Internal Medicine, National Taiwan University Hospital, Taipei, Taiwan

**Background: **According to the national HIV treatment guidelines, four single‐tablet regimens (STR), TDF/FTC/EFV, TDF/FTC/RPV, ABC/3TC/GTD and TAF/FTC/Cobi/EVG, are recommended as the first‐line regimens in the antiretroviral‐naïve HIV‐1‐infected patients in Taiwan since September 2016. A multicentre surveillance study of HIV‐1 genotypic resistance among antiretroviral‐naive patients in Taiwan was conducted to determine the prevalence of transmitted drug resistance (TDR) and to evaluate whether the pre‐treatment drug resistance testing is required in treatment‐naïve patients who will receive STR as the first‐line regimen.

**Methods: **Genotypic resistance assays were performed among the HIV strains from antiretroviral‐naïve patients receiving HIV care in the designated hospitals around Taiwan from June 2012 to May 2018. Resistance mutations were identified using the HIVdb programme of the Stanford University HIV Drug Resistance Database.

**Results: **Of the analysed 3987 blood specimens from treatment‐naïve patients, the overall prevalence of TDR to any drug was 13.6% (n = 543), which included 3.93% (n = 157), 8.85% (n = 353), and 2.33% (n = 93) with resistance‐associated mutations (RAMs) to NRTIs, nNRTIs, protease inhibitors (PIs), respectively; and 1.48% (n = 59) were multi‐drug resistance. Among these patients, 1343 patients underwent genotypic resistance testing for integrase inhibitors (INSTIs) and the prevalence of TDR was 2.31% (n = 31). The changes of TDR prevalence before and after September 2016 were compared. The prevalence of TDR to any class of drugs (12.84% vs. 16.18%, *p* = 0.009), NRTIs (3.47% vs. 5.47%, *p* = 0.006) and nNRTIs (7.96% vs. 11.79%, *p* = 0.0003) increased significantly after September 2016, while the prevalence of TDR to PIs (2.65% vs. 1.29%, *p* = 0.015) decreased significantly. The prevalence of TDR to INSTIs (2.81% vs. 1.43%) decreased and that to MDR (1.41% vs. 1.71%) increased without reaching statistical significance. There is no change in the most common RAMs to NRTIs and NRTIs between the two study periods. Nevertheless, more major INSTI‐related RAMs were observed in the first period, including five Q148R, five N155S, two E92Q and one Y143R; yet after September 2016, only one E92Q and one N155S major mutations were observed.

**Conclusions: **In Taiwan, the prevalence of TDR to nNRTIs increases significantly to 11.79% recently, which is above the threshold of the TDR prevalence recommended for pre‐treatment drug resistance testing. The prevalence of TDR to INSTIs remains low (1.43%). It is recommended that for those treatment‐naïve patients who choose nNRTI‐based STR as the first‐line regimen, a drug resistance testing should be performed, while drug resistance test is currently not required for those who choose INSTI‐based STR as the first‐line regimen in Taiwan.

## P307

### Next‐generation sequencing (NGS) in routine HIV‐1 resistance diagnostic: frequency of additional resistance‐relevant mutations in 2% and 1% population proportions correlated to viral load and additional patient follow‐ups

**R Ehret, A Moritz, S Breuerand M Obermeier**

Medical Laboratory Berlin, Berlin, Germany

**Background: **The relevance of mutations detected by NGS technologies in low frequencies is still a subject of debate. Clinical data are rare. We here report the frequency of additional mutations in population‐proportions of greater than 2% and 1% in routine laboratory testing and correlate them to viral load. Therapy implications for patients with relevant minor viral populations were monitored.

**Materials and methods: **Six hundred and forty‐five HIV‐1 resistance tests (reverse transcriptase/protease) performed between October 2014 and April 2016 with an in‐house PCR followed by NGS (Illumina MiSeq, sequences reported with >100 reads only) were analysed. Sequences were interpreted by HIV‐GRADE (http://www.hiv‐grade.de) for resistance mutations using 10%, 2% and 1% minority cut‐offs. A specific focus laid on differences in reported resistance‐associated mutations and resistance levels (e.g. additional drug class or further drugs same class). The proportion of subpopulations harbouring additional mutations with greater than 2000 copies/mL (= mutational load) were calculated, therapy data and follow‐up for those patients was monitored as far as available.

**Results: **Four hundred and eighty‐three (74.9%) samples were identified as subtype B. No drug resistance‐associated mutations were reported by HIV‐GRADE for 44% with a 10% cut‐off, 29.5% and 19.7% with 2% and 1% respectively. This also correlates with an increase of resistance level in the interpretation, especially for NNRTIs. With a cut‐off of 10% in 148 samples (105 non‐B subtype) only PI relevant mutations were detected. We found mutations only relevant for NRTIs in 21 samples and for NNRTI in 100 samples. Additional mutations could be detected in 94 of the samples using a 2% minority cut‐off. This corresponds to an additional mutational load of >2000 copies/mL in 76 cases with a 2% minority cut‐off and additional 134 mutations at 1% cut‐off. Consequences on treatment concepts and regimen were found rarely in therapy data.

**Conclusions: **A relative high proportion (56%) of investigated sequences showed resistance mutations at a minority cut‐off of 10% increasing substantially lowering the cut‐off range to 2% or 1% in number of mutations and also regarding resistance levels. Relevance of mutations in these low percentages is often discussed. The concept of “mutational load” tries to correlate the viral load with the proportion of mutation in the whole viral population. Despite the low percentage these viral quasispecies can be detected in relevant absolute quantities which increases the probability of detecting viable resistant virus. There is a clear need for clinical evaluation of the relevance of mutations in the low percentage range for resistance interpretation due to its broader use in clinical routine.

## P308

### Quiescent profile of T cells from Colombian MSM with extremely high‐risk sexual behaviours and HIV‐1 specific CTL response

**A Ossa‐Giraldo^1^, Y Blanquiceth^2^, K Contreras^2^, L Flórez^2^, J Hernández^1^ and W Zapata^2^**

^1^Infettare Research Group, Universidad Cooperativa de Colombia, School of Medicine, Medellin, Colombia. ^2^Immunovirología Research Group, Universidad de Antioquia, School of Medicine, Medellin, Colombia

**Background: **MSM still being a key population on HIV‐1 epidemiology [1]. MSM with high‐risk sexual behaviours are at great risk of exposure to infection [2]. Better intervention strategies are urgently needed, such as biomedical research to develop new options for prevention and treatment. The study of seronegative MSM with high‐risk behaviours represents an important opportunity to better understand HIV‐1 infection and immune response to improve the current intervention strategies [3,4].

**Methods: **Analysis of sociodemographic data and basal activation profile and functional response of T lymphocytes against stimuli with HIV‐1, in two groups of MSM from Medellín, Colombia, South America with different sexual behaviours.

**Results: ** We included 44 MSM with high and low risk of exposure (14 and 30, respectively). The high‐risk group presented a higher frequency of sexual partners in the three months prior to the inclusion of the study (Me = 31 vs. Me = 2; *p* < 0.05), sexual partners throughout life (Me = 900 vs Me = 30; *p* < 0.005) and unprotected anal intercourses, showing higher risk behaviours compared to other international MSM cohorts. All subjects are negative for anti‐HIV‐1 antibodies, HIV‐1 proviral DNA and delta 32 mutation in the CCR5 gene in a homozygous state. The individuals at high risk showed a lower percentage of CD4+CD38+ and CD8+CD38+ T cells (*p* < 0.05), a higher percentage of CD4+HLA‐DR+ and CD8+HLA‐DR+ T cells (*p* < 0.05) and lower CD4+Ki‐67 T cells (*p* < 0.05). Moreover, we found four individuals who exhibited a specific response to HIV‐1 by production of TNFα, IFNƔ or both, after overnight stimuli with HIV‐1 Gag peptides. Previously, the lower expression of CD38 has been associated with a lower T cell activation profile and seronegative status; T cells expressing HLA‐DR but not CD38 had been associated with viral control of HIV. The higher expression of Ki‐67 in T cells has been associated with disease progression, while a lower expression has been found in healthy controls.

**Conclusions: ** MSM with greater sexual exposure showed different profile of CD4+ and CD8+ T cell activation compared with the group of low risk. Some MSM showed specific CTL response to HIV‐1 peptides without evidence of infection. Taken together, our results can show a protective profile of HIV‐1 infection in MSM with high‐risk behaviours. It is necessary to continue the study of MSM in high risk of exposure to HIV‐1 to better understand their natural response to the virus and improve the prevention and therapy strategies against HIV‐1.

**References**

[1] Joint United Nations Programme on HIV and AIDS. Country fact sheet, Colombia 2016 [Internet]. 2018. Available from: http://www.unaids.org/es/regionscountries/countries/colombia.

[2] Martinez O, Muñoz‐Laboy M, Levine E, Starks T, Dolezal C, Dodge B, et al. Relationship factors associated with sexual risk behavior and high‐risk alcohol consumption among Latino MSM: challenges and opportunities to intervene on HIV risk. Arch Sex Behav. 2017;46:987‐99.

[3] Kuebler PJ, Mehrotra ML, Shaw BI, Leadabrand KS, Milush JM, York VA, et al. Persistent HIV type 1 seronegative status is associated with lower CD8 +  T‐cell activation. J Infect Dis. 2016;213:569‐73. 

[4] Buchbinder SP, Liu AY. CROI 2018: epidemic trends and advances in HIV prevention. Top Antivir Med. 2018;26:1‐16. 

## P309

### Cost‐effective Sanger sequencing assay for detecting HIV‐1 drug resistance mutations in major group‐M subtypes in resource‐limited settings

**K Clyde, S Chen, A Wong, L Tracy and S Williams**

Genetic Sciences Division, Thermo Fisher Scientific, South San Francisco, CA, USA

**Background: **With the introduction of antiretroviral therapies and the UNAIDS 90‐90‐90 targets comes the need for detecting drug resistance. However, for an HIV‐1 drug resistance assay to be successfully implemented worldwide, it must be both robust and cost effective. To this end, in collaboration with the US Centers for Disease Control and Prevention (CDC), we have introduced a low‐cost Sanger sequencing‐based HIV drug resistance assay for reverse transcriptase (RT) and protease (PR) inhibitors that is specifically designed for resource‐limited settings and a broad range of group‐M subtypes.

**Materials and methods: **HIV‐1 isolates comprising group‐M subtypes A, B, C and D and CRF01_AE and CRF02_AG from plasma, dried blood spots (DBS) or culture were extracted with commercially available extraction kits and tested on the ProFlex, Veriti and GeneAmp 9700 thermal cyclers and 3730‐series, 3500‐series and 3130‐series sequencers using the FastSeq50 sequencing protocol. Data were analyzed using the RECall sequence analysis tool (University of British Columbia) and Stanford University HIVDR Database.

**Results: **The HIV‐1 Genotyping Kit achieved a limit of detection of 1000 copies/mL for plasma and 2000 copies/mL for DBS across all subtypes and CRFs tested. The sensitivity of drug resistance mutation (DRM) detection was 98.1% for plasma and 98.4% for DBS, and specificity was 99.6% for both sample types at a viral load of 10,000 copies/mL. Turnaround time was approximately 14 hours from extracted RNA to results, with less than three hours of hands‐on time. The assay performed well on all thermal cyclers and sequencers tested.

**Conclusions: **The HIV‐1 Genotyping Kit, which includes positive, negative and sequencing controls and pre‐formulated master mixes for amplification and cycle sequencing, is a sensitive, specific and robust method of drug resistance genotyping designed for a broad range of settings and HIV‐1 group‐M genotypes. Moreover, the assay performs well on multiple thermal cyclers and on sequencers from four to 96 capillaries, making it highly flexible, scalable and practical. The low cost, range of subtypes detected and functionality on both new and legacy laboratory equipment make the HIV‐1 Genotyping Kit ideal for use in resource‐limited settings. Future efforts will focus on enabling DRM detection in Integrase, PR and RT in a single assay.

## P310

### Transmission of HIV drug resistance in Tel Aviv, Israel, 2010 to 2017

**D Turner^1^, S Girshengorn^1^, L Tau^1^, D Shasha^1^, S Rapaport^1^, E Katchman^1^ and B Avidor^2^**

^1^Crusaid Kobler AIDS Center, Tel Aviv Sourasky Medical Center, affiliated to the Sackler Faculty of Medicine, Tel Aviv University, Tel Aviv, Israel. ^2^Crusaid Kobler AIDS Center, Tel Aviv Sourasky Medical Center, Tel Aviv, Israel

**Background:** ****Until recently the rate of transmitted drug‐resistance mutations (TDR) was relatively high mainly to NNRTIs. The prevalence of TDRs is regularly evaluated in treatment‐naïve patients in Tel Aviv. This study evaluated the rate and pattern of TDR among HIV‐1 treatment‐naïve patients in Tel Aviv from 2010 to 2017.

**Material and methods**

We analysed TDR mutations prevalence in blood samples of treatment‐naïve patients in Tel Aviv (2010 to 2017). Transmission dynamics were analysed by reconstructing viral phylogenies from *pol* sequences of HIV‐1 subtype A, B and C viruses.

**Results: **Seven hundred and eighty‐four viral sequences revealed 13.1% TDR. Over three‐fourths (77%) of MSM were born in Israel, and 83% harboured subtype B viruses. Other groups include intravenous drug users (IVU), 75% of whom were born in the former Soviet Union countries and 88% of whom harboured subtype A viruses. The heterosexual group was very heterogeneous in origin, including patients born in Israel, Ethiopian immigrants, immigrants from the former Soviet Union and worker immigrants mainly from Africa. NNRTIs TDR was the major class resistance (41.7%) followed by PIs (27.1%) and NRTIs (24.2%) TDR. Phylogenetic analysis of subtype A and B viruses supported clustered transmission of TDR among MSM. These clusters were represented by the mutation K103N in RT and L90M in PR. No integrase inhibitors TDR were found in 2016–2017.

**Conclusion: **TDRs among patients followed in Tel Aviv were represented by clusters in MSM. These clusters contained resistance‐associated mutations to drugs less frequently prescribed in recent years, so their effect on treatment strategy is not straightforward.

## VIROLOGY AND IMMUNOLOGY: OTHER

## P311

### Comparison of viral replication below 50 copies/mL for two‐drug (DTG+RPV) versus three‐drug current antiretroviral regimen (CAR) therapy in the SWORD‐1 and SWORD‐2 studies

**M Underwood^1^, K Angelis^2^, R Wang^1^, B Wynne^3^, E Blair^4^, L Kahl^5^, T Vincent^6^, J Koteff^7^ and M Aboud^8^**

^1^Clinical Virology, Research, ViiV Healthcare, Triangle Park, NC, USA. ^2^Statistics, GlaxoSmithKline, Uxbridge, UK. ^3^Clinical Development, ViiV Healthcare, Project Physician, Dolutegravir, Research Triangle Park, NC, USA. ^4^ViiV Healthcare, Research Triangle Park, NC, USA. ^5^Clinical Development, ViiV Healthcare, Brentford, UK. ^6^Global Scientific Affairs, ViiV Healthcare, Brentford, UK. ^7^Medical Communications, ViiV Healthcare, Research Triangle Park, NC, USA. ^8^Global Medical, ViiV Healthcare, Brentford, UK

**Introduction: **Abbott Realtime assay measures quantitative HIV‐1 RNA viral load (VL) from 40 copies(c)/mL to 10,000,000 c/mL, and generates qualitative target detected (TD) or target not detected (TND) for VL <40 c/mL. FDA Snapshot algorithm uses 50 c/mL as cut‐off. Clinical significance and subject management implications of low level quantitative and qualitative VL data remains controversial. We assessed the number of participants having 40c/mL≤VL<50c/mL and TD/TND over 48 weeks for DTG+RPV two‐drug regimen versus CAR (PI‐, NNRTI‐ or INSTI‐based three‐drug CAR).

**Methodology: **SWORD‐1 and SWORD‐2 are identical open‐label, multicenter, global, Phase III, non‐inferiority studies evaluating efficacy and safety of switching from CAR to DTG+RPV once daily in HIV‐1‐infected adults, with HIV‐1 RNA <50 c/mL (VL<50 c/mL) for at least six months and no history of virologic failure. We explored VL shifts from baseline, cumulative and per visit classification of participants into >50 c/mL, 40 c/mL≤VL<50 c/mL or TD/TND when <40 c/mL, across arms throughout 48 weeks.

**Results: **One thousand and twenty‐four participants were randomized and exposed (DTG+RPV 513; CAR 511) across both studies. At Week 48, 95% of participants in both arms had Snapshot VL <50c/mL in intention‐to‐treat exposed (ITT‐E) population. Confirmed virologic withdrawal rates (CVW) were <1% in both arms. Similar proportion of participants at Week 48 had Snapshot VL <40 c/mL and TND in the two arms (84% vs. 80%, adjusted difference 3.1%, 95% CI ‐2.2% to 8.3%). Participants with baseline TD had similar and low occurrence of at least one VL ≥50c/mL (DTG+RPV 14%; CAR 17%), or at least one VL between 40 c/mL and 50 c/mL (DTG+RPV 4%; CAR 8%). Those participants with baseline TND had similar and low occurrence of at least one VL≥50 c/mL (DTG+RPV 5%; CAR 5%), or a similar percentage of at least one VL between 40 c/mL and 50 c/mL (DTG+RPV 3%; CAR 1%), or at least one VL<40 c/mL and TD (DTG+RPV 44%; CAR 41%) through Week 48. See Table 1.

**Table:** Abstract P311 – Table 1. Changes in quantifiable and non‐quantifiable VL levels by baseline VL category through Week 48

Baseline		DTG+RPV (N = 513)			CAR (N = 511)		
		TND	TD	40 to 50c/mL	TND	TD	40 to 50c/mL
		399 (78%)	98 (19%)	12 (2%)	422 (83%)	76 (15%)	11 (2%)
Post‐baseline	≥50c/mL^a^	21 (5%)	14 (14%)	4 (33%)	22 (5%)	13 (17%)	2 (18%)
	40 ≤ VL50c/mL^a^	12 (3%)	4 (4%)	2 (17%)	5 (1%)	6 (8%)	1 (9%)
	VL40c/mL & TD^a^	177 (44%)	61 (62%)	4 (33%)	172 (41%)	43 (57%)	7 (64%)
	VL40c/mL & TND^b^	189 (47%)	19 (19%)	2 (17%)	223 (53%)	14 (18%)	1 (9%)

Post‐baseline categories are mutually exclusive, with categories on the top to take precedence (categories further up indicate worst baseline VL state). Four participants in DTG+RPV and two in CAR had no post‐baseline viral load data and thus are not included at baseline. ^a^in at least one time point after baseline through Week 48. ^b^in all time points post‐baseline.

**Conclusions: **DTG+RPV was non‐inferior to CAR at Week 48 by Snapshot <50c/mL. The two groups were similar with Snapshot <40c/mL and TND as endpoint. Proportions of TD at baseline and over time were similar between arms with higher rates of TD post‐baseline in those with TD at baseline. Incident viremia (≥40 and ≥50c/mL) was similar between arms by baseline TD versus TND, but was more common with TD. However, this had limited clinical consequence, as efficacy rates were high (95%) and equal between arms and CVW numbers were low and equal between arms.

## P312

### HPV‐mediated cytological abnormalities and high‐risk HPV genotypes associate with altered gut microbiota composition and function in cART‐treated HIV+ males

**E Merlini^1^, C Tincati^1^, A Pandolfo^2^, B Cassani^3^, F Bai^1^, G Ancona^1^, A Barassi^4^, G Bulfamante^3^, A d'Arminio Monforte^1^ and G Marchetti^1^**

^1^Clinic of Infectious Disease, ASST Santi Paolo e Carlo, University of Milan, Health Sciences, Milan, Italy. ^2^Unit of Infectious Diseases, ASST Lecco, Lecco, Italy. ^3^Health Sciences, Pathology Unit, ASST Santi Paolo e Carlo, University of Milan, Milan, Italy. ^4^Health Sciences, Biochemistry Laboratory, ASST Santi Paolo e Carlo, University of Milan, Milan, Italy

**Background: **HIV‐infected individuals feature higher incidence of HPV persistence at both vaginal and anal level. Recently, the gut microbiota has been demonstrated to predict precancerous anal lesions, suggesting that some taxa featuring HIV‐associated dysbiosis might fuel HPV persistence and pathogenesis. Given these premises, we decided to explore whether the presence of HPV‐related cytological abnormalities might be associated with bacteria functional modifications and with HIV‐mediated gut mucosal dysfunction (i.e. increased gut permeability, microbial translocation [MT] and consequent immune activation [IA]) within the anal district of cART‐treated HIV+ males.

**Materials and methods: **We enrolled 36 HIV+ males on cART (HIV‐RNA <40 copies/mL) and collected anal swab, blood and stool samples. Normal cytology: absence of cytological abnormalities; altered cytology: presence of ASCUS, LSIL, HSIL. Lab analyses: (i) anal HPV genotyping and cytological evaluation; (ii) faecal microbiota composition (relative abundance, α‐/β‐diversity); (iii) bacterial metagenome prediction (PICRUSt); (iv) intestinal permeability (Calprotectin, I‐FABP); (v) MT (sCD14, LPS, 16S rDNA); (vi) T‐cell activation (CD8+CD38, CD8+CD38+CD45R0+). Mann‐Whitney, with Bonferroni's correction and chi‐squared tests were used.

**Results: **We identified 30/36 (83%) HPV+ patients, 24/30 harbouring high‐risk HPV genotypes (hrHPV). Of these, 18 had HPV‐related cytological abnormalities w/o neoplasia (aHPV). aHPV patients showed a marked dysbiosis, with higher proportion of *Prevotellaceae* and lower *Leuconostocaceae*. Interestingly, the presence of high‐risk HPV genotypes, irrespective of cytological abnormalities, seemed to have a greater impact on gut dysbiosis, with hrHPV displaying higher proportion of *Prevotellaceae* and *Veillonellaceae*, but lower *Bacteroidaceae, Lachnospiraceae* and *Rikenellaceae* as compared to lrHPV. This shift in bacterial composition was accompanied by changes in predicted metabolic capacity. Indeed, HIV+ patients with HPV‐mediated cytological abnormalities and/or high‐risk HPV genotypes showed increased abundance of genes related to immune system activation and to metabolic syndrome. While the presence of HPV‐mediated cytological abnormalities and high‐risk HPV genotypes associates with gut dysbiosis, we failed to detect any difference in markers of intestinal permeability, MT and IA.

**Conclusions: **In cART‐treated HIV+ males, the presence of HPV‐related cytological abnormalities within the anal district is characterised by unique bacteria composition and functional metagenomic capacity, supporting a pathogenic link between gut microbiota and HPV. From a clinical standpoint, the observations of a *Prevotellaceae*‐rich/*Bacteroidaceae*‐poor profile, coupled with changes in metabolites involved in sustaining immune activation and comorbidities seem to support the establishment of a pro‐inflammatory environment that favours high‐risk HPV genotype persistence and HPV‐mediated cytological abnormalities.

## P313

### Comparison of viral replication for two‐drug (DTG+RPV) versus three‐drug current antiretroviral regimen (CAR) in the SWORD‐1 and SWORD‐2 studies

**R Wang^1^, M Underwood^1^, J Koteff^2^, K Angelis^3^, B Wynne^4^, E Blair^5^, L Kahl^6^, T Vincent^7^ and M Aboud^8^**

^1^Clinical Virology, ViiV Healthcare, Research Triangle Park, NC, USA. ^2^Medical Communications, ViiV Healthcare, Research Triangle Park, NC, USA. ^3^Statistics, GlaxoSmithKline, Uxbridge, UK. ^4^Project Physician, Dolutegravir, ViiV Healthcare, Research Triangle Park, NC, USA. ^5^Clinical Development, ViiV Healthcare, Research Triangle Park, NC, USA. ^6^Clinical Development, ViiV Healthcare, Brentford, UK. ^7^Global Scientific Affairs, ViiV Healthcare, Brentford, UK. ^8^Global Medical Lead, ViiV Healthcare, Brentford, UK

**Background: **The overall goal of HIV therapy is to maintain virologic suppression over the entire course of a patient's treatment. Despite that the clinical significance and subject management of transient ‘blips’ remains controversial, their appearance may lead to concerns about the durability of an ART regimen. Within the SWORD trial we assessed elevated viral loads, including blips during two years of study conduct with the two‐drug regimen (2DR) of DTG+RPV.

**Methodology: **SWORD‐1 and SWORD‐2 are identical open‐label, multicenter, global, Phase III, non‐inferiority studies evaluating efficacy and safety of switching from CAR to DTG+RPV once daily in HIV‐1‐infected adults, with HIV‐1 RNA <50 copies/mL (VL<50c/mL) and no history of virologic failure. Subjects either switch to DTG+RPV on Day 1 (Early Switch [ES] DTG+RPV group) or remain on CAR (CAR group) and switch to DTG+RPV at Week 52 (Late Switch [LS] DTG+RPV group) if still on study and suppressed. FDA Snapshot algorithm uses HIV‐1 RNA 50 copies/mL as the viral suppression cut‐off. Patients with one or more on‐treatment viral loads ≥50c/mL were categorized as either: (1) subjects with viral loads between 50 and 200 copies/mL and no VL ≥200 copies/mL and (2) subjects with at least one VL ≥200 copies/mL. Blips were defined as any VL between 50 and 200 copies/mL preceded and followed by VL below 50 copies/mL.

**Results: **One thousand and twenty‐four participants were randomized and exposed (DTG+RPV 513; CAR 511) across both studies. At Week 100 in the ES DTG+RPV group, 456 (89%) participants had Snapshot VL<50c/mL and six (1.2%) met confirmed virologic withdrawal (CVW) criterion, and in the LS DTG+RPV group, 444 (93%) had Snapshot VL<50c/mL and two (<1%) met CVW criterion. During the first year of exposure to DTG+RPV 34 (6.6%) and 20 (4.2%) participants had blips in the Early and Late Switch groups, respectively. During the second year of follow‐up of the ES DTG+RPV group there were only an additional 3% of subjects with blips.

**Table:** Abstract P313 – Table 1

Category	ES DTG+RPV, Day 1 ‐ Week 52, N = 513	CAR, Day 1 ‐ Week 52, N = 511	ES DTG+RPV, Day 1 ‐ Week 100^a^ N = 513	LS DTG+RPV, Week 52 ‐ Week 100, N = 477
1. Subjects with viral loads between 50 and 200 c/mL and no viral load ≥200 c/mL
1a. At least one single occurrence of VL between 50 and 200 c/mL and adjacent values <50 c/mL (?blips?)	34 (6.6%)	28 (5.5%)	48 (9.4%)	20 (4.2%)
1b. At least two consecutive VL measurements between 50 and 200 c/mL	1 (0.2%)	1 (0.2%)	4 (0.8%)	3 (0.6%)
2. Subjects with at least one viral load ≥200 c/mL
2a. A single viral load ≥200 c/mL and no two consecutive viral loads ≥50 c/mL	2 (0.4%)	5 (1.0%)	5 (1.0%)	5 (1.0%)
2b. Two consecutive viral loads ≥50 c/mL with at least one ≥200 c/mL (may include subjects meeting CVW	2 (0.4%)	3 (0.6%)	6 (1.2%)	4 (0.8%)
Total (all categories)	39 (7.6%)	37 (7.2%)	63 (12.3%)	32 (6.7%)

^a^cumulative events through Week 100.

**Conclusions: **The incidence of blips in the first year after switching to DTG+RPV 2DR was low in both the ES and LS DTG+RPV groups, and comparable to the three‐drug regimen comparator and remained low in the second year. All other categories of VL>50 occurred infrequently in all groups. These results suggest no difference in blip rates or any clinical consequences from VL elevations ≥200 copies/mL, as efficacy rates were high and equal between arms (95% each) and CVW numbers were low DTG+RPV and traditional 3DRs of therapy.

## P314

### Association of new HIV diagnoses within long‐lived transmission clusters from Malaga area (Spain)

**I Viciana Ramos^1^, C González Domenech^2^, G Sena Corrales^1^, C Gómez Ayerbe^1^, M Villalobos^1^, G Ojeda^1^, E Nuño^1^, E Clavijo^1^, R Palacios Muñoz^1^ and J Santos^1^**

^1^UGC Infectious Diseases and Clinical Microbiology, Hospital Virgen de la Victoria, Malaga, Spain. ^2^Microbiology, University of Granada, Granada, Spain

**Background: **The early HIV diagnoses and the decrease of the hidden infections rate are the goals of a proactive partner notification programme (PNP) among new HIV diagnoses (NHIVD), performing at Virgen de la Victoria Hospital (southern Spain) from September 2017. Our aim was the molecular epidemiological determination of the transmission clusters (TC) comprising these NHIVD, clarifying if they are expanding previous TCs in our area or, on the contrary, actively arising new TCs.

**Materials and methods: **We considered all the HIV‐1 genotype resistance tests performed in NHIVD and participating in the PNP in our hospital**,** from January 2017 to June 2018. Drug resistance mutations were determined with Viroseq HIV^®^ system and the partial sequence of HIV‐1 *pol* gene provided phylogenetically compared to a cohort of 451 naïve patients diagnosed between 2004 and 2015 and clustered in 86 TCs previously described. The belonging to a prior TC was based on a phylogenetic criterion as well as using the mean pairwise distance. Therefore, a NHIVD was phylogenetically considered within any cluster if the branch‐support measure (SH‐like aLRT test) was ≥90%. The alignment was done by ClustalX and the phylogenetic reconstruction inferred by maximum likelihood method (FastTree programme). Regarding pairwise distances, they were estimated directly from the sequence alignment (MEGA‐X software package) and the difference of 0.015 substitution/site with any sequence included in a particular TC, as maximum threshold of belonging to such TC.

**Results: **During the study period, we had 59 NHIVD, 41 out of them with *pol* gene sequence available. Among them, 30 (73.2%) were phylogenetically grouped within some TC previously defined, with a mean pairwise‐distance intra‐cluster ≤0.015. Seven out of 30 sequences associated to any prior TC (23.3%) were clustered within the one comprising the CRF19_cpx variant, already described as an outbreak in our area. Seven out of the 41 sequences left (17%) were phylogenetically clustered to others but with SH‐like aLRT ≤90% and pairwise‐distance higher than 0.015. Finally, three of the NHIVD included in the contacts study (7.3%) were associated together and independently from the rest sequences, forming a new TC.

**Conclusions: **Almost three out of four NHIVD included in this study expand previous TC, already defined in our area. The incidence of new TCs is relatively low. The molecular epidemiology of the partner notification programme points out to networks including already diagnosed and under follow‐up patients.

## P315

### HIV‐1 diversity in the Moscow region, Russia: phylodynamics of the most common subtypes

**A Lebedev^1^, N Lebedeva^2^, F Moskaleychik^1^, A Pronin^2^, E Kazennova^1^ and M Bobkova^1^**

^1^N.F. Gamaleya National Research Center of Epidemiology and Microbiology, Ministry of Health, Moscow, Russian Federation. ^2^Moscow Regional AIDS Centre, Ministry of Health of the Moscow Region, Moscow, Russian Federation

**Background: **The Moscow region is the most densely populated subject of the country with an HIV prevalence of about 0.76%. Being a highly developed transport hub the Moscow region is characterised by a high intensity of internal and external migration. Taken together, these factors can cause a wide range of HIV‐1 subtypes in the region, on the one hand, and contribute to the emergence of new recombinant forms of the virus, on the other hand. This is the first large‐scale study in the Moscow region that includes all groups at risk of contracting HIV.

**Materials and methods: **We analysed demographic data and genotypes from 927 HIV‐infected individuals collected in Moscow region in 2011 to 2016. Phylogenetic analysis was performed with maximum‐likelihood and Bayesian coalescent‐based methods. The latter was used to estimated time of the most recent common ancestor and demographic history. Recombination analysis was performed using the jumping profile hidden Markov model algorithm.

**Results: **Our analysis revealed a broad HIV‐1 diversity in Moscow region, including sub‐subtype A6 (85.4%), subtype B (7.4%) and CRF02_AG (1.2%). Forty‐seven (4.2%) unique recombination forms (URF_A6/B) were also found. Other HIV‐1 subtypes were detected as single cases. Among heterosexuals, IDUs and MTCTs, the A6 subtype was most prevalent (>85.0%, in all groups); in MSM group ‐ subtype B (74.4%). The sub‐subtype A6 sequences were combined into the epidemic cluster that arose approximately 1997. Within the subtype B five major epidemic clusters containing 95.0% of the total subtype B sequences were identified. Each cluster contained sequences associated with only one or two dominant transmission routes. The age of these clusters varied widely between 1978 and 1990. Reconstruction of the demographic history of sub‐subtype A6 and B identified at least two epidemic growth phases. Both subtypes displayed the initial phase of exponential growth (subtype A6 ‐ until 2010; subtype B – until 1995), followed by a decrease in the growth rate and the stationary phase approaching the present time.

**Conclusions: **Our study demonstrated a high degree of HIV‐1 subtypes diversity in the Moscow region. We showed that HIV‐1 subtype B introduction in the Moscow region occurred much earlier than HIV‐1 A6 subtype, which agreed well with the known HIV‐1 epidemiological history in the region. Despite the different age of subtypes A6 and B populations, the rate of growth of epidemics caused by them stabilises, which may be the result of ARV therapy and/or preventive measures.

## P316

### Effects of immune checkpoint inhibitors on HIV reservoirs and responses in patients with HIV‐associated cancers

**J Forni^1^, A Dalla‐Pria^1^, W Ma^2^, M Liu^2^, N Immani^2^, J Evans^1^, T Newson‐Davis^1^, C Brock^1^, X Xu^2^ and M Bower^1^**

^1^National Centre for HIV Malignancy, Chelsea and Westminster Hospital, London, UK. ^2^Centre for Immunology and Vaccinology, Imperial College London, London, UK

**Background: **Immune checkpoint inhibitors including anti‐PD1 antibodies and anti‐CTLA4 antibodies are effective therapies for cancers. Latent reservoir cells infected with HIV express PD‐1 and are potential targets for these antibodies. A recent publication reported a decline in total HIV DNA in a patient treated with nivolumab for HIV‐associated lung cancer [1].

**Methods: **Peripheral blood samples were collected from HIV‐positive patients on antiretroviral therapy with undetectable plasma HIV RNA, who were receiving immune checkpoint inhibitor (ICI) therapy for cancer. Samples were collected before and after three cycles of ICI therapy (two months). HIV latent reservoirs were assayed by quantitative PCR for total HIV DNA and by Alu‐gag PCR for integrated proviral HIV DNA. Anti‐HIV T‐cell responses were evaluated by interferon gamma ELISPOT.

**Results: **Three patients were enrolled (one melanoma, two NSCLC). The patient with melanoma was treated with a combination of ipilimumab (anti‐CTLA4) and nivolumab (anti‐PD1), whilst the other two patients were treated with pembrolizumab (anti‐PD1). The baseline CD4 median was 376/mm^3^ and all had undetectable plasma HIV viral loads. After three cycles of ICI therapy, no changes in CD4 counts or percentages were observed. Similarly, there were no significant changes in the HIV reservoirs as measured by total HIV DNA (per PBMC or per CD4 cell) and integrated HIV DNA (per PBMC or per CD4 cell). ELISPOT assays using patient PBMCs and pooled HIV‐derived peptides will be presented.

**Conclusions: **Despite positive effects on HIV reservoirs reported elsewhere in one patient, we observed no significant effects on latent reservoirs in three patients.

**Reference: **[1] Guihot A, Marcelin AG, Massiani MA, Samri A, Soulie C, Autran B, et al. Drastic decrease of the HIV reservoir in a patient treated with nivolumab for lung cancer. Ann Oncol. 2018;29:517‐18.

## P317

### Western blot in treated people with HIV‐1 chronic infection: frequency of negative HIV‐1 pol genes

**F Rinaldi^1^, S Rolla^2^, L Galli^3^, P Andrea^3^, A Bigoloni^3^, C Muccini^4^, A Mastrangelo^4^, S Nozza^3^, C Tavano^2^, M Clementi^5^, A Lazzarin^3^, A Bartoloni^6^ and A Castagna^7^**

^1^Department of Experimental and Clinical Medicine, University of Florence, Florence, Italy. ^2^Laboratory of Microbiology and Virology, IRCCS San Raffaele Scientific Institute, Milan, Italy. ^3^Infectious Diseases, IRCCS San Raffaele Scientific Institute, Milan, Italy. ^4^Infectious Diseases, Vita‐Salute San Raffaele University, Milan, Italy. ^5^Laboratory of Microbiology and Virology, Vita‐Salute San Raffaele University, Milan, Italy. ^6^Infectious and Tropical Diseases Unit, Department of Experimental and Clinical Medicine, University of Florence, Florence, Italy. ^7^Infectious Diseases, Vita‐Salute San Raffaele University, IRCCS San Raffaele Scientific Institute, Milan, Italy

**Background: ** The aim of this study was to evaluate immune responses against HIV‐1 gag, env and pol genes in adult people with HIV‐1 chronic infection, with long exposure to ART.

**Materials and methods: ** Retrospective analysis on all adult people with HIV‐1 chronic infection, followed at the Infectious Diseases Clinic of the San Raffaele Hospital, Milan, Italy, with a western blot (WB) test performed after at least 12 months of ART (if multiple WB tests per patient were available, the most recent was considered). Patients’ characteristics at WB determination described by median (quartiles) or frequency (%) and compared by Mann‐Whitney test or chi‐square test. A multivariate logistic regression, including age, gender, HCV co‐infection, years of ART, change in CD4+ at WB since ART start (CD4+ slope), years of HIV‐RNA <50 copies/mL and months to ART start was performed to assess factors associated with a negative HIV‐1 pol.

**Results: ** Five hundred and thirty patients were included: 72% male, 89% Italian; at WB, they were infected with HIV since 19.3 years (14.1 to 24.6), on ART since 16.3 years (10.4 to 19.1), 39% started ART ≤6 months since HIV diagnosis, the median age was 51 years (47 to 56), pre‐ART CD4+ count 317/µL (177–471), CD4+ count 632/µL (443 to 878), CD4+/CD8+ ratio 0.33/µL (0.18 to 0.52), 92% had HIV‐RNA <50 copies/mL. A negative HIV‐1 pol was found in 88 (16.6%) patients; these patients were slightly older (*p* = 0.0007), had a longer duration of HIV‐RNA <50 copies/mL (*p* = 0.046), a shorter time to ART start (*p* = 0.010) and a better immunological profile (CD4+: *p* = 0.009; CD4+/CD8+ ratio: *p* = 0.0006) than those with a positive HIV‐1 pol. Changes in CD4+ and CD4+/CD8+ ratio since ART start tended to be higher in subjects with a negative versus positive HIV‐1 pol; pre‐ART CD4+ and CD4+/CD8+ ratio were similar between patients with a negative versus positive HIV‐1 pol gene (CD4+: *p* = 0.487; CD4+/CD8+ ratio: *p* = 0.314). By multivariate logistic regression, a negative HIV‐1 pol gene was associated with an early ART start (adjusted odds ratio [aOR] per 3‐months longer 0.95 [95% CI 0.90 to 0.99], *p* = 0.012) and a greater CD4+ recovery since ART start (aOR per 100‐cells/µL higher 1.11 [95% CI 1.01 to 1.24], *p* = 0.049).

**Conclusions: ** A negative HIV‐1 pol was found in around 17% of HIV‐1 infected subjects with a long exposure to ART. This finding is associated with an early ART start and a better immunological profile. The relationship between ART efficacy, recognition of the pol gene by the immune system and the production of specific antibodies need to be investigated.

## P318

### Transient viral load increase in HIV‐1 infected patients treated with the cobicistat‐boosted darunavir regimen in an Italian observational, multicentre, prospective study (the TMC114FD1HTX4003 / ST.O.RE. study)

**S Rusconi^1^, D Ripamonti^2^, A Gori^3^, A Antinori^4^, M Palma^5^, D Mancusi^5^, A Uglietti^5^ and R Termini^5^**

^1^Division of Infectious Diseases, DIBIC Luigi Sacco, Infectious Diseases Unit, Milan, Italy. ^2^ASST Papa Giovanni XXIII, Bergamo, Italy. ^3^Infectious Diseases Unit, IRCCS Ca’ Granda Foundation Ospedale Maggiore Policlinico, Milan, Italy. ^4^HIV/AIDS Department, National Institute for Infectious Diseases Lazzaro Spallanzani IRCCS, Rome, Italy. ^5^Medical Affairs, Janssen‐Cilag SpA, Cologno Monzese, Italy

**Background: **Treatment guidelines recommend HIV‐1 RNA suppression below the limit of detection of the assay (generally <50 copies/mL) as a key goal of antiretroviral therapy. Transient viral load (TVL) increase, i.e. >50 copies/mL, are observed in otherwise successfully treated patients. We evaluated the rate and the effect of TVL increase during the ST.O.RE. study, an Italian prospective, single‐arm, multicentre, non‐interventional study in patients switching from ritonavir‐boosted protease inhibitor to darunavir/cobicistat (DRV/c)‐based therapy, in order to collect data from clinical practice.

**Materials and methods: **All available HIV‐1 RNA data, collected in patients on treatment, were included throughout 48 weeks of observation. TVL increase was defined as HIV‐1 RNA values ≥50 copies/mL both single or confirmed measurements occurring anytime during the 48 weeks whether or not leading to virology failure. We also analysed the CD4 cell count, CD8 cell count and CD4/CD8 ratio in patients showing TVL increase to observe potential change in immunological parameters. Continuous data were presented as median and interquartile range (IQR). Comparisons between groups were performed using Wilcoxon rank‐sum test and correlation analyses were performed by calculating Pearson coefficient. All tests were two sided and a *p* value <0.05 was considered as statistically significant.

**Results: **All patients were virosuppressed at baseline. Among 336 enrolled patients, 59% and 34% were on triple and dual regimen, respectively. A total of 18 patients (11 were on triple therapy) showed a TVL increase over the study. All of them were Caucasian; 12 were male. We observed TVL increase >1000 copies/mL in eight patients (Group 1) while TVL between 50 and 1000 copies/mL (Group 2) was reported in 10. Reported reasons for TVL increase were virological blip (a single measurement between 50 and 1000 copies/mL) in nine patients, non‐adherence (VL >50 copies/mL and/or >1000 copies/mL in single or consecutive measurements during declared non‐adherence period) in seven, virological failure in one, drug‐drug interactions in one. We analysed comparison between TVL increase and immunological status when available. CD4 cell count and CD4/CD8 ratio decrease were statistically significant in patients with TVL >1000 copies/mL as compared to those with TVL <1000 copies/mL (Table 1). We found a negative correlation, albeit barely significant, between TVL and CD4/CD8 change (rho = −0.50; *p* = 0.056).

**Table:** Abstract P318 – Table 1. Comparisons between Group 1 and Group 2

	Group 1 HIV RNA >1000 copies/mL median (IQR) n = 8	Group 2 HIV RNA <1000 copies/mL median (IQR) n = 10	*p* for comparison^a^
HIV RNA	5836 (24,270)	90.5 (91.0)	0.0004
Mean (SD)	21,747 (34,413)	116 (61)
CD4 pre	725 (520)	560 (459)
CD4 post	322 (302)	560 (459)
CD4 change	‐280 (270)	‐53 (149)	0.01
CD8 pre	890 (711)	859 (231)
CD8 post	831 (482)	922 (749)
CD8 change	‐142 (662)	‐46 (782)	0.39
CD4/CD8 pre	0.68 (0.21)	0.48 (0.49)
CD4/CD8 post	0.40 (0.31)	0.50 (0.96)
CD4/CD8 change	‐0.195 (0.394)	0.0 (0.07)	0.001

^a^Wilcoxon rank sum test. SD = standard deviation.

**Conclusions: **In our study, TVL increase occurred infrequently, throughout 48 weeks. TVL increase (>1000 copies/mL) appears to affect the immunological status with possible increase of immune activation and inflammation.

## P319

### Persistent outbreak of the HIV‐1 CRF19_cpx variant in treatment‐naïve MSM patients in Malaga area (Spain)

**I Viciana Ramos^1^, C González Domenech^2^, M Mayorga^3^, J De La Torre Lima^4^, C Gómez Ayerbe^1^, M Castaño^3^, A Del Arco^4^, R Palacios Muñoz^1^ and J Santos^1^**

^1^UGC Infectious Diseases and Clinical Microbiology, Hospital Virgen de la Victoria, Malaga, Spain. ^2^Microbiology, University of Granada, Granada, Spain. ^3^Infectious Diseases Unit, Hospital Carlos Haya, Malaga, Spain. ^4^Infectious Diseases Unit, Hospital Costa del Sol, Marbella, Spain

**Background: **During the period 2011 to 2016, the HIV‐1 CRF19_cpx variant emerged as an outbreak in newly HIV diagnoses (NDVIH) in southern Spain. Our aim was to determine the current status of this outbreak, analysing the new cases of this variant in our area and their epidemiological relationship with the previous ones.

**Materials and methods: **We considered all the HIV‐1 genotype resistance tests performed in NDVIH at Virgen de la Victoria Hospital, reference centre in southern Spain**,** from January 2017 to June 2018. Drug resistance mutations were determined with Viroseq HIV^®^ system and the partial sequence of HIV‐1 *pol* gene provided submitted to REGA v.3.0 for subtyping. Sequences assigned as CRF19_cpx subtype were phylogenetically compared to the 254 reference sequences of the same variant retrieved from the LANL, as well as to the 55 ones comprising the already described CRF19_cpx variant outbreak. The alignment was done by ClustalX and the phylogenetic reconstruction inferred by maximum likelihood method (PhyML v.3.0 programme). The cluster reliability was supported on the value of SH‐like aLRT test. The resistance mutations were predicted using Stanford algorithm v.7.1.1. We also collected demographic, clinical and immunovirological data.

**Results: **During the study period, 523 resistance studies were performed in NDVIH; 12 (2.3%) had sequences consigned in REGA as subtype CRF19_cpx. All the new cases conformed a very well‐defined transmission cluster (*aLRT*=92%) with the CRF19_cpx sequences from the previous outbreak, already comprising up to 67 patients. Eight of the new sequences were clustering within two subclusters previously defined: E and F, currently including 18 and three patients, respectively. We have not found the G190A mutation in any of the new sequences. The new cases of the CRF19_cpx were MSM, with an average age of 32.5 years (IQR 27.1 to 43.6) and Spaniards, except one Italian patient. Half of them were seroconverters (mean seroconversion time of 17.0 months, 8.3 to 81.3). The initial T‐lymphocyte CD4 count was 423 cells/μL (200 to 562) and initial viral load was 4.9 log copies/mL (4.6 to 5.2).

**Conclusions: **All the new cases of the CRF19_cpx variant emerged in our area during 2017 and half this year are phylogenetically clustered with the previous outbreak, pointing out its active status. The NDVIH infected with this variant possess similar epidemiological, clinical and immunovirological characteristics to those already included in the outbreak. None of the new sequences of this subtype showed the G190A mutation. The active transmission of the CRF19_cpx variant in our area should warn us about the necessity of intense epidemiological surveillance programmes.

## P320

### HIV‐1 subtype diversity and international travel in Romanian people who inject drugs

**R Jipa^1^, S Paraschiv^2^, L Banica^2^, D Otelea^2^, E Manea^1^, I Nicolae^2^, A Abagiu^1^ and A Hristea^1^**

^1^Clinical Department, National Institute for Infectious Diseases “Prof Dr Matei Bals”, Bucharest, Romania. ^2^Molecular Diagnostics Laboratory, National Institute for Infectious Diseases “Prof Dr Matei Bals”, Bucharest, Romania

**Background: **Although subtype F1 is highly prevalent in Romania, increases in international travel have led to increased detection of other subtypes in newly diagnosed HIV patients [1].

**Objective: **Describe clinical and epidemiological patterns as correlated with recent history of international travel in newly diagnosed IVDUs with HIV and to assess how travel contributes to the spread of different subtypes.

**Methods: **We retrospectively studied 270 IVDUs diagnosed with HIV infection between October 2012 and September 2017. Epidemiological, demographic and clinical data were collected through a questionnaire survey. We excluded patients with missing data regarding travel history and/or available HIV *pol* sequences. Subtype analysis was done using Rega HIV‐1 subtyping tool v2.0 and further phylogenetic analysis was performed with FastTree. Differences between groups were analysed using the Mann‐Whitney U test for continuous variables and the chi‐square test for dichotomous variables.

**Results: **Sixty‐nine patients were excluded from the study. Out of 201 patients included, 92 (46%) had a recent history of international travel. The characteristics of patients from the two groups are shown in Table 1. Travellers who used intravenous drugs and had unprotected sex during travel were infected in a higher proportion with non‐F1 subtypes (73% vs. 61%, *p* = 0.162, OR [95% CI] 2.69 [0.7 to 10.31], and 38% vs. 36%, *p* = 0.798, OR [95% CI] 1.25 [0.45 to 3.41]). Travel to Spain was reported more frequently for IVDUs infected with F1 strains than for those infected with other subtypes (52% vs. 46%, *p* = 0.6, OR [95% CI] 0.74 [0.26 to 2.08]). More IVDUs with non‐F1 subtypes reported travel to Greece as compared to IVDUs with F1 subtype (*p* = 0.002, OR [95% CI] 6.71 [2.02 to 22.25]). Phylogenetic analysis indicated that IVDUs sequences are clustering together, regardless of their travelling status. The sequences of travelling IVDUs are intermixed in the phylogenetic tree with the sequences of non‐travelling IVDUs. For subtype B, the sequences from IVDUs who reported travelling abroad are more diverse and intermixed with reference sequences.

**Table:** Abstract P320 – Table 1. Epidemiological, clinical and laboratory characteristics in travellers versus non‐travellers

	International travellers N = 92	Non‐travellers N = 109	*p* OR [95% CI]
Male, N (%)	78 (85)	91 (84)	0.84 0.91 [0.42 to 1.94]
Age (years), median (IQR)	31 (26 to 34)	30 (26 to 35)	0.75
In prison, N (%)	63 (68)	56 (56)	0.01 2.05 [1.15 to 3.66]
HIV stage: A, N (%)	48 (52)	78 (72)	0.09 0.42 [0.16 to 1.07]
HIV stage: B, N (%)	11 (12)	7 (6)	0.07 2.53 [0.92 to 6.97]
HIV stage: C, N (%)	33 (36)	24 (22)	0.01 1.97 [1.06 to 3.71]
Chronic hepatitis B, N (%)	1 (1)	10 (9)	0.02 0.12 [0.01 to 1.01]
Chronic hepatitis C, N (%)	91 (99)	105 (96)	0.62 2.6 [0.26 to 25.44]
CD4 T‐cell count (cells/mm^3^), median (IQR)	404 (246 to 508)	402 (256 to 575)	0.91
Heroin abuse, N (%)	88 (96)	100 (92)	0.38 1.98 [0.58 to 6.65]
New psychoactive substances abuse, N (%)	80 (87)	100 (92)	0.35 0.6 [0.24 to 1.49]
HIV subtype F1, N (%)	66 (72)	82 (75)	0.63 0.83 [0.44 to 1.56]
HIV subtype B, N (%)	5 (5)	2 (2)	0.25 3.07 [0.58 to 16.23]
HIV subtype CRF14_BG, N (%)	14 (16)	14 (13)	0.68 1.21 [0.54 to 2.71]
HIV subtype CRF14_F1, N (%)	5 (5)	3 (3)	0.47 2.03 [0.47 to 8.73]
HIV other recombinant subtypes, N (%)	2 (2)	7 (6)	0.18 0.32 [0.06 to 1.59]

**Conclusions: **Regardless of the history of international travel, non‐F1 subtypes were found in similar proportions. Among travellers, drug use and unprotected sex were reported especially in patients with non‐F1 subtypes. Non‐F1 subtypes were associated with travelling to Greece. Phylogenetic analysis also suggested international transmission events for CRF14_BG and B subtypes.

**Reference: **[1] Niculescu I, Paraschiv S, Paraskevis D, Abagiu A, Batan I, Banica L, et al. Recent HIV‐1 outbreak among intravenous drug users in Romania: evidence for cocirculation of CRF14_BG and subtype F1 strains. AIDS Res Hum Retroviruses. 2015;31:488‐95. 

